# Pezizomycetes Genomes Reveal Diverse P450 Complements Characteristic of Saprotrophic and Ectomycorrhizal Lifestyles

**DOI:** 10.3390/jof9080830

**Published:** 2023-08-06

**Authors:** Nomfundo Ntombizinhle Nsele, Tiara Padayachee, David R. Nelson, Khajamohiddin Syed

**Affiliations:** 1 Department of Biochemistry and Microbiology, Faculty of Science and Agriculture, University of Zululand, KwaDlangezwa 3886, South Africa; nomfundonsele2@gmail.com (N.N.N.); teez07padayachee@gmail.com (T.P.); 2 Department of Microbiology, Immunology and Biochemistry, University of Tennessee Health Science Center, Memphis, TN 38163, USA

**Keywords:** cytochrome P450 monooxygenases, Pezizomycetes, saprophytic, mycorrhizal fungi, genome data mining, phylogenetic analysis, secondary metabolite biosynthetic gene clusters

## Abstract

Cytochrome P450 monooxygenases (CYPs/P450s) are heme proteins that play a role in organisms’ primary and secondary metabolism. P450s play an important role in organism adaptation since lifestyle influences P450 composition in their genome. This phenomenon is well-documented in bacteria but less so in fungi. This study observed this phenomenon where diverse P450 complements were identified in saprophytic and ectomycorrhizal Pezizomycetes. Genome-wide data mining, annotation, and phylogenetic analysis of P450s in 19 Pezizomycetes revealed 668 P450s that can be grouped into 153 P450 families and 245 P450 subfamilies. Only four P450 families, namely, CYP51, CYP61, CYP5093, and CYP6001, are conserved across 19 Pezizomycetes, indicating their important role in these species. A total of 5 saprophyte Pezizomycetes have 103 P450 families, whereas 14 ectomycorrhizal Pezizomycetes have 89 P450 families. Only 39 P450 families were common, and 50 and 64 P450 families, respectively, were unique to ectomycorrhizal and saprophytic Pezizomycetes. These findings suggest that the switch from a saprophytic to an ectomycorrhizal lifestyle led to both the development of diverse P450 families as well as the loss of P450s, which led to the lowest P450 family diversity, despite the emergence of novel P450 families in ectomycorrhizal Pezizomycetes.

## 1. Introduction

The fungal class Pezizomycetes forms a monophyletic group, yet Pezizomycetes have diverse lifestyles, including saprophytic, mycorrhizal, and parasitic [1]. Species in this class have significant scientific, ecological, and economic importance (Table 1). Some species have been used as model organisms to understand the development of multicellular structures, rehabilitation of post-fire soils, and as an income source, as many species in this class are truffles with great economic importance (Table 1). General information on some of the Pezizomycetes species focusing on their importance is listed in Table 1.

Some Pezizomycetes genomes were sequenced to understand the molecular mechanisms underlying the transition from saprophytic to mycorrhizal lifestyle [2]. The authors discovered that transitions from saprotrophic to symbiosis involve (i) loss of lignin and cellulose-degrading genes, (ii) the ancestral genes gaining novel functions, (iii) new, lineage-specific symbiotic gene diversity, (iii) multiplication of transposable elements, and (v) diverse genetic innovations behind the ectomycorrhizal guild’s convergent origins [3,4].

**Table 1:** Information about various Pezizomycetes and their well-known characteristics.

Species Name	Family	Lifestyle (Well-Known/Common Name)	General Information	Reference
*Ascobolus immersus* RN42	*Ascobolaceae*	Saprotroph (coprophilous fungus)	This fungus lives on herbivore dung and is used as a model fungus for epigenetic research.	[4]
*Ascodesmis nigricans* CBS 389.68	*Ascodesmidaceae*	Saprotroph (coprophilous fungus)	This fungus lives on both omnivorous and herbivore dung and is ideal for studying the complex multicellular structure in ascomycetes.	[5]
*Choiromyces venosus* 120613-1	*Tuberaceae*	Ectomycorrhizal truffle (pig truffle)	This symbiotic species coexists with coniferous and deciduous plants on clayey soils. Because of the potent and distinctive order of the fruiting body, different European regions place different values on the gourmet attributes of this white truffle.	[4]
*Kalaharituber pfeilii* F3	*Pezizaceae*	Ectomycorrhizal truffle (Kalahari or desert truffle)	This desert truffle is a food and economical source for the people who live in the dry regions of Southern Africa, which range from South Africa’s Northern Cape Province through Botswana, Namibia, and Angola. The truffle is remarkably resistant to harsh desert conditions. This truffle is the only one to create ectomycorrhizal relationships with dicot and monocot plants. It demonstrates extraordinary adaptability to harsh desert conditions.	[3]
*Morchella importuna* CCBAS932	*Morchellaceae*	A saprotrophic morel	This fungus belongs to the true morel fungi and lives in pre- and post-fire environments. This fungus maintains the fertility of the site and stabilizes the soil after a fire. Despite being widely prized as edible species, cultivation has proven difficult.	[4]
*Morchella importuna* SCYDJ1-A1	*Morchellaceae*	A saprotrophic morel	This species can be artificially grown in typical agricultural soil; thus, a popular variety of gourmet mushrooms contributes significantly to the global economy.	[6]
*Peziza echinospora* CBS 144458	*Pezizaceae*	Saprotroph (pyrophilous fungus)	This fungus is a moderate-size cup fungus with a contrast in color between its upper and lower surfaces. It strictly grows in post-fire environments and is thus an ideal candidate to study its enzymatic abilities.	[7]
*Pyronema confluens* CBS100304	*Pyronemataceae*	Saprotroph	It is a saprobe that lives in the soil and is found in temperate forests. After a forest fire, its fruiting bodies typically appear on the ground. This fungus serves as a model for investigating cell biology and forming fruiting bodies in filamentous ascomycetes.	[8]
*Pyronema domesticum* CBS 144463	*Pyronemataceae*	Saprotroph (pyrophilous fungus)	This fungus grows rapidly on post-fire soils and also on sterilized materials.	[7]
*Pyronema omphalodes* CBS 144459	*Pyronemataceae*	Saprotroph (pyrophilous fungus)	This fungus grows rapidly on post-fire soils.	[7]
*Terfezia boudieri* ATCC MYA-4762	*Terfeziaceae*	Ectomycorrhizal truffle (desert truffle)	This desert truffle has been an important food since dating back to 4000 years in the arid areas of the Middle East.	[4]
*Terfezia claveryi* T7	*Terfeziaceae*	Ectomycorrhizal truffle (desert truffle)	This desert truffle has been an important food in the Mediterranean Basin, Near East, and Middle East. It has a pleasant flavor, an unusual texture, significant antioxidant activity, and antibacterial properties.	[3]
*Tirmania nivea* G3	*Pezizaceae*	Ectomycorrhizal truffle (desert truffle)	It is one of the most appreciated desert truffles in the north of Africa, the Near East, and the Middle East. It grows to a diameter of more than 10 cm, has a mild flavor and a fungal odor, and is highly prized in the market. The heat and water stress this species can withstand in deserts is exceedingly unfavorable for other fungus.	[3]
*Tricharina praecox* CBS 144465	*Pezizaceae*	Saprotroph (pyrophilous fungus)	This fungus grows only on post-fire soils.	[7]
*Tuber aestivum* var. *urcinatum*	*Tuberaceae*	Ectomycorrhizal truffle (Burgundy truffle)	Burgundy truffle, summer truffle, and scorzone are all names for the edible fruiting bodies that are produced by tuber aestivum. This truffle is widely distributed from Morocco to Sweden in the north and from Ireland to Kazakhstan.	[4]
*Tuber borchii* Tbo3840	*Tuberaceae*	Ectomycorrhizal truffle (the white truffle or bianchetto)	Due to its highly prized gourmet qualities, this ectomycorrhizal ascomycete is regarded as the tuber species with the broadest biological distribution in Europe. It is growing in popularity as an Italian delicacy. *T. borchii* is one of the most extensively researched truffle species because it is amenable to laboratory manipulations.	[4]
*Tuber brumale*	*Tuberaceae*	Ectomycorrhizal truffle (the winter truffle)	This species is widespread in Europe, and its edible fruiting body (truffle) is harvested during the winter.	[9]
*Tuber indicum*	*Tuberaceae*	Ectomycorrhizal truffle (the Chinese black truffle)	At an elevation of 2.000 to 2.500 m in a temperate climate, this ectomycorrhizal Ascomycota forms a mutualistic association with oak and mountain pines in the Chinese provinces of Yunnan and Sichuan, and it has unintentionally spread to North America and Italy.	[9]
*Tuber magnatum*	*Tuberaceae*	Ectomycorrhizal truffle (the white truffle—the icon of European gastronomy)	This white truffle, a “cult food,” is a well-known symbol of European cuisine and culture. *T. magnatum*’s fruiting body is an edible truffle (also known as a hypogeous ascocarp) prized for its exquisite organoleptic qualities (i.e., taste and perfumes). In Italian and Balkan soils, it is generally found as mycelia. It forms a mutualistic mycorrhizal connection with the roots of deciduous trees such as poplars, oaks, and willows.	[4]
*Wilcoxina mikolae* CBS 423.85	*Pyronemataceae*	Ectomycorrhizal fungus	This fungus is a significant ectomycorrhizal symbiont of Pinaceae and numerous hardwood species. *Wilcoxina* species are among the most frequent colonizers of young pine, spruce, and larch trees and are found in nurseries and in forests that have experienced a fire or other disturbance.	[3]
*Sphaerosporella brunnea* Sb_GMNB300	*Pyronemataceae*	Ectomycorrhizal	This fungus is considered a vital pioneer ectomycorrhizal symbiont due to its ability to associate with diverse trees and shrub species.	[10]
*Trichophaea hybrida* UTF0779	*Pyronemataceae*	Ectomycorrhizal	This species is distributed throughout Northern and Central Europe and predominantly inhabits old forests, contrary to the *Wilcoxina* species.	[3]
*Tuber melanosporum* Mel28	*Pyronemataceae*	Ectomycorrhizal (Périgord black truffle)	This species is native to Southern Europe, and its fruiting body (truffle) is one of the most expensive edible mushrooms in the world.	[4,11]

Note: The lifestyle of different Pezizomycetes was retrieved from the published articles [3,4].

Most of the genomic analysis studies on Pezizomycetes focused on different sets of gene families, and one important gene family, cytochrome P450 monooxygenases (CYPs/P450s), needs to be covered. P450s have heme as a prosthetic group and are present in species belonging to all different kingdoms, including viruses and archaea [12,13,14]. Although these enzymes are called monooxygenases, research has demonstrated that they perform a variety of enzymatic processes with regio- and stereo-selectivity [15,16,17]. Their remarkable catalytic capabilities prompted researchers to investigate these enzymes’ applicability in all areas of biology [18,19,20,21,22].

P450s have a unique nomenclature and classification scheme [23,24,25,26]. The nomenclature scheme starts with the prefix “CYP” for cytochrome P450 monooxygenase, followed by an Arabic numeral indicating the family, a capital letter indicating the subfamily, and an Arabic digit indicating the individual P450 in a family. As part of the annotation/classification criterion, all P450s with >40% identity belong to the same family, and all P450s with >55% identity belong to the same subfamily.

In fungi, P450s are known to play a role in their primary and secondary metabolism and detoxification or degradation of xenobiotics [27]. Housekeeping tasks such as ergosterol biosynthesis, meiotic spore-wall biogenesis, and n-alkane and fatty acids hydroxylation are examples of primary fungal metabolism [27,28]. The involvement of several fungal P450s in the biosynthesis of secondary metabolites, polyketides, non-ribosomal peptides, terpenes, and other substances has been well-reviewed [27,29,30]. Fungal P450s may also detoxify and degrade a wide range of xenobiotic chemicals found in their surroundings, including polycyclic aromatic hydrocarbons (PAHs), phenolic compounds, and other hazardous environmental contaminants [28,31,32]. Fungal P450s were found to be an excellent drug target. CYP51, also known as sterol 14-demethylase, is the most conserved P450 across biological kingdoms and is the primary target of conventional antifungal azole drugs [20,33]. Studies indicated that the fungal P450 family CYP53 potentially serves as a unique alternative antifungal therapeutic target [34,35].

Based on the analysis of bacterial P450s, authors have proposed that P450s play a crucial role in an organism’s adaptation vis-à-vis the lifestyle of organisms, which impacts the P450 content in their genome [36]. This phenomenon has also been reported with fungal P450s, in a few cases distinct P450 families and subfamilies bloomed (are present in many copies within the same species) to help fungi adapt to ecological niches [35,37,38]. The CYP53 family bloomed in basidiomycetes due to its involvement in forming the wood-degrading oxidant veratryl alcohol and aiding the breakdown of wood-derived chemicals [35]. CYP53′s different role appears to have enriched this P450 family by significantly duplicating its members in Basidiomycete genomes (paralogous evolution) [35]. Analysis of putative P450s in Basidiomycete biotrophic plant pathogens revealed the presence of unique P450 families, possibly reflecting the characteristics of their order [37]. Compared to other Agaricomycotina saprophytes, the CYP63, CYP5037, CYP5136, CYP5137, and CYP5341 P450 families were expanded in *Armillaria mellea*, as were the CYP5221 and CYP5233 P450 families in *Puccinia graminis* and *Melampsora laricis-populina* [37]. The presence of distinct P450 families in these biotrophic plant pathogens demonstrates how a host can shape an organism’s P450 composition. The authors concluded that these distinct P450 family members may play an important role in the host’s successful infection [37].

Considering the importance of P450s, especially their role in adaptation to diverse ecological niches, it is essential to elucidate the P450 profiles in Pezizomycetes to understand P450s’ role in the transition from saprophytic to mycorrhizal lifestyles, if any. Thus, this study aimed to perform genome-wide data mining, annotation, and phylogenetic analysis of P450s to identify distinct P450 profiles, if any, between saprophytes and mycorrhizal Pezizomycetes.

## 2. Materials and Methods

### 2.1. Species and Databases

Nineteen Pezizomycetes were used in the study (Table 2). All the species’ genomes used in the study have been published and are available for public use at the Joint Genome Institute MycoCosm portal [2].

### 2.2. Genome Data Mining and Identification of P450s

Genome data mining and identification of P450s in Pezizomycetes was carried out following the protocol previously published by our laboratory [39,40]. Each Pezizomycetes genome was searched for P450s using the InterPro code “IPR001128”. The hit protein sequences were downloaded and searched for P450 characteristic motifs, including the EXXR and CXG motifs [41,42]. Proteins with all P450 characteristic motifs were considered P450s, and proteins with one of these motifs or short in amino acid length (less than 350 amino acids) were considered P450 fragments. Proteins considered P450s were subjected to P450 family and subfamily analysis.

### 2.3. Assigning P450 Family and Subfamily

To assign P450 families and subfamilies, we performed a Basic Local Alignment Search Tool (BLAST) analysis of Pezizomycetes P450s against all named fungal sequences on the Cytochrome P450 Homepage [26] to identify the percentage identity with named homolog P450s. The proteins were then grouped into different P450 families and subfamilies following the International P450 Nomenclature criteria [24,25], i.e., proteins with >40% and >55% amino acid identity were grouped under the same P450 family and subfamily. Proteins with less than 40% identity to the named homologs were assigned to the new P450 family. The P450s along with their assigned names and P450 fragment sequences are presented in Supplementary Dataset S1.

### 2.4. Phylogenetic Analysis

Phylogenetic analysis of P450s was carried out following the procedure previously published by our laboratory [14,43]. Briefly, the P450 protein sequences were aligned by the MAFFT v6.864 [44] program available at the T-REX web server [45]. The alignments were then automatically subjected to interpret the best tree using the Trex web server [45]. Finally, the best-inferred tree was visualized, colored, and generated by the Interactive Tree Of Life (iTOL) [46].

### 2.5. P450 Family Conservation Analysis

The presence or absence of P450s belonging to different families in Pezizomycetes was shown with heat maps generated using P450 family data following the method described by our laboratory [40,43]. The data were represented as −3 for gene absence (green) and 3 for gene presence (red). A tab-delimited file was imported into a multi-experiment viewer (MeV) [47]. Hierarchical clustering using a Euclidean distance metric was used to cluster the data. A total of 19 Pezizomycetes form the vertical axis, and 153 P450 families form the horizontal axis.

### 2.6. Identification of P450s That Are Part of Natural Metabolite Biosynthetic Gene Clusters

P450s, part of natural metabolite biosynthetic gene clusters (BGCs), were identified following the procedure published by our laboratory [36] with slight modification. Each of the fungal genome National Center for Biotechnology Information (NCBI) genome accession numbers (Table 3) was submitted for BGCs analysis at the Antibiotics and Secondary Metabolite Analysis Shell (anti-SMASH) program [48]. Anti-SMASH results were downloaded in gene cluster sequences and Excel spreadsheets representing species-wise cluster information. P450s that formed part of a specific gene cluster were identified by manual data mining of gene cluster sequences. Standard gene cluster abbreviation terminology available at the anti-SMASH database [48] was maintained in this study. Among the 19, only 12 Pezizomycetes NCBI genome accession numbers were successful at the anti-SMASH program, and the remaining seven species accession numbers have yet to provide any results. Thus, we presented information on BGCs for twelve species in this study.

## 3. Results and Discussion

### 3.1. Saprotrophs Have More P450s Than Ectomycorrhizal Pezizomycetes

Genome-wide data mining of P450s in nineteen Pezizomycetes resulted in 779 hit proteins (Table 4). Further analysis of hit proteins for characteristic P450 motifs (as indicated in Section 2.2) revealed that not all hit proteins are P450s. Among hits, 668 hits have all the P450 characteristic motifs and are thus considered P450s, 88 were P450 fragments, 7 were false positives, and 16 were different proteins, thus noting them as no hits. The presence of false positives and no hits indicates that automated allocation of P450s is not always accurate, and manual curation of P450 is needed to assess an accurate number of P450s in an organism.

The number of P450s in 19 Pezizomycetes ranged from 17 to 58 P450s with an average of 35 P450s. Among Pezizomycetes, *Ascobolus immersus* RN42 has the highest number of P450s (58), and *Terfezia claveryi* T7 has the lowest number of P450s (17) in their genomes (Table 4). *Tuber borchii* Tbo3840, despite having the highest number of hit proteins due to 19 P450 fragments, the P450 count was confined to only 55 P450s, second only to *A. immersus* (Table 4).

A comparison of P450s revealed that saprotrophic Pezizomycetes have more P450s in their genome compared to ectomycorrhizal Pezizomycetes (Table 4). The average number of P450s was 41 in saprotrophs compared to 33 in ectomycorrhizal Pezizomycetes (Table 4). The difference observed concerning P450 numbers is not statistically significant due to one or two species being outliers from saprophytes and ectomycorrhizal groups. However, the average P450s number difference indicates that most ectomycorrhizal have fewer P450s than saprophyte Pezizomycetes.

### 3.2. P450 Family and Subfamily Analysis in Pezizomycetes

**Figure 1:**
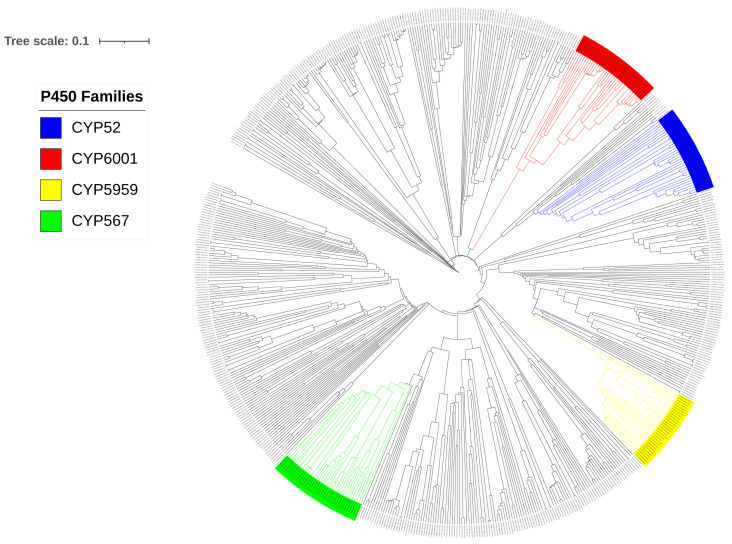
Phylogenetic analysis of Pezizomycetes P450s. P450 families that are populated in Pezizomycetes are highlighted in different colors. A high-quality figure is presented in Supplementary Figure S1.

Following the International P450 Nomenclature Committee rules and the phylogenetic analysis (Figure 1), the 668 P450s found in 19 Pezizomycetes were grouped into 153 P450 families and 245 P450 subfamilies (Table 5 and Table S1). Although P450s were assigned to different P450 families and subfamilies based on the percentage identity, as indicated in Section 2.3, phylogenetic analysis is critical in assigning the subfamilies to P450s that fall to around 55% identity, borderline with the named homolog P450s. Based on the alignment on the phylogenetic tree, these borderline P450s were assigned to the correct subfamilies. Furthermore, phylogenetic analysis will also help find evolutionary relationships, such as the closeness of P450s from two different species.

Among 153 P450 families found in 19 Pezizomycetes, only 4 P450 families, namely, CYP567, CYP6001, CYP52, and CYP5959, have ≥30 members. Thus, one can safely say that P450 families in Pezizomycetes are not bloomed (a few P450 families with many genes) (Table 5). This is unlike some fungal species where P450 family blooming is common [35,37,38,49]. This indicates that the Pezizomycetes species have a high P450 diversity concerning P450 families in their genome. Analysis of P450 subfamilies revealed the blooming of two P450 subfamilies, A and C, in the CYP5959 and CYP6001 families (Table 5 and Table S1).

The number of P450 families ranged from 40–14, with an average of 26 P450 families in 19 Pezizomycetes, where *Wilcoxina mikolae* CBS 423.85 and *T. claveryi* T7 had the highest and lowest number of P450 families in their genome (Table 6 and Table S2). The number of P450 subfamilies ranged from 49 to 16, with an average of 31 P450 families. *A. immersus RN42* and *T. claveryi* T7 had the highest and lowest number of P450 families in their genomes (Table 6 and Table S2). Detailed analysis of P450 families and subfamilies in 19 Pezizomycetes is presented in Table S2.

**Figure 2:**
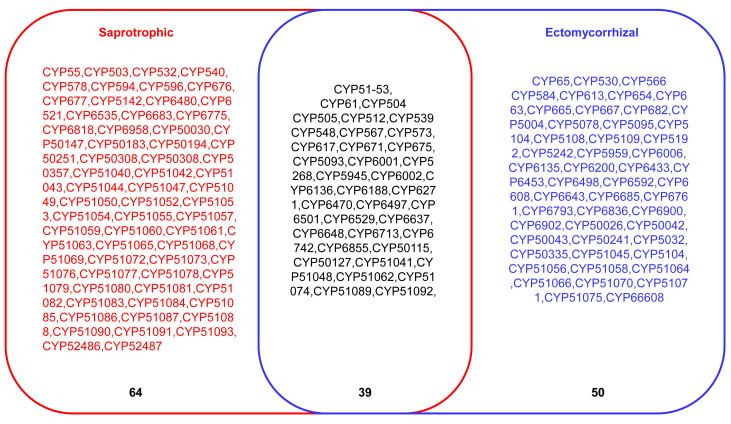
Comparative analysis of P450 families between saprotrophic and ectomycorrhizal Pezizomycetes. The number indicates the total number of P450 families.

A comparison of P450 families and P450 subfamilies indicated that saprophytes have more P450 families in their genome than ectomycorrhizal Pezizomycetes (Table 6). A total of 5 saprophyte Pezizomycetes have 103 P450 families, whereas 14 ectomycorrhizal Pezizomycetes have 89 P450 families (Figure 2). A total of 39 P450 families were found in common between saprotrophs and ectomycorrhizal Pezizomycetes (Figure 2). Furthermore, 50 and 64 P450 families were found to be unique to ectomycorrhizal and saprophytic Pezizomycetes (Figure 2).

### 3.3. A Few P450 Families Are Conserved in Pezizomycetes

**Figure 3:**
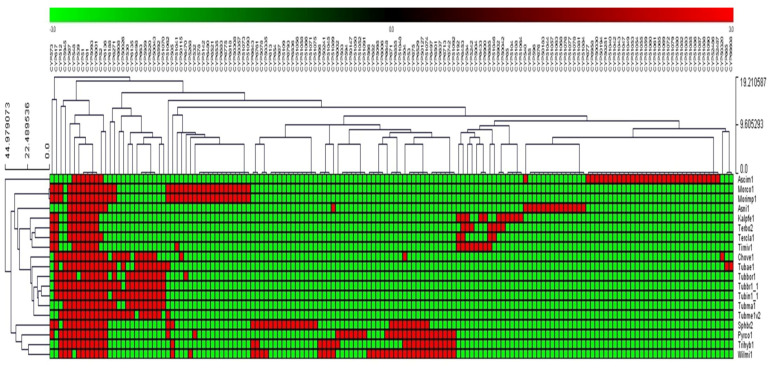
P450 family conservation analysis in 19 Pezizomycetes. The heat map represents the presence (red) or absence (green) of the P450 family in Pezizomycetes. Pezizomycetes form the vertical axis, and P450 families form the horizontal axis. A detailed analysis of P450 family conservation in Pezizomycetes is presented in Table S3.

Analysis of P450 family conservation revealed that out of 153 P450 families, only 4 P450 families, CYP51, CYP61, CYP5093, and CYP6001, were conserved across 19 Pezizomycetes (Figure 3). CYP539 and CYP548 were found to be conserved in 18 Pezizomycetes, followed by CYP567 in 17 species and CYP52 in 16 species (Figure 3 and Table S3). A detailed analysis of P450 family conservation across the 19 Pezizomycetes is presented in Table S3. Conservation of four P450 families across 19 Pezizomycetes indicates that these P450 families might be involved in critical functions. It is well-known that CYP51 and CYP61 are involved in sterol biosynthesis [33,50,51], the essential components of cell wall membranes, and CYP6001 members were also shown to be involved in the oxidation of fatty acids [52]. The reactions performed by these three P450 families are important in the physiology of these species, and thus, these families are conserved. The function of CYP5093 family members is not identified, and, thus, based on its conservation, one can assume it might be involved in critical functions as well.

### 3.4. Terpene Biosynthetic Gene Clusters Are Dominant in Pezizomycetes

**Figure 4:**
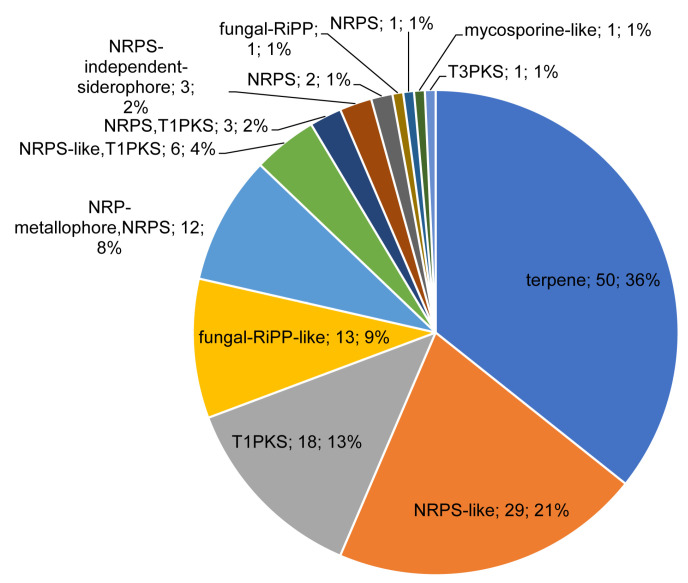
Comparative analysis of natural metabolite biosynthetic gene clusters (BGCs) in 19 Pezizomycetes. The number next to the BGCs indicates the number of BGCs and their percentage in the total number of BGCs. Standard abbreviations representing the BGCs as indicated in anti-SMASH (antibiotics and Secondary Metabolite Analysis Shell) [48] were used in the figure. Detailed information is presented in Table S4.

A natural metabolite biosynthetic gene clusters (BGCs) analysis across 12 Pezizomycetes revealed the presence of 142 clusters belonging to 13 cluster types (Figure 4 and Table S4). Among the BGC types, terpene was dominant with 50 clusters, followed by NRPS-like with 29 clusters and T1PKS with 18 clusters (Figure 4 and Table S4). An analysis of most similar BGCs revealed that six Pezizomycetes have a terpene BGC that has 100% similarity to clavaric acid, and one species has an NRPS, T1PKS BGCs that have 100% similarity to ACT-Toxin II (Table S5) indicating that these BGCs are certainly involved in the production of these metabolites.

Comparative analysis of BGCs revealed the highest number of BGCs in *W. mikolae* CBS 423.85 (21 BGCs), followed by *Sphaerosporella brunnea* (18 BGCs) and *Trichophaea hybrida* (16 BGCs) (Table 7). The average number of BGCs in 12 Pezizomycetes was 12. Some BGCs in a few species were found to have P450s (Table 7), indicating the possible involvement of P450s in the synthesis of natural metabolites, as fungal P450s are known to be involved in the production of various natural metabolites [28,30]. A total of nine P450s were found to be part of different BGCs, with five of them being part of the terpene BGC (Table 7). CYP6637B2 was found to be common between *T. hybrida* and *W. mikolae* CBS 423.85 as part of a terpene BGC (Table 7), indicating that this P450 is involved in the production of a terpene metabolite in both species. Compared to the number of P450s found in Pezizomycetes (668 P450s), the number of P450s (9 P450s) present in the BGCs seems to be very low (only 1%). This suggests that most of the Pezizomycetes P450s possibly play a role in primary metabolism.

## 4. Conclusions

In this post-genomic era, understanding the molecular basis behind fundamental aspects such as adaptation to diverse ecological niches by organisms is gaining momentum. This study observed that ectomycorrhizal Pezizomycetes have the lowest number of P450s, P450 families, and P450 subfamilies compared to saprotrophic Pezizomycetes. Furthermore, we also identified the development of many unique P450 families in ectomycorrhizal Pezizomycetes. Our study results strongly support previous studies that show that the transition from saprophytic to ectomycorrhizal lifestyle resulted in the loss of specific gene complements and enrichment of novel genes in Pezizomycetes, indicating genome-level changes for adaptation [3,4]. This phenomenon seems universal as it was observed in bacteria [36,53,54,55,56,57] and a few fungal species [35,37,38,43,58] where the transition from saprophytic to pathogenic or simple lifestyles resulted in the loss of P450s or the development of unique P450s. More fungal genomes from different fungal groups need to be investigated to obtain conclusive evidence on changes in P450 complements between saprotrophs and mycorrhizal lifestyle. Furthermore, future research will involve identifying the role of P450s in adaptation, especially the unique P450 families of ectomycorrhizal Pezizomycetes.

## Figures and Tables

**Table 2:** Pezizomycetes used in the study and their genome database links, along with reference articles, are listed in the table. The genome database of Pezizomycetes was accessed on 31 May 2023.

Species Name	Genome Version	Genome Database Link	Reference
*Ascobolus immersus* RN42	v1.0	https://mycocosm.jgi.doe.gov/Ascim1/Ascim1.home.html	[4]
*Ascodesmis nigricans* CBS 389.68	v1.0	https://mycocosm.jgi.doe.gov/Ascni1/Ascni1.home.html	[5]
*Choiromyces venosus* 120613-1	v1.0	https://mycocosm.jgi.doe.gov/Chove1/Chove1.home.html	[4]
*Kalaharituber pfeilii* F3	v1.0	https://mycocosm.jgi.doe.gov/Kalpfe1/Kalpfe1.home.html	[3]
*Morchella importuna* CCBAS932	v1.0	https://mycocosm.jgi.doe.gov/Morco1/Morco1.home.html	[4]
*Morchella importuna* SCYDJ1-A1	v1.0	https://mycocosm.jgi.doe.gov/Morimp1/Morimp1.home.html	[6]
*Pyronema confluens* CBS100304		https://mycocosm.jgi.doe.gov/Pyrco1/Pyrco1.home.html	[8]
*Sphaerosporella brunnea* Sb_GMNB300	v2.0	https://mycocosm.jgi.doe.gov/Sphbr2/Sphbr2.home.html	[10]
*Terfezia boudieri* ATCC MYA-4762	v1.1	https://mycocosm.jgi.doe.gov/Terbo2/Terbo2.home.html	[4]
*Terfezia claveryi* T7	v1.0	https://mycocosm.jgi.doe.gov/Tercla1/Tercla1.home.html	[3]
*Tirmania nivea* G3	v1.0	https://mycocosm.jgi.doe.gov/Tirniv1/Tirniv1.home.html	[3]
*Trichophaea hybrida* UTF0779	v1.0	https://mycocosm.jgi.doe.gov/Trihyb1/Trihyb1.home.html	[3]
*Tuber aestivum* var. *urcinatum*	v1.0	https://mycocosm.jgi.doe.gov/Tubae1/Tubae1.home.html	[4]
*Tuber borchii* Tbo3840	v1.0	https://mycocosm.jgi.doe.gov/Tubbor1/Tubbor1.home.html	[4]
*Tuber brumale*	v1.0	https://mycocosm.jgi.doe.gov/Tubbr1_1/Tubbr1_1.home.html	[9]
*Tuber indicum*	v1.0	https://mycocosm.jgi.doe.gov/Tubin1_1/Tubin1_1.home.html	[9]
*Tuber magnatum*	v1.0	https://mycocosm.jgi.doe.gov/Tubma1/Tubma1.home.html	[4]
*Tuber melanosporum* Mel28	v1.2	https://mycocosm.jgi.doe.gov/Tubme1v2/Tubme1v2.home.html	[11]
*Wilcoxina mikolae* CBS 423.85	v1.0	https://mycocosm.jgi.doe.gov/Wilmi1/Wilmi1.home.html	[3]

**Table 3:** Information on Pezizomycetes and their NCBI genome accession numbers used to analyze natural metabolite biosynthetic gene clusters in the anti-SMASH database [48].

Species Name	NCBI Genome Accession Number
*Ascobolus immersus*	PZQT00000000.1
*Tuber borchii*	NESQ00000000
*Terfezia claveryi*	WHUX00000000
*Wilcoxina mikolae* CBS 423.85	WITH00000000
*Tirmania nivea*	WHUY00000000
*Tuber brumale*	JACCEG00000000
*Tuber melanosporum*	CABJ00000000.1
*Trichophaea hybrida*	WHVE00000000
*Tuber indicum*	JACCEH00000000
*Sphaerosporella brunnea*	VXIS00000000
*Morchella importuna* SCYDJ1-A1	SSHS00000000.1
*Tuber magnatum*	DYWC00000000.1

**Table 4:** Genome-wide analysis of P450s in 19 Pezizomycetes.

Species Name	Lifestyle	Total Hits	P450s	No Hits	False Positive	Fragments
*Ascobolus immersus* RN42	SAP	63	58	4	1	0
*Ascodesmis nigricans* CBS 389.68	SAP	31	28	2	0	1
*Morchella importuna* CCBAS932	SAP	40	37	3	0	0
*Morchella importuna* SCYDJ1-A1	SAP	41	37	3	0	1
*Pyronema confluens* CBS100304	SAP	55	44	1	2	8
*Choiromyces venosus* 120613-1	ECM	52	33	1	1	17
*Kalaharituber pfeilii* F3	ECM	35	32	0	0	3
*Sphaerosporella brunnea* Sb_GMNB300	ECM	49	47	0	0	2
*Terfezia boudieri* ATCC MYA-4762	ECM	24	19	1	2	2
*Terfezia claveryi* T7	ECM	19	17	0	0	2
*Tirmania nivea* G3	ECM	21	19	0	0	2
*Trichophaea hybrida* UTF0779	ECM	44	37	0	0	7
*Tuber aestivum* var. *urcinatum*	ECM	31	29	0	0	2
*Tuber borchii* Tbo3840	ECM	74	55	0	0	19
*Tuber brumale*	ECM	37	32	0	0	5
*Tuber indicum*	ECM	39	35	0	0	4
*Tuber magnatum*	ECM	32	27	0	1	4
*Tuber melanosporum* Mel28	ECM	35	30	0	0	5
*Wilcoxina mikolae* CBS 423.85	ECM	57	52	1	0	4

Note: The lifestyle of different Pezizomycetes is retrieved from the published articles [3,4]. Abbreviations: SAP: saprotrophic; ECM: ectomycorrhizal.

**Table 5:** Analysis of P450 family and subfamily count in Pezizomycetes. The name of P450 families (F), their count (C), and the number of subfamilies (NSF) within a P450 family are presented in the table. A detailed analysis of the P450 families and subfamilies is presented in Table S1.

F	C	NSF	F	C	NSF	F	C	NSF	F	C	NSF
CYP567	38	12	CYP6608	4	1	CYP51075	2	2	CYP51066	1	1
CYP6001	35	3	CYP6637	4	2	CYP51079	2	2	CYP51068	1	1
CYP52	34	13	CYP6648	4	2	CYP51085	2	2	CYP51071	1	1
CYP5959	31	1	CYP6761	4	3	CYP51093	2	1	CYP51072	1	1
CYP548	24	2	CYP6855	4	4	CYP5142	2	1	CYP51076	1	1
CYP51	20	1	CYP50115	3	2	CYP5242	2	1	CYP51077	1	1
CYP5093	19	3	CYP50335	3	2	CYP540	2	1	CYP51078	1	1
CYP61	19	1	CYP5078	3	2	CYP578	2	1	CYP5108	1	1
CYP539	18	1	CYP51041	3	2	CYP6002	2	1	CYP51080	1	1
CYP6135	18	1	CYP51048	3	3	CYP6480	2	1	CYP51081	1	1
CYP6136	15	4	CYP51069	3	3	CYP6535	2	1	CYP51082	1	1
CYP617	15	4	CYP51083	3	3	CYP666	2	1	CYP51084	1	1
CYP663	14	2	CYP51089	3	2	CYP6683	2	1	CYP51086	1	1
CYP512	13	4	CYP51092	3	2	CYP6775	2	1	CYP51087	1	1
CYP5945	12	6	CYP5192	3	2	CYP6818	2	1	CYP51088	1	1
CYP6220	11	1	CYP5268	3	1	CYP6900	2	1	CYP5109	1	1
CYP51070	10	1	CYP532	3	2	CYP6958	2	1	CYP51090	1	1
CYP573	9	2	CYP584	3	2	CYP5004	1	1	CYP51091	1	1
CYP6271	9	1	CYP6470	3	1	CYP50147	1	1	CYP52486	1	1
CYP6713	8	6	CYP6497	3	2	CYP50183	1	1	CYP52487	1	1
CYP50043	7	1	CYP6521	3	2	CYP503	1	1	CYP55	1	1
CYP504	7	2	CYP6643	3	1	CYP5095	1	1	CYP566	1	1
CYP51062	6	3	CYP6685	3	1	CYP5104	1	1	CYP594	1	1
CYP530	6	1	CYP671	3	1	CYP51040	1	1	CYP596	1	1
CYP6188	6	1	CYP6742	3	2	CYP51043	1	1	CYP6006	1	1
CYP6498	6	1	CYP6902	3	2	CYP51047	1	1	CYP613	1	1
CYP6592	6	1	CYP50030	2	1	CYP51049	1	1	CYP65	1	1
CYP50194	5	2	CYP50042	2	2	CYP51050	1	1	CYP654	1	1
CYP505	5	1	CYP50241	2	1	CYP51053	1	1	CYP665	1	1
CYP675	5	2	CYP50308	2	1	CYP51054	1	1	CYP66608	1	1
CYP50026	4	2	CYP50320	2	1	CYP51055	1	1	CYP667	1	1
CYP50127	4	3	CYP50357	2	1	CYP51056	1	1	CYP676	1	1
CYP50251	4	2	CYP51042	2	2	CYP51058	1	1	CYP677	1	1
CYP51074	4	3	CYP51044	2	1	CYP51059	1	1	CYP6793	1	1
CYP53	4	1	CYP51045	2	1	CYP51060	1	1	CYP682	1	1
CYP6433	4	1	CYP51046	2	1	CYP51061	1	1	CYP6836	1	1
CYP6453	4	1	CYP51052	2	2	CYP51063	1	1			
CYP6501	4	1	CYP51057	2	1	CYP51064	1	1			
CYP6529	4	1	CYP51073	2	1	CYP51065	1	1			

**Table 6:** Comparative analysis of P450 families and subfamilies in Pezizomycetes. A detailed analysis of P450 families and subfamilies for each Pezizomycetes is presented in Table S2.

Species Name	No of P450 Families	No of P450 Subfamilies
*Ascobolus immersus* RN42	36	49
*Ascodesmis nigricans* CBS 389.68	24	26
*Choiromyces venosus* 120613-1	24	29
*Kalaharituber pfeilii* F3	20	22
*Morchella importuna* CCBAS932	33	36
*Morchella importuna* SCYDJ1-A1	33	36
*Pyronema confluens* CBS100304	37	43
*Sphaerosporella brunnea* Sb_GMNB300	38	45
*Terfezia boudieri* ATCC MYA-4762	16	18
*Terfezia claveryi* T7	14	16
*Tirmania nivea* G3	17	19
*Trichophaea hybrida* UTF0779	31	36
*Tuber aestivum* var. *urcinatum*	24	26
*Tuber borchii* Tbo3840	22	27
*Tuber brumale*	24	27
*Tuber indicum*	24	28
*Tuber magnatum*	21	23
*Tuber melanosporum* Mel28	23	25
*Wilcoxina mikolae* CBS 423.85	40	49

**Table 7:** Comparative analysis of natural metabolite gene clusters and P450s in the clusters in 12 Pezizomycetes. Detailed information is presented in Table S4.

Species Name	Number of Clusters	Clusters with P450	Cluster Type	P450(s) Part of the Cluster
*Ascobolus immersus*	14			
*Morchella importuna*	12			
*Sphaerosporella brunnea*	18	2	Terpene	CYP654C8, CYP667F1
		11	Fungal-RiPP	CYP51F1
		12	NRPS	CYP5109B1, CYP6836A1
		16	NRPS	CYP613S1
*Terfezia claveryi*	8			
*Tirmania nivea*	9			
*Trichophaea hybrida*	16	14	Terpene	CYP6637B2
*Tuber borchii*	8			
*Tuber brumale*	8			
*Tuber indicum*	10			
*Tuber magnatum*	10			
*Tuber melanosporum*	8			
*Wilcoxina mikolae* CBS 423.85	21	9	Terpene	CYP51048A1
		11	Terpene	CYP6637B2

Note: Standard abbreviations representing the BGCs as indicated in anti-SMASH (Antibiotics and Secondary Metabolite Analysis Shell) [48] were used in the table.

## Data Availability

Not applicable.

## Supplementary Materials

The following supporting information can be downloaded at: https://www.mdpi.com/article/10.3390/jof9080830/s1: Figure S1. A high-resolution figure of the phylogenetic analysis of P450s of Pezizomycetes. P450 families that are populated in Pezizomycetes are highlighted in different colors. Table S1. Analysis of P450 families and subfamilies in 19 Pezizomycetes. Table S2. Comparative analysis of P450 families and subfamilies in Pezizomycetes. Table S3. P450 family conservation analysis in 19 Pezizomycetes. Table S4. Comparative analysis of natural metabolite biosynthetic gene clusters (BGCs) in 12 Pezizomycetes. BGCs and P450s part of these BGCs were predicted as indicated in Section 2.6. Standard abbreviations representing the BGCs as indicated in anti-SMASH (Antibiotics and Secondary Metabolite Analysis Shell) [48] were used in the table. Supplementary Dataset S1. P450s identified and annotated in Pezizomycetes are presented with their assigned name, followed by protein ID from the Joint Genome Institute MycoCosm database as indicated in Table 2 and species code. P450 fragments identified in Pezizomycetes are also listed.
