# Supplementary material for: Pezizomycetes Genomes Reveal Diverse P450 Complements Characteristic of Saprotrophic and Ectomycorrhizal Lifestyles
Source: J Fungi (Basel). 2023 Aug 6;9(8):830. doi: 10.3390/jof9080830 (PMC10455484; doi:10.3390/jof9080830)
Supplement: Supplementary file 1 [file jof-09-00830-s001.zip › Supplementary Dataset S1.pdf]

Article

Pezizomycetes Genomes Reveal Diverse P450 Complements Characteristic of Saprotrophic and Ectomycorrhizal Lifestyles

Nomfundo Ntombizinhle Nsele [1], Tiara Padayachee [1], David R. Nelson [2,*] and Khajamohiddin Syed [1,*]

[1] Department of Biochemistry and Microbiology, Faculty of Science and Agriculture, University of Zululand, KwaDlangezwa 3886, South Africa; nomfundonsele2@gmail.com (N.N.N.); teez07padayachee@gmail.com (T.P.)

[2] Department of Microbiology, Immunology and Biochemistry, University of Tennessee Health Science Center, Memphis, TN 38163, USA

* Correspondence: drnelson1@gmail.com (D.R.N.); syedk@unizulu.ac.za (K.S.); Tel.: +1-901-448-8303 (D.R.N.); +27-0359026857 (K.S.)

Supplementary Dataset S1

P450s identified and annotated in Pezizomycetes are presented with their assigned name, followed by protein ID from the Joint Genome Institute MycoCosm database as indicated in Table 2 and species code. P450 fragments identified in Pezizomycetes are also listed.

Wilcoxina mikolae CBS 423.85 v1.0

>CYP51045A2|539162|Wilmi1

MATVNTTNLLTITVVAALASHHFIFCRNELDKYSAHVLSAGVISHLFLTIALRHASVPSAYLLSLSLSAFVYVLTVFTSIL

IYRIWFHPLRNFPGPFMARTTSFWQVFTNIKSDFHWRDVSKKLQEEYGDFVRTGPRELTVFDARATVALNGVQSKVMKGP

WYEGVGPNLMGIRDKEVHKKRRRVWDQAFSMKSLTDLAPIMDQQTDILLQRFSESVDAGKNVCVRDWCFFYSYDVMSVLA

FGTCLDLLKSAENRWVSELVHDAMAVFGILAPCMWVMHTLKLIPGMAEGLMRMERYSMEVVRKRKAMKHPKPDLFKYLID

GGLTEKILQLEARLIIILAGSDTTSATLTLALLHLALYPSIQQKLRASILESFPDHRSENLLSTKCDYLEAVITEIIRIHP

AIPPGLQRLTPKEGCWIGSTFIPGDTLVFASTYNIQRDPRYFVKPDGFIPERWTTSPELVIDKTAFAPFSTGPMGCVGRN

VALMEMRGVLMKTLRLYEIATTEGFDREEYFGRVQDKFTTTLPGLELGFRRLRE*

>CYP51074B1|541357|Wilmi1

MDLLTTHPYISILLGILILSTLGSTVSLVKNIRYAKTTGLNYIVYPLTSGGFSLIILTALRPVRRLLLVLPQKWQDYFNI

ATFGAHWRVKGRMTERYGGVYLTVSAARVACFVSDAEAANEIMSDRERFPKPVEAYETLTVFGPNIVATEGREWAHHRKH

AVAPFGERNYKLVWKESLQQAADMVETWEAKSYQPDTPNNCMSLALHVISAAGFGIPMKFADTEEKPVEEVELFTDGHKP

PKGFSFSFRQALQFITANMLQHALIIRLIPRWIPKEWFSYTHMHQQAHTDFSRYLREMVAKSHASPGGTSNLLDLLVAAE

KTSDDAALSLTELMGNIFIFTVAGHETTAQSLHYAFLMMALHPEMQQWVCDGIDKALEGQSRDIQKWKYEEVFPKLVTPL

CLMLETLRLFPVVPYIPKCTGESGTTLTYGGRTVHLRPMYIAVSATSVQRSPAYYGPSAGVFDPTRWDATNTNSFLSKNK

GQQGLMAAGLEYPTIHKPERGAFFPFSDRARGCLGRKFAQVTFVAVMAVVFRDLKVRIKELEGETREMTENRVWRAVGES

YATLSLRVVESVPLVFERR*

>CYP567W3|544416|Wilmi1

LVKGIYNLFFHPLAKLPGPRYAAFSQLYFSYVTLSRRYPFIIKELHDKYGPVVRVSPNQVSFDSASSWKDIYGHVGGRKP

FLKSDLYDSDARPKSIVTIRDPVEHGAMRKLLSNAFSAKALAGQESIVHIYVDLLVKQIGKHATGKPEGEGMVKWYNWCT

FDIIGDLAFGDPFGCLEAGVPHFWVSAILDSIKVGAYNAMLIKYIGNSKPALALKKLFVPKHLMAQRERHFGYGRDKMMK

RMNNPNSTRKDFMSNILSEKESREISVGALTVHGSLLIVAGSETTAAFLSATTYYLCRTPHAYKKLVDEIRSKFATYEDI

TNQNTEKCTYLKAVIDEGLRSYPPLPFGLGRDSPGETVDGIFIPEGTEVFTSPWASTHSEANFHRPYEFLPERWIDKDCT

DKKEASRPFLLGTRVCLGRNLAYLEMRVILAKMLWVYDMELKNEKLDWIAESDCHVFWKKPELRVNFTRRDGICVPPLDD

DAPPVVA*

>CYP5093H3|545796|Wilmi1

MYGSLVTVWAGRRPTIIIGDPKVACDLLDRRSAIYSSRPRFVVMGELFTNNDSLLTMPHGDKWRKTRKIFHSGLLRKACE

TYKPIQEAETQRLAHALLHTPEHFGRHLERYAASVMVCVAYGRRVDDLDDGDVRRIYQRMQYMSTLNVPGAYWAESFPLL

KLVPDCLAPWKREVKQQAESSSRLLLDLAQKVRNRMSDNTAPPSFTKTLWERFDANPGELTEREVAYATGSLFGAGSDTS

SSTLMSFILAMTCFPDVARKAQEELDRVVGGDRFPTWSDEPDLPYCRAVITETLRWRPVAVMGGTPHASIRDDYYEGHFI

PKGTTILGNLWAIHHNEKYFKNSHDFVPERYLEAQEGTVPYPHRDGHSAFGWGRRICPGKQLAENSLFISITRILWGFHI

RKSIDPATGKEDEYNIFAYTDGFNSKPQPFRCVIKPRSDQIREVIERGAKLGERFLESYN*

>CYP6136D1|552330|Wilmi1

MLGNLILGLLHAVSVFCKNHWLLISIACVGYQLYRRAVRYSQSPMKKQGIPGPFLAGFTCWYRAYYANIKRNWHAKLVEL

HEEYGPIVWIAPDEVSVSDPKLRSVLYGFADERKEESFFPKSKSFETGLFNEDFNFVFETDPARARLGKYALSHPYSEKG

LVRLEHNFDEAVQEFTQGFKEKVASNDKVHCFSDWTHYFMFDLATLLMAGYSNGLCRAGKDEGGAIRALRVIFNVVGSLV

PVPFALTVSTKFIRKSILNGKLEHLFRWGICYNNGESEKKDRINEVADKLPHNLMSKFRDGEKKIRKLFPKGNWTEAITN
NVFFIYAGSMVASNALPLVIKLIYSHPEVLSKVREELADLPREVKIDDITHHNGKCALPYLEATILEALRLSPTFGLSLG
RVVPSIGCQLNEYYIPPGYVVSMSGWATNVNKEYFGEDAKEFRPERWIGNHPTEIAKDGTGEPRTMRNYIEAGWFTFGAG
SRVCIGRHLSMIAFVKFVGNFVRQFDLEIVNEGYLWYGLIQHTEEMMVKAKVRDSNAVEVPVVEVPVQAEPAVVTAEN*
>CYP666C1|560811|Wilmi1
MFLTTPEIIGYIPTVFQAAAIAFILWYLLALLWNYYIASSAGIPVIIVPFYYQNILCVNWPYIPGLAWIVNNLLPSSTAS
YISDIGFTTRFWLRGQRFKTQGLCYFTVSPRNIVLNVADADVVNQIMSDREGFPKPAHKYKIIDMYGSNLLTASDEDWPH
HRRYIAGAFNEKNNNLVWEETLQQSGQMLSIWKSRTAANTTDPMVIDTLNYDVMTMALHVLSSAAFGIPLYFTEATTKRP
EAEQGAGHEIFLDTEQPPIGFTMTYRQSILFISQNISSTVGAVMFLPSWMKVVFKHQFAAHRNLGNYLRSIVELAGRGET
QAKSKYGQDSLLSLMVRATQEDSASRRAGKESGKGFTTQELMGNLFIFTVAGHESTGITLHYTLINLALNPEVQDWLCAE
IDEVLADQPSDPELWSYDLFPKLVAPLCVMLETLRLYPPVLSVPKWTGSSPQPITYSGTTHTLPPKTSIVLSGIGLHYNP
TYWGSDASTFDPLRWDASNASSFLAQNKGLTGLSGPGLESPAIHKPKRGSYLPFSDGFRACIGRKFVQVEFVAVLTRIFA
GNRVRIGRRKGETQGMADRRAKETMDSSLVGLTLKMAEEVEIELIPR*
>CYP51089A1|578952|Wilmi1
MFESRIIDWSAGTAMAILAVLYVVVKVIYRLHFHPLRDFPGRTAAKITNLYEFYHNGYRGGKYLEEIAKAHQEYGPVIRI
SPNRLHFSDPEVYTFIYTSRPTLVKDPSFYAAFGIPNGMGGTSDPSWHRMLRQTLNPFFSKRAVLELHGTIQTTVDNFLR
RVGETADTKQTIDLQWAFKCLTIDVISDYCFGESFDTLSEPGFDPPYLLALGSALRSIWLSMLFPWIARVAMSLPVSVAG
RLSMEVRGLVETHHRCLGYIKTFKKHMASGTRLGKRSVYEVFYARGKNIPNTVDDRTLGELAFELLGSGTPASNAIMFAA
WFVAKTPGVEERLLKELDDAFPETKEMPYNTLSQLPYLNGIFKEALRLSHGIPGILPRIVPQPGINIAGRFIPGGTSVTS
SAAMIHFNPRIFPNPHAFLPEKWFGEDGKKLDQYNLAFSKGTRQCIGVNLAEAELKMAIASLFRGFLVKLDLDGRNEMKW
EDYFTTTIVGKINAVVERR*
>CYP567W4|578981|Wilmi1
MAIFHIQDIFQLPLGVLASLPIVFLVGRFLAHGIHNLYFHPLSKIPGPKLAAFTQHYFSCIYLSGRYHLIVKDLHEKYGP
IVRLSPTQVSFNTAKSWRDIYGHTSGRKLFPKSEFYDGDLSIPRSIISSRDPEEHAGMRKLLSGAFSIKALTEQEDLVQS
HVDMLVKQIGVYATKEEGEDMVVWYNRATWDIIGDLAFGDPFGSLKDAETHFWVAVVLDMTKAFAYFSMWVKYIGNSWWG
AMLKRMLVPKRLVENRKRHRQYSHDKLAKRLAMETTRKDFLTHIISEKEAHNIKQETLDAHASILVVAGSETTATFLAGV
TYYLCRTPHAYKKLAEEIRSTFSSYNDITGQAAARCKYLGAVIEEGLRMYPPVPIGMGRLSPGETVDGVFIPEGFEVFTS
PWAACRSESNFHRPHDFFPERWLDKDCTDTLEASMPFHLGLRVCLGRSLAYLEMRLILAKMMWTYDMELKDNKLDYARDS
EVYVMFMKPKLPIKFTRREGAEVPLFNDA*
>CYP504A42|582970|Wilmi1
MSAQLLLIALSTAIYLLFHYANRTITPKIKGIPEVPGLPFLGSLLSIGSCHARKALEWSKTYGPVFQVRLGNRRVVFAND
YASVADLWVTQQKALISRPQLYTFHSVVSASQGFTIGTSPWSESLKRRRKAAATSLNRPAIASYMPIIDLESSISIRELL
EDSNYGKKEINPSSYFQRFALNTSLTLNYGFRIKDAEQLKEIVDVERQISNLRSTSNNWQDYLPILRWFGKKNNEAAETR
KRRDRYMDMLLGELKRRIAEGTDKPCITGNVLKDPEAVLNDVEIKSICLSMVSAGLDTVPGNFTMGLGYLSTPHGQEIQQ
RAYDAIMAVYPNNNAWEACLKEEKVSYITAFVKETLRFWTVLPICLPRVNIKEMVHNGAKIPAGTTFLMNAYAAAYDPSH
FAEPHKFSPERYLEGHVTTHFAYGAGSRLCIGHYLANRQLYTLFLRLIVAFRVLPAEKEEDKPVIDALECNRMMTSLTTD
PKDFKLRLVCRDRGMVEEWLKGSEERTREL*
>CYP512CT1|589150|Wilmi1
MDISWIPRWMNVVSWNTAEDSWPWIGVAIAVVLFACYQIYCIQCSRIDIPAYGVPRGPLGPWIAALRFVRNSAELIEGGV
QKYGPSGTSFKISTPGRWVVLATSEAVLNELKDADPRVLSMQAAANERNSISYTLSSTIHSNPYHVDILTKNLTQRLSKV
LPDVVDELRWTFEENVPSGIGEEWAPINTHALMLKCISSTTNRILVGLPLSRDQEYLDCLVELSKMVSRAGLVIDLAPRV
LKSFLAWCMIPRGGAFKIFLDKLGPVFAERRKKIAELGDGWTDRPNDAAQWVVETAPQDASLYELCVRILYLDFSAIHTS

SISITQALYDLATHPEFQTPIRDEIESVLAEHGGWTKPALTQMKKLDSTLRESQRLHPVTTATMMRMTMKPYTLADGTRL
PKGQWVVAPAWAINRSAEQYEDPLRFDAFRFSSAREEPGSEVRHQLASPDKGYLSFGMGKHACPGRFFAAAELKVLLAHV
ICNYEFKLDIEGSHMRPHNSFFSFLCSPDFAARLMFRSRADAGSQKGLSGLSGH*

>CYP6501A1|589347|Wilmi1

MEIFDLFRNIIYLSAVYVFLSFTYRLLRNILICRQIGFPVIVYPLHRMNPISLFSAPFNRWFIERLPFGLSEWKYLHFYY
RDWEYNTKFEQFAEFGDVFMEATSGGRTCYIANAEAAYQVFHRRDEFPKDIEYYKVVRFFGDSVLTTGGAKWRHQRKITS
PSFSEDVYSLVWAETLSQSVSIITQWQSAPNPLVSGPDNIPGHLIAPDTKSLALNLISKAGFGVSLPMVSASSRRTKTSN
TPSNRKGEIDITDDEYFSVDFTPPGHSFSFGEALDTVLSHAIFVAITPLSLLHRGTAFMKKLALAYEDVELYLTELLARE
RLSPSATLLGALAHGGGLTEEETIGNIFIFALAGLETTAGTLQYAVLLLALHPEIQEWLYQDTKEALDGESADPTEWDYN
KVYPKLVGCLCVIHETLRLHPTFQQLPKTTGPSAQILTVSGRTYLLPAETSVFATLTGLHYNPAYWGASPAEFTPRKWDA
RDPNSGWYAADGTSISTEIQPGTQLRQPLKGSWVAFAEGFRSCLGKKFALMEMCAFLAVLFGRYRVTIESGVGETQSQAN
DRARRIMRESTALVTVAMRGEVGVRIVER*

>CYP6529A3|589964|Wilmi1

MGSYVVALVGSMTAYRLWFHRLSHFPGPFAARITNLYHPYAYMRCRGKYFRLQKSWFEKYGDIVRTGPNELIINDPSAIP
ALAKASKGSWYLIGHNMQSVQLVRDIVTHSIRRRVWDKALSPKAVESYLPRIREHTALLVSALAGETNITEFLGYYAFDV
MGAISYGRSFSMLEKAGKDGSDYYMKMTHKSMRILGMLGHTPWTVLLLEKLGAAGKEHIRFMKWCSEIAEERQKRGGEGD
LFQVLMEAEPQNFGVHHVPLHGDSRTAVVAGSDTTASTLIALFTHLAASPEILNKLQRDFDANDTNSEYLEACISEALRL
NPAVPSGVPRTTPTEGIMLDNGTRIPGGINILLPLYATMRDPRWFDSPNEFKPERWIDPNPEELARMNAAFHPFWVGRYQ
CAGKVLAMTQLKIVTTAVVKRYSFCLKEGLTAEKAMDGCLDTFTMEMGPVWCVFTEREKT*

>CYP5268A10|590701|Wilmi1

MALSPVILAVAAVAATYLLHHLLSKRLPSGTTTPPGPSAIPLLGNLLQVPKAHSWLQFKTWADSYGPIFQLNMAGKNHIV
LSTEKTANELLRERGTLYSSREHMYFASDLLSHNLRPLLLPHNDVWRRGRKLMHTLAMPKMAASYEPAQSLESKKFLYDF
FNSPADYQRHLERYSSGLIFRIGFGKTVATGHEAYVRRILGVAHTLERAASPGAYLCDSLPILQWLPEFLAPFKKEGRRC
HEEEISLFRELQDDVREEMENGNAPECFTRHFLERQEEYGLSDDEGAYVIGTMFEAGSGTTASAMMSLLQCMVRYPEWQK
QGQEEVDTVCGDKLPQFDDIPSLPRVRAIVKEVLRYRPVTAGGVPHKLIKDDTYAGYFFPAGTIVHANQWAIHRDTSLYP
DPESFNPNRWLDPEWPTYKPNQSINPNLQNYSAFGFGRRICPGLNIAERSLYILTARVLWACDLRKKVDPETGKEVAVPE
YAYTEGFNVQPLPFDFKLVERKGRRKVCEDVWEKAKEEWGRVG*

>CYP6643A2|592684|Wilmi1

MHPLLLLLLLLLPPTILLFHHRHRRHSTIPTLHPLSTYTTLPILLPTLLGYRNKTIHAAHIRHGPIIRLSPNEISLSSPE
ALSTIYTSFPKPPWYNIFRNYGIPPMFSLLAGKSHAARKKILAGSYSNSVVSRSVVLREVAEEVIPELLSGFEGKEEVEV
WSRFVKLAMDFVTAFLFTREVGSRFLSGEDEGEILRRYHSRQGYFAVSSELPWLSRWVVPGWVDDANTEIEAWCERLCDA
RRGEWKGGEEKCVVDRLLSSGLEKTQISSEMLDHIGAGHETTALALSFTLAAMSTRPEMQREVRRSLEPLLEERNGWRIP
RLREEDGYKALEEHALLNAIIKESLRLYAPIPGSQPRVVPRDMDILGYFVPAGTTVSSQAWTLHRDPAIWGDDVEEFLPE
RWMEGRGVEKAWWAFGSGGRGCIGQYLARWELRVVVASVYANYETTWKGEGVEMVDAYTTAPVEDVLVGFRRV*

>CYP5945F1|593880|Wilmi1

MSLYSRQTFNNRGPFYESGYAASRSYNVLNVRDNEQHRLWRRIWDQAFKASALSEYAPRVELHVEKFISVLEKINGEEVN
CLKPIQNLAYDIMADLGFGRDIGTQDGEDGSLMEFLRSYLRAVTTIGSLRNLCDMMSILPSGLKSAKSIHFQKKSIEMLE
HRKRMGKSRADVFTHLLAEDTETGTTFSDAELASNSRLLIVAGSDTTSTTIACIFRELALNPSIQQKLYEEVHTAAQGFP
VLDSKNTKDLPYLNGVVKETLRLWNPVPSGAETLTGPEGATVVGRYIPPNTTIRVHHYALMTDDRYFPQGDKFIPERWTD
QKMEGVKDIRAWVPFSYGPHACVGKHLALTEIRLTVARTVERFSIELGETYDDELFRKEWKSYVTVVLGDVPVRFKPR*

>CYP51092A2|600600|Wilmi1

MGSFLKETLRIYMVGAGNSIRTVVSPGGFTFSDGLYVPAGVTVCVYSEGVHLDPKNYYNPEAFDAFRFGSPFNAQAGLGD

TSDAPKVHWTMPSSTFLTFGGGNNVCPGRRLADIQLRIALSYFLENYEFSLPPIRPAKTFMWLFQTPPLY

>CYP5095M1|601685| Wilmi1

MSLLSHVQTPLLTFPSHFPIIALSVLLYVLYLLSLAIYRLYFSPLASFPGPKLTALTGWYETYLECFHNGGGKFTFHIRA

WHEKYGPIVRINPWEIHIHDPLYYDTIYNTLPYDKLKENEYRFNAPHASFSTPSHDHHRIRRSALAPFFSKRKVLAQAPF

IQSRADKIVQRLRDEYKGTGRVLCLNDVFACFAGDVVTSYAFEKTYDFLGMPEFKSPFSKSVEGLKDLAHVIKHFPWVVK

MAECLPEKAVVWMKPDVEPVLRFQAELKDQVLSVIRSKSAPTPDEDKHGTIFSQLMESSLPPHELTLERLHDEAISVIGA

GIETTKWALTIACFHILDNPHVLQRLRAELEHAIPHAASIPPLSELEKLPYLKAVVDECLPLAFRSPPPCVRRDLPCLGV

RLSYGTVGRSPRISHDKSFTYGEWKIPPGTPVSMDTWHMHNDESLYPNAQSFRPERWLGEPKGPDGKSFLSRYMTAFGKG

NRICLGMHMALAEITIGIATVFRGAGGELELFETTREEVDCVHDMLAPEPRRGGKGIRVLVN*

>CYP51048A1|602513|Wilmi1

MHSVPYGRLAVFAFGFYYLTLAGYRLLFHPLRKFPGPKLAALSDIWLGYVTFCGRPHYITQDLHEKYGPIVRVAPNQLSF

ATPSSYQDIYARSGSRKIFTKTEFYDFIGGLHTKRGLGTERSPEENIRKKKLVAPLFSAQALLGVESIFQHQLNKFMQRV

EKEGGTAEGVDITEWSTYLAFDIAGQFVLGQDFGAVDSGSRHRWISLILDNAAGAAIIEIARRFYILRFLIKTLLAERAL

KVCRQHIQLTIEKMTSASKEASKSGQPRTVLTYLVEQQVPAGISDEEITANATELILGAAEGVSTSFTIIMYYLLTTPRV

LELLEREIREAFEKFDEIVVTSVTNLPYLTAVIKEGLRICPANPCGWPRYSPGEDIDGFYIPEGTQVSTHPWTLCRSPVY

FANPDEFRPERWDDSDPSNCDNKEASQPFLLGPRQCPGQTMAWIEMRLFFAKILFLYDMKLANPKDELDPWLGKTPTYFL

IGKAPLRVHLTRREESNGV*

>CYP5078A17|602549|Wilmi1

MGALADTILTNLSVTSVITLLKSYPLHLLCSLFVAHLLYNRLRPGLSNIPGPALAAWTGLWRLIDVSKGDAHNTAIKLHQ

KHGNLVRIGPNHVSVGDPAAIPIIYGLKSGFTKTAFYPIQCISWQKKPQMNLFSTRDEQYHRDQKKLVANAYSLTSLLEM

ESAVDSCTKLLIQRLDEYASQGKPVDIGEWMQYYAFDVVGEFSFAKKLGFLEQGGDVDGMMAGIAGILEYAAKIGQVPFM

HKLLLGNPLMPILFPSMESWNQVLNFTLKAINTRCTIQRDGELQVRKDQVLGKDMLSKWASAKIGDPDKMGTRDIIVHLS

TNVFAGSDTTAIALRAILYLLLRNPQKLSKLLSEIDTADSAGLLSHPISYKESQTHLPYFAAVMKEAMRLHPSVGLLLER

HVPAGGITISGQHIPAGTIVGINGWVLQHDPKVYPNPEAFQPERWLESTPEKLAEMERSFLAFGAGSRTCIGKNISLMEM

AKVVPQLLREYTVKLEDENRKWETKNMWFVQQSGVVVSLVRRK*

>CYP6637B2|602817|Wilmi1

MTPLEITTPLSLDFKRFSALFVENKALGYVIALVTLQILYWICLSIYRLTFHPLAKYPGPWIAAVSPFFYSWAFGRGRAG

PVIRAAHEKYGPVIRIAPNDLSFATPNAYRDIYARSPNRKLFVKTLFYEEIGLGFEHIAFSSERNPEVHAKSRKLFTPVF

SVQGVRAYEELLMVSLEKFLVQIERIGSTPRGVDISEWFHRLLYDVTADLAFGESSGATDSADEAYWLKLVNDNINIATY

VEVANRYTVLRFIMKNLVPKRLFEARDRHVSWSIATTSKRIHNPAMTGRPDMLTYLMENDNAKGVSIAEMTSHLSSIILA

GGGTTSIVLGAMIYYLILNPDMLQRVRDETIHLFQTSDEITAPKLSECKFLSAVIKEGLRMMPPAPTGLPRVSPGETVDG

HYVPEGVEVMVHPWTLTRSTKYWKDPWKYNPDRWLDPKSTDVKEAAQPFLLGPRGCIGQNLAWDQMRVIITKIFYLYDLD

LVNAPEDWPSECQTFLTWSTTPLHVSVKRREGAGSDPYFSRTRNLFK*

>CYP504E25|606190|Wilmi1

MSFEFETSNLTGCLILLAVLLLYLHWSFTSTDIGRIKGIPEIPHALPFVGHLLLLGEDHASTCENLWHRYKHSVFQIRLG

NTRAIVVNSFEDCRRMLIGHQSNIIDRPTLYTFHGVISSTQGLTIGSSPWDESCKRKRTAAAVTLGRRAMKNYLEMFDFE

SYCVIRDIEKDSEFGTVEISVKPYFQRYALNTTLTLGYGIRLDSVYDDMLREILDVGSAISLLRSASENYQDYIPILRYF

PNSDKNRRGKELRARRDKYLDILLSTVKDMIQQGIDRPCVSSAVLKDVDSKLSSAEVTSICLSLVSGGFESIPGTLVSCI

GSLSTLEGQKIQEKAYEDIRRYCPNISEAWQTSYQEEKVPYVKAIVKEALRYYTITPMIPPRRTTSELNWNGSIIPAKTM

ILVNAQAANHDTSHFGPTAHKFDPTRWLDATSPIPIERPSVGLQHLSFGGGSRACSGSIIANRLLYIALIRLITSYRIVA

SEKFPPNTDYIEYNSATSAMVAIPKDFKVRMIPRDKEGLKKVLADARCRSEHSYKA*

>CYP6713A1|611603|Wilmi1

MVDLPLSFLLALSSAAAALIFHISTIRVELDYKLWNLGGVYLCVYGLLAYAFSLNTNLTAGALKALLLSVCFNATLTISI
LAHRVFFHRLRHFPGPFGAKVSRFWHIFKLWDSQVGHLLSQEMHKKHGDIVRYGPCELSINLVDAIPAIYGPTSPCTKSL
FYSNVGPEENHSLFHVRNKQFHKERRRAWDKAFNGVNLAIYQPKIERCISVLLQQLRTRGVSPDGIDMTLWASFLTFDVM
GETGFGRSYNMLETGTLHPAVKCQKDSLPMYGIGTKIPWFVRLMMILPPSCSPMKPIMKWCGNEMEEKIKKFNRDEKPTD
IASVLLRDEQCGLGKLKIEATHDDARLVIGAGSETTGMALTGVLFYLATNRSVFTKLRNILDEKFPGGEPEYQYSPSLDI
PYLDAIINETLRLQPSVFSGLPRLTPPEGITINETYIPGNVVVQVPTYTIQRDPRYFSQPLDFIPERWTDESPELCKDKR
AFMPFGLGSHVCAGKAFGIMEIRIAIARICLSFDWELAEGQTESAYFEGQRDFFTCYLPNLFLRFTPRERG*
>CYP6648B1|611861|Wilmi1
MTLLDYLTLNPAAIAGTIFALVTLIPSVWAYLASFRALSSIPTVGLSERRSWTDFWRARQRFVTDSLGLSIEGHKKYPRN
SVYKLWTPDGFKIMVSPDLSGEITGAPDSVLSTHEAFQDSFFGKYTGVDANADLRIKCVRVDLTKSLGRKIPEMIDEADY
AIPLQIGECKEWTGFKLQPILLQIVALISGRLFIGPEKNRDPKWLNTAIQYTTDAFIAAEYLRILPAFLRPLGARIIPEV
RRCSQHLAVAKEIIGPLIEARTSGQKKPGDDMVQWMVDSAITPEEREIAFISQQFLVISFASIHSTTVALTKALYDLASR
QEYIQPLREEVEATLAEEGGVFTRQSVQKMELMDSFLKESQRLSPPQTVAMLRKVMSPSGFTFSSGVHLPQGSTILGIPY
AAESLDPAIYENPTEFDAYRFVKMKKESDDPNRWGFASTNLGHWGNGKHACPGRFFAALEVKLLLAEILLKYDVRMAGGK
PRPKDGLFAVNIMADMEAVVEFRKKR*
>CYP6855B1|611952|Wilmi1
MVLVANTPLLWVSIALVAVTAYIFRPKRVRPYSNAPFLTISLLERLQNLRGITALVQHGYSKYNQGTFQYTTIRGRRQVL
GCSDALIAEIKNASDENLSFAAWAQEFHQMDTVFPDYSGGVDMVRWPSFAKLSYRWFKNTLTKDLAETFPQMKKDLMDGW
DPEYLAAMQDPMYNVSRNCVIGAIGRYLVGEPYCKEPVFLKALDNLTAQLSIASIVASAFPSFLKSSAVRYLTGFEKPRQ
YVRKKLYELERAKDEKQVKDSYDFFSTALASREKNQRDDWPFERLITEICTTSFAAIHTTSMTITTVLLELVARPEYQVS
LRDEIRSMVASKGWTLEAIDAMSQLDSFMLETRRFRPLADIMLNRMCMKDTTLSDGTTITPGTYVSVAYSPRLLDGQYYS
SPTEFDGLRSLKTEERFTDVDGAKSLGFGAGKHACPGRFFATAMVKTSIAMLLERYELLPGSAEMEYEMAFEEQRVPSMK
DKILFKPL*
>CYP6742A1|613955|Wilmi1
MLLHTTIIQCLLQCFLFFFLLSTLILILKSSYRLFFHPLSSIPGPPLAATTNLYQVFQYFYRGRWGEGEHQMRLHKKYGP
VVRYGPNYVIVDLPHALPQIYHRKADKADWYRRGFGPVTAFSAKKHADHVVAKKRLAYGYSMTTMKAFEDEVDGRIQEWV
AALDKRYCETGEPLRFQEGITYLAYDVVTEIAFGEPLGFVNEWRDVRGLIKNFEDSIPTFQAFGRLPLLTKFSNTFGFLR
PKPSDKHGFGLLMAEADRIFEQNQHLHEKKLEKFEKTSLLSRFMRTTAEGGEPMTSDQVKFESITAMVAGSRTVPEVISP
FVLHILKNPNCYSRLIVELSEAENSDLLGEVAGVVTYETAIEKLPYFKACLREGFRISSVPFQMPRISPPEGILLEYEGK
STFIPPGVAVSCSARPIARHKDLYGEDANVFRPERWLEADAETIKSWEKYNLSWGYGTRICLGKNIAMMELGKICVQFFR
MFEPELLEYRDGDRGDEYEDFMVRLRRRMEV*
>CYP6648A1|615969|Wilmi1
MTLVETLTTSPAFSAVVILTLLSLLPRIWSYVTGFYALPSIPCVGLEGGTGFSSFWKAKKRFVLDAKSLLVEGYQKYGRG
GGVFKLWTPNGFGVAVAPGLVSEIKNAPGSVLDFHNSVIDHIAGKYTGLQHLPILAKCIRQDLTRNHGRKLPIMAIEAEY
AFSTHFGQEWTSFQLHPKVLQIVATISSCVFIGTPRNRDPEWLNVAVMFTVDVFNGSRTLRSLPLPKFLYPVTARFIPQI
RRTHLHRAAAQKIISPIVQARLEGTEEPGDDMLQWIMDSSHTAEGKHPHMIARHVLQISLVSIHTTALTLTKVLFDLACR
QEYMQPLREEAEKVLEEEGGVFTLQAVRKLDLFDSFVKESQRLSPAGQVAMLRRVVSPDGFTFSTGAHLPYGSTILGVTT
AAASMDPEIFENPNEFDGYRFQRLRSGEDWNKYTFSSTETLHWGIGKYACPGRFFASSEIKLLLASIVLKYDVRTKDGQR
PKDICWELSMSPDPTASIEFRKRR*
>CYP61A1|620166|Wilmi1
MDSSAYNPPPAASAVEFGGQAVVDGVGAVGKTLEGINMWQIVLTILVLSITYDQVKYIWNKGSIAGPAFKIPFMGPFMES
VDPKFSQYLAKWQSGPLSCVSVFHKFVVIASTRDLARKVFNSPMYVNPCVVDVAKKILRPTNWVFLDGKQHVDYRKGLNG

LFNRKSLAMYLPKQEEIYDQYFKRWLELSKDGKPRQYMTEFRDINCAVSLRTFCGHYITDEAVVDISENYYKITAALELV

NFPIIIPFTKTWYGKKCADYVLEEFSRCARLSKVAMEAGEEPQCTMDFWIKSMIDSREYKKLSQAEKDEYAGPKPIEVRE

FSDIEISMTIFTFLFASQDASSSASTWQFQILADRPDILAKVREEQLRVRDGDPYKRLDVDMVDKMVYTRAVIKEQLRYR

PPVLMVPYEVKKSFNVTPEYRVPKGAMIIPTTYPALHDPEVYVDPESFNPDRWLEGGEAEAATKNWLVFGAGPHVCLGQH

YAIMNFMSMIGKASMFLDWEHHPTPLSEEIKVFATIFPQDDCYLTFKERLPLRAP*

>CYP6002C32|622495|Wilmi1

MTSNPQPNGSGPPQLNGSAPQLNGSAPQVNGSGPQPNGAGKKRKAGSPSILQRLRSLLSAVIRRSSRSRRPAPPPPPPPP

PPPATHAAPPPAAAAGPTPAAAVAPPVTAAVTAPATTAVHTPEKEAATVVQNLQSQGAPIPVNTTQKVGATEALGSGGLI

NDREYTIEKIIQLAVSLPQGSDNQVALTGQLIRSLWNILQHPPMRYLGILGSLPTPQRGALDTDFRYRAADGSYNSLLFP

SIGMAGTPYAKSVAAKVKLPAKLPDPGLLFDTLFVRDKTIEHPTKTSSLLFNLASIIIHDVLKTDPTDSNKSSTSSYLDL

APLYGSNRAEQTLVRTMVDGKLKPDTFSEYRLLGFPPGVSAFLICFNRFHNYAVTQLAEINQDGRFTKPAALGANPTPEA

QDVYNKGMAKYDEDLFQTGKLVTCGLYVNIILNDYVKNILNLNRVQSSWTLDPRDDFGHLYDQTTAIPSGTGNAVSVEFN

LIYRWHACISTRDAKWTEDFYERNFGVPDPSTISPEELQRKLRAWAQGIPRDPSKWEFSGLKRGRDGKFDDRELVDLITK

STEDIAGSFGPKNIPKVMRSVEILGIIQARKWKVATLNEFRQFCQLTPHKTFEDLNPDPTIAQTLKNLYEHPDKVELYPG

ILAEDAKKPMIPGSGLCPGFTISKAILSDAVSLTRGDRFYTIDYTPSNLTNWGYTEVASDPAVIQGRVLYKLFINAFPGW

YRSNSVYAMFPFTVPSETKTILEGFGTAKDFSFDPPSYIPPPTPCLTYRAVKEVLEDNERFHVPWGPHIYELSGRDYMLG

GDQPSNYQEREEFGKSLYDPQNGVEEIGKFYEVTTWKMLKDKSYSTGKGFRVDAVRDIGNLVQSHFVATLFHIPIKTADN

PTGFTEQELYKMQATLFTWVFLDIDTAKSFELRQNARKAIATLTPAVEAAVEAVKAGKPPVNPLPSDAPLSTYGDHVISS

LLEGKTVQEAVGAILPTVPAIVLIAQAFAQLLELFLSPTYNNHWPIIQHLAQDTTNPASSFQQLRKYALEGTRLSPAAFG

VIRQFVSDTVTIQDGPGRSVNLKKDGSVFLDLVSTCLDPVAFPDPLKVDLKRPEVNYIQFGYGKHQCLGLQIAVTALASL

LRVFGKLQGLRIEDGQRMKSKVIRGGTRCYLKEEWDEWYQFPTTLKVRFDSFVE*

>CYP6761E1|629044|Wilmi1

MTVSDDIRCLLEDHTPEWTHPHQQWSNLAALLLLLFLAFWLILIIYRIWFHPLSHIPGPRLAAATYLYHAYYQVYKGGMF

FKKRPELLAKYGPVVRISPTNVEIYDPELYHSVYGQKSQYLKDPQHYRRMAGGRSSAFALDPQEHRLRRKLINPMLSKKA

VNDAADAIIYGVVDKFISVMEKLSEDGNPIPIANGFYCVTVDIISAYLFGEAWNMLDEPGLKGEWLDSLMSIANHTNLGQ

HFPGLVQTMLFLGGLFPSIIPVAIQKHTRNCTQLVQEYLDADKATKASGATTAAAEVAAELKETPPRKTLMDAIVNPPPS

LANYKTSFPELVDESLVLMVAGTDTTAVTVQYATWHFISKPDVKKRVLSELTSITRDSNGHLPLADLEALPYFSGFICET

LRHNVIVPGRLPRIVPAGGITVPKTNTFLPPGTSISFCTTMIHNSSEIWGDDAGEFKPERWVGNPGLDKWLLSFSKGDRN

CAGINLAYAELYLVLANLFTAFEMQPYMTTEQDMQSVDCGLAVPRKRLMVVAKRKVERPGTGKKVG*

>CYP675Q2|632433|Wilmi1

MLLITLFSNPLFYPIAFVVSVVGWVVYQRVFSPYAAIPGPFWASITRFWYLNRINAEDMHRYTKELHKKYGPLVRIAPNE

VSVSDPAAMKAIYAVNAGYTKTDFYPTQAPNLSPHGDSFTQLDEKKHTYRRRMIQNMFNLSSILESEKYIDNCTETFMKI

LSEYAESGETIDMGTWLQWYAIDVIGELFFGRMFGFMNERRDVGGLIGAVDIILPHAIRMGVLYKWMRPFQILLVPFSSS

LRHGISVFNSLAAESKRLVDERWGPDKKSARTDMLAKLIQVAEEKAPEFDITDVYTESYTAIFAGSDTTAIVMRTAIYHL

CRTPHAKEKLQAEIDEAQREGRLSSIISYAEAIKLPYLMAVIKESMRVHPSIALTFPRHVPAGGRTLCGHFFPEGCRVGV

NPYVLHYEPSVFGEDAEEFNPDRWFRPEAENMDRYMFNFGSGSRTCIGKNIALAEIYKFMPQFFREFDVKLENLEKEWKE

HNTWFVKQTGIMAKLAKRDVV*

>CYP6501A3|634366|Wilmi1

MALKIGLPVIFQPYQRLHPLAVLTAGLNRPIISLLPFGLSRWKYLHFLWRDWEFQTGFQQFAEYGEVFIEARSAGSVLYV

ANAEVAWQIFMKRNEFPKNLRLYKMVQFFGENVLTTGGTTWRHHRKITSPSFSESVYSAVWSETVLLTQTLLGQWTAAQN

KGQLVSKSGIPGIMFRPDMKTVARNVISKSGFGVSLPMISSVISEPETAKQSPQEGRGDKIDVTDDAYFSPDFTPKGHTL

PYGEALDHLLENILLLVIVPRAILHRGTAGMKKAAQAYEDVGIYVKELLQRERSSSPSGQQQNLLGALADSSNSEGGLSE

SETVGNIFIFALAGLETTAGALQYAILLLAIHPEIQDWLHKDIKNVLAQKAACPDPTTWEYNMLYPKLVGCACVINETLR

IFPPFQHLPKTTSQTPQPLTFDSKTYQIPAETSISISLTGLGLNPRYWGSSPEKFTPQKWDARDPTSGWYDSDGTPIATD

TQPGTQMRQPVKGAWVPFAEGFRSCLGKKFAMVEMVAFLAVVFAEFRVEIERMEGESREMADRRAWGVARSSTAAITVAM

RGEIGVRLVRR*

>CYP6713B1|635635| Wilmi1

MVLLSSVLAALATHLLFLHREIDFHVLHLLGLYLVVFAALAYNLTPLTALVCAAYYNATLTASILTHRLLLHRLRHFPGP

LGAKVSKIWLMLQTWKKPQMHLLTDKLHKEYGDFVRVGPRELSISHPAAIHAIYGVNLQKSLYYGYSGSGNEASLFLLRD

GQLHARRRQAWDRALNGTSLGSYVPKLRAGVEQLVTELKARAGESVNISEWAKFFAFDMIGSVGLGKSYGCMESGKLHEA

LPALEAGNWFFAVPGLVPWLMKCLFSIPGAGGAMVSFYKWCTHEMELRIQEMKESGGEKCNDVASHLLADPRCGLGKIPY

SATLDDCRLIIIAGSDTTGAALGVALFFLTLHPHIFETLYSYRKSGDNAYLDAVINETLRLFPPVGSTTGLTRVTPKQGI

NIDGTHIPGDIFVSVPPYTLFRDPRYWTRPNEFWPERWLENPELGRGLYVPFSTGAYQCPGKQFAMAQLRMAIGGIVDAF

EKLEFVDPKDAKERFESDMLDYFVKHPPACRVRFIPRKTE*

>CYP50127A1|643271|Wilmi1

MAVTVILVSLFALYLTHHFYRSYQARLLHSSFPACKPGIKIFDPFGLRRIRNVIKHVRQKRFLFYLGSVYKQYNRNTLPL

PLPGMTGYITAEPENIRALMSTEFKNCGVSEGRAAYGYFLGPGFFIQDNEAWSRPRALLRPNFARAQVSDFTLLERMMQN

LFAAIETHSGAFDIEPLFQSLTLDVATEFLLGDSADSLAGGEGALFSAALEKGLAHVNFAVSLGPVWWLWKPKAYRDSRN

FLHAFVDRYVVRAITRAEEGRTKDGYHFLDALTAEMRDPEVLRAHVLNTLFAGRDTTASLLSWLMWNLVRNPEVMARVEQ

EIEAVVGDELPTAKLLEEMKYFKAVVNETLRLYSPVPLTNRESLRPLALPRGGGDDGASPLLIPANVTVMTDFFTMHRRR

DIWGDDSEEFRPERWLETENLDREMTGAFKYLPFGGGPRSCLGQQLALNTAMYTIVRIVQRYAGFEKKEGDSDEVLFTST

PVPAPGAGVWITMDEKRV*

>CYP682V2|648990|Wilmi1

MPLLSASIDVLKPSLIFSFAAFLLASYIIREVVYRLFLSPISHFPGPRLAGLTYWYEFFYDVIAYPEYTFKIRHLHEKYG

PIIRINPDEIHISSPDFYDQVYASTKRKRDKWDWIVKSFGVDESLISTLSHDHHRIRRASLAPFFSKASVRALQPLLEKE

LEVLLGRFREFETKKEPLTLNVAFAAFTNDVVMQYAFGWSNHRLSSPDFDPSFQDALLAGGKAGHVLKHFPILLRLLRSL

PDSLVSQLSPMWGLYAKMQTSIKAQVADIIGAHSTMAFDKTRRTIFHEILDDNKLSDYDKSTERLWQEGEVVIAAGTITT

AWAMNVAAYHVLSDPQILRSLKRELEVAIPDPKGKMDLVALEQLPYLTGVVQEGVRLSHAVTDRLQRICPDETLVFNDGK

KDWHIPPGTPVKRWIENPGLTRYLVAFGKGGRVCLGMNLAYAELYLALAAVFRVYGSKEVQGKDDVGVLELWETTSRDVG

VMESDLETPQMPGGSKGIRIMNYMYDFK*

>CYP512CS3|652360|Wilmi1

MREGYQKYGKGAKAFKVATPSRYMVVFTHPKMVKELADLDDSIMSFEACVRERMSTDYVFSEKFASQAHHNIIIAKNLTN

RLGSILPEAMSELIMAFEENTDIGPDWTSVNNFNVMLNCVARTTNRIFVGLPLCRDQEYLDNVIAFAIQVTKSTAALDMF

PKFLKPFISNRDKALKKVMLSIGPIVEERRSKMQQFGEEWADKPNDAIQWILEAADPGESIRELCIQLLFLNFAAIHTSS

FSITNVIFDLAAHTEYQEPLRQEIESVITEYGGWSKQALTAMKKLDSVLRESQRMNGVVLVTGQRKAMVSHTFSDGTHVP

KGTLVFAPAHSLHNDTDIYKNPQEFDGFRFSRIREQPGQQAKHQMVATSSENIGFGTGKHACPGRFFAANELKMLLGYII

SNYEFKFEDGKKRPENTFYAYSCIPDVSAKIMYRERADRNKSFANSCLPKGY*

>CYP6713F1|655210|Wilmi1

MTAAVAICFNSALTSSILLHRIFFHRLRNIPGPFWAKVSKAWMLKKVWSKPQAFRYIEQLHEEYGNIVRVGPRELSILDV

AAVPLIYGGASPCMRGPYHSQTADGNNGNIFFVRDKRLHNMRRKAWDKAFGRTALATYAPRVHELTSILLRQLRLRTGVP

VDGTQIARWFAFDVIGEVGMGKSYEQLEKMEVHPAVDVLVKGGWYFGVPGHMPWALRILSSIPGGGGALSAFQDWCQSQM

EQKLKRDTASTSTTEKPQDVATYLLQDETCGYGKLPKEASCDDIRLVILGGSDTTASSIGGLMYYLTANPRVYRKLQSTL

DAIFPGGDAEYDYFKATNIPYLEAVINEILRLQPPIPCGPVRTTPREGLIVAGEYIPGDVCVSIPIHGIQRDPRYWEKPL

DFIPERWTEEQPELCKDKGAFIPYTTGIYQCAGKPLASLEMRMIVSRIALTFDLEFAEGKVPENYERDIQDLFTLRLPSF

FLKFTPRKGEREV*

>CYP5945G1|656212|Wilmi1

MENANLWLTRGRLRRDQALKKTSLDEYNPRVEHHVAKFIKLLEKTEGKPVDAALLLRNMTFDIMADLGFGLDDYGMQEGT

GDSSYSDLIYRYMWAVAVVGALRNLCQLLSLLPIDAGVREFQRKRQIIIDNRIKLGTSRRDVFSHLYEGDKQTGTKFTPT

ELAANAELMIIAGSDSTSSVLSFLFRELALHPEIQEKLYKEISLVSESELNVENTRPLPYLQAVVNESLRFWNPVPSGIQ

HQTGPEGVTVAGQYIPKNTVTRTHHMSLMRDDRYFPLGDKFIPERWIEDCAESVKDKRAFIPFGFGPHACVGKQLSLNEL

RLVTASVVKKFALEFGPGYDDKKFLSEWKEYLLVQIGSIEMKFVRRQ*

>CYP671K2|657353|Wilmi1

MVSDVVSLSIGAAALAVGFHISIIPHEIDFQLKRLFVLYTSVVGCLFYLLQRTNTSNAALKTVFAAGIFNVSLALSIFVH

RVFLHRTRKFPGPFWAKVSRFYAVYLTLKKIQHHKEVEKLHKKYGDYVRTGPREISIIKPAAVQAIYGSQSLCTKAGFYS

QATDHPNGSLNSTRVKSYHNQRRKAWDKGFSIAALKEYEPSVKQLTDLLISQLRARAGTVVDAVEWSNFYAFDVMGAVGF

GKPWGMLESAELHEAISQLHSAMVPLGVLGPVPWLLRLATDLPGANKAVQSFMDWCWNQLSEKKKNLDYEGKPKDVMTWI

LTDSMKLPDTAVNEDSRLMIIAGSDTTAAALANALYFLLVNPHVYKKLQNILDDIFPGGDSDFDYSKASSIPFLDGIIHE

TLRLKPSVPAGLTRITPAEGSPPPPSPQKGTNKPPGLTIEDVYIPGDVVVNVPVHTIQRDERYYEKALDFIPERWTEEKA

EMIKDKRAYAPFSLGTYGCVGKGLAMMELRMAIARIALGFDMEFAEGETGRKLDEETKDTFTLTIPGMEVVFRERRRG*

>CYP53A60|660372|Wilmi1

MAFIIYVFLGLLPIAYYLIPYLTDSKLRRFPAPFPGAYSDFWLFWQARKGKRYSAVHNLHKKHGKFVRVQPKHVSIADPA

AIPIVYGHGTGFLKSDYYDAFVSIMRGIFNTRDRAEHTRKRKTISSVFSTKNVLQFEPYIHHNLELLASQFNKLSAQPDL

PGGFHKLDILHWTNYLAFDIISDLTFGSPFGMLESGKDQAIVKDLNTGKITTATAIQVLNRRGEVSGTLGCAPWAKKYAK

WFPDKFFTQGIKAVEIVAGIAVARVSDRLDRGQGADREDLLKKLMNGRDENGNPLCRKEIEAEALTMLIAGSDTTSNTLC

SLMYWVLRTPGVLGKLQEELESALPGDWTVPNYASVKDLKYLRAVINETLRIHSTSSLGLPRVVPPQGATVCGEFFVGGT

VLSVPSYTIHHSDDIWGDAETFRPERWFELTDLQKKSFIPFSTGPRACVGQNVAELEMVTIVATLFSGWDWKFAEGEKQG

LPGAPLNTVEGFLRKPLGLNVGVKRRNV*

>CYP6761D1|660808|Wilmi1

MGILLRLESALEQIGLSYSLPTFRQFLIYAPLLWLIFCALVVIRRIWFHPLSHVPGPKLAAATYLYHAYYQVWRGGPAIR

ISPRDVEIWEPELYHTVYKQKTSYLKDPVYYHAQGLTLSVGTILDPSEHRTRRALLNPMLSKRKILEVSDVILQGQIEKF

VHILEGMAERNVPIPLAHGFYAITTDIMSVYLFGKCWNIMDEPGFRSELLDSVLSMIDYFNLHVHFKAFAQALAKLAVWF

PRLIPVAARRIRKNCEHLILEYLANPKKLAGNSHTTLMESMLNPPEGFPKQTFPFVDVVEEAVIMVMGGTDSTASTLQFA

TWRFLTEPGVKEKVLAELDSVERDEHDCFQLHKLEALPYFSGFIKEVLRVYIIAPTRTPRIVPEGGLTIPSTGLHLPAGS

CVTQYIGLLHHDSRIFEEPEMFKPERWIGNPGLDKWLLAFSKGDRICIGMNLAYAETNFVLANLFTRFDLQLWNTTEEDM

QWRDCGVAKPVGRIQVMAKKRRSLSSSTPVRTFNYILPPRRFTQSSTYMKLPPEDQFNTHKLDHIKIRESEPVFWIAPRL

STPHQLHFAASK*

>CYP6497A1|661595|Wilmi1

MSPTISLLIAVVSYHLYLILLYLHRRSQAKATGLPYFSFPIGDTKLWYIIILGLPPVIWVIENLLPPRLQDYVNTSCYLRR

WNAKHRVHKELGDVVLMVSSGQLSCYIADAAATREVFAGRGKYIKPSWNLTHLKLFGNNVVACDDAEWIHHRKHTKPPFN

EHNSALVWQQALVQTTDMLTEWESKPQSSPEYTSSGRIITSSRQDFRRLALHVISSAAFGISLSFSTPTAAKKDATIGND

HDVFSDGAPSKGYSRTWRDALEYISMNFITVLATLSVLPRWAAPGTVRVVQDVEKYLNTLVSYERANDNQRNRKGNLLSA

IVRKDGNSEEKGDFLTDREIVGNLFIFSVAGHETTASTLQYALVMLALHPEMQEWFLRRLDEQLEGVPLDATQWDYEKVY

ERLSAPRCLMYEILRLFPPIPGIPKWISTEQSLTVGNSTHLLPGGTFVTINAGGLHHNPKYWGPNADAFDPSRWDLENRD

SFLKSFEQPKDGWQEAPGLYRPLQGSFASFSGGQRVCLGRKFASVEFVGVMAALMRGRKVSLARMAGETEDMARDRAWGK

VRKSVALSALVMTDDVGFVLEGR*

>CYP50335B1|665034|Wilmi1

MYLFILVLIPSLLYILLNHRRKQSKTTITSLPQIRSLLSHNLTTHDRITALTTSLTARAIPNYRLILAFGINNSFTTISP
PHHKRFKTRVEKLLRLDPASWKIISFVARSLVPSQPNSGSLHLAPFLRKYVLTIIIHLFFPAYQNIGVETSNSIAELGDL
INDLWIQSKHGEPSRDDQRRLSETINVLFPGIDENPLNLLLPAYETLWRVVLRCFLEVQFRGGRKVWRDVLRRYLDNPGS
EEFVAVLEGEGDGDGDGVVSVKAIVAETLRLYPPTKRVYRAVTPTEVVAADIEAVHCDPELWGEDAGCFKPERWSEGVDE
VRCGFMPFGEGAFVCPAKKVFAPRLIGILVAAVLEGVPVDAGWVAEKKEDEIVMEGRLGNERNEFGTLVLKWGEEN*
>CYP50241A2|667237|Wilmi1
MNQNFRPEYIFQMKRKIAEYNRWTIPSIRHQIVPTMKYRIPALVNETLYALDRYFPSEEGKWTSFHAHDTITKTIAPIWS
RIFLGQYRCRNEKYLNAAVKYGKRSGVWSMVLNCCPTWLRPYASLASFFVICANGLSGWFTVLAVYRRHRIYWAKLRKIL
WLKSWRGSGKRSDIIMFYSLALNQAGILTNTLVSMNILYDLAAHPEYIAPLREELEAVLEKNGGWNMETIKGLVKMDSFI
KESSRLNTLAFSSMPRKVLFGYTFSSGLHVPKDSFISIPSYTTHLDPDLYGTNADTFEGFRFLKNGMDGPANKGSFQDPT
EYYHTFGWGPAACPGRAIVSPLLKISIGHILLRYDIRPRERPSPLHLGNFNVPCVRAKLEMRRRAFKVQDGLSL*
>CYP566D3|674974|Wilmi1
VFFVLCLAIYNCFFHPIVHLPGPLLARCSPLWTMFALHRKRFNSELQDLHTKYGPVVRIGPNEVSFATIEAQTTIYGTRP
GQAEDHFSKDGTFLTLFSDLVLNAPTLITIADPGLHKHLRKILQQAFTPQALAEIESIQHVHLEKLMPTFDRLAKEGEVF
DIALVLEEFFWDIIGDWSFGEPLLSGRKPMYESLKGLGKRTMPFVELLSYVTIMPGVNNVVRHALAMIPFQSQLQSKLLS
KARLRDCMDRQDGRKDFLTAIMSSKDQGLTLNSEATLSNAVGLTLAGYQTTATTLSSIFYQLLRCPKNLHKLQTELRTTF
SSASSIRHDRLLQLPFLNACIKETLRLLPPANGKTAQRAAPSGVIGDTYIPAGTLVSSDIYSIQRSPLYWADPEEFRPER
WIENGPGEKYEMDVRQAYRPFLIGTRSCIGRKMAMQSLRCVTARLVWGFDVEMARGEGEGWVWERDAGSSLIYTDYRVLV
SMRPRSVDES*
>CYP6001C28|581734|Wilmi1
MASNGSKATTSAAAEASSSRTSSNAPLTNRPTSKTVARINPPVGKPTRKDIDATFTKFAALIHASNRPLPNRYGDGRDDP
DDEKLTGIRHDIMVLRRGGFMMESLHTLWNTLQNKRKGGPTDDKTMIMERLIQLTSRLPPTSKLRHKLTTIQVNTLWDSL
QHPPLSYCGSKFEYRQADGGYNNIQDPSLGRAGSPYARSVKPMTKMPGAPPDAYTVFDAIYSRGKSGENFRSSNNNISSM
LFYIASIIIHDLFRTNRADSNISDTSSYLDLSPLYGINEEQQKTVRTFKDGRLKNDCFAEKRLLAFPPGVSVLLLMFGRF
HNYVATQLKVINEGGRFDLKHDRRWYGDDEATKNAKALKQQDEDLFQTARLITCGLYINFILNDYLRTIVNLNRVDTTWT
LDPRWEPSKMYNPDGTPAGVGNMVSVEFNLVYRWHSCISKRDDEWTQEFYKSLFPGRDTETLTMKEFIMGVHKWEQNIPA
DPAERTIEGFVRQADGHFSDDDLVKLLTESIEDTAGAFGARNVPHVLRLVEVLGIEQTRRWKVASLNEFREFFGLQRHKT
FEDINPDPAVANALRQLYDHPDYVELYPGIVAEDDKEPMVPGVGIGPTYAVSRAILSDAVTLVRSDRFYTVDYTAGALTN
WGIEEASSNPNVLQGCVGYKLILKAFPNHFKYNSIYALYPLTIPSENKKIYTALGLGDQFDYERPKFIRPRIPITSYAAT
KKILCDADNFKVTWGVGFDYIMEAQFMLSGDGAPYSNMKKFVGDRFYGQGDSPRIDWKAQIRDFYRDLTTKLIRKKAYQI
TGANCYQVDAVRDIGNIAQTIFAATIFNLPLKSEDNPKGIYTEQEMYMVLCAMFIAIFFDIDSSKSFPLRHAAYTATRQL
GALVEAQVKAVKSWGWLQGVWDPLNIRGRNKSPLVDYGYHMVNRLLESGESPADVTWKYIVPTAGASAPNQGQIFAQVLD
FYLQPENARHLAEIQRLATENTDEAWETIRKYALEGGRLAGTFGLYRRLDADGMTIDDGGRSVQLQRDDFVFVSFISASR
DPEVFPEPLEVKLDRPDESYLQYGDGPHECLGKHANIVGLTTMLMEFGKLPGLGRAPGLPGIMKTIPKPGGFKVYMKEDW
SSFWPFPTSMKVRFDDII*
>CYP52AV7|586221|Wilmi1
MGVNLTLKEACALVLSGLLLYRLLSTLLLWRNNRAFARKHGCKPLRRFPSGIYGLSHFWQVIAAAERKEHVQFLADRYKP
GWYTFMLNIFGSDIVHTVEPENIKTVLATSFKDFSLGPVRQEAFHAMLGDGIFTLDGKGWEYSRSLLRPQFSREQVADTE
VLDVHVSGVLDLMKKAEGKEVDLQPWFFSLTLDSATEFLFGESADSLLKEETGQKDFAYLFNEGQQWILWKLRWKKLSQI
WTPQAMIDVNNGVHKFVDHYVHMALNREKYPLPTTSSKKYIFLDAVAQTNKDPKALRDQMLNILLAGRDTTAGLIGWTFY
LLARHPHIYKKLRGELESAFGTGEPGVWRRPTFEGLKDVVYLRYVLNEVLRLYPSVPLNGRDAVRDTILPVGGGDDGLSP
IFVPKGGRVQYSVYAMHRRTDIYGPDALEFRPERWGEGTKSGRGWEYLPFNGGPRICLGQQYALTEAGFTVARILQCYER

MEAVYPDEVPKIEATLTISPQQCLVRLFPVTK*

>CYP6006B2|599481|Wilmi1

MWRPSLILLLFISVVFAGKWPYNRYEGFPKNSGLWKPNKTLPEFPHHQRPRNLFARWLRHQVENDSTGQLKKVFTNGSIP

RGFGSFDNLLDHIDAPTPPSETSSPFLPPTVPFSPQIEAAKLFEHLPYPIDPRCTVDRSKFWYRTYDGSCNWMKVGEISE

GQVGTKKSRDYNQYAYADGVSKPRDGPNPRAVSNAFFKRKKTLYYEHTPLLLGLIEFIMHDISYSQDSTTEFIDVPVPDD

EDVYSKNTTFRVWRTEAVPGTGTSPENPRENINMASTWLDVSALYGSTPDVAAKLRSFTGGKLLTQELQTRGRKTKASYL

PFNTMDVPTRTRPGLDPKELFAGGDPRTNEDWVMLGVHTLLLREHNRLCEILAKQHPEYDDERLYQTIRLVMAGKFALIA

NSYQMAYWTEEMPWPRDDGFPLYREMYGEDILQINPANSYPWPLVTKGGKPMVVSTEMAIVYRFHEFIIPSFPIKDALNE

TIWEQDLFGTGFNATGFIDAGLENVLRGMTATHIPNFKSGVDEAFRSAGQYRGQPFDIVTWSIVHEREQGLPTFNTYFRA

YNKQDPAVEVPIRERFEDFSSDPEAVANLKRLYKTPNDVDLVVGVQLDEEMFPGTTVPKTALIISLFSLFGMGNSDRFSV

GFAIMRCLLVDKPWDCHPSNALEELLWAPKPRDGFPNFRFFDQFWLTELDFQAHGTNLLWRLITENSDIKCLQKSPLFPF

DPVTNPILCSLPSQKVDVGVIIVTALEVLLALAKQNKWKILTALAALALTVISYRQNKKPKQPPVLYGWPIIGKALDFQK

DPKKVLLEGFEKYGNSPSKSFGIKLANLTHFVLSQRADLEAMMKDNPYEVIFNLHEFLRVINFSIITRKENFDSDLHTKL

IRNNFGNPETVKEFAKTVEAAAKEFLQKNPLAPENGESQHHNGLNDFFDRYITYVVSRCIVGPDSFDNEELMQTFMKFND

HATQAMGLASLLPSFLQFIAARNINKDFKTIRKILIPVIQRRRLASPTKNNLIFFLDFILDAVPDDTRAADLVAVIVFGG

LTNLQSTFSSTILDILNTPSLQSTLSPSLSQASSSNINVFSPPPQSSPWSPLRAAMFESIRLCGPITGPARIIASPTTLS

SDPKLHLPKGQAATLSSFYTHRDPGMWGANAACYEYDRFVGGDPPIGMPEYVPWGLKGPHTCPGRWFAMTTIQVMTKEVL

EAYDFVQDFVVGEEEKFIYSAGNVKRMEVGVEVKRRV*

>CYP52AU6|604433|Wilmi1

MRREPFYTFGLRGQLGTISAAKAQRLPARMVELCDELGDTWGYTLFGTRTIITRDHRNIQAMLATQFDDFGLATDRDSMH

QLLGTHGIFTQNGSSWAASRALLRPSFDRAQVADLDRLEIFFERLRQRIEDDATGCIELQNLLQKLTMDSSSDFLLGSPV

GALASEESGSGVNVQNFTEAFDIAQTVIATRWVLSNLYWLYNPKYFQQACSVVHSQVQKYVNRALKLRSSSPTTTTTSTT

STTPKKRYIFTEVLAETTQDPRVIQDQVLSVMLAGRDTTASLLSWTVLCLSRNPSVFQKLRAAISETVGVDSSARIPTQA

ELRSITYLRWVLHEVLRLYPPLHANTRCPIKPTTLPFGGGPDGTAPIALRKGEKVVASFFALHRRKDLYGSDADEFRPER

WGEEKLRKIGWGWIPFNGGPRICLGQQMALTHASYFLTRLLQVYSVLEEDPGVKGLEVRYDTKITMYSGRGVRVRLG*

>CYP539A39|617571| Wilmi1

MLLDSLPQSWAGLLATLTVAVVLMVVVAKVARNRKIYKLGKRSAVIESYFFGITETKEAFKYARMNRNRDYWTMRHEKYQ

NYTMEMEVLWDRLILTSEPENIKAILATQFHDYGKGEVFHEQWKPFLGDSIFTTDGKEWQGSRQLIRPQFVKDRVSDLHI

FERHTQHMLRLIPRDGSTIDIADLFFRLTLDSATDFLLGESVNSLGTTGESEFAKAFGQIQEHVNDITRAGPLAVFIPDR

EFKKNLAVLNSFVEPFVEKTLRMRPEELKGRNETDYNFLHALAGFTRDPKVLRDQLVAVLLAGRDTTAGTLSWALFELAK

RPDCVQRLRNEILDTVGPDAAPTYADLKGMKYLQHVMDETLRLYPAVPFNIRVALKDTFLPVGGGESKLEPVGVPAGTAC

AYSAIVMQRRTDIFGPDANEFKPERWDNWAPKPWTFIPFNGGPRICLGQQFAYTEMAYTLCRLFQNFDALLDRATQPQFE

RCETTISPGAGVKVALRPVEK*

>CYP6001E2|661035|Wilmi1

MHPLTEFFNQNSSVSQYGDDSLATKDAIKTGILADLKGIGGDLPKDAMTIIEALEAALKGEPIDDKKLMMEKMIQLVAAM

PRSSKNRTIITGKLIDQLWGSLQHPPLQYVGDKFQYRQADGSYNNIMSPDLGKAGMPYARSVRGEKMTAGAKPDPGLLFD

LLMARGDSVKENPAGLSSMLFYHASIIIHDVFRTNRKDPSISDTSSYLDLSPLYGKNLDEVNEIRTFVGGRIKPDTFAEK

RLLGLPPGICVMLVMYSRFHNHVVEKLAAINEGGRFSLNLRLPKEAAEKKRDEDLFQTGRLVTCGLYISISLHDYIRGIG

NLNHSTSDWTLDPRVEIGPSVFDKDGTPRGIGNQVSCEFNLLYRFHSGVSKRDEKWTEDFYRELFPVSDPHKLTMPQLME

GLAIFERHVAEKPPHERTFGGMKRQADGSFDDADLVKILKESIEDPAGAFGANNVPDILKQIEVLGIQQARNWQVATLNE

FRKFFGLQSHKTFADINPDPYVAETLQKLYDHPDLVELYPGLFLEDTKPRMDPGMGLCGPYTVTRAVFSDAVTLVRSDRF

YTLDYTPQNLTAWGITEVAPDYECMGGAKMSMLILNAFPKWFKYNSVYALQPFFTPKESKSIFTKFGTADKYSFEPPSLE

KDPIPILSHAGVKTVLKDTTNFNVPWGPAINFLMEGTFCLAFDGPQSVKQHQDIAQALFALPDYQTKFKVYTEEIICKLL

KRESYTLGKNHQYYQVDIVRDIVNLTALHFACTLFCIPLKSEENPHGTMTESELWDAFSKANIYIFFDHEPTKSWARRRE

GKAGIEALGKKMVPVIEELARTPSFGIIKGLKEGAEKILHPTKSSMADYGKTFAKALLKQGKSAKEVSWILISMATAAIA

NSAQGFAQAIDFYLNKENAEHLKAIQTLASKTDSQGEDKLMRYALEGHRLSNTLGLFRDVVQKVTVTDGGREYHLDKGDQ

VFCSFISSSRDPTAFPDPNNVKLDRPLASYIQWGDGPHTCIGKQINMIQSMCLLKVIGKLQNFRRTPGDEGKLKSIPKPG

GVKLYMNPDWSFYGPYPYAMRVQFDGPLPGPA*

>CYP505A67|588317|Wilmi1

MEELTSSLAALETSDTFPEDDSKPTLLPIPGPPGLPIIGNIRDFDPVFPLGTFLNFAEIYGPIFEMTLGIAGRRVFINSV

ELFEEVCDESRFRKVVIGALETLRSGVGTGLFTAHHGEKDWLIAHRILMPAFGPLKIRDMFDDMHDIASQLVLKWARHGP

EHRILATDDFTRLTLDTLSLCTMGYRFNSFYTKEMHPFVDAMIGYLLESGKRAFRPSIANKLMRRTNAQYDQDIKYMRDL

ARGLVQRRRNNPTDKNDLLNAMINGRDPKTGEGLNDELICDNMITFLIAGHETTSGLLSFTFYNLIKNSTAYRAAQKEVD

EVCGKGPVTIDHIPKLKYINAVLRETLRLNPTAPTFSLTPHPDLDEHPPTLGKGNYSLEGVPAVVCVLQKIHRDPKVYGA

DANEFKPERMLDEAFEKLPKGAWKPFGNGMRACIGRPFAWQEAVLVTAMLLQHFNFQFDDPGYELHVKQTLTIKPKDFYM

RATLREGMNAVALSAATDATHGDGAAQKHPAKKTSGSDKKQQKKRMSIFYGSNTGTCEALAQSLATNAAGHGFDADVRTL

DSATEKLPKDYPVVIITASYEGQPPDNATHFVEWLKSLSGNEASGTNYAVFGCGHRDWQATFQRIPTLVDDTLHKLGGTR

IVERGLADAAEGDMFTNFDTWEDELFWPAINSRYGSTSAAAASTTLVSTLTAEISSNRSSKLRVDVSDARVIAAKTLTAL

GQPEKRHLEIQLPSNAAYHCGDYLAVLPINPKQTIHRAMKRFQLPWDARITLTSTAPTVLPTNCSMSAHDIFAAYVELSQ

PASKKNVLSLAAAAVDPETKSQLEALAGDQFQAEITQKRISPLDLLERYPCLHLPLGSFLTMLPPMRVRQYSISSSPLHS

ADTCTVSYDRLEVAALSGSGGKHYGVASNFLSSLERGDILHVAVRASAKPFHLPLDPATPIIMIAVGTGIAPFRGFVQER

AVQIAAGRTLGNALLFVGCRYHDKDRLYAEEFDKWQEQGAVQLRYAFSREPELSKGCKYVQDRLRKEKVELCKAWEDGAR

VFVCGNARLGEEVAKVCKDGYRERRKDQGEETTEDAAEEWFGKLKEERFATDVFD*

>CYP51F1|650501|Wilmi1

MSPPGRPETQRTPASSPGPQETQSSTQVASQFAHPFQHEEEEYDPNAWGYLDPITDPSKKSIKLDKTPQCATDSKRRKSK

SAKKTTASGFLIGRHPECDLHIDAAVISNRHCVIYKAVLEDLSSNGTWVNGVIVGRNKRRELESGDEVEFAGGHTYHFRY

PAGMFAHGFRDTFELGAPLGSGHFATVYRAVEKKTGAEYAVKLFAKKRAEEPSRSQGLQQEIAMLMSVSHANVLCLKGTY

VEDDGVYLVLELAPEGELFNYIIEHQKLSEEETRKIFKQLLNGLKYLHDRNIAHRDIKPENILLVDKQLSVKLGDFGLAK

IIGEDSFTTSLYVAPEILESSAIRKYTRAVDIWSLGVVLYICLCGFPPFSDELYSAENPYTLSQQIRMGRYEFPSPYWDT

VDDSALALIERMLEVDPEKRITVDQALQHPWITGGAFNPADSCGSLADAIEGLGFVRRKVQHERTLLAQAPGLANPTLEN

PSKAKVRIHQGENGEATAAAEAVTPKASNNLDAQTKAFVNMGGKAGDETLYGDSYYEVQDGEGEPSPFNLGLRSTINETS

SSPSPLTPIAVGSTSLIPRRPIHHAMGVLSTLFDSLGSLTGDLAKLSTPLLLAVGTISFIIFSVVFNVVSQLACKDKNKP

PLVFHYFPFIGSAVIYGMDPYAFFSANQKKYGDVFTFVLFGKKMSVCLGPQGNDFVFNGKLAEVSAEEVYTHLTTPVFGE

GVVYDCPNHRLMEQKKFMKFGLTTETFKSYTPLIVEQVEDYIKKSKYFKGAQGQVPLGEIIPEVTIFTASRSLQGKEVRD

ALDGSFAALYHDLDLGFNPMNLMFPWFPFPGNRRRDAAQKKMARFYMDLIAKRRKDPNAAEQEKDMIWNLMDRSYKDGTE

IPDREVAHMMIALLMAGQHTSMATTTWLLLHLAEQPKIVAALYEEQQRICGQELRPLVYEDLAKMPLLNNVIREVLRMHP

PIHSIIRKVKSPMYVRDSNYVIPAGYHVLAAPGASAMDEKYFKNPTAFDPSRWDEMEDEEEAEKFDFGYGLVNKGTASPY

LPFGAGRHRCIGEQFANVQLGSIIATFIREFELALPGDGKVPPPDYTSMITLPTPPAAVLWKRRNP*

Ascobolus immersus RN42 v1.0

>CYP51085B1|85736|Ascim1

MSLSSLTPYLLTLLLLYPIIRLYRNWVHRSNLFFDGCAEPYTFPTGPFGLPYITTLIRAFQASTHLPLLTSHFHLAGRKT

FTFSALGMTNINTMDAENFRKVLSTGFTHFGFGSMRKANFASSFGTHGFFTIDGEEWVRARALVRPQFVFRREQMLEMMG
GNFEGAFQRWLGKVRLDGEVVDLQPLFERLTLETSLKFLWGVEMDGKGEGGLNMSEEELGEFEGAFNGVQIGLAYRHLLQ
ALYFLGGGREFNKQIRVLHSFVDRLVDSCIALLPDANTDSALSTDDSDKPKKYYFIHHLLSAGTPKEHLRDHTLNVLIAA
RDSTALTLSFLLSHLAYEPVIFHALRSQILAIYGPTLSTLLAKLAKNGPDSIETTPPLLANCLSECLRLHPPIPLNQRYA
FRDTSLPRGGGPDGTEPIFVAKGQRVDLNFYALHRDKDVWGADADEFRPERWEEMENGTRRKVTAWEYQPFSSGKRICLG
RGFAKVWHVIGKRV*

>CYP51083B1|28379|Ascim1

MDNPKDATVQYIALVQELGTKLQGLAPVFNTFKAEIRNLADGPLLLVSLKGIFALLIARFIILSIYRLYFHPLRHFPGPF
LAKITNLYSLYYDWWLEGQLTHHVRTVLHPKYGPIVRTRPHMLRFSEPEVFYQVHSVGYTFIRDPHTNTVWNMPRSSFST
DPKIHRARKTKIAPVFKKASIAPIEPLVHEKTQLLIDKIALRIDKNGEDRKGGKVLWLNVQNTTLAYVLDIVQEFLSEGV
GAYRLLEDDNSPDFHAPISDLLIFSLKLSHWHRHFPNAAVFMQNNTPNWLLEYMAPFLKNRKDIRTRIAEGVYGYLSLLK
DEDLEIKPKDSNVNGNGTEKGKKLVGYRELVPHFKDIPEDLVTEAQLLIAAGANTTAYVLTFLLYHLAVDQDMQEKVLEE
VKRLEDGSGKTPSWRTMEGSELLTAVIKEALRLSDPIPEALARQTTKPVTISGAHIPAGTAVEIAASQVLQNPAVFGAHP
EKFDHTRFLASSGATEEAIKQRLKFTNTVFAGGAYVCTGMHMAYMELYIGLAELLKAYKWELSEELKRDGAWLWQDAFVA
WKIGAEPVLGVTRR*

>CYP51069B1|90662|Ascim1

MSLQIEAPSPIALILVYLPLTLLTLLLARIIHLHYFHPLSHLPGPFYASISTLFRVHHMSHGTINLHEQALHAKYGPIVR
LGPNLVSSSSPAHIPLIHHKDVDKPAFLDMPGFGMEHGIVALRDHREHRWKKRRLTNPYSLTTILTSEHLLDKNITTFIT
ALDSRFRANSTPFDFARWIEFFVFDTVSEIAFGKPQGFITSGTDKGGLVQATKDTVKAGFLVVALANKIHALYKSLPQKL
ADWIFVPNMDSDKGIGVMLKLARSLVAETKAKVQAGKNDGERKHFVERLLALKEPDGSPVPDAYIVSELCAVMIGGGDTT
AASIRTFFDHLHDPANAHVLARLLAEIDAAYDQQATPSPVLPYSELAKLPYLHSALRESQRLFAPVGNLTPRLVSPPGLT
LNGIFIPPGVEIGQNPWVLGRDESYYGADASIFRPERWIECSEEERRRLERGEATWGFGPRGCIGRQLALVEMWKVSAAL
LRLLRFVPREGPTGRNCRQFGFWIAEGFWYGVERRDVAEWAWFEEKQDGCC*

>CYP51090A1|93662|Ascim1

MPNLISKDALYGLEHFIQLRALFNHISTQTTAFGVALSFAIVFYFVYYILLHPLAHIPGPFFSKFSDIPYLLRFLSGYEY
KHDYALFQRYGPVVRVGPNRVAVGEPEHVATVYNKNAWKGDNYNLSQNYGIFGIKSAKDHAERKKMLTHPFLPVAVKSYD
PIIQRNVDYFLDALQQTYATRKGEPAKIFDFLQWPAFFSYDTLTELAFGEPMGFVQEQRDIGDMYQSFETALYPIGAMIR
VPSVFKFLKFTGIHDMMAPKPTDGNGVGVILSFADTMIQKRIDNPSDKPDILNKYLSSKYLLENRDILFKELLLLIVAGT
DTVAITIRAILSNVLNDSRVNSHLLSLTPGSPGYRTYVSACIKETLRLSPPGQMPFARVTPPHGHHFGDVLVPGGFQVYP
SFYTINRCTRIWGPDANVFRPERWLGHLNGSEVWSHEKLRIMDLSFGVGPRMCLGKPVAETELQLVIEGLWERFEFDGSH
LDKIENVKVFNQMRFGSMPLGVRLRERI*

>CYP5093H1|97751|Ascim1

MSQLHLTSHSIPQASPLNQDGDFTATNLLRPTDEVTNASNGVLFTYPYHAFASALLVAFTLFMSLRSRRKSKMPPGPKPK
PFVGNKWDLPARKPWETFKAWTDSYGSLVTVWTGRQPTIVLGDAKAANDLLEKRSAIYSSRPRFVIMGELFTNNDSLLTM
PYGKRWIHTRKLFHWGLNKVACQSYKPIQEAESQRLVRDLVATPQLFAKHLERYAASVMVCVSYGRRVDMLDDPVVKKVY
DRMGYMATLNVPGAYWAETFPILKHVPDFLAPWKRDVKRRGEAGTQLLLDLALDVKRRMESPEGAPASFAKMLWERYGTN
NGPLTEREVAYATGSLFGAGSDTTASTLQTFILAMTCFPNVAEKAREELDRVVGRHRSPTWDDMPNLPYCAAVIKETLRW
RPVSVLGGTPHATTQDDVYEGHFIPKGSTVLGNLWAIHHNPKYFKDSHSFIPERYLDENEIREDGTQPYPHRDGHSAFGW
GRRICPGKLLAENSLYITITRMLWSLKISKATDACGNVIEPDIFNYTDGFNSKPNPFSCKIEPRDEQFAEVLRREAVGAE
QFLEQYKPE*

>CYP52486A1|144879|Ascim1

MQVPFLSTHIYHAEAVRATMNNSTPEMLAALEDELILAFEDEVSELEKAADNDGWFRYPADAGLRIVSRSASRAMFGIGL

CRNEAFNKLTIEFSLGFAKAGFAGMLLPNSVRNVLRPLLSRRPRESAKRITEIMRPELEKRMRRLESNLKAREEGRSGDI
VPLPADTLSCLIEETHKHGPDHFTVENIAGCCRIYSFVAIHSTNLCIFHSLLNLASLTYQQDDGQRASYVPSLRDELSEI
YASEVLPTATSEARHIDHQNAPKLHPFSKGWTGAALDGLRGLDSFVRENLRMKMQGPTSLHRTVAADGGITLKSGLHLPK
GTFIAVPARSIQIHESNDPDAAEFNGWRTGRPGQADKGDAATTTSSTYFPFGTGKHACPGRFFAVKEIKLVLKHILLNYE
IKEIEPRPKDPNLFVYQIPPTRGVSPILLRRRKVQ*

>CYP6958A1|154106|Ascim1

MATMKDSFSLLSARVFSQLSRLDLNLDVKSLLLIHLPLLLFTIVAGKIIYLRHFHPLSRFPGPFLGSITNFYQLYIITTG
RLEQYEAAWHAKYGPLVRIRPNVLSSSDPRHGPYIYHKNADKTSFQDLPSFGLEESVGSQKSHKVHAYLKKRVAPAFRLE
VVKRSENMVDDQILKLLKEWDRRYLKESKDGLLDCAPWTQYLAYDVISEIAFGESKGFLKKGGDVEGLITELLKAIKAGM
ILSAVADLFLTIYNEPLGIGRWLLKPGVDPKSGLGKIVTFAQNQITTRRNRIAEGKPVRNDVVNHIMSLQNPDGTLVPDS
YIRAELTLVTFAGSDTTAANIRHIIISVLSNPSVKAKLIAEIDGNYEAGRLSGMVGTYDDLNANSPYLYAVLKESRRIRA
PIPILLPRQVNAPGLHLPVGHGSEQTTVFIPPGVDIGQNSMVTTRDKHYYGPDADEFKPERWLETEVGKAQAEKLERADM
TFGAGARVCLGRNIAWMELIKSVSALFRYFDIERVEPPAGGKPYKCENFSLWYEDGIWIRIRRRDVREWNMVDAAVV*

>CYP61A1|157357|Ascim1

MATSTMAGASNYSADPVQAGDLSRGNWNQSSGFVGQLLAGVSGWTILFTILAVSVTYDQVMYILNRKGIAGPTFKIPFMG
PFLESINPVWEKYVAKWNSGPLSCVSVFHKFVVLASTRDLSRKVLNSPQYVSPCVVDIAKKLLRPTNWVFLDGKAHIDYR
RGLNGLFTRQALAVYLPGQEEIYDRYFKRWVQLSKEKPEMAYMGEFREINCAVSCRTFVGHYMSDEIVKRISDDYYCITA
ALDLVNFPIIIPFTRTWYGKKAADMVLDEFEKCAVLSKEKILAGGTPDCTLDFWVQAMFESEKAKEAKERGEEYLSPNGE
KPPSIRIFTNYEIAQTLFTFLFASQDASSSATTWMFQILADRPDVMERVREEQLAVRGGDPYKPLSMELLDKMIYTRAVV
KETLRYRPPVTMVPYEVKKDFPVTPGYVIKKGSMVVPALFPALHDPEVYNRPEIFNPDRWLEGGEAEAASKNWLVFGCGP
HYCLGQTYATLNFMAMIGKASLMLDWDHHVTDLSEKIKVFATIFPMDDCKLTIKERLPMH*

>CYP52AP1|189391|Ascim1

RANKARAAGCRDPIRIGTSRFFGFRTLQTFLRAAKNHAVTWFFVDSFKQYGNTYKMKMFGAGDSIFTIEPDNIKAVLATD
FEHFDLQHRYLALKPVFGDGIFNADGERWKYARGLLRPQFVKKTIANLSKIERHFQSLFTRISKDIKDGDIMDGGKVDLQ
PLFLRLALDSSTDFLLGYSANSLLQTQEEAAARTELPKENAPYKYGFMEAFDKTNSLSMLAFRLGALRFLVHWRKDFREC
KRIVHTFFETQIDRGIQLRKELDGKTSDAEGNYDNFLQALLSSTDDRLLIRDQLTNILLAGRDTTSAVLSFTLFILARHQ
PAYNKLRSEVLAAFGRDTSNFSFQSLKSCTYLQWVLNEVLRLYPAVPTNRRNGNADLILPTGGGPDGKSPIFCPKGTQVY
YVVFALHRRKDIYGSDADEFVPERWDSTKRKLPVGWEYLPFNGGPRICIGQQYALTEIAYIVARLLQVFERV

>CYP50194B1|202846|Ascim1

MLDTTLVDIKSGLSSLSSNLHGVTITVEDVIRLIPIFFAAGIAYFLSDAIYNLYFHPLAHIPGPFWAKISNIYCVRMFLS
RELEKKEWALHEKYGRVVRIQPNQITISDPRFIPAIFHKDADRPMWKEMSLLGLGGTSFNAKSSTDHKKARRIAPAYSM
NSIRKMEHFIDTRLVEMLGVFDERFVQQKQLCDFGAWSSYVAYDIVSDIVFGKRFGFLKAGADVGDLIKNFQVALPAVGV
LNRMRWINDLIMAIPGFGTLMKPSPSAKTGIGALMRFRDRLVERRYADIATKKRPDLLHNFINATNDDGTPMSLNDVKAE
ALIVMFAGSDTTASLMRKVIFYVIEEPSIYRKLRYEVDKAYTSFGPGIPSFPELVNLPYLAACIHEALRLGPPSPHPVQR
LISAGPGIQCHGYHLPPGTIVNMNPWVVGRDKKLYGPDADQYRPERWIEADLETKQLWDKLEFAFSSGPRICVGKNIALM
EVYKVIALLIRYFDLTGSGPPPGVSTYQREENLSVVVQEGFWLKLRRRDVKEWEEVDSFFARNEVMEDDKDIQL*

>CYP51083A1|207169|Ascim1

MDSTKLFTSDATFWLQSRLPSSSLSWLSALTIYDVLKIAGTLILARTILVSIYRLYFHPLAHVPGPTLAAISPFYELYYD
VFLGGEMTNHCMKVLHPKYGPLVRTSPSKVRSNDPESYHVIHAMGYKTERDPNFAKVWNLHRSIFGMTDAKLHKSRKGLF
GGLFGRQSLAGFEGAVQGKCKVLVDEIKRRCDSAVKKEQTWEWVNVRNATCALTMDVMMEFLADGIGSYNLLESPNFYGD
IIESIDLTTQLPHLFRLVTPIAMFLHHYCPKSLKYTLAPYLIQYENLENRVREGVDKYKEAMESGKAKPTQYLVAHRELL
PKFDINAEDYSIEAMVMFAAGVNTTAYTIQYTLYLLAKDKVIQQQVYEELRRLFPEKVSGQDTTDSFDFRRLESSKLLFG

VIQEGIRLGDPLPGVLPRVTTSHGLNVNNTYLPPNTVIDTTCTQMHQHPAIFPDPKKFDPTRYSGSPERERLSKYIVSFG

GGSYTCVGKNLAMMELFVTISRLVWEFELEVGEGLKGGWEAKDRWVAAKVGGDAVLGFRKRVHA*

>CYP50251A2|210673|Ascim1

MKNLIIALVVYKVVSFTLLAIYRLTLHPLASFPGPFLARISPLYEYYFAFCKTGVIQHCHDALHSKYGPIIRTMPNEITI

NDPEAYHEIHYVGTKFLKTDFYLGHRGFDSTFGSIDPKLHRKARSNVAYMFARSTVAKHEPVVVEKCGLATARIEREIGK

AGGKALVEMKPLVYGLLFDIVSDYTFGGKFEILKQEKIHHPIVESLEAAIEAHEILRYFPGFLEKFEALMPIWLADKLGL

IAITGQLELRTTLRNEIRALNAKRESGNLQKNEGKPTVLSDLLDKGVDVDALGRESNTLLGAALDTTSWTIMSSVYQMAK

NKDVQQKLYEALKELSPNRDDFVTQEQAESHPYVVAVLKEILRTTTSVMGKLPRYSPPGGATVMGTFIPEGTIINTSVYL

IHNNPKVFANPEKFDPERWLQPSSKKLERYLVSFSAGTRACVGMHFAYLNLYVGIAALYRKFEFDLTEEMKDEGWLFAER

LVAVKRGLEPNFLVRRRED*

>CYP677G1|213062|Ascim1

MAVIDLYDLRASLLSDEHFTRTILFRLATFYLLWKAVATTYSVFFGPLSKFPGPRMAATTQWYRTYIELVKNVSMSTKLK

ELHEQYGPVVRIGPNELHFSSPTAYNAIYQQKFDKEPILYKSFGEDESSFGYLSVKEAKPRKDLLNPLFSRRETIRMQGL

VQEKVDKLCKNMVKRYEEGKSCNMLMALRCAAMDVITTFCFAKSVDSLDEPDFEAPIIIAMESTLPSFLLFRHFPLIRWL

VFSLPPWLTMVTAPKMKGLLDLQKMLSGQIAEVKADPSVLEKTPHRTIYHPLLQEANVSAKSLYEEGQAMLFAGTDTVSN

TVMLAIFHILENPAIHKRLQEELIEFWPDLNTTPRYEELEKLPYLNAVIKESLRHTPAVAAGLLRIVGPESANIDGYEVP

QGTVISMAILFMHMNKTIFRHGEKFVPEAWLEPGAKELESKLVPFSKGPRMCLGQNLAYCELYLMFANIFRRFDLTLDGT

VASDLEWRECFLPWYYKRHIHCYTKPRTA*

>CYP51082A1|216741|Ascim1

MGVIEELLATQLHNFSFSDFAIAVGATFALYRLAIIFYRIYLSPLAKIPGPKLAAATSLYQTYFDIIKNGTFVFHVPQKL

HPKYGPTVRVGPNRIRLSDAESFQQIHKIGTKFTKDRDFYLMFGHDYIVNERSNEIHRKRRAPFANSFGPAEVRKQEPLV

REKMERLLNKFDELCKDGGEKASVNLDWAIQCMALEVFFQFSFGTEVEKYIDTPGFAHSIISDGEPFMNQNIRSRTFPTF

YYCVSPWFPEWFKNLTMDSWMVQIRFKAMGAGLIRDYGKKYLEAKRSGKELTGPDGGPKKYTYLDGLLDRLKEGEELSEQ

VVYDAVFIFIASFATVASVINQAIVGINRNQSIYKKVMKEIDEAYATEKVQKNGGYIDSDTIQGMSYFQAVYKEALRLAV

AASGPLPRVTPPEGATLAGHYFPGGTIVEHAIYTMQTNASVFPDPLVFRPERWLASPKEVAEMDKYMIAFGGGSRICLGQ

NLGAVQVQVTIVGILRRFDVELSKGLQEKGIEFLDKWGSLLISDFVCTIRPRKV*

>CYP50194A1|220867|Ascim1

MLADLKSELLNLRTIHAVLEVYHIIRTHWALVLVGILIFRYIRRLYFHPLSAFPGPFLARISNVYSMAIYFSWREHEIHM

ELHKKYGPVVRLSPDALIISDPKYLPIVYHMRVNKPDMSETQGANESVLGAAHYKEHAKRKRRVAGAYSMTNVRRMEHHV

DGHVIRLIKTFDDLYAKTGKVFDYAPWAQYFAYDVVSDVAFGKPLGFVETNSDVFGLLKMFQQGIPITAVFTKLPTLAWF

FRVTGLKGLLKPKPNQNFGIGGLMHFKDKLLAERYEEFKKGNGGRNDFLKFFFESKNEDGSPMSLNQVGAEALVIMFAGS

DTTSAALREMINFLHLNPRCYKKVVDEIDAAYAEGRMTTPVPNFDELSQLTYVNAVFKETLRLAAAVPVAIQRIVDMPGF

EIDGKFVPGGTLVGINSYVCARQKSLYGEDAEEFRPERWLEDEEKRKELERYDHSFGYGSRPCLGKNLATMEILKVLCTL

FHLFEVKEADAPEGCTYKREKKNIGLMLQRGIWLKLVRKNVEAWKAMDQAVVA*

>CYP51088A1|222449|Ascim1

MTARSHSHSATKSKMLVTYALLLAVALLLAQRLSANTTEYKKLKEQAALYGGSWIPGGWVLAGIKAFLYVPEYIRTGYHE

YNLIGKPFRVPNFFRGDIVFLPSSALKEVRSASSSKISNVAAIIDTLAIDKVLINKELQMHPYHIDLTHTAITKNLASIV

PDLEDEITHAFNTLIEPKLPEIEDAEGWTKLEWEDLKEIMDRTANRTFVGFPQCRNRKLLRIVNDFTSSVVTTGIIMGFF

PMFLRAYIAPFAALINNLRVKRVERMLAGIITQRLELANQFKERVEDVGSQQELPNDFLQALVNTAIEHGEGNVSNITLR

IMVMDFSSIHTTNNAIQNCLLNLASTHDYQSSRLETYVEELRTELLAVYADSDVKPKPTSYSQSFHGKTTGWTLNDLQKL

VGLDSFIGETLRLHYLNPGSVLRKVTADHGFDLKSIDLHLPNHTIFAFAGSEFNQENYEKPKQFNGFRSSLPYQYIKEQE

TIDAIELARRHGFTPNGRTSTNTGHSNFLSFGTGRHACSGRFYAVAALKLMLKHVLLNYDVRLEGGRKSRPECKRFFLYQ

LPYEGAGTRIEIRRRRKTSQ*

>CYP548BE1|230281|Ascim1

MLTLPANFYVPLAIPSAGSIANFFLLLVTGLLSYVAVVAFYRIYLHPLSSIPGPTLAKVTSLYTTYYAYRGDRFLNFYRL

HKKYGPVVRFGPHHVSFNSATAMQQIYGHGSGGRHFRKSDFYKAFPAVKGVHNTHNSIDKLEHGRKRRVLAHAFGEKALN

AMEIDGVLTHVREFLGIVDKSVDEKRAVDIGEASNWLSFDVMGELCFGKSFGMLVGEGQRFVSKMIEMAATNHYICGNFL

IFHTMKLSKFLFPEIAKTRWRFILHSRECANERMKLHEAGLDADKRDFFHYLLNAEDSETGSKFSKLELWGEANVLMIAG

SDTTATALSATLFYLVKHPEILDELRKELRAKFSDIEDIAMAKGPALGQCQLLQACIKEAMRLAPPVPGVLPRECVSDNV

KIDGISIPQGTVVGVPTWTIHHNPQYFPDPFTFNPYRWLSTDKSLCDQNAYAPFSIGSRACIGREMAMGELRVTIGRLVF

GWDFKEVPTEGKGELWHRGFKGGEGNEPEFRLVDGFTSRKEGPVLRFERLLPVA*

>CYP51061A1|238401|Ascim1

MFHLSVLQLMGTLFAIYIGKNVLDLYRNVQEIKKTGIPYFIAPVCPHRMLYRYSSLFWGPWVENIPGFSFVRYTRHNWNW

IRKWEPFEKHGEIFACVSPGATQLFVGDPVAMWDMANKRVEFPKDIGLYKAIDVYGKNVVTTEGKEWRLHRKITAPPFSE

KNNSHVFYESLRQAHEAAKYWTAPKNQKDLGAKKPVNTVSTFTDDFMRISLQVIAKAGFSKSLQWPSADGSIKGEVAEGS

HMMTFQGSLGMVLHKIIWYIATPKWLLRNSPFKFQQDVYTAVNEWREYMLEIYREKKQRLDKEGPDALLHGGDLMDSLVP

REVPEGTPFADGKKDGLTLPEILGNTYVFMLAGHETTAHTMHYTLIFLALNPQYQRKIQAELDALYPDPNTELNYERDYP

ALADGWVHAALNETLRLYQPVIHIPKTSYEPHLLKTRERGDIMIPPNWNIEISTMGAHRNPRYWSPHTDAYMPERWFSSK

EAEDVSTWRPTDKDEKRLKKPVNGSFMPFSEGFRACLGKKFAQVEMVAVMAEFLRNYSVELAVDERKESWEQVAERTKKD

FDQCFITITLSLTNTIPLSIVRRGEERFAGKVGSL*

>CYP50030A1|238985|Ascim1

MEAFQNLKNQSEKFLASNSVDLGFYRSYIDPIKQHWIITALVVFVVYEVGVTFYRLYFHPLAKFPGPKLAAASTLYEFYY

DYIGPKRGYLSQHVLDLHDQYGDIFRIGPNKLRVNSLDGYDKIHAVGSPFCLTYFYDGFQLDKGVLCLKDNKVHGDRRKL

MNNLFSKKEMSRLMPLVRSKTQLFFDRIMKDCQEGEGETVFEAHHRVQAIASDIVMEFSYGRSYQLLEQENQRHLMNIAM

DQAINTFPLGKYIPWYSAILQRLPDSVNEWIFPGSSGFSTPLHLLPFSNSSTAAFPRSSREMGKEIEALFKKKEAGTFKK

DPNAPATIYYELLEQYDDPEALLQDATLMYSAGVDTTTWFLSCAFYQLAANPEIERKLLEEIKTVWPVKEQRPEILVLES

LPYLAAFIKETRRLSYGVLGALPRLSPPSGATLSNQFIPGGVVCETSSYCIHHHRAFGPRAETLEFRPERWLDPANKHLE

KYIVNFGSGSRSCLGMHMAMLEIYHVLPGMVRLFEIGLGEKLKKEGLQWRDRWLPLKIMEDVDFVLKVREE*

>CYP51042A1|242779|Ascim1

MFFSFQTVRGLTFLQLCAISIGLFFTHALFTAIYNVYFHPLRNFPGPFWAKVTIFYEYYWDNIQDGQFTNHCYEVLHPKY

GPVVRVAPHKLRVNGTGDFFKIHAMGTTFTKDPHFYSHGNREGVLALLENKPHHERRKLLAPLFSKTTIAKLQPLVQEKI

DRLLIRIKADERKAGLRRDRYVFDCFRYFQAIVSDVLMKFAFEKSYDALGQESLTHSLLESSEKLIENIPTFKYLPGWAH

KIIQAVPPWLAQIILKPIGEHSRFQAQIFTEIVDLFKRKNAGLLKPRAANEPPTVFSELIDIYPSEHALSNDSVTIYSAG

SITVTHNLSHAVFLLAKNKVIHQRVFQEIKAIWPTLETRPSYEQLEKLPYLTAVVKEVLRLSPGVIGGIARRVPKGGVTL

SDRFIPEDTSIDSSIYNMNMNPDVFHDPFKFDPERWLQPDSILLEKYSVSFGLGSRNCLGIYLAYLEIYMILAAIIRNFE

WECTEVCEREGWKWVDRWSPAKRGDPGTFYFKSRAE*

>CYP51080A1|244133|Ascim1

MEKVLLPVFDAAKKLTVTPASVLGAVGTLFLCYHLSSIIYNIFFHPLRSFPGPFLAKFSRLYELWYDIPKGGQFCFHCEE

VLHPTYGPIVRTGPNRLRIQGIDAFAHMSRMGTRFIRDEKHYQSNGTEDTMVTTPSNEDHRQRRKYFEPGFTKNEVIKRE

PLLRRKFERAFGKLEDIMKKNGGVAEVDMNFMFMCTALEIFSEFSFGIDSTEFLDAPNFAHPFVVGIPKIEEDFNVVKHF

KFFLITLPGTLPKWFRDKANIDKYHKQFLAEQAGGLRTRSEKGKPGFKPDTILDSVFNQLGTSGIPPTMYADIGITFAAS

FGTISKSSACAITHINREPRIKELLLAELRSITPNSKEWITYSQCEHLPYISACVKETLRLAQPVCGALSRISPVNPPNT

PTVFHNRVIPPNTNCECTIYSNHMNPKVFPNPTEFLPERWLLSHATQAEINERERWMVPFGRGAKICLGMHMAYMQVYLT

IANFSSRYEVELNDELLKEGYRFNDKFFGVPRSNLRVKFRERGD*

>CYP50194B1|244282|Ascim1

MGILTPENLREAGNFALLALSFLKSNFLYILTALLLTRAIYRRYFHPLSAFPGPFWGSITHWYSVYLFFAGGYKMHLHEL

ELHKRYGPVVRLKPDMLLIASASYLDTVFHLKCDKAAFIDIEIAGFGDAVITARSHASHVRLRKRIASAYSMTNIRRMET

SVDAIVVSLLHQLNTRFAAQKKACDFALWAQYFAYDVVTDVAFGRAVGFVEAGKDVAGLVGNFHRNLPVGNILVRVRWLA

QLLAMVPGLRWAMPKSGDEVGVGVMMTYRDGLIRERIREGNPEKKPDLLHHFLNAKNDDGTPMTFREIGDECYLIMVAGS

DTTSAVLRLLTRNLYSNPAIMAKLYAELSSARLSRPVPTFDQLVKLPYLQAVLSEALRLGSLGMYIPRAVSEPGFECHGK

FVPGGMAVAMNPWVLSQSKEIYGEDASEFNPERWLRGEEEKARLAKYEFAFGRGGRSCLGRNIALMELNKVVAGLVLGFE

GRLMREGEEGVEYPGELRNLSIWAEKGMWVEFKLREGVEEGWAKVE*

>CYP51087A1|245242|Ascim1

MGLLKFLTDDARGSLSPLHLLRSSKLLLTVLASIFILYRISVIIYRIFFHPLRHFPGPILAKVTTAYQVYYEIVKDGNLS

THCHHVLHEKYGPVVRIGPNRLRIQSVEAFHQMTRIGTPFTRDSSTYIMFGFENMMADPSNERHRERRSIFQNAFRRSEI

LKAEPLVTRRMKRFMRKLDEMCKESGGQKELNVLWALNCQGLEIFTEFAFAQDSRYIDTPEFADRFAVGLRDVLDATVWT

KAFPGIFLTFRDQVPVWLRSILFPRSGILNSMLEYSHYAVDSRRANFYQDSKADNTAGDKGQNDFRPHTFLDKIFSEHPE

NHTFPPEFYRDVAQAFAASFGTVAYTTFSGLYYLQKYPEVRERVLRELMTIVPAGKRGEFIDHNTIQEKLPYFCAVVKEV

IRLSLTIAGAFPRITPPEGATLLLPSGPVHVPGGSAVEVTVYSAHMDPVAFPEPSKFDPERWMDKREAMRLEKHIILFGA

GSTICLGMHMGLMQLHMNLANLIRSYEIELGDELKERGLVWVEKWAAVERVESLMKFKVRRE*

>CYP50194B2|302258|Ascim1

MALLSAENLQKASPYAFLALNFVRANWLYLLVGFYVVRAIRRRYFHPLSGFPGPFLGSITSWYSVYVFFSKNYNMHLTEV

ELHKKYGPVFRLKPDLLLCNDPKVLPKLFHKNADKAAFIDIEIAGFGDSVLTARSSALHVRLKKRIAGAYSMTNIRRMES

DIDILVLELIKQLDTRFATTGKACDFALWAQYFAYDVVADVAFGKPLGFIEAGSDVGQLVENFHRSLPAAGVMVRAHWLA

DLLVKIPGFTYLMPKSGDTVGVGALMTFRDKLIKERIKEGNSGKKADLLYHFLNAKNADGTPMTFKEIGDECYLILVAGS

DTTAATIRYLIRNLIKPGNEAMLEKLHAELSQANLTRPVPSYDELVKLPYLGAALYEAMRLNGLGMFIPRAISEPGLEVC

GKFVPPGAGHAIAMTPWVVARSKEVYGEDAEVFNPERWLGSEEEKRNLLRYDFGWGYGNRTCLGKSIAMLEMTKVVACLL

LGFDMRLMKEDEGVEYPNQLKNLSVWVEKGMWVELKRKEGIESMMFGQTEKVKA*

>CYP51069C1|302619|Ascim1

MKDSPLNLNLLLPPGFLQKWNSTLSSTIPADLKLAIPPISTLLFVYLPVTLFTLLAARIIYLRHFHPLAHIPGPFLESIT

NLTRMYRMNTQSPPIYEYNMHQKYGPIVRIGPNFVSSDDPRHINIISHAKVDKPSYLDMPGLGMEGSMAGTRGYREHRHK

RRALGPAYTLSTILKFEPNIDRLIYKFIGKINRNFASAGKEFDITHWNMYFSYDVVSEIAFGEARGFLDMEGDKDGMIAS

LHEAVRTGYSVIAMADTIYAVYKSLPEKFANWLLIPKLNADAGIGKMIAAAKKYVVEMRAKRASGFAGKHIVERIMEMTD

ENGNPVTDDYIVAELISIMFGGSDTTAGSLRHFYELVHSHPEVLTKLLAQIDQAYETHGTGKPVLPYEALTKLTYLHNCV

REAQRVHSPAGSLFPRMVSEPGLDLSGFHIPAGTEIGQTPWVLCHNKECYGDPENFRPERWEESPERTRELERWESTWGF

GPRRCPGQNLATTELWKIGSVMLRMFKWTRVKAPEGKPERTLWQLGIWFGEGYYYKVERRDVPEWKTFEKMMAEGVVA*

>CYP548BD1|317371| Ascim1

MGLIYNLLSKEAAVVAFSCGLLYVLGLAIYRLYFHPLAAYPGPLLARITSFHAAYHAYIGDSHLVLFEAHRKYGEFVRFT

PNFISINSSEGLRDIYTHGKNVQKSSFYSVMPPYPHAFDTHNVIDKSLHGRKRRVLSQAFSEGALRTMEDQVLEHIRIFC

KGLGAANTVVEHTAEKKLASAPETPSEPRNMSFWSNWLTFDVIGDLCYGKSYGMLEREEPRFWPFLIDYAIHRHAIAGIS

MKIHKTGLGNLLFPKIAAGQKTFVQYARTQAAERIALGKNGRKDMFYHLLDARDPETGNGYSLSEIWSESNLLIAAGSHT

PSTALAAAFYYLTNYPEVLAKLSNEVRSKFSNLEEIRSGNLLGSCHYVKHCLDEAMRLSPPVPTTLPREVSEGGAWIAGH

YFPAGTDVGTPAFTLQTDERYYNEPFKFMPERWDASIVGEEAVERATSAFAPFSLGPRACIGRKLAYMELTVALARTVWL

YDMRISTFTLLMIIILRLAPERGHTSHSDLGRESLEMLLTVP*

>CYP51060A1|317389|Ascim1

MSWQTLILGALGFWLVKRIVLTIYRLYFHPLARFPGPPLARASTFYQFWCEMILPGVFNTRTFDELHPKFGPIVRTGPNR

LHFSDPAVFHQIHYTGSPFPKDPAIYGTIGPQGDLFSLVDPKEHRQRRKMLSPMFSRSQILKVEPMVRNKVGILLDRIDA
IIDGNDQIGGRKEGEFGWISMQKPFQAMVADIVMEFTLFQSYNALSEPNFENPLIIALFTITTTNRLFRHFPLLFKLPQY
LPLWLVNRLSPVAFELANWQVQLRKHLQTQLALIETQKFHKRSYNVLLSSVLDQYPSDLNLLTKECTSIVIAGIVTTPLI
LTHTLYRLALDPTLQARLLAELKSALPTLTTELDLQIIEKLPLLRATLLESLRLAYGIVAPLSRLTPPAGATLAGQPIPP
GTAVDISCYAIHHNEELFPDSFTFNPERWMQGEKSKRLEKYIVSFGGGSRACLGMQLAWGELYLAVAGVVRRFEVTTTRE
VQEGGWEWEDQWSAEGRGVEKVFGFRRRGE*
>CYP51065A1|320304|Ascim1
MLHLGLAKRLPDDYLLAQLGNNVAIPVIVAATFAISFIYNVFIYPFYLSPLRNLPEPPNGHWIWGHTREIRDLPKGVAHE
RWMHTVPNNGLMRYRYLFNVERLVITDAKGLQEMLVTKDYDFKKPHYREAVVAPLLGAQSILFIDGDAHKAQRRHMNPSF
SQRFIRTQLPVFWRYANLLGARLIEDNKLDSYPTTQPAPAFDMQKWSTRITLDIMGDVGFSTKFGALTETGTPIATAYGK
LFGSSPSSVVRLLKMLGPVYNFEGLLTLLPSKHKKEFLRYLGIVRGACRKVVQKNRAEREFGAGLGKESLSEKEARYDDA
EYVPRESSIIQTLLSEEIGFSDDVLVDQCMAFLAAGHETTASALTWGIYELCLNPTLQRILRSEIRGFGLHDPSINTHSM
PNHQSLPQLYAFVAEILRFHPTITLNHRESLKDNTILSQPIKKGTIIKIPVLGFNHSVHEWGPTAGTFDHSRYLKQDGHG
NVVYDGTGKAKSNWSLQTFNHGPRSCIGKEFAKEELTIVLAVLVGRFEWELEREGQVVEIERAAMFKKIKGGLMVRAKIV
V*
>CYP540A14|327254|Ascim1
MAIQKFFLVGEDPKTALDIEVNPSMEYKTLQSGIATQYHICEAKGIAFQNDEGPLDSVESILDSRSPVGITVDGQAVKSP
SGPEGFPFVGSFYEIFPDHLGNHARLFRKYGSVIKTTNLGKTTYLTDSPEVAAHALSESIYFTKLINSSHPLWGVKDNTA
IFVNDTETENWRLAHKFLPPALSPKAVRHYTPLMSSTARASFPVFDELDQRGEAWSVYHYMLKLASQTIGKLTLDLDLGQ
FSSPEAPLHPIVTSIAEYLHLNKRVTSRGEWYRYLPFGDGAKLRHVQQEVYSIIQSAMDDCKATSLASGEKTDLPINDAA
LSARCVLDYILRAVDEKGEKLPEELVIGNMMIMTGAGFTTTSSLLSWLLYSLVTYEGNQDRLLQELVDNGITNSTIWDPD
LANTLPFLDKFIKETQRLHNPSYQPGRTTKQEVIVPGGYRLPKDAVIISAIHAIHTNPNVWSNPLRFDPDRWDTDEVKKR
HRCAYIPFATGPRGCIGFNFALQEVKVIVSELVYRYEFSREDRDAIEYDPEFQLIRPLNLYLRAKRRTTWPEPTPGSKAA
QVAARL*
>CYP51055A1|327684|Ascim1
MELPFLLAAFLSVFVLFIGFVWNKYLQPPSDFPTNLPVIPLYFSLLGYLTDRGQDELYNQYLRDPLNEFGAVIIFFGSRW
NILVQRPSYLAEIFKNSGEYAKSGNQKKIPYSVVAALTGDNIISAHGDNWNLYRRIMKKGTQRKLDLQTVRSMRLKVGKV
AGVLREQNNRGLEVVSLDNLIQRLSMDMLGVGMLETDFQTIEDPNARIFYLHNNIKKAIFNPLFLNFPILDRIIPTRWRI
FKLIQQLEEELLSRTINICNTSSSSRTKRDTPTDIPLEPTSLITDMQDALFRSSWTRRQCLDNLKIIFIAGHENLHQHLV
SLFFLLGTHPHAMQTLHKELSKAAHDAGHITNDPLSTEALDHLPYLTATCYEALRLLPPIPQLINRKTTDPVLLGGKIPI
PEGTYVGWTAFGAHRDTSSPNPYPYSSTSLDKFDPSRWGNTIDEIRRVHRRTQARGEFVAFTGGMRACLGQAYADDVAKL
VLDAMVRGVGMWEVVGKEDVKMTPGGLLRPRGLRIRFVEK*
>CYP51054A1|334460|Ascim1
MFLAWRRHQALKHIPAVGIPPGWGPLGPYLAARRFISDSPGLLIEGYEKYSPTQQTFKIATPTRWVLVATSPAIIRELGT
CNEALLSQQATANERNSMSYILGPSIHGNPYHMKIILRKLTPHTGAIIPDLVEEATWVLNTHPEAKQLVDEEWRGVKLQG
LITGCISAMTNRVLVGQDLARDEEFLGSLADLSMVISRAGVAIDLAPDVAIVKDILAAILVPKGRGPFKVFLDKLTPVFE
KRRKEYGQDKERYDDLIQWVLDAAPEEASMYELCTRILYLNFSAIHTSTVSVMQSLYDLASRPEAQDQIRDEIQMALSEG
EGWTKESIASMWKLDSSIRESQRLTPVTTATMMRLAQETYTLVDGTVLQKGQWIITPAWAANRFTTAFDNPHEFDPFRFE
KMRSQPGQELKHQLALPEEGYYSFGGPGKHACPGRYYAATEIKVLVGYIISNFRFRISHDSITDPEKQKNNFLSFSCLPN
FNDGLEFQRRETTCST*
>CYP51079A1|335701|Ascim1
MERYIDLKQPMATYPSQLYTHITQTYTNKQIFLAVFAIFLAYKVTDTIYVAFFGPLSKIPGPFILKFTEMPDVIGNLRGR

AHESRIAYHLKYGDVVRIGPNTLSFGGADQVKQIMINLDLPKGTVYKLFRPAGGRVNLFSETDKAAAKVRRRLMANGFSV
SYLKNVHPLILKVVETFCDVVDERIEKDGKDGEVVMDLYHPLSLFAADVIGESAFGSSFDLLTNSDNETEFANMIGANFR
KAVVGSVFPFIKYIGFNNYEPLIDEYLQRGLQKRQLAGGKRNDLWQYYMDTSEANPKEFDAVSMNMELHLLVMAGGDTTS
RALIFLIRLLVDHPEHLRTLENEITNAIYPNKIPIPASDIKALPVMKDDELSKLSFLDACIHETLRLFGPIQNGFVRIAD
KDCNIMGTYVPTGTNVVANGHVTHLDPKIYPNPNKWDPTRWLPQPPDSLYPDNLHQLSKHHFYPFSAGSRNCIGKAFAMT
EMRFAMVMFLARFTLGDVVEGQSWDFTNFITLHFTDNRYLVRVKRRFRESGKSVDGEQAVVMPKVLG*
>CYP52487A1|360547|Ascim1
MSFDSHLQSFTLTNILVTTFTLLLVRFTILAVYRVFFHPNRNIPGPLIGRITNLYEYYHEIFRGNGGELSTYAREVLHPK
YGPIIRTAPDVVRIADKDAWFKIHAIGYTFNVDPQFAGTFGSTASFFASSDTELHRKTRTILLPYFTKKAVFEAEGQVQE
KLDKFMKILRNLTATDEDGAGASVNIRDAYTCYLTDFITEFLFEANWNTMEAPAFHHPIADLNHLEIPPLRRALPSVVAS
IYSMPPWLSVHLLPTMFHGTIYLRRVVHEQMKAFTTSSYHKGPSSDAGIDAPPFTSGNIGNGISRKLIDKLPFFQLGEES
TQLFGAAVNSTGWTLCSILYGIVATEGVQERLIAELKAAYPEKVRMDYQTLKELPYLTACIKEGMRYSSSVPGALSRVAP
PTGATLCGYYIAPGTMIETTIASMHHNPAIFPSPKKIVPERWIPSETPFPEAPDNLERYLLNFSSGSRMCLGYNLAWIFL
YDITARLVREFDVQLGEGLKKDGDWLMADRWAAAKRGDPGIFILKERDE*
>CYP50030A|360698|Ascim1
MESITSSDLRWWAQEQLSSLRSILDSSHPLVKSALELAEKHWIATVVISWIVYEIGLAIYRLYFHPLAKYPGPKLAAVTA
FYEFYYDFYATPKGQTHEKILSLHDLYGDIFRLGPNKLRVNDLQAYDKIHGIGTRFILSDFYKGSQMPKALFTQEDNKVH
RERRRILSPLFTKTETNRLLPLVSRKTRMFFDRIERDCQATGKVAWEAHHRMQAILSDIIMEFCYGRSYNLLAQEDQHHA
MNEAMNEVVKKYPILKYVPWITGIFKALPFAVNEWLIPGSTMLDQAALTLAKELEVQFKQKEAGTFKKDPNGPVTVYNEL
LESYDDKEALVQDATMIYAAGVHTVAHLLTVAFYHIAADKTIQDRLFKEIKSVWPDRDGARPAVETLEALPYLTAFIKEA
RRLSYGISGALPRKTPPGGAELAGQRLPANIVVETSPFSIHHHRAFGTREETRKFRPERWLDPSSRNLEKYIVNFSSGSR
ICLGIHLAMLEIYHILPEMVRRYEIELGDVMKEEGFRFIDSWMPLAERHDLDFLLTVREE*
>CYP51049A1|373664|Ascim1
MALLGSLNLTDALPALRITLQQHWFSILIFLFATRIIYLRYFHPLAKFPGPFWGSITDLYSCYIYLTKEQHLRELELHKK
YGPIVRYKPNLVLVDDPNYLPVIYHKHVDKTAFQKASGLGFSSAVVSALPHKEHAALKKRVAPSFTMTNIRGMEGAVDTR
VREWITRIDQDFAQKKMAMDFGPWSQYYAYDVVSELAFGKPFGFVKAASDVAGLIHSLHITMPGAGILQRIPSLADVLSL
PILEKKMMPNPTDKTGIGAIMGFRNKLIRKRIESGESGSGENGVKDILGHLLAYRDENGNGISEEDLNSELMIVLLAGSD
TTAAAFRNFLKHILGHPDVYKRLQAELDEAYDSGKLSSPPNYDEILELKYFLACIKESMRHEAPVPSHLPRMVSEPGYNI
AGHFIPGGAEIGCNAWVTARHKTFGEDAHLFNPDRYYNASEKELIRLDKLDYVWGYGSRACLGRNLAQVELYKILCEFFR
RFKPSWTVAPEGQEPKEMTEENFGIWIQFGLWTNVERRDVKEWKYSY*
>CYP51042B1|380448|Ascim1
MAVELAKEILQAYQLGRDHLGITLLSAFIAWRIWLIIYRLYFHPLAQIGVPGPFLAKITSLYEFYYDYLCRPNGQLQFHV
GDVLHPKYGEIVRISPDKVRINNLEAYRKIHSVGSKFTKDPAFYSSFGIASSSAFMVDQKDHRIRRNLMNHPLSKKEVAK
LEPLMRETTQILVKSIDEAIVKGGTGFLVFELHRDMRAISSDLIMVYAYGESYDLIRTKGFNKPHPMLDMGDASNENIPF
MKYLPVSKYVKSDFATKVIDWFLPFTGAGESFKTQQRVFERVKKLRSRLKAGLKPLLPGDPITLFSELIDAIEDDNVLAE
ESLLVYFAGLDTAAFALARACYFLAKDKALQNRLLENIRSIWPDPTTPIPHYQEFERLPYFSAFLKEVFRLSYGVTGTLP
RLVPPGGAKLGDYFFPEGTRVETSCYTIHHHPKLWGSPEESHRFDPERWLRADSKELEKWIVNFGYGSRSCVGIYFATME
VILVLCTMVNNYEIELGETLEKEGMIWVDKWLPINRNKWEVKLRKRSDNESS*
>CYP50251B1|410231|Ascim1
MGVSSTAIFQQLNDRAPVFIPVDRAEQIFRDVSPHITWSNMLLAFVSYVVVKFVVVAIYRVTFHPLAGYPGPFLAKITNL
YEMYYAVFKDGQYTQHCYDVLHPKYGPMVRTNPFTVRVSDPDSFFQIHHVGSPFLKWPEFYLPFQSPHSSFGSVDPKIHR
TSRSNIAPMLSKSAVKKHEHVVIEKAKDMLGRLDRFIAECGGVQGRYNGRTLYFAYLFDAFSGYGFGQQYDLLKLPVIQH

RIIDALTQGAEATMFIKYFPIMMTILEKMPFWVVHKMSPAAVGQEEVRGSIRVEVDRLQAKKDAGELEKKVTVLSSLIDK
GVSPKHVAEEGHTLVAAALDTNAWTLQGSSYHLAANKDVQEKLYRVLKEAAPGKDEVPTWSQLEQIPYFNAVVKECLRIT
HSIAGPLQRVIPEGASVLNKPLPKGYCVETDIYSIVRNPKLFPNPHKFDPERWMGPDSKGLDKYLLVFGTGTRACGGIEF
AKMNLFIGLAAIFRHFEIDLTKELEEEGWQWTEKWGCVKRGALPEFLAKRRPE*
>CYP51086A1|411143|Ascim1
MKDLFLLTGHPIVLDLYRAYQSITAQHSIFTIAATAFITYFTVYVIYQVYFHPLAHIPGPFLCKISDLGFLFKYYSNREW
EKDMELFEKYGPVVRVSPTCVAFSDPAWTPLVYHSKADRTDHYRSFDQYFGIFAIIPHKEHADVRKKIGGAYTKAAVKKM
LPAFQKRINRWCNNIGDEYADTGKVFDFYPWVSYLTTDVLTEQCFGTSLDISGMRADPNRVTQSLEDAMFELGTMQRWPL
LRNIFWGTPIHKFFTPKPTDKSGLGILQNVANQFLPDRLANPTDAHDLLNVLIAARTDPKTGEVDRERLDKDVVLTIAAG
LDTGAGAIRNCVVLTVETPGVLQWLEAGNFSDRHINAICVEGQRLHNTSQSAFPRVVQDGGVQLGPYHIPGGTEAYIQGS
APGRSKKLFGDDVDVFRPQRWFEMTDQEWAHKERQLFVWGAGTRICIGRYFAEYEMMLFLRAFFQEFEVILHHVGERYWQ
NLMFYDGGIQMELKRKRKGPPLEERPEHIVVDLRP*
>CYP50194A1|411835|Ascim1
MEPGVEWNSLASKAVLAAALLKQYWVHLFFVFLAYRFIQRRYFHPLSGFPGPFLASITNWYSVWVYFTWDMQRWEKELHE
KYGPVVRYTPHLLLISDHKYLPIVYHLAVDKREGFQDMKISGLGDAVITAIDHKDHARLKKRVAGAYSMTNIRRMEHIID
GHVISLLRHLNNRFAKPGKLCDFAKWIQYFAYDVVTDVAFGRAIGFMEAGKDLGGFVDYMRSSLPATAVLTRVRWLQLLV
EATPGMGYFMPKKSDKLGAGAMYRFRDELIEERAKTRDDASQRPDLLSHFLKAKNEDGSPMSLKEIGDESFLIMLAGSDT
TALVFRYLFRQLLIPSNRSILDQLLQEITELELPNNAVPSYDTLQQLTYFQAVLSEGLRLGALPLFIPRRVTAPGFEING
YHVPGGAAVAMNPWVVARDKTLWGEDAEVFKPARWLGISKEEKWHYQKYADFTWGYGSRGCLGKNIALMEIHKATFMLLR
LFDMQLKEDGEDVPYPHVVKNLSLFMEEGMWFDIKAKEEIQNYASVL*
>CYP51069A1|412841|Ascim1
MSDLESFLLWKHGEANRWLQLSQQWFISNISRLPATSHLAPTSTIVFISLTLFTIIAYSIIYNRFFHPLAHVPGPFWASV
TPLVRVYHCTTGTIHLYDQELHRKYGSVVRSAPNLITSNDPRHASKIHHSKSDRPAFFDLPGFGLQHSFATARDHKVHRW
KKRRMMDPYSITNILKSESRIDDQVCNLVSAFDNRFARTSTTFDFAEWTPLFTFDVVSMVSYGEPLGCVSAGGDPHGAYT
EVRKVMKSSFWLVPMGNMIFGIYKSLPQRLADWLFIPNLKNDVGVGRLTKIAKNLIKETRLRQELEARNGGERKARNFLE
RVMELREPDGSPIPEYCIVSEIIATIFAGGDTTASLLRQLIDDVHSSPTVLARLIDEIDATYDSSPPSSSPVLSFQAIQS
MKYLQICVRESLRLFAPVAFPLNRVVSSPGITLDGIYIPPGAEAGNNPWLLGRNEECYEAAETFRPERWLEEPLIGGLGD
KLEFVWGDPSGGRLCMGKQLALLESWKVSAALLRMFRFTPVHPAEKEGKKDREVMNWGFWFAEGFWYKIERRDVPEWKWF
DGQLDST*
>CYP51043A1|412874|Ascim1
MFTLGQTVLATAAASAVYVRFAPAEYRYFSNDKLTVALFFASTLTFKLLYELVLYPICLSPIRNLPGPKADSLFNGNQKT
IKALPTGAPHEKWIKIPNDGLITYKAIFNTERVSPTTVAAMAEVLVHKAYEFEKPWFMREGVATIIGKNGIFFAEGEQHK
FQRKHMNPAFSYRYLKQSLLPIFWSCSTTFIDRLVEEHQILPSSSPEEGFTPFLDIQEWLSRCTLDIIGQAGFGVNFDAM
HKDGSELSRAYATIFGKPGPLTTAWRIVSFFIPVYFTYPLIRWMEEFRTVDRCIEVVRGASKKVVQDKKREIEKRGGRDA
ASAMDGKDKNLISIIMEEMDFPEETLIDQTLTFLAAGHETTATALTWCVYQLTTHPEWQTLIRNEVRTNIPSPTRSDLTP
ENIESLTFLKAFASEVLRFSAPISLMYRQASNDTTIAGHPIHKGCIVTIPVQAFNRDEAQWGPTAKKFDPYRFLKKAPGS
TEESSEYHYDPSGGSHSNFNFMTFFHGPRSCIGKDFAKEELYCVLAALIGRFEFVGDGKEPVIEFSLTNKMRGGLPVKMR
WVAGW*
>CYP51053A1|414174|Ascim1
MDFFSSRIIQHFLATEPAWLLLEILLPITLWLGAKTVYNLYFHPLSHLPSPPFASVTSLVLVYYSHYDLRTETLAKWHRI
YGPIVRIAPNEVSFTSQKAVKEIYQDPEMEKCIPLYGIFQHFGADNAFTSRTRHEHGWRRKGVADRYTLTYVMSEEEKEG
KIRRAAKDYCAFVENEAVPVGEKKNSWDVDLYAANIFYATDNITSHLFGEDLGSHALRQPPFSSTNSVIPADADSMRKRI

FAHYGAALRSKVYLYVAFPAVMNVVDYFRKLRIALFPVAGELVDGLDRMREFGWKRQQYLKESKDTRTVAGKLSMLVDAG
SREWSDEVAGSELMDHILAGMDTTSDTLSFLMYHISHPNHESIQKELRDELRRSITGSPYDAPLSLIMGLPYLSAVIFET
LRVYTAIPVTLPRVVTSPGKTIDGISIPVGTVVGSLAKAIHQDDDIYSGDGQWPVDEFLPERWLTRKGINNEGERIKKME
NRLWAFGSGARGCVGRHLAMVEMKVLLAVVYWTYQTTLVPHTTIKLPHNQWNQRRTFRDTLPFKGANGILRFTRSV*
>CYP51079B1|414412|Ascim1
MRWKLQYGDVVRIGPNTVMVSQVDAVHDAFWKADLPKSSMYHRFRRSPEVVALFTETDKKEARNKRRMLAHGFSVSYLRN
IQPMMLAVVNRMCDNFAGQIDAQSSASANVDVYNILSHLTTDVIGDTCFGESFGLLEDPRNPFSERLTAYFKRNFVLDML
FPFLKQMPIIKNGDPIFNQMLDEKLNKRRNNPGKEARKDILTFLLDAQKQEGFDDQFLLATMMMLLLAGGDTTSRTLALV
LRFLVENPEVLKDLEAEILGYAKSLPKDEPISDEKLQDLPLLNAVINETLRMYPPALSGFSRQADKDLNYCNTFIPAGTD
VTIFIQNLHMDPKVYPNPTQWNPYRWTHPDFVFQKTHFFPFSVGSRNCIGKGFAMTEMRLAISRTIIDFELSEVEDERLK
ASWEITQYLTVHLTDGRYFVKMKKRAGA*
>CYP51040A1|414614|Ascim1
MSNLFNTISTIVTIPTLLLFSLVAVTIHKTHRYFTSPLRHQQIPGPLLAKFTTVYSLYYVLRRNWHLKMIDLHTQYGPIV
QVSPHEVLVNDPKYRDVIYSFASKDKSFLPKAKMFETGKINNDLSFIFERSPQAARAGRKELGHIYSEAGLRLFEVDFDQ
CVEDLITGLKNHHIEAGTESTNLTKWMQFFNFDLATRLATGESAGFCLAGKDLGSANSALRIIMDVVGTLFFVPVTLSIT
TAWVRKMLLNRQLEQIWATSFDTKGSTKEARLEEIRMNKPGYLLARFWQAQEKLRRLYTTGNETEGITTHMFNLIAGAVG
VAPQAHISILDHLLASPPVLQKCITELNAMGTTTVSMADYFPFDGPQQRLPYLQACIKESLRIAPPLGFSLLREAPEGGV
RIGEFHIPKGVDIGMAAGPVHHSTFFGPDASLYRPSRWFETHPSLLNLEGEPLEMKRFIERNWIPFGAGARMCLGRHLAQ
NALMKVTGRMLAEFEIEVVSRPKGWFGFVVHQDGFLVRLKRRRDGDFAVAM*
>CYP51083C1|415120|Ascim1
MEQLNAYRSQAEAFLQTTALPTLHNLKIALQQSLDSKLAGTPLSGFKLDQITPLLAVQALVGLFVVRFVVIVIYRLYFHP
LRQFPGPFLARTTNLYSLYYDWWLRGQYTDHVRTVLHPKYGPVVRTRPHVLRFAEPEAFNKINRTTYTLIRDPDQYGGLN
MEGTSFGADPKIHKSCRTKTAALFSKSRLMQAEPLVHDKSELFLEKVKMRIKRDGKETLLNIHTSMVALICDIVQQFLQG
EVDNFRLLENDDAPSFHTPIEDVLMYAVRYGHFDRHFAKLTVFLQNHLPNSLLVWFTPYRKHFHFMHSKVYSSVLHYKSL
IESGTLTKANSKKLIGHLELVPSFKDNLFVYAFEAQVFLQAGTNTTAYTMGYLLYQLAANPSLQLEARAEIARLKKQTGG
KLDYTTLQTSELLTALIKEALRMANPVPMALPRLATVPVEISGVIVPAGTTCEMSPAQLFHNPAIYGAHCDQFDHTRWLA
SAGHDAETLARRTKYLQPFGAGTYVCLGMWMAWMELYVCTARILDSGLWWELTEELEKERQNGGWRGVDEMSMHKDGLAP
VLRVSVRE*
>CYP52AQ1|415257|Ascim1
MAVLSTELGAFQPFNLLVALTAAYIFYSIITRLYTSHLRKQLAAKLGCQPAFNSTPGILAAIKNSYECLLVFKEIRYNEW
MEAKFTSTGLKTVQQTVFGHVSISTCDAENIKHVLSTRFEDFALGRRNDHMVPLLGHGIFSTDGEVWKYSRSVVRPHFSK
KDIEDFRSIERHFRNLVKHIDARPGLVVDNFQSLFYKMTLDSSTEFLTGTSSNTLAQLPVDGVETKNIFMEAFDKAQELI
VYSMATDLESIIFKLPAWKRARDDVFKFVDEKIQQALAENPEGLEKRADAKPREFTILHHLISDTRDPVFLRDQILSLML
AGRDTTACLLSFATYELARHPELYAKLRREIITQLGSDESRFTFSSLKDCSYLQYVLNETLRLYPPVPDNMRYAVEDTYI
PRGGGPDQMSPVFLQKGQPVNYNVYAMQRDPRIWGPNAAKFIPERWEKGHPEYRKTGAWEYLPFNGGPRICIGQQYALAN
GGYVLARLCQLYERMDTLDPERKDPLMNSSLVMSLYNPAPIKLVRA*
>CYP50251A1|415626|Ascim1
MATSCSNFVGLLDGKLHPNHIDSAQKYCERAQPLLTLRNVALAFLLYQAVAFIITAIYRITFHPLAKFPGPFLAKITPLY
EYYYTFVKDGKMVQHCMDNLHPQYGPIVRTMPNHLTINDSDAFHEVHSVGMKYLKDPGFYEGFRCPDSSFSAVDPYIHRK
VRGLLNPMFSKATVLKHEPVVLEKAALVVKRIEGEIEKAGGNQTLLAMRRLIYAYIFEIASDYIFAGKYDILNQPDMKHR
LLDGLVSAVEALPMATHLPTLAYVLEKLFPYPVAVKLGLDALTGQAELRQGTHGEVKRILAERESGTLKKKTIQGKPSVL
SDLLDKGDEPGRVAREANFLIGAALEESAWTILATVYQLAANKDCQQRFYEELKQLSPNKEEYVSFQQAESNSYVAAVIK

EALRVTTSVSGFLPRVCPPEGATIAGKHIPGGTVLDMSIYMVHNDPKIFPNPKKFDPERWLQPNSKKLDRYMVGFGAGTR
ICLGMQLAYLNLYVGLSALFRKYEFDLTEEMKKEGWLWTEKMVSTKRGLEPDFLVKHRED*
>CYP51072A1|416203|Ascim1
MNSTTAQPLPHTVLLTTTYSYLRTTALLLYLYPLGTLTTAFVLYLTTLSIYRLLFHPLRHFPGPTLAKLSRLYEVYFDIF
SPGFVFHAEKVLHPKYGKVVRVGPNRLRVADGESWFEIHAVTTPFTKDPSFYRAFNRSTSLFSLIDNQEHKERRKLLMPL
FTRKSILQLTPLVRQKARLLVEKWGELCEAETSDDGFTTVDVGKGFMALTADLVGEYIYGESYNILNSADLSHPLLDTVA
AFPSFLYIGKLAPWISPLIERLPERIIGTLNPGTLALKHTQVEAEARINALQASSVPTTPTASILLDKASSPSPFPAQPA
LPTNLITELAPVYPSAHILATDASTILTASLLSTSYTLTHLTHHLALLQESSSAQTRVLSELLSAYPGGLSDFLLNVQLE
VLEKMPYLTAFIKESLRLSYGIAGGLRRLSPKRETKLAGLAIPPGTEVVSSIYTLHHTAEFFPNPDIFNPERWLPTPAAK
EGENNERNMVAFGTGRRMCLGKELALLEIYTVVGAVVLGFEVRGEGEWKWKERWLPVRVGGGEVGLRRRR*
>CYP51059A1|418813|Ascim1
METVFERFSATPQSVTYAVGVVALAIIYNFVSYFRVWTALVDLPYVGLEKFSVPALWKASKKVNSSDVWDLVQEGYKKYG
KDDQPFKLPTFGRNTVFLPAKYWDEIRSLPNDVASFQATLNDTMESKITEVESTEEEMHHSIQLIRGDLTQSIAHLVDDV
QAEAIDAITSNLSDVTDDGWTQIPVFNKVLNIVAQTSARIFVGPVICKDESWIKETIMYTVNVFNAATSLKKLPWIKKKL
FSSTVPEVKTLRASRQKAFDILRPEIARRRAAEKEAKENGTEWKEVDDYLEWIRRRLDSHPTMGTIQYQAKLQLDLSMAA
IHTTSLTLTHIIFDLAANPQYIPELREEIRSVLATTGGKVTKLGMHKMSKLDSCMKESQRMNVLGLSMVSREIVDRKGLT
LKSGLHLPYGTRVHTPHHDMLRDSNRWDDPEKFDPFRFHRLRQTPGKETHGQFVTTASDFTAFGHGKHACPGRFFASAEL
KGILIIILLARYDLRLPEGVTERPESFKSGTSLVPNPMAMIDFKLRPASERFY*
>CYP51067A1|419261|Ascim1
MATGLFLEASNALGYIQSHALQFVLSLSAAWLIKLLYTGYQHRKLLHSLPGPPHSWFWGHLKLFGEAMKKLPPDAHPHFV
HNYISKTYPELNEAGLFYLDMWPISMTQVIITDGKMAAQVTQVQSFDKHPGCYHHSLGNAAGMKAFILLEGEAWRNTAKL
FSRGFSHQNIVDLLPMIIEETGIYLEELGKKVKQVKEEARSGGAPYLRMLDYTTGVTADIIGRAVFGIKLRYQTKGNSFM
TSIVDSLPMLRESPTIPVGLEFRKLRRLATREAKHYIKARWNWIKTGKVVDEFKDLEIDMLPTTSKPVLDFAFMEYHNGV
KDPQLTDELLSLLIDNVKLFVIAGHDTTSSAIAHVLQLLSKHPKAMEKVIAELDEVLGPADQTANRILSDPTVLNRLPYL
TATIRETLRIYTLASTVRAAPPGATILGKDGVHYPTDPEMMIWPFPMMILNKDTYGPTTHDFIPERFLDMSTIPKDAYRP
FSKSPRVCIGQELAMIELKVVVAMTVRRFRFKNGYPEFHKREKGDIEWLNRFVPFEQGGVPEAYGTLSTSAKPHGGVPLI
VEEIGK*
>CYP51F1|419767|Ascim1
MGVLAGVADQLPPLTPLTFVGYAAAFIVTSVVVNVLQQLLFKDPSKPPVVFHYFPFFGSTVDYGMDPYAFFFKNQKKYGD
VFTFILLGNKMTVALGPKGNDFILNGKLSEVNAEEAYTGLTTPVFGEGVVYDVPNHVLMEQKKFIKVGLTVEAFRSYVPL
IVEQVTNYFNSHKDFAGKSGSVSLLKVIPEITVFTASRTLQGKEVRDALDGSFADLYHDLDNGFTPVNFMFPWFPFPHNF
ARDRAHKKIARTYMDLVEKRRANPDSKSENDMIWNLMNKTYKDGRPLTDKEIAHIMIALLMAGQHTSMSTSCWAILHLAE
QPDLVEALYQEQKKVFGDNLEPLSYEKLADCTLMNFVIKETLRIHPPLHSIMRKVKSPMRFPDTNIVVPEGHYVLAAPGC
SAIDAQYFPNPLKFDPYRWEGRAPEEEGEKVDFGFGVISKGTASPYLPFGAGRHRCVGEQFANVQLGTILATFVREFTLR
LPNGQVSVPPPDYNSMIASPTAPAAVEWVRRK*
>CYP51047A1|419945|Ascim1
MAVPSPLDPIDPLGTNTSFKQLLGTIIRHAFTLFFGYLTFDCIYNLFLHPLRSFPGPVMARASNLYSFFYALSGHQEEAE
YALHILYGPFVRARPNQILIADPAYIPTVYHLRADKDSMHMGEALGLHTSTLAPGPWEQHAMRRRRIAGAYSMTNIKRME
PLIDARIMEFTAALDERFVKTGETFDFAVWANYYTYDVVSTIAFGEPFGFVRTGTDVASLLKQFHGGAGYATSIAKIPAF
EAALGMIPGFSKLWIPKPTDKKGLGALMGFRDKLLDARMAEWKEGKGRDRKDLLSHFLAAQNLDGTPMTREEIAGEALLI
MIAGSDTTATTLRHLLFNLLKKPEAFEKLRNQIDKAYDTGKLSKPVPTYAQVEKEVPFLWCCLQETLRIAAPVAAPMGRV
VSEPIAAGNLVIPVGAEVGMNPWVVHRHEETWGEDCEEFRPERWEVRESERREMEKKMLTFGGGARVCLGKGIALMEVSK

VIVELLRKMEWEIAEAPKGEEAEVRWNTGTWQERGYWVRAERRDLGR*

>CYP6001C32|72118|Ascim1

MERAIQLSARLPALSKNRLKLTAQQVDQLWDSLQHPPLSYLGSEFNYRKPDGSCNNILYPHLGKAGTPYARSIKPLTPQP

GALPDAGLIFDAVMARRQYKKHPNNVSSVLYYVASIIIHDLFRTNRRDLNISDTSSYLDLSPLYGCNEAEQKTIRTFKDG

RLKPDAFAEKRLLGFPPGVSVLLITFNRFHNYVVENLAQINEKDRFGLKFARGERRDDPEAIKRAEAKRDEHLFQTGRLV

TCGLYINFILNDYLRTIVNLNRVDTTWTLDPRFDPARVWNPDGTPAGVGNQVSVEFNLVYRWHSCISDRDEKWTESFMRD

VFPDVEDTSKLSLPQFIGGVMKWESQIPEDPVERTFANLQRRPDGKFEDGDLVDILCDSIEDCAGASGARNIPIAMRMIE

ILGIEQARSWQCATLNEFREFFGLKAYNEFEEINSDPEVADALRQLYDHPDFVELYPGITAEEAKTPMEPGVGIAPTYTI

SRAILSDAVTLVRGDRFYTVDYTAANLTNWGFAEVATDPNIECGCIAYKLLIKAFPNHFKYNSVYAHYPMTIPTENHKIL

TKLGWVNRYNFERPTPIAPRINILSYNALQRVLNEQHNFTLTWGEAHDFIFKAETMLSRDNSINAQMKEFVGGCIYGQAA

WKEQVLKFYEEKTLEMLRKKSYFIGGNYMVDAIRDIGNMVHTHFAAELFSLPLKTKDNPKGIYTEQELYGVLSAMFVAIF

FDIDPSKSFPLRHMAYKVTEQLIKVMTMKVKAIKSWSFLRNKFAKHQPSELKDYGVKLIERFLQSKKSPEEITRSFIIPT

AGASVPNQAQVFSQVLDFYLEPQNAVHLAEIQRLARLDTVDSFDAIQRYALEGTRLAGTFGLYRKVEVDNINLNDGDKEY

QLNKGDVIFMSFISASRDPVIFPDPLEIRLDRPLDAYIQYGVGPHKCLGTDINMLSMTMMLKIFAKLPGLRRAPGEQGKL

KFVSRPGGFKVYLKEDWSGMWPFPTTMKVQFDELI*

>CYP52AR1|210595|Ascim1

MSNYLLFLAFIFSYGVYRLITTTIESRKLANLAEERGCKPEKRFDSGFLGLSRFRLVMKHAKAGTVIQFITARFQEKKTW

SVNLFGKRVLGTCESENIKAMLAMQFEEFALEYRKVDFNPLLGDGIFNSDGKVWHHSRALLRPQFSRTQISDLDALEVHV

QRFMKLIPTDGTEIDIQDLIYRLTIDSATEFLFGKCTEALLSDDGNASLKYGDYSFARAFSEAQEWCIWRLRLGVLSPIA

WLIPSYSASNKAVHKIMDRLIASCLAEMKHNPQSLAEKEYNPTDGRYIFLHALAHDTQDPRFLKDQLLNILLAGRDTTAV

LITWTLFSLARNPDIYRKIRKEILTHFGTGTEKISFESLKNCTYLRYTLQETLRLFPPVPINSRLAIRNTTLPRGGGPDE

SAPVIVPKGQLIHYYPYELHRRKEVFGPDAEEFLPDRWDPSHPSYRKIGGWDYVPFNGGPRICLGQQFALTEAGYVVARL

VQRIGELGMEDKREPVFAPNMTMPSARPVMMSFREA*

>CYP6958A2|412268|Ascim1

MILQVPLSTLSPQLPNVEVPFDIKTVSLIYIPAVLLTLIIGKIIYLRHFHPLARFPGPFLGSITNFYQAYIIATGKLELY

ELEWHRKYGPVVRIRPNYLSSSDPRHIPLIYHKDADKTNFQDLPSFGFELAIASQRDHKHHAFLKKRIAPAFSLKVVKES

EGLVDLQITTLLKELDRRYLSGTCDGKNDGVLDIAPWTQYLAYDVITELAFDDNKGFLKANKDVDGLIGELLKALKGGII

ISAVADLFLEIYNEPLGIGRRLLNTKQDPNSGLGRILSFAQAQIDQRKRRIREGKPVRNDLVNHIMNLRDPNPDYDSKEP

GPLVSDAYVRSELTVITFAGSDTTAANIRTIVLEVLRNPSIKAKLIAEIDSCYERGTLTGKIASYDQLNAKDNCPYLYAV

LEESRRFRAPAPIILPRKVAAPGMQLPIGNYGDNVYIPAGADIGCNSMVTTRDTNFYGPDAEVFKPERWLESLVGEKRVI

ALKRADITFGAGSRTCLGKNIAWMELVKTISSLFRLYELDLVPPPPGEKPYHFENYSLWHESGIWIKIKRRNVPEWFELD

EATI*

>CYP539A36|413555|Ascim1

MLADLLPPFPVLVALGLVSTVFAAIFNHIQTNNRLKRLGVRARKRDDYNPLFLGADVVWNGVQSGKVNKTIDNWFDGFKT

NSTGTAEFTILGYFRVLMTMDPENIKAILATQFNDFGKGERFHHQWKEFLGDSIFATDGQMWHNSRQLIRPQFLKERIAD

LHTFERHIQEMFKYLPVDGSPVDIADFFYRYTLDNATDFLLGHSVGSLHNPKARFADAFQYIQRFQNNIARSGPFWRLWN

RKKYNEEIQVLNDFVNPFVETTLQMKPEDLKPKTDTEYNFLHALAEFTTDRTILRDQLVAVLLAARDTTAATMSWLFHEI

STRPDVVKKIRAEILEIVGPTDTPTYAQLKDCKYLMHVINETLRLYPAVPFNVRTALKDTVLPRGGGDNGMEPIAILKGT

DVSYSTLCMQRREDIFGPDVNEFKPERWETWTPKSWQFIPFNGGPRICIGQQFAYTEIQYTLVRLFQKVESVEERMGGRK

QELKAEVVITPAYPVMVALNPAKSQ*

>CYP52AN1|417670|Ascim1

MIDLSALFDSPTHAALVFITITAIYHITSSIFTTLRHRRASHTPPCSPPFLLPESGWFGIGGYRWIKATFRSGRHLPVFF

ERCQKYGVDTIQLQVLHNTSTHTMAPENVKAILATQFEDFSLAVARKRFFDPVFGDGIFNSEGETWKHSRGLLRPQFARG
NIADLEEIGECVESFIKRIYEDGKPDASNGVEIDLQPLLYMLTLDSATGFLFGASVDSLKRRDLFEKKEDDYTFAEAFDK
SLLYTRDQVRAGPLRLDWFIRQKADFKKCVQGVHSFVDSHIRNSLNRVSQREKDGLHTENPKFLESLASETSDPIVIRDQ
LTNLLLAGRDTTAAMLEFSLFELARHPDIYAKLRSEVLKHFGRDPKKLTYEALKDCHYLQWIMNETLRLHPPVPINGRIA
IRDTTLPTGGGPTGTQPFFVPKGGRVTYSVWSMHRRPDLFGPDAASYIPERWDPKEVSEGRRKKVPSWAYVPFNGGPRIC
LGQQYALTEGGYVLARLAMEFEKVAAWTGEEEEEVEMAVSLTAKSARGCRVRLWRDPVGVEGH*

>CYP51085A1|419008|Ascim1

MDSELPSINFPYLLCFLVACPFLVHFLRLHASRQYAAPIGYQSPFYFPSGRLPFGISFLLLTIKGFKTHTHLPLMHSRFL
VAKRKTYAFTILGTTAITTMDAENFREILGSGFENWGFGELRRGNFGSSFGETGILVVDGDAWSALRGVLKCVFRMRREV
VMEMMENNWEKAWKRLAAKIDLEGEKVVDLQPLFFRASLEISLWFLYGVDVDSLCMSEEELCRFEWAFNGVQIHLAKRSR
FGKFYWLGGGKEFGEMIGVIHKFADKVIRQCLDREENKDNFLLSLPPSERRYHVLSLLLAARDSTAVTLSFLLTHLAHNL
AIFSTLQTQIRDLYGTTLSSFLSVIRKKGPDYIESHPQLLNHCINETLRVHPPVPLNQRFAARDCTLPHGGGSDGTSPIF
IEKGQRVDLNFWALHHDSDVWGSDSDVWKPERWAEMEKGERRKPEMWQFAPFSGGKRNCMGRGMAMKEIAFLVVRFCQEV
ERVEAEEEERNRYESEGVRAGSYLAMYVAGGAKVRCWGRGRQKLA*

Ascodesmis nigricans CBS 389.68 v1.0

>CYP51076A1|312548|Ascni1

MDSLIYHPTLQHLLSLSWVELLIHLAILFTTYTLLEILYNLYLHPLRHVPGPRWAAASPAWAIYWTIYRPGQLSYELERA
HLKYGPVLRIEPNRLSFSSASAYTTIYTHRTSPQTFTKDPHLYSRLGDTDALGFYLDHRLARTRREFLNPDFTRRRILLS
QGVVAGHVEHLVARLLEESQNRYKDQDGERVGDGDGEEVDIYRAFRSLTLDTISRLVLGRELGALDAPGFSHELVTGMEL
VTLSIWWRFRSRVVCVLMNMLAQVVVKWAQINLFGQVWFNRRCEEAYERTVEKVRAGVVEGGVIPRMVQENWGKRSVVAE
CADLIFAGSDTVGNALTTGFTHLLRNPDALFTLLAELKTIWPDPSSPLPAVEALEKLPYLQAVIKEFLRLSHGVTTPLFR
VTAVETVVDGWRLPKGTYLGCANPVLHLDPSVYKDPHRFDPERWLNADSEELARMGANLVAFSRGPRSCLGMQ*

>CYP51044A2|51757|Ascni1

MCDNQTDNLDPTVFRDKGFTSAALEDYAPRVQYHVHRLMEVIKSSKGKEVRIDLRTDEFAHDVVSDLGFGVEKGLQEGYG
NREYFESINGLLALIQPIGSLRMLGEFFSVLPLLKGSNRLNSTGEALFEARRSVGLSRKDIFSHLMSADHRTAPPHLVAF
HGNELSSNAQVLLIAGTITTSTTISMALYELSRNKGLQTRLYQELLEAFPNSPYDEITVDVVKGLPYLNAVINETLRLHP
PAPNGTQYIVPDDGRTIDGTWLPGGTVVRTCPFAIQRDERYFAQAEVFFPERWTTRPELIKNRNAFIPFATGPYICVGRN
LALQEIRLALANIIRVNRVSLGLSFNNKVFWDEWKDRFVLRIGPLPLVFTSRID*

>CYP51F1|321934|Ascni1

MGVLSTLAQPLQPVLLELSALPTPLYVLILGSAILVFAVVANVLQQILLRDPKKPPVVFHYFPWIGSTVAYGMDPYRFFG
DNQKKYGDVFTYVMLGRNMTVCLGPHGNEMVFNAKLSEVSAEEVYTNLTTPVFGEGVVYDCPNHRLMEQKKFMKFGLTTE
TFRSYVPLIVEQVEDYIKKSPFFKGPKGTASLSKIIPEITIFTASRTLQGKEVRDALNGSFAADLHDLDMGFNPMNFLMP
GIPTPGNRRRDIAQRKMARFYMDVIQRRREDPEQSKDKSDMVWNLMDRKYKDGTPVTDRDVAHMMIALLMAGQHTSMATT
MWIILHLAHQPKLLEQLWQEQLSVLGNPPRPLAYEDLSRLPLHNNVIKEVLRLHPPLHSIMRKVKSPLVVRGTPYTIPST
HYVLAAPGASARDSRYFKNPSIFDPSRWETQTNGAADENEEMHDFGFGMISKGTQSPYLPFGAGRHRCIGEQFANVQLGT
ILATWVRLFEMELPGEFPEVDYTSMIAMAKAPAEVVWRKRDY*

>CYP540A14|323378|Ascni1

MAVKQFYLVGSSPDTARDIDITPSADFAALQKQLALEFNIVEPTGLCIGLQTDDSGALTTLDSITATITPIGITIDGQPI
REPTGPDGFPLVGSFYEIFPDHLGNHARLFRKYGSVIKTTNMGKTTYLTEDPRVAAHALTESQYFSKRINKNHPLWGIKD

NNAIFINDTETEQWRQTHKFIPPVMSPKAVRHYTPLMEHTVRESFKVFDALDDRGETWNAYQYMLKLASQTIGKFALDLD
LGQFETPESPLHPIVTNIAALLHLNKAITARGEWYRYLPFGDPKKLRQVQQTTYSILQECIDRCLARQTDARDIPLHEAA
LKATCVVDALVRGVDDQGNRLPRELLLASMVPVTGAGFTTTSALLSWCLYALCTYPGVQDRLLQDLVDNGINNDITWDPD
RPNTLHYLDNFIKETQRLHNPSYQPGRTAKKEVIVPGGYKLPAESVIIPAIRAIHVNPDVWSNPQRFEPERWDTEEVKNR
HRCAYIPFAAGQRGCIGFNFALQEVKVLLAELVYRYEFEREGTDAIEYDPEFQLIRPLNLYIRARRRTQWPEKS
>CYP51044A1|326192|Ascni1
MIDDGIFLLTLTVLVIIQRLFFHPLRCFPGEKLAAVSLIHEAWYNTIGKLPLHAQELYKKYGDFVRTGPNQVSVNNVDAL
NPKHGIYERGPMYEIATLFGAAGLSTMRDRARHKIWRKIWDKGFTSAALEDYIPRVQYHVDRLMDVIQNSKEKEIRIDLR
TDEFAHDVISDLGFGVEKGLQEGHGNREYFESIKLPLALVQPIGSLRMLGEFFSVFPLLKGNNPLNSAGEALFEARRSMG
LSRKDIFSHLMSADYRTAPPHLVAFHGNELSSNAQVLLLAGTITISTTISMALYELSRNKDLQTRLYQELLETFPNSPYD
EITVNVVKRLPYLNAVINETLRLHPPTPNGTQYIVPDDGRTIDGSWLPGGTVMRTCPFAIQRDERYFSQAEAFFPERWTT
RPELIKDRNAFIPFATGPYVCVGKNLALQEIRLALANIIRVYRVSLGPSFNNKVFWDEWKDRFVLRIGPSPLVFTSRID*
>CYP51077A1|334956|Ascni1
MNQTNLTFPAILPSLSTIVLSLPILLLATWLANAIYNIFFHPLSHIPGPFWGKITNFQFILAYIRGRDVEWDRRNFESYG
DVYRTAPNLLVFSNPAYLPLVYHRHAEKGDTYLITAPYGLIALSGHREHAAARKRLQAPFTVSAVRKTAALVNEVVDAWV
TGLGEAVDELHINPCSSSSDDGRKKGKTVELDFTIWPSFLTYDVLTKLCFGKEYGFCRERRDLGGLVKAGEDNIVPVGVA
MRLPVVMRWVRRLGLERLLEVTEETPGMGEFIKYRDRMLKERLEKPSDTPDILNHLLKTWNPNGAPLSETDITQLKEELL
GLMTAGTDTSAQFLRDLIYQIGTHPDSLSRLYSEIPPTPSSSASPPPPSPYLTACILETLRLHAPATFPFPRTISSPGLH
LGPHFIPPCGSHIEIYASALNIGRSREVFGEDAEEWRPERWVEAGEEVRSRWEKASFAWGFSARGCVGRPVAEVEIEGAV
RGLLGAFVVEGARVVGGGEVGEMGRLHVGRRGERVLVELRRRE*
>CYP51057A1|337094|Ascni1
MIILTLSTFFILYVLHSLFRLHRNLQVARAIGLPILLHPYYEDPFIFALSMSPVGTYIFSNLFPLLDRIFSPADTTPITD
RMNFSTTVRRQMVQNRIHEKYGQVLILVGPNGIGLQVADEEAAKELLGIKVPEGTHGQGARFPKNAYHYRALALYGANVI
STEGKEWARHRRFTAGPFNERNSSLVWRDSLYQAMGMVEKWQQPPNEPSASAIIQAPEFQRHIRALALHVISASGFGVNL
CFTPKQDSSSTKVSEEDSFFLDDAPPPGFRRTYRHALEYAIHHLINLIFVSQFPDWILRRSWGFFEDVYVCREEVRTYLN
TLIGNERRKIEQQNSDSAHEVDNRRNNLLSLLVRNEEHHEPQTATETKHEAKSKLQPFNNQEILGNAFLFSLAGHETTAG
TLQFALAMLALRPAAQDWLHKQLDSMFVTAANVFGEEFDVEDPATWPYELYSRLKGPLAIMYETLRLFPPAGNIPRWTAS
NPQTLTYKNKTHHLPAYTDVTLNGFGLHFNESYWGKDVKDFVPSRWDPDNLDSYLYRYENLNPPPNTNANNDPETTASKE
PNIL*
>CYP51057A1|339012|Ascni1
MLSQFNFKDNLIPYWQDVALLLFVGYLSRVAYLGFYNLFLHPLRKYPGPKLAAITRGYAFYWNVTRDGELYEQLLRLHAR
YGSVVRIEPNKLHFSTPSAYNTIYTASTNTIQKDPDFYNALSVTPGLGFETDPKAARRRREFISHDFSRRNVLSMEKLIT
DRANQLIAALTCVSESETGSPSSSSHLLKKSNLYRAFRSITLDLTADFGLGTSMGAIETPGFSHPLLLVMESSIEPIWWK
VHDPVASFLLGEFLLWVVQTLGLEIGGFGDIIGRVMKLYDEAFSKKSERAEKRGAGVREEGVQLDEAIIPRMVDAGWSQK
EVLGIISDLVFAGTDTVGNTLLFAATHLLLNPKFDRRLLEELSTAWPDITEPPPPIQVLEKLPFLQAVVRESLRVAPGVL
SPLWRLTKKNVTIDGFHIPAGTTVASSAPQIHFNPDIFENPHEFDPTRWLDLPPAELSKRLSCLVSFSKGSRSCVGINLG
YVELTLILAMVWRKCEMKLDEGFSMEQLRAKDHFVAIYPGTHVHAWVRRRTD*
>CYP548BE3|339759|Ascni1
MGSHTLMVEVPHALGTISLSALRNGGILLAIYLVTLVVYRLYFHPLAQHPGPFWAKITDWYSVYHAYKGDRHLVIARAHE
KYGPVIRFAPNGLSFNSATALKAIYGHSAVARAVQKGKFYLAFPAVPGVHNTHNAISKLEHGRKRRVLSAAFSDAALKSM
EPLVLQNVDELMRVVEEQGLKGSGGIDMGEAFSWFTFDVMGELCFGRSFAMLSEPAQRFVTGLIDKAAHMHYIAGNYLPI
KTLGIGRILFPTIVADRWRFIEHSRACANDRMKAGHSAKKDFFYYLLHAKDPETGEGFSTKELWGESNVLMIAGSDTTAT

ALSSTLFHVTKNPAVYEKLQKEVRERFNSVDEIVTGPELGGLPYLKACIDEAMRLAPPVPGILPREVIAKEGCTVDGVHV
PFGTVIGCPIYALHHNDKYYPDPYTYNPERWLGDEKDVEAARSAFTPFSIGARGCIGKSVAYMELRLAIARLMFLYDIEQ
KKVEGKADMWKEGFATKEGEYRLKDHFTSRKEGPICEFSPRKLTA*

>CYP52AT1|341881|Ascni1
MTALTDTLREKVSSVPCGDFTPLLLIPLLLILTILYHRTRNAHVPRYPDPFGLMFIYGAKKAADRKDVPFWFASLFDRYG
ETFVIPSILGKTSYATMAPENIKTFLSTSFNSWALGKLREQAFEPLLGKGIFTQDGGEWSHSRKLLRPQFQRRELEGLGS
IHEHIGKMVELIPDGEVVDMQPLVYRLTLDTATEFLFGETAGSLDSTDKATNGQGFADAFNTAQSYIVWRFRVGKILNTL
FHSSEGEDAIRRCHSYVDQYIFRNTQKPLSQSGRYVFLDSLKSEETDPFKLRCQSLHVLLAGRDTTAGTLGWTFMLLAQH
PQVWKKLKLACEVLPKEPGFSELKDIPYLRHVIAEVLRLYPSVPSNGRVATQDTILPHPAPGIKVKKGEFVRYSPMAMQR
RKDIWGEDADEFRPERWEEEGLMQRVGSWGYLPFNGGPRVCLGQQFALMEASAVVQAVVRSFEGVEYCGGEMHVVEPALT
MAPAASVVRFWGKREVEK*

>CYP51089B1|342013|Ascni1
MSIPSGDQLAIPFLPVPISLPPKLTELITLASEDPIKYGGVFAIGSLVCWFLGLAIYRVYFHPLSRFPGPTAAKLTEFYR
FYWNYVRKGGLVFEVERLHAKYGPVIRISPNELHFSTLSAHSAIYPPSRPPFLKEPTFYSTLSTNNALFGRLNPTAHRIR
RDLLNPFFSKKSISALSTTSQLIPRTATTFLTRLTSFAASGEEFRIDLAFKCFTIDVISTYTFNRCFNTLQLANFDPLEV
SAILGAIRQATLAEFFPWAQWAVLKAPGGRAFARLARMELGMLMDALGKSYQEVVAYKERGETEGMMKRMLTPEEKGSGY
EVVDSDQVLAELGFELLGAGMEETGNALMFATWHVTTTEGVEERLVKELREWCPVVGEEAVGGVEKLPYLHGVWKEALRM
SHGIPGRLPRIAPPNGITIDNHFIPGGTIVSVSQYLIHRNTAVFPDPELFRPERWLSADSRSLEKHVIAFSRGPRQCIGM
NLAEAEFKILFANMMRRFSIKVELETGDKLEWRDFLTVFVKGKVRALAKEREE*

>CYP50183B1|344694|Ascni1
MLELQALLEHIGVLPLVLLILTVSALIYALRQLVFHPLADYPGPKLAALSPLPFVFSNIRGHSHDDLLNLHRKYGTHVRI
GPSIVSIATYKAQERVHGGNPHFPKGQHYKNWQLGVAKPGLTPATDVHDHAVQRAALNPMFFPQNLKKQERHIQEGLDLL
MEKLYQHKNDPDGVDINFWALAFSSDVITDLAFGETFGSLKAEKLHWVVEQMNKFFSMILVLDSFYRILPSWLWSYRESL
VPKKVTAQRQRLAEFAQTRLVERMNNTALREDFITALRDVPEEKRLNDAQLRMNCIGFIIAGGETTSTALTAAFYFLVTI
PRAYTALTTELRSAFTSPHEITATNVSALPYLQHCINETLRLFPPLATLGIRESPGADVDGVWVPHGVEVVTPQWVINRD
EGNFRRAEEFWPERWGNLKEGDGAHGGEDRKRACQPFGMGQRMCIGKALAMMEMRLVIARTLFEFDIELARKVDLVRESR
VLMVWNKAPVYLKLTPRYTGPQA*

>CYP51078A1|344712|Ascni1
MLSELLGISGVWHLKIHASTASPSIAGLMTLLAIIAAWIVHFFNISRRRQQKSYDQEPPTLPYWIPWLGHAVHFLRNSNG
IIDRAIAYFGNNRPFTIYAGGERLYILTNPKDVRQLYRKTRQLSFSPFVETIVLNVYHFDEHDFKTLTAVHPVTGATILG
ETHGFYRHHLLPGERLNTLKARYIQELEHSMENITFPSEGVNLFPWVCDTIGSTSTSSVFGRGLLERNPGLLKDLWVFDE
LHLWMISGMPRYFTQPGFDARDRLLTAVEEYFDQGYAKKEERLDMVWMREEMISKTGLSRRARAALHLTIYWAMQTNSVR
AAYWTLSYILRTPSLLNKIRAEIAPAFSTSSPTKLINPDLIITDCPLFNATFNEALRLTSGSASSRVVAEDTEVGGYILK
VGGKVMAPSIQPHMDASMWGDDVEEFRPERFLNKDGQWDGEVERKMGYGFRPFGGGETFCPGRHFARFQDLTFVASVIHR
WEARVPEKTTWPTELRNRSAIGVLDPSHIGPRVNFESRLNPEPSS*

>CYP5093G1|355103|Ascni1
MWSGPRPTVVIGDPQVAHDLLHLKPIIFGSRPRFVVMGELFAKNGLLLTMPYGPKFTATKRMMNMSLVARDLNAHAVQEA
ESKKLARDLINSGYSFERHINRYVASVMMCLAYGRRIDNMDDPVLEKIYQRARYMAQLNTAGRYWAETFPLLTWIPDFLA
PWKREIKRNGAASSQMLYDLAKEAEEPIGYDRAAPKSFAKNLWEKKKHEPALNLNEWEIANAAGSLFNAGSDTSSATLQT
FILAMTVFPEVAAKAQEEIDRVVGHDRVPTWEDRQNLPYCRAILKETLRWRPVAVLGGNPHCSTEDYHYRGHYIPKGTAV
MGNLWAIHHNEEYFVDSHRFWPERYTEPEKVQAFCKPYPNPEGHSSFGWGVRACPGKGLAEDSLFISIVRILWGFNITKA
RDPATKEEITPSTFSYTTGFNTKPTPFPCVFTPRSDKHREILLQDASAADEFLKGYRTG*

>CYP6136B3|355547|Ascni1

MITDLLHDIFDTIVANPFLILAIFLGYSIGSRLLRYYTSPLRRAGLPGPLLGHFTSWYRAYHVLIHRNWHHKLQSLHVHY

GPIVWIAPDEISVSDPKLRSTIYGFADERKEQSFFPKAHVFETGQINEDFNFVFETDPKKARVGKWAMAHAYSERGLAVL

EEEFDKAVKDLVDGLATHHARPNRSANLGKWCHYFMYDLCSLLCSGYTSGLCQAGRDKDSQIYSMRVIFEAVGGLIPVNF

TLKVTTRWFRKMLLNSYLEHFFKGCLLNDEYYSKEERINKISDDKPQHLMSRFRTAEQKIRKLFPIGNWTEAITNNIFFI

YAGSTVASNAFPRVLQLIYSHPRVLRKIRAEIASLPNQTVTISSLDRTSGHTCQIPYLEAAILEALRLTPTFGLSLSRTV

PPMGCKLQDYTIPPGYIVGMSGWTVNYDNAYFGADAAEFKPERWLGNHPTEIGRDGTGLPRTMRNYLEAGWFAFGAGARV

CVGRHMTMIAFTKGVAEMVKRFEVEVEREGKDWYALIVHTEGLVVRLKERWGVESEGERELLGVCKEFMGVGA*

>CYP676B1|356436|Ascni1

MTGGKWTFVRISHKEVSIDEPAAISPWYEIFSLPDSSYPNQMSECDHIRHMHKTKNLSGGFTLTNLLRPEARIDSCISLL

CSRLATLSTTQRTIPLYKWLHYLGFDVLGEMTFSESFGFLQEGRDIRHAIRITDELATYVAVMGYLPWVHKFLLGNPIIG

LLGLRPNQHILDTAVSVQEARREKWDARLNILEQWNWRYHAVKGKGGMEKREIVGAALANIGAGVGAVNTVMQGLFYFLI

RNKEWMEKVREEVDQAHREGRLSWPAVQYREAVGLRVVDACYKETLRLFPTIAFALLPIVPTKTTVSVNFWTLHRNTSIF

GADAESYNPASWLTGTPETLRNREKFLMPFGVGYNSCPGQNVAVIEVLKTVVTLLRRFEFDQREPEKKENQWK*

>CYP596V1|356731|Ascni1

MIWEGVKKFQGKPFQVIGVNGPDLMFPGRYAKMVGGDKRFSFDEFIEKDFFPSYPGFEGPKASIVHANIMVTTIRTKLTQ

SLGHVTGDLCDETEKALHDVFGENPDWQECAFNKLSAPLVARLSTRVFLGPILAANQRWLEIAVSYTINMMITSRLLRQI

PHALRPFVYRFVPGWRALRKDAIDGRKIISAEIERRRSIWEENKKLGISTAKTADSLGWFQEVAGEQQFDQAGCQLSLTF

AAIHTTGQMLSKALFRLAENVEWQKEVREEMVRVLRTDGWQKTSLYKMKLLDSFLKEVHRFDPGSTTSMHRVTKESVALP

DGTVVPKGAQIKVLQDLHMDPVVYSNPEVFDPARYLKMRSQPGQENNWQWITVTEHSLGFGYGKHACPGRFFASNEVKIA

LCWMLIKYDWELAEPLKPKWWLGSEMIPDAAAKLRYRRREEEIDLSTL*

>CYP51063A1|374018|Ascni1

MVLVDKLTSFAIILLGVVLNLSVGEVMCAFLTALLTFFLLHITHNLFFHPLSHIPGPRLAAATHLYLTYHHFHGRLPFVL

KLLHSHYGPVLRIAPNTLNFSSGTSLADIYGSSPHRKFFRKAPFYTNFDAGGNTNLNTETDVLKHRELKRLLGPSLSHRS

VMGVEATVRRYVEVLVGGCGRLGAEEEGVEVTEVYTEFAFDLVADLVFGRRFESLETGKRHFWIKMLLENVRFMILLNAL

NKLFGISLRRIAATLPKFLLRDYYKTINFTTEVTQERITALSTPSPTPPRADLLTALLPSLLTASFPLPRVADNVQALVI

GGTDTLSLFFSACTYFLCQYPSTQSRLLAELRAEFKSAVEITGERLEGEGCRYLNAVIRETMRVLPPVALPLPRVSPGET

VDGVWVPEGTEVSTSFYAAATDDGHFKDPMRFEPLRWLDPETTDMLEASQPLLLGPRHLTYLEMRLAIARLVWEYEMQLV

DRQRDWVNENRLYAFWLKAELRVRFKRRGAVGV*

>CYP567W6|375597|Ascni1

MGVIGDLEFSRVQNLPTWVFFAIPIAFLILRTLLTGIYNLYFHPLSHIPGPKRALFIPGYYTILLLSGRVHITVARLHNL

YGPHLRLGPSTISFSSPTSWKHIYGHIGGRKTFLKSPMYDQGGDPHVRDIVSERDPAKHGPLRRLTSHAFSAKALTEQED

VVQAYVDKLVCQINVHATKQPEEMVKWYNFATFDVIGDLAFGQPFGSLDNGEPHFWVSMILDSVTAAAWKMVAVKIAGTG

RLSKYLMPRGLREKRERHYEYSRKTVERRINSPTTRKDFMSKILSEKDAKGYDLTFLTHHSSVLIVAGSETTATFLSGVT

YYLCRTPHAYAKLTQELRSAFSSYEHITGMETERCSYLKAVIDEGLRIYPPVSFGLPRTSPGEIVDGVFLPEGTECFTSS

WAATHSELNFHRPYDFLPERWLDPDCTDQKEASQPFSLGSRVCLGRNLALMEMRLILAKMLWVYDMELVDDVDKLDWVRD

GTANPLWRKPPLMVRFTRREGVRVGVLDDEA*

>CYP51081A1|394760|Ascni1

MALNLPDAVTNHPYLFAVFTAVTGYAISCIFGLARNILIARSTGLPYLIYPIHERNMIYGIITAIPAVQKWVDTWKNEEW

RDWWNAGPYPTKWKARKAYARWGTMYLRVAPGGIVLECADAKAIDEIMSHRERFPKPWWTYTMLMMYGPNIVVSEGQEWL

RHRRVANPVFNERSNKMVWEVSVEQAHDILAHWKLKSDNPPTAVNTMAHDFMSVALHVISAIGFGIPMFFSEDKTATLPR

PPFTNRQPDPGYDFTFAQALNFISFNITIHAAMMGGMLPVWFPKSLLAVYQKHKAIHHDFSRYLTKMIGDAQEAMKDSTE

IRATAAKEYMSHNLLSVMVANSVSQAAESNGKNKPLSPEELKGDVFVFMLAGHETTAQALNYAFLAMAVEEEAQDWIAEV

IHEETKDLPADSRNWTYEEVYEKLNAAKYLMNETLRYFCVVSVVPKHTSFSGCTITPSSTVSPGPIHISEPMNINLVASG

AHFDPRSWGSDAATFNPKRWDNDFQPPKGAFIPFSGGVRACLGKKFSTVEFTAIATIILKDWKISVKQKEGETKEQAKRR

VWKLLEASHAEITNNVGEEVEVVFERRK*

>CYP61A1|135684|Ascni1

MNAPPASSGFQSPSAVHSAEMPFQTLINGMSGTIGKSLEGVSTWQVLLALVLVTVTYDQFKYIYNKGSIAGDKFKIPFMG

PFMQSVNPKFEEYSAKWASGPLSCVSVFHKFVVIASTRDLARKVFNSPAYVSPCVVDVAKKILRPENWVFLDGRKHVEYR

KGLNNLFNRKALAMYLPGQEAIYDTYFKQFLEYTKDGKPRQYMGAFRDINCAVSLRTFCGHWISDEAVADISENYYKITA

ALELVNFPIIIPFTKTWYGKKCADFVLGTFADCARKSRVAMAAGQEPTCTMDQWIKSMIDSKAEVEGVEGKKSQIRDFSD

HEISMTIFTFLFASQDASSSASTWQFQILANRPDVLEKVREEQLRVRGGDPYKRLDLDMIDQMVYTRAVVKEQLRYRPPV

IMVPYECKKSFNVTPEYRVPKGAMIIPTTYPALHDPEVYVDPESFNPDRWLDGGEAEAAHKNWLVFGTGPHVCLGQHYAI

MNFMAMIGKASLFLDWEHHPTEKSEEIKVFATIFPQDDCYLTFTERLPLEKAAN*

>CYP51068A1|162579|Ascni1

MGILKLSGYTVPELIGGAVVLSVLYALTLAVYRLFFHPLAKIPGAPLSKITYNREVYYAFFRNGRWPQQYSKDHEKYGPI

IRLNPDEVHISDPEFYNSIYYMGTKFTKPGFFYGAWGIKSIMTAQSNSVHRFLRAPLEGLFARPSILAMNPMLQEKVDYL

VDRATKVSNTSNGKLNIHAVTRAFTVDVIMEMCIGKGLDMLRREDLGKPYLDAVTGKSVLLWVADMYLSRWLKVDAPISR

TVRLIPRKWIKKMEPTDIGFFYLYLMGEDEVTRLASLSPEELEKETYGPGKRTNIFKELLKPGIREKRNLSLEYMVDESF

FILAAGLETTACTMTKTIYNILRNPDVHEKLFQELKEAFPDINTPMPYAQLDCLPYFSACIKEGLRCGDAVPMRLPRVVP

EGGIKYKDYYLPAGTMVSMSAYMQHRDATVFPDPEKFDPSRWLGEEGLQRETYLVALSKGSRSCLGRQLAYAELYMAIAA

LFRRFEMKLVDVDDKDMVLQHIYIGSFHSNSQRPCKVTLVKRTE*

>CYP6001C31|308786| Ascni1

MTQKNSTPSKGGPVTKNPTRKEVDDVFSKFAGLIQASNRPIPNRYGDGRDHDATEEQQTGVWNDMKVLKKGGYLGETLKT

LWMHMSHARKGGPVDDKTMIMERMIQLSARLPPTSKLRVSLTTKQVGQLWNSLQHPPLSYCGDKFTYRQADGSYNNIQEP

HLGAAGSPYARTVKPMTKMPGAPPDAETLFDSIFSRGKNGEHYRESDNNISSMLFYTASIIIHDLFRTNRQDWNISDTSS

YLDLSPLYGHNQEMQNTVRTFKDGKLKPDCFAEKRLLAFPPGVSVFLIMFNRFHNHVAENLKLINENNRFGIKFDHAWIG

EDEATWRPKAIKKQDEDLFQTARLVTCGLYINYILNDYLRVIVNLNRVDTTWTLDPRFEASKVYNPDGTPAGIGNMVSVE

FNLVYRWHSCISKRDDAWTQEFYKGLFPDKDPDQLTLMEFVMGVKKWEATIPEDPAERTIENFQRTKTGHFNDDDLVGLL

KESIEDPAGCFGARNVPHVLKLVEVMGIEQTRKWKVASLNEFREFFGLKKHATFEDINPDPEIANTLRQLYDSPEYVELY

PGLVAEADKKPMVPGVGIGPTYTISRAILSDAVTLVRSDRFYTVDYTAGALTNWGIEEASSNPDILHGCVAYKLFLKAFP

NHFKYNSIYALYPMTIPSENKKIYDALGTSHEFSWDPPRREPGRITVQSYAAVTHILKNPQDFSIQWKPGFDFIMEAPFM

LSGDGPPYSNIREHVQGCFYTEGVDWKRQIREFYEDLTTQMIRNKAYRIHGTGCYQVDAVRDIGNMAQTHFAATIFNLPL

KTATNPKGIYTEQELYMILCAMFTAIFFDMDVSKSYPLRRAAYAATRQLGSIVELQVKALKSRSWLLKLWDPMNVLSRNQ

SALADYGHHMIRRLLETGESPSDVTWKYIIPTAGASAPNQGQIFAQVLDFYLQPENAVHLAEIQRLAQETGVDNFELIKK

YALEGARLAGTFGLYRRVERDSLHLSDGSYGDLNLRKGDMVFVSFISASRDPDTFPDPMEIKLDRPEDSYIQYGVGPHTC

LGKAANIVSLTTMLMMFGRLKGLRRAPGGQGVMKTLPKPGGFKVYMREDWSAVWPFPTSLKVRFDDII*

>CYP55A31|311948|Ascni1

MRGSSPAIPSEIPQFPFKRPTGTEPPAEYAKMRAKCPVAKVQLFDGSQPFLVTKHKDICDVLTDNRLSKQRMRSGFPELS

PGGKLAAKNRPTFVDMDPPDHMHQRRMVEPFFTHEYVDSLRPQIQQTVDTQLDNMLKKGCAKPVDLVEKFALPVPTHTIY

SILGVPFEDLPYLTEQAAIRSNGSSTAAEASQANQQLLDYIGALVEKRLDIPEKDLISMLVTEYVQPGKLEKADAVQIAF

LLLVAGNATMVNMIALGVWTLDQHPDQLAKLKQDPSLVPKFVEELCRYHTASALATRRVAKETIQLHGQTIPAGTGLIAA

TQSGNRDADVFPNPDKFDMMRPRGRESALGFGYGEHRCVGERLARTELEIVFSTLYRKIPGLRVADGGMGDMGEGKDKAV

RFSPAGKDVGIAELRVVW*

>CYP52AS1|329010|Ascni1

MVHTIEPANIQAILATQFQDFELGELRRRQTMRLLGNGIFTADGKAWSWARALMRPQFARVQVEDMGVLEPHVQALFDAI

PEDDGELIELSELLYRLTLDTATEFLFGESINSQRTAMGEDGTKILSLGEKNSTFSPADDNKGKKQMGFSEAFNGALESI

QYRMRLGPLVWFVPPIIYQKQCNVVHTFTDEYVNLAYHRLNNKESTGRFVLGDALAAEIQDPIELRTQLLNILLAGRDTT

ASLLTFTFLLLARHTRVWNRLRLEIIAKFGTSTEDITFTRLKNLRYLQWVLNEALRLYPSVPVNFRMAVRDTTLPVGGGP

DGKSPFPVMEGQIIIYSVYALHRREEFWGKDALEFRPERWEGRKTGWEYLPFNGGPRICLGQQYALGEASYVVTRLLQRF

ERVECLPNEKIEKVMNVTLYAPKGARLKFFKAAN*

>CYP6001E4|331902|Ascni1

MVILDHIYDRLASHIDDEANADTEHYGSILKDLPPEELPMGGILVGLIKLAVSKEPLDDRMMLIEKAIGVVAGLPEKSKA

RKKVTGVLVDLLWNSLQHPPLTYVGDQYQFRQADGSHNNIMYPELGRAGTPYARTVRTEVKGNGAKPDPGLLFDLLMSRG

EEFETNPAGISSMLLYHASIITHDIFNSDRRDGNINHASSYLDLSPLYGSSQEAQNLVRLGQDGLLKPDTFSERRLLAFP

PGVNVMLVMYNRFHNYVAQTLAAINEGGRFTPPRHLRSEEALKWRDEQLFQVARLVTNGLYVNVSLHDYLRAIANVPATD

STWTLDPRVEVKKIFDKRGTPRGIGNHVSCEFNLLYRFHSAISDRDAKWTEEFYHSLRRTDTNKREDPSAKFFRGIMKYE

KSIPANPAERTFSGLQRGKDGKFNDEDLVKILKESIEDPAGRFGANHVPEIMKPVEILGILQARKWQVASLNEFRKFFDL

RPYKKFEDINPDPYVADTLKRLYGGDVDQVEMYPGMFLEATKPKMDPGMGLCAPYTVTRAVFSDAITLVRGDRFLTLDYT

PANLTNWGIVEVDSDLDFLGGAKMQHLIINAFPNHFKFNSVYAAQPFYTPAKNKEIFTKLGKADLFTFDPPTPLKPVILI

TSHASLKAVLADQKNFHVPWGAKMSSLETYMLASDRDESGQQRDLVSKLIYGDVPVHALQRFVSFSEALTLKLLEKESYP

LGRKGTRQVDIVAKIGNFAALHFASELCLLPLLSKSQPSYSEDELYKLLTDITLYVFSDADPTKSWTRRRNANKAAEELV

AKMEQVVQHVTPIKSNTACPVGDDLTSPLTPSTANATGPYQSKSRLGRAVFGRKGTEAPSNATEVTSPPQTPGKTGYQPK

TIIGRAIFGSSDDGYFASNTLRVGGNTMAGRLLGSGKSVREVAQILVGTASAFVANTACAFAQLIDFYLESENAEHWDKI

KALAAEGSPEADQTLMKYILEGFRLSNSLGLFRNVNPVDGDMVQIDQNGQTITMTKGDRIFLSFIAAGRDPTVFPSPNKI

RLDRPEDAYITFGYGPHECLGKDLNLMHTLAMLKVLAGLKNLRRTPGDEGKLKCITKPGGIKLYLTPDWSKLTPYPTTMK

LMWDE*

>CYP51084A1|362852|Ascni1

MAILATLNTLTSHLPPSVLLALPTQILLSSIHEIHTVSVLIIYTLTNTSLLLHQLILNTSPVSLLSTLLDLVKANILFLG

TLAVLTVIRRLYFHPLSRFPGQKRYAVSKLWEGYTNGLGENGIRVRDLHRRYNTPILRVGPNELTVVSVEALPHLYSRSI

PHKSRGPWYNIWKIQGSLNLLTASDEVHRQWRPLWEQAFSAPAMERFTKRVEEHVGRLVEAVGREVGKEFSVGEVIGRFA

FDVMADLGFAIPDYGLLSLTGDPGYMNIMHDFMCTSNVMGTFRNFMDVALYLPLERNPEVKKFRQYGRDLLKHRLSIENP

PKDVAKAFLESPGTRFTDIEIEANVQLLIVAGSDTTSTILTVLLYYLSKFPEFQETLYAEIQSATPFATASLGSLPYLNA

LINETLRLYPAVPSGAQAQTGPNGLTVEGQYIPPKTPVRVNQFAMHTDPRYWRRPDEFLPERWVDESLGLIKDRRAFIPF

SYGRHACVGKKLALQEIRLATAKLVEKYKFRPGENALGTEEYRKAWRDYFTAMPGPNWVKVELRE*

>CYP539A42|343016|Ascni1

MLADLILHSSPALAVSIGVALVLTLAFARIQQNRKIKALGGRAKVLPTKSLGINNVLTTVKYAKADRNRELWDERIAEYG

THTMEMEVLTKRIIMSTDPENIKAVLATQFHDYGKGENFHESWFEFLGDSIFTTDGKQWQESRALIRTQFMKDRIADLHT

FERHLQHMFSRLPMDGSPINISDWVFRFTLDTGTDFILGETVNSIANAKVEFSRAFADVQAHMNLVTRVGPFRHVLSKRK

FHKDLKILNAFVEPFVERTLAMSVEDLKGKKENEYNFLHALAQYTRDPRMLRDQLVAVLIAARDTTAGTLAWCFYELSKK

PQIVKKIRAEVLNKLGPDGIPTYSDLKEMKYVMNVINETLRLYPAVPFNIRVALKDTTLPRGNANNENEPVGMPAGTAFA

YSPLVMQRRADIFGEDVLEFSPERWEGRAPKSWQYIPFNGGPRICVGQQFALTQMQYFLARMFQRYETVENAMGPDHRQF

EKCEITITPGNDVKVFMKPAKYN*

Choiromyces venosus 120613-1 v1.0

>CYP50320A1|48499|Chove1

MSRPAALLAEFTFTKSSLFGILIGSVLSRAVYLRYLHPLANFPGPLLATIVDFDIRGLWMHFSGQGHVKNYEFFKKYGPV

VRMRANILIVNDPKAIPIVYYRTPEKLEASKLTEIFELLTRSEHAVIEKGTKSHSQMSATENLNFHLGTRLSKWMSVLDE

RFADTGVTFDYREWAHLLANDLVTDITPGKDTGASTQVDNAQGIQKSSQPTSAVKYILLLPRIVVDVFAWPLRDFTIPIM

GNATPVWKLSLRENGGVGIRHQAKISKEPNSLNKDAQSEDKGGSSISDGQTNVNPIRLVAAVSNTTDDVSIPLLQNVLGD

SRVYRNLLEDIDSSTAYPFFIPTLPSLRPNGPLSYLHACIKETIRYSITSRFNTRVVPNGGYEIYGRHIPGGARVLMNPW

VVSRDKAVYGEDADDFKPERWTRASSDQVRELEKCIIVWGYGDCMGGREGHRAGGDL*

>CYP663N1|1267439|Chove1

MAGENVSREMRIVLLQRGDKWRTHRRIQGQVINQNIANKYKGFQNLESTQLIKELVDRPEGFFDSFHRYNSSVIFAMAYG

KRMPRGDEEDVLAVDEITENFLYSARLGTWIVDSFPFLNYLPTFMAPWKRIGDNFYNWAEKMHTFNRNEALQRKGWNWTK

ETARMKEARNVSPLELAFMVGFLYEAGSDSTTIALEVFILALLKHPEVLKRAQEELDRVVGPDRLPTFDDKDNLPYVRNC

VDEVLRWRSPSAGGVPHVVEEDDEYMGYRIPKGAIVVGNLWSIHQDPEVYPNPTKFMPERWDDEENTHYGFGFGRRACPG

RHIASNSLFVNYARILWGFNVEHAKNPDGTEIPVDEWNMTQGFMSRPVRYKASITPRDAKRVEVINNAWKSAEGQLQDMS

KILRMEDF*

>CYP663L4|1288097|Chove1

MAGSYYTCKFCSLFNFPSSPFNKMCIAGNCTEDPGGGGGSGTSPPASIRPLTIIISFLAVVIASYLFQQKRRRLPLPPGP

RGVPIFGNLFQIARKYQWRQQQEWSQKYGPVFKMQLGTHTVIVLGTYQAARDLLDKRSKIYSDRPKFIVCAKHLSGGYRS

LLMNGEIWNTHHRLQATVLSSRMSQKYRPVQDLESKHLIHALLKKPEDFSLQFHRYSASLIFAMGYGKRLITGQEKELQS

IDAIMRNFTEAGAVGRWWVDMFPILDLLPKYFAEWKKISAEFHKFESELHVTNLNNARERKGWNWAKMYKNSPFSQGMSD

LEVGYDLGTLYEAGSDTTTIALEIFLVAMIKFPHVARRAQEELDRVVGKSRLPSWQDKDSLPYIEKVVQETLRWRPVAVN

AFYHAVTEDDEYLGYRIPKGAWVVGNSWGIHLDPEVYPNPNDFNPDRFDDETLGHVAFGFGRRACPGKYIAKNSLFINIS

RLLWSFDIGPKIRPDGTEVLVDDMAFTNGFLSQPLPFECSIKPRDQGRVEVIEREWAEANKDLLSLVAEERFT*

>CYP6220A2|292754|Chove1

MTDSKYFPNGIANKEYLTAAICAFATLVVWKVISGIFFSPLSHIPGPPVTAITPHYINFLSALNRRTVGTYALHKRYGPI

VRISPTEVSVLSPQAIKEVYSSPHYTKYTPLYSIFTHFGAQNAFTSCTREEHGWRRKAVSETYSLSFVLRDEAATGKVLK

VVKDYLSFVESDRRVDIYNANTFYATDVVTGKIFGLEASMKTLAGNEAHREIVLGHYSRTRRTQVWMYIEFPLIMNVFEW

FAFYTGRMWSWACGEEIEDWEMVSQIQKWGWDAYMDAKSNSRDGTVAGRLARLVQEGSNTEGVWNDRGAVSEVMDQMLAG

MDTTGDTLSFLMYQLSLPESRNVQKRLHSELLDAFPKSAEASSYGWPHTMDIPTLPHETILKVLNLPYLDAVLKETLRLY

TAIPITLPRVVPPSTTTDKISGHRNGRIVEGHFIPAGTTVGTLAYGIHRDEVFSLEPEQKGSPGGVDSFLPERWLINGGV

EGELTPDELAQEKQRIRTMEARLWAFGSGGRNCVGRHLSILEMKLLLVTIYSRYQTQVTPNSGVKITHNRWNERRTFRDV

LPFRGVDGIVTFTPYEN*

>CYP567W7|1357890|Chove1

MAVHLRVSDLLKLSPRELLGGLGALFVVCFVWRAIYNLYFHPLAKVPGPRICAINSFYTSYHWVSGRYPYVIRQLHEKYG

TAVRLAPNQVSFCSEGSWKEIYGHVGGRKQFLKSNFYTQQFPHIVNERDPIKHSEIRRLLSHGFSPRAVGEQEAYVHEYV

DRFIQKVNIYATKKDSPEEGEEMTKWYSFLTFDIIGDLAFGEPFGSLDDGRPHFWVSIILDNIKAIAWSTIFRQFLILRP

VAIWLTPKALMSKRIQHFEYSRDKIARRVQSKTTRKDFLSNMLDKKELKGITMDNLGANASILITAGSETTATFLSSTSF

YLCRTLSSYQKLVNEIRSSFNSYNEITGQATENLTYLKAVIDEGLRIFPPVPIGMPRESPGENVEGIYIPKGVEVFTSSY

SATHYSGNFHRPDSFIPERWIDPNCTDKKRASQPFLLGSRGCLGISLALLEMRVILAKMLWVYDMELMDPNLSWGKDTNC

YVLWDKLKLPIRYTRRAGIVVPPLDG*

>CYP50320A1|516044|Chove1

MSRPAALLAEFTFTKSSLFGILIGSVLSRAVYLRYLHPLANFPGPLLATIVDFDIRGLWMHFSGQGHVKNYEFFKKYGPV

VRMRANILIVNDPKAIPIVYYRTPEKLEASKLTEIFELLTRSEHAVIEKGTKSHSQMSATENLNFHLGTRLSKWMSVLDE

RFADTGVTFDYREWAHLLANDLVTDITPGKDTGASTQVDNAQGIQKSSQPTSAVKYILLLPRIVVDVFAWPLRDFTIPIM

GNATPVWKLSLRENGGVGIRHQAKISKEPNSLNKDAQSEDKGGSSISDGQTNVNPIRLVAAVSNTTDDVSIPLLQNVLGD

SRVYRNLLEDIDSSTAYPFFIPTLPSLRPNGPLSYLHACIKETIRYSITSRFNTRVVPNGGYEIYGRHIPGGARVLMNPW

VVSRDKAVYGEDADDFKPERWTRASSDQVRELEKCIIVWGYGDCMGGREGHRAGGDL*

>CYP50026B1|1661839|Chove1

MAISSLIPVIASVLTILRQNLVLILLVTGASAIIRRKYFHPLSKFPGPCKSLLSYLHGHTLLIAPVFAGITTLWRAYIML

TRRMHLLDMDLHRKYGPVFRDGPNSLSFSEPAAIEQIYGYRPRGEFEKSDWVLIFSENGKGVDYNTFSALYNEQHTKMRK

WVAGTYSMSRILDYESRFNENISRWIGRLAGFNGEPIEFFSWLHYLTLDIISDIVYGNPLGFVEKGFDIHNYRKDLHAAF

GVITVAGYLPSLVALTKTRLLRPLLAPSPKDKSGYGRLIGIAQSVVQERIRKGNTLNKPHLADDFINARREGGQNPPRED

QVLAEVRSTIDAGSDSTAAAITGAINFLLRNQHAYDKLVAEIKDFSASGQLSSPVTYHETTKMEYLNAVIKESLRLFPPF

QGAFTRLVPESGAEVMPGVFVPGGVEVGVNCYVSNRNKDVFGEDAEEFLPERWLTIDNPETRRKEKYINTFGYGSRICLG

KNIAMIEIGKCLVELLRNFHISLVNPDIPMKEVNVLQMLVEDFFLTMRERENKF*

>CYP567W7|1666549|Chove1

FVWRAIYNLYFHPLAKVPGPRIYAISSFYTSYHWVSGRYPYAIRQLHEKYGTAVRLAPNQVSFCSEGSWKEIYGHVGGRK

QFLKSNFYTQQFPHIVNERDPIKHSEIRRLLSHGFSPRAVGEQEAYVHEYVDRFIQKVNIYATKKDSPEEGEEMTKWYSF

LTFDIIGDLAFGEPFGSLDDGRPHFWVSIILDNIKAIAWSTIFRQFLILRPVAIWLTPKALMSKRIQHFEYSRDKIARQE

PPHPKKDFLSNMLDKKELKGITMDNLGANASILITAGSETTATFLSSTSFYLCRTLSSYQKLVNEIRSSFNSYNEITGQA

TENLTYLKAVIDEGLRIFPPVPIGMPRESPGENVEGIYIPKGVEVFTSSYSATHYSGNFHRPDSFIPERWIDPNCTDKKR

ASQPFLLGSRGLALLEMRVILAKMLWVYDMELMDPNLSWGKDNNCYLLWDKPKLPIRYTRRAGIVVPPLDG*

>CYP567X1|1670327|Chove1

MAVSHDFRSALVQVQDIVKDVPAWKLCVYATLLFPLYAITMGIYNIFFHPLRNFPGPKKAALSNIWYSYVWLSGRYPHTM

HALHEKYGSVVRVAPNQLSFNSSTSWKDIYASRGHQIFRKSGFYAPDKQDPETNMLRESDPIKHGQIRKMFSHAFSAKSL

MEQEPIVQENIDLFIEQIGKHGTGKDGLDMVKWYNYCSFDIIGDLAFGESFNATKEGKTHFWISLILDSIYAASFIDVIR

RFPWLNKLIPSIVPADAAERRERHLNYCRDKVNLRINSDNKRKDFLTNVLDNYRDQISDEELSSNVQFLIVAGSETTATT

LSGLTYYLLRNPHTYQRLVSEITSAFTSYSQITSITARKLQYLGAVIDEVLRVYPPVPIGLPRYSPGETVDGYFIPKGVE

VSTSSWAAGHDPKNFHEPWTFKPERWLDGECKEKDIREASQPFSLGPRVCLGRNLAIMELRLIICKMLYTYHMELLDSKL

DWERDSPAYVLWVKPDLRVRLHKR*

>CYP50043A5|1677066|Chove1

MGLLNLDVVSFAVYLFAGWAAYLVLLVFYRLYLHPLRRFPGPSLAAATGWYAGYWDLHMSGHMVKHLVDLHREYGPVVRC

EPNHLHFSSPEAYSTIYSASSKLTKDPNLYLGFSASESVFTILDPALARTRREVIGPMFSRRAVLSLQPLIRDKIRTLCD

KLSEYASRGEAANIARGFRSAAMDIITQYCHNECLDALEVEEFKHELFLSIRANSETFWIVKYFPIMEWILTLPRSVAFW

LWPELKGFLAITDTMEDQVSRYTKDPSLLEKSAHATVYQRFLDPEVKGGIPSHSSLVGEAQNLLFAGSDTVAGALAFGTY

HILTTPGMQEEFFAEICQLWPALEEEPTYEQLEKSAYLTAVIKESLRLSHGAVTPLSRVVPSSGMTIQDQHIPGGTIVSM

DAPTIHLNPTLFPQPDLFLPSRWLDSATKDLDKYLVAFSKGPRACIGSNLAWAELYIAFAAVFRRFEMVVHGTSKEDMEW

MDCFTPITRGDLKVKMRVREE*

>CYP5093H2|1681200|Chove1

MYGSLVTVWTGRRPTIVIGDPKVACDLLDRRSAIYSSRPRFVVMGELFTNNDSLLTMPHGDKWRKTRKIFHMGLHRRACE

SYKPIQEAESQRLTRDLLVTPGIFGKHLERYASSVMICVAYGRRVDDMEDPIVKKIYDRMAYMSTLNVPGAFWVESFPIL

KLIPDCLAPWKREVKRRAKDSTEMLSKLALDVRDQIRKGDAPASFTKTLWEKREGNPEALSEREIAYATGSLFGAGSDTS

SATLMSFFLAMTCFPRVAAEAQEELDRVVGRDRSPTWSDEPNLPYCNAIIKETLRWRPVAVMGGTPHASIKDDQYNGHFI

PKGTTILGNLWAIHHNEKYFKDSHDFIPERYLGSGRIDGMEPYPHRDGHSAFGWGRRICPGKQLAENSLFITITRVLWAF

NISKATDKDGQEITPNIFAYTDGFNSKPQPFQCRIQPRTPGIQQVIEREARLGDQFLDKYKCN*

>CYP548BE2|1683722|Chove1

MITFLNLVKLLCTSFAVYISSLVLYRLFFHPLARIPGPFLAKITDWYTVYYAYRGDRYLALYKAHEKYGPVIRFAPNLIS

FNSASSLKAIYGHSPLSRSLQKSQFYSAFPAVKGVYNTHNAISKVEHGRKRRVLSAAFSDAALKSVEDLVLSNINVFSSK
VNDAFKQGVGVDMGDLFSWLTFDVMGELCFGKSFAMLTEEKTRFVTDLISQAAHNHYINGNYLPLTTLKLSKVLFPTISR
DRWRFIEHSRACANERMSLGRGYKKDFFYYLLDAKDPETGEGFETKELWGEANVLMIAGSDTTATAMSAAMFYLCRNPQI
LETLKNEIRSTFTSKDQIVGGKELGDCHYLKACIDEAMRLAPPVPGLLPREVIAKEGVEIDGIYIPEGTVAGTPIYALHH
NPAYFPDPFSFKPERWLSTYTSNDDVEAARSAFTPFSIGPRGCIGKSVAYMELRLTLARLLFEYDCEEVKTEGKAALWRE
GYAMVDGEYRLMDHFTSRKEGPIVKFSKR*
>CYP6001E1|1700003|Chove1
MSFLGLFGGGSSANPQSEYGDDSGATKEITYTGILEDIKAAGGKIPEDLKLLVETGLQGVSKGPIDDKELVMERLIGLVA
SLPQNSANRKKLTSTIIDTLWDSLQHPPLSYVGDKYQYRQADGSHNNIMYPDLGKAGTEYARTVRQDKKLAGAKPDPGLL
FDLLMARGGNFKQNPAGISSVLFYHASIIIHDVFRTNREDFSKSDSSSYLDLAPLYGSSQQEQDQIRTKQDGLIKPDTFS
EKRLLGLPPGICILLVMYSRFHNYAAKTLKAINENGRFSLPRTHATDTPENQTKNLAKLDNDLFQTARLITNGLYVNISL
HDYIRGIANLNHSESTWTLDPRLEIDKSFDGEGTPRGVGNQVSCEFNLLYRFHSGVSKRDDTWTKDFFGKLFPGQDPLSI
GMPQLLEGIKTFEKSIVEDPSKRTFGGLKRTGADGSGAFNDDELVKILKESIEDPAGAFGANTVPEILKPVEVLGIIQAR
KWQVASLNEFRAFFNLKKHKTFEDINPDPYVANTLRKLYDHPDLVEMYPGMFLEDTKPRMDPGMGLCAPYTVTRAVFSDA
VTLVRSDRHLTLDYTPANLTNWGITEVAQDYDTLGGSKMYHLILNAFPSYFKYNSVYAMQPFYTPTESRKIFDKFGKSHL
YSFEPPAKSSPPIPILTHAGLKRVLNDQKNFKVPWGEAMEALNNEHDFMLAGDLSSHTQQRNLVGDAIYDITGSRKQFKD
YTEEITLKLLKREVYQLGKVPFNQVDIVRDVGNLASLHFAASLLYLPLKSNENPTGQYSEQELYKTLTDLTWFVFSDSDP
TKSWEHRREAKKSIDKLGGVMVEEIKKFQPPIGIWDKLTGGHAVRSPPPSLKDYGSNLVKRLLNSGRSVEDVAWMLLWSG
CAFVANSACAFAQLIDFYLQDDNRKHWAEIQRLSTLNTPEADKLLTKYTLEGTRLSNSLGIFRTVDPVDSQTITIRDLNK
DVVLKKGEKVFVSFVYASKDPTVFPDPEVVKLDRPTELYITHGEGQHQCLGKDINIIQNTYMLKALAKLRNFRRAPGDEG
KLKFIPKPGGIKLYMNADWSKFTPYPTTMRVQFDGPIVA*
>CYP6136A2|1700600|Chove1
MHLITLVQPVYALIQHKLYLLAGLLILALAYWTHAYLTCPFRTQKIPGPFLGKFTNAYRWYHVMRHTWHRDLMDLHKKYG
PIVWIAPDEVSVSDPTLRNVIYGFQNHKKDFTFFKKSPSYEAGSINQDFSFIFEQDPEKARLGKYHMSHFYSERGLFNLE
ENFDKAVEELIEGLDKHHARTGAPCKMVDWTEFFALDLVAQITADQSAGFCLAGKDINDTAYGARVIIRAVGVLTPMPWV
LSVTSRAIRQNFLLRFLINLYRNLLLLPTFTFDTGMEDLKALKEKNPKHLLAKFYNAQSRMREHYPHGNQAEGTTIQIFN
LIAGALGVVPHCQVRLIQELAAHPEVVQKIREELATTNNTLNFEDFLRYNNRQNKYPILESAVREAVRLTPAASFPLSRK
VPPSGCQLHQYNIPPGYNVSMASYQVNYDEGYFGPDVAEFRPERWLGDHPTEMLDGEKRSMKNYLEAGWLTFGAGGRVCI
GRNLAMFMMLKFTAALVREFDIQVVKQPVEYYTLMTEMLGMEALLTRRCAV*
>CYP51F1|1716686|Chove1
MGVLSTLLVPMGPVYGELMKLGEPALCGIGFVAFVVLSVIINVLQQLLFKDPTKPPIVFHYFPFFGSTVIYGMDPYKFFF
DCQEKYGDVFTFVMLGRKMTATLGPKGNDFVLNGKLSEVSAEDAYAHLTVPVFGEGVVYDVPNHVLMEQKKFMKFGLTSE
NFRQYVPLIVEQVEDYIKKSKFFKGAKGSVPLSEIIPELTIFTAARTLQGKEIRDALDGSFAKLYHHLDSGFTPMNFLFP
WFPFPQNKRRDHAQRTMAQFYMDKINKRRADEHKDDQERSDMMWNLMNCSYRDGRKVPDKEIAHMMIALVSSLTHPDLVA
QILAEQKRVFGDELAPLSYEKLVFECTFLTHIIRETLRMHPPLHSILRKVKSPMHVDGTNWVIPKGHYLLAAPGVSAMDQ
NYFKDPNSFDPTRWEGQKAEEETEKFDFGFGLISKGTASPYLPFGAGRHRCIGEQFANVQLMTIMATFVRNFEMQRPGGG
NDVPAPDYSSMIALPTPPCTIEWVKRDP*
>CYP504A39|1719882|Chove1
MSVLLFVAIIAVCYLLFRYAYSTETPKIEGIPEVPGLPFLGGLVALGECHARKAKEWSEKYGDVFQVRLGRKRIVFANSY
ASVKDLWITQQSSLISRPKFHTFHGIVSTSQGFTIGTSPWDASCKAKRRAAATALNRPAVQTYMPVIDLESAVSIQELVN
DSQKRKFGINPVAYFQRYALNTSLTLNYGYRIDGSVENEVLKEIIDVERLISNFRSTSHNWQDYVPLLRYFKGKNEKAVE
ARKRRDAYLEVLLKRLKDDIRSGVDKPCITGNILKDPEAKLNHEEIKSICLSMVSAGLDTVPGNVIMGLGYLSSPHGQTI

QQKAFEAIREVYPNNDAWDMCLKEEKVPYITALVKEVLRFWIVIPIGLPRTSIGDMTYNGAIIPAGTTFYMNAWAANYDP

AQFTDPEAFSPERYLNSAPEGTPHFAYGAGSRMCAGSHLANRELYAAFVRLICAFEITEADRVEDRPILDALECNGIPTS

LTTDPKDFKCKFRVRDEGLLNGWLEKSLGDVKM*

>CYP512CQ3|1742126|Chove1

MEVCYFGSTSPPPTFRLPPLLESLLQHRITREVVLLLTPWGLTLSVTTCLLLWTFWKSYSLRVNVPTIGASPGPFGGWRA

MFKYFSHGSEWIAEGYDKYTPTAQTFKIPSISRYILFPTSTKILEEMMAEPDHVLSFGEALTEKLAVEWTLHPSIKYDPY

HLQLIRTKLTQRLSTILPEVMDELVTAWGDGAGGDVGKEGWTRVRVWEVMVVVVARTTNRMFVGLPLCRDQEYLDNVIAY

SLKVVKAGAILDILPRFLRAPLTNLLMQKSHHYALMRKHVGHIFRERKAKMQECLASGQEWKDCPDDLIQWILDFSEEEG

GSSGSGKRREEEELIARILFMNFASIHTTATTVTQAIFDIAAHPELQPDLHIEITEVLSKEGMSKQSLTKMKKLDSVIRE

SQRLNTISTITMMRKSLIPYTFSDGTHLPTGTWIAAPATALQRSLLSIPNPAVFNGFRWERLRVEEEEAGRPGRYAAVTT

SFEHLVFGHGKHACPGRFFAVNELKVLLAHVVGRFEMRCLPEQGRPKNWIFGVACRADPEGVVEFRLR*

>CYP5945C1|1769407|Chove1

MAFDVMADLGFGQKQEESMQSGAGDPSYMEFLHGWMRASTVLTSLRNLCELGAYIPQDAESKEFNKMGEKMLGARQKMGK

SRQDIFTHLLAEDKESGVNFTQAQLLINAQMLMVAGSDTTAVTLTCLFRLLAMHPEKQQKLRKEITEAFPNGEIPTCATT

AALPFLNGAVQESLRMWPAVPSGPQATTPPSGYTIAGTYVPGNVEVRIPHMTMMSDARYFPRPDEFLPERWTPEMPELVK

DKRAFIAFGFGAHSCIGRPLALNEMRTAVARVIQRFEVQLGESYDDKVFRDGWRDYFSVKLGPCPLKFVPRKM*

>CYP6608A1|1773898|Chove1

MPSLMVILAPIFKSVFIRTFAQILPLALLAIVASCVYSRYASPLKKVPGPVLASFSRIWLNVGLVARGDLHGGILDAHRK

YGPVVRVGPNKVYFSDPALILEIYGAGTKYRKSDLYMMSDPEPGHGSTFSERSAAKHAELRRRVGPAYSNSAVLQMESYI

DTLVSAWIHALSSEFTAAKTSFKSCDFARWSQFLTYDVISELAFGKAFGFIATRSDYKQYIASMQQALPIIVTMSYFEEL

VKLMTLGWVRKFTIPSIKDETGYGNLTKTARELVSERWAKGGVGERRDMLQRFYEQGMTEKEAMVDSTLILVAGSDSTAT

AIRAAVMFICSNPGVYTTLKSELATNLTRTNPPSVVSFSIARKLPYLSACIKESLRLFPPGAGAMERVVPAGGATLNGYF

IPENTVVCIHPWPVCRDEVFGEDVDSFRPERWLEEKDEKRLAKMEKTLDFVFGAGPHYCLGKQIAYVELYKSVAELFLTF

DIGLSNPTQPWKSREYGFFLQRDMMVNIKRVDG*

>CYP617Y1|1791406|Chove1

MSIANLLAGCAASTILLSRYTDFSAITTFLLLSSLVLTCRLFYGLVLWPKLLSPTRHLPGPSNPSFLMGNFATIREMQSG

APQVIWMRTIPNSGLLRYLGAFNMERIFPTTPEMLKEVLHTKSYSFIKPPLLTKDVGNIFGTKGLLFAEGEVHKLQRKLM

LPAFSHAHIKGLVPGFWSKGMEMTEKVAEVVRASGDGGEEGVVVEMGKWFSLATLDIIGSSGFGYEFRALESASISGSDT

TVENSGSELADAYNTIFNMGSPSRNVAILSMIFPPWLINSLPLKRIRDVAQASLTIKRVTAQIIATKKSAMAATPITSSE

DSESKDILSIMLKSNTYTGPDGESSMRDQMMTFLAAGHETTATSMIWAIHALSLPENRHIQSRLRAEIHAAFPSGAPETI

TYDQLSTLKYLSHVTSEVLRLYPPVAVTLRVAAEDTSLNGVFVPKGSYVVLSPFAINHSVALWGADAEEFRPERWAGGED

GTAAVESNYGFLTFLAGPRGCIGNVFAKVEFKCLLAATIGKFEFEQDGKREIVVKGGITAKPQGGLPVTVKEVVWG*

>CYP5959A9|1792378|Chove1

MSTTVKLITYTKDELLHGYSLYGVALTLFFSGLFFFLKKEWSIHSPSKLPIVGIDSPGYFGLLKARRHFVTNGRQILKDA

YYKYYKKNFVITTNSCEKIILTFDQTKELSNAPEDTVSFIHAINELMMADYTGLNRAPGSHILKDVIRIKLTQNLGSMKE

AQMEETQFALNTEMPQCTTDGCYSPTPILICLVRMLIKLIMAEWTSVNIYSATLRMVARISGRAFIGLPLCRDEDWLTVS

INFTNDVFNTAAKLATISKLIRPFYAYIQNSTKAIEAHRRKARALLAPTIQRRLEEETLGKESGTAHREYNDLLQWLSDR

IESQHKNVDHLAELQLSTSMASIHTTTSSLVNTILDLAEHQEYIQPIREEIEAVISTNDGMLDRRALRKMRKTDSFLKES

MKARLGLLTFNRKVMKSLTLSDGTYLPKGTLIAAPHSIFSTDPDYIEDPEAFDGFRWYKKALALKDGMVDNNYSASAAPE

NLAFGQGKHACPGRFFATEEIKLILIYILLQYDLKYPEGQSRPDNIDNGEFTFPDFRQKLLFKKLPGPKKFSFL*

>CYP61A1|1793167|Chove1

MDEIANSSTFVPTSAVSSTEQPFQTLVEGFETIGKTLEGLSGWQIFFSIVILSITYDQCLFSLSSLPLFCKFRRDARLLI

YIRFADSRFCRYIWNKGSIVGPFLKIPFMGPFLESVNPKFSEYLAKWNSGPLSCVSVFHKFVVIASTRDLARKVFNSPGY

VSPCVVDVAKKILRPENWVFLDGRAHVEYRKGLNGLFSRQAMGIYLPGQEEIYDIYFKRWLELSKEGKPIPFMSEFRDIN

CAVSLRTFVGHYISDEAVKEISENYYKITAALELVNFPIIFPWTKTWYGKKCADFVLNEFAACAAKSRIAMMEGKEPGCT

MDSWIKSMIEAKESDAAKVAAAKGLPQIREFSDMEISMTIFTFLFASQDASSSASTWQFQIMADRPDIMKKIREEQLRVR

DGDPHKRLDLDMLDKMVYTRAVVKEQLRYRPPVIMVPYEVKKSFQITPEYKVPKGAMVVPTIYPALHDPEVYNDPESFVP

ERWLEGGEAEAAKKNWLVFGAGPHVCLGQSYAIMNFMAMIGKASLHMDWEHHATSKSEDIKVFATIFPEDDCFLVFKERD

PYAPA*

>CYP50115B1|1815467|Chove1

MTKDYAEAGKEMDYAYWSQFFAYDIGFGKPSGFVKTASDVHGLIEAFHGAAKAIGLMARCEKMKDILRSEFATKYIIPDI

NKESRLGRIIVTCRGENFYGESASQIDLMQHLLQAKLPGDSPLPIQDTKAELLILMFAGSDTTAATMRHLLLYALRNPKV

YTRLMEEMDTFVATHPPFDVIPYDDAQSLPYFTACLRETMRIAPSTPIGMPRHRRGGITLPEPDGRYIPEHIKLICDSFV

TQKDKSVYGPNTHEFNPDRWLGKGNEEKVREFEKYDLHFGGGARVCLGENIAYL*

>CYP6498A1|1825200|Chove1

MNAFHVLTCGFNRALWPYLPQWAYFKIFWRDWCHHTKFELFEKYGDVICAVSPGGVTIYVGSVEVVRQMYERRSDFPKVT

KVYEYVRFYGDNVLTLEGVEWRRHNKYTRPPFNEAVHKVVWEEGVKQAHTALNVWSRKNSCVKVGRDLRTISMNVLSLSN

FGVALPFDYESETTEHFGQKNIPPGHRMSYGNAVNHVLDNIIALVITPKWLLRNGPESLKKTGQSYEELGLYLKELIQNH

GKAGSSDRKNLLRSLVKASTGDGLGKGSGLTDTEVIGNAFIFAVAGLETTTGTLHYAVMYLALNPDVQDWLYKDLQEALK

DEDQDPSKWEYEKVYPKMASVLCVIHETLRLNAPHMHIPKWTADKYQPVNWKGKQCLIPPGASTYITITALHYNPFLWGD

TVKEFQPQRWDLRSSVGWVHTDPRTGIKAPTTPSETPWTETQSAPYCHLRTPVKGAFAPFSDGWRACVGKNFALVEMTAV

LATLFRGCSVRIKRQEGETQEMADNRGKSAISNSESYLTIMIRHDVELEWVRR*

>CYP567W7|1825644|Chove1

MAVHLRVSDLLKLSPRELLGGLGALFVVCFVWRAIYNLYFHPLAKVPGPRICAINSFYTSYHWVSGRYPYVIRQLHEKYG

TAVRLAPNQVSFCSEESWKEIYGHVGGRKQFLKSNFYTQQFPHIVNERDPIKHSEIRRLLSHGFSPRAVGEQEAYVHEYV

DRFIQKVNIYATKKDSPEEGEEMTKWYSFLTFDIIGDLAFGEPFGCLDDGRPHFWVSIILDNIKAIAWSTIFRQFLILRP

VAIWLTPKALMSKRIQHFEYSRDKIARQEPPHPKRVQSKTTRKDFLSNMLDKKELKGITMDNLGANASILITAGSETTAT

FLSSTSFYLCRTLSSYQKLVNEIRSSFNSYNEITGQATENLTYLKAVIDEGLRIFPPVPIGMPRESPGENVEGIYIPKGV

EVFTSSYSATHYSGNFHRPDSFIPERWIDPNCTDKKRASQPFLLGSRGLALLEMRVILAKMLWVYDMELMDPNLSWGKDT

NCYVFWDKPKLPIRYTRRAGIVVPPLDG*

>CYP6001C26|1827170|Chove1

MTSNTNGTNGATNAHSNGHSNGASNGASNGVSNGKTSKYATQKKPPPPVAKPTRAAVDSTFEQFANLLHASNRPLPNRFG

DGKDERPPEVKQTGILTDINTLRRGGFFWESVGTLWTLLKSKVKGGPVDDKTMIMERVIQLTTRLPATSKVRVALTTTQV

GQLWDSLQHPPQSYLGNEFTYRHADGGYNNVFCPQLGRSGSAYARSVKPLAKMPGAPPDAYTLFDSIFSRGPNDEHFREH

DNNISSMLFYTASIIIHDLFRTNHVDPNISDTSAYLDLAPLYGNSVEDQAKIRTFQDGKIKPDSFYDKRLLAFPPGAPVL

LIMFGRFHNYVVENLKAINEGGRFSLKFPRYPNGDDDATRQAKAVKQQDEDLFQTGRLVTCGLYINFILNDYLRTIVNLN

RVDTTWTLDPRFEASKAYNPDGTPAGVGNMVSAEFNLVYRWHSCISKRDDQWTKEFYQTLFPGRDIKDIEMPEFLAGVRG

WERSLSDDPAQRNIAGLERKLDGSYHDDDLVKIITESVEDVAGAFGARNVPHVLRLVDVLGIEQSRKWKVASLNEFREFF

GLKPHATFEDINPDPAVANALRQLYDHPDFVEMYAGLVAEADKKPMVPGVGIGPTYTISRAILSDAVTLVRGDRFYTTDY

TAAHLTNWGFQEASSDPAIVYGCVGYKLILKAFPNHFKFNSIYAHYPLTIPAENHKIHTALGTVDQFDFERPVYTPIRIP

ISSYSATKQILCDAQNFKVTWGAGFDFIMRADFMLSGDKLTNSEQKAFVRDRLYLQGVDWKQQIRQFYEETTEKLIRKKA

YSLGGAYQVDAVRDIGNIAQTIFAASIFNLPMKSEDHPKGIYTEQELYTILCVMFIVIFFDTDSSKSFPLRQACFKVVRQ

YGTLVEAQVKAIKSWSWLQGVWDPLNIRGRNKSSPLKSYGWTMIKRLLESGKSPYDVTWCYIVPIAGASAPNQGQIFAQV

LDFYLEDKNAHHLAEIQRLAHVGTEEAWETIKKYALEGGRLAGTFGLYRRVEADNITIEDNGRNVELRKGDMVFVNFITA

SRDPVVFPDPLEVKLDRPEASYMQYGDGAHECLGKAANIIGLTTMLMQFGKLKGLRRAPGPQGNLKYVPKPGGFKVYMKE
DWSAYWPFPTSMKVRFDDII*

>CYP567Y1|1827481|Chove1

MINVTSLPATASDGRAFYNLFFHPLSKIPGPKLCAATDVSTHRRPLSMSSLHDKYGTVVRIGPNQLSFSSASSWRDIYGH
SHGRKQFPKSHFYAFGTRHLINERDPQKHSEMKRKLSHGFSVKALQEQEDIVQNYVDKLIRQINVYATRPEGDEMVKWYN
FFTFDLIGDLAFGESFGSLNDGIFFPSGLILGNIRAIAWRSVARWFPVFDKLGVWTVPKRVMEMRIKHSEYSRKMIIQRM
NMKTTRRDLLSGQFGPDGPGMTIPELSGQASTIITAGSETIATFLSGTTYHLLKNPRVYNLLAEEIRSAYNSYEDITDIN
ATKLKYLSAVIDEGLRVYPPVPIGLYRESPGETVDGIYIPKGVELSTSSWTTCRSPENFHSPEEFKPERWLDPECTDKKQ
ASQPFSLGSRVCIGRNLALKEIRLLLSKMFWVYDMELVNKEVDINRDSTSFVLWNKPDLWVRFARRPGVEVPGLDGE*

>CYP6001C26|1831942|Chove1

MTSNTNGTNGATNAHSNGHSNGASNGASNGVSNGKTSKYATQKKPPPPVAKPTRAAVDSTFEQFANLLHASNRPLPNRFG
DGKDERPPEVKQTGILTDINTLRRGGFFWESVGTLWTLLKSKVKGGPVDDKTMIMERVIQLTTRLPATSKVRVALTTTQV
GQLWDSLQHPPQSYLGNEFTYRHADGGYNNVFCPQLGRSGSAYARSVKPLAKMPGAPPDAYTLFDSIFSRGPNDEHFREH
DNNISSMLFYTASIIIHDLFRTNHVDPNISDTSAYLDLAPLYGNSVEDQAKIRTFQDGKIKPDSFYDKRLLAFPPGAPVL
LIMFGRFHNYVVENLKAINEGGRFSLKFPRYPNGDDDATRQAKAVKQQDEDLFQTGRLVTCGLYINFILNDYLRTIVNLN
RVDTTWTLDPRFEASKAYNPDGTPAGVGNMVSAEFNLVYRWHSCISKRDDQWTKEFYQTLFPGRDIKDIEMPEFLAGVRG
WERSLSDDPAQRNIAGLERKLDGSYHDDDLVKIITESVEDVAGAFGARNVPHVLRLVDVLGIEQSRKWKVASLNEFREFF
GLKPHATFEDINPDPAVANALRQLYDHPDFVEMYAGLVAEADKKPMVPGVGIGPTYTISRAILSDAVTLVRGDRFYTTDY
TAAHLTNWGFQEASSDPAIVYGCVGYKLILKAFPNHFKFNSIYAHYPLTIPAENHKIHTALGTVDQFDFERPVYTPIRIP
ISSYSATKQILCDAQNFKVTWGAGFDFIMRADFMLSGDKLTNSEQKAFVRDRLYLQGVDWKQQIRQFYEETTEKLIRKKA
YSLGGAYQVDAVRDIGNIAQTIFAASIFNLPMKSEDHPKGIYTEQELYTILCVMFIVIFFDTDSSKSFPLRQACFKVVRQ
YGTLVEAQVKAIKSWSWLQGVWDPLNIRGRNKSSPLKSYGWTMIKRLLESGKSPYDVTWCYIVPIAGASAPNQGQIFAQV
LDFYLEDKNAHHLAEIQRLAHVGTEEAWETIKKYALEGGRLAGTFGLYRRVEADNITIEDNGRNVELRKGDMVFVNFITA
SRDPVVFPDPLEVKLDRPEASYMQYGDGAHECLGKAANIIGLTTMLMQFGKLKGLRRAPGPQGNLKYVPKPGGFKVYMKE
DWSAYWPFPTSMKVRFDDII*

>CYP6271A2|1838604|Chove1

MALDAVISYVREQAPGISRGTIASTVFVAWLVYRIGIVFYRLYLDPLSKFPGPKLAAATSLYEMYYDIVEGGTLVWKMDE
LHRKYGPIVRISPHSLRLRKSSAYHEIHRMGTPFTKDFRFYHLFGIPRSSFSTIDINLHRQRRSMLNPMFSRKGILDLEF
LIKEKIELLCRRMKEHEVQDKIFNCHQAFAALTVDIVTEFAYAKSYDVLSTPDFTSRTFDAFDAQHEAFLVLKHFPLMAK
IMQNLPFWILLRLMPDGAGFAELEEDAKVHLSALFDRIRTGTMEKNTTHRTIFEEILAEHPDPKNADPAELTDEAMSVVG
AGIHTSRWALCVGLLEIARDPLIQTKLYEELKLASPDIDAEFSYLHCEKLPYLRGVILETLRLSYGVMGPLPRRVPRGGA
VVGGYHLPGDSTIEMDNYSLHHDEDIFPDSRRFWPERWSTPESKQNEKFVNAFGAGPRQCLGINLAMCELYLSFATIFRR
FEIDISARGTQRMKYKEHWMGILRDEPLKCKFISRKE*

>CYP530G1|969883|Chove1

MAIPVFWTTTPGLALGVCLASFIATLIYFAKGTSRGKNFPPGPPTLPILGNMHLVPQERPYLKFTEWAKQYGGIYSIKIA
KQTIVLISDVKILKELYDKRGAIYSSRPLPHIGAEIVCPDQTHIIYMPYGETSRNYRAQYHQFMGPGKVEQILPWQSAES
TLLLKKIATSPDRYYEHTMRMATAVILESVFGVLPKDYDDPEVTELWTVQREFSEILALTGPPVDHFPFLKWLPDIVSPW
RIHARSVRAMHRKLYFRLLNLNKARMEKGERYGTFVEKLIDDKPKHGLSDERIAYVCATLMEAGSDTTASQALDFMMALL
AFPDVLKKAQEEVDRVCGTSRLPTLDDRDQMPYIEACVNEVLRWRPPLPYGAPHLLMKDDWYEGYFLPKGTVLFQVQWAM
NMDENVYEKPQDFIPDRYIRNRFGTKFDAEKDAETGRKEQYGFGLGRKICPGQWFARNTLFVLFAKLVWAFDMKIPTDPK
TGKPAPLDTDVRTAFMDGLTTTPFKFPVDFKIRSKAHEEAMNTDLVASDKIFAKYGGATV*

>CYP539A46|1699914|Chove1

EHWESRFKISGGHTVEITVVGKRIIITDDPENMKAVLASQFHDYGKGEPFHRDWKSFLGDSIFTTDGQLWHASRQLIRPQ

FIKDRVSDLDTFERHVTHMLEQVPKDGVTVNISNLFYRFTLDTATDFLLGQSVDSLGSPQVEFARAFADIQKHMSGKSRL

GPLGWMLPEGKYKKDLKVLNSFVEPYVEQTLKMRPEELKSKNEKSYNFLHALAEFSRDKQMLRDQLVAVLLAARDSTAAT

LSWTLYEIARQPQIVQKLRAEILDRLGPDGKPTYTDLKEMKYSQHVINETLRLYPAGKINNLNQFCLTLY*

>CYP52AV1|1787345|Chove1

MGPSFMSLFLLVIGIHCLLKLTSYLITAHQNRKFAKSHNCLPPRRLPSAFWGLPNWVHIMRAAKRGDVLEHIASRFPKYG

NTWKGRILFVTSIGTIEPENIKTILATGFKDFSLGSERHDNFYPLLGDGIFTLDGAGWEHSRANLRPQFSRDQISDIEAL

EVHVQRLMNRLPEGDGKVADLQPLFYCLTLDSATEFLFGESVDSLLSPELNPTGAVSGRGGEEQMSFARAFNVSQAYLIK

RARLRGLYWMINPPRFRRSNAIVHALVDRYVDMALHPEKRTRKVSGKYVFLDAIAAETKDAKYLRDQCLNILLAGRDTTA

GLLGFTFWLLARHPHVYQKLREEILSAFGTSRNGEGRRPSFSALKDVTYLRYVLNETLRLYPSVPLNGRTAVRNTILPRG

GGEDGLSPVFIPKGQRVDYTCYGLHRRKDLYGEDADSFRPERWAEGVGRGWEFLPFNGGPRICLGQQYALTEAAYTVTRI

LQKYARIEVADTYTGPMMDLTLTMAPKKVLLKLWKA*

>CYP52AU1|1807266|Chove1

MELHDQMGDTYASTLFGMRAIRTRDPRNIKAILATQFEDYGLAQERKAHRSLLGNMGIFAVDGLSWSHSRGLLRPSFDKA

NVSDLSQLEGFMQIFFSRINELPHQPFSNARAVELQELFQCLTMDSSTNFLFGNPIGALASQKYTPANGEMTFDQAFDIA

QYGLAMRAPLSSLYWLYNGREFRKACSTVRTQAAIYVSRTLRKLKLEGRGTGGKDSGKKYIFLEELAERTQDPQVLQDQV

LSVMLAGRDTTAAVLSWTFLCLAKHPAVFRKLRAEIAAVVGVDGDARLPTQAELRSIQYLRWIIQEVLRLYPPVPVNGRR

ALHATTLPYGGGPDGNSPISIRKGERIVYSTFSLHRRTDIYGPDANAFRPERWGEEAMRKVGWSWLPFNGGPRICLGQQM

ALTHASYFVTRMMQVYKEITPQDFALANDVAYDTKLVMASGRGVHVFLS*

Kalaharituber pfeilii F3 v1.0

>CYP5108D1|621937|Kalpfe1

IPSAVSGLPLIGNITEYSSNPIEYLKNAKAKYGKFFEVNMLFTNTIWLMGSDMNKLYTEKKEDVWSFGGGMGLFLNKVVV

PGYFSNLKTFVNSVNRGVNRPVALRAACTVMDDQSKKTLGSWTQDDKINLFEAVSLLVHQILTRCLMGEDFFDHHFNELN

HLLHEMERDIGHVFNFILPEWVPHPPARRLRAARDRVVAIFQERLSQREKEPERWANSEDYISYTLRDPATAHISHLYPA

HHTLLMFAAHTSTVANISWTIIELMRNPSRINILKEEMDRLIPPGTSFFETYARPAMLQSALRESGRHYSGINMLRLSRE

PVQLPSGISVPQNTVVSISPYLTHHDPALYPNPQEWMPERFIENPDLPREMNKDGKVSYLPFGAGAHRCPGEKFANMLAS

IAVGMLIRDYEVSWPKGLENQNFSELDFNKIGAAWSKNPVYISVK

>CYP567S3|653905|Kalpfe1

YNLYLHPLAKFPGPKLAAISPARLAFSDHSNLELLLTLHFATSTGPVIRVAPNELSFSSAAAWKDIHGHRPRGSAFVKSD

FYFPADDIYNRLNIFTEKDVVRHAELRRLLAHAFSAKALQEQEELIHKHIDLLVQRLGKRFADKEKGPDAEHCNLVHWYN

FTTFDIIGDLAFGDAAAFECLENEKPHPFVTMILNSAYVMCVADIFRRYPFMLKLGYFFIPKKLLKDRIQQIQYSEALVK

ERMSTDTDRKDFMTYILDKKDAFPHIDIPSNAEVLVIAGSETTATQLSGLTYYLFKTPDAYQRLTDEIRTSFNNYSDITS

SSTQHLPYLNAVIEEGLRIYPPAPFGMPRISPGAMVAGHFIPPGTEVSVHTYSASHSPENFAEPDRFLPERWLQDGNFND

RKEASQPFLLGPRGCLGRNLAYMQLRLILAKMLYCYDLEWVNTDLDWEKKSRTWLIWAKPELKVHIKSRLGIRWDELGLP

DHA*

>CYP6902A2|656221|Kalpfe1

MSILLQALVGLLLGYLIYHLYCLYCNIRIARTIGLPYVVTPVFEFGVFWMIFGRFLHPLIKCYLPSFLTKSWEPFLAPDW

HWNMKHGACKLMKTDVFLIVSPTAIIMSVADADMINEIVTRREDFPKPMNVYPPLEIYGMNIASANGEQWKTYRRIITPS

FNEKMNQLVWTTFVEKTKLLRDNWVTPVAPSTEPPDVRTLSNGCYRLAFHVWSWVGCRLQLTWPKQLSGNSFEPYPDEKI

GQSHSMSFQSAMHFVLARCFFLAGAPKWYLQLTPYKPHRKAWLAYKEWEAYMKEIAEERKKELDSGKATTVDTLSAMVSA

YYSGAEGKGEKGKRTFTKEELFGNILFMMFGGHSTTARTLHYAIMMLAIHPEYQKPLQEDFSKILGDRDPDYDKDFKPLM

SGWCGAIINETFRLYSQVANIPKATATPQTIVYNNKMHVIPAFVVIHLNVNAVQRNPKYWVLPNQTEEEAQINEFRPQRW

FLNNDSSNFSSSSSDESDNENDGTNSRGHTSKSFKPCKGSFVPFSDGPRVCLGKKYAQVALMAAMSVLFREHRVELVVKE

GETWEEARVRARRYVENSGMGLTLAPKGQEVGVRFVRNRV*

>CYP573C1|663942|Kalpfe1

MWLGLIGHIIRNRYFHPLSKFPGPFWASVSNLYCSLMILTGKSQEMEFDYHKKYGPIVRIAPNLLVVSDPRMLPVIYQRK

ADKTDFYITGTLGKGPESVFNVRAHQEHYHVRKRLAAAYGLRATKGMENLVSERIVEWTAQLGRLFADTGNVLDFSMWSQ

FLAYDVITELGFGESMGFVRRATDVGGLIKCFHYALPAIGSLARLPILTKMVVGWDCVTSKPTDKYGLGPVMAFRDRLIE

QRLASGSSPDRQDLLQNLLDQRTPDGLPAMSIDQIKAETLIVLLAGSDTPASAFRACLLYVMTTPGVYQTLMEEINAAVD

KNLLSSPVVSFDEAKRLPYFSACLRETMRLSPSAPVLLPRLVPEGGTVLCEKYVPAGAEVAANPWIVHRSKEIYGEDAER

YRPERWLEDPAKTKQMDKYDFQFGYGDRSCLGKSIALMELYKGLVQFFRLFEPEIVNPKRPCVYRNMGVAIHTDFWIRLK

KRSVDKA*

>CYP51064A1|664326|Kalpfe1

MHVFIETIRPYGDNVVTATEQKWPRQRKLVSGIFNEKNHRVVWRAAISQAMQMLNEWTALSSSTEKRSSGHVITEMQRDV

RKTALHVISDAGFGVTLPFSPKGVARDDSYDHMTSAEELSLEEEFSDVKPAPGCDLVYRESLAYMSVEYIRAFVTVLLFP

KFLKPFATKTIKHTLNAHRDCGYYLQALIKRARMAKQRGEIEGKENLLASLVTQQEEIKKMLQAEKTEGKAKTMEEITEA

DVMGNTFIIAIAGHETTAGTLAFAFALLAMNQDKQDWVLKNLDEALEGESEDPMNWDYNVVFPKLISPMALMYETLRLYS

PVLAIPKSPIGTAYVHYRGKRCLVPPNTIVELDVPALQYQHKMWGPNSYSFQPEIWDGRRPADPAIEAFSNSVCGGRGHM

FPNVHTPQKGSFIPFSDGNRACIGQKFSQVEFAAVMAVVLRKWRVELVMNDSESREEAVKRVQRILDGSTVKLTLMMKEK

LEIRLVPR*

>CYP567S2|665178|Kalpfe1

FYNLYLHPLAKFPGPKLAAISPLWFSVAVLSGKQADYHKEAHRKYGSVVRVAQNELSFASPAAWKDIHSHHDGKPTFIKA

GFYHQDSKHLTLPHIINEADVAKHAQLRRLHAPAFSEKALKEEETIIDRYINLLVQRLWKRYANRQKGPDGEYCNLVDWY

TYTTFDVVGDLAFGDSAAFACLENGIHPWVSTILDAAFFLGLRDVFRRYPFMVNVGYWLTPKKITNVRARNIEYSRELVK

QRMSKETDRKDFMNYILENKDDPEFSQVDFAANAQILIPAGGETTSTLLSGLTYYIIKTRNVYQRLKEEIRSSFQNYWDI

NSLTTQQLPYLNAVIEEGLRIYPPIPIGLPRVSPGAMVDGHYIPAGATVSVHGYAVTHNPDNFQDPDEFIPERWLPECKF

NDKKDASQPFLLGPRTCIGINLAYLEIRVIIAKLIYCYDFDFVRPDLDWEKESKSWTLWWKPELNVYVKTRDGVQVETLG

LPTA*

>CYP573C1|666738|Kalpfe1

MWLGLIGHIIKNRYFHPLSKFPGPFWASVSNLYCSLMILTGKSQEMEFDYHKKYGPIVRIAPNLLVVSDPRMLPVIYQRK

ADKTDFYITGTLGKGPESVFNVRAHQEHYHVRKRLAAAYGLRATKGMENLVSERIVEWTAQLGRLFADTGNVLDFSMWSQ

FLAYDVITELGFGESMGFVRRATDVGGLIKCFHYALPAIGSLARLPILTKMVVGWDCVTSKPTDKYGLGPVMAFRDRLIE

QRLASGSSPDRQDLLQNLLDQRTPDGLPAMSIDQIKAETLIVLLAGSDTPASAFRACLLYVMTTPGVYQTLMEEINAAVD

KNLLSSPVVSFDEAKRLPYFSACLRETMRLSPSAPVLLPRLVPEGGTVLCEKYVPAGAEVAANPWIVHRSKEIYGEDAER

YRPERWLEDPAKTKEMDKYDFQFGYGDRSCLGKSIALMELYKGLVQFFRLFEPEIVNPKRPCVYRNMGVAIHTDFWIRLK

KRSVDKA*

>CYP584C1|668094|Kalpfe1

MYPILTLALAYLIISFVRRLHYQYGLNKFAAEHGALPVKKFPGRLPFGLSFLIELVGNLRQSRFLKGQHERYERIGNTYE

SNLLGARDIITCEPENIKAILAKQFDEFDLGTRTGKFTPLLGMKGIFVQSQGAGWEHSRALLRPQFHKNQITNDLDSLDF

HTKRFLSLLPSDKVFDIQPLLFKLTLDSATEFLFGESTESLLEGNDENAAGTFAKAFETAQYWVSIKMKTGPINKFIGGK

KFHEACQTTRQYVSRFVDKALSMDLEEKSTEKSKYVFLEALAKETKDKVELTDQILNILLAGRDTTAGLLSMTLWFLARN

RQVWHKLREEVVQRVGANGKPDWELMKDMQYLKNVLNESLRLLPAVPSNGRAARVRTTLPVGGGPDEKSPIVVEKGQRVA

YTVYTLHRRKDIYGPDADEFKPERWDTLKPGWGYLPFNGGPRICLGQQYALIEASFVLIRILQQFKDIKGYDPATGQEGL

TLTLASGNGVHVKLTPA*

>CYP548BD2|682979|Kalpfe1

MLIFYRLYLHPLAEYPGPLLARITSLHAAYHAWVGDSHLVLYHAHRKHGKFVRFTPNFVSVNDAVALNDIYGHGRNVQKS

TFYSVMPPYPHAFDTHTVIDRNLHGRKRRVMSQAFSEAAIRGMEENVLTHIRTFVKRLGSYSCGKKSDDSQPRNMAHWSN

WLTFDVIGDLCFGKTFGMLTREEWRDWPTLIDMAIHRHAIAGISLKIHKLGLGKYLFPKIAEGQKTFVKYSREQAKERIA

LGAGSRKDFFHYLLDAKDPQSGEGYSMSELWSESNLLIAAGSHTPSTALVASFFFLANYPKVLAELTILVRKSFQDLEEI

RGDNFNNPKCQAAYKFVKACLDEAMRMAPPVPTTLPRQVLKGGITVAGRYFPEGVDIATPSFTLHMNETYYSDPFTYNPH

RFLADKAESPEAYQKALSAFAPFSLGPRSCIGRTLAYLELTIALARTVWMYDFDYAGGGRETRLDNLKFVEELGGGKRPI

MYSIDDHHTAFASREGPNIRFIPRLDLQQR*

>CYP617V2|685526|Kalpfe1

MHFLHSLALATALTTYLLHTHLRPFLTFILTFLPSCLQPPSPVCTFLTIFCTTTVLRIIYNVFIYPHIFSPIRSIPGPKS

VSIWNGNYPLMYGKPSGEPHRKWIDELPDEGLIRYLSIFNLERVMPTNAKVLQEVLHTKSYMFEKPATLRNSISQILGAS

GMLLSEGEQHKIQRRHMLPAFSYRHLRELIPTFWEKSLELVEAMGKEVHLSQSSDAGKQTGVIMEVSRWLNRGTLDIIGS

TGFGYEFNCVKNDHGELVRAYRKVFAPETNGQQLVRIVTQYLPLWMVNLVPSKRRREAQEAIAMVKGLCMRMVKEKKREY

ENGGKVGEKDLLSLITKDSPFDEEEMRDQLMTFLAAGHETTASALTWALYLLSQHPEVQSRLRAEIRSHIPSPMTAGSNF

TTMQFLQESLDTLPYLRNVTLEVLRFFPPVPATMRVAAEDTTLGGHYIPKGTVVMLCPWAINRSKKIWGSTADVFDPERW

EHHKEGKNGASNYDFMTFLHGPRSCIGKDFSRLEFKALLIALVGRFEFSEVLDEMGRRKEISIKGGITSKPESGMHLWVR

AVDGW*

>CYP6900A2|693368|Kalpfe1

MALINLLATGASERSPSFFDCLFILTVALPAYHLVIVPIYNSINSRLSTLPGPKLTLLTPLYITALDALLLRSKTLHKWH

QRYGPVVRVGPNEVSFISPTAVKQIYQDPTMEKDTRLYGQFTHFGANNAFSSRTRYEHGWRRKGVAALLSWSKILESEVA

DRWIGQVIGRYLKAIHDSALYKSRLISQDGMKLVDVYGLNSLFATDVVSGFIFGPVWGTKTLCFEDIKNEAGNMWWQKPS

YHRRMVKEHYSSSLRAQTYLYVEFPRTMHLVLAIVRIFKSCRRNILAFAGFGEGTAKREKEIICETVEVWGWKVWTEVLN

GKKGHQTGKYVAEVLASYVNGEGNTRPQSDEQWTSEIAASELMDQILAGMDTTSDTLSFLMYHLSLPSSQHIQQRLHLEL

ITAFPPSSPHTSVPTFFIHGQNNLTSDSNSKYMEFIIKLNALPYLDAVIKETLRVYPAIPVTLPRVVPDISGQGKVIDGY

YMPPGTVVGAFAYGIHRDKSVFGEDAYEPENWITDSQNYEKASRMEKHWLGIQKETENPGKCEGRKEDIKIHQVKGMEKR

LWAFGSGGRICVGRHLAMLEMKMLVAAVFWNYKTQVVDDLTAASCVKLDHSYWNERKTFRDVLPFKGVNGLVSFLPRKYG

SEVAGPEASS*

>CYP65Z5|695460|Kalpfe1

MLSQLGPLFWFDSLEGIKTKEELMSTLLIGLASLIVGSIAWRCFYNLYLHPLAKFPGPKLAAISPLWYSAASLSGNQIWH

YREAHRKYGSVVRIAPNDLSFSSAAAWKDIHGHHPHQSTFVKTEFYNSDPKNFGTPHILNEKDIAKHAQIRRLLAHAFSA

KALQEQEALIDRYINLFVQRLGKRYADRQKGPDGEYCNLVRWYNYTTFDIIGDLAFGDSGAFACLENERSHPWVSAILGG

IYASGLRDIFRRYPFMINIGYLLTPKRITNMRVHINQYGKDMVEKRLSKETDRKDFMTYILDKKDDPGFSDIHLPGNAHI

LIIAGSETTATQLSGLTYYLLKTPDAYQRLKDEIRTSFRSYWDINSLTTQHLPYLNAVIEEGLRIYPPVPIGMPRKSPGA

MVAGYYVPAGAEVGVHAYSTSHNPDNFQDPDQFIPERWLAGSKFNDKKEASQPFLLGPRGCLGRNLAYMELRMIIAKMFY

CYDLELVRPDMDWEKESKSWTLWWKPELNVYVKTREGIEVETLGLPTA*

>CYP5142C1|698358|Kalpfe1

MQPVMAYQSLVTTTVNQYLALARTYPLPLVTAVVVSCLYILIQLFLIAYRLSPFHPLARFPGPKVARATGWYRTYHDIWR

GGNMLDELKRLHKIYGPVVRFGPNDLHFSAPNAYLAIYSSSSKLTKDPWLYKSFSEDQSSFGYIDPVLAKQRRDRIAPFF

ARRNILNLQGVIIGKIDLLCEQLASAAGSSRVVDLGSAFRSLTVDVIMSFCFAECFDALRHPEFRHPVIRLLETSIPMCW

TFKHFPIVRMLVMNTPPWVSGLLGSDSRGIMMLKGLIDRQLDSFLKNPYAEDHAHPVIYHRLLEPDAKHGQGIPSRRSLF

DEAQVLYVAGSDTVGTTLSAGSYHILSNPHVYTALREELQEAWPNVNLEDKLENGRPGWEALEKLPYLTAVIKESLRMSH

GTVSPLSRIVPPQGVSIGDEFVPGGTVIASSAPFVHHNAAIFPDPYEFKPERWLGTSGKDLDKWLVAFSKGPRMCAGINL

AWCELYLAFATLYRKFDFELWETTAEDVKFRDFFVPSFEGTITARVKVRED*

>CYP567Z1|699064|Kalpfe1

MLSQLGPLFWFDRLVGIKTKEELMSTLLLGLASLIVGSIAWRCFYNLYLHPLAKFPGPKLAAISPLWYSAVSLSGNQIWH

YREAHRKYGPVVRIAPNDLSFSSAAAWKDIHGHHARQSTFVKTEFYNTDPKNFGTSHILSEKDIAKHAEIRRLLAHAFSA

KALQEQEVLIDRYINLFVQRLGKRYADRQKGPDGEYCNLVHWYNYTTFDIIGDLAFGDSGAFACLENERSHPWVSGILGG

IYATGLRDIFRRYPFMINIGYLLTPKRITNMRLRINQYSKDMVEKRLSKETDRKDFMTYILDKKDDPGFSDIHLPGNAQI

LVIAGSETTATQLSGLTYYLLKTPDAYQRLKDEIRISFRSYWDINSLTTQHLPYLNAVIEEGLRIYPPVPIGMPRKSPGA

MVAGYYVPAGAEVGVHAYSTSHNPDNFQDPDQFIPERWLAGSKFNDKKEASQPFLLGPRGCLGRNLAYMELRMIIAKMFY

CYDLELVRPDMDWEKESKSWTLWWKPELNVYVKSREGIEVETRGLPTA*

>CYP6433A1|700758|Kalpfe1

MNPLKLTSFHTPWVNVQRSMQIAHRGLKRSFDPEADDGIFLNNDLIGLLHDIPLQIIVISLFVLCVIARKVYIQWKRQVK

LPYVTVGGGPHWIRSWRTAIHWVANSKSLIDQGYYEHRHKTKIGAYQIPTIGGTFVVICAKDLIEEFCNAPSNKLSFAHA

TSDLLQAEFTIGTDILFNPYHVPIVREQLTRQIDAFSLPLMEEFAFAMEYYMDLNGGEWKTFKAYELFVDILAQGMSRVF

VGPELCRNPEYLKLAIGFTQEVFVNAHIIHLVPKPVRYILSPLLNAFTINKSLRAAIRLLGPMIEERKRRANEEGRNWKG

RPEDLLQWVIEGAPPGEQNLKAHVKRLMELNTAGLHTTVNTLYQALFWLVMYPEYIPELRQEMESVIGEFGWNKASLSKM

LKLDSFMKESHRLHGPSVATMGRKVLGDGFTFSNGLYLPPGVTAGVPDTIHRDEVEYGPNAKEFDGFRFSRPVENAGVNG

EKEESGSSRVKRKYFVTTSLNYLRFGHGHGSCPGRFYAAHELKILICLFLMNYDLRLEDVPKPIYFLIHRVADMSARISI

RRRKTASF*

>CYP51F1|709484|Kalpfe1

MGLLSSQLPPLEVIIAHLTPQTITYMVIIGLVDFFVVATIFNIVQQLFFADPNKPPVVFHYFPFFGSTVIYGIDPYKFFF

SCQEKYGDTFTFILLGKKMTVYLGPKGNEFIFNGKLSQVSAEEAYTHLTTPVFGTDVVYDCPNHKLMDQKRFMKFGLTSE

TFRAYVPLIVEELENFVRSNPLLSAPKGPVDLLAIIPELTIFTASRTLQGKEIRKGFTGEIAKLYHDLDMGFTPLNFMFP

WFPFPQNKRRDAAQRKMAQIYMDIIKRRRGGEETGEQDMIWNLMNQKYKNGDLVPDKEIAHMMIALLMAGQHTSMATTSW

ILLHLASRPDVVQDLYEEQQRVFGKDLGPLTYESLSELKLHNYVIKETLRLHPPLHSIMRKVKSPMQVPNTHWVIPDTHY

LMAAPGVSAVDPKYFKNPTAYDPYRWATEKDQEEEEEKFDFGYGLVSKGTSSPYLPFGAGRHRCIGEQFANVQLGTLVAM

FVRLFKMRLPDGQTKPAPPDYTSLIAMPSRPATIMYEWRNPDWRNSEKH*

>CYP50042B1|716771|Kalpfe1

MAVLSVLVGFTPLQLAGIAISLWIVSYLIAYLKRDSNPSKLPVVGVPPGPLGRIRAIPASLWRLKEMVNTGYHQYHKQKN

PTAFLAPYFFFDYICVIPPSLVGEVKSAPEHVLSQNEAFEDASGGLYSFGDSGLSLNLYHVVIVRQKLTATIGKILGVLQ

DEIVAAFTEEWDVSWGERAKKADFIADEKGDAGGWVDVDVLDDTRNIVARVSNRAFSGLEICRDKAFLENAVDYSVSIVF

CGWGIQLVPKSLRWLFGPIIAYRNQRRVKFAQKKLAPLIAKRVAIREHNVMAKEEDQIDEPEDVLQFIIQRALEVGHHDK

EITVRYLILNFAAIHTTSLTLSSFLLNMATCGSTDSSGHRCWDLLREEVLAVDRDSEEAPGVWTKQKLNKLVGMDSYLRE

TIRWNPLVGLLSLVRKVMPKEGYTFSNGLWLKQGDYVGAPAYNIHYDDELSGEDASAFGGFRFSKPYQQLRAEGKSIAEI

AALGVGKFAAVTTGDNYLSFGHGKHACPGRFFAISEIKLILIHILLNYEVKEIDKWPNPIFLWHSELPPIGLKLKMRRRR

TE*

>CYP6902A1|717064|Kalpfe1

MLILFQALIGLFVGYSIYHFYCLYCNIRIARTSGLPYIVIPIFEYGIFWMVFCPLLHPLINRCLPSFLTKSWEPFLAPDW

QWRMKHSACKLLKSDVFLVVSPTAIIMSVADADMINEIITRREDFPKPTKAYRALDIFGKNVVTTTGKIWRVNRKITAPS

FNEKNNQLVWTETVEKTKALLDHWVTPIAPSMEPPDIRTLSDDCMRLAFHVISWAGFRLKLTWPKRVSGKSFDELPSPDK

TIEQGHSMSFQSALHYVLVWIFLLVGAPKLYMQSTPYKPHRTAWKAFKEWGVYMNEMIAERKRELENGMVETGDIMGALV

GAYYSGVEEKGEKEKPMITKQEVIGNTFIMMLAGHETTARTLHYTIMMLAVHPELQKPLQEEISRILGDREPDYERDFQP

LASGWCGAIMNETLRVFCPVVVTIVYNKKKCIIPADTVIHINNHAVHRNPKYWVLPNQTEEEAQINEYRPQRWFLNNNND
NSNSSSSTSITENYDEEDDTNPAYEPQGKDTSKSFFRPHRGSYMPFSDGPRACLGRKFAQVEFVAALAVLFREYCVELVV
RKGETWEEARGRARRYVDNSGAVITLGPKGEEVGVRFVRRV*

>CYP6433A1|726533|Kalpfe1

MHPLKLTSFHTPWVNVLRSMQTAHRGLKRSFDPEADDGIFLNDDLIGLLHDIPLHVIVISLFVLCVIASKVYIEWKRQVK
LPYVTVGGGPHWIRSWRTAIHWVANSKSLIDQGYYEHRHKTKIGAYQIPTIGGTYVVICAKDLIEEFRNAPSNKLSFAHA
TSDILQAEFTLGTNILFNPYHVPIVREQLTRQMDAFSVPLMEEFAFAMEYYMDLNGGEWKTFKAYELCADILAQGMSRVF
VGPELCRNPEYIKLAIGFTQEVFVNAHILHLVPTPMRYILSPLLNAFTINKSLRAAIRLLGPMIEERKRRANEEGRNWKG
RPEDLLQWVIEGAPPGEQNLKAHVKRLMELNMAGLHTTVNTLYQALFWLVMYPEYIPELRQEMESVIGEFGWNKASFSKM
LKLDSFMKESHRLHGPSVAIMGRKVLGDGFTFSNGLYLPPGVTVGVPETIHRDEVEYGPNAKEFDGFRFSRPVEDAAAGV
NGEKEESGSSRVKRKYFVTTSLNYLRFGHGHGSCPGRFYAAHELKILICLFLMNYDLRLEDVPKPIYFLIHRVPDMSARI
SIRRRKTASF*

>CYP6001C34|728085|Kalpfe1

MEKNDDQIAATNGHSNDRTNGQTNGHTNGQTNGHSNGHSNGQSNGHSKGKGAATAEVPTSTQVSKYKGSKAKPTRKDVDQ
TMGAITNLLHASNKPLPHRYGDGKQRKSIYEQKYTGIWADIKYLSKEGNLKESYKTLRTLAKHKKRPGYTDDKTYTMEYV
IRLSAHLPTDSLHRLKLTALQVDQLWNTLQHPPMSLLSNEYRFRQPDGGHNNPMYPNLGRAGTSYARTVKPMTMMPMSQP
DPGLVFDAVMCRREFRPHRNNVSSMLFYIASIIIHDLFQTNRKDVTINDTSSYLELSPLYGNNAEDQRKMRTFKDGKLKP
DCYSEKRLLGFPPGVGVILIMFNRFHNYAAEQLAAINENGRFDPKIDHRNRNREEALAQAEAKREEELFQTARLVTCGLY
INIILTDYLRTIVNLNRTNSTWTLDPRFDPSKIYNPHGTEQGTGNQVSVEFNLVYRWHSCISKRDEIWTEDLYKQIFGEN
VDPLNMPLSEFLKGLGQWESSLPDDPAQRTFSNMQRNDKGMFEDDDLVRILTEGIEDPCGAFGARNVPAVLRAVEIMGME
QSRKWKVASLNEFRAFFGLEPHKTFESINSNPEVADALRQLYDQPDFVELYPGLVAEEDKVPMEPGVGIAPTFTISRAIL
SDAVVLVRGDRFHTIDYTTAGLTNWGFQEVASDFNVLHGCVFYKLFLKCFPNHFTWNSVYAMYPLTIPEENRKIFTNLGT
VDNFDYSRPKYVKPRVPIRSYAATKRILDDKQTFNMMWEGFTWVFGKPYMLSGDGEWEADLRKFIGKCIYGQQDWEKQVR
QFYEDTLTRLVKEKRYKVPAPGCQGEGSWQIDAVRDVNNLAHANFAATLFGLPLKTKDNPKDFFTEQEIYQILTLVFVCI
FFDVDPSKSYPLRHATQQLARQFGKLVEIEVKAVDSIFGALVSGKYFKKRNTSALRNYGIHMIKRLLESGKSAEDVTWSH
ILPTAGAACANQGQVFAQVLDFYLRPENAQHLAEIQRLARLDTPEADNLILRYALEGTRLAGTFGLYRRVNCAGDTITVQ
DDDRTVHLDHGDRVFVSFISASTDSTIFPDPHAIRLDRPEESYIQYGSGPHQCLGMNINKVSFPTMLKFFGKLKNLRRAP
GIQGELKAVEREGKFKVYLREDWSGFWPFPVNMKVRFDGVEGWEGE*

>CYP6453A4|732447|Kalpfe1

MALFSAFEAAFAGNNKADVRSYYYLNSFLFSVRELWSRMFILSALILLYPIGLILYRLYFHPLARFPGPKLAAITSWYEV
YWNVVKDGQACYKRKQWHEQYGPVIRINPNEIHVTDPSTYNTIYLRSHPLFLKYHPFYRIFLADSASVGIMNPQVHRVRR
EQLTLLFSRNNVVEMEGIVWDTVNSLCKKVKEYALDSQNDIPAKKLGMKTLFKMVTSDVISAFCYGMSFSSLPAAQMTCN
RSGVVMATPPRFLNSMARAAEAFWFFQRFWRLQLLLVNLPDWFVDSFNLEAFLGMRDIMSECMSHIRDIQNKTETERKTL
FTQLLSRPESQSSPHSKEKQYTAPTTEQLLEEAITVMSAGVEEASNAMMYGLHKVCKNKKIYNNTKRELRRIWPNKDENF
SILCLEKSPYFMGVIKEILRMSIPVPGKLPRIVPSGGIVVDGWHIPSGSVISMSAIMQNFHPAIFENPDEFIPERWYNSD
GTPRRDLDKYLVTFSTGSRMCLGLHLATAEIYITIATLLRRFDLEVVDKDTDMRWVDRIAAQFLGDLVVRVVAEEKD*

>CYP61A1|744067|Kalpfe1

MDSQTSPSAVPGDVSAQKLLRTGVFEQLFAGVSYWSFAVTLLLLCVTYDQVRYQWNKYGIVGPAFKLPFMGPFLESINPK
FEEYAAKWASGPLSCVSVFHKFVVIASTRDLARKVLNSPTYVKPCVVDIAKKILRPTNWVFLDGKAHVNYRRGLNALFTR
KALSEYLPGQEDMHKRYFKRWIEISKATPGIAFMPEFREINCAVSCRTFVGDYTTDEKVKKISDDYYKITAALDLVNFPI
IIPYTRTWYGKKCADMVLDEFSQCAEKSKENMAAGGTPHCLLDHWVKAMYEYRDYQERLDRGEALAPGEKAPVATPMFTN
YEIAQTLFTFLFASQDASSSATTWLFQLLADRPEVLNRVREEALRVREGDPYKPINVDMLDKMVYTRAVVKETLRYRPPV

TMVPYEVKKDFPVVDNYVVPKGSMIVPTLYPALHDPEVYVNPETFNPDRWLEGGEAEAAVKNWLVFGTGPHYCLGQTYAI
YNFMSMISLASLMMDWEHHVTPKSEEIKVFATIFPMDDCLLTFKGRLPILD*
>CYP5192E1|744105|Kalpfe1
MAAMQLNPVYVAIAVIFAGCIVRFFIKAYELRKFFRDKPGPPHSWVWGHLPVFSTIFQHVPRDSHPHSYAYHVSTIYNLP
RFYYMDLWPVFERNLVITDPAIAQQVTVTKSLKKHPILKKFIRPIAGKENILSADGEQWRRWRAVFNPGFSAAHLMTLVP
MIVEHTETFVEVLGGMADKGVIFRLEEAATRYTVDIIGRVVLNAKLNAQVSDHPLLTAFRKQINWLPRAQATQLSWYNPI
RMYHLWKNGRAMDQYLEAELDKRFESQDMEASKDKKARKRYIIDLALQTYLEEEGGKKGEGLDNEFKRNAISQIKSFIFA
GHDTTSSTIAYACHILSKHPSVVAKLCSEHDALFGPDVSGTADLLREDPYLINKLSYTTAVIKETLRLFPPASSVRVGEE
GVEIVDPLTGEKYPTKGCLVWVTLEAMHRLPCLWERADEFVPERWLSQESTPPAQEKGLPPQPPANAWRPFEKGPRGCMG
QELAMIEMKVALAMTVRRFKFQAVYKEKGWRDKVVGWDGVRREGRGDNVGRLGKGNEEEGGKGVKENHGEEAYQVLLGTA
KPKEGLPVRVWRVR*
>CYP6433A1|747534|Kalpfe1
MHPLQLTSFHTPWVNVLRSMQTAHRGLKRSFDPEADDGIFLNDDLIGLLHDIPLQVIIISLFVLCVIASKVYIQWRRQVK
LPYVTVGGGPHWIRSWRTSIHWVANSKSLIDQGYYEHRHKTKIGAYQIPTIGGTYVVICAKDLIEEFRNAPSNKLSFAHA
TSDILQAEFTLGTNILFNPYHVPIVREQLTRQMDAFSVPLMEEFAFAMEYYMDLNGGEWKTFKAYELCADILAQGMSRVF
VGPELCRNPEYLKLAIGFTQEVFVNAHILHLVPTPMRYILSPLLNAFTINKSLRAAIRLLGPMIEERKRRANEEGRNWKG
RPEDLLQWVIEGAPPGEQNLKAHVKRLMELNMAGLHTTVNTLYQALFWLVMYPEYIPELRQEMESVIGEFGWNKASFSKM
LKLDSFMKESHRLHGPSVAIMGRKVLGDGFTFSNGLYLPPGVTVGVPETIHRDEVEYGPNAKEFDGFRFSRPVEDAAAGV
NGEKEESGSSRVKRKYFVTTSLNYLRFGHGHGSCPGRFYAAHELKILICLFLMNYDLRLEDVPKPIYFLIHRVPDMSARI
SIRRRKTASF*
>CYP567Z2|760196|Kalpfe1
MLSLIGPLFGLNGLGTIETKWELVSSLLVGLLWLIVGSIAWRCFYNLYLHPLAKFPGPKLAAISPLWFSVAVLSGKQAEY
HKEVHRKYGSIVRVAQNELSFTSPAAWKDIHSHHAGQATFIKAGFYHQDTKHLSRPNIVNEANIEKHAQIRRLLRHAFSA
KALQEQETIIDRYINLFVQRLWKRYADRQKGPDGEYCNLVDWYTYTTFDTVGDLAFGDSAAFASLENGSIYYLGLRDVFR
RYPFMINLGYWLTPKKLTNVRARTLEYSGEMVKQRMSKETDRRDFMSYILDKKDDPDFSHVDFAANAQILIAAGSETTST
LLSGLTYYLIKTPNVCQRLKEEIRSSFQNYWDINSLTTQHLPYLNAVIEEGLRIYPPVPIGLPRVSPGAMVDGHYIPAGV
TVSVHGYAATHNSDNFKDPDEFIPERWLPECKFNDKKEASQPFLLGPRSCIGMNLAYMEIRVIVAKLIYCYDLELVRPDL
DWDKESRSWTLWWKPDLNVYVKTREGIQVETLGLPTA*
>CYP539A47|767423|Kalpfe1
MLLQSLVTSAPLILLAVTFSSLLLVVLEKTQEYFKMRQLGKRPRSLASWFPWLGFDIIYASVQAAKNNESYEGWKKKTDA
FGYTFEMTLLLQRMVFSVEPENVKAILTSQFYDFGKGELFHKMWYPFLGDSIFATDGEKWHNSRQLIRPQFIKDRVSDLH
IFERHSQKMVSIIRNVGGRTIDIQDLFFRLTLDTATDFLLGQSVNSLESPQVGFAEAFANIQQIHNTMERLGPLHHFFPK
GTYKKDLATLNGFVYPFVEKVIRMAPEELESKGGHNYNFLHALAGFTKDQKVLRDQIVAVLLAGRDTTAVTLSWALYELS
RQPHYVQRLRQEILETVGPTDAPTYEHLKNMRFLQHVLNETLRLYPAVPFNIRTALKDTTFPRGGGPNGRDPVAIPKGTP
VAYSAMIMQRRVDLFGPNANEFRPERWDGPAPKAWHYIPFNGGPRICIGQQFALTEMGYFLVRLFQTFEEVHYMDTEPQK
ARIEITISCAGGVNVQFREAGIV*
>CYP567Z3|772102|Kalpfe1
MLSLTGGKCLSGPLIVGSIIWKCYYNLYLHPLAKFPGPKLAAISPLWHSWVSLSGRSIWYYQEAHKTYGPIIRVAPNELS
FSSAAAWKDIHGHRPRVNIISEKDIVRHAEVRRLLAHAFSAKALQEQEELIHKHIDLFVQRLGKRYADKEKGPDGEHCNL
VHWYNFTTFDVIGDLAFGDAAAFGCLENAKPHPFVTMILNSIYVVSLANIFRRYPFMLKLGYFFTPKKLLDGRIQNMQYS
EALVKGRMNMDTDRKDFMAYILDKKDSFPHIDIPSNAEVLVIAGSETTATLLSGLTYYLFKTPEAYQRLTEEIRTSFQNY
SDIDSISTQHLPYLNAVIEEGLRIYPPVPVGLPRISPGAMVAGHFIPPGTEVSVHAYSASHNPDNFAEPDRFLPERWLQE

GKFNDRKEASQPFLLGTRGCLGRNLAYMELRLILAKMLYCYDLEWVNTDLDWEEKSRTWVLWTKPDLKVHIKSREGMRWD

ELGLPDHDA*

>CYP5093H5|790361|Kalpfe1

MDYQRSLKMPPGPIPIPILGNKWELPLRKPWYKLKQWTDRYGSIVTVWTGRRPTVVIGDPKVASDLLDKRSSIYSSRPRF

VVMGELFTNNDSLLTMPYGERWRATKKIFHLGLCKKACDSYKPIQEAESRRLARDLLKTPAVFGKHLERYAASVMFCVAY

GRRVDELNDPLVTKIYERMGYMATLNVPGAFWAESYPILKLVPDCLAPWKREVKRRGEESTKLLSTLATAVRDQMRKTIA

PPSFTKTLWEKHIAEPGSMTEREIAYATGSLFGAGSDTTAATLMSFILAMTCFPHVAKKAQEEIDRVVPKNRSPTWEDMA

QLPYCNAVIKETLRWRPVAVMGGTPHATTQDDYYNGLLIPKGTTIMGNLWAIHHNEKYFSDSHTFIPERYLEEPPAGMEK

YPNRDGHSAFGWGRRICPGKLLAENSLYITITRILWGFTISKAKDDFGNELTPNIFAYTDGFNSKPQPFSCEVKVRNAAI

EAIIDNEAKTAENYLDRYLYE*

>CYP548BE5|791692|Kalpfe1

MDGFTSSLPTQLASTHIGHFLSHPLSVRLPSPSNISITLLYTATALLTALVSVCLYRVYFHPLSRYPGPLLSKITPLHAT

WHAYKGDRHLFLHRLHMKYGPVVRWAPNAVSINTATALKEVYGHGLNARNVRKSDFYKAFPAVKGVHNTHNSIDKAEHAR

KRRVLSQAFSENALKGLEGLMLKNINVFFEKLDERMAKGKVGEDLYAGKGGRKGLDMGEMFSWLTFDVLGELCFGKAFGM

LVDSSQRFVERLIDNAAHNHHICGHYLPLSTLGLSKLLFPTIAGERWRFILHSRACANERMELHKSGQDADKRDFFHYLL

QAVDPETGKGFETVELWGEANVLMIAGSDTTSTALAATLYYLTKNPEKLKKIQEEVRGKFDRIEDIVSGKELTDCVYLRA

CIEEGLRMCPPVPGLLPREVIAPAGLTIDCSKDGPVEKGFGKYYFPQGTVLGVNIYSIHHHPEYYPEPYKYTPERWLLPH

EDPRGEGQGTEESTEKARAAFTPFSIGSRGCIGKSLAMMEMRLVLGRMMWGWDVEGVEGVEGKSECWRGGYRWEGEGGIE

EEYRIFDHFTARKEGPVIEFKRRIVE*

>CYP567Z3|813990|Kalpfe1

GPIIRVAPNELSFSSAAAWKDIHGHRPRGPPFVKSDFYFTGDTYNRVNIITEKDIVRHAEVRRLLAHAFSAKALQEQEEL

IHKHIDLFVQRLGKRYADKEKGPDGEHCNLVHWYNFTTFDVIGDLAFGDTAAFGCLENEKPHPFVTMILSSPYVLSLANI

FRRYPFMLKLGYFFTPKNFSMVGFKIFSIVKHLSKGIRMSMDTNRKDFMAYILDKKDSFPHIDIPSNAELLVIAGSETTA

TLLSGLTYYLFKMPEAYQRLTEEIRTSFQNYSDIDSISTQHLPYLNAVIEEGLRIYPPVPVGLPRISPGAMVAGHFIPPG

TEVSVHAYSASHNPDNFAEPDRFLPERWLQEGKFNDRKEASQPFLLGTRGCLGRNLAYMELRLILAKMLYCYDLEWVNTD

LDWEEKSRVWILWMKPDLKVHIKSREGMRWDELGLPDDAA*

>CYP567S1|829689|Kalpfe1

IAWRCFYNLYLHPLAKFPGPKLAAISPLWFSVAALSGKQAEYYKEVHRKYGSVVRVAQNDLSFTSPAAWKDIHSHHAGQP

TFIKAGFYYQDPKHLTLPNIINEANVAKHAQVRRLHAPAFSAKALKEQETIIDRYINLLVQRLWKRYADRQKGPDGEYCN

FVDWYNYTTFDAIGDLAFGDSTAFACLENERSHPWVSTLLDSIFFLGSRDVFRCYPFMVNVGNWLTPKKITNVRARSIKY

SREMVKQCMSKETDRKDFMNYMVQCTNDPEFSQVDFAANAQVLIAAGAETTSTLLSGVTYYIMKTRNVYQRLKEEIRSSF

QNYWDINSLTTQQLPYLNTVIEEGLRIYPPVPIGLPRVSPGAVVDGHYIPAGVTVSVHGYAVTHNPDNFKDPDTFIPERW

LPECKFNDKKEASQPFLLGPRTCIGTNVAYLEIRVILAKLIYCYDFDFVRPDLD*

>CYP617V1|848719|Kalpfe1

MHFLHSLALATALTTYLLHTHLRPYVTLSVLQPTSAVCTFLTIFCTTAVFRILYNVLLYPHIFSPIRGIPGPKPLTIWNG

SYRLIYSNPTGEPQLKWLDEFSDEGLIRYLDFFNVERVMPTNTRVLQEVLVTKSYVFHKPELVQNTIAQILGATGILFSE

GEQHKIQRRHMLPAFSYRHLRELIPTFWEKSLELIEAMGDKVLTNQGSDAEKQSGVIIEVSCWLSRVTLDIIGSTGFGYE

FNCVRNNYNELITAYRNVFEPVTKDQKLIACVMLYLPLWMVNLIPSKQRRQAQEAIAMVKGLCMRMVREKKREYETGGKV

GEKDLLSLITRGSPFNEKEMHDQLMTFLFAGHETTATALTWALYLLSQHPEVQSRLRAEIRDHIPSPMKPGSKISTMQLL

QESLDTLPYLQNVTLEVLRIFPPVPMTSRVAAEDTSLGGYYIPKGTMVVLCPWAVNRSKKIWGPTADVFDPDRWKQRKEG

KEGVSNYDFMTFLHGPRSCMGKDFSRLEFKALLIALVGRFEFSEVLDEMGRRKEIVTRGVVTSKPESGLHLWVRSVDGW*

Morchella importuna CCBAS932 v1.0

>CYP6775A1|605753|Morco1

MTFLSTLIALPPIPAISTVCAIYITSICIYRLYLHPLSRIPGPKLAALTLLYEAWYNLFAQDGRFIFHTENLHKKYGPIV

RIAPNEVSISDPDFYNKVHYPGSQFEKDPVTYEAFGFGDSIFSTPTNELHAKRRAPIKDYFSRRSILAIEPVLLNTIKKF

LGRIDAAIAMQPERLFDIQNGLRATSVDMSTTFSFNFPLGMLDMEDLGKRFSEDGQRRLKGFWVVKYFPWLPTVLSVLPK

SVEKWMLPAIQSLKDLTMMSRGSFDSIKASGETGTSIFHHLLSPENMKRDRLSDDDIIQNGVVCFAAGAETVSYTLIMTA

FGIVSNPDVEETLYRELREAFPDPNELCLSKLEKLPYLVAVVKEGLRLAPGAFGYTPRVTPKGGIKYNEYTLPHESVIST

SAFYLHYNPTIFTNPEKFDPERWLSPDSKNLEKYLVPFGRGQRMCVGMSLAFCELYLVLGSIFRRYQMNLHNTSWEDVKQ

GMDYFSGFPAKGSRDFTVKAQKRGD*

>CYP50357A1|192534|Morco1

MYLEILPENLTCTQIHTHNHSTKNIIISSALSSKMHVPEFHPVTTIFVILFALSFSIISISPTLLFLCAKGRKAKDIKNK

TFIYQSLKQAKVILNGSSDGKFRRLEDISTILKSRAEPNSRLIRAFGIDNAFTTSDPQHHRDFVNRASDLLNLSNSQWKS

LAKHIDTLVKGNVERQRVSSFDGHSRTVIFSQLLQITVLRAVLSVLFPNGGTPQLSEEEQDSIAYEVAEKITSLWIESKS

TEITSNPRDLESLMEACQKLLPGIDTRVENPLNLILPAFETLWRIVLRGFLEVQFRDKENFSLNKSLFSEYLTSPTSTVF

MREDTRGVSVSHIINESLRLYPPTKRVYRKIQTESTQPDVAIDIEQIHRDPETWGSDANIFRPSRWRKSEMNKTIAAAFI

PFGMMPLICPARKIFGPRIIGIFLAALLGAINEFGELDVVGAMVEGEVLLENGRNSYGEMGMRSRK*

>CYP6521B1|294597|Morco1

MITIYWSFLLGLAASILVYRIWLHPVRNMPGPFWAKTSKWWVVYQQWRTGLRFHQYSQKLHKRYGDILRIGPNDLSIIDP

DIIPMLGKAKEFGARVTLKSVFIRYETGKPTQYAGGSSWDRDFTVKALQGYVPQMAKYSDQLMSNISKSEGAPMNISNLF

MFLTFDVMGDLAFGKPFGILETEGLDGADSFIKTMHGFTRVVGSVSHVPWFMFLLQWLPGSLSGEFDNFERWCGHMVEKR

KQFKPEKPDIFHFLLEAEPQNIGEHHIDLIGDAQLASVAGSDTTANTFINIFTQLASDPHVLQKLQHEIDAIYNAQYDTD

KGSVVPEVILNGNSATTRYLDSVINEGLRLNPAVPGGVQRVTPEEGVTINGIMGFHK*

>CYP548BE7|478657|Morco1

MSILSSLSILLSSLVVYLASLVFYRLYLHPLAKHPGPFLARITDWYNVYHAYLGDRHLALHNAHLKYGPVVRFAPNLISI

NTSTALKTIYGHSPASRSLQKGSFYSAFPAVKGVYNTHNCINKTEHGRKRRVLSAAFSENALKSMEDLVLGNIQVFVDEI

ERRSVQLRKGIDMGEMFSWLTFDVMGELCFGKSFGMLRDETTRFVTHLIAQAAHSHYINGNYLPLATLKISRILFPTIQR

DRWRFIQHSRGCANERMKLGADYKKDFFHYLLDAKDPETGKGFETAELWGEANVLMIAGSDTTATALAATLFYLLRNPTK

LATLQKEIRSAFASADEIVGGKQLTDCHYLKACIDEAMRLAPPVPGLLPREVTAPEGITVDGVFLPCGTVTGTPIYALHH

NPTYFPSPHAFDPERWLQPYTTADAIESARAAFTPFSIGPRGCIGKSVAYVELRLTVARLMWEFDVEEVVQEGKGVLWRE

GVAVREGEFRLLDHFTSRKEGPVVVFQRR*

>CYP532S1|481334|Morco1

MAVTSIILSYPIFTPGVLAIIGLFIICLYCLKWRYRSGLRSIPGPFLASITNLYRFFDAWSWKCQDNQLALHEKYGKFVR

YGPNLVSISDPEAIQVIYTINKGFVKSPLYKVFFVLSNGKTIQSMFDTQDELYHQSMRKPISHAFAMSHLMDYEPYVDST

SKFFTSRLTELYANTGDICDLGEWLQYYAFDVIGELTYSKRLGFLDRAKDVDGIMRSIDKSFDYAGVVGQMPWLDTLLEK

NPIWNYFNPPTSAVAAFTLDCMAERMREMEKGNLRKQDMLARMIEAHKADPEKVTPAHILGWASSNTYAGSDTTAISLRA

LFYHLLKNPGSMEKLLSEISSFEGLSNPVSWTEARKMPYLEACIKEALRIHPAPSMLLERVVPKGGKDICGRFFGEGTIV

GINPWVVQRDKDVYGADAELWRPERWLEAGEISRKRMDRTAFAFGGGSRACIGKNISYLEMYKVIPQLLRTFHIELAYPE

KEWTLHNNWIHKQSGLLVKLTQKGF*

>CYP6188A6|483725|Morco1

MHRIRNDTPLRDIPGPWLASCSPLWRFWYAVCKSNYHHDLTNLHRKYGNIVRIAPNEVSVWDPEYVSEIYSHGENVYPKC

DMYDIALPNGFFNLAVERDIKTHTLGRRAIAKDYSMTAILKSEKYFDAVIKDFITALDKNFAQTGKKCNFTVWSEYFTYD

MITDIVFGKSFGFCNAARDVEGGLRDLRQMLNLSPFLSYLPWIWPLTQLEIIKKTGMKHYARCMVREIQKRVREGNPTGR

QDLLQGLMDARYQDGHALPLGEITNHAYIFVLAAPDTASVALRNIIINLCRNRDVYQQLMDSLLALNTANPTWKDLQNIP
LLTAVVKETLRLHPPAGFSLPRAVPAGGRTVCGKFLPENTTIGMSAWSVHANTEYWGEDTLEFKPERWMDPNQMYKLERH
SLIFGQGSRQCLGKHIAMINLVKVTAQILLNFDFGLLNEKNIKEVFLLLVVVDGMDVWFKRRAGGPLDDTIGSESEKPAA
VTTGA*
>CYP6271A6|486944|Morco1
MDTHIIIKQYAEWASIAKTWLLSLSKLEAFIILTTLWTVYRIGVYTYRLFLHPLSKYPGPRLAAASSVYEMYHDIVKKGD
MTFHMDELHRKYGPIVRISPNKVRLRDSSAFHELHKVGSPLIKDPGFYSLFGLANSTFSTINPIIHRKQRRILDPMFSRR
SILGFENVVQEKCDLLCYKIRELEQADSKVSFHNAFVALTIDIVTEYAYAKSYNTLLNEGFVSKVSSAFDAQQEAFMVLK
SFPIIAKVFQSLPPWLLMKVFPDGAGFRELEVDAEAQLKTVLEKTRNGEVKTGHRTVFLEMLEGYHNPNPQDLVHEAVSV
VGAGMHTTRWILCVGALEVARNPEIAWKLYEELKTAIPNINDNLPYEQLENLPYLRGVVKEALRLGYGIVSASPRLVPRE
GAVIGGYHLPSDSVIEIDHYSVSHDEEIFPDSYTFSPERWLSPESKTKEKYVIAFGAGSRQCLGVNLAYCELYLAFAGIF
RRFELDAVGHDHMTFSDHWMPILRGESFACKVRSRQD*
>CYP51F1|487368|Morco1
MGLLATLLEPLGPYYAELQKLGTPVIVVVGVLTFIVLSVVINVLNQLLFKDPTKPPVVFHYFPFFGSTVRYGMDPYAFFN
SCQEQYGDVFTYVMLGRKMTACLGPKGNDFVFNGKLSEVSAEEAYTHLTTPVFGDGVVYDVPNNILMEQKRFMKFGLTAE
TFKSYVPLIVEQVEDYTKKSKFFKGPKGTVSLSAIIPELTIFTAARSLQGKEVRDALDGSFAKLFHDLDMGFTPMNFLFP
WFPFPSNKRRDNAQRTMAKFYMSIIEKRRKEEASGVDHSDMIWNLMGRSYKDGTPVTDRNVAHMMIALLMAGQHTSMATI
TWMLLHVADQPKLVKDLYDEQVRVFGNGKGGIEPLSYEKLSECTILNHTIRETLRTHPPLHSIMRKVKAPMHVDGTNYVI
PKGHYILGAPGVSAMDEKYFKNPHVFDVSRWEGQKTEEEGEKMDFGFGMVSKGTASPYLPFGAGRHRCIGEQFANVQLSS
IMATFIRNFELGYPEGCGVPKPDYSSMIALPTPPCTISWVKRDP*
>CYP573A23|490211|Morco1
MLYSTKEGVFTSVVVIIVLSIAKSIITSRYFHPLSQFPGPFIASFSRLWIVYWNLLGVEYLKEYDLHKKYGPVIRITPTM
LLVSDPKMLPEIYHRRADKADHYVTGSFGKTPSVFNIQPHEVHAAARKRIAQPYSMTAVKPMEGLIDARIVEWTDKLEND
FVKTGKKFDFAPWVTYFAYDVISEVAFGKSLGFVTKGIDIDGLIQSFHDGLPAFGFLCRLHPFTKWIKETWVADAYMIPK
PGDNTGIGNIMTFRDNLLEERVKNNNDPDHKVEYKDLLQNFLAAKNNDGSPMPIEDIKAETLLVLLAGSDTTATSVQALM
VDLLSNPRCLEKLIAEISEKDAAGLLSPVPKYDEVLEHLPYYCACLREAMRMTPAAPNIFPRVVSQGGLNLYGKHVPAGF
EVTENPWISQRDKTVYGEDSEIFRPERWLEDPKKAQEMDKYDFTWGFGTRVCLGKNIALMELHKAPVQFFRLFTPELVNK
EKPSTYVVAGGVSFHKDLWLNIKKRQV*
>CYP578X1|507282|Morco1
MLASLSMVLILFVFFLLHSLYTHLRCPLRRIPSAHPIAPFTRLWILSRTLTGRRNRSIHAAHVKHGPVVRLGPTEISIND
PSLIKSVYARLDKSPWYYIFTNYGSKPLFSLIHGREHSQRKRLIANIYSNSTISSSPTLLANTRKILEERMTPILDTREV
HEVHELYNALTLDLITSYLFTQRHGTDFVRDEATRKRFLVDLYQIRRPYFSWASELPGLVKWVGRLTAGKVKIIPSWVDD
ANDQIEEWCLQMCDAAEADMPTEKGADDDCVYYRLRRNNALGRTDAASEILDHIGAGHETTATALTYATYNLSVSPERQA
RLRDELKTAENLWRFKDVDALPFLNSVVLETLRLNSPIPGSQPRVTTDGGCMVGEYCVPGGVTVAGQAYSLHRTFWADAE
VWRPERWVNNDEEGKEGERWLWMFGSGGRGCVGKWLAVYEMKMTLACIYANYETRIVDDEGIEMDDRYTTGPISNQLLVG
FHKL*
>CYP6535A2|517252|Morco1
MSRLVGTYLTALFEQAASIPTQKLVLYSTLLGINSHLGYFIHGDRDHLSGSFFIGFFFLPSLIFGVFHKHLGIATVEALT
LTATIWISFLAGLTASIITYRLLFHPLRKFPGPFGAKVTKWWSVVNAAKTLQNHELGLDLHAKYGDFVRTGPNELSINCE
AIVPIVVKLAKGPWYNLGKPKPSTQLTRDRDVHRANRRLWDKAFTSDAVMQYLPKLIIRSTELLDIIAAQNPVNISDYFN
YYMWDSMGDATFSKSFNMLPTHGKENDYMRITKNATTGLAIFGHVPYLPHILDLIPGTNRDYKGFLRWCDELVNERRARN
PEKKDVFSWLLTADAKTMAGIGMANEARLAILAGSDTTAATLTNICFHLADNQDQLKILREEIDALYSSYQDDEDRIFKD

LAMGKCDNTDHLEGVISEVLRLHPVLPSSPQRMAPPEGIMINDTFVPGNTAIYAPMHTIFRDPRNFERPKEFLPERWYKT

SKYPLTRDQKVFIPFWTGTWSCAGKSLANMHLRIVITRIVRRFSIQLAPGMNEEKLNKDAQDLFILYMGDVNLILKERNM

*

>CYP6818A1|517951|Morco1

MAILYELLETHRTSALVLLGVSVFFFARQATKSKAGPPFAGTWSSDFLSVYIYGCFKFVKYARSMLDDGYSKFPDTPFRV

PAPTSTKIIIPPKFVKELRNYPDSKLSFNAAIYDILGLKRVELQSLGEDNTPHQKMTRSQLTANLAKLIAPMLDESKYAF

QVEIGSAKEWKAVPLHSTIVHIIARTSSRVTVGEEICRNEDWLRASTGFSKDIFDNAFCMFLFPSFMRPLAVFLLPFRRR

IRCQRMESRKILGPTYTQRLADTEAGKPRVEDVFGWMVANALPKQRNINDLVDYHLALSLGAIQVPASTLVSIIYDLAAR

PEYIEPIREEIIQAVTEDGGQINMSTLAKLMKMDSFMKESQRFNAVLTSFSRKALQSFHLSDGTHIPAGSILGASAVSVH

FDPKYYENPDEFDGFRFYKMRQQKDAINKHLFVSVSPTEINFGYGAHACPGRFFVANKIKVLLVRMLMKYDFKYPEGESR

PRNIMLGDATLTNTTRNLMMKERVDAPKWDFL*

>CYP512CR1|519716|Morco1

MALRELIEELSSIDKTTLAAVVFVGLVTYRVYYHYSVYVDVESVGVPPGILGPWKAALRHTKEADALIKEGYKKYSKDGK

FFKIASIPRYLVFPTDPKHLQEMNSAPDHVLSFGDAGADRISIDYTMDHSFRTETYHLKVIQVYLTQRLSSILPEIISEV

TLAWQENTNIGDEWTEVNVWDIMLKVISRTINRMYVGLPLCRNQEYLDNLVEYATTTSKSGIIIDMAPRILKPLVSWYIL

DKPKKLKKLMKFVGPLFEERKKKMKELGDKWTDRPDDAIQWILDSAPLGADTDTESLVNRILFLNFAAIHTTSISITQAL

YDLAVHTDLHNPLKDEITSVIAEEGGWTKQGLTKMKKLDSVLKESQRMNNVTSIAMMRKAMVPYTFSDGTFIPKGTWLCA

PAAAIHKQEEMQYDGFRWERLRKEEGQAAKHQMVSTSVEYLSFGHGRHACPGRFFAANELKILVASIILNYEFKSPTGER

PKNGHFGVSCVPDRGVNMLFRKRKDV*

>CYP617Y3|521406|Morco1

MSKRLILASTTVASLLLARHDPAHFPSSTWTFLGISTLLLTGLFVYNAVIFPLMLSPFRHLPTPKGANWLLGHFLDIHRS

ATGVPQLRWMEELPNNNGLIRYRGLFNGERIVPTSAKTLSEVLHQKSYSFIKPDFMRNGIGRILGLQGILFSEGDEHRHQ

RKLLLPAFSHSQIKNLVPTFWDVSLELTEKISEIVSASVETPVIDMSHWFSLATLDIIGLAGFGYQFNALASVKNGDATS

ESGSELAQAYNTIFKTPAVALAMHIARLVFPRWFLRLLPIKRTQEVKEASKVVKRVSLEIIDAKKLEIAEKKAVTDRDIL

SVMLKSGQYDGPGGVEIMRDQMMTFLAAGHETTATAMIWALYVLSLKENIHIQNRLREEIRDAFPHGIPEKVSYEQIESL

KYLRNVTSEVLRIYPPVTLTARYAAEDTTLGDQFIPKGAHIVIVPYAINRSKELWGEDADQFRPDRWNEGQGESNYAFLT

FLAGPRGCIGNVFAKLEFKCLLAAMIGKFAFVEAEEGREIVVRGGITAKPIGGIPLKVSIVEGW*

>CYP61A1|523863|Morco1

MAEITANSSTFAHPSAVSSAEQPFQSLVNTVGELGKSLEGISAWQAIIITILLLSVTYDQVRYLWQKGSIAGPSFKIPFMG

PFLESMNPKFSEYLAKWNSGPLSCVSVFHKFVVIASTRDLARKVFNSPAYVSPCVVDVAKKILRPSNWVFLDGKNHVEYR

KGLNGLFSRQAMGMYLPGQEEIYDTYFKRWLEISKDGKPRPYMSEFRDINCAVSLRTFVGHYITDEAVKEISENYYKITE

ALELVNFPIIIPFTKTWYGKKCADFVLNEFARCAALSKVAMEAGKEPGCTMDAWIKSMIEAREAAKKHESGELEGEMKAS

AKIRVFSDMEISMTIFTFLFASQDASSSASTWQFQLLADRPDVMAKVREEQLRVRGGDPYKRLDLDLIDQMVYTRAVVKE

QLRYRPPVIMVPYEVKKSFKLTENYTVPKGAMVIPTLYPALHDPEVYNDPEAFNPDRWLEGGEAEAAKKNWLVFGTGPHV

CLGQNYAIMNFMSMIGKASLMMDWEHHTTPQSEDIKVFATIFPEDDCHLVFKERLPLTPPTSS*

>CYP539A37|528243|Morco1

MLLDALQQSAPGIALTLLVASILAFCFAKIRQNLRIRKLGGRAPNINTYPFLFGLDVILDTMRMAWKNQNFELWRRRFDN

SGHTVEMELLGQRIILTEDPENIKAILAAQFSDYGKGKDFHRQWLPFLGNSIFTTDGSEWHSSRQLIRPQFIKDRISDLE

TFERNITHMMSLIPHDGSAVDIKDLFYRYTLDIATEFLLGNTVNSLGSPRAEFAVAFAELQKFMNDISRVGPAENLFPRG

KFNKNLAILNSFVEPYVEQTLQLKPSELKDKTEKNYNFLHALAEFTRDKRMLRDQLVAVLLAARDTTAATLSWTLHELSK

KPEVVQKLREEILRSVGPDNNPTYSDLKNMKYLSAILNETLRLYPAVPFNVRTSLKDTYLPRGAGVDGNEPVGVPKDTPI

AYSTLTMQRREDIFGPSVNEFDPDRWSKWVPKSWQYIPFNGGPRICIGQQFALTEMSYTIVRLFQEFEAVGPRMTKVQWM

QTEIVGSPGEDVVLSFKPAKC*

>CYP50308A1|536854|Morco1

MAIVETLIYTVGSIAGAGFWKYGILAVMPFLWFWLNLRYGLGDFDIPTIGVPPRLLGPWIAPYNWMKNSPHLIREGMAKY

GARGLPFKIRSPSRWMVFVNKPTIIDELIRLPKNVASSSAALDDIFFTQFTMYKDAASDPPFAEIVLKQLSGRCWEFMPL

AVEEVKMAWDEKTKIGEEWTGVPVWEVFIQLAARAVIRSIIGAPLCRNQELLDNIIAYAEGVIMHSELLEIIPATLRPFV

NKFIFRNKPLDVCMSYFGPIFQERRNLMKDGKYSGNVKPDDGFQWVIDTCPPDTTIERMVKKLLFIFTAGIRTTAVAGTH

CVHDIAAMPHFQGPAREEIAQEPGAASTVKETKEAWAPMRKLDSVLRESARMNRTHLGTVMRKFMVPYTFSDGMEIPKGA

WVVGASSTIHRTNIYEDPDTFDGFRFSRMREEAGIGDRFQLTGTSREFLAWGGWGACVPGQVLCGDAVEGYICIAFTEL*

>CYP51062A2|564943|Morco1

MVLLWTLLKKQEPIPTSFNGRPCEPLKLRKDPYILGIHGIRFGVRSIMTRDHRNIQAMLATQFNDFGLAHERRAHQAILG

TRGIFTKDGPEWKESRALLRPSFDRASVADLSALETFVTRMIAKINASPGSDGYHEVDLQALLLELIMDASTDFLFGSPI

GAQEGRVSAEGQMSFSESFDVSQDVVSIRFTLNDLYWLVNPKRFRIAITVVHKLVGHYVTRALQKHNSNSSADKPYTKDT

STSGKYIFLEALSQKTQNPQVLQDQILSVMMAGRDTTASTPHGPFTSSPATRKYSRSCSDLREMVYLRCFLHEELRMYPP

VGINSRSALRTTTLPYGGGADGNAPIVVRKGEGAMYSVFVVHRREDV*

>CYP52AX1|469011|Morco1

ANAKYAREHNCHPPKVHLTKTVFPFSIVNVYQFARAAASKRHINHFLDQVQRFGNTRVYSSPLFIGHAIVSIEPEIIKTI

LATNFADWELGKVRHEAFSALLGDGIFTSDGKVWEHARALLRPQFVKDQISDLNDLEIHFQDLLSILTAPGEGQVVELHK

LFLSYTMDTSTSSLFGESVFSLRRRGGDGVGESFQDDFDYAQLAMGYRLVVWGYHLLYRPRRLPKALKRIHDYVDEFVDR

AIQRQRVEKKEEGKYVFLEELAKATQDRKVLRDQTLSVMIAGRDTTATLLSWVFWLLSKNPKVWGKLRQEVLYEVGKELP

NYQQLKDCRYLKWVINEALRLYPVVPINFRYCNKNTTLPIGGGPSGDLPMFVPKGSRVVYSVYALHRRTDIYGADADEFR

PERWGEMTPRAWEYLPFNGGPRICIGQQMALITASYTIVRMVQEFRAVEDADIVREQVLTNIGLTLSPANGTPVRL

>CYP6001C27|501111|Morco1

MASNGASSSSAGIKPPPPLAKQTRKDVDSSLDKFSALINASNRPLPNRYGNGTDRSPEAEQTGIWTDISTLRKGGFLLES

VATLWQLVQQKRRGGPVDDKTMIMERVIQLTSRLPPTSKVRISLTTHQVDQLWDSLQHPPLSYLGSQFHYRQPDGSYNNV

MYPQLGAAGSPYARSVKPMTKMPAAPPDPYLVFDSIFSRGKNDENYREHDNNVSSMLFYTASIIIHDLFRTNRDDANISD

ASSYLDLAPLYGNNKDDLDKMRTFVDGKIKPDSFSEKRLLAFPPGVSVMVIMFGRFHNYVAEQLKAINEGGRFDLKYPRR

PVPDKTREQIHAEALANQDYELFQTARLVTCGLYINFILNDYLRTIVNLNRVDTTWTLDPRFDPDKIYNPDGTPAGVGNM

VSVEFNLVYRWHSCISKRDDAWTKEFYAGLFPGKNPDDVNMREFLVGVNQWESKIPSDPVKRTFGGLSRQGDGSFRDDDL

VKILTESIEDVAGAFGARNVPHVLRLVEVLGIEQCRKWRIASLNEFREFFALKPHKTFEDINPDPKIADTLRQLYDHPDY

VELYPGLVAESDKKPMVPGVGIGPTYTISRAILSDAVTLVRADRFYTVDYTAGALTNWGLAEASSDVNVLHGCVGYKLIL

KAFPNHFKSNSVYAMYPLTIPEENRRILTSLNTVDQFDFSRPKFTPVRIPISSYAATKQILNDSTNFKVTWGEGFDFIMK

ADFMLSGDTSSNAQQKQFVRERFYLKDVDWKAQIKQFYVETTRELLKQKSYHIGGRYMVDAIRDIGNMAVTHFAATIFNL

PMKSEDHPKGIYTEQELYMILCVMFIVIFFDVDPSKSFPLRQAGYGVVRQYGSLVELQVKSIKNWGWLQGIWDPTNVRGR

NNSALSSYGHHMIKRLLDGGKSAYDVTWSYIVPVAGASAPNQGQIFAQVLDFYLEEKNAHHLAEIQRLAALDTDEAWETI

KKYALEGGRLAGTFGLYRRCSSDVTINDYDRILHLKDGDLVFVNFITASRDKDVFPDPEVIKLDRPESLYMQYGVGPHEC

LGKAANIIGLTTMLMEFGKLKGLRRASGKPGILKAIPKPGGFKVYMKEDWSGFWPFPTSMKIEFDELS*

>CYP505A66|479546|Morco1

MTTPIPAPPGFPIIGNLADLDSELPTKTLRHFADIYGPIYRFDFGGGATKVVISSQEIVEEACNEERFIKVIQGGLNQVR

NGVGDGLFTAHHDEKNWGIAHRILMPAFGPLSVQGMFNEMHDIASQLVMKWARHGPDHKIVATDDFTRLTLDTIALCAMD

YRFNSFYTDELHPFVGAMGDFLSESGRRSQRPAIANLLMRGSSQKYESDIALMKNVSDEVIKRRKEKPTGKKDLLNAMLN

GKDPVTGEGMSNESIINNMITFLIAGHETTSGMLSFAFYLLIKNSAAYRTAQQEVDDVCGKGPIKVEHISKLKYLNAVLR

ETLRLHPTAPAFSMCPKPGVEEHPTLCGGKYAIGKNEPIICLLPKVHTDPAVYGEDAEEFKPERMLDEAFEKLPKYAWKP

FGNGMRGCIGRAFAWQEALLVVAILLQNFNFQLDDPSYQLKLKQTLTVKPKDFKMRATLRDGIDATMLEKKLQAGSAPTA
EDSTLKETLNKPSNLKPMSIFYGSNTGTCEALAARLAKDAIQHGFDAKVDPLDSATDKLPKDQPVVIITASYEGAPPDNA
SHFVEWLKSLKEDELKGVQFAVFGCGHRDWQATFQKIPSLVDELIGKWGGERIVNKGTADAANGDMFTDFDGWEDTQYWP
AVEKNYGGTATSPDAVSTPSIEVTVSSQSRSTNLKQDVRGALLMENRLLTAPGEPEKRHVEIKLPTDMVYKAGDYLAILP
TNPKNNIRRTVARFGLAWDAVLNIKANGPTVLPTNVNITAADVLSNYVELAQPATKKDVLAIAKAAADEKTRTALENLAG
DLFNVEVSQKRLSPLDILEKYPSVAFTFSEFLAILPPMRVRQYSISSSPLYAPDSCTLTFAVLDQEAISGQGRYVGVASN
YLAHLEAGDHIQVSVRASAHPFHLPLDPVSTPLIMVCAGSGLAPFRGFIQERAAQIGAGRKLARALLFVGCRGKGDTLYD
EEFKKWSAMGAVDIRYAFSRETERSEGCKYVQDRLWHDRVEAVELFDEGAKVFVCGSREIGEAVKVTCKKMYSEKAAALG
KPKSEEEVEEWFSNLRNARYATDVFA*

>CYP567W5|525230|Morco1
MALLSKFAGAHNVSDLNVIDLVTISGVLVIVYFVSHAIYNIYFHPLSKFPGPKRAAVSHVWLMRILTSGTSPKTHKEIHD
KYGPVVRISPNQLSFNSASSWKDIYGHVGGRQQFLKSDAYQNEYHPSIVSARDPVQHGQMRKLLSHGFSQKALMEQEDIV
HEYVDLLIKQIQTHATNKPQGEEMVKWYNYITFDIIGDLAFGDPFGSLKSAEPHFWVDSIFKSIEFLPWITVFKRVPITK
YIKQYITPPGAAEARKAHFQYSRDKIIKRMNNNTPRKDFLTRILAAKEAQGISVDELQSHSNVLILAGSETTATFLSGFT
YYLCKNPSAYAKLTTEIRDAFQNYEDIDGISTEKLKYLNAVIDETLRIYPPVPVGMPRVSPGETVDGMFVPKGIEVSVSA
WASTHSEANFHKPYEFIPERWIDPSCTDKKEASQPFMLGSRVCLGRNLALLEMRAIVAKMMWTYDMELKNKDLDWDRDNM
CYTLWRKPSLMVNFTRRSGIVVPPLDC*
>CYP50115A2|525718|Morco1
MDAILTPNWGLVLLISPFLFLIAVGIRNRYFHPLSCFPGPFLASTGPLYSMWASNTGREHEIIASLHKKYGPIVRYQSDM
LQFAEAGMVPVIYSRYANKTEFNRINLPGQVPGVAGLIEHKEHSARRKQIASMYSMSAAKQVEDMVDMRIMEFKSRVREK
YVGVGKKLDFARWTQYFAYDVISEVAFGKPLGFMASNSDVHNLIASAHNGLVVLVTLARSDWLKKAVRHPLVMKYFTPAM
SKETGIGRLLEERDTIVAERINEQSTGAHIGKRADLMQHLLNTRDTAAPLTLNEIKAELLVVMFAGSDTTAATLRGILLN
LLHTPSYYQTLISEIDAYTAARPPFSVISYDDAYALPYLTACIKESMRFSPATPSILPRYPNKGGLTLADGRYIPEFTKM
TVNARVCQRDPKVYGEDADVYNPQRWLEGGDKVREMERHDIHFGAGSRTCLGKNIAYLELWKMTFEFFRTFKPTIVEPEK
MTIRNIGLLVHDKLYVQLEERA*
>CYP52AV8|531094| Morco1
MADLPPLSIKSLAVGLLILYFVRKLILFVYRSYTNYRFATANKCLPAARFPSPWIGIPNWWSIVKAARTSEHVALICNRY
KQYGNTITGYMLGATHIATCEPENIKTILATNFKDWGLGSARYNSFYPLLGPGIFTSDGPAWEHSRALLRPQFSRDQVSD
IESLDIHVNRLMGWIDKSAGKIVDLQPLFFRFTLDSATEFLLGESVNTLLEEDDGKRANGQMNFGQAFDTAQKYLMVRMR
ARGLYWLVNPKEFKAANKICHDLVDRFVYAALGKKNSMKSEPQPEKKKYVFLDSLVEQNEDVEYLRYQLLHILLAGRDTT
ASLLGACFVLLSRHPQVYAKLRTEILNSIGDGKDGRIPSFAEIKDITYLRYVLNESESPTSFAGLQQY*
>CYP6136C1|531605|Morco1
MLLENLSSILAILWQNKLLLSLALIVYLLLKQTYQFLNSPFRAQRIPGPFLASFTNLYRSYRTMTGTWHRDIMQLHLKYG
PVVWIAPDEVSCSDPYLRNVIYGFANDKREETFFKKAPLYETGSVNEDFSFLFEREPEKARLGKRLMSHFYSETALSGLE
KNFDQAVDEFVQGIEKHVIQPGKVCNFTHWVEYYIWDVTAQIACNNSNGFCLAGEDHFGTLWGIKLILSVVGWLLPTPQA
LMITTRWIRRMLLVYWLDQLFYTGMGIGEKKWKVPLEERIDEVRTENPDHLLAKFHSAQASMRDHYPLGDTVQGTTVQYF
NLMAGAVGVTPHTTITAIHNLLKDPTALSTVRAELAALPKAVTFADFIHYGGSNKIPYLEAVINEATRICPAVGFSLSRT
SPPAGCQLNQFFIPPGYTVGMSAWAVNYDKGYFGADAAEFKPERWLGVDEHGRKRASKMEAGWLSFGAGGRVCIGRHLAM
FLMVKLVATVVARWELEEVEAPKERFAMVVEMQGMMVKIKERAVEG*
>CYP52AW1|533076|Morco1
MGMSTLIVQISAGLGLYIICLTFLALRRRFKNARFSREHGCLPPSQAPTDWTFNISRPFILSKVRKAHKFMPTQQESFHR
NGNTFAFSIAGKRLIMTCEPENMKAILATQFKDFELGKLRHDNWGEVLGDGIFTVDGHQWEHSRAMLKPQFNKNQITDLE

DLETHVQNLFSRLSLAENGGSVLNLHEYFLNFTLDFSTAFLFGKSVGSQLCQGQTSKFAQDLDYAQKVISFRVSLENFWW
LYRPKKFTKAVADVQAFIGESVQQALDKQKSEKGKTSEKYVFLEALARETQDPVVLRDQMMNILVAGRDTTAGFLSWIVY
SLARNPRVWDILRREILTDFGRDTPTYQQLKDAKYLKWVLNEVLRLYPLVPTNFRYANKNTTLPLGGGPDGLSPIFVEKG
ERCVYSVYSAHRRKDIYGEDADIFRPERWGEGKPRGWEFLPFNGGPRICLGRKCSLLYLCLALRGGGGVLHNPCTSCMID
HTPRSVRSILPSSLRFFRVTEGC*
>CYP6521A1|535142|Morco1
MASNIVIRLSQGLTVLADLPLYQLSLLSFTTAVTSHITYFCHSERDLIAKQIFLVAIFAPLAFQSLFQFHLKLPFWSCVQ
LTSTIYGSFLLGMWSSMVVYRIWFHPLRNFPGPFWAKVTKLWIPYTHWRTNWQYHLVMLELHKKYGDIVRIGPNDISINH
ADGLQALAKAKKGTWYNFADGEHSLQSTRNSKTHAIRRRVWDKAFTTKAVQDYLPRVLKYTDTLMAHISKNAGSPINVTD
WFNNFMLDIMGDLAFGKPFNYMKPEKRVGAFDFGAVMHDSAYVISSLGQVSYSFTILSMLGAGGDVQRFLDFCTDLVRER
KTYTPKERDIFSYILDAEPQNVGEHKLSLMGETRLIVVAASDTTSATLVAIFALLGTHPHQLRKLRADIDAIYDAYPDER
VPKEIINGGTPATRHLEATITEALRMSPAVANGVQRMTPSDGIVVDGVSIPGGVNAIYCFRAAHMDERWFPDAQEFKPER
WYEADKETLARQRSAYAPFWLGSYECVGKPLANMQLRIVTSQLLRKFDIRLAEENVDVWKMLNRSKDLFTTMMAPTNVIF
TPRGSS*
>CYP51093A1|538641|Morco1
MGYATAVLIALLVYRLGLYLYRYTLHPLAKFPGPRLAAVSSLYQIYYDIIKDGQLTYHLQDLHKKYGPIVRISHNEIHLS
PPSAYHEIYRLNTPLIKHHGFYQLFNLSNSSFGTVSNADHRRKRSVLNQSFSRRTIFELEPLVQSKVDRLVQRLEDEYAG
VGREVCVHNAYQAVTIDTVSDFSFGISYGVLNNADFRSRAMEAFDKQQEAFLVFKHFPLLARLAGNMPFWLANLLMPDGA
GFLEMEQDCRHQIQHLLSPAADTPPAKAGHPTIFNRLLEAYPDPDEKTIDYLAKEGVTVVSAGTHTTRHALCVGTVYALQ
KPEVGERLLRELREVMPGVRDVASLKELESLPYLTAFMKESLRLSYGVVGPLPRVVPAEGATIAGAFFPGGTVVIMDSYI
VHHNEDLFPSSHEFDPTRWLVPNAKELDKYLVSFSAGSRQCLGMNLAWAELYMVFARVFRRFEMELYEHEGEGIKIVEHW
LPTVRGEKVRCTLKVREV*
>CYP5268A9|540389|Morco1
MQIVIVGAACAVLFFAYRAYQTYLSIQNEQSLKSSKTCGTVLPGPTPAPIIGNILQIPKTHTWIHFKQWADIYGPIYRFQ
VGSKNNIVVSTEKIANDLLRGRGNIYSSREFLYFATELLSKNLRPLLLPYNDTWRRGRKLMHTLGMPKVVDTFRPAQSLE
AKKLLHDMLEDPESYENHFERYAAGIIFRLVFGKAVETGREPYVREIYNIVHTVERVAAPGAYLCDSIPLLRYIPEFLSP
FKREAKWLHNREIKMFRELQNDIRVGIKNGSAPECLTRTFLENEGEFGMTSDESAYVIGTFFEAGSGTTAGAMLTFCLAM
CLHPHVQTRGQAEVDAVCGDRMPDFEDLPNLPYARAVMKEILRWRPVLAGGMPHELTKDDEYEGYKFSKGTVVHANLWAI
HRNPEQYPDPETFNPDRWVNPAYPTYKEPLSVYPNLQNFSAFGFGRRICPGMNIAERSLYLLTIRLLWGFQMNKKRDASG
NEIDIPYYDYDSGLSSRAKPFQFDCVARSPMRASVIRKSWDEAKATDPLRSRN*
>CYP51041A1|544994|Morco1
MILEQKTYYDVWMGGELAHQVKKLHKKYDIEKVQFSNSRMIKDEFMYQCFGEGDSTFGFIDPNLSRRRREVLSPLFSRRN
IISLQRLVIEKVEKLCEKLTEYHDTSDAADISSTFRCTTMDIITSFCFAESLNALDHKGFRHPVLVGVKIALPMYWVFKH
FRPVQWIFMHTPTWLSLLTSPETAGIMGVQNSISDHLDRFLANPKSPAIADHVIYHRLLDPENKRAGESLPTRHGLFDEA
MALLIAGSDTVGNTLAVGGFHVLNNPSVYEKLFNELKKNWPVLEKSLGYEELEKLPYLESLRVSHGVVSALPRIVPPSGA
TIGGAHILGGTVVGMSSPYVHTNEGIFPDAHRFIPERWLQPNSKHLEHWLVPFSRGPRMCLGVNLAWCELTIGFAHIFRK
FDLRLHETTERDIRWKEYFVPHFTGKRVQSFIKLRPS*
>CYP567W8|549086|Morco1
MGSTLSRLEVFTPYLTYPNLLSLAAAYCALYLVVEAFYNLFINPNSIYPGPKLAAVTKHWISVPWVAGNYPLIIKGLHEK
YGPVVRIGPNELSYSSASSWKDIYGHVGGRKVFTKSSFYNDGTEPSVVSERDPHKHGRLRRLLSNGFSARSLAEQEPVVH
QFVDKFIMQINKHVTNPKGDDMVKWYTFVAFDIIGDLAFGDPFGSLEDGKTHFWVENVSKGVAAGKWIFAFQKNWILSNL
IPQLVPKHLKIAREKHLNYCQGKIDKRLEEKNPKKDLLTVLIAKHNAGERFTPGEMRSNSQLIIVAGSETTATFLSGTSF

YLCRNPATYKKLVDEIRGAFSSYDEITGLATESLPYLKAVIDEGLRIYPPVPMGMPRVSPGETIDGKYVPEGTIVMTSSY
AATHSELNFHRPDDFVPERWIDPNCTDKKEASQPFLLGSRVCLGRSLALLEMRLILCKMLWSYDMEMVNPEQEWFQENTF
GMLWEKPKLPLRFIRREGVEVPPADY*
>CYP5093H7|551692|Morco1
MLVTVWAGRRATIVIGDPQVACDLLDHRSAIYSSRPRFVIMGELFTNNDALLTMPHGDKWRKTRKIFHSGLLTKACNSYK
PIQVAESQRLVMDLVRSPENFGKHLERYAASLMVCVAYGQRVDNLEDPLIKRIYERMAYMATLNVPGAFWAESFPVLKLI
PDCLAPWKREVKRRAEESTQLLSELAFDVRDRMETGDAPACFTKTLWEKREENPEALSEREIAYATGSLFGAGSDTSSST
LTSFILAMTCFPRVAAKAQEELDRVVGHDRSPTWCDAPNLPYCSAIIKETLRWRPVAVMGGTPHASIIDDHYNGHFIPKG
TTILGNLWAIHHNEKYFKDSHDFIPERYLSPCEDGTLPYPHRDGHSAFGWGRRICPGQKLAENSLFITITRILWGFNISK
ARDQITGKEIVPDIFAYTDGFNSKPQPFQCRIEPRCPRVLEVIEQESLLGEQFLEKYKSR*
>CYP6470A1|562426|Morco1
MGLVLGTAVPQPTALAYLTPQNIAVAIIAFLLEYIIKWRFFGPLRHIPGPWWSHYTQIPDGYHLMRGERALYIHSLHERY
GPTIRVGPTLVGVSGTAGVKMVYGTALKKPFTRNPQITAMFNFQRKPEADNIASFHLPGDALKRRRAYGNIFSRGNILAM
QDVFKKCFENYFAKLETLRTASPNGIVPMVRCFRALALDAITEVSFGGFYKGASDPDHQMKLLDNLMSANLVQLHLGNAI
YSLLKSIPWKRLDWVRSMEDFCRITSDSTALYLSNRAANPSQARKDSLSQLLSYGLPRAAVEGEALANVFAGTDTTGNST
AFIVCEVLKKPNVHRRVVEEVFNAFPEGVYRMGEVMEFRKVEEECRLLKWCIKEGMRRHSLAPGPALRVVPDDGVVVDGM
FIPGKVDIFAQSHTSTHDPVAFPQPEEFIPDRWENETSEMRTLWTVFGSGPRICIGENLAMMEMQMMLALLFRNYELVQV
PGTDMRITEFWLRSMKAGELHLKLVPRSV*
>CYP5142W1|563696|Morco1
MEPQGGPRSLIITYGAYFIPPAVVLYYVALVIYRVFFHPLAKFPGPKKAAATGWYRSYYDVWMQGEMPRQIAVLHKKYGR
IVRFGPNHLHFSEPSAYHEIYANNSKLLKDSWVYNSFNASQSGFAMADPSAAKGRRDVLSSLFSHRAISSMQGLLAENFD
LLCTRLAEHNTTSSAPINLSGAIRSTTVDIITTFCFAQSMHALDVPGFKAPILLGMRTSLRMIQVFKHFPLIQRIVLNFP
RPLLMFGEVAGFIDLSRRLESQVANFLKNPADLDKTPHPIIYNRLLDPDAKGSRAAPITQTALEHEAHTMLFAGADTVAL
TMTVGLYQLLKSGRQGELVEELIKAWPELDRAPKLEELEKLPFLSAVIKESLRMGSGVVSLLDRKVPKSGAVIDGEQIPG
GAVVAMGTTIVHTNEDIFPNSGSFIPERWTGPNGKELERYLVPFSKGARQCLGINLGYAELYIGFARLFRRFEITLDGTT
EEDMRWKDYFVPLFEEGHMKGFVKPREA*
>CYP6480B1|598031|Morco1
MIILETLKKHAIGAIFLLFVSNVFYDLYLHPLARFPGPIWARITRLWWLRVVLSGKQHLKNYELHERYGPVVRITPNILS
FSDHRAIKVIYGRGAGEFAKGDGFGNGFLPGGDPPVFFAKDPVVHSRVRKRIAHAYSMTSVAQMEPSVSALIEEFSRNID
RFAERGEEFDLVEWVNHLTFDLISELGYGKKFGFMEKGGDVNGYQESMHHNSPMFPILAAFPTLAALLGVVIKYLPPPES
ATGIGHLFGVTKRIIADRRASTSSSTNKKDLLGGFLASKTEDGSPISESSVFGEVMGTLIAGADTTAASIQGASLYLLQN
PPALQRLVDELDQTNPVSYADTLPLEYFNAVLKETLRLSPSIGATFGRKVPVGGAEILPGIWVPGGFEVGINNWVVGRDK
TLYGDDAMEFRPERWLEKGNRTRFAEFEFGFGSGNRVCIGKNVAMMEICKTIPELFEKFELELMDPEDPWVEESRLFLYK
SGLRVRARRRVLKV*
>CYP6683A1|603348|Morco1
MGLLTIFSCLVALFLARWFTLLRRSLQAAKKTGLPYTCRPLYEGVLAYLLWMPILDYLIDYLSDWLATILDPSDYASIYR
SWRFKAGYENHRVLGDVFLVVSPGPLVLEVADPHVCAQITDRRMDFVKDFDTYLPLRILGRNIVEAEGETWKRHRRAVTP
QYTDAIHAFVWDQATIIGREMLSEWKHLEGADTPIKAPYTSRESRDLGTLANRVVMAAVFGVSLSLRTGKSPEALALSEF
ARSMEVINAGLLFVMIFPQLVMPYVPGIGHLSKAMNHVNLYMKNILDLHINAKDKPESSAKAGILDGLLKVLAPPGTVPT
EDQLSRSDVAGNMFALAFAASTTSAETMHFSMVLLALYPKVQLWVMEELDQLAVEYGQLNEGWKYQNVFPKLIRLSCVLM
ETLRLFPAVPMIPKCVEDRPQILSYKNSTFALPPHTLIALSACALHRNPKVWGADAGEFRPERWLADNGKSLRPEPVEGC
FIGFSRGTRRCLGRRFAEAEYAAVLSEVLRNCRIELAPQKPGETVKEMSLRARKVLEMSRTGFTLRVGGKVMLRFVDRF*

>CYP51073A1|605564|Morco1

MESLHPSTSHLPDLSTLLLTLLLISLIILLFHLLPPTTTAPQVGRATGLFSFFSHYSSGQHWLSFSAALIASGISLHGAH

GRPFKIRSYPRWITFLTSPALLSEARRLPLSILSYRETADEALQTEHTLCRGLMKRAWHMKPIQRDLPAKLARAMHEVHE

EAVAAWGESTGVAGGGWVKMNPHRALVNVVSRATNRVLVGAELCRDAEFMAATTALATGIIATAGRLEMLPGFVRPVAAW

WMRWGDRRLGAFLKYTDPIFAERRRRQGTWGMGEKPDDAFQWILEAAPKGASLRDMAFILIFLNLASIHTTAGTLTQVLF

DLCVHPHYQPLLREEAEEALKDGLSGSALGRMKRLDSVIRETLRMSPTNVAVTPRKVITSHTFSDGTRVGRGSWVAEPVM

HANRSGACYDRPDVYDGFRFVDMAGGRNQCVSTSVEFLSWGHGASACPGRFFAVAEMKIVLAIYSATMRLASSQGWSRGR

RALRWEFSRFPIQVWS*

Morchella importuna SCYDJ1-A1 v1.0

>CYP6188A6|534064|Morimp1

MHRIRNDTPLRDIPGPWLASCSPLWRFWYAVCKSNYHHDLTNLHRKYGNIVRIAPNEVSVWDPEYVSEIYSHGENVYPKC

DMYDIALPNGFFNLAVERDIKTHTLGRRAIAKDYSMTAILKSEKYFDAVIKDFITALDKNFAQTGKKCNFTVWSEYFTYD

MITDIVFGKSFGFCNAARDVEGGLRDLRQMLNLSPFLSYLPWIWPLTQLEIIKKTGMKHYARCMVREIQKRVREGNPTGR

QDLLQGLMDARYQDGHALPLGEITNHAYIFVLAAPDTASVALRNIIINLCRNRDVYQQLMDSLLALNTANPTWKDLQNIP

LLTAVVKETLRLHPPAGFSLPRAVPAGGRTVCGKFLPENTTIGMSAWSVHANTEYWGEDTLEFKPERWMDPNQMYKLERH

SLIFGQGSRQCLGKHIAMINLVKVTAQILLNFDFGLLNEKNIKEVFLLLVVVDGMDVWFKRRAGGPLDDTIGSESEKPAA

VTTGA*

>CYP532S1|535463|Morimp1

MAVTSIILSYPIFTPGVLAIIGLFIICLYCLKWRYRSGLRSIPGPFLASITNLYRFFDAWSWKCQDNQLALHEKYGKFVR

YGPNLVSISDPEAIQVIYTINKGFVKSPLYKVFFVLSNGKTIQSMFDTQDELYHQSMRKPISHAFAMSHLMDYEPYVDST

SKFFTSRLTELYANTGDICDLGEWLQYYAFDVIGELTYSKRLGFLDRAKDVDGIMRSIDKSFDYAGVVGQMPWLDTLLEK

NPIWNYFNPPTSAVAAFTLDCMAERMREMEKGNLRKQDMLARMIEAHKADPEKVTPAHILGWASSNTYAGSDTTAISLRA

LFYHLLKNPGSMEKLLSEISSFEGLSNPVSWTEARKMPYLEACIKEALRIHPAPSMLLERVVPKGGKDICGRFFGEGTIV

GINPWVVQRDKDVYGADAELWRPERWLEAGEISRKRMDRTAFAFGGGSRACIGKNISYLEMYKVIPQLLRTFHIELAYPE

KEWTLHNNWIHKQSGLLVKLTQKGF*

>CYP50357A1|541921|Morimp1

MHVPEFHPVTTIFVILFALSFSIISISPTLLFLCAKGRKAKDIKNKTFIYQSLKQAKVILNGSSDGKFRRLEDISTILKS

RAEPNSRLIRAFGIDNAFTTSDPQHHRDFVNRASDLLNLSNSRWKSLAKHIDTLVKGNVERQRVSSFDGHSRTVIFSQLL

QITVLRAVLSVLFPNGGTPQLSEEEQDSIAYEVAEKITSLWIESKSTEITSNPRDLESLMEACQKLLPGIDTRVENPLNL

ILPAFETLWRIVLRGFLEVQFRDKENFSLNKSLFSEYLTSPTSTVFMREDTRGVSVSHIINESLRLYPPTKRVYRKIQTE

STQPDVAIDIEQIHRDPETWGSDANIFRPSRWRKSEMNKTIAAAFIPFGMMPLICPARKIFGPRIIGIFLAALLGAINEF

GELDVVGAMVEGEVLLENGRNSYGEMGMRSRK*

>CYP512CR1|546061|Morimp1

MALRELIEELSSIDKTTLAAVVFVGLVTYRVYYHYSVYVDVESVGVPPGILGPWKAALRHTKEADALIKEGYKKYSKDGK

FFKIASIPRYLVFPTDPKHLQEMNSAPDHVLSFGDAGADRISIDYTMDHSFRTETYHLKVIQVYLTQRLSSILPEIISEV

TLAWQENTNIGDEWTEVNVWDIMLKVISRTINRMYVGLPLCRNQEYLDNLVEYATTTSKSGIIIDMAPRILKPLVSWYIL

DKPKKLKKLMKFVGPLFEERKKKMKELGDKWTDRPDDAIQWILDSAPLGADTDTESLVNRILFLNFAAIHTTSISITQAL

YDLAVHTDLHNPLKDEITSVIAEEGGWTKQGLTKMKKLDSVLKESQRMNNVTSIAMMRKAMVPYTFSDGTFIPKGTWLCA

PAAAIHKQEEMQYDGFRWERLRKEEGQAAKHQMVSTSVEYLSFGHGRHACPGRFFAANELKILVASIILNYEFKSPTGER

PKNGHFGVSCVPDRGVNMLFRKRKDV*

>CYP6535A2|561570|Morimp1

MSRLVGTYLTALFEQAASIPTQKLVLYSTLLGINSHLGYFIHGDHDHLSGSFFIGFFFFPSLIFGVFHKHLGIATVEALT
LTATIWTSFLAGLTASIITYRLLFHPLRKFPGPFGAKVTKWWSVVNAAKTLQNHELGLDLHAKYGDFVRTGPNELSINCE
AIVPIVVKLAKGPWYNLGKPKPSTQLTRDRDVHRANRRLWDKAFTSDAVMQYLPKLIIRSTELLDIIAAQNPVNISDYFN
YYMWDSMGDATFSKSFNMLPTHGKENDYMRITKNATTGLAIFGHVPYLPHILDLIPGTNRDYKGFLRWCDELVNERRARN
PEKKDVFSWLLTADAKTMAGIGMANEARLAILAGSDTTATTLTNICFHLADNQDQLKILREEIDALYSSYQDDEDRIFKD
LAMGKKCDNTDHLEGVISEVLRLHPVLPSSPQRMAPPEGIMINDTFVPGNTAIYAPMHTIFRDPRNFERPKEFLPERWYKT
SKYPLTRDQKVFIPFWTGTWSCAGKSLANMHLRIVITRIVRRFSIQLAPGMNEEKLNKYAQDLFILYMGDVNLILKERNM
*

>CYP61A1|564505|Morimp1

MAEITANSSTFAHPSAVSSAEQPFQSLVNTVGELGKSLEGISAWQAIITILLLSVTYDQVRYLWQKGSIAGPSFKIPFMG
PFLESMNPKFSEYLAKWNSGPLSCVSVFHKFVVIASTRDLARKVFNSPAYVSPCVVDVAKKILRPSNWVFLDGKNHVEYR
KGLNGLFSRQAMGMYLPGQEEIYDTYFKRWLEISKDGKPRPYMSEFRDINCAVSLRTFVGHYITDEAVKEISENYYKITE
ALELVNFPIIIPFTKTWYGKKCADFVLNEFARCAALSKVAMEAGKEPGCTMDAWIKSMIEAREAAKKHESGELEGEMKAS
AKIRVFSDMEISMTIFTFLFASQDASSSASTWQFQLLADRPDVMAKVREEQLRVRGGDPYKRLDLDLIDQMVYTRAVVKE
QLRYRPPVIMVPYEVKKSFKLTENYTVPKGAMVIPTLYPALHDPEVYNDPEAFNPDRWLEGGEAEAAKKNWLVFGTGPHV
CLGQNYAIMNFMSMIGKASLMMDWEHHTTPQSEDIKVFATIFPEDDCHLVFKERLPLTPPTSS*

>CYP6271A6|570057|Morimp1

MDTHIIIKQYAEWASIAKTWLLSLSKLEAFIILTTLWTVYRIGVYTYRLFLHPLSKYPGPRLAAASSVYEMYHDIVKKGD
MTFHMDELHRKYGPIVRISPNKVRLRDSSAFHELHKVGSPLIKDPGFYSLFGLANSTFSTINPIIHRKQRRILDPMFSRR
SILGFENVVQEKCDLLCYKIRELEQADSKVSFHNAFVALTIDIVTEYAYAKSYNTLLNEGFVSKVSSAFDAQQEAFMVLK
SFPIIAKVFQSLPPWLLMKVFPDGAGFRELEVDAEAQLKTVLEKTRNGEVKTGHRTVFLEMLEGYHNPNPQDLVHEAVSV
VGAGMHTTRWILCVGALEVARNPEIAWKLYEELKTAIPNINDNLPYEQLENLPYLRGVVKEALRLGYGIVSASPRLVPRE
GAVIGGYHLPSDSVIEIDHYSVSHDEEIFPDSYTFSPERWLSPESKTKEKYVIAFGAGSRQCLGVNLAYCELYLAFAGIF
RRFELDAVGHDHMTFSDHWMPILRGESFACKVRSRQD*

>CYP51093A1|574290|Morimp1

MGYATAVLIALLVYRLGLYLYRYTLHPLAKFPGPRLAAVSSLYQIYYDIIKDGQLTYHLQDLHKKYGPIVRISHNEIHLS
PPSAYHEIYRLNTPLIKHHGFYQLFNLSNSSFGTVSNADHRRKRSVLNQSFSRRTIFELEPLVQSKVDRLVQRLEDEYAG
VGREVCVHNAYQAVTIDTVSDFSFGISYGVLNNADFRSRAMEAFDKQQEAFLVFKHFPLLARLAGNMPFWLANLLMPDGA
GFLEMEQDCRHQIQHLLSPAADTPPAKAGHPTIFNRLLEAYPDPDEKTIDYLAKEGVTVVSAGTHTTRHALCVGTVYALQ
KPEVGERLLRELREVMPGVRDVASLKELESLPYLTAFIKESLRLSYGVVGPLPRVVPAEGATIAGAFFPGGTVVIMDSYI
VHHNEDLFPSSHEFDPTRWLVPNAKELDKYLVSFSAGSRQCLGMNLAWAELYMVFARVFRRFEMELYEHEGEGIKIVEHW
LPTVRGEKVRCTLKVREV*

>CYP567W5|574459|Morimp1

MALLSKFAGAHNVSDLNVIDLVTISGVLVIVYFVSHAIYNIYFHPLSKFPGPKRAAVSHVWLMRILTSGTSPKTHKEIHD
KYGPVVRISPNQLSFNSASSWKDIYGHVGGRQQFLKSDAYQNEYHPSIVSARDPVQHGQMRKLLSHGFSQKALMEQEDIV
HEYVDLLIKQIQTHATNKPQGEEMVKWYNYITFDIIGDLAFGDPFGSLKSAEPHFWVDSIFKSIEFLPWITVFKRVPITK
YIKQYITPPGAAEARKAHFQYSRDKIIKRMNNNTPRKDFLTRILAAKEAQGISVDELQSHSNVLILAGSETTATFLSGFT
YYLCKNPSAYAKLTTEIRDAFQNYEDIDGISTEKLKYLNAVIDETLRIYPPVPVGMPRVSPGETVDGMFVPKGIEVSVSA
WASTHSEANFHKPYEFIPERWIDPSCTDKKEASQPFMLGSRVCLGRNLALLEMRAIVAKMMWTYDMELKNKDLDWDRDNM
CYTLWRKPSLMVNFTRRSGIVVPPLDC*

>CYP617Y3|579469|Morimp1

MSKRLILASTTVASLLLARHDPAHFPSSTWTFLGISTLLLTGLFVYNAVIFPLMLSPFRHLPTPKGANWLLGHFLDIHRS

ATGVPQLRWMEELPNNNGLIRYRGLFNGERIVPTSAKTLSEVLHQKSYSFIKPDFMRNGIGRILGLQGILFSEGDEHRHQ

RKLLLPAFSHSQIKNLVPTFWDVSLELTEKISEIVSASVETPVIDMSHWFSLATLDIIGLAGFGYQFNALASVKNGDATS

ESGSELAQAYNTIFKTPAVALAMHIARLVFPRWFLRLLPIKRTQEVKEASKVVKRVSLEIIDAKKLEIAEKKAVTDRDIL

SVMLKSGQYDGPGGVEIMRDQMMTFLAAGHETTATAMIWALYVLSLKENIHIQNRLREEIRDAFPHGIPEKVSYEQIESL

KYLRNVTSEVLRIYPPVTLTARYAAEDTTLGDQFIPKGAHIVIVPYAINRSKELWGEDADQFRPDRWNEGQGESNYAFLT

FLAGPRGCIGNVFAKLEFKCLLAAMIGKFAFVEAEEGREIVVRGGITAKPIGGIPLKVSIVEGW*

>CYP52AV8|581551|Morimp1

MADLPPLSIKSLAVGLLILYFVRKLILFVYRSYTNYRFATANKCLPAARFPSPWIGIPNWWSIVKAARTSEHVALICNRY

KQYGNTITGYMLGATHIATCEPENIKTILATNFKDWGLGSARYNSFYPLLGPGIFTSDGPAWEHSRALLRPQFSRDQVSD

IESLDIHVNRLMGWIDKSAGKIVDLQPLFFRFTLDSATEFLLGESVNTLLEEDDGKRANGQMNFGQAFDTAQKYLMVRMR

ARGLYWLVNPKEFKAANKICHDLVDRFVYAALGKKNSMKSEPQPEKKKYVFLDSLVEQNEDVEYLRYQLLHILLAGRDTT

ASLLGACFVLLSRHPQVYAKLRTEILNSIGDGKDGRIPSFAEIKDITYLRYVLNETLRLYPSVPLNGRTSVRNTILPKGG

GPDGQSPVFVPKGQRCDYSVRAMHLREDLYGPDAAKFRPERWAEGAGKGWDYLPFNGGPRICLGQQYALTEASYTVLRIL

QKYESIEPADDLPYEKVPLVSSLTTAPALATVRLQKAA*

>CYP6136C1|582511|Morimp1

MLLENLSSILAILWQNKLLLSLALIVYLLLKQTYQFLNSPFRAQRIPGPFLASFTNLYRSYRTMTGTWHRDIMQLHLKYG

PVVWIAPDEVSCSDPYLRNVIYGFANDKREETFFKKAPLYETGSVNEDFSFLFEREPEKARLGKRLMSHFYSETALSGLE

KNFDQAVDEFVQGIEKHVIQPGKVCNFTHWVEYYIWDVTAQIACNNSNGFCLAGEDHFGTLWGIKLILSVVGWLLPTPQA

LMITTRWIRRMLLVYWLDQLFYTGMGIGEKKWKVPLEERIDEVRTENPDHLLAKFHSAQASMRDHYPLGDTVQGTTVQYF

NLMAGAVGVTPHTTITAIHNLLKDPTALSTVRAELAALPKAVTFADFIHYGGSNKIPYLEAVINEATRICPAVGFSLSRT

SPPAGCQLNQFFIPPGYTVGMSAWAVNYDKGYFGADAAEFKPERWLGVDEHGRKRASKMEAGWLSFGAGGRVCIGRHLAM

FLMVKLVATVVARWELEEVEAPKERFAMVVEMQGMMVKIKERAVEG*

>CYP50115A2|585227|Morimp1

MDAILTPNWGLVLLISPFLFLIAVGIRNRYFHPLSCFPGPFLASTGPLYSMWASNTGREHEIIASLHKKYGPIVRYQSDM

LQFAEAGMVPVIYSRYANKTEFNRINLPGQVPGVAGLIEHKEHSARRKQIASMYSMSAAKQVEDMVDMRIMEFKSRVREK

YVGVGKKLDFARWTQYFAYDVISEVAFGKPLGFMASNSDVHNLIASAHNGLVVLVTLARSDWLKKAVRHPLVMKYFTPAM

SKETGIGRLLEERDTIVAERINEQSTGAHIGKRADLMQHLLNTRDTAAPLTLNEIKAELLVVMFAGSDTTAATLRGILLN

LLHTPSYYQTLISEIDAYTAARPPFSVISYDDAYALPYLTACIKESMRFSPATPSILPRYPNKGGLTLADGRYIPEFTKM

TVNARVCQRDPKVYGEDADVYNPQRWLEGGDKVREMERHDIHFGAGSRTCLGKNIAYLELWKMTFEFFRTFKPTIVEPEK

MTIRNIGLLVHDKLYVQLEERA*

>CYP6521A1|588681|Morimp1

MASNIVIRLSQGLTVLADLPLYQLSLLSFTTAVTSHITYFCHSERDLIAKQIFLVAIFAPLAFQSLFQFHLKLPFWSCVQ

LTSTIYGSFLLGMWSSMVVYRIWFHPLRNFPGPFWAKVTKLWIPYTHWRTNWQYHLVMLELHKKYGDIVRIGPNDISINH

ADGLQALAKAKKGTWYNFADGEHSLQSTRNSKTHAIRRRVWDKAFTTKAVQDYLPRVLKYTDTLMAHISKNAGSPINVTD

WFNNFMLDIMGDLAFGKPFNYMKPEKRVGAFDFGAVMHDSAYVISSLGQVSYSFTILSMLGAGGDVQRFLDFCTDLVRER

KTYTPKERDIFSYILDAEPQNVGEHKLSLMGETRLIVVAASDTTSATLVAIFALLGTHPHQLRKLRADIDAIYDAYPDER

VPKEIINGGTPATRHLEATITEALRMSPAVANGVQRMTPSDGIVVDGVSIPGGVNAIYCFRAAHMDERWFPDAQEFKPER

WYEADKETLARQRSAYAPFWLGKMFISRITLYFV*

>CYP6775A1|588987|Morimp1

MTFLSTLIALPPIPAISTVCAIYITSICIYRLYLHPLSRIPGPKLAALTLLYEAWYNLFAQDGRFIFHTENLHKKYGPIV

RIAPNEVSISDPDFYNKVHYPGSQFEKDPVTYEAFGFGDSIFSTPTNELHAKRRAPIKDYFSRRSILAIEPVLLNTIKKF

LGRIDAAIATQPERLFDIQNGLRATSVDMSTTFSFNFPLGMLDMEDLGKRFSEDGQRRLKGFWVVKYFPWLPAVLSVLPK

SVEKWMLPAIQSLKDLTMMSRGSFDSIKASGETGTSIFHHLLSPENMKRDRLSDDDIIQNGVVCFAAGAETVSYTLIMTA
FGIVSNPDVEEKLYRELREAFPDPNELCLSKLEKLPYLVAVVKEGLRLAPGAFGYTPRVTPKGGIKYNEYTLPHEVWAHF
TTNTHIADGHCSRV*

>CYP548BE7|591920|Morimp1

MSILSSLSILLSSLVVYLASLVFYRLYLHPLAKHPGPFLARITDWYNVYHAYLGDRHLALHNAHLKYGPVVRFAPNLISI
NTSTALKTIYGHSPASRSLQKGSFYSAFPAVKGVYNTHNCINKTEHGRKRRVLSAAFSENALKSMEDLVLGNIQVFVDEI
ERRSVQLRKGIDMGEMFSWLTFDVMGELCFGKSFGMLRDETTRFVTHLIAQAAHSHYINGNYLPLATLKISRILFPTIQR
DRWRFIQHSRGCANERMKLGADYKKDFFHYLLDAKDPETGKGFETAELWGEANVLMIAGSDTTATALAATLFYLLRNPTK
LATLQKEIRSAFASADEIVGGKQLTDCHYLKACIDEAMRLAPPVPGLLPREVTAPEGITVDGVFLPCGTVTGTPIYALHH
NPTYFPSPHAFDPERWLQPYTTADAIESARAAFTPFSIGPRGCIGKSVAYVELRLTVARLMWEFDVEEVVQEGKGVLWRE
GVAVREGEFRLLDHFTSRKEGPVVVFQRR*

>CYP567W8|601780|Morimp1

MGSTLSRLEVFTPYLTYPNLLSLAAAYCALYLVVEAFYNLFINPNSIYPGPKLAAVTKHWISVPWVAGNYPLIIKGLHEK
YGPVVRIGPNELSYSSASSWKDIYGHVGGRKVFTKSSFYNDGTEPSVVSERDPHKHGRLRRLLSNGFSARSLAEQEPVVH
QFVDKFIMQINKHVTNPKGDDMVKWYTFVAFDIIGDLAFGDPFGSLEDGKTHFWVENVSKGVAAGKWIFAFQKNWILSNL
IPQLVPKHLKIAREKHLNYCQGKIDKRLEEKNPKKDLLTVLIAKHNAGERFTPGEMRSNSQLIIVAGSETTATFLSGTSF
YLCRNPATYKKLVDEIRGAFSSYDEITGLATESLPYLKAVIDEGLRIYPPVPMGMPRVSPGETIDGKYVPEGTIVMTSSY
AATHSELNFHRPDDFVPERWIDPNCTDKKEASQPFLLGSRVCLGRSLALLEMRLILCKMLWSYDMEMVNPEQEWFQENTF
GMLWEKPKLPLRFIRREGVEVPPADY*

>CYP5093H7|604361|Morimp1

MYGELVTVWAGRRATIVIGDPQVACDLLDHRSAIYSSRPRFVIMGELFTNNDALLTMPHGDKWRKTRKIFHSGLLTKACN
SYKPIQVAESQRLVMDLVRSPENFGKHLERYAASLMVCVAYGQRVDNLEDPLIKRIYERMAYMATLNVPGAFWAESFPVL
KLIPDCLAPWKREVKRRAEESTQLLSELAFDVRDRMETGDAPACFTKTLWEKREENPEALSEREIAYATGSLFGAGSDTS
SSTLTSFILAMTCFPRVAAKAQEELDRVVGHDRSPTWCDAPNLPYCSAIIKETLRWRPVAVMGGTPHASIIDDHYNGHFI
PKGTTILGNLWAIHHNEKYFKDSHDFIPERYLSPCEDGTLPYPHRDGHSAFGWGRRICPGQKLAENSLFITITRILWGFN
ISKARDQITGKEIVPDIFAYTDGFNSKPQSFQCRIEPRCPRVLEVIEQESLLGEQFLEKYKSR*

>CYP6818A1|611846|Morimp1

MAILYELLETHRTSALVLLGVSVFFFARQATKSKAGPPFAGTWSSDFLSVYIYGCFKFVKYARSMLDDGYSKFPDTPFRV
PAPTSTKIIIPPKFVKELRNYPDSKLSFNAAIYDILGLKRVELQSLGEDNTPHQKMTRSQLTANLAKLIAPMLDESKYAF
QVEIGSAKEWKAVPLHSTIVHIIARTSSRVTVGEEICRNEDWLRASTGFSKDIFDNAFCMFLFPSFMRPLAVFLLPFRRR
IRCQRMESRKILGPTYTQRLADTEAGKPRVEDVFGWMVANALPKQRNINDLVDYHLALSLGAIQVPASTLVSIIYDLAAR
PEYIEPIREEIIQAVTEDGGQINMSTLAKLMKMDSFMKESQRFNAVLTSFSRKALQSFHLSDGTHIPAGSILGASAVSVH
FDPKYYENPDEFDGFRFYKMRQQKDAINKHLFVSVSPTEINFGYGAHACPGRFFVANKIKVLLVRMLMKYDFKYPEGESR
PRNITLGDATLTNTTRNLMMKERVDAPKWDFL*

>CYP51F1|616437|Morimp1

MGLLATLLEPLGPYYAELQKLGTPVIVVVGVLTFIVLSVVINVLNQLLFKDPTKPPVVFHYFPFFGSTVRYGMDPYAFFN
SCQEQYGDVFTYVMLGRKMTACLGPKGNDFVFNGKLSEVSAEEAYTHLTTPVFGDGVVYDVPNNILMEQKRFMKFGLTAE
TFKSYVPLIVEQVEDYTKKSKFFKGPKGTVSLSAIIPELTIFTAARSLQGKEVRDALDGSFAKLFHDLDMGFTPMNFLFP
WFPFPSNKRRDNAQRTMAKFYMSIIEKRRKEEASGVDHSDMIWNLMGRSYKDGTPVTDRNVAHMMIALLMAGQHTSMATI
TWMLLHVADQPKLVKDLYDEQVRVFGNGKGGIEPLSYEKLSECTILNHTIRETLRTHPPLHSIMRKVKAPMHVDGTNYVI
PKGHYILGAPGVSAMDEKYFKNPHVFDVSRWEGQKTEEEGEKMDFGFGMVSKGTASPYLPFGAGRHRCIGEQFANVQLSS
IMATFIRNFELGYPEGCGVPKPDYSSMIALPTPPCTISWVKRDP*

>CYP50308A1|621677|Morimp1

MAIVETLIYTVGSIAGAGFWKYGILAVMPFLWFWLNLRYGLGDFDIPTIGVPPGLLGPWIAPYNWMKNSPHLIREGMAKY

GARGLPFKIRSPSRWMVFVNKPTIIDELIRLPKNVVSSSAALDDIFFTQFTMYKDAASDPPFAEIVLKQLSGRCWEFMPL

AVEEVKMAWDEKTKIGEEWTDVPVWEVFIQLAARAVIRSIIGAPLCRNQELLDNIIAYAEGVIMHSELLEIIPATLRPFV

NKFIFRNKPLDVCMSYFGPIFQERRNIMKDGKYSGNVKPDDGFQWVIDTCPPDTTIERMVKKLLFIFTAGIHTTAVAGTH

CVHDIAAIPHFQGPAREEIAQELGAASTVKETKEAWAPMRKLDSVLRESARMNRTHLGTVMRKFMVPYTFSDGMEIPKGA

WVVGASSTIHRTNIYEDPDTFDGFRFSRMREEAGIGDRFQLTGTSREFLAWGGGAHACPGRFYAAMLLKVIFA*

>CYP51041A2|626598|Morimp1

MILEQKSLLPSSFEVSRPKLVAATVWYQTYYDVWMGGELAHQVKKLHKKYGKLLENLLLAFELRHREGPIVRVEPNELHF

SEPEVYHEIYSSNSRMIKDEFMYQCFGEGDSTFGFIDPNLSRRRREVLSPLFSRRNIISLQRLVIEKVEKLCEKLTEYHD

TSDAAGISSTFRCTTMDIITSFCFAESLNSLDYKGFRHLVLVGVKVALPMYWVFKHFRPVQWIFMHTPTWLSLLTSPETA

GIMGVQNSVSDHLDRFLANPKSPAIADHVIYHRLLGPENKRAGGSLPTRHGLFDETMALLIAGSDTVGNTLAVGGFHVLN

NPSVYEKLFNELKKNWPVLEKSLGYEELEKLPYLNAVIKESLRVSHGVVSALPRVVPPSGATIGGAHILGGTVVGMSSPY

VHTNEGIFPDAHRFIPERWLQPNSKHLEHWLVPFSRGPRMCLGVNLAWCELTIGFAHIFRKFDLRLHETTERDIRWKEYF

VPHFTGKRVQSFIKLRPS*

>CYP578X1|627595|Morimp1

MLASLSMVLILFVFFLLHSLYTHLRCPLRRIPSAHPIAPFTRLWILSRTLTGRRNRSIHAAHVKHGPVVRLGPTEISIND

PSLIKSVYARLDKSPWYYIFTNYGSKPLFSLIHGREHSQRKRLIANIYSNSTISSSPTLLANTRKILEERMTPILDTREV

HEVHELYNALTLDLITSYLFTQRHGTDFVRDEATRKRFLVDLYQIRRPYFSWASELPGLVKWVGRLTAGKVKIIPSWVDD

ANDQIEEWCLQMCDAAEADMPTEKGADDDCVYYRLRRNNALGRTDAASEILDHIGAGHETTATALTYATYNLSVSPERQA

RLRDELKTAENLWRFKDVDALPFLNSVVLETLRLNSPIPGSQPRVTTDGGCMVGEYCVPGGVTVAGQAYSLHRTFWADAE

VWRPERWVNNDEEGKEGERWLWMFGSGGRGCVGKWLAVYEMKMTLACIYANYETRIVDDEGIEMDDRYTTGPISNQLLVG

FHKL*

>CYP6683A1|630710|Morimp1

MGLLTIFSCLVALFLARWFTLLRRSLQAAKKTGLPYTCRPLYEGVLAYLLWMPILDYLIDYLSDWLATILDPSDYAGIYR

SWRFKAGYENHRVLGDVFLVVSPGSLVLEVADPHVCAQITDRRMDFVKDFDTYLPLRILGRNIVETEGETWKRHRRAVTP

QYTDAIHAFVWDQATIIGREMLSEWKHLEGADTPIKAPYTSRESRDLGTLANRVVMAAVFGVSLSLRTGKSPEALALSEF

ARSMEVINAGLLFVMIFPQLVMPYVPGIGHLSKAMNHVNLYMKNILDLHINAKDKPESSAKAGILDGLLKVLAPPGTVPT

EDQLSRSDVAGNMFALAFAASTTSAETMHFSMVLLALYPKVQLWVMEELDQLAVEYGQLNEGWKYQNVFPKLIRLSCVLM

ETLRLFPAVPMIPKCVEDRPQILSYKNSTFVLPPHTLIALSACALHRNPKVWGADAGEFRPERWLADNGKSLRPEPVEGC

FIGFSRGTRRCLGRRFAEAEYAAVLSEVLRNCRIELAPQKPGETVKEMSLRARKVLEMSRTGFTLRVGGKVMLRFVDRF*

>CYP567R1|638376|Morimp1

YLVTEAIYNRFLNPNKQYPGPLLAALTKHYIDIPWLTGRYPTHIHALHQRYGPVVRISPDELSYASASSWKDIYGTSSTR

KPFLKSAFYDDGTAPSIVSARDPATHRRIHRLLSPGFSARSIGEQEPLVQGHVDLFLSQVGRYATGSEGGNMVGWYTWVA

FNIIGGLAFGEPFGSLDDGEEAAFWVENVFGALAATAWIWTFKKNRLLQHLIPYLLPKSVKEARVKHLGYCMDKISQRLR

GKTARKDLLSVLVAQHTSGDSFTTEEIRGNAGNIIVAGSETTASFLCGVTWYLGRGPAAYKKLTDEIRSAFSSYEDINGR

ATEHLVYLKAVIEEGLRLYPPVGLGAPRVSPGETVDECWVSKGTVVMSSFYAAGRSEDNFHRPDDFLPERWIDPECTDKN

NGSQPFLLGSRVCIRRALVLLEIRLIMPKMLWAYNMELVDPAQEWLSENGFAVVWEKPALYVRYTRRQGVGS*

>CYP6470A1|660088|Morimp1

MGLVLGTAVPQPTALAYLTPQNIAVAIIAFLLEYIIKWRFFGPLRHIPGPWWSHYTQIPDGYHLMRGERALYIHSLHERY

GPTIRVGPTLVGVSGTAGVKMVYGTALKKPFTRNPQITAMFNFQRKPEADNIASFHLPGDALKRRRAYGNIFSRGNILAM

QDVFKKCFENYFAKLETLRTASPNGIVPMVRCFRALALDAITEVSFGGFYKGASDPDHQMKLLDNLMSANLVQLHLGNAI

YSLLKSIPWKRLDWVRSMEDFCRITSDSTALYLSNRAANPSQARKDSLSQLLSYGLPRAAVEGEALANVFAGTDTTGNST

AFIVCEVLKKPNVHRRVVEEVFNAFPEGVYRMGEVMEFRKVEEECRLLKWCIKEGMRRHSLAPGPALRVVPDDGVVVDGM

FIPGKVDIFAQSHTSTHDPVAFPQPEEFIPDRWENETSEMRTLWTVFGSGPRICIGENLAMMEMQMMLALLFRNYELVQV

PGTDMRITEFWLRSMKAGELHLKLVPRSV*

>CYP5142W1|661197|Morimp1

MEPQGGPRSLIITYGAYFIPPAVVLYYVALVIYRVFFHPLAKFPGPKKAAATGWYRSYYDVWMQGEMPRQIAVLHKKYGR

IVRFGPNHLHFSEPSAYHEIYANNSKLLKDSWVYNSFNASQSGFAMVDPSAAKGRRDVLSSLFSHRAISSMQGLLAENFD

LLCTRLAEHNTTSSAPINLSGAIRSTTVDIITTFCFAQSMHALDVPGFKAPILLGMRTSLRMIQVFKHFPLIQRIVLNFP

RPLLMFGEVAGFIDLSRRLESQVANFLKNPADLDKTPHPIIYNRLLDPDAKGSRAAPITQTALEHEAHTMLFAGADTVAL

TMTVGLYQLLKSGRQGELVEELIKAWPELDRAPKLEELEKLPFLSAVIKESLRMGSGVVSLLDRKVPKSGAVIDGEQIPG

GAVVAMGTTIVHTNEDIFPNSGSFIPERWTGPNGKELERYLVPFSKGARQCLGINLGYAELYIGFARLFRRFEITLDGTT

EEDMRWKDYFVPLFEEGHMKGFVKPREA*

>CYP51062A1|662304|Morimp1

MVLPWVGVSLATLLFLKTLLKKQEPIPTSFNGRPCEPLKLQKDPYILGIHGIVRAIKSNVAGRLPLSFIDLFNDLGHTFG

VKRFGVRSIMTRDHRNIQAMLATQFNDFGLAHERRAHQAILGTWGIFTQDGPEWKESRALLRPSFDRTSVADLSALETFV

TRMIAKINTSPGSDGYHEVDLQALLLELTMDASTDFLFGSPIGAQEGKVSAEGQMSFSEAFDVSQDVVSIRFTLNDLYWL

VNPKRFRIAITVVHKLVGHYVTRALQKHNSNSSADKPYTKDTSTSGKYIFLEALSQKTQNPQVLQDQILSVMMAGRDTTA

STPHGPFTSSPATRKYSRSCMVYLRCFLHEVLRMYPPVGINSRSALRTTTLPYGGGADGNALIVGPDAGTFRPERWGEEA

MRKVGWAWLPFNGGPRVCLGQQMALTMAGYVLVRVLQAFAEVRDNGVPGEEVRYAVKIIMVPGQGLKVKMR*

>CYP5268A9|667883|Morimp1

MQIVIVGAACAVLFFAYRAYQTYLSIQNEQSLKSSKTCGTVLPGPTPAPIIGNILQIPKTHTWIHFKQWADIYGPIYRFQ

VGSKNNIVVSTEKIANDLLRGRGNIYSSREFLYFATELLSKNLRPLLLPYNDTWRRGRKLMHTLGMPKVVDTFRPAQSLE

AKKLLHDMLEDPESYENHFERYAAGIIFRLVFGKAVETGREPYVREIYNIVHTVERVAAPGAYLCDSIPLLRYIPEFLSP

FKREAKWLHNREIKMFRELQNDIRVGIKNGSAPECLTRTFLENEGEFGMTSDESAYVIGTFFEAGSGTTAGAMLTFCLAM

CLHPHVQTRGQAEVDAVCGDRMPDFEDLPNLPYARAVMKEILRWRPVLAGGMPHELTKDDEYEGYKFSKGTVVHANLWAI

HRNPEQYPDPETFNPDRWVNPAYPTYKEPLSVYPNLQNFSAFGFGRRICPGMNIAERSLYLLTIRLLWGFQMNKKRDASG

NEIDIPYYDYDSGLSSRAKPFQFDCVARSPMRASVIRKSWDEAKATDPLRSRN*

>CYP6480B1|161519|Morimp1

MAGHLFMIILETLKKHAIGAIFLLFVSNVFYDLYLHPLARFPGPIWARITRLWWLRVVLSGKQHLKNYELHERYGPVVRI

TPNILSFSDHRAIKVIYGRGAGEFAKGDGFGNGFLPGGDPPVFFAKDPVVHSRVRKRIAHAYSMTSVAQMEPSVSALIEE

FSRNIDRFAERGEEFDLVEWVNHLTFDLISELGYGKKFGFMEKGGDVNGYQESMHHNSPMFPILAAFPTLAALLGVVIKY

LPPPESATGIGHLFGLAKRIIADRRASTSSSTNKKDLLGGFLASKTEDGSPISESSVFGEVMGTLIAGADTTAASIQGAS

LYLLQNPPALQHLVDELDQTNPVSYADTLPLEYFNAVLKETLRLSPSIGATFGRKVPVGGAEILPGIWVPGGFEVGINNW

VVGRDKTLYGDDAMEFRPERWLEKGNRTRFAEFEFGFGSGNRVCIGKNVAMMEICKTIPELFGKFELELMDPEDPWVEES

RLFLYKSGLRVRARRRVLKV*

>CYP573A23|247220|Morimp1

MASSPLSMLYSTKEGVFTSVVVIIILSIAKSIITSRYFHPLSQFPGPFIASFSRLWIVYWNLLGVEYLKEYDLHKKYGPV

IRITPTMLLVSDPKMLPEIYHRRADKADHYVTGSFGKTPSVFNIQPHEVHAAARKRIAQPYSMTAVKPMEGLIDARIVEW

ADKLENDFVKTGKKFDFAPWVTYFAYDVISEVAFGKSLGFVTKGIDIDGLIQSFHDGLPAFGFLCRLHPFTKWIKETWVA

DAYMIPKPGDNTGIGNIMTFRDNLLEERIKNNNDPDHKVEYKDLLQNFLAAKNNDGSPMPIEDIKAETLLVLLAGSDTTA

TSVQALMVDLLSNPRCLEKLIAEISEKDAAGLLSPVPKYDEVLEHLPYYCACLREAMRMTPAAPNIFPRVVSQGGLNLYG

KHVPAGFEVTENPWISQRDKTVYGEDSEIFRPERWLEDPKKAQEMDKYDFTWGFGTRVCLGKNIALMELHKAPVQFFRLF

TPELVNKEKPSTYVVAGGVSFHKDLWLNIKKRQV*

>CYP51073A1|522888|Morimp1

EIRSGATNRVLVGAELCRDAKFMAATTALATGIIATAGRLEMLPGFVRPVAAWWMRWGDRRLGAFLKYTDPIFAERRRRQ

GTWGMGEKPDDAFQWILEAAPKGASLRDMAFTLIFLNLASIHTTAGTLTQVLFDLCVHPHYQPLLREEAEEALKDGLSGS

ALGRMKRLDSVIRETLRMSPTNVAVTPRKVITSHTFSDGTRVGRGSWVAEPVMHANRSGACYDRPDVYDGFRFVDMAGGR

NQCVSTSVEFLSWGHGASACPGRFFAVAEMKIVLAYILCNYEISFQPGMVERPKSSTVGIFQVPNTSVELELRKRK

>CYP6001C27|574980|Morimp1

MASNGASSSSAGIKPPPPLAKQTRKDVDSSLDKFSALINASNRPLPNRYGNGTDRSPEAEQTGIWTDISTLRKGGFLLES

VATLWQLVQQKRRGGPVDDKTMIMERVIQLTSRLPPTSKVRISLTTHQVDQLWDSLQHPPLSYLGSQFHYRQPDGSYNNV

MYPQLGAAGSPYARSVKPMTKMPAAPPDPYLVFDSIFSRGKNDENYREHDNNVSSMLFYTASIIIHDLFRTNRDDANISD

ASSYLDLAPLYGNNKDDLDKMRTFVDGKIKPDSFSEKRLLAFPPGVSVMVIMFGRFHNYVAEQLKAINEGGRFDLKYPRR

PVPDKTREQIHAEALANQDYELFQTARLVTCGLYINFILNDYLRTIVNLNRVDTTWTLDPRFDPDKIYNPDGTPAGVGNM

VSVEFNLVYRWHSCISKRDDAWTKEFYAGLFPGKNPDDVNMREFLVGVNQWESKIPSDPVKRTFGGLSRQGDGSFRDDDL

VKILTESIEDVAGAFGARNVPHVLRLVEVLGIEQCRKWRIASLNEFREFFALKPHKTFEDINPDPKIADTLRQLYDHPDY

VELYPGLVAESDKKPMVPGVGIGPTYTISRAILSDAVTLVRADRFYTVDYTAGALTNWGLAEASSDVNVLHGCVGYKLIL

KAFPNHFKSNSVYAMYPLTIPEENRRILTSLNTVDQFDFSRPKFTPVRIPISSYAATKQILNDSTNFKVTWGEGFDFIMK

ADFMLSGDTSSNAQQKQFVRERFYLKDVDWKAQIKQFYVETTRELLKQKSYHIGGRYMVDAIRDIGNMAVTHFAATIFNL

PMKSEDHPKGIYTEQELYMILCVMFIVIFFDVDPSKSFPLRQAGYGVVRQYGSLVELQVKSIKNWGWLQGIWDPTNVRGR

NNSALSSYGHHMIKRLLDGGKSAYDVTWSYIVPVAGASAPNQGQIFAQVLDFYLEEKNAHHLAEIQRLAALDTDEAWETI

KKYALEGGRLAGTFGLYRRCSSDVTINDYDRILHLKDGDLVFVNFITASRDKDVFPDPEVIKLDRPESLYMQYGVGPHEC

LGKAANIIGLTTMLMEFGKLKGLRRASGKPGILKAIPKPGGFKVYMKEDWSGFWPFPTSMKIEFDELS*

>CYP539A37|591301|Morimp1

MLLDALQQSAPGIALTLLVASILAFCFAKIRQNLRIRKLGGRAPNINTYPFLFGLDVILDTMRMAWKNQNFELWRRRFDN

SGHTVEMELLGQRIILTEDPENIKAILAAQFSDYGKGKDFHRQWLPFLGNSIFTTDGSEWHSSRQLIRPQFIKDRISDLE

TFERNITHTMSLIPHDGSAVDIKDLFYRYTLDIATEFLLGNTVNSLGSPRAEFAVAFAELQKFMNDISRVGPAENLFPRG

KFNKNLAILNSFVEPYVEQTLQLKPSELKDKTEKNYNFLHALAEFTRDKRMLRDQLVAVLLAARDTTAATLSWTLHELSK

KPEVVQKLREEILRSVGPDNNPTYSDLKNMKYLSAILNETLRLYPAVPFNVRTSLKDTYLPRGAGVDGNEPVGVPKDTPI

AYSTLTMQRREDIFGPSVNEFDPDRWSKWVPKSWQYIPFNGGPRICIGQQFALTEMSYTIVRLFQEFEAVEPRMTKVQWM

QTEIVGSPGEDVVLSFKPAKC*

>CYP52AX1|591982|Morimp1

MAYTTLLWVLFALLLPYYFRPLIHNLLNHRANAKYAREHNCHPPKVHLTKTVFPFSIVNVYQFARAAASKRHINHFLDQV

QRFGNTRVYSSPLFIGHAIVSIEPEIIKTILATNFADWELGKVRHEAFSALLGDGIFTSDGKVWEHARALLRPQFVKDQI

SDLNDLEIHFQDLLSILTAPGEGQVVELHKLFLSYTMDTSTSSLFGESVFSLRRRGGDGVGESFQDDFDYAQLAMGYRLV

VWGYHLLYRPRRLPKALKRIHDYVDEFVDRAIQRQRVEKKEEGKYVFLEELAKATQDRKVLRDQTLSVMIAGRDTTATLL

SWVFWLLSKNPKVWGKLRQEVLYEVGEELPNYQQLKDCRYLKWVINEALRLYPVVPINFRYCNKNTTLPIGGGPSGDLPM

FVPKGSRVVYSVYALHRRTDIYGADADEFRPERWGEMTPRAWEYLPFNGGPRICIGQQMALITASYTIVRMVQEFRAVED

ADIVREQVLTNIGLTLSPANGTPVRLYRDVE*

>CYP52AW1|604750|Morimp1

MGMSTLIVQISAGLGLYIICLTFLALRRRFKNARFSREHGCLPPSQAPTDWTFNISRPFILSKVRKAHKFMPTQQESFHR

NGNTFAFSIAGKRLIMTCEPENMKAILATQFKDFELGKLRHDNWGEVLGDGIFTVDGHQWEHSRAMLKPQFNKNQITDLE

DLETHVQNLFSRLSLAENGGSVLNLHEYFLNFTLDFSTAFLFGKSVGSQLCQGQTSKFAQDLDYAQKVISFRVSLENFWW

LYRPKKFTKAVADVQAFIGESVQQALDKQKSEKGKTSEKYVFLEALARETQDPVVLRDQMMNILVAGRDTTAGFLSWIVY

SLARNPRVWDILRREILTDFGRDTPTYQQLKDAKYLKWVLNEVLRLYPLVPTNFRYANKNTTLPLGGGPDGLSPIFVEKG
ERCVYSVYSAHRRKDIYGEDADIFRPERWGEGKPRGWEFLPFNGGPRICLGQQLALTVASYVIVRMVQNFERIENADTNK
EELGDLTLTFMPYPGTLVRLFKDPET*
>CYP505A66|568596|Morimp1
MTTPIPAPPGFPIIGNLADLDSELPTKTLRHFADIYGPIYRFDFGGGATKVVISSQEIVEEACNEERFIKVIQGGLNQVR
NGVGDGLFTAHHDEKNWGIAHRILMPAFGPLSVQGMFNEMHDIASQLVMKWARHGPDHKIVATDDFTRLTLDTIALCAMD
YRFNSFYTDELHPFVGAMGDFLSESGRRSQRPAIANLLMRGSSQKYESDIALMKNVSDEVIKRRKEKPTGKKDLLNAMLN
GKDPVTGEGMSNESIINNMITFLIAGHETTSGMLSFAFYLLIKNSAAYRTAQQEVDDVCGKGPIKVEHISKLKYLNAVLR
ETLRLHPTAPAFSMCPKPGVEEHPTLCGGKYAIGKNEPIICLLPKVHTDPAVYGEDAEEFKPERMLDEAFEKLPKYAWKP
FGNGMRGCIGRAFAWQEALLVVAILLQNFNFQLDDPSYQLKLKQTLTVKPKDFKMRATLRDGIDATMLEKKLQAGSAPTA
EDSTLKETLNKPSNLKPMSIFYGSNTGTCEALAARLAKDAIQHGFDAKVDPLDSATDKLPKDQPVVIITASYEGAPPDNA
SHFVEWLKSLKEDELKGVQFAVFGCGHRDWQATFQKIPSLVDELIGKWGGERIVNKGTADAANGDMFTDFDGWEDTQYWP
AVEKNYGGTATSPDAVSTPSIEVTVSSQSRSTNLKQDVRGALLMENRLLTAPGEPEKRHVEIKLPTDMVYKAGDYLAILP
TNPKNNIRRTVARFGLAWDAVLNIKANGPTVLPTNVNITAADVLSNYVELAQPATKKDVLAIAKAAADEKTRTALENLAG
DLFNVEVSQKRLSPLDILEKYPSVAFTFSEFLAILPPMRVRQYSISSSPLYAPDSCTLTFAVLDQEAISGQGRYVGVASN
YLAHLEAGDHIQVSVRASAHPFHLPLDPVSTPLIMVCAGSGLAPFRGFIQERAAQIGAGRKLARALLFVGCRGKGDTLYD
EEFKKWSAMGAVDIRYAFSRETERSEGCKYVQDRLWHDRVEAVELFDEGAKVFVCGSREIGEAVKVTCKKMYSEKAAALG
KPKSEEEVEEWFSNLRNARYATDVFA*

Pyronema confluens CBS100304

>CYP504A40|614|Pyrco1
MNTLTVAATAVVTVFYLLYRYAYSTNTPKIKGLPEVPGLPFLGSLLAIGDCHARKALEWSKTYGPVFQVRLGSRRVVFAN
TFQSVSHLWITQQSALISRPELYTFHNVVSSSQGYTIGTSPWSESVKRRRKAAATALNRPSVASYMPIIDLESAASIREL
LDDTHYGQAAIDPSPYFQRFALNTSLTLNYGFRIKGNINDEQLKEIIEVERRVSNLRSTSNNWQDYLPILRWFGNKNGEA
EEMRRRRDRYMENLLSELKERIAKGTDKPCITGNVLKDPETKLNDDELKSICLTMVSAGLDTVPGNLIMGLAYLSTPHGQ
EIQQKAYAAIMDQYPDHSAWEACIKEEKVKYITALVKEVLRFWTVIPICLPRVNIQPLEFNGVQIPAGTTFLMNAYAADY
DPTHFLDPERFLPERYLTDEVTGTSHFAFGAGSRQCIGTHLANRELYTAFVRLIVAFEILPSDDAKDHPIIDALECNRMM
TGLTTDPKDFKLKLVARDRNRLEEWLMGSEYRTREL*
>CYP50147B1|1052|Pyrco1
MLLDPQLLERLSKEKYTAILTVVAGFTAILATKWLFTAPDKRNYVTLPAGPFSYLKGQLRYLLHADDMIREGIAQYGENT
PFWLPSMIGPMLLAPRYLMNEIKNHPHLSFAHFLKATFSGKHTGMGEPFADPQYIGIIKGKLTKALNSFTPEIEEEIIEA
LKDRIPLDEEWTPHIAFPGFCQSIVARSSARIFIGAELCRDQRWLHIATRYTAQMVGVTLFLKLWPEFLKPFSKYLSPSW
WRAAAMYSRARILIAEVVTKRQKELASAGSEAPPRMDGIQWALDLGMDLERMTEFEITMAIASVHNTAVALTQLVLDLCE
RREYIQILREEIREVLGDGPARNNKGINGLQKMDSFLKESQRTSPPQHATMQRRAMKDIEFSDGTKLKKGEFVMFDSDSV
LSDARYYENPEVFDGLRFYNLRQHAENEATAQYANTNEQFFVFGNGKSACPGRFFAGHELKLILVYLLRMYDFKFPDGQT
KRPANLHYEDIIIPAKGQQILFRRRRV*
>CYP675Q1|1469|Pyrco1
MKGPSVLSSQPPLGIIYLHPSFPRFYARSLTFIYLPPLSSSYFLSCTFYLRFRFCIVRVIAAMLLSALLSNPLFYPIAFV
ISVTGWVVYQRVFSPYAKIPGPFWASITRFWYLNRIIEEDMHQYTKQLHKKYGPLVRIAPNEVSVSEPAAMKQIYAINAG
YTKTDFYPTQAPNLSPHGDSFTQLDEKKHTYRRRMIQGIFNLSSILESEKYIDVCTETYMKVLSDHAESGAVMDMSEWMQ
WYAIDVIGELFFGKMFGFMNERRDVGGYIGAVDIILPHAIRMGVLYNWMRPLQILLVPFSASLRHGIKIFGELGAESKRL
VDERWGKKSARTDMLSKLIQVAEAKAPEFDITDVYTESYTAIFAGSDTTAIVLRTAVYHLCRNPDAMAKLQAEIDEAQKD

GRLSPVITYAEAIKLPYLMAVVKESMRVHPSISLTFPRHVPKGGREICGHFFPEGCRVGVNPYVLHYQQSIFGEDAEDYN
PDRWFRADAKTMESYLFQFGSGSRTCIGKNIALAEIYKLIPQIFRAFNIELVDPKKDWVEHNTWFVKQTGIDCRLSKRQS
A*

>CYP50127C1|1778|Pyrco1
MNKCIRTKRLPFYPSTLYNRYNRYTLPLDPAQSAFITADPANIRRLMTDTKMIGVSPGRKRFRYLLGTGFFVLDGKGWSG
PRSQISHLFSHPNLLDFTALEERIQRILFPVLIPGHTVNLTPAVEKVSLDESLNFLLGCDTTAPDTEEFVKAYEDGLWYI
NLSRVVKIINWVRPAGYRKAARIVRNWTDQRIDEASYREGTFLKAIEYCLPEEKRGHVLNSLFAGKDTTAAVVIWAIWNL
VRRVDIMHKLREEVAATVGQDIPTFEKLQEMKLLRSVMNETMRLTPPVPMTNRESHVPVVLPVGGGNGSLPLLVPEGKTV
MLDIFSMQRRRDIWGDDAEEWRPERWIENEALESQSVAACTFIPFGVGPRNCLGAGMAWNTAGYTIVRMLQEFKEIRKKE
GESDEATFTSAPQPMPGNGVWITLGSRTDETYFDRRLC*

>CYP671K3|1779|Pyrco1
MIVDTTTFLLGFGAIFSGLAFHQAILPHEIDFRLKSLSLGYLASFLSLFYLLIRLSIPSPLLLTSLTFLLVNFTLTLSIL
THRLFFHRLHNFPGPFLAKVTKWYSVYLSVKNYQHYKEVEKLHQRYGDFVRTGPREISIRKVEALLEIYGAKSGCWKATN
YGMNSDDPKGSLHLSRGKTLHTHRRKAWDRAFSISALSSYSSSVSSLTSLLISQLRSQNGAAVDATAWSNYFSFDIMGII
GFGKSWGMLETGEYHDAIKKLHESMFILAVLWQTPWVIRGLDMIPGAARAMKGFRGWCKEQMEEKQKALKDEKFDGKPRN
VMEWLLTDEFTMSLPTEAINDDSRLVIIAGSDTISAALAHAIYLMIKNPGVYMKLQRLLDKEFPGGDADFDYTLLSDVPF
LDGIINETLRLKPSVPGGLTRITPKEGITIDGTHIPGDVIVSVPIHSIHRDPRYFSQPLEFIPERWTSESPELVKDKRAF
VPFSMGSQVCVAKGLAMMELRMALARIALAFDLEFEDGETGRRIEEEVRDTFTLTMPRMGVRFLPRD*

>CYP5945B1|2716|Pyrco1
MPKDIASMLSPPTFLAIGLALLAQNTIWGINEAPTRTIFYLWSALQIAIFILLRNIPTYIFINTIFHSVFLTTTAIRRLY
FHPLSSWPGHKRAALTKLYEAYLYFTGINALEIRRLHRSHGDFLRTGPNEVAINNVEALQLYSRQNFEVIRGPFYKVSAI
VGDVNLISTRESSRHKTLRGIWEQAFKTNAVKEYSPRVELHVDRFIENLKATEGKPFDCVPVICNMTFDIMADLGFGKDY
GMQLGIGDPSYMGYMHKYMRIIAMTGALPTLSDLVPLLPQHADTKAFQKKGRVMMEERISLGKNRQDIFNHLLSADSESG
RKLSHSELDSNAQLIIIAGADTTSSVLSNLFRELALNPEIQERLYQEIMDAKIRNGGTKFDCENTKSLPYLQAVIDESLR
LWPPVPAGAQAQTGPSGATVAGRYIPPFTGVRVHHLSMLTDERYFPQGSRFWPERWLGENRQEGVKDIRAFVPFSYGPHV
CIGKHLAYNEMRLAVARTVEGFKVTLGEGFCEERYRDEWRDYFTVMIGGIEMVFRPRD*

>CYP567Q1|3224|Pyrco1
MAPTSFHVQDLLSLPTWALACLPVALIATYFLANGIYNVYFHPLANIPGPKSASFAQYYFTKIWLSGKYPYYIKSLHDKY
GPVVRVGPSQVSFNTPSSWKEIYGHIGGRKQFLKSDFYEGDGRPKNIVSSRSIAEHGAMRKMLSNAFSAKALADQEETIQ
SYVDTLVKQVAKHATGKPHGEEMVKWYNWITFDIIGDLAFGDPFGCLKAGVEHTWVSSIMDATAAGSYYNAYIKYLGNSP
IEKWIKKLFVPSHLFAKRQNHFGFAKDKVMRRVEKGANARKDFMSNILNEKETQGISNDTLTVQSALLIVAGSETTATFL
SGVTYYLCRSPKAYKNLIDEIRSTFSSYDEINARATEHCKYLKAVIEEGLRIYPPVPIGMPRFSPGETVDGVYLPQGTEC
FTSSWAATHSEDNFHKPYEFIPERWIDKDCTDKKDASQPFLLGYPVLALVEMRLILAKLLWSYDMQLKDQTLDWVKDSEC
NLLWRKPDMHVDFTRRAGVYIPPVDDVKEE*

>CYP61A1|3756|Pyrco1
MDSMAYNPPAASAVESAEQPIQTLIGGGFGAVSKSKSLEGVSTWQIVLTILVLSITYDQVKYIWNKGSIAGPAFKIPFMGPF
MESVDPKFSKYLEKWNSGPLSCVSVFHKFVVIASTRDLARKVFNSPMYVNPCVVDIAKKILRPTNWVFLDGKQHVDYRKG
LNGLFNRKALAGYLPKQEEIYDHYFKRWIELSKDGKPRQYMGEFRDINCAVSLRTFCGRYITDEAVADISENYYKITAAL
ELVNFPIILPYTKTWYGKKCADYVLEEFSKCAKMSKIAMEKREEPQCTMDYWVKSMIDSREYEKLSAEEKEDYKGPKPMV
IRTFSDIEIAMTIFTFLFASQDASSSASTWQFQILADRPDILAKVREEQLRVRDGDPYKRLDIDMVDKMVYTRAVVKEQL
RYRPPVLMVPYEVKKAFNVTPEYRVPKGAMIIPTTYPALHDPEVYVDPESFNPDRWLEGGEAEAATKNWLVFGTGPHVCL
GQHYAIMNFMSMIGKAAMFLDWEHMPTPLSEEIKVFATIFPQDDCYLTFKERMPLEKPSS*

>CYP51F1|3971|Pyrco1

MGILTELLVPLQPLTGDLSKLSTPVFVAVATAAFVVLSVVLNVVQQIVFKDKGKPPVVFHFFPFFGSTVVYGMDPYAFFA

SNQEKYGDVFTFILLGKKMTVCLGPNGNDFVLNGKLAEVSAEEAYTHLTTPVFGEGVVYDCPNHRLMEQKKFMKFGLTTE

TFKSYVPLIVEQVEDYIKKSKYFKGPTGSVPLMNIIPEVTIFTASRSLQGKEVRDALDGSFAALYHDLDLGFNPMNFMFP

WFPFPGNKRRDAAQRKMARFYMDLIDKRRKDPNAASKEKDMLWNLMDRSYKDGKEISDREVAHMMIALLMAGQHTSMATT

TWLLLHLAEKPEVVAELWAEQQAICGQDPRPLAYEDLAKMPLLNNVIREVLRMHPPIHSIIRKVKSPMHVKSQNYVIPAG

YHVLAAPGASAMDEKYFKNANEFDPHRWDEVEEEDAGEKFDFGYGLVSKGTASPYLPFGAGRHRCIGEQFANVQLGSIIA

TFVRTFTFSLPGDGKVPGPDYTSMITLPTQPAAINWKKRNP*

>CYP6713D1|6163|Pyrco1

MELDFILLQLGAIYTLVLSILTYIFSRGSGLSHGLFSTFLISSAFNITLVISIGVHRIFFHRLRHFPGPSGAKISSFWHI

YQLWGNNKGHLLAKSMHDEYGNVVRYGPRELSITLVEAIPAIYSSSSPCKKSPFYAILGKDKSVSVFQMRDPALHRARRK

GWDKAFNGSSIAMYRDKVHDCISTMIGQFYSRKTVDFNHWASYLAFDIIGQAGFSKSYGMLETGEMHEAVLCQKDVTAYY

AMAQSIPWFAALVMNFPERWSPLKPNLEWCANEVNEKIKKLTSDKPTDIMSILLRDEHWETLEMGAIHDESRLVIAAGSE

TVVTAMVGIIFHLAQSPRVYKKLCKILDEKFPGGDKDYVYSPGDLILYVEDVINEVLRIHPSIITGIPRVTPPEGLTIGD

HFIPGDTVVSVPIYCMHRDARFFQRPLEFIPERWSDQPELVIDKRAFTPFGIGDYSCAGKSLALMEMRMFVARMCLNFEW

CLAEGLTEEKYVANQRMMFGMQLGPTPITFTPRKRM*

>CYP573A24|6298|Pyrco1

MAPGFYCSLKGTTDMASTLLDQVYLAYQTATPIQIAATVIALITASILHSIIKTRYFHPLSQFPGPFWASITRWWIVYHL

LKGDEYSILYELHKKYGPVFRVTPTMLMCSDPKMLPVIYHRRADKSNHYGPGGFGKTPGVFNIQSHDDHVVARKLIASPY

SMTNLKRTEPLIDGRVVEWISKLDKEFATTGKTMEYSSWSSYFTYDVISELAFGKPLGFVEKGYDIDDLIVSFHKGALSF

NVMARLHPLTNFFKKTGLGALLLPKPGDGSGVGNVMKIRDDLIASRLKELENPLASPRKDMLQSFLDARSITHDDGSSLD

MENLKAEVLLVLLGGADTTATAFEALMYYLMTNPGCFKKVLAEIDNAPLSAIPQYDEVLQHCPYYVACVKEALRLCPPTP

NMFPRVVPPGGLNLYGKFAPEGMEMSCNAFVTQRNTDMYGEDALEYKPERWLDTEKAAEFEKFDFTFGYGTRKCMGMNIS

FMELYKAPLQLFKGYAPRLVKKDGKEPRVIVVGGMARWENLYVAIEKRKL*

>CYP6713E1|7023|Pyrco1

MDPLQYLNSKFVQEFTFPRQLAISSILALAAHYFTLSIELDYLVIHLTTLYLVVYVCLIRLYSAYTDIYSAVISATKLAA

VFNATLITSIVIHRLFLHRTRRIPGPFLARLSPLWIVSKSWKKRKGYLIVEALHKQHGDIIRIAPRQLSINNVQAITAIY

GGNSPCTKTTFYDHRFPEHQSSLFSQRDRSTHQLRLRGWEKAFSPLSIAAYEPTMRNCQRLFLDQLAEAGTIDTTMWSNF

LAFDIIGQVGLGKTWGLLEAKEFPPAIRKIKDSQTGFWLAGYMGWLMRLAIVLPFAGRFSGAMAFAAWCKEAVEEQIREF

EQDREKEVGRVKTIMRTLLEDERAGLGRMHRMAHWDDGGLLFAAGSETTPTAFTGAMWYLAMNKDKLQKLQAALDDAFPN

GDEEVTYAKVQAIPYVSAVLYETLRLQPPVAGVIPRNTPPQGITINGQYIPGNTTVGVPVWTIHRDERYFERPLEFIPER

WTKEMPGLVKDRRAYMPWSIGPTACAGKPLANMSLKLMISRICLSFDFDFAEGQDPRAYKEDMKENFGFQVPALRMTFTP

RK*

>CYP53A58|7135|Pyrco1

MAYTTYILIGLLPLVYYLLPYIVDAKKLRRFNAPFPAAFSDFWLFWQARKGVRHFAVHDMHKKYGKFVRIQPEHISVADP

AAIPIVYGHGTGFLKSDYYDAFVSIQRGLFNTRDRAEHTRKRKTISHVFSTKSVLQFEPYIHHNLELLAKQFNKMSDSPD

LSGSYHKLDILHWTNYLAFDIISDLTFGSPFGMLESGKDQAFVKDPATGKVTTAPAIQVLNRRGEVSGTLGCAPWVKQFA

KYLPDKFFTEGVKAVEDLAGIAIARVSDRLDHGIGNEREDLLKKLMDGRDEHGKPLGRKETEAEALTMLIAGSDTTSNTL

CSLMYWVLRTPGVLESLQKELDEALQGDWSVPNYAAIKDCKYLRAVINETLRIHSTSSLGLPRVVPPKGYTVCGEFFEGG

TVLSVPSYTIHHSDEIWGDASAFRPERWFNLTELQKKSFIPFSVGPRACVGQNVAELEMITIVATMFSGWDWKFAEGEKQ

GLPGAPLDTVEGFLRKPLGLNVGIKRRRA*

>CYP532T1|7741|Pyrco1

MAASALVTQLLVRAHQLLNFAESNQLATISTVLGAFILWFLSARYNDLSDLPGPFWASITNLYRFFDQWTWSPHLNHISL
HKRHGTFLRYGPNVVSISSPDAIPIIYGVNKGFLKSEFYPVQQPISGGRKVEGMFNTTSDKIHAAFRKPVANMYAQSTLI
GYEPLVDSTIRMLQKRLDEFADGRDLDLGQWLQYFAFDVIGEMTFSKRLGFLETGGDVEGVIVGIEDSLRYSALIGQMPW
LDYILRTNPIIRLISAPTSYVVQFARNRLSERLASGSEKPTNDFLSKFLAANEKDPVSAPQSHVFAWTVSNVNAGSDTTA
ISLRSIFYYLLKNPQSMRRLREEIDWAVKEALIRPGELVSWATSQQMPYLEAVIMEGMRLHPVTGTILERVVPKGGREIC
GRFFKEGTIVGISPWVVQRDRTVYGEDAELWRPERWTEAGEKERKRMDGAFLGFGAGSRTCIGKNISRLEMFKLVPWMVG
NYEMELADPKKEWDLLNAWFVKQTGVIVKLRKRDWSA*

>CYP6742B1|8676|Pyrco1

MATFNSHFDLTFALRRLHLDSLISHTTPLQAILTLAFIYVSSTIIYRLYFHPLRHIPGPRLAAITDWYGIYLYLSNGLWE
EGENQRFLHRRYGPVVRYGPNSVICSTISSLPLIYHRRADKTDFYATSFGRNTSFTSLLHADHVIAKKRLAYGYSMTAIK
QWEHEIDVRVSEWIQQLDTRYAKTGEQLWFYKAVQFLVSDIVTEICFGEALGCVKEWTDVRNLIASFARSAPLLQVTGRI
PTLGRFLRKFNLYRPKPGDKTGFGMLLAEVERAAGKYKDLDIDTQNLEKNQKAGLFFRFMTTTAQGGETMTLEQAKWEAI
TAMVAGSRTVADMVSPTILNILKNPRCMKRLQEELDTITEEGTVGYETAMGLPYFSAVLKEGLRSSAQAFQMPRLSPTEG
LVIEGVSIPAGVSVSSSPMSVMFDEDLYGADARVFRPERYLEADEETLKKWEKYNMRFGYGTRTCIGKNIAMMEVAKTMV
EFFRVFDPEVVWNDKKLEAHMTFKVRLKRRIARSTVCGAA*

>CYP6713C1|8965|Pyrco1

MAVKFIHHLLYSVFALIFHRIILYREVDFHIFPILGLFLFSYSSLTYTFHHFGHYTLQQSLLKSTSLASTFLITLTISIL
LHRTFFHRLRRFPGPFWARITKFYAVRKAWIVPRSYEQTAALHAKYGNIVRVGPRELSILNAAAIPAIYSSTTPCIRGPF
HNLSAAEGKGSLFNTRNKPTHAQRRRAWDRAMNTTSIASYTGRVDGLVTLLLQRLSEANGEPMDWTKMARYLAFDIIGEI
GLGQSFGGLQKMETHPAVEELEQAVWWFGVPGLVPWLVRAMAEIPGLSGSMGAFIKWAEEQMQVKLEAGFKTDKEQKDIL
SCLLSDEVCGYGKMSRESSADDTRLVVIAGSDTTSAALSSLFFYLAHQPTIYAKLHTTLLSAFPSGQWNPHTPIPYLDAV
INETLRLQPPVPSGLVRTTPKEGLTIDGTFIPGDIHVSVPTWSIHRDPRYWDRADEFLPERWLEGEASEGRKAWIPFTTG
AYACAGKSLAYLEMRMVAARVVMRFRCELTKEMEDAAAWERGIRDLFMGQNPRLWLKFQERL*

>CYP5047B1|9248|Pyrco1

MFGTLTSQLLSESGAFVRNHWLLVIIGILIGNIFRLISRYWTSPLRAERIPGPFLAGFTHFYRLYYADITRDWHDQLVKL
HEKYGPIVWIAPYEVSVSDPKLRSVLYGFADERKADSFFRKSRSFETGLFNEDFNFVFETDPARARLGKYALSHTYSEKG
LIKLEHHFDEAVDEFSNGFKEHVASNDKSHCFSDWTHFFMFDLGTLLMGGYSRGLLRAGKDEHDAIWAIRLIFDVVGSLV
PVPLSTYVTTRPIRKFLLNAQIEQLYRWAICYDKDKGEAHKMDRINEIADTNPDNFMSKFRDGEKKMRKLFPKGNWTEAI
TNNAFFIYAGGMVASSALPLVVRLIYSHPRVLAKIREELSTLDREIRISDITHKNGQSSLPYLEASILEALRLSPTFGLS
LGRTVPSIGCRLNEYFIPPTYTVSMSGWSVNVDESYFGPDAKEFKPERWLGNHPTEIAKDGSGLPRTMRNYLEAGWFTFG
AGSRICIGRHYSEIAFAKFIGNLVRTFDLEITEPGYVWHGLIQHTEKMMVKAKLRGDAPNPMRKTEVLVV*

>CYP6529A5|10256|Pyrco1

MSLTSAVTAIGNLPTNQLASGAAILGVLSHWTYFIHDERDRQAWEYLLTLIGTPPVLVSLVHFAGGKTIGDAGYIIAVAT
GSFLVALYGSIIVYRLWFHPLRKFPGPPMARLTKVYYAYKYSQSKGRYYEVQKEWFAKYGNVVRTGPNEITTIDPNALQV
LSKTNKGTWYVFGEPTPSIQLVRDITTHSIRRRVWDKALSAKAIEGYLPHIRRHISLLLPQLAGEVDISRFFSYYAFDTM
GVITYGRSFNMLEKAGRDGSDYFLRMTHASMRTLGLLAHIPWTLVLLENLGAAGKDHTNFLKWCDEISQERKQRGKGDDI
FGVLLDVEPQNVGQHHIPLAGDSRTAIVAGSDTTASTLISLIAYLCSEPLVRQKLQEDVDKHGAESEYLEACINEALRLN
PAVPSGTPRYSPPEGLVLEDGRRVPGGINMLFPLHAAQRDPRWFDSPEEFLPERWINPGPEQFARMNTVFQPFWVGRYQC
AGKALAMTQLKMVTAAVVQKYAFHFKEGWTIERAMEGAIDTFTMEMKSVWCVFSERI*

>CYP503S1|10419|Pyrco1

MANETNFTNPLLGLKLPESIELPKIFLSLFGIWFTFNFVEALFTRDVFGSKAPYVGPSVQWLPGFVKRLLFHANASRIIY
TGYGKFKNSIFRVMRNDYDLVVLSTKYLEELRALPDTKVSSIRAHIMNLAGKYSTTDILIESPLHTQTLQTRLTPNLGLL

VEPMQKELLSTINSSFSTITDTWTPLAVFPTILNGIARVSASVFVSPLAENAAWLHGSVHYTENLFLVVYILKLIPSFLW
PFVSPCIPAYWRIHYYLRSAKAMVSPIVLERRKAAAENPDWKPPFDFLQWMMEEAKTPEEQNPEKLAHRLLLVSLASIHT
TTMCTTHVLYDLAQYPEYIEPLRQEVREAVESEGGWNKQTINRLKLMDSFIRESQRFNPPSLLSFHRVVIEPVTLADGTF
LPKNTQICMPSGPLGMDSTVLIGDKPPTEFDGYRYFNERKKEGGGHRHQFTMTGKDHLHFGAGRYACPGRFLAANEIKML
LGTLLQEGYEWKFKEGTGRPKNWNMEEKIVPHTTAELLFRKVKQ*

>CYP51050A1|10420|Pyrco1

MALQAFFDAFQTAFTGFNVWTAIGYAIIFVIARQFVKQHGDQLRKVPRVGMDPGWFSMSKARRNFLAYGPELIEEGYTKF
KHSMFRVQTNDMERLIISTSFIPELSRLPEETLSVAAGISVRHLGKYTALDFVDHNTIQVDACRIHLTNDIDHLTPALAD
ELRYGMNIDLPSDGKDWVSVNAFSVLLRLVARSSARVFVGKPLCRDEVWLETSIGFAGEVFLTATDIRPYPNFLRPFVAP
FLKSRKRLLKRLDVAAEKIVSVMKSYREGAMDRKDMPAALWMMDVAGKGSKEQTDPKDLALKQLFLSLAALHTTSTHLLQ
AVYDLCANPEYVQELKKEIKDVLGDDVWGSWNNAKSLKLQKMDSFLKESQRVNHPGMFSFNRVVMKPTTLSDNTKIPPGT
LIGVATKCIADDPEFYENPKTFDPWRFYKLAQDASSAEEKGSTAFASTSPTSMVFGHGRRACPGRFFAAHQLKLFLVGLL
TEYEFKFPKKMDRPKNLAYGEHIVPDRAQEIAFRKI*

>CYP6637A1|10579|Pyrco1

MSSLPSVPLLIHTIPVLDYIPDNRYVRYATLVSIASFCYCFVLFVYRLYFHPLAKFPGPFWARTTQFWLVKTLISGQNAQ
IVKAAHDKYGPIIRLTPNDLSFATTSSWRDIYGKDTGRKQFTKTEWYQMVNSGFGEHGIGSEPDIDKHAKKRKLLAPIFS
KQAVRDFEKLLVATFDKFTRIVQLEGSKPEGIDMSEWIHKLMYDLMADLAFGEPSGVMDTGGDSYWVNLVNNNINVTAFV
EAANRFVKVDFMLKYMVPKSMLSARDEHVARSKAATAKRIEKEAVLKGRSDMISYFLKDSDYKGTSIDEIACHFSQVILG
GGGTTATVLTGTVNHLIRQPDLTRRLLQEIVPMFKTSDEILAPALSECTFLTACIKEGLRLMLPTPGGMPRMSHGETVDG
HYIPAGTIVYSHGYTLSRSEENFKDANGFHPDRWIDPDNTDNKAASQPFALGPRQCIGQALAWDQMRVILAKMFYLFEME
LVNPPENWEDGAETYFTWKTNDLPVAVKRRASAKNDPIWTRVQNKN*

>CYP6648B2|10796|Pyrco1

MDSDGDLRNKCVRVDLTKKHAKQIAPLVEENKYAIPKHIGECDEWTAYPLQPTLLKVIALLSGRIMIGSPRNRNEEWCNT
VIQYSMDAFGGGAKLRLLPTFLRPIGAALLPDIRRVKNHQDVARRTVIPIVLARRSGEEKPQDDFLQWMMDSANENEQSP
EFLAKQFLIISLASIHTTTMSLSKAIFDLLSHPEYIEPMRQEIERVYKEEGGVFTNAMSKKLELMDSFFKESQRRSPPQP
IGTPRIAVHPDGFTFSTGHHVPQGTEIIGCPWLAASFDPAILEKPDEFDGYRFIKMRAQSAEADGRYTYAATSLGHWGYG
KHVCPGRFFADTEMKIVLCDILMKYDIRFAGSRKERPNDLVFELNIAADNDCQVEFRRRRL*

>CYP5093F1|11596|Pyrco1

MTAMSQQLSENLAQSSTAEQAVAPVASSLQIFSEDVRLQSSTIPCFTIRPSIMIGVLWAFIMMACYIKDRRPRKPMPPGP
PAKLFVGNQFDLGNDKPPWYKFKDWTDVYGDLITVWEGRRPTIVIGDPQVAHDLLNERATKYSSRPRFVVMGELYTNNDS
LVTMPHGKKFTKTRKLFNKNLNDKECLESYQPIQEAESAILLKHILEGSIPGNSKEEFVLETCIDRFTASIMTCISYGRR
IEKDDDELILEIRERMQYMATLNVPGKYWANTWPFLSSLPDFISPWKRRVKGKAEEATKMLTNLSQAVRARMELPDPPAS
FNQKLWKDKAEMELSEREIAYATGSLFGAGADTTAATLLSFVLAMICFPEAAKKAQEELDCKIGRDRMPTWEDMRKVKNG
EVKDGKAKSKPKDYLPYCQALIKEVLRWRPVAVMGGTPHASMTDDVYKGHFIPAGSTIVGNLWAIHHNEKYFSNPHEFIP
ERHLGTQDGVEKYPGGDGHSAFGWGGRKCPGQKLAENTIFIAITRILWAFNISKEIDSDTGMEKTYDINKYTIGINVRPE
RFKCVIKPRFEEVATIIAKQCTDVEGFLRKYEQSTESSTEHRTNGV*

>CYP6637B1|11910|Pyrco1

MGNHLLIQIAPITLSLERFAQLLQENRILGYPALCVSLYVFYWICLSFYRLTFHPLAKYPGPWLAAVSPLFYCWVFARGR
AGPEIKAAHDKYGPVVRIAPNDLSFATPTSYRDIYTKQNGRGTFIKTEFYKQISMGFETVGFASEDRVDVHAKQRKLFAP
VFSAQGVRAYEHLLLSTMEKFLKQIERIGKTDEGVDIAEWFHRLLYDVTADLAFGEPSGATDTSGDNYWLKLVNDNINIA
TYVDCACRFAVFKFLIENFTPKRALEARDRHVKWSIATTGQRILDPRREGRPDMLTYLLENKNAEGVTLDEMTSHLSQII
LAGGGTTAIVLGAMIYFLILNPEMLQRVRDETIHLFNNSDEIKAPALAKCEFLTAVIKEGLRMMPPAPTGLPRYSPGETV

DGHYVPKGTQVFVHPWTLTRSEKYWKNAWTFNPERWMDPNSTDVKEAAMPFLLGPRQCIGQNLAWDQMRVIITKIFYLFD
LELVRDIKDWPSECGTFLTWSTTPLNVRVKPREGALSDPFFARPPAKDE*
>CYP6855C1|12043|Pyrco1
MVAVDPSLIWLSIAVVTLTTMYIRRKLNQPYSNAPVLSVSFIEKLKLVGGMTEFVKRGYEQFGGATFQYTTFRGRRQVLV
CNDELINELKMLPAENASFASWSQVITQVDAILPLYAGGAKLIYWPEFAVKISHRWFKSTLWKDLYTTFPQLYEDVVQGW
ATQIDSEEPLFSLARKAIMPTVARYLVGAPLCNDPEFLEKVDELTHHIGLGSQISLMMPKILKRPMVRCFTKFFSVKAYM
DKTLHKLAAERSGKTEGLYDYFSVALAAVEKSNKPDWNVDRIITDFSTNAFAAHHTTSTTMSNVLLELAVRPEYQSRIRE
EVRRVTAEKGWTLEAIDEMVHLDSFMRESRRFRPLADMMVNRLVNKDTTLSDGTKLSRGMNVSALYSAREMDDTFYKSPK
EFDGFRFVGSNDRFTDADGPRYLAFGAGKHACPGRFFAAAIVKTSVAHLLDQYELLPGSEKLEFEISFEEQRLPSMKDRV
LFKRL*
>CYP51092B1|12627|Pyrco1
MLKSFILSSSHNLHSISTPINYAINHPLCSSFIILSVYIIWYLISCYFYENTFNSILRCDKLMEEGYEKYGKFNLPFRIP
NLFSGSMVILPPKMITELRSAREDVLSMHSWFDDENSNSHVFFTKHLPLQGYHLKTVRQAAKLPDTIPNLEREIEQSFDR
ALKALGKYEQLNMGEWAEIDPLELSHCIVQRLTWAVLGGKELANNEELMKLVQDYAISVFTGVAMLRVWKRWIRCVIGPF
VTASNRVRVWKIKRILGPAVEHRMEAFQKYFYNGKDNKDILDSLISESYRLGPAHHNIHEIIMRVLMLSWAALHTTSGAL
ANALFNLAAVDQNTGQDYRTILENELQAVFSEGSKLPVMKPRWTKITLSRVVGMASFLKETLRIHMVGAGQHCRRVVKEG
GHTFSNRLYVPAGVCICVSGIIIHSDPENYPNPKVFDGLRFGGPYERSREEMNDSDVKLTMPTKNFVVFGGGHHICPGRN
LADIELRMALAHFIENYDLELVSERPERSFMWVFQTPPLGLRIKIRRKS*
>CYP6497B1|13204|Pyrco1
MSLIISILLTGILAIAIYNLSLFLRCRAQAKASGFPYFCFPIADTKHWYMIANLDPVIWIIDNLFPLRLRDYINSSSYTR
RWHVSYRLHEKLGDVLLQVSAAPLSVYIADADAAKEVFSGWGNYVKPGWNMDHLRLFGDNVVACEDVDAAYHRKHVQPPF
NERNSALVWQQSLKQAEDMLSLWVTSPVVEKSRVDFRRLALHILSSAAFGVNLSFLPKVGIEKNSIFIDGEPKEGYTRSW
RHTLEYMAGNLVEIIAAKSMLPEWAMPDMMSVLRDMRCYIDTLIDGADMKAGVNGNLLSAIASTGKGDMKYEHIMGNVFI
LSVAGQETMSNTMQYAFIMLALNLEAQEWFARRLEEQLKDLPENWTYEEAYNRLSAPRCLMYETLRLFPPLPSIVKWTAT
PQTLLGHLIPGNSNISVNLGGLHHNPKYWGDNAAAWDPSRWDFQNKDSFLGWLEQPMEWAEAPGLWRPRQGSYYPFSGGG
RACLGRKFAMVEFVAVFAKIMKGKRVRLAEGESMDGAWKKIRRSKALTSLVANEDIIIVLEDR*
>CYP51042A1|2427|Pyrco1
MTNLVEFALSALSLLVILIVRRCRAPQAKAPPVFPYQIPWIGLASTFSYGAFKHLLQAHRIFGSLQPVTFVVAGKRIHVF
THPRDITAVYRNKLDLSFEKTVETFFLGCDYPSDPESISRLRTLLDSQHTLFATHMSPTKTVDIVRSMLPIMQSQVTKLA
QHTEEVDLFEWSYSALTTAVLTAMWGPRLLAESPELIPTLRKLDDDMLKLSAGFPKWMRRNETAHLEAVIGMFRNYYRVP
QTEGPSVTKDRAIECFEIGCTVDEVAAQNTFLVFGFTTNMPRHAAWATYWVSQDRHLLERIRNEIEPAFKREEVDTDHLM
TKCPVFKAAWNESIRVSGSAQSSRSVLRDTKIGEYEVKEGSIVLCVSRSAQTLEDVWGPNPEEFNADRFLNNPALERSPY
FRPFGGGTSLCAGRHMAARIGLTFTATLLRQFEIDADRTEVSIESPLLAGTFRPKDTMRAKIRERDIAA*
>CYP51074A1|2671|Pyrco1
MEHPLLTLSALLIFLPLLLNTFRLFHNYRLARSTGLPIVVYPFSPSNGLIIILLLSLPPIQRLVNRFPQQCADYFNNATFT
NHWKVKGRFAMMQGGGGGTMATDGKRQGAFISVSSAKIVVWVTNAEWANEVLNSRNHNMFPKPAKAYEIMKLHGPNILTS
EGSEFVHHKKHALPAFGERNNTLVWTESLRQATEMCGTWTSPLDIGSSTLSLALHVLSSAAYGIPMSFSERSEGFHSVLN
FVTGNLPMHALATSTFPSWFLKRFLKQHWNAWEDFGGYLRSLIHKARTRERGESERRDLLELLVRGGNNEKEGLTTEELT
GNLFIFTVAGHETSAQTLHFALVSLALEQEAQEWAAEGVKEALQGEDEDSGNWKYEEVYPKLARVLCVMLETMRLYPIVP
YIPKSTGPLGATLGGIDIPSSVVIVDPTALHRMPEYWGPEADSFDPSRWDVNNEKSYLAANKGQQGLQAYGLEYPTIHKP
RRGAYCPFSDGARGCIGRKFAQVTFVAAVATVLKGFKIRVAREDGEDQEAANKRARRALDNSYATVSLRGGGECEVVF*
>CYP6648A2|3223|Pyrco1

MALIEYLSTNNTAISAVLVCIIITALNRLFTYINGLDNLKNIPSVGFEGGYKKARERFLFDNLSLMKEGYKKDGAFKVVA
DNGKFQAMVSPNMAMEMRNTPNDVLSFTATTRDLLMTEYTGIQESDTAVKCVRLDLTKNHGRLIPAMAIETKWSFDKHFG
ECKEWTKFQIHPMLLQVVAVVSGVAFVGNPENRNPDWLDCAINYTVDVFSGTQKLRQKLLPKFLLPITARLSPEISRTKQ
HRIKARRLIGPLVEARLAGTVPPGDDMMQWLIDSSPAEERNVDRFAQMLLQLSMASIHTTSMTVTKALYDLASRPEYTKP
LREEVETVLKEEGGFTQNGIRRLDLIDSFLKESQRLGPIGNMTMRRKVVGSKGFTFSNGVHIPHGATILGVTSAAASLDP
EIFENPEEFDGYRFLKLRQEDPNKHVFSSTSTYHWGLGKHACPGRFFASTEIKMLLAEIVVRYDIRIKDNKRPADICWGI
SNSPDVSAFVEFRARQDIL*
>CYP51052A1|3348|Pyrco1
MRSKRLGYKEACMNLNRFCCMPLSNMDYRQHVLVGLAALVFHYAQHDYELDFVVLHLLGIFFSLCTLHTTVLTVSLGLSI
NAALHTTASLASTFAGTLTVSISIYRVFFHRLHHFPGPFMARLTKGYQLYRTVLDSRKYALVHEYHQKYGPVVRTGPREL
SILSLPAIQAIYGASTTTTKSVFYNQQYNDGHGTLMFTRDKENFLRRRKAWDKAMNGAALRMYEPRMEVILNILLDTLEK
ANGDPVDWTLLARYYAFDMIGEVGVGKSYDQLITGKTNSAVPALRMTGWWVGVVGSMPWLFRVVACIPIAKIGGVVSEFL
TWCGEQMDTTLKNGPKPDGHTTILDFLLYDEITGFGKLPYTATEDDCRALIAGGSDTAAGQISSMFYYLSQHPDVMKKLQ
KDLDEAFPNHKYDSKISVPYLDAIINESLRLLPALARGLPRTTCKEGMTIDGLYIPGYTIVSVPTYSIHRDPRYWERPND
FWPERWIKGVGAEAMKCFMPFTMATNCVSYKVENGCCTFYSDMGCEVKLFTAHNRSHLYRNWRTSCTQTSFLQMPLQWLR
GLVDGRRMADVTCT*
>CYP512CS1|4420|Pyrco1
MSVEQIEINGLVAALLKLEPWQLAALGGLGFIAYCVYYHQSRKIDLPSVGIEPGIFGPWKAAINFVHNSDALIKEGYYKY
GKKGQAFKVATPARYMVLFSDPKMIKELWQTDESIMSFAKAGQERIASDYTLSRDLGAHPYHIEIILKNLTNRLSSILPE
VFDELIKSFEDNTSITSAWTPVHNFSVMLKCISRTTNRLFVGLPLCRNEDYLNHCIEFATAASRGGATIDMFPFFMQPLV
AKFFANRDKALAKVIAHVGPLFVERQKKMKELGEAWTDRPNDAVQWILEAAPPGTNLKDMCLRILFLNFAAIHTTSFSVL
QVLYDLAAHPEYQEPLRQEIEAVVTQFGGWTKQALTAMKKLDSVLRESQRTSGVTILTGMRKAMKNHTFSDGTRVPKGTW
VLAPTLAIHGDKDIYEDGLKWEGFRFSKMREQPGQEAKLQMVSGSTEYLAFGTGKHACPGRFFAANELKVLVAYIVLNYE
FKFEDGKRPENKYYAYSCVPDLKAEMMYRERGDRHQSFANNCLPRD*
>CYP51052B1|5415|Pyrco1
MEFREHILSSLAAVIFLRATLYTELDYQVIRFVNIFLSLCFFNILFLVLHTTSFLPALLHTLSLAATFLITLTLGIFLHR
VLFHRINHFPGPFLAKVSKIYTLIICWRRPRRFEYHAAWHKQHGDIVRIGPRDLSINSINAVALIYGSSSPCQKASFYSQ
SIPEGYGSIFSIRNKKLAAHRRRAWDRTMSGVNLATYTPRLERFSSLMLQRIGEQVERGQEVDLTKMLRWFTFDAIGEVG
MGKSYGMLDTNKQHEAVEALLNSQWWVGVVGVTPWVLRILPKVPMGKDPGIYKFMKWTHDQVELKTKEGVEKDDEKTIFG
LLLEDDVTGYGKIPQSCLLDDARSIVAAGSDTTAATMSGTFYFLVHHPEILAKLRQTLDEVFPNRKYDPKVHVPYLDACI
NETMRLQPPVPCGLIRTTPMEGLTIDGVYIPGDINVSVPTQLIHRDPRIWKKPNDYWPERWIEGVGEGSKVFMPFTIGSY
ACTGKPLAWLEMRMAVAKTLLRFDVEKLGSLDDWEARIDDYFGAQVPELKLKFTERNA*
>CYP51048B1|7360|Pyrco1
MDHESTPREIWPCCPHCAQRLVVLHPTSYRDINKSKVPFPKTEFFDAIDNGFNEAGIGTERDVEKHLRKRKFLTPAFTPA
ATKMYEPLVAPHLDSFLKEIEERGKLPEGVDFVEWYQFLTFDTAGDLAFGESFKALSMGVQHRWMSLVTESIDIAAYLEA
TRRFPVFLAIFQKFIPAKITEARKWHVDWSKQQTYRRIANPSSRGDMLGFMVDSEGKTSISHDELTAHASQLILGGAETM
TGMLVGTTYFLAKTPRVQQFLQKEIREAFESVDDITAAKLSTMKYLTAVIHEGLRSFPPGPTGLPRYSPGALVDGHFVPK
GTKVSTHPWTVTHSEEYWDKPDEFRPERWLEEGNKDVKDASVPFGLGTRQCLGQNIAWVEMRLYLAKMMWLYNLELVDKE
RDWVKDCKTYFMWMKTPLMIKVERREGF*
>CYP6501A2|9329|Pyrco1
MNPIALLSRDLVTSIIKRLPFGLDEWKYLHFFYRDWEFRTKFQQFDEYGEVFMEVSSGGRACYIGSAEVAQQVFQSRNGF
LKDIDFYRVVRVYGDNVLTTAGHQWRHQRKITSRSFTEDVYATAWTEGLSQSSAIISQWMQGKILSGPEESTPGGFVAPA

TKAMALNLISKAGFGVSLPMVASKSQASETKNKASIGIMDDAYFSADWTPAGHTLSYAEALDMFLENIVLARVIPFGILR
RGNNFMRKLAAASDDVGLYMKELVARERSKPSTTLLNALAHDKGLPEQEVIGNLFIFALAGLDTTASTLQFALCLLALNQ
DVQEWLHRDIKKALEGESEDPAEWNYSQVYPKLVGCLCVIHETLRLHPIILTFPKTTGPSPQPIVYKGKTFIIPEDTNVF
VSMPALSYHPSYWGSMPSKFAPQKWDARDPDSGWGNGSELMPGTQLRQPVKGAWAAFSEGTRSCLGKKFALVEMCAFLVM
IFGKYRITIAPKEGETQEMAHERVKRVMSESTALISVTMREDVGVKIEKRVS*

>CYP548BE8|10404|Pyrco1

MSVLSGMPIPGVAEVATLSLSAIRNGFVLLVTYLVTIVVYRLWFHPLAKYPGPLLARITDWNTAWRAWKGDRYLELYYAH
QKYGPVVRVAPNMLSFNTATALKTIYGHTANAKNIQKGQFYTAFPAVKGVHNTHNCISKMEHGFKRRVLSVAFSDNALKS
MEGAVIDAVNTLVDGVRKDGKNGVDMGERFSWLTFDVMAELCFGKSFGMLTDETQRFVTDLISKATHNHYICGNYLPIRF
LNLGRFLFPTIARDRWRFIEHSRSCANERMALDQAAKKDFFYYLLNAKDSETGKGFSTKELWGEANVLMIAGSDTTATAL
SSTLYYLSRNPSALDKLRHEIRSTFNSPDEIVTGKDLADCHYLKACVDEGMRMAPPVSGLLPREALASFEVDGHVVPEGT
VVGVPIYTIHHNPDYYPEPYQYRPERWLDDDQEQIEKAQSAFNPFSIGARGCIGKSVAYMELRLSIAKLVWEFEIRHKAT
EGKAELWDEGFAVMEGEYRLLDHFTCKKEGPVIEFTRREM*

>CYP5945D1|11801|Pyrco1

MDLPTILTPLLLGISTQFALHPIPEVPTLPVLSSWILFDIVFLIYNLLHLMIPTAVLSLIQFNALYFISLLLTCAFHRLY
LNPLRAFPGHRLSALTKLQETHLNLQGRQCFVLESLHAQHGDFIRIGPNELSIRNVDALKLLLRQPYNNRGPFYLIGKAG
GSDHLSVTRSNAKHLKWRRIWDKAFGRVALEEYNPRVEWHARKLIRKLEEIAGGEVDIRGPVEGFAFDIMADLAFGVPNY
GIQDGTGDPSIISFAHDFVRIVAAVAPLRNICQIASLLPIPAGVQRFRLKQHEMINARLSLGTSRSDIFAHLIGEDTESG
ERFTRDQLTSNAELVIVAGTDTTATVLTQLFRELAIRREVQRKLYEEIVEKMGEEVELDVNNVKTMPYLQAVIDEIMRLW
TPLPSGLQHQTGPDGAWVDGVFVPPNTAFRIPHMALMKDERYFPRGREFWPERWIEEGGVKEPKAFVPFSYGAHKCVGMQ
LALNELRLATASVVRKFDIQLGDSYNEEDYSQGWKDYFLVALPEVKLKFVPRK*

>CYP52AL1|1182|Pyrco1

MDPATIIASVKAMTPIQLGAVGLIGFYILASLFTSIQTSIKSRSLGCQSPPWAWDPLGLRRVYNMMRYALNHQLPLYSVK
LFKDFNTKTVPIIDFGKPGYLTCDPRNIQAALATNFKDWGFGSARYPMLPILGDGIFTQDGEAWAHSRSMIRPSFTKSQI
ADFESLEEHMQEFFTTLEMTTKSDGAVSLKPLFSDLTMDFASEFLFGETANSLKQRREGIAETGMAHWFDAGMHHVTMSF
NMGRLHNFWRPKEYRQSIKFVREFVDGFVHRALEERLAIGDIKEKERAKMNGGRYVFLNALTDNITDPIVLRNQVLNIML
AGRDTTAALLSWVLWNLARRPEVLERLKQEVAETIGVAENATLPTWQVLKDMRYLQAVIHETLRLFPSVPFSNRIATRDT
VLPYGGGSDGNAPLFAPKDTQLIYSIYAMQRRPEIWGADAAEFSPDRWLRADAGKMLREVGWGNMPFSGGPRICPGQQFA
LTETSYVVSRLLQRYQWVEKAPGESDVPSFEASLVTPPADMVEVMFTKY*

>CYP51062B1|8795|Pyrco1

MDDTSQHQNLILPAIATLIAAYILSKLAKWVIAESQKVCSFNGRPCEAPPMRPESWYTFGLAGQLRTIKSAQAQRLPARM
AELCEELGDTWAYTLFGTRTIITRDHRNVQAMLATQFDDFTLSTDRDAMHQLLGTNAIFTQSGHAWKSSRALLKPTFDKA
SIADLDRLELFFLQFRDRISSSLSEAGTIELQSLLQKLTMDSSADFLLGSPVGALQEANNPSVNAANFTESFDIAQQVIV
TRWLLNHLFWLYNPPHFRAACSVVHEQVQKFVQKALIKPKNEKRYIFTEALAEATKDAKVIQDQVLSVMLAGRDTTASLL
AWTVLSLSRHPEVLEKLRAAVATAVGVGESAKIPTQEELRGITYLKWVLHEVLRLYPPVYANTRCAAKATTLPYGGGKDG
QSPIALKKGERVVASFFGLMRRKDLYGEDAEEFRPERWGEEQLRKIGWGWVPFNGGPRICLGQQMALTHASYFLVRILQT
WEKLEEDEKFKQKEITYDAKITMYSGLGVNVKLA*

>CYP6001C30|9322|Pyrco1

MSCPVKASSSTAAASAPSQASSRAPITNGASHHNNYTAAPIKVNPPVAKPTRKEVEATFEKFASLIHASNRPLPHRYGDG
RTEKEDDQTTGIRNDIAVLRKGGFLMESLQTIWMVVQNKRRGGPVDDKTMIMERLIQLTSRLPESSRLRTKLTSTQVETL
WNSLQHPPISYCGDQFVYRQADGGYNNIQDPNLGRAGSPYARSVKPMIKMPGAPPDAYTVFDSIFSRGPSGENYRPHNNN
VNSMLFYIASIIIHDLFRTNRADPNISDTSSYLDLSPLYGINQTQQDTVRTFKDGKLKPDVFAEKRLLAFPPGVSVLLLC

FGRFHNHVATQLKEINEGGRFDLKHDRRWHNDDLETKNAKALKKQDEDLFQTARLVTCGLYINFVLNDYLRTIVNLNRVD
TTWTLDPRFDPSKMYNPDGTPAGVGNMVSIEFNLVYRWHSCISKRDDLWTQEFYQKLFGPDVDVQKVTLPEFLRAVHIWE
MSIPEDPAERTLEDFVRQPDGSFNDDDLVKVLMESVEDPAGAFGARNVPHVMRLVEVLGIEQTRRWKVASLNEFREFFGL
QKHKTFESINPDPVVANTLRQLYDHPDFVELYPGIVAEDDKEPMVPGVGIGPTYTVSRAILSDAVTLVRADRFYTIDYTA
AALTNWGIEEASSNKDVLHGTVAYKLFLKAFPNHFKYNSIYALHPLTIPSENRKIFTALNIVDQFDFDRPKRIKQRIPIR
SYGATKAILTDPTNFKVTWGAGFDYIMEAKFMLSGDGSPFTGMKKFVGERLYGQGGNINWKQQIKDFYKETTTKLIRKKA
YQLAGTDCYQVDAVRDIGNIAQTIFAADIFNLPLKSDDHPKGIYTEQELYMVLCAMFIAIFFDMDSSKSFPLRHAAYAAT
RQLGAVVEQQVKALKSWGWLQGVWDPLNIRGRNKSALRDYGYHMINRLIESGDSAAEITWKYIIPTAGASAPNQGQIFAQ
VLDFYLQEENAEHLAEIQRLAAEDTDDAWEVIKKYALEGGRLAGTFGLYRRVDCDEIVLKDGAEEMTLVKDDLVFVSFIS
ASRDENIFPDPLQIKLDRPEASYMQYGDGPHECLGKHANIVGLTTMLMEFGKLKGLRRAPGLPGQMKTIPKPGGFKIYMK
EDWSGFWPFPTSMKVRFDDII*

>CYP539A41|10086|Pyrco1

MQYPGTNLQQVFNELSITMITDLLFNKIPTLLAASLLGGCLMIFIYKLTWKQQISRLGKRSALIPASFMGLSEVYCVLKY
AKMNKNREFWAMRFTELQNYTMELEMLWKRIIFTSEPENIKAVLATQFNDFGKGEVFHDSWHEFLGDSIFSTDGELWKGS
RALIRPQFIKDRVSDLHIFENHVQHMISLIPRNGETVDIAELFFRFTLDSATDFLLGESVNSLGTTGEMKFVKAFAQIQQ
YQNDVTRLGPLRVFMPKGNFKENIKILNSFVEPFVKKTLQLKPEELLEKTETNYNFLHALAGFTRDPKVLRDQLVAVLLA
GRDTTAGTLSWCFYELAKRPECVEILRQEILDTVGPDAAPTYAHLKGMKYLQHVMDETLRLYPAVPFNIRVALKDTVLPT
GGGATKLEPVGVPAGTPIAYSALVMQRRKDIFGPDADEFRPERWRNWSPKPWTFIPFNGGPRICIGQQFAYAEMAYTIVR
LFQTFDGVQDRMVVPQFERCETTISPGAGVKIALRPVKN*

>CYP6001E3|10931|Pyrco1

MSIIKDVHDLRTAVHTTIANRQDKEERVFGKILDELRAAGGEFAKHAVLAVEILNEKLSGQLTDDRHLLIEKIIRLISKL
PSTSFARRKITEILVDKLWDSLHHPPLTYVGEQYQYRQADGSHNNIMYPDLGKAGTPYARTVRSETKSPGAKPDPGLLFD
LLMSRGDDFSPNEAGISSMLFYHASIITHDIFHTNRKDANISDTSSYLDLAPLYGSNQEQQNQVRSFINGELKPDTFHEK
RLLGFPPGVNVMLVMYSRFHNHAARQLAAINEGGRFTAPRHMPMEEGMKWRDEQIFQTARLITNGLYVNISLHDYLRAIA
NVPATDSDWTLDPRVKIKKIFNAEGVPQGVGNQVSCEFNLLYRFHSAISDRDAKWTKEFYKKIFPGQDPLQISMPVLFQG
IKAFEESIPEDPSVRTFADIKRNADGTFRDEDLVQILKESIEDPAGRFGANHVPDILKPVEILGIIQARKWQCASLNEFR
KFFKLKPYEKFEDINDDPYVAKTLERVYGNVDNVEMYPGMFLESTKPKMDAGMGLCAPYTVSRAVFSDAIVLVRGDRFLT
LDYTPANLTNWGITEAASDYKTLGGAKMHHLILNAFPNHIKFNSVYAMQPFYTPTKSKEIFEKLGVADKYSFDAPSANSR
LIPITSLAGLKQVLSNKQNFRVPWGAKMSSLESYMLASDRPECAAQREVVKDALYGPNGSLQNFATYSEEITRKLLKREA
YELGRKGVHQVDIVKDISNLAALHFAAELCYLPLETAPNGSGKSYTEEQLYKVLCDITTYVFSDADPTRSWARRRDAQEG
TAKLCAEMERIVQSLPPAAISASANPTTPVTGYAPKTMLGKHLAGRLTAANNAAQSVGAAEVGGGCPVGNGPLGSYGINM
ARKLVASGRSAREVAEILVGTASAFVANTATAFAQLIDFYLEKANEPHWNEIKKLSAENSAAADEKLTKYVLEGFRLSNT
LGIARIAVPDSDSVSVKDHGVTVTAKKGDRLFISFVATSRDPLAFPSPNEIKLDRPIDNYVTFGEGPHQCLGKDLNIVHS
RAMLKVLARLPGLRRTPGEEGHLKFVPKPGGLKVYLTPDWSEYTPYPTTMKLMWDGAPPS*

>CYP6002C37|12884|Pyrco1

MSLAKSLADGGLIDDRQYVVEKIIQLAASLPDHSENQVKLTGQLIRSLWNVLQHPPMQYLSNIGSVAAPEGKSSDFRYRS
ADGSYNNPFIPTLGMAGTPYAKSVTGKTLLPVDLPDPGMLFDLLFAREKIVEHPTKTSSMLFYLATIIIHDIFRTSSLDS
NKSSTSSYLDLAPLYGNNLAEVNAMRTMKNGMLKMDTFNEPRLLGFPPGVSAFLIAFNRFHNYAAGQLAIINEGGRFTKP
TLKPQATKEESEAYDKAMSNYDEHLFQTARLVTTGLYINIILNDYVKNILNFNRTNSSWTLDPRGNFGQLYDQSAMIPAG
VGNAVSVEFNLIYRWHACVSARDEKWTEDFFKNEMCVTDASAISIEQLQEKLKAWGHSIPRDPGQRIIHGWKRSDNGKIA
DKDLVEELIRSTEDVAGSFGPQNIPKVLRSVEILGIIQARKWNVATLNEFRQFCQLKPHDSFEDINPDPEIAQKLRTMYD
HPDRVELYPGILAEDAKQALAPGSGLCPGFTVSKAILSDAVALARGDRFYTTDATPANLTNWGWNEVASNPEIAQGRVLY

KLLMTAYPGWYKFNSVYSMYPFTVPEETKVIMEGLGTAATYNFDRPTFAPSPTPILSYSAAKEVIMDNKRFLVPWGPKIY
GMTGQDYMLSGDKQWNYDQKDHFWKCMYSPENGIKEITKFYEEVTRKMVKQKAYAVPGGFRLDAVRDIGNPMQSLFFSAL
FGIPIKTQETPLGVFTEQEIYEMTAAQFAWTFLDLDPVHSFKLRQAAHKATATLSPVIMDVCKAVKHGAPLIPRHPRDEN
GIVSIYGKHLISKLLDGRSLEEISAAVMPTAAGGVATQSHQFALLLEFYLSLKNASHWTKIRSLAASNTPESFEQIRRYV
LEGMRLSPAAYGVVRHYAGAEPTTIGSTQVQPGDNVFVDFITANLDPTAFPNPMEIDLTRDEDRYIGQGAGIHKCLGLPI
TSVAMASMLKTFAGLEGLRLERQEGMRGKVVNGLRSFLGEDGESWEVYPNSMKVRFDGFLE*

>CYP52AV9|13046|Pyrco1

MAVMSTLNQISSSVTPTETIIFLLGALALHHLVTYLFTQYNNAKFAREHGCLPPPRWKAGILGIPHFLEMVRAAKKKEHV
QFITSRWTPGWYTFIQNQFGYDSIQTADPENIKTILATSFKDFELGFTRKDAFHDMLGEGIFTLDGKGWEYSRGLLRPQF
SREQVADTEMLKIHVERVLEHMKNAEGTEVDLQPWFYCLTLDSATEFLFGESAESLIKGEEDQKGFAYAFNEGQHWIMWK
LRWRKLAKLWNPSEMVRINKNVHGFVDRYVNMALNREKYPLPIEMQKKYVFLDQVAQHQKDPKALRDQMLNILLAGRDTT
AGLIGWTFYLLSRHQHIYKKLRDELEEAFGTGEPGVWRLPTFEGLKDVVYLRYVLNEVLRLYPSVPLNGRDCVRNTVLPV
GGGPDGLSPVFVHKGMRVQYSVYAMHRRKDIYGEDALEFRPERWGDGSKIGRGWEYLPFNGGPRICLGQQYALTEAGFTI
ARIMQNYEMMEAVNPFEDPKIEATLTMGPQQCCVRLIPVRK*

Sphaerosporella brunnea Sb_GMNB300 v2.0

>CYP6643A1|61890|Sphbr2

MMLILFLFIVLVPAVFLSHRRRRKSPSDIPLLHPLSAFTALPILIPTLLGRRNTTIHRAHVRHGPIVRLSPTEISISSPA
ALSAVYTSFPKHPWYNIFRNYGAAPMFAILPGREHAARKKLLAAAYSNSAVLKSSVLRRTANETLPVLLKELANSQGREL
EVWMVFVRLTMDFITASLFTRDVGSRFLDGGDQHILELYHSRRAFFALSSELPWLARWIVPKWVDDANDKLEAWCRDLCD
RRRRQWRQGDEECSLDRLISAGLPEVDAASEMLDHIGAGHETTALALAFILVAMSERPELQRELRESLEPLMEVRDGWKL
PKLDGEGLEEHGLLNAVIKESLRLYTPIPGSQPRVAPKDLEILGHIVPAGTAVSAQAWSLHRDPAVWKDHETFFPERWLG
EEKKAEMERAWWAFGSGGRGCIGRYLATWEMRVVVASVYANFETECNGAAAVLDAYTTAPLGEKVGVVFRKV*

>CYP5109B1|138193|Sphbr2

MSRPAIHLSVIDADATHTALSIVGLAVAAFGLLVVYRRFFHPLAHFPGPFWASVSSLYGVWAFVSGGEHLIHRGLHEKYG
PVVRFGPNELIVNDPYMLPLIYHRKADKTEVYAPNFGIETVFTWLRHQDHVAAKRRIAHAYSLLSIKNLEEYIDECLAQW
ISALSDISKDPSKPINFARWAEWFTYDVISYLSFGEPIGFIAARADVRDLIKNYSDASMLLEILALLPKVSWWMRKTWVG
RKFLMAKAGDRKGVGVIMAERDRIFEKHTRDMEKGDGSKGILLTKFIAAKNADGTPMSTDDVKAEALLAMIAGSSTTSNA
LVRLIFNILQHPSCLSRLTAELDAITPTLTSPIPTFEEARTLPFLSACIRETFRYSPSTVQFPRVSDGLELNGKWVPAGT
SVSASPWIMHRNKEMYGEDADTFRPDRWLEANAETTALWDKYDFRWGYGARKCLGRNIALMEIYKATILFFKHFRPELVE
EIGFTSSGGPKSTTLYIHPRN*

>CYP51074C1|165127|Sphbr2

MTMAAAYTPLVDSAHAHPYLTLVALLLAVHAAHSFISLLHNIRLARATGLPYLIYPYSFSNPLLVLLVSWPWVQNLIATI
LPQLGQDYIFQTMYSTHWRVQGRWNQRLGAVFLAVSPTRITAFVADAGAIREIMADRERFPKSLEQYQTLNMYGPNLVAS
EGEQWAHHKKFITSPFGERNYKLVWRVALQQARELLGTWNTDMPDVQNDLFRQALHTFSEAIYGIPMSFSTELREGEDSE
SLGFQKALTVITTNLLPHAISISVLPHWLRPKRHADAHTNFERFLKGMVQNADKGGSSLLSLLVDGEGLTDQEIMGNLFV
FTVAGHETTAQSMYFAMVTMALMPEVQDWVREEVDRVLGAQESQRIEDWRYEDVFPKLGRVLFLMLETLRIYALIPYIPK
SCTYPTTLTVGGKAHPLPPMDISLSTASLHLSPLYWGPTASSFDPTRWDASNTSSFLAKNRGAQGLQVAGLEFPNIHRPE
RGAFVAFSDGARGCIGRKFGQVLFVATITMVLRGWEVGIKCGEGETREMARERVRGVISRSSATVALGVREEVGLVLTRR
GA*

>CYP61A1|235223|Sphbr2

MDTTYNPPSAVSSAEQPLQTILNGVGAVSKSLEGVSMWQVLATILVLSITYDQVKYIWNKGSIVGPAFKIPFMGPFMESV

DPKFSEYLAKWNSGPLSCVSVFHKFVVIASTRDLARKVFNSPMYVNPCVVDVAKKILRPTNWVFLDGKAHVDYRKGLNGL
FARKTVAQYLPAQEEIYDLYFKRWIELSKDGKPRQYMGEFRDINCHVSLRTFCGTYISDEAVADISENYYKITAALELVN
FPIILPYTKTWYGKKCADFVLEEFSRCAKLSKEAMEAGKKPICTMDFWVKSMIESREYGKLTQEEKESFSGPKPMVIRWF
SDEEIAMTIFTFLFASQDASSSASTWQFQILADRPDILAKVREEQLRVRGGDPYKRIDIDMVDQMVYTRAVVKEQLRYRP
PVLMVPYEVKKSFNVTPEYRVPKGAMIIPTTYPALHDPEVYVDPETFNPDRWLEGGEAEAATKNWLVFGTGPHVCLGQHY
AIMNFMSMIGKASLFLDWEHSPTELSEEIKVFATIFPKDDCYLTFKQRLPLEKASS*

>CYP51075B1|299224|Sphbr2

MRRTSGLMDALKTLNSHELWLAFQALDKTALSYDLLRATAALSIGYVIIKCIYNRYFHPLAKFPGPFLASITEFYHIYLY
LTVAAEHIVDEELHRKYGPVVRKAPNFLIINDASMIPVVYSKNAWKPDMFYVPEGFGRTNVPVAFFQKDPKEHTISRRRI
TPPFSPKSMTAMKPIIQTRVSEWTAKNKKYADSGKAIDWTEWTQALAYDVLSELVFGEPFGFIATESDVHGLLREFYQAI
PYAGTLVRLPWLVKWITSLPFAGAMLPDPSDKRGMGKIMGIRDAHINHRLQNPTNKPDILNHLLSYKNEDGSPIPLPVIK
FEALVMMNAGSETTASTLCCFVLNILRFPHIYKKLMAGDLSTENISSPDPQFRYLSAVIKETLRWTHPAPSLFPRLISGN
GVVLPDGRHIPAGAVVNINIQLAMRDKKVFGEDAEEFVPERWLVESGWGSPEAIKHMEQYNFVWGYGPRMCVGKPLAEAE
LLMATKELLVNFVPELVNPEKPYSRMTNYTTIVHEGFYIRLRNRTEA*

>CYP51075A1|442356|Sphbr2

MSKAAPFDMATFLRFDTVDKKTFFYQVSTIFAAASIVYIVGKCIYNRYFHPLRHFPGPVAASLTEFYLPWSFYFGVAEHV
TDEQRHARYGSITRKAPNVLQIDDPELLPVFNNRTTHKPKRVYYSPAWGVGDVPTILNQLHPKEHQVARKRLTPAFIGTA
VKKMQPLMQKRVDEWIEKSWEYANEGTAIDWSKWTQSLAYDVITELVFGAPIGFVKTKTDVYGVLQQFKQATPYIGILNR
LPWLVEAIWKSPLGSYLIPAADTPLGKIFSVRDRLLQERLDKSVDKPDILSVLLKGVNEDGSPVSLDVIKSESFILMAAG
SETTFSVMCSFTINIIQHPRVYKKIMETDLSPENLAQEDPQFPYLYLAIKESMRLMHPAPSFFPRLVGKGGVTLKDGRFI
PEGSEVAMNPYCVLRSKKIFGDDADQFRPERWIDSPPEQLALMETYNPIWSYGRTSCPGKPVAEMELLMAFRALLLNFVP
EFANPEKPWDHITNYGSLVYEGLYIKLKPRVA*

>CYP5078A16|545427|Sphbr2

MHDIAETIMTNFSVKLLVDLLKAYPLQLFFTIAVLHLLRNRFLTGLNRIPGPAIAAWSALWRLYDVSKGDAHNTAIALHR
KYGPLVRIGPKHVSVGDPAEIQNIYGLKKGFTKTAFYPIQCISWNKTPQMNLFSTRDPQYHRDQKKLVANAYSMSALIEM
ESAVDDCTKLFMSKMAPYAASGNPVDLGEWLQYYAFDIVGLFSFNKYLGFLDKGGDVDAMMEGIAGILNYAATIGQIPFA
HNFLLGNPLNPLFFPNMETWNQVLQFTLKAINQRCSIVVNGELEVRKDQVVGKDMLSKWASAKLGDPLKMGTREVIVHLS
TNVFAGSDTTAIALRAIVYMLLKNPEKLEKLLKQLDEADAAGLLSDPVKYKETQSHLPYFTAVMKEAMRVHPSVGLLLER
HVPTGGATICGEYIPAGTIVGINAWVLHNDPKVFPNPEKFEPERWLDSPEEKLQEMERSFFAFGAGSRTCIGKNISLMEM
SKIIPQLLREYKLTLANPDKSWKTRNMWFVQQSGVDIILERRK*

>CYP53A59|629714|Sphbr2

MLATYILIALLPVAYWLIPYLIDSKKLRRFPSPFPAQFSEFWLFYQARRGKRYLAVHEAHGKYGKFVRIQPEQVSIADSA
AIPVVYGHNTGFLKPEYYDAFVSIQRGLFNTRDRAEHTRKRKTVSHVFSTKNVLQFEPFIHHNLEMLASQWNKLSDHPDL
PGGFRKIDCLHWFNYLAFDIISDLTFGAPFGMLESGKDQAIVKDPVTGKVTTAPAIQVLNRRGEVSGTLGCAPWVKPYAK
WLPDKFFTQGVKAVQDLAGIAIARVSERLDQGKGVGREDLLKKLMDGRDANGNPLSRQETEAEALTMLIAGSDTTSNTLC
SLMFWVLRTPGVLQKLQEELDNALPGEWSIPNYAAVKDLPYLRAVINETLRIHSTSSLGLPRAVPAGGATVCGEFFTGGT
VLSVPAYTIHHSKEIWGADASEFRPERWFELTAVQKRSFIPFSVGPRACVGQNVAELEMTNIVATVFSGWEWKFAEGEKQ
GLPGMQLETCEGFLRKPLGLNVGVKKRNRA*

>CYP52AV10|770624|Sphbr2

MELQQFATLSPKILALFLATAYVLLQLITAAITTYQDRKFARANGCRAPRRWYSGPYGLIEFRRFMQAANKGQHVEYVAS
RWEKFGAHTFVMRSFSTEIINTIDPRNIQTILATKFKDFSLGPARRNGFHALFGEGIFTLDGKGWEYSRTLLRPQFSRDQ
VADVDMLDTHVTRLLNLMKEAGSEPVDLQPWFFSLTLDSATEFLFGESADSLLMGDQAGFAYAFNRGLEWVMWKLRFRKL

AQLYEPKEMREVNAMVHGFVDRYVDMALNRDKHPLPKEAEDKYIFLDAVVADTRDRKALRDQMLNILLAGRDTTAGLIGW
TIYCLAWHPHVYRKLRAELEKAFGTATPGVWKRPTFESLKDVVYLRHVLNEVLRLYPSVPLNSRDAIRDTILPAGGGPDG
HSPIFIKKGTRVQYSVYQLHRRKDIYGPDANEFRPERWAEAKVGRGWDYLPFNGGPRICLGQQYALTEAGFTVTRILQHF
ESITPGDPSVTYPHMLSTLTMSPLRCFVKMVPVRRDN*
>CYP617X1|776030|Sphbr2
MGSLSRILLTSAIATLLLSRILAAGSTGLLLTFAATTALLALAKFVYAVLLWPYLFSPLRHLPGPKSRSWFMGELSKILA
LPSGEPQKEYLRIPNDGLLRYLWMWNEERIFPTNARVVQQALSTDADIWTKPPAVRSGLALVLGTRSVLFTEGEEHRLQR
RILAPAFSRRQIRNLVPVFWEKAVLMGGKVLADAAAQENGAVNISRWASLATLDIIGAAGLGYEFRALEKGENGSELAAA
YATLFSPRPAANLLQLANLFLPSWFVYRLPIRRVREVRAAHRTIRKVARELAAAAKTRELASKKPAEDDSDDERDILSVL
VKSGEFNTPDGDVVIRDQLMTFLAAGHETTATLLVWAMHLLTLHPQWQHTLRAEVRAAFPAGCPDTVTYEQLEGLKHLHH
FTLETARYFPPVPLTMRMNNRATTLAGVFVPKGTVLVVVPWALHRDAKIWGADADEFRPERWAAEHPPMESNYALLTFLA
GPRNCIGKGFAEAEFKALLAALVGRFSFEGTGQVIEIQGGITSRPKGGLSVRVEEVPGWA*
>CYP51056A1|783005|Sphbr2
MLDLHHFLARSAGAAVVCGLLLSIFHATHGFQAAAKSVGMTAVVFDSGLLSSVLTHRLFFHRLRRFPGPFFAKASKLWTF
LRVANKPQAHVLTMQLHQRYGDWVRIDPRELSTTVAAAIPAIYSSGSALIPGDDNHGSIFSVRNKEIHSDRRKAWSRPFN
GAAVASCTGWMVRRSMREILQDVEKSGEAYSLSALWFTHLSFQPLFRIVLRLITMISICSGTSEPRSTLHGAALRLLISL
PGAAKALEPFQDWCNNELNQEIKKREAGEKPNGSIVSRLVNDPEGLFTLTEIARNLDSRLTVAAGSFEYSDTVASAIVGV
WCYMAANPTIYKKLQRILDQDFTGGDREYSNSVGSHTPYLGGVIHEFLRLQPLIPSALPRETPPEGITIEGVYVPGSTII
SVPVHAIGRDGRYYERPFEFVAERWTEEMPEPVRDSRVFMPFTLGPYQCAGNPRACLEMRMVLSRVALNFDLALANHGTE
DVPRSPSGPLFVAGAGFVDQVFAEEEEWGNKSAA*
>CYP671K1|891864|Sphbr2
MNTEPVVLSVSAAAALAICFHISIIPHEIDFKIKRLLGLYVASIAGLYYLLHRETVTNPGTVTVLSATVFNAALTASILI
HRMFLHRAGKFPGPFMARVSKFYSVFLSVKKMQYHKEVEQLHRKYGDFVRTGPREVSVIRASAVQTIYGAQSTCSRAPFY
SQVADHPNCSLHASRDRVYHNLRRRAWDRGFSTAMLKDYEPRMGALTDLLIAQLRSRVGTPVDITTWSNYYSFDVMGDVG
FGKSWGMLESGKLHKAINELHAAMALLGYIGQVPWLSRLLMELPAATKALKGFQDWCWKQMEEKKKAVAQFRPIEAFCWL
AVETPSFGPWGARAVDSWVSVLPVRNLSAATRLFGRVEVLDKPRDLITWLLTDSKLDTKLPSQAINEDSRLIIIAGSDTT
ASTLAHAFYFLAKYPGAYKKLQNVLDAAFPGGDAEFSNATACGLKFLEGVIFETLRLKPAVPGGMLRMTPPEGLTIDEVY
IPGDVVVSVPTHSIQRDERYYEKASEFIPERWMDEKAHMVKDKRAFIPFSKGTFGCVGKNLALMELRMVLAKVALNFELE
FAEGENGSRIEEETKDTFTLTVTPLFVIFKEREK*
>CYP6761C1|898162|Sphbr2
MEKIATSILHSIPSGLSLLSSFLLYRVLLVVYRLYFHPLSRIPGPKLAAATTLYVAYYDVYKNGTFLRDALPALHKKYGP
VVRISPTEVQIHQPELYHRVNAMHSPFIKDPVYYGSIGVSSSAFGTTDQSLHRSRRSLINPMFSKRRILEASTIMQDVVM
KLCTILETFCDSNKPVPLSNAFYCVTVDTITAYTFGESWNMLDEPNFTSEWVESVLSFAGMVQAKIQFPRALGVLEALGK
VFPSLTPLAFIKLIQYCENRVQQALEDDAAAKAAGIPPKERPDTIYDALLYPPSEKAQRPSYEELVGESISMVTAGTDTS
ATVLQFLAWHFLTRPEVQEKLLQELATVEAEPNGLLPLKRLEELPYLTGFIKETLRYNTPAPGRQNRIVPEGGLTIPSTG
AFLPAGTRIAFCVGMLHHDPRIFEDPAEFRPERWMGQKGKELDKWMLAFSKGDRVCVGINLAYAELHLVVANLFSRFKLE
LFETTKEDLECVDHFVGHLKGRIKVIASRREREAMMV*
>CYP51058A1|898479|Sphbr2
MWSIASTLLLAVLSYWVLTVLYKLFLSPLSRIPGPWYTALTSLWLKPYTLSGRRHHIVHSLHQRYGPIVRLTPTQISIID
PTATCEIYSSTGNYQKSRFYEIMGAMSRVQMFVMTDPIEARWRRKLLSGGFSLGSLKSFDGVVARKVDAAIGKMVQVARK
EGVVDMYLWWYLMATDLIMMFGFGGEYQMLEKGEVTSYITDMKTLTMLMGLRGEFPRLVKVLSKIPFSPLRLVPELIRRV
ESYGSQALQAFDGEELEFENGFKKATWFSQIIAEERRLKGKMDPRFRLEREDIEMEAASMIFGGELLYNPQIGLGAASLM

LVVIGTDTVATTITYLIYTILKHPDIRDKLLDALEPLRKHDIATLVDSQLVKIPYLQMLVDEGLRLHPGIQGALPRSVPA
GGRTLAGYYIPEGTSVESQNWTVHRDPKVFPEPERFWPERWENPTDEMKRMHMPFGGGHRSCLGMPLALMELRRATAIFF
LKLGKNVELAPSTTDDSMEGLTFFTTTPKAGKCEIVIKGMEVTA*

>CYP5093H8|900468|Sphbr2

MYGSLVTVWTGRRPTIVIGDPKVACDLLDRRSAIYSSRPRFVVMGELFTNNDSLLTMPHGDKWRKTRKIFHSGLLRRACE
TYKPIQEAESQRLARDLLERPADFGRHLERYAASVMVCVAYGRRVDDLDDSIVRRIYERMRYMSTLNVPGAFWAESFPLL
KRVPDCLARWKREVKQRAEASSTLLMELAQQVHDRMQAGETPASFTKNLWEKYEADPTELTPREIAYATGSLFGAGSDTS
SATLMSFILAMTCFPHVARKAQAEIDSVVGRGRAPTWSDEPSLPYCAAVIKETLRWRPVAVMGGTPHASIKDDYYEGHFI
PKGTTILGNLWAIHHNSKYFKDSHEFIPERYLEVQEGTEPYPHRDGHSAFGWGRRICPGKLLAENSLFISITRILWGFNI
EKHVDPATGVEDVYNTFAYTDGFNSKPQPFRCCITPRSAQIRDTIEHEAKLGEKFLERYS*

>CYP5945E1|901353|Sphbr2

MGGLNAITTRDASAHRPLRGIWEQGFKSNSIREYSTRVELHVDRFIRILEQTKGKFVDCVPLVTNMTFDIMADLSFGTDY
GMQAGTGDPSYMGYLHDYLCALSVTGSLRTLCDFMTVLPYDKATAEYRKQGEKLLDGRIAMGKSRPDIFTHLLGEDIETG
KKLTYSELNANAQLLIVAGSDTTSTVLSNVFRELSLHPDVQEKLYQEIHAAAEAGPFDCQNTKNLPYLQAVIDETLRLWP
PVPAGAQAQTGPAGATIAGRFVPPLTAVRVHHLALMSDDRYFPQGERFWPERWTESREDGVKDVRAFIPFSFGTHVCVGK
HLAYNELRLTVARVSQKFKIELGPNYDDQQYRKACKDYFTIMIGSVEARFVPRI*

>CYP52AL2|907218|Sphbr2

MMWPLVYQVLAVCIGIYALRILHQKIQNQRFARSHGCKPAGTNLDILGLYRFYELLASSLNQTIPLYGEALFSRQKRNTI
QVVDLGRSNLVTCEPKNIQYILSNINEYGLGGARDNFHYLLGNGIFTADGEAWAQSRALVRPSFTWTQIADFESLEEHIH
ELFLAIEALEKDGVTEMKPLLHDLTMDFASEFLFGQTSHALKQRMEGATDGLAHAFDRGMVHLTLSWILGELHWLWRPSE
YRKVRDAVHSYVDGFILKALEARSSKSSMLEAETEKERVLREGGRYVFLNALTTINQDPEMLRAQVLNIMLAGRDTTAAL
ISWIMWVLAREPKIWQKLRQEVKSNLGAGKNARLPTWKVLKDMKYLQAVINETLRFYAVVPWSNRIALRNTILPVGGGPD
QSSPIFVAKGAMVSYSLFSMHRRKDIWGEDAQVFSPDRWLRGDAGKKLREIGWGYLPFSGGPRICPGQQFALTEASYIVV
RFCQRYKSIEKALGESDEPTWLSSAVAPPGDNIRVRLEKESNL*

>CYP6836A1|909178|Sphbr2

MEWMPLRSAAIEYLSEHWKAAILSVILLRVVGTIIHRRWLHPLAKFPGPFWGSITNLYSVQVNWSGKQYLIHHELHKKYG
PIVRYRPNLLIVSDPTALPEIYHRYADKTPFYTQIIGDKTTFTSLPSTEHTISRKRISPAYAPAYVRSLEPKMDQWIVDW
TELNRRVFLKSGPVKDFNNRLMYMAYDLLSDLTFGRPFGFMAKNGDVNEIIKGASVSRLMHHLLALVPSFLWLLRNTFLG
YYILPRRYPDRGFGYLMAEAKRLTLSEEVNPPGGEKTPLRIMHDTKSTDGQTMGMERLASEARILLIGGAEPAAAITIEF
LLNVLAHPGAYQKILDEIAAAEAAGRLSSPVATYEECQNLTYFSTCFKETLRGAETTRGMMPRYAPKGGLTIAGQFVPEG
THLSATPWVTHRTKSMYGEDAEMFRPERWLEASPETLLQWNKYDFQFGYGARVCPGKMMGLMVLYKQTLEILRHFEIRKL
KEDDPKAACFTRKLDDEVIRDTGLYIHALRK*

>CYP50335A2|913544|Sphbr2

MRPFILPIPAVVLVVIPALPILSLLLAFILRRRRRKPTITTIRCLHVGRALLTSSPGHLTPLASRALPNQRLVTAFGIDN
SFTTASYSRHKAFRSRVEHLLRMSSASWQQTSTLAQTLFRQNTSSLHLVPFVRQYVLTTILHIFFADFVPFQPDAVAELG
DLIHSLWLQSKVGAPSVTSQRRLQETLRHLFPGMTENPLDLLLPAYETLWRTVMRCFLEVQFRAGEQAPAWRELLAEYLG
QPSSPRFYASLDGVSPAFLVKETLRLYPPTKRIYRATTEKNWVAVDVEGLQCSPEVWGKDAEHFRPERWGEADAGTWIPF
GTGTFVCPAKGEFAPRLIGILVAAMLGAVPKDARWTVEHEADDVLRGGRLNNERDGYESLKLFW*

>CYP50335A2|913550|Sphbr2

MRPFILPIPAVVLVVIPALPILSLLLAFILRRRRRKPTITTIRCLHVGRALLTSSPGHLTPLASRALPNQRLVTAFGIDN
SFTTASYSRHKAFRSRVEHLLRMSSASWQQTSTLAQTLFRQNTSSLHLVPFVRQYVLTTILHIFFADFVPFQPDAVAELG
DLIHSLWLQSKVGAPSVTSQRRLQETLRHLFPGMTENPLDLLLPAYETLWRTVMRCFLEVQFRAGEQAPAWRELLAEYLG

QPSSPRFYASLDGVSPAFLVKETLRLYPPTKRIYRATTEKNWVAVDVEGLQCSPEVWGKDAEHFRPERWGEADAGTWIPF

GTGTFVCPAKGEFAPRLIGILVAAMLGAVPKDARWTVEHEADDVLRGGRLNNERDGYESLKLFW*

>CYP51062B2|913595|Sphbr2

MILYAAISLLLGAFVFRWISHNAKRICTVNGRRCEDPPMRPEPWYTFGLRGQLATVKASKAQRLPLRMVQMCDALGETWA

YSLFGTRTIITRDQRNIQAVLATQFDDFGLATDRDAMHALLGTEAIFTQNGPAWSKSRALLRPSFDRAAVADLDRLEIFF

TRMKQRIEETIPADRCMDMQTMYQKLTMDASSDFLLGNAVGALEEEGKDDGSGLAVQTFTGAFDVAQSVIAIRWALSNLY

WVYNPRFFRDACSIVHTQVQRYVKRAIQLRTAGTPQDGKKKRYVFMEVLSESSQDPRVLQDQVLSVMLAGRDTTASLLSW

TTLCLARHPEAQKKLRQAIRETIGDRIPTQAELRSITYLRWVLHEVLRLYPPLHANTRCATKYTTLPYGGGADGSSPIAL

APGEKVIYSLFALHRRKDLYGDDADSFRPERWGEERVRKIGWGWLPFNGGPRICLGQQMALTHASYFVARMLQTFAVLEE

DPKVVGQPVTFDAKITMYSGNGVGVRLVL*

>CYP6793A1|921476|Sphbr2

MLLLTLVTSLPSWMLLFGSFLIYWIIKIVYRIWFHPLAHVPGPKIAAATHLYHFYHNAFNKGQFVPYILPGLHEKYGRVV

RFSPDEVDVHDVDVIQSFYAHKFAFKKDAKFWDAFGIPRSSGLLYDYSEWRPRRNLLNPMFSKQSINTATNSVIYPMIEK

FINRLQEFADSNKVLPMDGALYCLTVDIITEYTCGKAWGFLSEPGMDSAKLQSVRWLTAGSPYSIEFPWLKKVVLRLHKT

FPKLNLGGYSVTRRAAVETVEHALALNAGNSQKVNSKPKYILDALLNPDHSKNFPTLGFDDLLDEAMMLFAGGTDTTAHM

VLFSIWRLCIDPDTRKKVTADVRTLVRGSDGRFIVAEVEQLPYLSAFIKEMLRCYYGAIGRTPRLVPAGGITVPSTGTFL

PAGTRLSTAFISYHTDPDLFVEPHLFRPERWIGAPGKALEKWLLSFGKGERICLGINLAYREMYLLLAELVTRFEIEPYQ

TTAKDMEMIDQLAVAPRGRFKVLLKSKEL*

>CYP51048C1|929139|Sphbr2

MSLFEQSIRVSAHQAWTFVASHPKLSVVGVCILLQIARWIYLLFFHPLARFPGPKIAAISELWYAYVYASGQSVWVLDEA

HKKYGPVVRTSPNSLSFATSASIRDIYRQKNFLKASSFDDIDAVFEVPGLGTDKDPESHAKQRKLSNPAFTTSAVREYEP

LINEYLDPFLKAIEERGKDVQGVNMEEWFKYLIFDTAGALSTGESFDALKGGVPHRWMKMVTGLLDMAAYHTVLSRITGI

KFLLSLITPHLMVEERKFHTDWIKDHVNRRIASPSDRKDFLHHFIEKDGSTTCSVEQLYAHTSHFLPGTTETTAIVILAT

FYHLLKTPAAWHQLQEEIRRAFTSVEDINSTALSALPYLNAVLREGLRIMPGFSNGFPRYSPGAVVSGHFVPKGTVVSTH

HWTVVHDETNFEAPYEFRPERWIDPNNGDTKEASQPFGLGSRNCIGQNLAWVLMRLYLAKTIFLYDLELVDPKQDWMRES

KSYFLWIKAPLMVRVSRRNGV*

>CYP548BE10|929130|Sphbr2

MGVFADMPLYLPSIVHLMTALRNAAILAAVYLFTLAFYRLYFHPLAKYPGPFLAKITDWNTAYHALRGDRFLELYKAHEK

YGPIVRVAPNMLSFNSATALKSIYGHSSIARCIQKGQFYTAFPAVPGVHNTHNAISKMEHGFKRRVLSVAFSEAALKGME

GMILNAVDTMLEGIRRDGAKGVDMADRFSWLTFDIMGELCFGKTFGMLTEQSQRFVSDLIGKATHNHYICGNYLPIRHLG

LGRFLFPTIARDRWRFIEHSRACANERMEMAHAAKKDFFYYLLNAKDPETGRGFSTKELWGEANVLMIAGSDTTATALSS

TIFHLCRHPQMLAKLKAEIRGAFASPDDIVTGKELADCHYLKACVDEAMRLAPPVPGLLPREVIAPCKIDGHDIPEGTVV

GVPIYTIHHNPEYFPEPFEFRPERWLEGNEEQLEKARSAFNPFSIGSRGCIGKSVAYVELRLSIAKLVWGFEMKQLPVRG

KADLWEEGFAVKDGEYRLMDHFTVKKEGPVVEFLPRSD*

>CYP6529A2|929240|Sphbr2

MVISTVSSLPSQLSLASAVLGVTSHWAYFIHGDRNTQSFVFFIVLIVGPAALAAALHGVAGVALMQALAVAAVSTGSYLA

GLFASIVVYRLWLHPAAHFPGPFPARVTQLFYPYAYMRCNGQYYRLQKAWFDTYGDVVRTGPNEVIVADPSVLPHLAKAA

KGAWYSIGQSNPSVHSVRDVRTHSIRRRVWDRAFTPAAIAGCVATVQRYRERLLQALASGERGETNITACVSHFAFDTMG

RLTYGRDFGMLEKQGRDGSDYFLRMTHASMRVVGLLGHVPWLLVLLERLGAAGKDHMRFLKWCDEMTEERRRRGMVGGVR

DLFQVLLEAEDGNKHPHHVPLRGDSRTAIVAGSDTTASTLITLFTYLADSPEALKELQQDLDSIPEDSTDSRLLNACIDE

ALRLNPPVPSGVSRTSPPEGIALDNGTFIPGGIDVLFPLWASSHDPRWFDSPDEFRPHRWLDSSPEELAKMKAVFHPFWL

GRYGCAGKPLAMVQLRDVAAAVVRRFEFSLVGSVEEAFDRCVDTFTVEMGEVRCVFKGRERA*

>CYP567E9|941206|Sphbr2

MKDITTAVPFISNMATSLSVSQVAAAVALSSLVYFVITTLRNHFTHPLAKFPGPVSASFSNFTYCRRFLGGRQPYKVLEL
HEKYGPVVRTAPNELSFSSSQSWRDIYGQRKGHQPFIKSPFYDGGNFAAEAHSIVSVRDPDEHNKMRKYLSNAFSDRSLK
EQEDLITEVVDLFIEKIGQKGKEGINMVTYFNLTTFDIIGSLAFGETFGGVKSGIEHFWVAIVISSLTQGALADCFGRFP
LVASIFKTLFPGVLKKITENTKKHEDYSMNLVKKRINRKTDRKDFFARILENREQFNLSDVQLAAHASDFVIAGSETTAT
ALACITYYLLRQPDILKKLQQEVRTAFRSYDQINAQSTASLKYLQAVALEGMRVYPPLPFALPRVVPPGGDTVDGHFIPE
GTIVSTAPLAASMSSANFKDPWKFDPGRWIGQNDVDDLEASQPFSLGVRGCLGRSLGWMELRTILAKMHFKYDLELINKD
IDWHRDSEMHTLWKKPELMVKVLPRRS*

>CYP567D15|941205|Sphbr2

MASTIDVNKLLPLFQAATPAERARYAGILAVSLFSVYFLSTSIYNLFFHPLAKFPGPFLARSSLLWRIWKSMRGRFHRAI
EREHRRYGPVVRISPNELSFASVASWKDIYGHAVGGKSTMIKSEFYEMYGSGFDSLCIGSERDPKNHNRMRKSLSAAFST
KALSEQEFIIQKCVDGLIAQLGRKGTQPKGVNMTKWYEMFAFDVLGEMAFGESFHCVESGEPHFWSELVTKHLFFITVLD
NLSRYPLLVSLGKVFLPWLTVSVRDKHTGYTRAKVAQRLAAKTSRKDFLTNLIGKVESGEVNTEELTAHSSTLVIAGGET
IATFFAAVTYYLLTTPEAYGKLRMEIRARYKSYDEIDSTTAQQLPYLQAVISEGLRMYPPGAQGFPRVCPGAMIDGQYVP
AGAEVYTSAWTVTHDPQYFHEPMSFKPERWLDPNCADVKDASQPFSLGPRGCLGRNFAYVEMSLLLAKLHYMYDMELLNK
KLDWEGNSHMHVMWWKPALNVHFTRRVGA*

>CYP667F1|941224|Sphbr2

MLSLSLLVLLAGVGIYNVFSFLHCLRQARRTGLPYVFSPVHELSNWAFLTNATLRWLYTDSLLTGQGWPRWARYMIKDGM
YEDKGRAHRELGDVFLVVTPGGLLCYTADAGMSMNVLARRRDFIKPREKMKMIEPFGPNIVSTELPHWRRHFKITAPAVG
DKVNDLVWKETTAQTTALQGAWVRNGVENLNGDVNLLALNVMGAAGFGRQMQWRASDETTLPAGHTMTLLAALNDLVRHL
GVILLLPKKAIQWSPWKKGYVAHKEFEQYMQELIAEEKQGLAQNALYEGKLKGNLLTAVLRTNATEEKNEKATATGEKGT
LSGTLSDTEVLGNIFMFFMAGYDTTANAMSFGSVLLALYPKIQDRIIAELDQVYAEAAAAGRSELTQAEDFPKFRYVLAY
MYEALRVFPVVIPIARGTTQSQQVIADSSATFVLPAACEVVINNTAVHFHARYWPEPETFEPLRWLSEDPNAYVPSAPTA
QQRREIGKKLQERGQWNPNHRRGTFLTFGEGPRACIGRRFAHAEYAAYFACLLRRHRVHLADGLAAGEVERRLRMTSGGG
LVTLTPRDNVKLKLVAR*

>CYP654C8|941218|Sphbr2

MASDTTVSSLPSAVVHAQLWLRAKVTEPVQLPWISTSLHPLVLLTLVCSVLYLLTPPRTKKQRLVPNIPVVGVGVGGIGA
ARKRFLSESRDMLLEGYRKYGGNGGMFYVPSPLGERLMIPPKYVEELKSAPVDKVDFVATFIEMFEGKYTTMGSRSTLHP
RTVKHQLNQNLINVMPAVEAEIRDAFAAQIPPCEDWTALNMADKFVQIVARVSSRMFGGTALSQHDEWVQSTINFATDGF
IGAQKLKKTPVWLRPIASRFMPEIQKISHHYDVARKVLIPLLQERDKTGERPLDLLQWMKENAQGEETDYNYIAEIQLKV
SFAAIHTSAAAPTQLIYDLCAMPEYIEPLRDEIIQALREENGKLTKQAFLKMPRLDSFMKESQRFNPLLLITFERLVTDD
LTLSDGFTIPANTQIGVPTHAVTMDSTLYPDPEKFDGFRFHRLKSHDPIAASRQVYAASNLASMSFGYGRHACPGRFFAS
NEIKAIMVYLLLNYDFKFPEGQQRPPSLVVETQYLPNHAATVLMRKRTPEF*

>CYP6855D1|944239|Sphbr2

MTALLLLLLGLATTAISSYLLYTPSRKRTSNAPHLPAGLRQKLQHLGGMQAYLYAGYRRHRQGAFQYRTLFGRQEVVVCS
SALIRELGEAANESMSFSAWTQKTLQLDYLFPGWNGGGGGGVAVAVNIPSFSMRVTHRWIRRTLVKELAASFAGLWRDVV
AGFGPGRLELAGEIPPVYALARKCLIPAIARYSVPAPLCEDAQFLATLDELVYQIGTAGVVVGLFPEALKPWVVRYALSI
QKSKQVVWEKLGRLAEEITPSSDATDHFSFGLGLIQRSNQDNWTVDQLISNTCIQLFAAFHTTATTLTMVLLELAVRPEY
QHILREEAAGISPTLDAMDSLWKLDSFIRETRRFRPHVEIILRRLVIKETTLSDGTVLTSGLNVAAAYGPRERDPRYYSA
PDEFDGLRFYKLRMAALEGGASGGGGGFMATSTDGTEYLGFGAGKHECPGRFFAVATIKAVVVHLLSQYELLPGSEPMQM
TMRFEEQQLPSNKDRVIFKPLR*

>CYP675Q3|946202|Sphbr2

MFVATLLSNPLFYPIAIVLSVAGWFYYQRVLSPYAGVPGPFWASLTRFWYLNRINAQDMHRYTKMLHKQYGPLVRIAPNE
VSVSDPAAMKQIYAVNAGYTKTDFYPTQAPNLSPHGDSFTQLDEKKHTYRRRMISHIFSMSSVMEGEKFIDNATAAYFQV
IEEHAQKGEKMEMGHWLHMYAFDIIGELFFGRMFGFIKERRDIGGYMGAVDVILPHAIRLAVLYEWMRPIINVVMYPLSA
NLRQAIRCFEALGAESKRYVDERIGKPSARPDMLSKLIKVMEEKSPDFDITDVYTEAYTAIFAGSETTATAMRSAIYHLC
RNPQYKAKLVAEIDAAQREGKLSEMIAYGEAIKLPYLAAVIKEGMRVQPSIVLTFPRHVPAGGATICGHFFPEGCRVGVN
PYVLHYQTSIFGEDAEDFNPDRWFRPDAANMDSYMFQFGSGSRTCIGKNVAMAEIYKFLPKLFREYDVNLADPTKEWKEE
GAWFVKQTGVEVKFTRRNVE*

>CYP504A41|955448|Sphbr2

MALPIVFAVAALVVYLVLKVSNRTISPKIPGLPAVPGLPFVGSLFDIGSCHSRKAQEWAQTYGPVFQVRLGNRWIVFANT
YDAIQELWIKQQSALISRPTLYTFHKVVSSSQGFTIGTSPWDESCKRRRKAAATALNRPAVESYMPIVDLESSASIRELL
VDSQYGEKEINPAPYFHRFALNTSLTLNYGYRIKGNVDDAQLREIVDVERAVSNLRSTSNNWQDYVPALRLLSGRNQEAV
EMRKRRDKYMIDLLNELQRRIKEGTDRPCITGNILKDPEAKLNEAEIRSICLTMVSAGLDTVPGNLVMGLGYLSTPQGQE
IQKKCYDAIMQVYPNGEAWDACLREEKVPYVMALVKEVLRFWCVVPISLPRVNIKDVVYKGVTIPAGTTFFMVSLGN*

>CYP539A38|960204|Sphbr2

MLLDVVMLSRPAVAAALAVVFVLVIAVRRLLEIRKIRRLGKRASIIHSRFFGIDETLKVFEYARKHQNRELWEMRFKEFN
NHTMEVRVLGKRIIMTAEPENVKAVLATQFLDFGKGETFHQQWQAFLGDSIFTTDGKEWSASRHLIRPQFIKDRVSDLHI
FERHTQRMLSLIPRDGSKIDISELFFRVTLDAATDFLLGASVDSLKYGNVEFAVAFQRIQAHMNNVSRAGPLRIFLDTKQ
FKQDLKVLNAFVEPFVEKVSRMRPEELKGKDESDYNFLHALAQFTRDPKMLRDQLVAILLAARDTTAGTMAWALYELAKR
PDTVQRLRKEILGTVGPTAAPTYEDLKGMRFLQHVINETLRLYPAVPFNVRVALKDTSLPHGGGPYGLDPVGVPAGTLVA
YSTLTMQRRVDLFGPDVAEFKPERWDHWAPKAWQYVPFNGGPRICLGQQFALTEMGYILCRLFQNFDAVEDRSTEPQIER
CEVTISPGSGVWVAFRPAKNG*

>CYP6855A1|964987|Sphbr2

MAVVVWLTAGVLVLTAYLLHWQTRVKPSNAPTLHSSFVQKVKHLGGMTALLHSAYKTHNQGTFQYVTIRGRRQVVVSSDK
LITELKNAPHEILSFARWTQDLIEFDTILPLYTGGQKLVQWPPLVQKLTHRFFKGPMTKDLATNFPDLFDDLVYGFSKAP
QDAPVYTLVRCAVLPAVARQIIGAPLCRDEEFLKNTDEMTHLFGVAPQLAGMFPGKFLKRLAVRYLTRWETVKRYIEEAC
EAQAKDLNRPKGKDFFSLSLAAIEMNGAKGTGEMWDIPRLATEVCSNFFAAHHTTSATIAMALLELAVRPEYQPILREEV
RQAVDEKGWSMDAIDSLEKLDSFLVEMRRWRPLADMMLNRAVIRETTLSDGTILKPGTYISASYSARQLDPRYYTSPEEF
DGMRFYKLRKEGDQGRLFTDVDGTLHLGFGGGKHPCPGRFFATAVVKLGIAYLLNQYQVLPGSEKLEMEMHFEEQRLPSM
KDKVVFKKL*

>CYP6136B2|967823|Sphbr2

MLKRLVRYVDSPMRKQGIPGPFLAGYTSWYRAFYADITHNWHDKLVQLHDEYGPIVWIAPDEVSVSDPKLRGVLYSFADE
RREESFFPKAKSFETGLFNNDFNFVFETDPARARIGKYALSHPYSEKGLAKLEHHFDEAVQAFTEGFTEHVVSTNTAARL
NAWAHFFMFDLATLLMSGYSRGLCLDGKDSDGAIRALRDIFNVVGALVPVSFALKISTKAIRKAILNSRLEHLFSWSIGY
QNGESHKNARINEVSDKLPNNLMAKFRAGEKKIRKLFPDGNWTEAITNNVFFIYAGSMVASNALPMVIRYIYSDKRVLDK
IREELAGYEGPIKMSEIAHVNGHCNLPYLEAAILEALRLNPTFGISLGRTVPSIGCKLNEFFIPRGYVVSMSPWATNIDK
DYFGEDAREFRPERWLGNHPTELAKDDSGAPRTMRNYIEAGWFSFGAGARVCIGRHFTEIAFMKFVGSMVRNFEVEVVNP
GYVWYGLIQHAEDVMVKATVREDTKPAVEEVVAPEAIVAN*

>CYP50127B1|969067|Sphbr2

MPLLLTTLAVALLTVFCCTIVPHLRRLHTILHFIKTKQLPLYPGTLYAEHNSFTIPFPIPGKAGLLTAEPECIKTMMSSE
FKNVGVSLGRAPYTPLLGAGLFIQDGARWSHPRSLLRPLFTRSAVSSFALLESLLQPLFAHVLAAQKSGEAIDLEPLFQT
LTLDLTTAFLLGEPAGEKGKEFADALEEGMKHVNFSLQLGRFWWVWRPRGFTAAIRQVEGFVEHYLRRNSSGAFVKTLLD
AGESEQQVRAHVLNTLFAGRDSTAATLSWVVWNLVRRRDIAARVRAEIADVVGDALPTVQALAEMKFFRAVLNETLRLHP

PVPLTNREPLGPTVILPGKAPIRLKQGEPVYLDFFTLHRRQDLWGDDAAAFRPERWGENEQFEREMTARWCFLPFGLGGR

SCLGQQLAINTVMYTIVRLSQKVGGLERVVKTQEDGDQVRFTNSPVPAPGEGVWVALKE*

>CYP613|969646|Sphbr2

MAWTNFLTLFGSSSFAPAIAIAGVVVCYHFRRILKSITCRALSRVIHLFLCWRYPIKGLPTAPYAWPNGHGDAAKFLNGA

RNSIEWGDLYGNVYRIWSGMNPEVVVTRSEHVREIFRDSDCHIKAENNDSGYLLSQLLGQCVGLISGDLWRAVRTRVEVP

FRHRAAVEYLPRILEQTENYFRDWERTAEGSADASALLLDPAADLKMYPFLLTARLVYGDLDETQVARLRELAPLREHLF

TYVIAGGVARYAWSKYLPTKANELLTKFLADWLKFNKEAYERAKVLQSGAPIVAMWEAAEAGVLEKTHLLQTLDEQLFAN

LDVTTGAIAWNLVHLATHDSEQQQLLKEIRENLRGEKRREDYFLRSDTFLAACINESSRLRPVAAFSIPQSAPSDKIVGG

YTIPKRTNIIVDSYALNIRNEYWGPDSTIYRPSRFLKLKPSDYRYQMWRFGFGPRQCMGKYLADMMLRACVAYLVENYQL

SPTKSEGDLEVEVNPDCWITHPQTKIRCVRR*

>CYP567W1|980732|Sphbr2

IAVAVVATGIRNVYFHPISKIPGPKLAAFNRIYFSYIWLSGRYPQIVKDLHDKYGPVVRLSPNQVSFNTATSWKDIYGHV

KGRKQFLKSDQYDDDGTRARSIVTSRDPAEHGKIRKLLSNAFSAKALTEQEDIVHNYVDLLIKQLHEYGVKKDETLEMNM

WYHACTFDIIGDLAFGDSFGSLETGKQHFWVSNIPDFVSAGAYLAMLNKWIGNGQLMQLIKHRVVPKSLYEKRKKHLSYS

EDKIISRMNATKTRKDFMSKILSEKEAQGVTVPTLISNSSTLVIAGSETTASFLSGTTYSLCRNPRVYKLLAGEIRSSFS

DYSHINGHSTESLPYLKAVIEEGLRTYPPTPFGMGRFSPGETVDGIYIAEGIEVFTSSWSATHSEDNFHRPYEFIPERWL

DKGCTDKKEASQPFLLGTRVCLGRNLAMLEMRVILAKMLWAYDMTLQDDALDWVRDSTAVMLWRKPDLPVKFTRREGVAV

PPLDG*

>CYP675P1|996403|Sphbr2

MLLAAALANPLFYPAALVVAAAGWVVYQRVFSPYAGIPGPFWASVTRFWYLRRVIAEDIHRCTKSLHQRYGPIVRIAPDE

VSISDPAAMKQIYAVTGGFTKTDFYTAQTRISVSPRNDLVTERDEAVHAVRRRLVNRMFTMSSIMESEAHINRVTEAFFQ

ALSEHATNSKTLDMAQWLRMYTMDIIGELFFGRMFGFIQERKDIGGYMAAIDAVAPHGMRVGVMSSWMRPFQMLLIPFSS

QFLPGIRGFKALAAESKRYVEERVGMKTERRDMLGALLQIAEEHNPEFDLRDVHMEAYVAIFAGAETTATALRSTVYHLS

RTPHARAKLQAEIDSAHRRGKLSPMITYGEAVELPYLDAVLKEAMRVQPPIAFTLPRHVPAGGRTICGRFSPAGSRVGIS

PYVLHYQKSVFGEDAEDFSPERWLGPDAARMDRYMFQFGYGSRSCLGKNLALAELYKLVPQIYRAFDVRLVDPTKEWKEQ

GSFFWKHSEIEVLLVKREVQ*

>CYP573A21|1004409|Sphbr2

MLAQLSIGRIATAIIAITVLNIIRSIIVTRYFHPLSKFPGPFWASVTRLWITYHNLTGKEHEVLYELHKKYGPVIRITPT

MLMCSDPKMLPVIYHRRADKADHYVTGSFGKTPSVFNIQPHEAHAAARKKIAQPYAFSAVKPMEPLVDRQIDAWISKLDT

DFAAPGKKFDFAAWATFFAYDVISEVAFGKALGFTRKGYDIDNLIRSFHEGLPAFGFLCRLHPFTKWIKTTWIAEKYMIP

KSGDPSGIGNIMEFRDDLIEERLKENKANSRMGRHDLLQRFLEARDVEDGIDMEDLKAETLLVLLAGSDTTATEFQAVMI

DILKAPGVYEKLMAEIDAAPLSRVPTYDEALAHCPYYVACVKEAMRLCPAAPNMFPRVVSQGGLQLYGKFAPEGTEITCN

PYTTHRNKELYGNDAEVFRPERWLEDPERATEWEKYDFGFGYGSRKCLGQSIALMELYKGPLQFFRTFKPTLVNPENPAK

YIVAGGVSRHVDLWLTIVKRA*

>CYP51066A1|1008140|Sphbr2

MALRLLFDVLLPALLCLPGLYLLHLLFTSLLDLRKNYQAAKTIGLEGGARPILTPVYEVNFLYKLTLPLHEPIIRFFGLA

DSLYWQLFFMDWCFKTRHDQFKQRGTDIITTVSPLRTVLTTANAEVAYQLGSRFEKPVHLYSRVSFMGENVVASEGAQWR

RLRKILQRPFNEAVMSTVWQATLDQSIPLRKKWCDAAEEGVNVERDIPRMALGVLCGAGFGVTIPFDSDGKQELPPGDEF

FASSTPTPGFHFNFGDVLAFLLSNIVTVLATKALLPAWLLRHGPVPRKWKNGAKALEETRNYIQKLVDRERRRNETAYYQ

QEGHHSNLLTILTNPESSGGLTEDEQIANTFIMVLAGHETSAGILRYSLVLLALFEDKQDWFLKELDQALAGIEPEDWTY

DKLFPRLSCCLCIMNEALRLFPGVPVLPKWCPTPTTLKYNQKILHIPAGTMINISLPALQQNPKYFGAKSYEFRPEVWDA

RSSTGWTEETSNQPIDEGMALPYRYIRKPIPGSYVPFSEGRRACLGKKFAQIEFCLVLAILFQKHRVKLVLKPGESKDAA

KARCWRVLRGSLSLLSLQMQEQVPVKWVRR*

>CYP51F1|1022663|Sphbr2

MGVLSVLLMPLGPWQGELSTPVLVAVGTFAFIVVSVVVNVLSQLLFKDKAKPPMVFHYFPFFGSTVVYGMDPYAFFEANQ

KKHGDVFTFILLGKKITVCLGPQGNDFVFNGKLAEVSAEEAYTHLTTPVFGEGVVYDCPNHRLMEQKKFMKFGLTIETFK

TYVPLIVDQVEDYIKKSKYFKGGKGTVPLTEIIPEITIFTASRSLQGKEVRDALDGSFAALYHDLDLGFNPMNFMFPWFP

FPGNKRRDAAQRKMARFYMDIIQRRRKDPNSAAKEQDMLWNLMDRSYKDGTPISDKEVAHMMIALLMAGQHTSMATTTWT

LLHLAEQPHIVADLYKEQQEVCGLEPRPLAYDDLAKMPLLNNVIREVLRMHPPIHSIMRKVKAPMTVKDKGYVIPPGYHV

LAAPGASAMDEKYFKNPKEFDPHRWDAVEDEDSGETFDFGFGLISKGTASPYLPFGAGRHRCIGEQFANVQLGSIIATFT

REFEFALPGDGKVPPPDYTSMITLPTPPASVMWKRRNP*

>CYP51071A1|1024912|Sphbr2

MRLESLDFLSPWLDLFPNQFPSPISVAVAIVFLISSWSFLSIARRGNAQTDLGSVPILGSRFYLRGCFDDSYVIVREGYQ

KFSKQGKVWAVRISTGELMYILPPCVLNEIKILPPSKASFMRVVNDSLKWSLHGDILSNIHIDVVRYSLNSNLAGMTSFL

VREREAAFESALGHITKDWKVIKVWEMVFKIMHSISIAVTFGEDFRKDSSYSNHVLSYLHTTPLLTFLYFALPGPLRTPF

WWLSPVGLKLRYHVRKVKSKLLPEIQRRASLIQDTAAGTTTNTQKERKPSDFVFIDALLRRRPLPLTPSEANRSADELMF

MGFEQGPIALIIIQLIYQVLSEPDCIEPLRAEVDEALVEGNGWTDDALNRMSKLDSFIRETLRLRLPAPFTTTRRVLQPL

QLSNGLKLRPGMLISSPSLGVNTDEEIYPDAERFDGYRFYDAATKTARPSAGTTTKDFLSFGAGNQVCPGRLLAVKVIKL

VVGGLLHDFDLAFDNGRKERPRDVYSMWQIMPDRSASIVIRRRERAPAP*

>CYP5078H1|1027441|Sphbr2

MAGYYPDRIVSEPLTWKEKDSGFLAGFIRTHALTVLATLFLLLLLLLLLRNKFRRGLFSLPGPTRLYDVWKGQAHLTHIR

LHQKHGALTDADVHRERKRLHGHTHNLSSLLKMETAIDACTSQLLETLRKCSLVFGKKLGFFEEARDVDGMMRHIQGVLV

YASHCGQIPEAHPLLLGNPLFPVLIPAMETWNALKTINEVREGLTLRRDGGLQIQEKLEGKKDMMSRLSRLKEEDPEQYN

TRDIIIHTTELKSANKKQKLVAEIDRKDAAEKLSHLIIYGEATEELPYLQAVMRLHPSVGLMLERYVPPEGVTIASHFMP

AGTIVGVNTWVMHRNPAIFPSPEEFRPERWSESTPAQLKVMAQTMFQFGAGSRIWLGRWMSLIEMSKVVPQIFREFDVEL

ADPEKEWSVRNVWFVQQSGVICRLAHRT*

>CYP6001C35|892777|Sphbr2

MSNSQVTAFDRESPGIFQRLRSLLEAVLRPPPPQLGDGLTDAEAEPTKVKETGIKEDLQSQGTRITKDIELLLDSLETFK

NGGLINDRDYVVEKLIQLASTLPPHSRNQIKLTGQLIRSLWNILPHPPLQYIGPSYLGSLPAPPPSDQRYRSADGSYYNV

MLPNLGRAGSAYARSVAPKTVLPPNLPDAGVLFDTMFARDKIIEHPTKMSSMLFYLATIIIHDVFKTDPMDKSRSNTSSY

LDLAPLYGNNITEQRQVRTMADGKLKPDCFSEARILGFPPGVSVFLICFNRFHNYAASQLAVINEKGRFTKPSPLGQNPT

PEDEFKYHEALVKYDEELFQTARLVTCGLYVQIILNDYVKNILNFNRTKSSWSLDPRGDYDHVFDQTTAIPSAMGNQVSA

EFNLIYRWHACVGERDEKWTQDFFKDELKVDNPENLSVEELQILLKKWGHSIPREPEQRVMPGWKRQADGRFADADLVAE

LTKSTEAVAGSFGPKNIPKVLRSIEILGIEQARDWNMATLNEFRQFCGLRAHTTFEDINPDPEIARSLRIFYNHPDFVEL

YPGLLAEDAKEAMIPGSGLCASFTISKAILSDAVALVRSDRFYTTDFTPANLTNWGFNEVSTNPDLVNGRVIYKLLYTAF

PGWYRADSVYAMFPFTVPVETKEILENLGTVDDFDFTTPSFVPDWTPIFSYKGVVNVLEDPSRFRVPWGPHIYQMTNQDY

MLSGDEPANKKGRDDMVACMYGPKGGLDQITKFYEDTTLKLLKQKSYRIGSSYEVDVVRDVINLAHTHFSAAMFAIPLKT

EENPHGIFTEQELYKIHAILFAWVFLDADPGKSFQLRQATRQAAEAFKGVVEPICRAIKLGKPILDVIDALHKDHGPMSI

YGKHMIERIMKGKSVAETVGQILPTAGAAVARQSQGSIQMLEIFFTEKYQHHWPTIQALARDKTDPEAAFQKLRKYALEA

SRLSPAAYGVTRNMVAESATIDDGPNRTVNVKKGETVFVDFITACLDPEVFPNPHEIDLTRPEEIYLQYGYGPHSCIGAP

IVINALASMLRVFGRLENLRRAPGPAGEMKYTLLKGQFRMYMTEDWSSWWPFPTTMKLHYDGIVE*

>CYP6001C29|925366|Sphbr2

MAENGSGSNGKTAVSTWASEPTSAPLAPVSTPVAKRPIKTGRNDVEDTFRKFASLIHASNRPLPNRYGSGQDGPSEEEMT

GIRHDIAVLKKGGFLKESVQTLLMFANGKRKGLPVDDKTMIMEKMIQLTSRLPPTSKLRVKLTTAQVDTLWDSLQHPPLS

YCGPKFTYRQADGSHNNIMEPTLGQAGSPYARSVKPMTKMPGAPPDAYTVFDAIFSRGKDGENYRPSNNNISSMLFYIAS

IIIHDLFRTNRADPTISDTSSYLDLSPLYGINQEQQNSVRAFKDGKLKNDVFAEKRLLAFPPGVSVLLLMFARFHNHVAT

QLKEINEGGRFDLKFDRRWHNDDQETKNAKALKQQEEDLFQTARLVTCGLYINFVLNDYLRTIVNLNRVDTTWTLDPRFD

PSKMYNPDGTPAGVGNMVSVEFNLVYRWHSCISKRDDEWTQEFYKSLFPGKDTDKLTLQEFMVGVHIWENSIPADPAERT

IENFVRQPDGHFKDDDLVKVLMESIEDPAGAFGARNVPHVLRLVEVLGIEQTRKWKVASLNEFREFFGLAKHKTFESINP

DPVVANTLRQLYDHPDFVELYPGIVAEDDKEPMVPGVGIGPTYTVSRAILSDAVTLVRSDRFYTVDYTAGALTNWGIEEA

SSNPNVMYGCVGYKLILKAFPNHFKYNSIYACHPLTIPLENRKIFTALGLVHEYDFERPKFIRPRIPVTSYALTKQILND

DTNFKVTWGAGFDYIMEAPFMLSGDGAPFATMRKFVGDRIYLAGVNWRSQIRAFYKELTVKLIRKKAYSITGAGCYQVDA

VRDIGNMAQTIFAASIFNLPLKTEDHPKGIYTEQELYMVLCAMFIAIFFDMDSSKSFPLRHAAYAATRQFGAVVEAQVKA

VKSWGWLQGVWDPLNIRGRNKSPLHDYGYHMISRLLESGESPASVTWKYIVPTAGASAPNQAQLFAQVLDFYLQDENAHH

LAEIQRLAALDTDESWETIKKYALEGGRLAGTFGLYRRFDGASMTVHDGAHPIELVKDDLVFVSFVSASRDPIVFPDPLE

IRLDRPEEFYMQYGDGPHECLGKAANIIGLTTMLMEFGKLPGLRRAPGPAGMMKTIPKPGGFKIYMKEDWSSFWPFPTCM

KVRFDDII*

>CYP505A68|959165|Sphbr2

MSISNLAAALARLETDDDTPPPAKASTPLPPPCPPGLPLVGNLAELDRDTPLTTFLRLADTYGPIYSLQLGGGIRRVFIN

SVELLEEVCDESRFRKVVSGSLVELRAGTGAGLFTAHHGEHEWAIAHRILMPAFGPMAIREMFDDMHDIASQLVMKWARY

GPQNRILATDDFTRLTLDTLALCTMDYRFNSFYQSEMHEFVDAMVDFLVESGKRANRPKIAGLFLRGTNMKYDADIKLMK

DVAAGVIKARREKPSDKKDLLNAMINGRDPKTGEGLSDDLIIANMITFLIAGHETTSGMLSFAFYGLLRNSSAYRAAQQE

VDEVCGKGPITVEHIPKLKYIAAVLRETLRLNPTASIFAVSPHPDLNEDPVTIGNGKYALEKGQPIHAVLPKIHRDPKVY

GDDAEEFRPERMLDENFEKLPSAAWKPFGNGMRGCIGRPFAWQEAILVTALLLQNFNFQLDDPNYRLQIKQTLTIKPKDF

YMRATLRDGVDPIHLEKALAAGDHGSVKTKQSVSKAESGGEKKPRKQMSIFYGSNTGTCETLAQRLSTNAVAHGFDASVA

PLDSATEKLSKDRPVVIITASYEGQPPDNAAHFVEWLQSLSGDEVSGTNFAVFGCGHKDWRTTFQRIPNLVDDLLEARGG

HRIVKRGLADASTGDMFTEFDSWEDGMFWPAVTERFGGGGTIDASSSVVTVEISSQTRSSNLRIDVRNARVKSVGVLTTG

GEKEKHHIEIELPSDMPYRAGDYLAVLPVNPKEDVKRAMKRFQLPWDAQLKISSNAPTSLPTNCDISARAVLDAYVELSQ

PATKKNVLALAAAAGEDHAVKAELEALAAEKFQDEITAKRTSPLDLLERYPKLNVPIGDFLAMLPPMRVRQYSMSSSPLQ

NPETCTLTYSRVEGDARSGAPRRHLGVATSYLASLEPGDILQVSVRASAKPFHLPLNPETTPIVMICAGTGLAPFRGFVQ

ERAVQIAAGRKLAKALLFVGCRYHDRDRLYADEFDHWEKQGAVELRYAFSREPEASLGCRYVQDRMRREKDELHEAWESG

AKVFVCGGAKVGEAVAEVCKELYRNKAKELGKEKTEELVEEWFAALRNERFATDVF

Terfezia boudieri ATCC MYA-4762 v1.1

>CYP51046A1|139181|Terbo2

MFPLLLLAAICYPLYHTYCLLRNIQIARSTGLPYLILPFNQYSLPWIVLGRALYPLLATLLPFFPRLRWIRPDWFWARKY

AMYREVGADVFAMVSASGVFIVVCDADVVVDVLARKDDFPKPPVVHPGLDIYGRNVVTTSGAEWRRHRKVVTPHFGESYN

RLAWTKGVEKTSELVKHWSTRLLHETDGPVLRNVENDFIRLTMNITTHNMFGTNLRWPNRIAPSSQTDDGYDEDGPPNDE

VLAPNHKMSYQASIKHMLNNCNMLMVAPSWYLKWTPNATHRKAGVACRELGVYLNEMGLTEQEVIGETFMLIFTGHATTA

NALHNTITMLAIYPEYQIKLQEDLDRILGDRVPEYENDYPALAESWAGAIFHEINRLYPALPLVPKFTPSPQYITVNGTK

HLIPAHTPVLLDCCNTQRNPKYWVPPHETEAVAAPDEFRPQRWLNAPGESQNTNNPNGNFLKPYKGSYYPWSDGQRVCLG

KKYSVVELLAIYAEVFRGHSVELDVGAGETWEEARGRAKRYMERVDAGLLLKTVGKEPGLRYVKRGEERFFPRR*

>CYP6453A3|771110|Terbo2

LLYPICLVIYRLYFHPLARFPGPKLAAATSWYEFYWNAVKDGQATHKRKEWHEKYGPVVRINPNEIHITTPAAYNEIYLK

TKPVFNKYYPFYRMFLADTASIGIMDNHAHRARRELLTPLFCRQNVIDMERVIWKTVYSLCGKLQAYTPQSGQSQAGRKI

GIKVLFKMLTSDIISDFCYGRSFESVEKAEIETTNAGIDKAIPPRFIRAMAGAAQGFWFMQRFWRLQLFLVNLPDWFVNI

FHIERSLGMRDLITVRCLHVARCKYSLKNLFSQLLFPSRPQYNPQNYKSQAYKHPTAKELLEEAITVMGGGVEEASNAMM
YGIYYILTTDGIAEEVRKELRRIWPDKNSPITFLELERSSYFMGCIKEIIRMSVPVPGKLPRVVPPGGFNVDGWHVPSGS
IVSMSANMQNFHPEIWYEPNQFIPERWLEKDGTPRRDMDRNLCSFSKGNRMCLGSNLATAELYIALAILFRRFDL
>CYP50042A10|779725|Terbo2
MAILTSYILSGVSPAELAGILIFLCALKVTFSLYHYNPNPSKLPVVGIPPGPFGRIRAIPASIRHYHSIIADGYLRYHKQ
KNPTAFMAPWIMSHYIHVIPLQLISEHKSAPENVLSQAVAFQEEYGVLAIGGSEIFTHSYHVPILRQKLTASLPRILDPM
VEELEMSFKEEWEQPWVQRKNAEGWVEMPIFEETLKIVSRTTNRVFGGTVTSRDKVFLKNSIDFAIGAIMASFGVQLFPR
GMHTDGSRLVEPLLSFRNRRRMAFAAKILTPIIEERRAIIERNSKDEGSPEEPDDLLQYLLHQAIGLKAPHDSSYQVSCR
YLMINFAAIHTTSLTFCNSIANMAAYTDELTGCSYWDLLREEVEAVDRESEEGPGVWTKRKLNKLVGLDSFIRETLRKNV
TSPVGLVRKVMPKEGYTYANGLHVKHGELVGMPTLSVHFDDDTTGKQALDFIGFRYCRPYQELSGQAATDISATGGTGKL
AAVTTADEYLSFGHGKHACPGRFFGVIELKMFLKFCLLNYEIQPTKRAPTQYLWANPTPPFNMVIKMRKRRVD*
>CYP573C2|783449|Terbo2
MLPHRTQVIDINGELIGEFISDARFLIRAHLGLIILGLMYFGLVSHVVKNRYFHPLSKFPGPFWGSITNLYCSLMILTGE
SQKMEFDFHKKYGPVVRIAPNLLLVSDPRMLPVIYQRKADKTDFYITGTLGKGAESVFNTRAHHDHYRLRKRLAGAYHLK
ATKGMETLVGERISEWTAKLGRTFADTGHILDLAMWSQYLAYDVITELGFGEAMGFVRQGEDVGGLIKCFHYALPAIGSL
ARLPILTKMIVGWDWVTAKPTDNHGLGPVMAFRDRCIEQRQAAAGSNSRQDLLQNLFDQRTADGQPAMSVDQIKAETLIV
LLAGSDTPASAFRACVLYTLTTPGVYEKLMEEINTAVDQNALSSPVVTFDEAKKLRYFSACLREAMRLSPSAPVLLPRLV
PHGGVMLCGQFVPEGAEVAANPWVIHRSKELYGEDAEEYRPERWLESEEKTKEMDKYDFQFGYGDRSCLGKSIALMELYK
GLVQFFRLFSPELVNPKRPCVYRNMGVAVHTDFWVRVKNRGIDST*
>CYP567Q1|786861|Terbo2
WALWRVVYNLNFHPLAKFPGPWLAAASTLWFEHDILSGRSPFKLAEAHKKYGPIMRIAPNQLSVADVRAWKEIHGHHPRE
KSFLKGDFYNDDVKNFGLENIFTMKDAVKHAEMRRMLAYAFSTKALTDQEGIIQGYIDLLIKRLGQRYAEKERGPDGEYC
NMVTWYNYITFDIIGDLAFGESTAFACLKEQKEHPWVTIMLDGFYLNALNESLRRICGTRKLAHWLIPKSLKMKRMRMDA
YARNLVVNRLASQTNRKDFWHYILNRPESKDTSVNELCMQAQPLVAAGSITTSTTLSGITFYLLKYSRVYNILKDEIRSS
FKSYADITEQNTVRMQYLNAVIEEGLRLYPAAPGGMPRISPGATVAGHYIPRGTDIAVPLFTAARDPANWSVPNEMYPER
WIDPNNTDKKEASQPFLLGPRGCLGKNLAYMELRNILAKVVYAYDLELVDKDLDWEKKSRAWSVWWKPELHVKVTKRPGL
RWTADDIPL*
>CYP584AN1|823137|Terbo2
MLSVTSLTIGGIFLAFLITKLIYQYQLRKFARSRGCLPPILISRWPLGIDTFRESMHNFKNMRFCKMSNGRHVTYGDTFK
LNVLGDVVIITRHPENVKAILSTQFDEFYLAHRIGGKLDLLLGKHGIFVQWGPAWAHSRALLRPQFNKGQIVNDMDSLEF
HVKRMISLLPDNKVFDIQPYFFALTMDTATEFLFGESVESLLTEKGDEPAKGTFPEAFNTASWWISKKVKFGPWHWAVGN
KETARGCEISRNFVGKFVDKALAMDPGKKKEGEQKYVFLNALAEEYKDRTILTDQIINILLAGRDTTAALLAFVMWFLAR
NKRVWHKLRAEVLERVGKENKPTWEIIKDMKYLKYVLNETLRLLPAVPMNTRSAAKRTTLPCGGGPDQKSPIVVEKNSLI
HYSVYSMHRRREVYGDDAEEFNPDRWETLKPGAWDYLPFNGGPRICLGQQYALNEASYAIIRVLQKYQDIVAIDPDTGKE
VPGWGEDKTDKMFKERLGLTLSSMDGVHVRLTPTA*
>CYP5242B1|832017|Terbo2
MTSFAIFLTALTNALQLSPRLLYTLALLLVPSSLILLSRIIGNAVTDFRTKDGRKMVVLKQDTRVSRFTHGPALSQEGKR
LAGIDPYLVRNGGNLEVVIHSPEHVRDFFAKDGKDHLKPPNANFGVYFGRMLGECVGMLNGSVWDKAKVHLIPHFAHGAA
MAAVPMFKAEYLRWVEKLPEDKLVSYKRSDGSGFVIDALSACRKLPFKLIAMVLYRDMLTDKLFDELWELNEVHERITYA
ALLRDLPAKKWYSFLPTRDNKLLEAYLKDWARLNMAVINEARVTGTYCPVTEMYKGVENGDMTLEQFLQSLDEILFTNVD
VTSSIFAYVLINLARDREFQSALRQEILSYSHGLDAYITDDEALLHFAYLEALRVNPASWFSIPETTGPDPKYIGSFLIP
PHTSVIIDLKILNTQSPIWGSDGHIFRPLRWRGMSPSAVRYSFHRYGMGPRKCMGKNVANILIKMLMVTLLEKYEVVADA

EEGEGKYRTDRFTRAPEKEVEFRVLLGKGGGQ*

>CYP548BE4|839093|Terbo2

MFTWLTYDVLGELCFGKAFGMLVDEGQRFVAGLIDNATHNHHICGHYLPLSTLNLGRLLFPTIAAERWRFILHSRACTND

RMALHASSLDKDKRDFFHYLLSAVDPETGKGFETVELWGEANVLIIAGSDTTSSALAATLYYLVQNPDKLEIVKKEVRNK

FNSVEEITSGKELNECVYLRACIDEAMRLCPPVPGLLPREVINPKGMTIDCTKTGGGKYFLPKGTTIGGNIYTMHHSPMH

YPQPFKFHPERWLLPTDHPLGTATQESLVLAREAYTPFSIGSRGCIGKALALMELRLVMGRMMWEWEVEGVWGVEGKGEK

WNGGFRWEGLDEKGQEEGWEGEFRIFDHFTTRKEGPVVRFEKRVLT*

>CYP5093H4|851979|Terbo2

MSAHHKNFVNSITQIQTVGSQVLTMESTITEPAPQLGTFSSAIQSRLPMASLNTEPAFEPYPHGLVYALCTNEPDATPSP

FSPILGLSILLLLFLVAMDYQRSLKMPPGPLPIPILGNKWALPLRKPWYKLKQWTDEYGSIVTIWTGRRPTIVIGDPKVA

CDLLERRSAKYSSRPRFVVLGELFTNNDALLTMPYGERWRATKKIFHLGLCKNACDSYKPIQESESRRLARDLLRDPSRF

GKHLERYTASVMFCVAYGRRVDTLDDPLIEKIYERMSYMATLNVPGAFWAESYPILKLIPDCIAPWKREVKRRGAESTKM

LLDLANAVKLQIEAPESFTKTLWKKHMEQPDSMTEREIAYATGSLFGAGSDTTASTLQSFILAMTCFPHTAKKAQEELDR

VIPKSRSPTWEDMPNLPYCNAVIKETLRWRPVAVMGGTPHATTQDDYYNGLFIPKGTTVMGNLWAIHHNEKYFNDSHNFI

PERYLGEVQSGLDKYPSRDGHSAFGWGRRICPGKLLAENSLFITITRILWAFNISKAKDESGVEITPNIFDYTDGFNSKP

QPFFSDIKVRSPAIEEVINSEAQEGERFLGRYLYERK*

>CYP617V4|858580|Terbo2

MRALLHGSIARINCTDQLHRSLVLGRPPKLSSPVPSPGYNNLVCTFVYIFSAGLFAKLTYGILIYPHFFSPLRHIPGPKC

ISIWNGQFPTIHRQPSGQPHIKWMDEVPNDGLIRYLAFCNADRVFPTSPKLLQEVMHTKGYIFQKAPIVRRSIGKMFGMH

GMLFSEGEQHKVQRRHMLPAFSYRHLRDLIPNFWEKSINLNKVMENDLRTRQDADTQKQIGMVVEISQWLNRATLDIIGS

AGFGYEFNCINNNDESNELAQAYRKVFQPVSKGVQLQRVVFQYLPDWLIKLIPSKRRKESAEAIEMVKGMCMRMVREKKR

EYETEGKAGQKGLREIDILSLVTKDSPFTEEEMRDQLMTFLAAGHETTASALTWALFLLSQNPEIQSRLRAEIRSQIPSP

TSTATPAVPLEDYLDTLPYLRNVTNEVFRFFPPVPMTLRMAGEDTTLGRHYIPKGTIVMISPWAINRSKKVWGPRADVFD

PDRWEAYKEGKEGASNYDFMTFLHGPRSCIGKDFSRLEFKALLAALVGKFEFNEVLDKNGRRKELVIKSGITSRPENGLP

LWVRSLDGW*

>CYP548BD5|866124|Terbo2

MGWLLSISTIILPNGWNEIPQLLFFTVGGLLTTLVFYRLYFHPLAEYPGPLLARCTSLHAAYHAWIGDSHLVLYHAHRKY

GKFVRFTPNCISVNDAVAVNEIYGNGRNVQKSSFYSVMPPYPHATDIHSVIDKALHGRKRRVMAQAFLETAIKGMEDTVL

THIRTFVRSLGTDSCENENPGIGESNASRNMVYRSIWLTFDVTGDLSFGKSFDMLTREEWRFWPTLIDRAIHRHAIAGIS

IKIHKLGLGNYLFPAIAEGQKTFIKYSKEQAKERVALGAGVRKDFFHYLLNAKDPQTGEGYSMSELWGESNVLIVAGSHT

PSVALAATFFFLANYPKVLAELTTLVRTTFSDCEEIRSEKFSTPECQPAYKLVRACLDEAMRMAPPIPTTLLRQVLKGGI

TIAGKYFPEGVDIATPCFTLHMNEEYYPDPFTYNPKRFLADKAESLEAFQRARNAFAPFSIGPRNCIGRSLAYLELTLTL

ARTVWMYDFVYVGGGRETRLDNIAFVEKLGGNKNPILYRFDDHFTAFVSREGPKIKFIRRGDF*

>CYP6902B1|866192|Terbo2

MSLFTQALLGFVVFYPIYHIYCTWRNVLKARSTGLPYIVTPWNGHSLHWMFLEPILIPWLKKLPGFIVNPWISYTEANWE

WRRKHDVYKDMKSDLFWVISGSIMVLWVSDADVVNEIITRRDDFTKPGERYAVLDLFGSNVLSTTGKHWRAHRKITAPPF

NEKNNLNLSDDSMRVALHVIAWAGFSQKLKWPIRISEDKYDEKSPEDGKLERGHKLSFQAAIHTVLVNIYLILGSPRLYL

KYSPIKSHRRVYEAYMEFGTYMKEMVANRKLEMENGTATVGDILGAIVRGYYNGEKAGKGEAVITEQEVFGNCFIMILAG

HETTANVIRYSIMMLAMYPEPQIKLQDDINRILGDQEPDYERNYQALAERWCGAIMIHLDAVAVHRNPRFWVPEGKTEEE

AQPNNYNPERWLLVTKRTEATDFGEEVGLTPEGKDTSKAFYRPHRGSFIPFSDGARACLGRKFANVEFVGVMAVLFRDYS

VELEVKEGESWEDAKRRAWEYVEDSGVILTLKPKRPDIGIRWVKRGEEKYFPDR*

>CYP61A1|886128|Terbo2

MASQSSPHAVPGDSSPEMLGGNMVSQLFGGVSYWSLAVTLFILCVTYDQAKYQWNKWGNIVGPAFKLPFMGPFLESIDPK
FEEYAAKWASGPLSCVSVFHKFVVIASTRDLARKVLNSPMYVRPCVVDIAKKILRPTNWVFLDGKAHVDYRKGLNGLFTR
KALSEYLPGQEDMHKRYFKRWIQISKKTPNVKFMPEFREINCAVSCRTFVGNYSSDEKVKKISDDYYKVTAALDLVNFPI
IIPYTRTWYGKKCADMVLEEFSECAQKSKDHMAAGGVPHCLLDYWVKSMFEYKAYEERLARGETLAPGEKAPASIRMFTN
WEIAQTIFTFLFASQDASSSATTWLFQIMADRPDCLEKVREEALRVRGGDPYKPADIDMLDQMVYTRAVVKECLRYRPPV
TMVPYEVKKSFPVAEGYIVPKGSMIVPTLYPALHDPEVYVDPETFNPDRWLEGGEAEAAVKNWLVFGTGPHYCLGQTYAT
YNFMAMISLASLMLEWEHHVTPKSEKIKVFATIFPMDDCFLTFKERLPILD*
>CYP6001H1|801144|Terbo2
MSLEIPLDVLNHLGSEAPEDILHQGASRPPPVSHFRRPDGSLGNIRNEELGRAGCPFARIIRTEAEIHGAKPEPEKLIDG
LMLRGRKRHTPGVSMLFFYYSSLIAYDIIYESPQDMNINDTSCYLDLTTLYGRNQGEQNSVRTFKNGLLKPDVYADPRPS
ITPGVAVLLVLFNRFHNFAARTILAINEKGKFTPPADKPKHEQDKWLDEQLFQTARAVTVGLYVNIILHDYLRTLTKSPF
KKGKDPHVTLHDFFDKQGLPKGAGNQVSIESNLLYRLYFAIPRTDTSWLQESYRKSFPQKDPAGLSDLELLKALDDHAQA
NPLEPSQRTFGALKRGKDGVVLEADLLKVWLHIVEKEAGHYGVNISPDVYKPLAKMAIEQARKWHAPSLNEFRKFLKLAP
FKKFEEVHSDPDVATNLKNLYEDIDHVELYPGLYFEEQEEDSVGSSTAVRNMLASTLSLVRGDRFLTHDCTAANLTAWGF
KEISSDPTILGGAKIFLLVLNALGSYIVFNSVYAMLPFYIPKRSEEIVKKLKIAYLFSFTRPAGLRPPPVPITTYAGLTT
VLGDQEKFWVPWAPHMTSLSTFMLASDKPESAKQKRDLGEAIYGQKDALKDFEAFAMKLTHEFLTKKSHTIGDGPGLQNV
IDIVKDVADLVATHFVAKLAYLPLNCSTGRSYTEVELAKLLGDIFIYIFADSDPTKSWARRRDGDNPCAKLVDAMVERVK
HVQDTVAADSGSTSECPVSGSAVASDDEPLANYGIMLVKRLLATNRSVREVAEILSGIAVGFHTNSSMLFTQMVNFYLDK
ANAKYWEDIKTLAANPSADSDSDVKLQGYVLEGYRLSLTLGLLRYTAPGVDMEIEGHKIKGGDVIFTNLITASRDASAFP
NPTEVNTNRPIDSYVHYGYGPHRCFGRPLNMVYARGMLKVLAQLPGLERAPGDDGVLHYIAFPSGLKSYLSADWGELSPL
PSTWKLVWKGEPKGVSCTH*
>CYP6685A1|829312|Terbo2
MTLQEILYHSALLSHQSVQLILVLCAVYLVLESISTIRLWLQRCRLVREYGCQPVAHSVGSWPLGLNAILEEKRANEAND
LPTLMDERFNKYGHTLGISGLRHMKYITCEPRIVQTVLSTHFGNFPKRPLITVASTFLGDHSIFMLDGPKWEHARAMLKP
QFARDQVADFTDLEKHVERFLTRAYPFPLTNAEIKGESKQVAFTTDIQPLIFDLLFDSVTENLLGESADTQLRKEAGLAD
DVIAFENAVELAAQVAAIQVGLGKLYSWLEMNIKYSQACKTNFEYVRPYVEKALHLHSMKMRAAHTLANGGKETSKAKKW
VALNELAAVDEDLTQSKAFKDALASHAISLLVAGRDTTSAMLSWAIHLLARNPQVYSKLRQEILKAFNKDNGSMQLPDYP
TLKALPYVRWVLKETLRLYPVVTVNERIANKDTVLPIGGGPDGQAPLLLKAGEGIAWSLHSMHRRKDLYGDDAAEFRPER
WGEDKKGQGLRTIGWGYLPFHGGPRTCMGQQRALNEGSYILARIAQTFSAIQVSDDIDIEKDRPQFKMTITTTSSNGVWV
KLTKAA*
>CYP539A45|832996|Terbo2
MSLDSLTQLPASTILLLGVGILVAWVGVSKAYESYKLRKLGKRPPAAPTWIPLLGLDIIQDSMKNARNYTSYDGWKDKSE
RVGYTFEMTLLLNRLVFTVEPENMKALLTSQFNDFGKGEKFHKGWFPFLGDSIFATDGDKWHNSRQLIRPQFIKERVSDL
HTFETHIQKMISLIREAQGQTIDVQDLFFRLTLDAATDFLLGSSVNSLDSPQVEFAEAFAIVQKTHTDMERLGALHHFIR
FPKYKKSLEILNRFVNQFVHQVVRMTPEELESKGSHNYNFLHALAGFTRDPKTLRDQLVAVLLAGRDTTAVTLSWALYEL
SRKPEVVQRLRQEILETVGPMEPPTYENLKNMKYLQHIINETLRLYPAVPFNVRTALKDTTFPVGGGPDRKQPIAILKGT
QVAYGPIIMQRRKDLFGPDADEFKPERWNNWTPKAWQYIPFNGGPRICIGQQFALAEMGYCLVRLFQSFEEVNYMGTKPP
RMRCEITVSAAGGVEVAFREASKA*
>CYP6001C33|864289|Terbo2
MDKENDLQIQPANITSNGDSNDHSNDHSNDHSNGHPNGASNGKTKATPVPLKGSDPNWKHKGAKERPTRKEMEQTMGALT
NLLHASNKPLPNRYGDGKERKSIYDEKYEGVWRDIKALVKQGNLKESYKTISTVGKHKKHGGYTDDKTYIMEYVIRLSSH
LPANSMTRLKLTAQQVDQLWNTLQHPPLSLLGNAHKFRQPDGAYNNPMHPNLGRAGTAYARTVKPMTLSPMSLPDAGLVF

DSVMSRREYRSHPNNVSSMLFYVASIIIHDVFRTNRTDININDTSSYLDLSPLYGNNLEDQKNMRTFKDGKLKPDCYNEK
RILAFPPGVSVLLVMFNRFHNYAAEQLAGINEGGRFDLKINQRDPDREGATRRAEEAREEELFQTARLVTCGLYINMILS
DYLRTIVNLNRTDSTWTLDPRFEPSRIYNPNATEMGTGNCCSVEFNLVYRWHSAISKRDEIWTEELYKEIFGEHVDAVDM
PLMDFLKGVGHWEASLPEDPAKRTFNKLERNSQTGMFEDDDLVQILTESIEDPAGAFGARNVPAILRAVEIMGIEQGRKW
RVSSLNEFRAFFGLEPHKTFESINRDPEVADALRQLYDHPDFVELYPGLVAEEGKVPMVPGVGISPTFTISRAILSDAVC
LVRGDRFYTIDYTPANLTNWGFQEVASDLNVLNGCVFYKLFLKCFPNHFTWNSIYAMYPLTIPSENERIMKNLGKYDLFD
YSPPKYVKPRVPILSYATTKRILEDKQTYNVTWDGFTWIYGKEFMLSGDTDWHEGMRQFVGECIFGQQAWEREVKQFYED
TCTRLLKDKRYRIPSPGGKGPGCWQIDAVRDVNNLVQANFAATLWGLPLKTKDNPKDFFTEQEMQQILTLMFVCIFFDVD
PSKSFPLRYAVQQLVRQFGKLVEFEVKAVHSPIGAMLSGRYFKKRNTSALQSYGLKMIQRLLEGGSSPEDVAWSYILPAA
GASNANQGQVFAQVLDFYLRPENALHLAEIQRLAKLDTPDADELIKRYALEGTRLAGTFGLYRKVNCGDGETITLQDGQR
TVNLQKGDRIFVSFISASRDSSVFPDPLLVKLDRPMESYLQYGTGPHKCLGANINMVSLPTMLKFFGKLKGLRRAPGIAG
ELKAVEKEGGFKVYMREDWSGYWPFPVNMKIRFDGVEGWEGE*

>CYP548BE4|849246|Terbo2

MDHFTSSLPHLSSTHIGNFLTSPLSLPSISTLLTGFTYLTSIAFISLLTVSLYRVLLHPLSQYPGPILCKLTPLVATWHA
YKGDRHLFLHRLHKKYGPIVRWAPNAVSIDSASALKEIYGHGINARNVQKADFYEAFPAVKGVHNTHNCINKAEHARKRR
VLSQAFSENALKGLESLVLKNVQVFFEVVEERMKGKAGKEKMVGKGGKEGLDMGEMFTWLTYDVLGELCFGKAFGMLVDE
GQRFVAGLIDNATHNHHICGHYLPLSTLNLGRLLFPTIAAERWRFILHSRACTNDRMALHASGLDKDKRDFFHYLLSAVD
PETGKGFETVELWGEANVLIIAGSDTTSSALAATLYYLVQNPDKLEIVKKEVRNKFNSVEEIASGKELNECVYLRACIDE
AMRLCPPVPGLLPREVINPKGMTIDCTKTGGGKYFLPKGTTIGFNIYTMHHSPVHYPQPFKFHPERWLLPTDHPLGTATQ
ESLVLAREAYTPFSIGSRGCIGKALALMELRLVMGRMIWEWEVEGVWGVEGKGEKWNGGYRWEGLDEMGQEEGWKGEFRI
FDHFTTRKEGPVAKANLNLHHFIDQLMALAILSPIAGSLKSASIRIGTRNILMTPSARMTMYLYARPTLTQRDYVNPSLA
DVYPARLQQVELELHLALEGCWATQDTSHLIMSSPTSEAPASTSTSISASTPPSEYPPIIPGGSLIIAYQIRNKRVLIIG
GGNVAASRLYSILCADANVTLIAPHPLHPEIQHCLTTHANQVTHIPRPFSATEDITPETAPTYALVLTAIDDPTTSSQIY
NLCHTFRIPVNIADVPSECDFYFGSIHRDGPLQIMVSTNGNGPKIANLIRRKIADSLPPNVGDAITKVGELRRKLRKAAP
TLEEGPKRMEWMTKVSETWSLEELGGMGEEEMDRLLDNWMVWKKSGGDVPSYRKLMEKKKDEEVVEEAVSGAGGWFGSLG
WF*

>CYP51F1|862473|Terbo2

MPAKKDQSVLKRNLPPSSPSSPESSAKKPRRSQRINPVVQNEAKEERLVHTASPSRPAKDGATTPRSSSPPPFQDTQVAS
QVHFTQPDHTEEAKDIWGYLTPMNSGNGEVLTLSKRTSCSKKPDYNASTKKGTKKPSVGAGGYLIGRHAECDIVVETPVV
SNRHCVIFKETSGAEPVAILEDLSSNGTCVNGTIVGRNHRRTLKTGDEISIAGAAIFVFRYPHHTTAGRFEEAYTMGQRL
GSGHFATVHIATEKRTGTNYAVKIFRKPKAEEKSRLTGLHQEIAVLMSVSHPNVLCLKETFDQDDGVYLVLELASEGELF
EYIIQQGKLSEEETRKVFAQLLQGLKYLHERNIVHRDIKPENILLCDKNLSVKLADFGLAKIIGEDSFTTSLCGTPSYVA
PEILENHKNRKYSKAVDVWSLGVVLYICLCGFPPFSDELYSEDFQYNLSQQIKLGLFEYPMPYWSNISDEALDLIDRMLT
VNPDERITVQRALEHPWTQKRRMAPPGITESTESLDGAFNAMGFSKRKVERERTLLSNAVKNSRKKPDQEKPVVVKEVET
PSEEAKANSSISTPAATTAFMEIGGKGVYAYVRRLYSYGCRLDISPDMAFIFVLGWLGMRLHICSECEKAAEPHISILLY
TLFLAKRVTLNMGVLSALVDQLNPVTQRLAQQSTPVLVFGGIFAFCVITTVLNIIQQLLFVDSNKPPVVFHIFPPFFGSTI
NYGIDPYKFFFACQEKYGDTFTFILLGRKMTVYLGPKGNDFIFNGKLSQVCAEEAYTHLTTPVFGTDVVYDCPNHKLMDQ
KRFMKFGLTSETFRAYVPLIIEEVETYLKNSPLFSARKGPVDLLAVIPELTIFTASRTLQGKEIRKGFTGEVAGLYHDLD
AGFTPMNFMFPWFPFPQNKRRDAAQRKMAQTYMDIITKRRAAAARGKQPEEKDMIWNLMNQKYKDGTVVPDKEIAHMMIA
LLMAGQHTSMATMTWILLHLASRPDVVQDLYEEQLRIFGKDLEPLTYESLGELTLHNYVIKETLRIHPPLHSIMRKVKSP
MRVPNTHWVIPDNHYLMAAPGVSAIDGKYFKNPTAYDPYRWAGEKVTIEEEEEKFDFGYGLVSKGTSSPYLPFGAGRHRC
IGEQFANVQLGTLLAMFVRLLKMNLPDGQKEPAPPDYASLIAMPQRPATMIYEWRKPEKK*

Terfezia claveryi T7 v1.0

>CYP6453A3|1088007|Tercla1

MTAFAGLFDETTSDYLTSVLFHVPFSLLVRGILLPLGLLYPICLVIYRLYFHPLARFPGPKLAAATSWHEFYWNFVKDGQ

ATHKRKEWHEKYGPVVRINPNEIHITTPAAYNEIYLKTKPVFNKYYPFYRMFLADTASIGIMDNHAHRARRELLTPLFCR

QNVIDMERVIWKTVYSLCDKLQAYTPQSGQSQAGRKIGTKVLFKMLTSDIISDFCYGRSFESVEKVEIKTTKAGIDKAIP

PRFIRALAGAAQGFWFMQRFWRLQLFLVNLPDWFVNIFHIEASLGMRDLMTVRCLHVSRAKHKNLFSQLLFPSTPHYNPQ

SDKIQAYKQPTAKEMLEEAITVMGGGVEEASNAMMYGIYYMLATDGISEKVGKELRRIWPDKNSPITFLELERSSYFMGC

IKEIIRMSVPVPGKLPRVVPSGGFNVDGWHVPSGSIISMSANMQNFHPEIWYEPNQFIPERWLEKDGTPRRDMDRNLCSF

SKGNRMCLGSNLATAELYITLAILFRRFDLEIVEEDTDMRWVDRVAAQSVGELVLLVRELREGS*

>CYP5192F2|1090047|Tercla1

MAVLQLQPIILAFALLLGAWLVKFFREGYQWRKKFQDKTLALPCPPHNWIFGHLPVMVKLFQKVPRDAHPQLYGAVIGPD

YKLPKLYYMDNWPFGPSNLVITDPEIAQQVTVLASYDKHPELMKFTKPLAGDNNLVSSNGETWKKWRRIFNPGFAIGHLM

GMVPEIVEDTEAFVKALGKLADSGAVFRLEDIATRFTVDVIGRVTMDVQFHAQEADHPLINAFRSQVRWLPRALAPNPFY

NLNPIRYFMMRRNVKTMRGFLAAEIEKRLTTFDFTLEKGNAEFRKKRSIIDLALQEYLKEARAEGRMITKLDEEFKENAI

TQALIFIFAGHDTTSSTICYIYHLLSKHPESLARIRAEHNEILGTDPSAAADIIRNDPHIINKLHYTLAVIKETLRIWPP

ASTIRNGTPGLNITDPETGKTYPTDGFLVWPVVFGIHRSARNWRRPSEFIPERFMPNPPEGLVPLPNAWRPFEKGPRNCI

GQELALIESKIAIALTIRTFEFEVQYDNEGTFEEGLKPEQGKVDNFMGDKAYQVLLGTAKPKEGMPSVVRRVKV*

>CYP51046A2|1100367|Tercla1

MFPLLFLAAICYPLYHTYCLLRNIQIARSTGLPYLILPFNQYSLPWIVLGRALYPLLATLLPFFPRLRWIRPDWFWARKY

AMYREVGADVFAMVSASGVFIVVCDADVVVDVLARKDDFPKPPVVHPGLDIYGRNVVTTSGAEWRRHRKVVTPHFGESYN

RQVVEIRAHQAVELVKHWSTRLPHETDGPVLRNVENDFIRLTINITTHNMFGTDLRWPNRIAPSSQTDDGYDEDGPPNDE

VLAPNHKMSYQASIKHMLNNCNMLMVAPSWYLKWTPNATHRKAGVACRELGVYLNEMVDEKRREMSAGTARGGDLITALI

RSEQGGEKGLGADKGLTEQEVIGETFMLVFTGHATTANALHNTITMLAIYPEYQIKLQEDLDRILGDRVPDYENDYPALA

ESWAGAIFHEINRLYPALPLVPKFTPSPQYITVNGTKHLIPAHTPVLLDCCNTQRNPKYWVPPHETEAVAAPDEFRPQRW

LNAPGESQNTNNPNGNFLKPYKGSYYPWSDGQRVCLGKKYSVVELLAIYAEVFRGHSVELDVGAGETWEEARGRAKRYME

RVDSGLLLKTFGKEPGVRYVKRGEERFFPRR*

>CYP5093H4|1139554|Tercla1

MSAHHQNFVNLITQIRTVGSQVSTMESTITEPAPQLGTFSSAIQSRLPMASLNTDLAFEPYPYGLVYALCTNEPDATPSP

FSPILGLSILLLMFLVAMDYQRSLKMPPGPLPIPIFGNKWALPLRKPWYKLKQWTDEYGSIVTIWTGRRPTIVIGDPKVA

CDLLERRSAKYSSRPRFVILGELFTNNDALLTMPYGERWRATKKIFHLGLCKNACDSYKPIQESESRRLARDLLMDPSRF

GKHLERYTASVMFCVAYGRRVDTLNDPVIEKIYERMSYMATLNVPGAFWAESYPILKLIPDCIAPWKREVKRRGAESTKM

LLDLANAVKLQTEAPESFTKTLWKKHIEQPDSMTEREIAYATGSLFGAGSDTTASTLQSFVLAMTCFPHTAKKAQEELDR

VLPKGRSPTWGDMPNLPYCNAVIKETLRWRPVAVMGGTPHATTQDDYYNGLFIPKGTTVMGNLWAIHHNEKYFNDSHNFI

PERYLGEAQSGLEIYPSRDGHSAFGWGRRICPGKLLAENSLFISITRILWAFNISKAKDESGVEVTPNIFDYTDGFNSKP

QPFFCDIKVRNPAIEEIINSEAQEGERFLGRYLYDGK*

>CYP548BD4|1161452|Tercla1

MGWLLGPSTIILPIGWGHEIPQLLFFTIGALLTTLVLYRLYFHPLAEYPGPLLARCTSLHAAYHAWIGDSHLVLYHAHRR

YGKFVRFTPNCVSVNDAVAVNEIYGHGRNVQKSSFYSVMPPYPHAFDTHSVIDKALHGRKRRVMAQAFLDTAIKGMEDNV

LTHIRTFVRNIGTDSCENENPGIGESNASRNMAYWSIWLTFDVIGDLCFGKTFGMLTREEWRFWPTLIDMAIHRHAIAGV

SLKMHKLGLGKYLFPAIAEGQKTFVKYSQEQAKERIALGAGVRKDFFHYLLNAKDPQSGEGYSMSELWSESNLLIAAGSH

TPSTTLAATFFYLANYPKVLAELTTLVRTTFSDCEEISSEKFSTPECQPAYKLVRACLDEAMRMAPPIPTTLTRQVLKGG
ITVAGKYFPEGVDIATPSFTLHMNEDYYSDPFTYNPNRFLADKADSPEAFQRARSAFAPFSVGPRNCIGRSLAYLELTLT
LARTVWMYDFVYVGGGRETRLDNIAFVEKLGGSKNPIMYNIDDHFTAFASREGPNIKFIRPGNL*

>CYP51F1|1174909|Tercla1

MDQKRFMKFGLSSETFRAYVPLIVEEVETYLKNSPLFPARKGPVNLLATIPELTLFTASRTLQGKEIRKGFTGEVAGLYH
DLDTSFTPMNFMFPRFPFPQNKPAL*

>CYP61A1|1175039|Tercla1

MASQSSPPAVPGDSSPEMLGGNMVKQLFGGVSYWSLAVTLFILCVTYDQAKYQWNKWGNIVGPAFKLPFMGPFLESIDPK
FEEYAAKWASGPLSCVSVFHKFVVIASTRDLARKVLNSPMYVKPCVVDIAKKILRPTNWVFLDGKAHVDYRKGLNGLFTR
KALSEYLPGQEDMHKRYFKRWIQISKKTPNVKFMPEFREINCAVSCRTFVGNYSSDEKVKKISDDYYKVTAALDLVNFPI
IIPYTRTWYGKKCADMVLEEFSECAQKSKDHITAGGVPHCLLDYWVKSMFEYKAHEERLARGETLAPGEKAPASIRMFTN
WEIAQTIFTFLFASQDASSSATTWLFQIMADRPDCLEKVREEALRVRGGDPYKPADIDMLDSMVYTRAVVKECLRYRPPV
TMVPYEVKKSFPVAEGYIVPKGSMIVPTLYPALHDPEVYVDPETFNPDRWLEGGEAEAAVKNWLVFGTGPHYCLGQTYAT
YNFMAMISLASLMLEWEHHVTPTSEKIKVFATIFPMDDCFLTFKERLPILD*

>CYP617V4|1177909|Tercla1

MHTKGYIFQKAPIVRRSIGKMLGMHGMLFSEGEQHKVQRRHMLPAFSYRHLRDLIPNFWEKSIKLNKVIENELRTRQDAD
TERQIGVVVEISQWLNRATLDIIGSAGFGYEFNCVNNDDESNELVQAYRKVFQSVSKGVQLQRVVFQYLPDWLIKLIPSK
RRKESAEAIEMVKGMCMRMVREKKREYETGGKAGQKDILSLVTKDSPFTEEEMRDQLMTFLAAGHETTASALTWALYLLS
QNPEIQSRLRAEIRSQISSPTSTTTPAVTLDTLPYLRNVTNEVFRFFPPVPMTLRMAGEDTTLGGHYIPKGTIVMISPWA
INRSKKVWGAKADVFDPDRWEAHKEGKEGASNYDFMTFLHGPRSCIGKDFSRLEFKALLAALVGKFEFNEVLDENGERKE
LVIKGGITSRPENGLPLWVRSLDGW*

>CYP51F1|1202758|Tercla1

MGVLSEFVIQLNPVTQTLAQQSTPVLVFGGIFAFCVITTVLNIIQQLLFADSNKPPVVFHIFPFFGSTIIYGIDPYKFFF
ACQEKYGDTFTFILLGRKMTVYLGPKGNDFIFNGKLSQVCAEEAYTHLTTPVFGTDVVYDCPNHKLMDQKRFMKFGLTSE
TFRAYVPLIVEEVETYLKNSPLFSARKGPVDLLAAIPELTIFTASRTLQGKEIRKGFTGEVAGLYHDLDAGFTPMNFMFP
WFPFPQNKRRDAAQRKMAQTYMDIITKRRAAAARGEQPEEKDMIWNLMNQKYKDGTVVPDKEIAHMMIALLMAGQHTSMA
TMTWILLHLASRPDVVQDLYEEQQRIFGKDLEPLTYETLGELTLHNHVIKETLRIHPPLHSIMRKVKSPMRVPNTNWVIP
DNHYLMAAPGVSAIDGKYFKNPTAYDPYRWAGEKVTTEEEEEKFDFGYGLVSKGTASPYLPFGAGRHRCIGEQFANVQLG
TLVAMFVRFLKMNLPDGQKEPAPPDYTTLIAMPQHPATMIYEWRKPEKK*

>CYP6685A1|1210502|Tercla1

MTLQEISYHSALLSHPSAQLILVLCAVYLVLEGISTIRLWLQRCRLVREHGCQPVAYSLGSWPLGLNAILEEKRANEEND
LPTLMDERFNKYGHTLGVSGLRHMKYITCEPRIVQAVLSTHFENFPKRPLINAASTFLGDHSIFMVDGPKWEHARAMLKP
QFVRDQVANFTDLEKHVERFLARAYPFPLTNAEIRESNPVAITTDIQPLIFDLLFDSATEDLLGESADIQLRIDAGLADD
VIAFNNAVDLAAHVTSIQVGFGKLYSWLYLGWEMNIKYSQACKTNFEYVRPYVEKALRRHSTKVKAAHTLANGAKETSKA
KKWVALNELAAADENLTQSKAYKDALASHAIGLLVAGRDTTSAMLSWAIHLLARNPQVYSKLRQEILKAFNNDNGSTPLP
DYPTLRALPYVRWVLKETLRLYPVVTVNERIATKDTVLPIGGGPDGQAPWLLKAGEGIAWSLHSMHRRKDLYGDDAAEFR
PERWGENEKGQGLRTIGWGYLPFHGGPRICMGQQRALNEGSYILARIAQTFSAIQVSDDIDIEKDRPQFKMTITTTSANG
VWVKLTKA*

>CYP567R1|1250376|Tercla1

MTDPLHLSHFWSFFPWERSVLDVNNPSGVKLLVLPALISFVTGWALWRVVYNLNFHPLAKFPGPWLAATSTLWFVHDSLS
GRSPFNLAEAHKKYGPIMRVAPNQLSVADAGAWKEIHGHHPREKTFIKGDFYNEDVKNFGLRNIFSTKDAVKHAEMRRML
AHAFSVKALTEQESIIQGYVDLLIKRLGERYAEKDRGPDGEYCNIVTWYNYTTFDIIGDLAFGESTAFACLKEQKAHPWV

TIMLDSIYLNALNEAFRRILGTRKLGQWLTPKSLKENRRRMDVYARDLVANRLGSQTNRKDFWHYILNRPESKDASVNEL
CMQAQTLVMAGSETTSTTISSITFYLLKTPRVYNILKDEIRSTFKSYADITEQNTVRMRYLNAVIEEGLRLYPAAPGGMP
RVSPGATVAGQYIPRGTDIAVHLFTASRDPAYWSMPNEMYPERWIDPKNTDNKEASQPFLLGPRGCLGKNLAYMEIRCIL
AKVIYAYNLELVDKDLDWEKKSMAWSLWWKPDLHVKVKKRPGLRWTTDEIPL*

>CYP548BE4|1250917|Tercla1

MSQQGPSSFWGVFKEGLGIISNCGGCIQITFLDDHDTSGIITSNPRSLVGGFTYLTSIAFISLLTLSLYRVLLHPLSQYP
GPILCKLTPLVATWHAYKGDRHLFLHRLHKKYGPIVRWAPNAVSIDSACALKEIYGHGINARNVQKADFYEAFPAVKGVH
NTHNCINKAEHARKRRVLSQAFSENALKGLESLVLKNVQVFFEVVEERMKGKAGKEKMVGKGGKEGLDMGEMFTWLTYDI
LGELCFGKAFGMLVDEGQRFVAGLIDNATHNHHICGHYLPLSTLNLGRLLFPTIAAERWRFILHSRACTNDRMALHASGL
DKDKRDFFHFLLSAVDPETGKGFETVELWGESNVLIIAGSDTTSSALAATLYYLVQNPDKLEIVKEEVRNKFNSVEEIVS
GKELNECVYLRACIDEAMRLCPPVPGLLPREVINPKGMTIDCTKTGGGKYSLPKGTTIGVNIYTMHHSPVHYPQPYKFHP
ERWLLPTDHPLGTATLESLVLAREAYTPFSIGSRGCIGKALALMELRLVMGRMMWEWEVEGVWGVEGKGEKWNGGYRWEG
LDEKGQEEGWEGEFRIFDHFTTRKEGPVVRFEKRVLT*

>CYP52AY1|1255740|Tercla1

MLSVTTLTIGGIFLAFLIRKLIYQYQLRKFARSRGCLPPTPISRWPLGIDTFRESMHNFKNMRFCKMNHGRHVTYGDTFK
LNVLGVVNICTRHPENVKAILTTQFQEFILAHRIGGKLDLLLGNHGIFVQWGPAWAHSRALLRPQFNKGQIVNDMDSLEF
HVKRMTSLLPDNEVFDIQPYFLALTMDTATEFLFGESVESLLTEELGVPAKGTFSEAFNTASWLISKKVRSGRWHWAVAN
KETARGCEISRNFVGKFVDKALAMDPEKKKEGKQKYVFLNALAEEYKDRTILTDQIINILLAGRDTTAGLLAFVMWFLAR
NKRVWHKLRAEVLERVGRENKPTWEIMKDMKYLRYVLNETLRLLPVVPINTRTAAKRTTLPHGGGPDQQSPIVIEKNHLI
FYSVYSMHRRPEVYGDDAEEFNPDRWETLKLGAWDYLPFNGGPRICLGQQYALNEASYAIIRVLQKYQDLVGIDPDTGKE
VPVFDLVPIRDGETIHSTTPAMTHILRYYAG*

>CYP539A45|1267081|Tercla1

MLLDSLTQLPASTILLLGVGNLVAWVGVSKAYESYRLRKLGKRPPAVPTWTPLLGLDIIQDSMKNARNNTTYDGWKDKSE
RVGYTFEMTLLLNRFVFTVEPENLKALLTSQFNDFGKGEEFHKNWFPFLGDSIFATDGEKWHNSRQLIRPQFIKDRVSDL
HTFETHIQKMISLVREAQGQTIDVQDLFFRLTLDTATDFLLGSSVNSLDSPQVEFAEAFAIVQKTHNDMERLGALRHFVR
FPEYKKSLEILNRFVNQFVHQVVRMTPEELESKGSHNYNFLHALAGFTRDPKTLRDQLVAVLLAGRDTTAGTLSWAFYEL
SRKPEAVQRLRQEILETVGPTEPPTYENLKNMKYLQHVINETLRLYPAVPFNVRTALKDTTFPVGGGPDRKQPIAILKGT
QVAYGPIIMQRRKDLFGPDADEFKPERWNNWTPKAWQYIPFNGGPRICIGQQFALAEMGYCLVRLFQSFEEVNYMGTKPP
RMRCEITISAVGGVEVAFREASKA*

>CYP6001H2|1273680|Tercla1

MSLEIPLDVLNHLGSEVPEDILHQGAPQPPPASHFRLPDGSLGNIRNEKLGHAGCPFARIIHTEAEIHGAKPEPEKLIDG
LMPQGHNFYASGVSQLTFYYTSLIAYDILYQNHQDMSINDTSSYLDLSPLYGRNQAEQNSVRAFKNGLLKPDIYADPRPS
ISPGVAVLLVLFNRFHNFAAQTVLAINEKGKFTPPADKPKREQDKWLDEQVFQTARAITVGLYVNIILQDYLRALTNTPI
KDTEGKDPRVTLHDFFDKQGLPKGAGNQVSVELNLLYRLYFAIPRTNTSWLQESYRKIFPQKDPAGLSDLELLKALDDHA
QAHPIDPSQRTFGALKRGKDGVLLEADLIEVWLEIVEQGASPFGVNVSPDVYKPLAKTAIEQARKWHAPTLNEFRKFLKL
APFKKFEEVHSDPDVATNLKNLYGDIDYVELYPGLYFEEQKEDSFGSSTAVRSMLVSSLSLVRGDRFLTHDFTAANLTAW
GIKEIAPDYTILGGAKIFLLVLNGFGRYIAFNSVYAMLPFFTPTRSKEIVEKLEIAHLFSFTRPAGQSPQPVPILTHAGV
TTVLGDQKNFRVPWAPHMTSLSTFMLASDKPESAKQKRDLGEAIYGQKDALKDFEAFAMKLTHGFLTKKSHPIGHGTGLR
NVIDIAKDVADLVATHFVAKLVYLPLNCSTGPSYTEVELTKLLDDVFIYIFADSDPTRSWARRRDGDNPCAKLVDAMVER
VKHVQDTGAADSESKSECPVSSSAGASGDEPLANYGIMLVKRLLATHRSVREVAEILSGIAVGFHANASMLFTQMVDFYL
NEAKYWEDIKALAAKPGADSDAKLQGYVLEGYRLSLTLALLRYTAPGVNTEIEGRKIKGGDVIFINLMTASRDPSAFPNP
TEVNLTRPIDSYVHYGYGPHKCFGKPLNMSYARGMLKVLAQLPGLERAPGDDGVLHYTTSLSGLKNYLSADWSELSPLPS

SKLIS*

>CYP573C2|829836|Tercla1

MLSLPNHHHGSTLPPRIQVININGEHIGEIISDARSLIRAHLGLIILGLMYFGLVSHVVKNRYFHPLSKFPGPFWGSITN

LYCSLMILTGESQKMEFDFHKKYGPVVRIAPNLLIVSDPRMLPVIYQRKADKTDFYITGTLGKGAESVFNTRAHHDHYHL

RKRLAGAYHLRATKGMETLVGERILEWTAKMGRTFADTGHILDLSMWSQYLAYDVITELGFGEAMGFVRQGEDVGGLIKC

FHYALPAIGSLARLPILTKMIVGWDWVTAKPTDNYGLGPVMAFRDRCIEQRQAAGSNSRQDLLQNLLDQRTADGQPAMSV

DQIKAETLIVLLAGSDTPASAFRACVLYTLSTPGVYEKLMEEINTAVDQNALSSPVVTFDEAKKLRYFSACLREAMRLSP

SAPVLLPRLVPEGGNMLCGQFVPEGAEVAANPWVIHRSKELYGEDAEEYRPERWLESEEKTKEMDKYDFQFGYGDRSCLG

KSIALMELYKGLVQFFRLFSPELVNPKRPCVYRNMGVAVHTDFWVRVKNRGIDST*

>CYP6001C33|1141080|Tercla1

MDKENDLQIQPANITSNGNSHDHSNDHSNGHPTGASNGKAKATPVPLGGRDSNWKYKGAKERPTRKEMEQTMSALTNLLH

ASNKPLPNRYGDGKERKSIYDEKYEGVWGDIKALAKQGKLSESYKTISTVAKHKKHGGYTVDKTYITFVSSSSKLHGPSS

IDGSASRSVMEYTPASPLSLLGNAHKFRQPDGAYNNPMHPNLGRAGTAYSRTVKPMTLTPMSLPDAGLVFDSVMSRREYR

PHPNNVSSMLFYVASIIIHDVFRTNRTDISINDTSSYLDLSPLYGNNLEDQKKMRTFKGGKLKPDCYNEKRILAFPPGVS

ILLVMFNRFHNHAAEQLAGINEGGRFDLKINHRDPDREGAARRAEEAREEELFQTARLVTCGLYINMILSDYLRTIVNLN

RTDSTWTLDPRFEPSKIYNPNATEMGTGNCCSIEFNLVYRWHSAISKRDEIWTEELYKEIFGEHVNALDMPLMDFLKGLG

HWEASLPEDPAKRTFSRLQRNPQTGMFEDDDLVQILTDSIEDPAGAFGARNVPAILRAVEIMGIEQGRKWRVSSLNEFRS

FFGLEPHKTFESINRDPEVADALRQLYDQPDFVELYPGLVAEEDKVPMVPGVGISPTFTISRAILSDAVCLVRGDRFYTL

DYTPANLTNWGFQEVASDLNVLNGCVFYKLFLKCFPNHFTWNSIYAMYPLTIPSENEKIMKDLGKYDLFDYSPRNKQTYN

VMWDGFTWIYGKEFMLSGDTDWHEGMRQFVGECLFGQQAWEREVKQFYEDTCTRLLKDKRYRIPSPGGKGPGCWQIDAVR

DVNNLAQANFAATLWGLPLKTKDNPKDFFTEQEMQQILTLMFVCIFFDVDPSKSFPLRYAVQQLVRQFGKLVEFEVKAVH

SPIGAMLSGRYFKKRNTSALQSYGLKMIQRLLEGGSSPEDVAWSYVLPAAGASNANQGQVFAQVLDFYLRPENTLHLAEI

QRLAKLDTPDADELIKRYALEGTRLAGTFGLYRKVNCGDGETITLEDGQRTVNLQKGDRVFVSFISASRDSSVFPDPEMV

KLDRPMESYLQYGTGPHKCLGANINMVSLPTMLKFFGKLKGLRRAPGIAGELKAVEKEGGFKVYMREDWSGYWPFPVNMK

IRFDGVEGWEGE*

Tirmania nivea G3 v1.0

>CYP61A1|529996|Tirniv1

MASQISPSAVPGDSSPKVLGGTMVEQLLGGVSCWNIAVTLLVLCVTYDQARYQWNKWGNIVGPAFKLPFMGPFLESINPK

FEEYAAKWASGPLSCVSVFHKFVVIASTRDLARKVLNSPMYVKPCVVDVAKKILRPTNWVFLDGKAHVDYRKGLNGLFTR

KALSEYLPGQEDMHKRYFKRWIQISKKTPNVQFMPEFREINCAISCRTFVGNYSSDEKVKEISNDYYKVTAALDLVNFPI

IIPYTRTWYGKRCADMVLDEFSKCAQKSKDHIADGGVPHCLLDHWVKAMFEYKAHEERLARGEILAPGEKAPTSIRMFTN

WEIAQTLFTFLFASQDASSSATTWLFQIMADRPDCLERVREEALHVRGGDPYKPADIDMLDQMVYTRAVVKECLRYRPPV

TMVPYEVKKNFPVAEGYVVPKGSMIVPTLYPALHDPEVYIDPETFNPDRWLEGGEAEAAVKNWLVFGTGPHYCLGQTYAI

YNFMAMISLASLMLEWEHHVTPKSEEIKVFATIFPMDDCYLTFKERLPILE*

>CYP548BE6|532052|Tirniv1

MDHFTSSLPLLTSTHIGNFLTSPLNFPLTSTLLSGFTYLTSIIFISLLTLSIYRILLHPLSQYPGPILCKLTPLVATWHA

YRGDRHLFLHRLHKRYGPIVRWAPNAVSIDSAAALKEVYGHGINARNVQKADFYEAFPAVKGVHNTHNCINKSEHARKRR

VLSQAFSENALKGLEGLVLKNVQVFFEAVEERMAKGKVGKEKMVGTGGKEGVDMGEMFTWFTYDVLGELCFGKAFGMLVN

EGQRFVAGLIDNATHNHHICGHYLPLATMNLGRLLFPTIATERWRFILHSRACANDRMALHTSGLDKDKRDFFHYLLDAV

DPETGKKFETVELWGEANVLMIAGSDTTSSALAATLYYLVQNPDKLELVKEEVRNKFNSVEEIVSGKELSECVYLRACID

EAMRLCPPVPGLLPREVINPKGMTIDCTKTGGGKYFLHKGTTLGVNIYTMHHSPVHYPQPFKFHPERWLLPTDHPSGTAT

KDSLELARAAYTPFSIGSRGCIGKALALMELRLVLGRMMWEWEVEGEGWEGEFKIFDHFTTRKEGPVVRFERRNLAE*

>CYP539A48|532383|Tirniv1

MFLDSLTQFPASTILLLGVGNLVAWVGVSKAYECYKLSKLGKRPPTIPTWTPLLGLDIIRNSMKNAKNYTTFDGWKEKSE

RVGYTFEMTLLLNRCMFTAEPENMKALLTSQFNDFGKGEEFHKNWFPFLGDSIFATDGDKWHSSRQLIRPQFIKDRVSDL

HTFEIHVQKMISLIRLAQGQTIDVQALFFRLTLDVTTHFLLGSSVNSLDSPQVEFAEAFAVVQKAHNDMERLGPLRYFVR

FPGYKKSLEILNRFVNQFVDQVVRMTPEELESKGSHNYNFLHALAGFTKDPKILRDQLVAVLLAGRDTTACTLSWALYEL

SQKPEAVQQLRQEILETLGPKEPPTYENLKNMKYLQHVMNETLRLYPAVPFNLRTALKDTTIPVGGGPDRKQPISILKGT

HIAYSPIIMQRRKDLFGPDADEFRPERWNNWTPKAWQYIPFNGGPRICIGQQFALTEMGYCLVRLFQSFEEVNYMDTKPQ

RMRCEITISAAEGVKVAFKEAVKA*

>CYP6685A2|538867|Tirniv1

MALLSHQSEFLLLVFSAIYLVLKSISTIRLWLQRRNLVREHGCQPVAHNVGSWPLGLDAILEEKRANEANDLPTLMDERF

NKYGNTLGMNGLRRMKYFTCEPKIVQTVLSTNFENFPKRPLIKAASTFLGDHSIFMLDGPEWEHARAMLKPQFVRDQIAD

FTDLEKHVERFLARAYPIVDAGVKGESKTVAVTTDIQPLIFDLVFDSATENLLGESADTQLRKDAGLANDAIVFSNAIDL

AAHVVAIKIGLGKLSSWLKTNLKYNQACKTNFEYIRPYVEKALRRHSEKVQAAHSMASGDKETSKKAKKWVALNEFAAAD

EYRMQDKINKDALASHAMGILLAGRETTSAMLSWAIYLLARDPRAYSRLRQEIMVAFKDDNGTMRLPDYATLKALPYIRW

VLKETLRLYPVVTVNERMATNDIVLPIGGGPDGQSPLLVKAGEGIAWSLYSMHRRKDLYGDDAAEFRPERWGEDEKGQGL

RAIGWGYLPFHGGPRICMGQQRALNEGSYILARIAQTFSAIEVPDDVDIHKDRPHFKMAITTTSSNGVRVKLRKAA*

>CYP51041B1|551490|Tirniv1

MFARASGWYRAYHEIWRSGKFAEELQRLHKVYGPVVRIGPNDVHFSSPEAYEAIYNARSKVIKDPWLYKCFSEDESLFGY

TDPVAAKVRRHQLAPFFSRQNILSLQDMITEKVDLLCEKLNHSIVEGTSGAVVDVGSAIKSMAMDAVMSVCLGESLNTLE

DPKFRHPVIEFMEQSLPLLWVFKHIPIIRVMMLSIPDGVSARMGTHGILLLKKKLDAFLAGLEDLDATQNTRKHGFNTDF

GSKEIIFHRLLDDNNKPVAHYGSLFDEAQALFVAGSDTVGNTLSLSIFHILSNEEVYSNLCKELKEAWPSGDLKNISTRP

RWENFEKLPYLTAVIKEALRLSHGIVSPLSRIVPAQGLTVDGIFMPPATVISGSAPFVHLNERLFPDPRTFKPERWLSQE

SKGLEKYIVAFSRGPRSCIGINLAWCEMYLTLALLFRKFDLELFETSAKDIEYKDYFVPSFYGRIRATIKQRCD*

>CYP6001H3|71273|Tirniv1

MSLEIPLDVLRHLGGEAPEEHQGARATPRPPLVSHESRFRRPDGSLGNIRNEELGQAGCLFTRIPHTEAVAHEAKPEPEK

LLELLDALTRGRKCNNFYPTGVSQLVFYYTSLIAYDIFYQNCHDMNINDTSSYLDLSPLYGRNQEEQNSVRAFKNGLLKP

DTYADPRPSLPQGVAVLLVMFNRFHNFAAQTILAINEKGRFTPPADMPKGEQDKWLDEQLFQTARAITVGLYVNIILHDY

MRAIANRPINNTEGKDPRVTLHDIFDKLGLPKGAGNQASVELNLLYRLNLAIPRTDASWLQENYQEQFPQKDPAGLSVLE

LLKALDSYAQANPIDPTQRTFGALKRGKDGVLPEADLLEIWIEMVEKGTGPFGVNIAPDVYIPFAKLAIEQARKWHAPSL

NEFRKFLKLAPFKKFEEVHSDPDVATNLKNLYRDIDHVELYPGIYFEEHNEASPGSSTAVRSLLFSAMCLVRGDRFLTHD

YTAANLTAWGIKEIAPDYTILGGARIFLLVLNAFGSYIAFNSVYAMLPFFTPARSKEIAKKLGIADLFSFTRPAGATPPP

VPIVTYAGLKTVLGDQKNFWVPWAAHMTSLSTFMLASDTPEAAKQKRDVGEALYGQKDALKDFEAFAMKLTHELLAKKSH

PVGYGASVMNVVDIVKDVANLVATHFVAKLAYLPLNCSTGLSYTEDELTKLLDDVFIYIFADSDPTKSWARRRDGDNPCA

KLVDAMVERVQHVQDTEAADSGGTSGCPFSGSADASGDDPMANYGINFVKRLLATHRNVREVAEILAGLGVGFHANAAML

LTQMVDFYLTDANAKYWDDIKGLAAKPDANSDTKLQGYVLEGYRLSNTLALLRYTAPGVDMEIEGRKVKGGDVIFLSIIF

ASRDPSAFPDPNTVNITRPSDSYIHYGYGPHQCLGKSLNMVYARGMLKVLAQLPGLKRVPGDDGVLHYTTSRSGLKTYLS

ADWSELSPLPSTMKLMWQGRPKVYPMHTKEGYE*

>CYP584AN2|602901|Tirniv1

MLSVPTLAIGGVILAFLIRKLIHQYQLRKFALSRGCLPPTAISRWPLGLDTFRETMRGLNNLSFCKMNYERHLKYGHTYE

LNMLGKINISTCHPDNVKEILTTQFQNFNIAHRLGSKIDLLLGKHGIFTQMGSAWAHSRALLRPQFNKGQIVNDMDSLEF

HAMRMTSMLPDNKIFDIQPYFFALTMDTATEFLFGESLESLLTEKGVEPAKGTFSEAFNTAQWWISLKLKSGPMHWVIGN
KETARSCEISRNFISKYVDKALTMDMEKKKKEGKARYVFLDALAEEYKDRTVLTDQLINILLAGRDTTAGLLSFIMWFLA
RNKRVWHKLRAEVLENVGKENKPTWEVIKDMKYLKYVLNETLRLLPVVPLNTRSAVKRTTLPRGGGPDQQSPLVLEKGQL
VFYSVYSMQRRPEVYGDDAEEFNPERWETLKLGAWDYLPFNGGPRICLGQQYALIEASYVMIKVLQKYQDIVGIDPATGK
EVPGWGEDKTVKAFKERLGLTLSCMDGVHVKLTPAA*

>CYP548BD3|608766|Tirniv1

MGWLSSTSTINLPAGGHLDTSRLLLFAAGGLILHLTTVVLYRLYFHPLAEYPGPLLARCTTLYAAYHAWIGDSHLVLYHA
HRKYGKFVRFTPNFISVNNISALNEIYGHGRNVQKSSFYSVMPEYPHAFDTHTAIDKALHGRKRRVMAQAFSDAAMRGME
DNVLTHIRTFVRNLGADSCENEEESNASRNMAYWCSWLSFDVIGDLCFGKAFDMLTREEWRFWPTLIDMAIHRHAICGIS
LKIHKLGLGKYLFPTIAEGQKTFIKYSKEQAKERIALGAGERKDFFHYLLDAKDPQTGEGYSMSELWSESNLLIAAGSHT
PSIALGATFFFLVNYPKVLAELTTLVRTTFPEFEEINGEKFAKPECQPAYKLVRACLDEAMRMAPPIPTTLPRQVLKGGI
TVAGKYFPEGVDIATPCFTLHMNEEYFPDPFTYNPNRFLADKAESPEAFQRAQSAFSPFSLGPRGCIGRRLAYLELTLAL
ARTVWTYNLVYIGGGREIRLDNIAFVEELGGGKSPIIYSIDDHYTAFASKDGPNIRFTRQKDF*

>CYP5242B2|609588|Tirniv1

MITMAVFDLSSTALENTLQLSPRLLYTLVLLLVPGSLILLSRIIGNAAADFKTKDGRKMVVLKQDTRVSRFTNGFALSQE
GKRLAGGDPYLVRNGANLEVVIHTPEHVRDFFAKDGKDHLKPRNGDFGAYFGRMLGNCVGMLNGKLWKTAKEHLSPHFAH
GRTMTAVPIFRAEYLKWIEQLPEDTMISYKRSDGSGFVIDALSACRKLPFKMIAMVLYRDMLTDKLFDELWELNEVHERI
LFAALLKTLPAKTWYSLLPTEDNKLLEAYCKDWERINMAVINETRMTGTYCPVTEMYKGVENGDITLEQFLQSLDEILFT
NIDVTSTIFAYILINLGRDREFQSALRQEILSYSHSLDAYITDDESLLHFAYLEALRINPAAWFSIPETTGPDPKYISSF
LIPPHTSVIIDLKTLNTQSPIWGSDGHVFRPLRWRGLSPSAVRYSFHRYGMGPRKCMGKNVANIIIKMLMVTLLEKYEVV
ADAEGVENDMKYRTDRFTCTPEKEVEFRALSGKGGEQLVA*

>CYP6453A2|612280|Tirniv1

MTALAGLFGETTSENFWPYYYLTSVIFHVSFSLLVRGILLPLGLLYPICVVIYRLYFHPLAGFPGPKLAAATSWYEFYWN
AVKDGQAAHKRKEWHEKYGPVVRINPNEIHITTPTAYNEIYLKTKPAFNKYYPFYRMFLADTASIGIMDNHAHRARRELL
TPLFCRQNVIDMEHVIWRSVYSLCDKLQAYTPQSGQPQAGRKIGTKVLFKMLTSDIISGFCYGKSFESVERVEIEATKAG
IDKAIPPRFIRAMAGAAQGFWFMQRFWRLQLFLVNLPDWVVDTFHMEASLGMRDLITECMAHIDDDSLLTDGAKRKNLFS
LLLSPSTSQYDYQGDKSKAYKQPTRKELLEEAITVMGGGVEEASNAMMYGIHYMLITDGISEEVRKELRRIWPDKNSPIT
FLELERSSYFMGCIKEIIRMSIPVPGKLPRVVPPGGFDADGWHIPSGSIISMSANMQNFHPEIWYEPNKFIPERWLEEDG
TPRRDMDRNLCSFSKGNRMCLGSNLATAELYITLATLFRRFNLEVVKEDTDMRWADRVAAQSVGELVLLVREES*

>CYP617V3|612799|Tirniv1

MHFLHNLGLTTALMTYLLHTHLPPYTSFLPSCFTSSVVCTFVYLFSASLFAKLTYRILIYPHFLSPLRHIPGPKSVSFWN
GQFPIIQRLPSGQPHIKWIDGVPNDGLIRYLGFCNVDRVFPTNPKVLQEVMHTKCYIFQKAPMVRRSIGKMLGMHGMLFS
EGEQHKVQRRHMLPAFSYRHLRDLIPNFWGKSIVLNRAIENVLRTKQGADTERQTGVNVEISQWLNRATLDIIGSAGFGY
EFNCVDNDDERNELVQAYRKVFQPVSKTVQLQRVIFQYLPEWLIKLIPSKRRKESAEAIEMLKGMCMRMVREKKREYETG
GKVGQKDILSLVTKDSPFTEEEMRDQLMTFLAAGHETTASALTWALYLLSQNLEVQSRLRAEIRSEIPSPTSNATPAIPL
EEYLDTLPYLRNVTNEVFRFFPPVPMTLRIASEDTTLGGHYIPKGTIVMISPWAINRSKKVWGPGADVFDPDRWEVHKEG
KEGASNYDFMTFLHGPRSCIGKDFSRLEFKALLAALVGKFEFNEVLDENGKRKEIVIKGGITSRPENGLPLLVRSLDGW*

>CYP51F1|625879|Tirniv1

MGVLSELVAQLDPVTHTLAQQSTPVLVFGGIFAFCLVATVLNIIQQLLFPDPNKPPVVFHIFPFFGSTITYGIDPYKFFF
ACQEKYGDIFTFILLGRKMTVYLGPKGNDFIFNGKLSQVCAEEAYTHLTTPVFGTDVVYDCPNHKLMDQKRFMKFGLTSE
TFRAYVPLIVEEVEAYVKNSPLFTASKGPVDLLAIIPELTIFTASRTLQGKEIRKGFTGEVANLYHDLDMGFTPMNFMFP
WFPFPQNKRRDAAQRKMAQTYMDIITKRRAAATRGEQPEEKDMIWNLMNQKYKDGTVVPDKEIAHMMIALLMAGQHTSMA

TMTWILLHLASRPDVVQDLYEEQQRVFGKDLGPLTYESLSELTLHNHVIKETLRIHPPLHSIMRKVKSPMRVPNTHWVIP
DDHYLMAAPGVSAIDGKYFKNPTAYDPYRWAGEKETPEEEEEKFDFGYGLVSKGTASPYLPFGAGRHRCIGEQFANVQLG
TLVAMFVRLLKITLPDGQKEPAPPDYTSLIAMPQHPATMIYEWRELGKK*

>CYP6900A1|131549|Tirniv1

MALITFMSGILAHSLPSFSTFVLILITALPAYHLVILPIYYSAKSKLSTLPGPTLTLLTPYYITLLDALRLRTTSLYKWH
LKYGPVVRVGPNEVSFTSATAIKQIYQDPTMEKDSRLYGLFTHFGAKNAFSSKTRSEHGWRRKGVAATLSRSKVLESERT
DMWIGIVMGRYLKTIEENGLASKLVNEGNVRRYLVDVYLLSNWFAADVVTGFIFGCKRGTKTLQSDTNLHGQDEEQWWQK
SSYHRRIINEHYAPTFRAHTYLYVEFPRLMRLVDKIINAKETVLKKLSALAEGTSDIGGWQPEVDGSKIDDWGWRTWMAV
RADTRRCGINDTCVAEVLAGLVDGAGDRSTISEFDNDKDHRASWSDEIAASELMDLILAGMDTTSDTLSFLLYQLSLPSS
GLIQSKLHHELVTAFPPSAWNQLPPFFANPHWGNLEVPPNYSYFLQAIDTLPYLDAVIKETLRLYTAIPGTLPRLVPEGT
GRGKIIDGLYMPPGTVVGSFAYGIHRDTSVFGNEIFEPQRWIIIGSPEAKERQGNDDEEDWNEGTDKHAARGYSTATASI
HKNSVQGANDETIRIKTMEKRLWAFGSGGRGCVGRHLAILEMKILVATIFWRYQTEIVTDKDARMVGKLDHYHWDHRKAF
RDILPFKGVNGVLAFIPYTYDT*

>CYP6433A2|668888|Tirniv1

MEHWLKIPENVIRHRIFRFRETFLYSVEAPVGRGAYWLRSWQTAIEWVANAKSIIDRGYYEHRDKTGAFQVPTLSENHVL
ICSDQLVEEFRNAREDQLSFTQRVADVLQLEFTIGPGLFFNGWHIPFIRNQLTKGLEGFTIPLMEEFKLAIEDHMMSNSG
QWTSVKAYGFCSTVIARGMSRTFVGLELCRNKEYLKTTLEFTQQLFIAANLLRFIPTPVKYLISPLINERFLNKPLRISK
RLLAPIIEERKRRAQEEGEDWKGRPDDMLQWLIEGAPESEQSTDALVLRMTEVNMSALHTTGATLYECLFRLAMHPEYIP
DLRQEINYTIVHNGWTKVAMAKLVKLDSFMRETHRFSGTTLVSMGRKVIGKGFTFSNALHLPAGVTVSIPETPHMDEREY
ENAHVFDGFRFSRPFERGEVAQGIDNKGSKRKYFTTASTDFLRFGYGHSVCPGRFFAAHKIKIILCLMVMYYDIRLEDKP
RPVYFEFVRGSDLNACLSFKRRQSEIS*

>CYP5192F1|707137|Tirniv1

MTVLQLQPFILAFAMILGAWLVKFIKEGYQWRKKFQDTSLDLPCPPHHWILGHLPVMVKLFQKIPRDAHPHIYGAVIGPE
YKLPKFYYMDNWPYGPSNLVIIDPEIAQQVTVLTSYDKHPELMRFTKPLAGDNNLVSVNGETWKKWRRIFNPGFAIGHLM
GLVPEIVEDTEAFVKALGKFADSGAVFRLEDTATRFTIDVIGRVTMDVQFHAQETDHPLIKAFRSQVRWLPRDLTPNPFY
SLNPIRRFMMRRNIKIMRGFLDAELEKRLATFDFTLDPNSPESRKKKSVIDLALQEYLKEARSEGRVITKLDKEFKENAI
TQALIFIFAGHDTTSSTLCYIYHLLSKHPEALAKIRAEHNKVLGPDPSAAADIIRNDPHIISRLHYTLAVIKETLRMWPA
ASTVRSGAPGKNITDPETGKTYPTDGFLVWPIVFGIHRNAKIWPRPAEFIPERFMPDPPEELVPLPNAWRAFEKGPRNCI
GQELALIESRIAVALTIRTFQFEVQYDDESWRDKVVEFDGKPEVPGTFEEGLKPEQGKIDNFMGDKAYQVLLGTAKPKEG
MPVVVRRVKVY*

>CYP50042A2|204653|Tirniv1

MAILTSSILSRLSPAELAGILIIILCALKVTFSLYRHDPNPSKLPVVGIPPGLFGRIRAIPACLTQYNSIIADGYLRYHKQ
KNPTAFVAPWVLSNYIHIIPPHLIPEHKSAPENVLSQVAAFQGENGNQAIGGREIFTHMYHVPIIRQKLTASLAKMLDPM
VEELEMSFKEEWEQPWMQTKDAEGWVEMRIFEETMKIVSRTSNRVFGGVVICRDKAFLKNAIDFAVGVVMAGYGVQLAPK
KMRWLVEPLVSIRNRRRLAFAARKLTPIIEERRAIIERNSRDESNTLEEPDDLLQYLIHEAINLGPPHDSVYQLACRYLV
VNFVAIHTTSLGFSNNVANMAAYTDPTTGRSYWDILREEVEAVDRVSEEGPGVWTKRKLNKLVGLDSFIRETLRKNISGA
VGMVRKVMPKEGHTYSNGLHVNHGQLIGVPTLSMHNDDDSTGQQALDFIGFRYSRPYQELSAEAAADTSATGGVGKLAAV
TTADHYLSFGHGKHACPGRFFGVIEIKMLLNYCLLNYEIQSTERAPPQFVLANQAPPFNIVIKMRKR*

>CYP573C3|399601|Tirniv1

MLSLPNHHYGSTLPPRAQVIDINGEHIGEFISDVRLFIRAHLGLVILGFMYLALVSHVVKNRYFHPLSNFPGPFWGSITN
LYCSLMILTGESQKTEFDFHKKYGPVVRIAPNLLLVSDPKMLPVIYQRKADKTDFYITGTLGKGAESVFNTRAHHDHYRL
RKKLAGAYHLRATKGMETLVGERILEWTAKLGRTFADTGDMLDLSMWSQYLAYDVITELGFGEAMGFVRQGQDVGGLIKC

FHYALPPIGSLARLPNLTKMVVGWDWVTAKPTDKYGLGPVMAFRDRCIEQRLAAGSNSRQDLLQNLLDQRTTDGQHAMSV

DQIKAETLIVLLAGSDTPASAFRACVLYTLTTPGVYEKLMDEINTAVDQNELSSPVVTFDEAKRLRYFSACLREAMRLSP

SAPVLLPRLVPEGGTMLCGQYVPEGAEIAANPWVIHRSKELYGEDAEEYRPERWLESEEKTKEMDKYDFQFGYGDRSCLG

KSIALMELYKGLVQFFRLFEPELVNPKKPCVYRNMGIAVHTNFWIRVKNKGIDSA*

>CYP5093H6|405759|Tirniv1

MSTMESSITDSAPQLGTFGSAVQSRLPLASLNTEPALKPYPHGLAYAVCANEPTAPPSPFSPILGLSILFLMFLVTMDYQ

RSLKMPPGPLPIPILGNKWALPLKKPWYKLKQWTDEYGSIVTVWTGRRPTIVIGDPKVACDLLERRSAKYSSRPRFVVMG

ELFTNNDSLLTMPYGERWRATKKIFHLGLCKKACDSYKPIQESESRRLARDLLRDPSRFGKHLERYTASVMFCVAYGRRV

DTLNDPLVEKIYERMSYMATLNVYECLFSYQYHYTHFVIRPGAFWAESYPILKLVPDCLAPWKREVKRRGAESTKLLSDL

AKAVKHQIVKTEAPASFTKTLWEKHMEQPDSITEREIAYATGSLFGAGSDTTASTLQSFILAMTCFPHVAKKAQEELDHV

VPKGRSPTWEDMPNLPYCNAVIKETLRWRPVAVMGGTPHATTQDDYYNGLFIPKGTTVMGNLWAIHHNEKYFSDSHNFIP

ERYLDDTESGLKVYPNRDGHSAFGWGRRICPGKLLAENSLFISITRVLWAFNITKAKDESGVEITPNIFDYTDGFNSKPQ

PFLCDIQVRSLAIEEVINTEAQAGESFLSSYLYEPK*

>CYP6001C33|711752|Tirniv1

MDKETDSQIQPANNTSNGNSHDQSNGYSNGASNGKTKAAPVTLKGKDPNWKYNGAKEKPTRKEVEQTMGALTNLLHASNK

PLPHRYGDGKQRKSIYDDKYEGVWGDIKALIKQGHLKESYKTVSTVAKHKKHGGYTDDKTYIMEYVIRLSSHLPPNSMNR

LKLTAQQVDQLWGTLQHPPLSLLGNAYRFRQPDGAYNNPMHPNLGRAGSPYARTVKPMILMPMSLPDAGLVFDSVMSRRE

YRPHPNNVSSMLFYIATIIIHDVFRTNRTDITINDTSSYLDLSPLYGNNLEDQTNMRTLKDGKLKPDCYNEKRIVAFPPG

VSVILVMFNRFHNYVAEQLAGINEGGRFDLKINHRDPDQEGAKRKAEEAREEELFQTARLVTCGLYINMILTDYLRTIVN

LNRTDSTWTLDPRFEPSKIYNPNATEQGTGNCCSVEFNLVYRWHSAISKRDEIWTEELYREIFGERVDALDMPLMDFLKG

LAHWEAALPADPAKRTFSKLERNPQTGMFYDDNLVQILTESIEDPAGAFGARNVPAILRAVEIMGIEQSRKWRVSSLNEF

RAFFGLEPHKTFESINRNPEVVDALRQLYDQPDFVELYPGLVAEEDKVPMAPGVGIAPTFTISRAILSDAVCLVRGDRFY

TIDYTPANLTNWGFQEVASDFNVLHGCVFYKLFLKCFPNHFTWNSIYAMYPLTIPSENEKIMKDLGKYDLFDYSPPKFVK

PRIPILSYATTKRILEDKHTFNVMWDGFTWIYGKEFMLSGDTDWHEGMRQFVGECLFGQQAWEREVKQFYEDTCTRLLKD

KRYWVPGPGGQGTGCWQIDAVRDVNNLAQANFAATLWGLPLKTKDNPKDFFTEQEMQQILTLMFVCIFFDVDPSKSFPLR

YAVQQLVRQFGKLVEFEVKTVHSRIGAMFSGRYFKKRNTSALQSYGLKMIQRLLEGGNSPEDVAWSYVLPAAGASNANQG

QVFAQVLDFYLRPENAQHLAEIQRLAKLDTPEADESIKRYAMEGTRLAGTFGLYRKVNCDDSETITLQDGQRTVNLQNGD

RVFVSFISASRDPSVFPDPLVVNLDRPMESYIQYGTGPHKCLGTNINMVSLPTMLKFFGRLKGLRRAPGIAGELKAVEKE

GGFKVYLREDWSGYWPFPVNMKIRFDGVEGWEGE*

Trichophaea hybrida UTF0779 v1.0

>CYP53A60|48984|Trihyb1

MAFITYVFLGLLPIAYYLVPYLTDSKKLRRFSAPFPGAFSDFWLFWQARKGKRYFAVHNLHKKHGKFVRVQPKHVSIADP

AAIPIVYGHGTDFLKSDYYDAFVSIMRGLFNTRDRAEHTRKRKTISHVFSTKNVLQFEPYIHHNLELLASQFNKLSAQPD

LPGGFHKLDILHWTNYLAFDIISDLTFGSPFGMLVSGKDQAIVKDPNTGKITTAPAIQVLNRRGEVSGTLGCAPWAKKYA

KWFPDKFFTQGIKAVENLAGIAVARVSDRLDRGLGADREDLLKKLIDRRDENGNPLCRKEIEAEALTIMIAGSDTTSNTL

CSLMYWVLRTPGVLGKLQEELESALPGDWSVPNYASVKDLKYLRAVINETLRIHSTSSLGLPRVVPPQGATVCGEFFVGG

TVLSVPSYSIHHSDDIWGDAETFRPERWFELTDLQKKSFIPFSIGPRACVGQNVAELEMVTIVATIFSGWDWKFAEGEKQ

GLPGAPLNTAEGFLRKPLGLNVGVKRRNA*

>CYP51074B2|660881|Trihyb1

LPQKWQDYFNITTFGAHWRVKGRMAERYGGVYLTVSAARVACFVSDAEAANEIMIGRERFPKPVKSYETLTVFGTNIVAT
EGREWTHHRKHAVAPFGERNYKLVWKESLQQAADMAETWEAKSYQPDTPSNCMSLALHVISAAGFGIPMKFADTEERPVE
EVELFTDGHKPPKGFSFSFRQALQFITANMLQHALIIRLMPRWIPKEWFSYTHMHQQAHTDFSRYLREMVAKSHTSPGGT
SNLLDLLVAAEKTSEDAALSLSELMGNIFIFTVAGHETTAQSLHYAFLMMALHPEMQQWVCDGIDKALEGQSRDIQKWKY
EEVFPKLVTPLCLMLETLRLFPVVPYIPKSTGESGTTLTYGGRTVHLRPMYIAVSATSVQRSPAYYGPSADVFDPTRWDA
TNTNSFLSKNKGQQGLMAAGLEYPTIHKPERGAFFPFSDRARGCLGRKFAQVTFVAVIAVVFRDLKVRIKELEGETREMT
ENRAWRAIEESYATLSLRVTESLPLVFEKR
>CYP666C1|680313|Trihyb1
YLLALLWNYHIASSAGIPVIIVPFYYQNIFCVNWPYIPGLAWIVNNLLPSSTASYISDIGFTTRFWLRGKRFKTQGLCYF
TVSPRNIVLNVADADVVNQIMSDREGFPKPAHKYKIIDMYGSNLLTASDEDWPHHRRYIAGAFNEKNNNLVWEETLLQSG
QMLSIWKSRAAPNTTDPMAIDTLNYDAMTMALHVLSSAAFGIPLYFTEATTKRPEAEQGAGHEIFLDTEQPPTGFTMTYR
QSILFISQNISSTVGAVMFLPSWMKVVFKHQFAAHRNLGNYLRSIVEETQAKSKYGQDSLLSLMVRATQEDSASRRVGKE
SGKGFTTQELMGNLFIFTVAGHESTGITLQYTL
>CYP504A42|726506|Trihyb1
MSAQLLLITLSSAIYLLFHYANRTATPKIKDIPEVPGIPFLGSLLSIGSCHARKALEWSKTYGPVFQVRLGNRRVVFAND
YASVTDLWVTQQSALISRPELYTFHSVVSASQGFTIGTSPWSESLKRRRKAAATSLNRPAIASYMPIIDLESSISIRELL
EDSNYGEKEINPSSCFQRFALNTSLTLNYGFRIKDAEQLKEIVDVERQISNLRSTSNNWQDYLPILRWFSKKNNEAAETR
KRRDKYMDMLLGELKRRIAEGTDKPCITGNVLKDPEAVLNDVEIKSICLSMVSAGIDTVPGNFTMGLGYLSTPHGQEIQQ
RAYDAIMAVYPNNNAWEACLKEEKVPYITAFVKETLRFWTVLPICLPRVNIKEMVYNGAKIPTGTTFLMNAYAAAYDPSH
FAEPHKFSPERYLEGHVTTHFAYGAGSRLCIGHYLANRQLYTLFLRLIVAFRVLPAEKEEDKPVIDALECNRMMTSLTTD
PKDFKLRLICRDRGMVEEWLKGSEERTREL*
>CYP512CS2|732425|Trihyb1
VDVPTVGVPPGIFGPWKAALVFFGKSESLMREGYQKYKKDAKAFKVSTPSRYMVVFTHPKMIKELADLDDSIMSFQDSVR
ERMLTNYLFSEKFASQAHHITIITKNITNRLSSILPEVMSELIMAFEENTDIGPDWTSVNNFNVMLNCVARTTNRIFVGF
PLCRDQEYLDHVVAFAIQVTKSTAALDMIPKFLRPLAVIFISNREKTLKKVMISIKPIIEERRSKMQQFGEEWADKPEDA
IQWILETADPGESIRELCIKLLFLNFVSIHTTSFNITNVIFDLAAHPEYQEPLRQEIESVITEYGGWSKQALTAMKKLDS
VLRESQRMNGLIIGTGQRKAMVSHTFSDGTHVPKGSFVFAPTHAIHNDPDIYKNPQEFDGFRFSRIREQPGQQTKHQMVA
TSSEYIVFGTGKHACPGRFFATNELKILLGYIICNYEFKFENVKRPESTFYAYSCIPDVSTKIMYRERADRNASFANSCL
PMRY*
>CYP5945F1|734756|Trihyb1
MSLYSRQTFNNRGPFYEGGYAISRSYNVFSVRDNEQHRLWRRIWDQAFKASALSEYAPRVELHIEKFISVLGKTNGEEVN
CLKPIQNLAYDIMADLGFGRDFGMQDGEDGGHMEFLQSFIRTITTIGSLRNLCDMMPILPLGLKSAKSIHFQKKSIEMLE
YRKRMGRSRADVFTHLLAEDTETGTTFSDAELASNSRLLIVAGSDTTSTTIACIFRELALNPSTQQKLYEEVHTAAQNFP
VLDSKKTKDLPYLNGVVKEALRLWNPVPCGAETLTGPEGATVAGRYIPPNTTIRVHHYALMTDDRYFPQGDRFIPERWTD
QKMEGVKDIRAWVPFSYGPHACVGKHLALTEIRLTVARTVERFSIELGETYDDELFRKEWKSYVNVVLGDIPVRFRPR*
>CYP548BE9|738138|Trihyb1
MSLPLPFFDPKKQKKIHNNVVYASQVKSDIPCSADRNTAWHAWKGDRYLELYNAHQKYGPIVRVAPNMLSFNSATALKSI
YGHSPNSRNIQKGQFYTAYPAVKGVHNTHNAISKMEHGFKRRVLSVAFSEAALKTMEGTVLELVDRLVDGVRRDGPKGVD
MGERFSWLTFDVMGELCFGKTFGMLTDETQRFVTDLISKATHNHYICGNYLPIRFMGLGRLLFPTIAKDRWRFIEHSRAC
ANDRMKLDHAAKKDFFYYLLGAKDPETGKGFSTKELWGEANVLMIAGSDTTATALSSTLFHLCRNPSTMTKLQHEIRSAF
SSPDDIVTGKELADCAYLKACIDEALRMTPPVPGLLPRETVASCKIDGCELPEGTGVGVPIYTIHHNEEYFPEPYEYRPE
RWLHGDQAALEKAKTAFNPFSLGARGCIGKSVAYMELRLATAKLIWGFEMQQVCVQGKAEQWREGFAVKEGEYRLMDHFT

CKKEGPVVAFRERKVEGA*

>CYP6761D1|741523|Trihyb1

MGILLHLESTLEQVRLSYSIPTFRQFLIYAPLLWLIFCALVVIRRIWFHPLSHVPGPKLAAATHLYHAYYQVWRGGEFYQ

HRPALHAKYGPAIRISPRDVEIWEPELYHTIYKQKTSYRKDPVHYHSQGLTLSVATMLDPSEHRTRRALLNPMLSKRKVL

EASDVILQGQIEKFVRILEGMAERNVPIPLSHGFYAITSDIMSVYLFGKSWNLMDEPGFRSELLDSVLSIIDYFNLHVHF

KAFAQALAKLGVWFPRLIPVAVRRIRKNCEHLILEYLANPEKLAGNSHTTLMESMLNPPVGFPKQTFPFVDVVEEAVIMI

MGGTDSTANSLQFATWRFLTEPGVKEKVLAELDSVERDEHDRFQLHKLEALPYFTGFIKEVLRVYIIVPVRLPRIVPEDG

LTIPSTGLHIPAGSCVTQYIGLLHHDPRIFEHPEMFKPERWIGNPGLDKWLLSFSKGDRNCIGMHLAYAEINFVLANLFT

RFDLQLWNTTKEDMQWKDCGAARPVGRIHVIAKKRVR*

>CYP512CT1|745140|Trihyb1

MQIEIPAYGVPPGPLGSWIAALRFVRNSAELIEGGVQKYGPSGTSFKISTPARWVVVATSEAVLNELKDADPRVLSMQAA

ANERNSISYTLSSTIHSNPYHVDILTKNLTQRLSKVLPDVVDELRRTFEKNVPSGIGEEWVPINTHALMLKCISSTTNRI

LVGLPLSRDQEYLDCLVELSKMVSRAGLVIDLAPRVLKSFIAWCMIPRGSAFKIFLDKLGPVFEERRKRIAELGDGWTDR

PNDAAQWVVETAPQDASVYELCVRILYLDFSAIHTSSISITQALYDLATHPEFQTPIRDEIESVLAEHGGWTKPALTQMK

KLDSTLRESQRLHPVTTATMMRMTMKPYTLADGTRLPKGQWVVAPAWASNRSAEQYDDPLRFDAFRFSSAREEAGSEVRY

QLASPDKGYLSFGMGKHACPGRFFAAAELKVLLAHVICNYEFKLDAEGSKMRPHNSFFSFLCSPDFTARLMFRVRTDAGN

QKGHSELSGH*

>CYP52AU6|750502|Trihyb1

MHQLLGTNGIFTQSGSSWAASRALLRPSFDRAQVADLDRLEIFFERLRQRIEDDTSGCIELQNLLQKLTMDSSSDFLLGS

PVGALASEESGSGVNVQNFTEAFDIAQTVIATRWVLSNLYWLYNPKYFQQACSVVHSQVQKYVNRALKLRSSAPTTTTTS

PKKRYIFTEVLAETTQDPRIIQDQVLSVMLAGRDTTASLLSWTVLCLSRNPSVFQKLRAAISDTVGVDSSARIPTQAELR

SITYLRWVLHEVLRLYPPLHANTRCPIKPTTLPFGGGPDGTAPIALRKGEKVVASFFGLHRRKDLYGTDADEFRPERWGE

EKLRKIGWGWIPFNGGPRICLGQQMALTHASYFLTRLLQVYSVLEEEPGVKGLEVRYDTKITMYSGRGVRVRLG*

>CYP6713A1|764089|Trihyb1

MVDLPLSFLLTLSSATALIFHISTIRVELDYKLWNLGGVYLCVYGLLVYAFSLDSNLTAGALKALLLSICFNATLTISIL

THRIFFHRLRHFPGPFGAKVSRFWHIFKLWDSQAGHLLSQEMHKHGDIVRYGPCELSINLVDAIPAIYGPTSLCTKSLFY

NIVGPEENHSIFHVRNKQFHKERRRAWDKAFNGVNLAIYQPKIERCISVLLQELRTCDVTSDGINITLWASFLAFDVMGE

TGFGRSYNMLETGSLHPAVKCQKDSLPMYGIGTKIPWFVRLMMILPPSYSPIKPIMKWCGNEMEEKIKKFNQGEKPTDIA

SVLLCDEQCGLGKLKIEATHDDARLVIGAGSETTGMTLTGVLFYLATNRRVFTKLRNILDERFPGGESQYQYSPSLDIPY

LDAIINETLRLQPSVISGLPRLTPPEGVTINGTYIPGNVVVQVPTYTIQRDPRYFSQPLDFIPERWTDESPELCKDKRAF

MPFGLGSHVCAGKAFGIMEIRIAIARICLSFDWELAEGQTEKAYFEGQRDFFTCCLPNLFLRFTPWERG*

>CYP6643A2|802344|Trihyb1

MYPLLLLLLLPPTILLLHHRRRHHSTIPTFHPLSTYTTLPILLPTLLGHRNKTIHAAHIRHGPIIRLSPTEISLSSPETL

STIYTSFPKPSWYNIFRNYGIPPMFSLLPGKSHAARKKILAGSYSNSVVSRSAVLREVAGEVIPDLLSGFEDKEVEVWSR

FVELAMDFVTAFLFTREVGSRFLSGEDEGEILRRYHSRRGYFAVSSELPWLSRWVVPGWVDDANAKIEAWCERLCEARRG

EWKDGEEKCVIDRLLSSGLENTQIASEMLDHIGAGHETTALALSFTLAAMSTRPEMQRELRRSLEPLLEERDGWRIPRLR

EEDGYKALEEHALLNAIIKESLRLYAPIPGSQPRVAPRNMEILGYFVPAGTTVSSQAWTLHRDPAIWGDDVEEFLPKRWM

EGRGVEKAWWAFGSGGRGCIGQYLARWELRVVVASVYANYETMWKGEAVKVVDAYTTAPVEDVMVGFRRV*

>CYP51F1|814687|Trihyb1

MGVLFTLFDPLGSLTGDLAKLSTPLLLAIGAISFIIFSVVFNVVSQLACKDKNKPPLVFHYFPFIGSAVTYGMDPYAFFS

ANQKKYGDVFTFVLFGKRMTVYTHLTTPVFGEGVVYDCPNHRLMEQKKFMKFGLTTETFKSYTPLIVEQVEDYIKKSKYF

KGAQGQGPLGEIIPEVTIFTASRSLQGKEVRDALDGSFATLYHDLDLGFNPMNLMFPWFPFPGNRRRDAAQKKMARFYMD

LIAKRRKNPNAAEQEKDMIWNLMDRSYKDGTEIPDREVAHMMIALLMAGQHTSMATTTWLLLHLAEQPKIVAALYEEQQH

ICGQELRPLVYDDLAKMPLLNNVIREVLRMHPPIHSIIRKVKSPMYVRDSNYVIPAGYHVLAAPGTSAMDEKYFKNPNAF

DPNRWDDMEDEEEAEKFDFGYGLVNKGTASPYLPFGAGRHRCIGEQFANVQLGSIIATFIREFELALPGDGKVPPPDYTS

MITLPTPPAAVLWKRRNP*

>CYP51045A1|819192|Trihyb1

MATIAINTANLLAITAIAALASHHLIFRRYELDKYSAHILSAGVISHLFLAVASRHASVPSPYLLFMFSAVYISAVFTSI

LIYRLWFHPLHNFPGPFMARTSSFWQVFTNIKSDLRWRDVSKKLQEEYGDFVRTGPRELTVFDARATVALNGVQSKVMKG

PWYDGVGLNLMGIRDKNVHKKRRRVWDQAFSMKSLADLTPVMDQQTDILLQRFSESVDAGKNVCVTDWCFFYSYDIMSVL

AFGTCLDLLKSAENRWLSELVHSAMAVFGILTPCMWVMHIAKLIPGMAEGLMRMERYSMEEVKKRKTMKHPKPDLFKYLI

GGGLTDEILQLEARLIILAGSDTTFAVLTLALLHLALHPSIQQKLRVSILESFPDHSSENLLSTKCEYLEAVITEITRLH

PAVPPGLQRLTPKEGCWIGSTFIPGDTLVFASTYNIQRDPRYFVKPDEFIPERWTTSPELVIDKTAFAPFSTGPAACVGR

NVAQMEIRCVLMKTLRLYEIAATEGFDREVYFGGVQDKFTTALPGLELRFRKL*

>CYP567T1|819300|Trihyb1

MAVFQFQDIFQLPLWVLASLPIVFLVGRFLAHGIHNLYFHPLSKIPGPKLAAFTQHYFSCIYLSGRYHITVKDLHDKYGP

IVRLSPTQVSFITAKSWRDIYGHANGRKLFTKSNFYDGDLSIPRSIISSRDPEEHAGMRKLLSGAFSVKALTEQEDLVQA

HVDMLVKQIGVYATKEEGEDMVVWYNRAAWDIIGDLAFGDPFGSLKDAETHFWVAVMLDMTKAFAYFSMWVKYIGNSWWG

RVLKRMLVPKRLVKNRKRHRQYSHDKLAKRLAMETTRKDFLTNIINEEDVKHETLDAHASILVTAGSGTIATFLSGVTYY

LCRTPHAYKKLTEEIRSTFSSYNDITGQTAARCKYLGAVIEEGLRLYPPVPIGMGRLSPGEIVDGVFIPEGFEVFTSAWA

ASRSECNFHRPHDFLPERWLDKDCTDTLEASMPFHLGSRVCLGRNLAYLEMRLILAKIMWTYDMELKDNKLDYARDTEVY

VMCVKPKLPIKFTRREGAEVPLFDDA*

>CYP50127A1|832781|Trihyb1

MAVTIILISLFALYLTHHFYRSYQSRLLHSSFPACKPGIKIFDPFGLRRIRNVIKHLRQKRFLFYLGSVYKQYNRNTLPL

PLPGMTGYITAEPENIRALMSTEFKNCGVSEGRAAYGYFLGPGFFIQDNEAWSRPRALLRPNFARAQISDFTLLERMMQN

LFAAIEAHSGAFDIELLFQSLTLDVATEFLLGDSADSLAGGEGVLFSAALEKGLAHVNFAVSLGPVWWLWKPKAYRDSRN

FLHAFVDRYVVRAITRAEEGRTKDGYHFLDALTAEMRDPEVLRAHVLNTLFAGRDTTASLLSWLMWNLVRNPEVMARVKQ

EINAVVGDELPTAKLLEEMKYFKSVVNETLRLYSPVPLTNRESLHPIALPRGGGDDGASPLLIPANVTVMTDFFTMHRRR

DIWGDDAEEFRPERWLETENLDREMTGAFKYLPFGGGPRSCLGQQLAINTAMYTIVRIVQRYAGFEKKEGESDEVLFTST

PVPAPGAGVWITMSGKRM*

>CYP5093H3|839667|Trihyb1

MAARNFQTYKPYSSAAEQPLASMAPPLHVFSEHGRLQPYQSSMTAAIHLTSVMTLLILIMTLAFLYVVMTDRRRHAVMPP

GPSPVPFVGNKWDLPPCKPWYKFKQWTDMYGSLVTVWAGRRPTIIIGDPKVACDLLDRRSAIYSSRPRFVVMGELFTNND

SLLTMPHGDKWRKTRKIFHSGLLRKACETYKPIQEAETQRLAHALLHTPELFGRHLERYAASVMVCVAYGRRVDDLDDGD

VRRIYQRMQYMSTLNVPGAYWAESFPLLKLVPDCLAPWKREVKQQAESSSRLLLDLAQKVRNRMSDNTAPPSFTKTLWER

FDANPGELTEREVAYATGSLFGAGSDTSSSTLMSFILAMTCFPDVARKAQEELDRVVGRDRFPTWSDEPDLPYCRAVITE

TLRWRPVAVMGGTPHASIRDDYYEGHFIPKGTTILGNLWAIHHNEKYFKNSHDFVPERYLEAQEGTVPYPHRDGHSAFGW

GRRICPGKQLAENSLFISITRILWGFHIRKSIDPATGKEDEYNIFAYTDGFNSKPQPFRCVIKPRSDQIREVIERGAKLG

EKFLERYN*

>CYP6002C36|841479|Trihyb1

MRYLGILGSLPTPQRGAFDTDFRYRAADGSYNSLLFPSIGMAGTPYAKSVTAKVKLPAKLPDPALLFDTLFVRDKIIEHP

TKTSSLLFNLAAIIIHDVLKTDPMDTNKSSTSSYLDLAPLYGSNRAEQTLVRTMADGKLKPDTFSEYRLLGFPPGVSAFL

ISFNRFHNYAVTQLAVINQDGRFTKPAALGANPTAEAHDAYNKGMVKYDEDLFQTAKLVTCGLYVNIILNDYVKNILNLN

RVQSSWSLDPRDDFGHLYDQTTAIPSATGNAVSVEFNLIYRWHACISTRDAKWTENFYEHNFGVSDPSTIPPEELQRKLR

AWAQGLPKDPSKWEFSGLKRGRDGKFDDRELVDLITKSTEDIAGSFGPKNIPKVMRSVEILGIIQARKWKVATLNEFRQF
FQLTPHKTFEDLNPDPTIAQTLKNLYEHPDKVELYPGILAEDAKKPLVPGSGLCAGFTISKAILSDAVSLTRGDRLYTID
YTPSSLTNWGYTEVASDPAVIQGRVLYKLFMNAFPGWYRSNSVYAMFPFTVPSETKTILEGFGTAKDFSFDLPSYIPQPT
PCFTYRAVKEVLRDNNRFHVPWGPHVHELTGRDYMLSGDQPSNYQEKDGWVKSLYGPQNSVEEIEKFYEVTTWKMLKDKS
YSAGKGFRIDAVRDIGNLVQSHFVATLFHIPLKTADNPTGFTEEELYKMHATLFAWVFLDIDTAKSFELRQNARKAIATL
APAVQAAVEAVKAGKPPVNPLPNDAPLSKFGNQVISSLLQDKTVQEAVGTILPTVPAIVLIGQAIAQLLELFLSPPYNNH
WPIIQRLAKDTTNPASSQQLRKYVLEGTRLSPIAFGVIRQFVSDMVTIQDGPGRSVNLKKDGSVFLDLVSACLDPLAFPD
PLKIDLKRPEENYIQFGYGKHQCLGQQLAVTALTSLLRVFGKLQGLRIEDGQKMKSKVIRGSTRCYMKEEWDEWYQFPTT
LQMRFDSFVE*
>CYP539A40|844187|Trihyb1
MPISTMSSGFLFQSLPALFAALAVAVVLTTVIAKLNRNRQIFKLGKRSAVIKARFFGITEVLAIFKYARMHKTRELWTMR
FKQYKHYTIETEIVLQRFILTAEPENIKAILATQFLDYGKGRVFHDQWKEVLGDSIFTTDGKEWQSSRQLIRPQFIKDRV
SDLHIFDRHIQHMISLIPRDGSTVDISGLFFRLTLDTVTDFLLGESVNSLGAASDHGEVFAKAFEEIMLHMNDIMRAGPL
GIFIPKQKFRKNIKILNAFVEPFVAKTLRLRPEEFKSKNETDYNFLHALAGFTKDPKVLRDQLVAVLVVGRDTTAGTLSW
ALFELAKRPECVQRLRNEILNSVGPNALPTYADLKGMKYLQHVMDETLRLYPAVPFNIRVSLKDTYLPLGGGESGLEPVG
IPAGTRCAYSAMVMQRREDIFGPDSHEFKPERWDKWSPKPWTFIPFNGGPRICLGQQFAYTEMAYTLCRLFQNFDGVLDR
ATQPQSERCETTISPGAGVKVALNPVKK*
>CYP6713B1|846786|Trihyb1
MVFLSSVLGAFATHLLVLHREIDFHVLHLLGLYLVVFAALAYNLTPLSALVCAAYYNATLTASILTHRLLLHRLRRFPGP
LGAKVSKIWLMLQTWKKPQMHLLTDKLHKEYGDFVRVGPRELSISHPAAIHAIYGVNLQKSLYYGYSGSGNEASLFLLRD
GQLHARRRQAWDRALNGTSLGSYVPKLRVGVEQLVTELKARAGESVDISEWAKFFAFDMIGSVGLGKSYGCMESGKLHEA
LPALEGGNWFFAVPGLVPWLMKCLFSIPGAGGAMVSFYKWCTHEMELRIQEMKESGSERCNDVASHLLSDPRCGLGKIPY
SATLDDCRLIIIAGSDTTGAALGVALFFLTLHPHIFETLYSYHKSKDNAYLDAVINETLRLFPPVGSTTGLTRVTPKQGI
NIDGTHIPGDVFVSIPPYTLFRDPRYWTRPNEFWPERWLENSELGRGLYVPFSTGAYQCPGKQFAMAQLRMAIGGIVDAF
EKLEFVDPKDAQERFEADMLDYFVKHPPACRVRFTPRKTE*
>CYP567W2|853925|Trihyb1
MVLTLCCPSWILASLPFILIASHFLVKGIYNLFFHPLAKLPGPRYAAFSQLYFSYVFLSSKYPIIIKELHDKYGPVVRVS
PNQVSFNSASSWKDIYGHVGGRKPFLKSDFYDADPRPKSIVTTRDPVEHGAMRRLLSNAFSARALTEQESIVQSYVDLLV
KQIGKHATGKPEGEAMVKWYNWCTFDIIGDLAFGDPFGCLQAGVPHFWVSVIIDSINAGAYYAVLIKYIGNSKPALALKK
LLIPKHLMVQRKRHFGYGRDKMMKRMNNPNPTRKDFMTNILSKKENRGISVDALTVHGSFLIVAGSETTATFLSGTTYYL
CRTPHAYKKLVDEIRSKFATYEDITNQNAEKCTYLKAVIDEGLRSYPPLPFGLGRDSPGETVDGIFIPEGTEVFTSPWAS
THSEANFHRPYEFLPERWIDKDCTDKKEASQPFLLGTRVCLGRNLAYLEMRLILAKMLWVYDMELKNEKLDWLAESDCYL
FWKKPELRVNFTRRDGICVPPLDNDASPVVA*
>CYP6497A1|863230|Trihyb1
MSPTISLLIAVVISYHLYLIHLYLHRRSQAKATGLPYFSFPIGDTKLWYIILGLPPVIWVIENLPPRLQDYVNTSCYLRR
WNVKHRLSCYIADAAATREVFAGRGKYIKPSWNLMHMRLFGNNVVACDDTEWIHHRKHTKPPFNEHNSALVWQQALVQTT
DMLAEWESKPQSSPEYTSSGRIITSSRQDFRRLALHVMSSAAFGVSLSFSTATAAKKDITIGSDVFSDGEPSKGYSRTWR
DALEYISMNFITVVATVSMLPRWAAPGTVRVVQDVEKYLNTLVSYERANVNERNGKGNLLSAIVRKDRNSEEKGDFLTDR
EIVGNIFIFSVAGHETTASTLQYALVMLALHPEMQDWFLRRLDEQLEGLPVDAMQWDYEKVYERLSAPRCLMYEILRLFP
PVPGILKWISTEQSLTVGNSTHLLPGGTFVTINAGGLHHNPKYWGTNADAFDPSRWDLENRDSFLKSFEQPKDGWQEAPG
LYRPLQGSFASFSGGQRVCLGRKFASVEFVGVMAVLMRGRKVSLARMAGETEDMARDRAWGKVRKSIALSALVMMDDVGL
VLEER*

>CYP6742A1|866511|Trihyb1

MEKLTIMSSMLPHATIIQCLLQCFLFVFILSTLILILKSSYRLFFHPLSSIPGPPLAATTNLYQFFQYFYRRRWGEGQHQ

LRLHKKYGPVVRYGPNHVIVNLPHALPQIYHRKADKADWYRTGFEPITAFSATKHADHVVAKKRLAYGYSMTAMKAFEDE

VDGKIQEWVVALDRRYCESGEPLRFHEGINYLAYDVVTEIVFGESLGFVKEWRDVRGLIKSFEDFIPTIQAFGRLPLLKK

LPSIFGFLRPKPSDKHGFGLLMAEADRIFEQNQHLYEKKLEKFEKTSLLSRFMKATAQGGEPMTSDQVKFEAITAMVAGS

RTVSEVISPFIFHILKNPNCYSRLIAELCEAENSGLLGEVSGVVTYEAVIEKLPYFKACLREGLRIASVPFQMPRVSPPE

GILLEYEGKSTFIPPGVAVSCSLRLIARHKDLYGGDADVFRPERWLEADAETIKSWEKYNLSWGYGTRVCLGKNIAMMEL

WKICLQFFRKFEPELLEYRGGDCGDEYIDFMVRLRRRMEF*

>CYP61A1|872269|Trihyb1

MDSSAYNPPPAASAVELGGQAVVDGVGAVGKTLEGINMWQIVLTILVLSITYDQVKYIWNKGSIAGPTFKIPFMGPFMES

VDPKFSQYLAKWQSGPLSCVSVFHKFVVIASTRDLARKVFNSPMYVNPCVVDVAKKILRPTNWVFLDGKQHVDYRKGLNG

LFNRKSLAMYLPKQEEIYDHYFKRWLELSKDGKPRQYMTEFRDINCAVSLRTFCGHYITDEAVTDISENYYKITAALELV

NFPIIIPFTKTWYGKKCADYVLEEFSRCARLSKVAMEAGEEPQCTMDFWIKSMIDSREYKKLSQAEKEEYAGPKPIDVRE

FSDIEISMTIFTFLFASQDASSSASTWQFQILADRPDILAKVREEQLRVRDGDPYKRLDVDIVDKMVYTRAVIKEQLRYR

PPVLMVPYEVKKSFNVTPEYRVPKGAMIIPTTYPALHDPEVYVDPESFNPDRWLEGGEAEAATKNWLVFGAGPHVCLGQH

YAIMNFMSMVGKASMFLDWEHHPTPLSEEIKVFATIFPQDDCYLTFKERLPLRAP*

>CYP51089A2|874120|Trihyb1

MSLPVSVAGRLSMEVRGFVETHHRCLGYTKAFKKQLASGTRSEKRSVYEVFYAPSKNIPNTVDDRTLGELAFELLGSGTL

ASNAITFAAWFVANTPGVEERLLKEMEDAFPGNREMPYDTLSQLPYLVRDTFHATSAIAFLTIYLERYL*

>CYP6136D1|886930|Trihyb1

MKKQGIPGPFLAGFTCWYRAYYANIKRNWHAKLVELHEEYGPIVWIAPDEISVSDPKLRSVLYGFADERKEESFFPKSKS

FETGLFNEDFNFVFETDPARARLGKYTLSHPYSEKGLVRLEHNFDEAVQEFTQGFKEKVASNDKVHCFSDWTHYFMFDLA

TLLMTGYSNGLCRAGKDEGGAIRALRVIFNVVGSLVPVPFALTVSTKFIRKSILNGKLEHLFRWGICYSNGEVEKKDRIN

ELADKLPHNLMSKFRDGEKKVRKLFPKGNWTEAITNNVFFIYAGSMVASNALPLIIKLIYTHPEVLSKVREELADLPRAV

KIDDVTHHNGKCALPYLEATVLEALRLSPMFGLSLGRVVPSIGCQLNEYYIPSGYVVSMSGWATNVNKEYFGEDAKEFRP

ERWIGNHPTEIAKDGTGEPRTMRNYIEAGWFTFGAGSRVCIGRHLSVIAFAKFVGSFVRQFDLEIVNEGYLWYGLIQHME

EMTVKAKVRDSNADEVPVAEVPVRAEPAVITAEN*

>CYP6529A4|889281|Trihyb1

MTTLLYLSTEKSLEDASYIVATSVGSYLAALAGSMIIYRLWFHQLYKFPGPFMARITKLYYPYAYIGCSGKYHRLQKTWF

EIYGDIVRTGPNELIINDPSVIPALAKSSKGSWYALGHSLQSVQLVRDVLTHSIRRRVWDKGLSPKAIEFYLPGIREHTA

LLLSALEGETNITEIFGYYAFDVMGAISYGRSFGMLEKAGKDGSDYYRIMTHKSMWTIGLLGHVPWTVLLFEKLGVAGRD

YMRFMKWCSEIVEERLRRGGERDLFQVWMDAEPQNVGVHHIPLNGDSRTAIIAGSDTTASTLVGLFTHLAASPEILKKLQ

IDVDANDTDSEYLEACISEALRLYPAVPSGVSRKTPPEGITLENGTYIPGGINLLLPLYTAMRDPRWFDSPDEFIPERWI

DSNSEELARMNAVFHPFWVGRYQCAGKGLAMTQLKFVAAAVVKRYSFCLKEGLTVEMAMDGCIDTFTMEMGPVSCVFTER

NGKYEV*

>CYP6637B2|898719|Trihyb1

MTPLEITTPLSLDLKRFSALFVENKALGYVIALVTLQILYWICLSIYRLTFHPLAKYPGPWIAAVSPFFYSWAFGRGRAG

PVIRAAHEKYGPVIRIAPNDLSFATPNSYRDIYARSPNRKLFVKTLFYEEIGFGFEHIAFSSERNPEVHAKSRKLFTPVF

SVQGVRAYEALLMVSLEKFLAQIERIGSTPRGVDISEWFHRLLYDVTADLAFGESSGATDSADKAYWLKLVNDNINIATY

IEVANRYTTLRFIIKNLVPKRLFEARDRHLSWSIATTSKRIHNPAKIGRPDMLTYLMENDNAKGVSIAEMTSHLSSIILA

GGGTTSIVLGAMIYYLILNPDMLQRVRDETIHLFQTSDEITAPKLSECKFLTAVIKEGLRMMPPAPTGLPRVSPGETVDG

HYVPEGVEVIVHPWTLTRSTKYWKDPWKYNPDRWLDPESTDVKEAAQPFLLGPRGCIGQNLAWDQMRVIIAKIFYLYDLD

LVNAPEDWPSECQTFLTWSTTPLHVFVKRREGASSDPYFSRTRNIFK*

>CYP512CT2|904864|Trihyb1

MDISWISRWVVVATSEAVNELKDANSRVLSMQAAANERNSISYTLSSTIHSNPYHRLSKVLPDVVDELRWTFEENVPRGI

GNEWAPINTHALMLKCISSTTNRILVGLPLSRDLEYLDCLVELSKMVSRAGHVIDLAPRVLKSFLDWCMIPRENDAAQWV

VETAPQDAPLYELCVRILYLDFSAIHTSSISITQALYVLATHPEFQTRIRDEIESVRAEHGGWTKPALTQMKKLDSTLRE

SQRLHPVTTATMMRMTMKPYTLADGTPLPKGQWLVAPAWASNRSAEQYEDPLRFDAFGFSSTREEPGSKIRHQLASPDKG

YLSFGMGKHACPGRFFAVAELTVLLAHVICNYEFKLDTEGSKTRPHNTFFSFLCSPDFTAPLMFRVRSDAGSQKGLSELL

SH*

>CYP6501A3|912293|Trihyb1

MELLHYAFKLFLFYLLSTYTYNLLRHYLISLKIGLPVIFQPYQRLHPLAILTAGLNQPIISRLPFGLSRWKYLHFLWRDW

EFQTGFQQYAEYGEVFIEARSAGRVLFVANAELAWHIFMKRNEFPKDLGLYKIVQFFGENVLTTGGTTWRHHRKITSPSF

SESVYSDVWSETILLTQTLLGQWTVAQNKGQLVSKSGIPGIMFRPDMKTIARNVISKSGFGVSLPMISSVISEPETAKKS

PQEGRGDKIDVTDDAYFSPSFTPKGHTLPYGEALDILLENILLLAIVPRAILRRSTAGMKKAAQAYEDVGIYLKELLQRE

RSSSPSRQQNLLGVLAESSNSEEGGLSESETVGNIFIFALAGLETTAGALQYALLLLAIHPEIQDWLHKDIKNVLAQEDA

DPDPTTWEYNKLYPKLVGCTCVINETLRIFPPFQHLPKTTSQTPQTLTFDSKTYHIPAETSISISLTGLGLNPRYWGSSP

EKFTPQRWDARDPTSGWYDSDGSPIATDIQPGTQLRQPVKGAWVPFAEGFRSCLGKKFSMVEMVAFLAIMFAEYKVEIER

MEGESREMADRRAWGVARSSMATITVAMREEIGVRLVRRLLHPPIVQPKPKNKRTKASEAGLTIVATPVTRHSLTKKAGL

SSPLESAGQASAPASGKQQSSVARPLKTIDGWETARRILREMFYGWDLLTMIVMIYD*

>CYP50241A1|913626|Trihyb1

MLLHHKTKPRYVDGVPFVVTIPSIPWGGIIATFFHYFSFDKQHTTGYEKYSTKNRAFQVSTLAFYTTVVPSSWLNEVRRA

PKGTFDLYAAVDANFRPEYIFQMKRRIAEYNRWTIPSIRHHILPTMKYRIPALANETLYALDRYFPSKEEKWTSFYVYDT

IIKAIAPIWSRIFLGQYRCRNEKYLNAFVKFGRRSGIWSMVLNCCPEWLRPVAYRFSGVLGAQNILSEVTENLVAEKMEK

FKEEGLLADKGKNIEVIDSIDALIKVSCILDEPLESDIIMFYSLALNQAGILPNTMVSMNILYDLAAYPEYIAPLREELE

AVLEKNGGWNMETIKDLVKMDSFIKESSRLNTLAFSSMPRKVLAPGGYTFSSGLHIPKDSFISIPSYNTHLDPNLYGTNA

DTFEGFRFLKNGVDGPANKGSFQDPTEHYHTFGWGPAACPGRVITSPLLKIFIGHILLRYDIRPRERPSPLCLGNFNIPC

VTAKLEMRRRAFKAQDGLSS*

>CYP675Q2|945733|Trihyb1

MLLITLFSNPLFYPIAFVVSVVGWVVYQRVFSPYAAIPGPFWASITRFWYLNRINAEDMHRYTKELHKKYGPLVRIAPNE

VSVSDPAAMKAIYALTCGASKQTDFYPTQAPNLSPHGDSFTQLDEKKHTYRRRMIQNMFNLSSILESEQYIDNCTKTFMK

ILTEHAESGETIDIGTWLQWYAIDVIGELFFGRMFGFMNERRDVGGLIGAVDIILPHAIRMGVLYNWMRPFQILLVPFSS

SLRHGISVFNSLAAESKRLVDERWGLDKKSARTDMLAKLIQVAEEKAPEFDITDVYTESYTAIFAGSDTTAIVMRTAIYH

LCRTLHAKEKLQAEIDEAQREGRLSSIISYAEAIKLPYLMAVIKESMRVHPSIALTFPRHVPAGGRTLCGHFFPEGCRVG

VNPYVLHYEQSVFGEDAEDFNPDRWFRPEAENMDRYMFNFGTGSRTCIGKNIALAEIYKFMPQFFREFDVKLENPEKEWK

EHNTWFVKQTGIIVKLAKRGVV*

>CYP51092A1|981576|Trihyb1

MIIHLFQYPSILQALFSRLWAHVVFLSSTILVFSVIAYLVWCYYYEQTLSHIPKVGFGCFWTRPLAGVRVLLHCNDIMRE

GHQQFGIYGKAFRIPNLFSGSAVVLPPWQLQELRSGKKDSLSMHQWFDEEHSTPQVFFSPHLSSLGYHLQCLRKISKMPN

IVSDTANNVEFAFTSTLRRIGVDEMLDDGGWATLDPLQLAMGVVSRLTWVAIGGGELARNEALMSEVSTYGEAVFIGVMC

LRLLPFWARRILGPIVTLPNRFRVWRIERLFGPTVESRMQKLEDDLDSRKIDQDILDGVVNTSHALGPLHHNVQEIVRRL

LSVSWAALHTSASTLAHSILQLHHVDPESGEYYGTILERELRDVFDGLPTIGRELIREDTGWNKDSLSKLVGMGSFLKET

LRIYMIGAGNSIRTVVSPGGFTFSDGLYVPEGVTVCMYSEGIHLDPKNYYNPEVFDAFRFGSPFNAQTGLGDTGDAPKVD

WTMPNNTFLTFGGGNNVCPGRRLADIHLRIALSYLLENYELSLPPIRPAKTFMWLFQTPPLYTTIKIRRKRTNGQGLWSL

M*

>CYP504E25|873080|Trihyb1

MELPTLNFSKFLHGSELERVELSNALVDSFRDHGFVKIINHGVPEGTVKNFLESTRLFFSLPQEAKSRIVNVPGPHPQRG

WSWVGSEQTSRLWKGNLNGAMGKDEREHFDAGPANDKLYPNKWPREEDLPGFQSLMENCYEVLQDVSLQIMAAMEIGLKL

APGTFISRCIPAASEIRLNHYPKVSLETLSKGKIKRTWPHTDFGIITLLFQDTVGGLELQDRKSPKNFMPVTPGDSNSPT

EMVVNISDTFQRWTNGVIRAGIHQVSVPAPMKGNKDGICPDRYSSIFFFKADRNTSVGPLQDFIAPERPAAYEEMTALEY

QQQRTKSFTSTDIGRIKGIPEIPYALPLVGHLLLLGEDHASTCENLWHRYKHSVFQIRLGNTRAIVVNSFEDCRRMLIGH

QSNIIDRPTLYTFHGVISSTQGFTIGSSPWDESCKRKRTAAAVTLGRRAMKNYLEMFDFESYCVIRDIEKDSEFGTVEIS

LKPYFQRYALNTTLTLGYGIRLDSVYDDMLREILDVGSAISLLRSASENYQDYIPILRYFPNSEKKRRGKELRARRDKYL

GILLSTVKDMIQQGIDRPCVSSAVLKDVDSKLSSAEVTSICLSLVSGGFESIPGTLVSCIGSLSTLEGQKIQEKAYEDIR

RHCPNIGEAWQTSYQEEKVPYIKAIVKEALRYYTVTPMIPPRRTTSELNWNGSIIPAKTMILVNAQAANHDTSHFGPTAH

TFDPTRWLEATSPIPIERPSVGLQHLSFGGGSRACSGNIIANRLLYIALIRLITSYQIVASEKFPPNTDYIKYNSATSAM

VAIPKDFKVRMIPRDREGLKKVLADARCRSEHSCKA*

>CYP6001C28|891794|Trihyb1

MASNGSKTTTSAVAEASASRTSSNAPLTNQTTSKTVTRINPPVGKPTRKDIDATFTKFAALIHASNRPLPNRYGDGRDDP

DDEKLTGIRHDIMVLRRGGFITESLHTLWNVLQNKRKGGPTDDKTMIMERLIQLTSRLPPTSKLRLKLTTIQVGTLWDSL

QHPPLSYCGSKFVYRQADGGYNNIQDPNLGRAGSPYARSVKRMTQMPGAPPDAYTVFDAIYSRGKNGENFRSSNNNISSM

LFYIASIIIHDLFRTNRVDPNISDTSAYLDLSPLYGINEEQQKTVRTYKDGLLKNDCFAEKRLLAFPPGVSVLLLMFGRF

HNYVAAQLKVINEGGRFDLKYDRRWYGDDEATKNAKALKQQDEDLFQTARLVTCGLYINFVLNDYLRTIVNLNRVDTTWT

LDPRFEPSRMYNPNGTPAGVGNMVSVEFNLVYRWHSCISQRDDAWTQEFYKSLFPGRDTETLTMEEFIMGVHKWEQSIPE

DPAERTIENFVRQADGHFSDDDLVKLLTESIEDTAGAFGARNVPNVLRLVEVLGIEQTRRWKVASLNEFREFFGLQRHKT

FEDINPDPAVATALRQLYDHPDYVELYPGIVAEDDKEPMVPGVGIGPTFAVSRAILSDAVTLVRSDRFYTVDYTAGTLTN

WGIEEASSNPNVLQGCVGYKLILKAFPNHFKYNSIYALYPLTIPSENKKIYTALGIGDQFDYERPKFIRPRIPITSYAAT

KKILCDADNFKVTWGVGFDYIMEAKFMLSGDGAPYSEMKTFARDRLYGEGGSPRIDWKAEIRNFYRDLTTKLIRRTAYQI

TGADCYQVDAVRDIGNIAQTIFAATIFNLPLKSEDNPKGIYTEQEMYMVLCAMFIAIFFDVDSSKSFPLRHAAYTATRQL

GALVEAQVKAVKSWGWLQGVWDPLNIRGRNKSPLVDYGYHMVNRLLESGGSPADVTWKYIIPTAGASAPNQGQIFAQVLD

FYLQPENARHLEEIQRLATENTDEAWETIRKYALEGGRLAGTFGLYRRLDADEMTIDDGGRSVQLQRDDFVFVSFISASR

DPEVFPDPLEVKLDRPDESYMQYGDGPHECLGKRANIVGLTTMLMEFGKLPGLRRAPGLPGIMKTIPKPGGFKVYMKEDW

SSFWPFPTSMKVRFDNII*

>CYP52AV7|897496|Trihyb1

MGVNLTLKEACALVLSGFLLYRILSALLLWRNNRAFARKHGCKPPRRFPSGIYGLSNFWKVIAAAERKEHVQFIADRYKP

GWYTFVVNIFGSDVVQTVEPENIKTVLATLFKDFSLGPVRQEAFHAMLGDGIFTLDGKGWEYSRSLLRPQFSREQVADTE

VLDVHVSRVLDLIQKVEGKEVDLQPWFFGLTLDSATEFLFGESADSLLKEETGQKDFAYLFNEGQQWILWKLRWKKLSRV

WTPQAMIDVNNGVHKFVDHYVHMALNREKYPLPVASSKKYIFLDAVAQTNKDPKALRDQMLNILLAGRDTTAGLIGWTFY

LLARHPNIYKKLRGELESAFGTGEPGVWRRPSFEGLKDVAYLRYVLNEVLRLYPSVPLNGRDAVRDTILPVGGGDDGLSP

VFVPKGGRVQYSVYAMHRRTDIYGPDALEFRPERWGEGTKSGRGWEYLPFNGGPRICLGQQYALTEAGFTVARILQCYER

MEAVYPDEVPKIEATLTISPQQCLVRLFPVTK*

>CYP505A67|894758|Trihyb1

MEELTRNLAALETSDTFPEDDSKPTLLPIPGPPGLPIIGNIRDFDPAFPLSTFLNFAEIYGPIFEMTLGTAGRRVFISSV

ELFEEVCDESRFHKVVTGALETLRNGVGSGLFTAHHGEKDWLIAHRILMPAFGPLKIRDMFDDMHDIASQLVLKWARYGP

KHKILATDDFTRLTLDTLSLCAMGYRFNSFYTEEMHPFVDAMVGYLFESGKRAFRPSIANKLMRRTNARYDQDIKYMRDL

ARELVQGRRNNPTDKNDLLNAMINGRDPKTGEGLNDELICNNMITFLIAGHETTSGLLSFTFYNLIKNSAAYRAAQKEVD

EVCGKGPITIDHIPKLKYINAVLRETLRLNPTATTFSLAPHSDLDEHPPTLGKGKYSLEGVPAVICVLQKIHRDPKVYGA
DANEFKPERMLDEAFEKLPKAAWKPFGNGMRACIGRPFAWQEAVLVTTMLLQHFNFQFDDPGYELHIKQTLTIKPKDFYM
RATLREGMSAVAPSAAADVIHSDGGSQKHPAKETSGSDKKQQRKLMSIFYGSNTGTCEALAQSLATNAAGHGFDADVRTL
DSATEKLPKDHPVVIITASYEGQPPDNATHFVEWLKSLSGNEASGTNYAVFGCGHRDWQTTFQRIPTLVNDTLHKLGGTR
MVERGVADAAEGDMFTKFDTWEDELFWPAIDSKYGGTSVAAASTTLVSTLTAEISSNRSSKLRVDVSDARVIATKTLTAP
DQPEKRHLEIQLPSNAAYSSGDYLAVLPINPKQAIHRAMKRFQLPWDARITLTSTAPTALPTNYSMSAHDIFAAYVELSQ
PATKKNVLSLAAAAVDPETKSQLEALAGDQFQAEITQKRISPLDLLERYPCLHIPLGSFLAMLPPMRVRQYSISSSPLHN
ADTCTVSYDRLEVAALSGSGGNHYGVASNFLSSLEPGDILRVAVRASAKPFHLPLNPAMPIVMIAIGTGIAPFRGFVQER
AVQILAGRTLGNALLFVGCRYHDKDRLYAEEFDKWQEQGAVQLRYAFSREPELSEGCKYVQDRLGKEKAELFKAWEDGAR
VFVCGNARLGEEVAKVCKDGYRERRKNQGEETTEEAAEEWFGKLKEERFATDVFD*

Tuber aestivum var. *urcinatum* v1.0

>CYP61A1|645|Tubae1
MDEIANSSTFTPPSAVGSAEQPFQTLVESFETIGRTLEGLSGWQIFFSIIILSITYDQCRYIWNKGSIVGPSLKIPFMGP
FLESVNPKFSQYLAKWNSGPLSCVSVFHKFVVIASTRDLARKVFNSPGYVSPCVVDVAKKILRPDNWVFLDGRAHVEYRK
GLNGLFSRQAIGMYLPGQEEIYDAYFKRWLGLSKEGKPIPFMSEFRDINCAVSLRTFVGHYISDEAVKEISENYYKITAA
LELVNFPIILPWTKTWYGKKCADFVLNEFAACAAKSRIAMKEGKEPGCTMDSWIKSMIEAKESDAAKIAAAKGLPQIREF
SDMEISMTIFTFLFASQDASSSATTWQFQIMADRPDIMKKIREEQLRVRDGDPHKKLDPDLVDKMVYTRAVVKEQLRYRP
PVIMVPYEVKKSFQITPEYRVPKGAMVVPTLYPALHDPEVYNDPDSFVPERWLEGGEAEAAKKNWLVFGAGPHVCLGQNY
AIMNFMSMIGKASLMMDWEHHATPKSEDIKVFATIFPEVCFPMLLQEQHRTNLIFRMIVSWCSRSVIHTLPRNYLSLCPP
PPISLTFTPPLQSFQGFFLFLPLNYKPFPSSLAGGCCI*
>CYP6498A1|1096|Tubae1
MGTLLALAICLGAVTTAYAISSIIHLLYNIREVRKSGLPYIILPWHEMNPFHVLTCGFNRELWQYLPKWTYFKFFWRDWC
HHTKFELFKEYGDVICAVSPGGVTIYVGSVEVARQMYERRNDFPKATKVYEYVRFYGDNVLTLEGAEWRRHNKYTRPPFS
EAVHKVVWDEGIKQAYAAVNVWSRGNSGVMVGRDLRTISMNVLSLSNFGVALSFDCGNETTEHFGQKNIPPGHKMSYGNA
VNHVLDNIIPLAIAPKWLLRNGPESLRKIGRSYDELGIYLRELTQTGGRAATSGRRNLLGSLAKASVGDGREKGPGLADA
EVIGNAFIFAVAGLETTTGTLHYAIIHLALNLDVQDWLHRDLQEALKDEDQDPSMWEYEKVYPKMASVLCVIHETLRLYA
PHAHIPKWTADRYQPVNWRGRECLMPPGASAYITTTALHYNPSLWGDTVKGFHPQRWDLRSSSEGWVRTDQETGVKTPIT
PPETPRTETQSAPYCYLRTPVKGAFAPFSDGWRACVGKNFALVEMTAILAALFRDSSVRIKRQEGETQEMADDRGRSAIS
NSEGYLTVMIRHDVELEWVRR*
>CYP50026A1|1913|Tubae1
MDQATDLLSRVEHLLLKPLHHSSLQALAAFLVWYVVYQRYFHPLRKFPGPFLASLTVFWRLSNILTFRQSLNDHALHQKY
GPVFRDGPNSLSIADPRALEPIYGTRNELSKTPWYLIMDPDNTGEDYSVFSSRRAEQHKRLLKKRIAGAYSMTSVRVYEPV
IDRNVNDLLAWMKELKTLDVSVWTHYFAMDCMSEIAYGNRMGFLINGTDVNGYRKALHESVVFIATMGYLTGLNYVIKSK
WLSPYLAPSPKDKHGYGHMIGMTQALATDLLENGNTTGKRNMSHDLLQCRNDDNTPLSKKELIGEMLAFTTAGSDPTAYE
ISSILDRICRHGEVREKVLQELRGVGELEQSSAEGVVTYAQTLRLPYFLAVVKETMRLSPAFQGQFSRVAPEGGEGLEVL
PGVVVPGGVWLSVNTYISQRDKLIFGEDAEEFKPERWLPIGGDRYHAMAKHLSVFGYGSTACMGKYLASQKINKTVVEIL
RRFNVELRDPKAPLKEKNIIQMFISDLFMTFTERENKI*
>CYP5093H2|2036|Tubae1
MKGTMIASAPRVPQSSPFTSSVEAPLASLVTGLHLSSGLKFSRQYEGTVIVPKDPVARFHSLSCLAACLLLSALAISCLL
VALQDYRRHAIMPPGPSPVPFVGNKWDLPLQKPWYKFKQWTDMYGSLVTVWTGRRPTIVIGDPKVACDLLDRRSGIYSSR

PRFVVMGELFTNNDSLLTMPHGDKWRKTRKIFRMGLHGRACESYKPIQEAESQRLTRDLLVTPEIFGKHLERYASSVMIC
VAYGRRVDSLEDPIVKKIYDRMAYMSTLNVPGAFWAESFPILKLIPDCLAPWKREVKRRAKASTEMLSRLALDVRDRMKK
GDAPASFTKTLWEKKEGNPEALSEREIAYATGSLFGAGSDTSSATLMSFFLAMTCFPRVAAEAQEELDRVVGRDRSPTWS
DKPNLPYCHAVIKETLRWRPVAVMGGTPHASIKDDRYNGHFIPKGTTILGNLWAIHHNEKYFKDSHDFIPERYLGSGKID
GMEPYPHRDGHSAFGWGRRLCPGKQLAENSLFITITRVLWAFNISKATDKSGQEITPNIFAYTDGFNSKPQPFQCLIQPR
TPGIRQVIEREASLGEQFLDKYKCN*

>CYP5959A|2409|Tubae1

MATIMQLIDNTKDEKLYAYSLFGSTLALLFSGLFLLWRKDWSIHRATGLPIVGIESPGYLGLVKARSNFGANGFRIVRNA
YKKYPRKNFVVTTYGYDKIILTHEQVKELASAPDDTINATHAIVETLLGDYTGLGRFVDARYIEDVVRIKLTQNLVIGSK
IKKKKTLVPGHLFPSTSKQFYQLSDSALLTKTQPTGNLKEALLEEARFALNTEFPECTTEEWTSVGIFIPILRMVARVSG
RAFVGLPLCRNEDWLKITIQFTGDVFTTFSKLSTVSKLLRPLYAYMWNSVKNLTASRKKGEALLTPILRERLEEKRLAEK
NGAVFQRHNDMIEWLSDRVQPKDRNPEILSELLLLVGLAAIHSTSLAFLNALLDLAQHRECIQPIREEIETVLSENNGVF
DREALRKMRKTDSFFKESMRGKVGLFSFNRKVMKNYTLSDGTYLPKGTLVTAPYSMFSVDPDFIEDPEVFDGFRWYKKSL
GTEGGGGPGRIQYWATTSPKDLNWGHGKHACPGRFFATEEMKILLAFIILQYDIKYPEGQSRPETIRNGEFTTPDINQKL
LFKKLPGPKKFSFL*

>CYP6271A1|2513|Tubae1

MTLETVISYVREQSSDLSKGTIAATVFGVWLLNRIILVLYRLYLDPLSKFPGPKLAAATSMYEMYYDIVEDGTFVWKMDE
LHRKYGPIVRVSPHSLRLRKSSAYHEVSCSSRDRRLSVLRAKILKVHRMGSPFAKDNRFYHFSGVPRSTFATIDIDLHRQ
RRAPLNPMFSRRGILDTEFFVKEKIELLCRRMKEHEVQGKLFNCYNGFAAMAADIITEYAYGKSYDVLKSPDLTCPAFTS
LHAQHELFLILRHFPLFRMAMQSLPLSVLRLVPAGAGLAELEEEAKANLKAVYARMGTTMEKNSAHRTVFEELLARLPDP
KNADPTDLTNEAMAVVTAGFHTVRWTICTGILEVAGNPLIQTKLFEELKTASPDVNAEFSYLQCEKLPYLRGVILESLRL
SYGIIGPLPRRVPKEGAVVGGYHLPGDSTIEMDNYSLHHDEEIFPDSHRFWPERWLTPESKQKEKFVNSFGAGPRQCLGI
NLALCELHLTFATVFRRFEVDISARGNKRMKIKDHWLSILRDEPLKCKLISRKE*

>CYP5959A3|4118|Tubae1

MGILTQLVDHSEYKVLYDYSLLCTTIAVLFLGVFLLWMKEWSIHAPTKLPIVGIESSGYFALIKARRNYYKVVLTHDQVK
ELSNAPEDTVSFIHASAETMMAEYTGLRQFSVDQYIPDVLRAKLTQNLGNMKEALLDETGFALNSEIPGSAIDDGQDFHR
TYFGVSILIPTEWVPVNVFTTILRIIGRINGRVFIGLPLCRNEDWLTISITFTGIVFRAIEKLAGVPKLIRPLYSYTRNL
VKDINLHKRKARSLLSPVIQKRLDEETLAKKNGRAYEKHNDILQWFTDRVNPEHKTVEALTETQLHLNMASVHTTAIYFL
NAILDLAEHQECMQPIREEMEAVISANGGELDRTTLRNMRKTDSFFREAMRGKFALFTFNRKVMKNLTLSDGTYFPKGTL
LAAPSAMFSTNPEFIENPDTFDGFRWYKKSLEAEDRGADNKNN*

>CYP6188A4|4207|Tubae1

MSKVFRSIEPVLRGLHLEALAQKMVPENTTAKYLIALLTTHSAGFQWDHIFSLIAGLLGVLFVYMVVKLGLHLQKTRNDT
PLKDVPGPWLASCSPLYRFWYAVVKGNFHNDLTNLHRKYGNVVRIAPNEVSIWDPLVTREIYAHGDKGYPKCDMYDIALP
NGYFNLAVERDIQTHAEGRRAIAKDYSMTATLTAEAHFDNVIGDFILALDRNFAQSGRVCDFTTWSEYFTYDMITDLVFG
EAYGFCRTGIDVDGSLKDLRQMLLLSPFLSYLPWIWPITQNRWVKETGMNHYARVCHTPILKRLNHGNVSGRLDLLDGLL
KSRYRDGHALPMGEIVNHAYIFILAAPDTASVALRALLINLVRNRDVYNAVMDELTGLKLSNPATWKELADAPLLHAIVK
ETLRLHPPAGFNLPRAVPAGGRTICGYYLPEGTTVGMSAWCVHANENFWGKDTLEFKPERWLDPESAFKLDRYGLNFGQG
ARACLGKNIAMVQLVKVAAQILLNFEFELVDEHKIRGLFLLLVVLDGVKVSFKRRVGGPLDDTVEMEGAKF*

>CYP665B1|4458|Tubae1

MAIPVFWTTAPGLALGLCLASFIATIVYFVKGTSRGKNFPPGPPTLPILGNMHLVPQERPYLKFTEWAKQYGGIYSIKIA
KQTIVLISDVKILKELYDKRGAIYSSRPLPHIGAEVVCPDQTHIIFMPYGETSRSYRAQYHQFMGPGKVEQILPWQSAEA
TLLLKKIATSPDRYYEHTMRMATAIILESVFGVLPKDYDDPEVTELWTVQREFSEVLALTGPPVDHFPFLKWLPDIVSPW

RIHARNIRAMHRKLYFRLLNLNKARMEKGERYGTFVEKLIDDRPKHGLSDERMAYVCATLMEAGSDTTASQALDFMMALL
AFPNVLKKAQEEVDRICGTSRLPTLDDRDQMPYIEACINEVLRWRPPLPYGAPHLLMKDDWYEGYFMPKGTVLFQVQWAM
NMDESVYERPQDFVPERYIRNRFGTKFDAEKDAETGRKEQYGFGLGRKICPGQWFARNTLVSYPPLPRGIGFGADATQFV
LFAKLVWAFDMKIPTDPKTGKPVPLDTDVRTAFMDGLTTTPFKFPIDFKIRSKAHEEAMNADLVESDKVFAKYGGATV*
>CYP51062C2|4474|Tubae1
MFTDPVPELDCHGYYFRGGVFFGSMFCVLVWWIVNRQAPPTAANGRKFQSPPMLQSKLPFGIDSMLTLMRSVREFRLPVA
LMELHDRMGDTYACMLFGMRAIRTRDPRNIKAILATQFEGKWYFPLNYGLAQERKAHHSLLGNMGIFSVDGVSWSHSRAL
LRPCFDRANVSDLGQLEGFMQIFFSRINELPREPFTHARAVELQELFQCLTMDSSANFLFGNPIGALAPKKYALGNGEMT
FNQAFDIAQYGLAMRAPLSSFYWLYNGREFREACSTARTQVAVYVSRTLRKLNLAEKNPEKKGLGKKYVFLEELAERTQD
PRVLQDQVLGVMLAGRDTTAALLSWTFLCLARNPEVFRKLRAEISAAVGVDGDARLPTQEELRSMQYLRWIIQEVLRLYP
PVPINARRALRATTLPYGGGPDGNSPISIRKGERVVYSAFSLHRRTDVYGPDANEFRPERWGEEALRRIGWGWLPFNGGP
RICLGREFTTLSPLLEIVLTVETEQMALTHASYVVTRMMQVYKEITPKDFTLADDAYDTKLVMASGRGVHVFLS*
>CYP50043A1|4562|Tubae1
MIWVGRVYNFEHPLLGQCLIHHRHTNTNITTLIIMGILNIGLVTSAVYLLGAWAAYLVLLVFYRLHLHPLRRFPGPKKAA
ATGWYSAYWDLYVGGQMAKHLVDLHKEYGPIVRFEPNHLHFSSPEVYSTIYSSTSKLTKDPNLYLSFGAPESVFTLLDPA
IARTRREVISPMFSRRMVLSLQPLISGKIRKLCDKLSGYADRDEAADIVSGFRSASIDIITQYCYNECLDSLDVEGFKHD
IVLTIKATSQSFWFVKYFSLADWILTLPTKVTLRLLPELKGFVDLRESIEEQVKRYMKNPSLLEKSSHPTVYHRFLDPEV
KGGIPSASSLSDEAQNLFFAGSDTVGTTLGFGTYYILATPGLQEKLFAEICEVWPVLEEEPTYEHLEKSAYLTAVIKESL
RISHGTVAPLSRVVPASGMTIQDQPIPGGTVVSMDAPTLHLNPTIFPSPDTFLPARWLDSNAKDLENVQQRTASMYWIKV
TTPPNISSLDFPGANDDSLAWAELYIAFATVFRRFEMAVWETSKEDMEWIDCFTPCSKGELKVKFKIRRE*
>CYP6592A4|4591|Tubae1
MNTTYTPIPWREHTTLSSIDSSLLALALLLTLIVIVYNRYFHPLSPFPGPFLASIWSGWRAWGDYGNLEDQDPLLHERYG
PIIRVSPNVISVADPEYLKQIYTQTHRTGCWLSFAHNGTVYGSSIRSSALHEELGKRAKTLYSADVTGGWEGVVDRKVSE
WIGKLKGECSGGWVRFNLSESARALACDLLTGNVWMAKGDKVGPAAVRAKAGSIIDVAWKARKEVEACGEECYLSRERTT
QEVNLFGESTTLTINRAISIKKPNRTRLTKEEVYFPCTPPPSPQAALAKANTPTGLAGEDSTAFAITSALKLLLTNPRVL
AALLSELTTYYEKKALPHIPPWGDIAHFDTKLPYLSAVLRESLRFHPTFPMSILCVAPPSGVTITHSNRLYTIPGGCTIR
ANPIVIGRNKLVFGEDAHCFRPERWLEGSEEGIEMMKAVELEWDTDSGDCLGTALARMAVAKAIVMVLRNFEVGLVGGRP
RGEAWKLAATGVHHATELWVKLEPREDKMDGNRRAGEMGGASELS*
>CYP51F1|4676|Tubae1
MGVLSTLLVPMGPFYEQLVKLGGPALCAIGFVAFVLLSVIINVLQQLLFKDPVKPPVVFHYFPFFGSTVMYGMDPYKFFS
DCQEKYGEVFTFVMLGRNMTAALGPKGNDFVFNGKLSEVSAEDAYAHLTVPVFGEGVVYDVPNHVLMEQKKFMKFGLTTE
NFRSYVPLIVDQVEDYIKKSKFFKGAKGSVPLSEIIPELTIFTAARTLQGKEIRDALDGSFAKLYHHLDSGFTPMNFLFP
WFPFPQNNRRDHAQRTMAQFYMDKIEKRRANEKHDDDQERSDMMWNLMNRSYKDGRKVPDKEVAHMMIALLMAGQHTSMA
TITWMLLHVAAQPKLVAQILAEQKRVFGDELAPLSYEKLVLECTFLGQIIRETLRMHPPLHSILRKVKSPMHVDGTNWVI
PKGHYLLAAPGVSSMDQKYFKDPNSFNPSRWDGQKAEEETEKFDFGFGLISKGAASPYLPFGAGRHRCIGEQFANVQLMT
IMATFVRNFDMQRPGGGSDVPAPDYSSMIALPTPPCTIEWVRRDP*
>CYP51070A10|4816|Tubae1
MRKNMTEKLSSLLPDVINEVTTGFEELTNIRNGSNKQNFWYIIILLLSCIGIRYMLIFLVGMPMCQNKDYLESINEFSINV
MRRATVIDTVPWFLRNIVSYFLKNSTVEDVIMTHIGREFEARQKARWFQSSYDIVVSDAIQWILDAAPPETPILKLVQKL
MLFNFASIHTSSISLAHILYDLAANPEFQDPVCAEIEEVLRAEGGWTKQALTKMRKLDSIFRESLRMNGTNIVTLIRKVI
TPYTFIDGTFVSKGAWVGAATINIHYSPDLYKDPEKFDGFRFYKKRQLEGNAHHFQMASPTLDYLPFGFGKNSCPGRFFA
SNELKIAVAYILCNYQLRLNGGTTGERPQNIYDGLVCLPNPTVGIELKEREGRQKSILYPGHMASYGN*

>CYP663N1|4866|Tubae1

MSLTQTIFSLPGGSTAALILLVLSAGILAQQLFRGKDPLPLPPGPPRLPIVGNSFQIPLINPWRKQAEWTKQYGPIYRLK

LGKDTVIVLGTQKAARDLLEKRSKIYSSRPRSVMAGENVSRGKQSLIHPSYVEIILMKCDKWRMHRRLQGQVINQNIVNK

YKGFQDLESIQLIKELVDRPEGFFDSFHRYNSSVIFALAYGKRMPRGDEEDVVAIDEITSNFLYSARLGTWIVDSFPLLN

YLPTFVAPWKRIGDNFYNWAEKMHTFNRDEALQRKGWNWTKEIARTKEAQNVSPLELAFMVGFLYEAGSDSTTIALEIFI

LALLKHPEALKRAQEELDRVVGPDRLPTFDDKDNLPYVCNCVDEVLRWRSPSAGGVPHVVEEDDEYMGYRIPKGAIVVGN

LWSIHQDPEAYPNPTKFMPERWDDRENVHYGFGFGRRACPGRHISSNSLFVNYARILWGFNVEYAKNSDGTVIPVDEWNM

TQGFMSRPVRYKASITPRDAKRVEVIDRAWKGAEMQLQDMSNILKIEDF*

>CYP5945C3|5465|Tubae1

MSGADTRIEFSKGSGSVEVSQNYGHGMVKAPSDFVSGFPVLIIGTATIAASAAFLTQQLLSPIPEVSTTKSILFYTIANG

LLALALKRAGIDVFLHFLARISLINISYLTSLTFFTIVHRLYFHPLRNFPGSKIARLSKLYEAWLNYNGRNGLVVRDLCR

KHGDFVRTGPNELAISNVEAVEIIWGRTRPTARGPFYEFANFVGERHLVSQRDKAVHAAWRRIWDRGFTSQTVASYSPRV

KYHVDKMISILERLNGEETNSVDIYDNMTFDIMADLGFGQKPEESMQSGAGDPSYMRFLHGWMRASTSITGEIGAYIPQD

AESKEFDKMGEKMLGARQKMGKSRQDIFTHLLDQDNESGVNFTQTQLLVNTQLLMVAGTGLSTPSSQATTSMRPNLLKRP

LDTTSVTLTCLFRLLSMHAEKQQKLYKEIMETFPNGETPTCANTAALPFLNGAVQESLRMWPAALPSGPQATTPPSGYTI

AGTFVPGNVEVRIPPMTIMSDARYFPQPDEFLPERWTSEMPELVKDKRAFIVFGFGAHSCIGRPLAMNEMRTTVARVIQR

FEVQLGESYDDRVFRDGWRDYSTVKLGPCPLKFVPRKM*

>CYP6136A1|5904|Tubae1

MFLSLLGWAVYSLVQHKLCSLAAILILTLAYWTRAYLKSPFRAQQIPGPFLGKFTNAYRWYYIMRHTWHRDLMDLHKKYG

PIVWIAPNEISVSDPSLRNVIYGFQNHKKDFTFFRKSPSYETGSINQDFSFIFEQDPERARLGKYHMSHFYSEQGLLNLE

ENFDKAVDELIEGLDKHHVKTGTPCKMVDWAEFFALDLVAQITADQSAGFCLAGKDVNDTAYGTRVIIKTVGSLMPLPWV

LTATSRAIRQTLLLRFLINLYRNVLLLPTFAFDTGAADLISLKDRNPKHLLAKFYNAQSNMREHYPNGNQAEGTTIQIFN

LIAGAVGVVPHTQVKLIHELTLHPEVLQKIREELQTTGDTFRLEDYLPYNNRQNKYPIFESAVREAVRLWPAVAFSLSRV

VPPSGCQLHQYHIPPGYNVGMAAYQVNYDEGYFGSDVAQFRPERWLEDHPTQMLGGEKRSMKNYIEAGWLTFGSGGRVCI

GRHLAMFMMMKFTAAVVREFDIRVVKQPVEYHTLMTEMLGMEVLLSRKRAV*

>CYP5959A|6270|Tubae1

MGILIELRDYAGDGKLHAHILFYATLALLSFGLFLLLKKEFSIHSPTKLPIAGIESPGYLGILKARFKFVSNGFNIVRDA

YHKYPGRNFVVTTYGCDKIILTHAQVKELASAPEDVVSIQHASAETMMIECTGLGRFLGTGYAEEVVRVKLTQNLANLKA

GILEETRFALDTELPGCSTDEWRSVEIFTTILRVVARTSSRAFVGLPLCRNEDWLTVSIMFTRDVFMTLGKLTHVPKPIR

PLFAYIFNSIGEIRSHKEKAQALLDPIFQSRLEEERLAEKNGTVYQKPNDMLQWLTDRVDPPYKSVESLSELQLLIILSS

IHTMSLALLNTIFDLAEHQECVQQIREEIEAVLSANNGVLDSAALRKMKKTDSFLKEALRARIGLFSFNRMVMKSLTLSD

GTYLPKGVVIAAPTSMFSTDPDFVEDPEKFDGFRWYKKSLEAEGGVLHSNNFTTTSPNDLTFGHGKHACPGRFFAAEEMK

IILVFLLFRYDFKYPEGQSRPRNLNHGEFSYPDTTKHLLFKKSTGPMKFPFLQ*

>CYP539A44|6872|Tubae1

MLFGDTIRSVPLLLIALAVGGVLTSAFKKVRENWKIHKLGGRAKSVPCRQFLFGLDIVARAFVAYKANKNMELWKSWFKV

AGSHTIEANIIGMRAILTEDPENIKAVLASQFHDYGKGEPFRRDWKPFLGDSIFTTDGQLWHASRQLIRPQFIKNRVSDL

DIFERNVSHMLEYIPGDGATVDISDLFYRFALDTATDFLLGHSVDSLGSPQVEFARAFDDIQKHMSIKSRVGPLSWIFPE

RKYKRDLKVLNSFVEPYVEQTLEMRPEELKSRNEKSYNFLHALAEFTRDKQMLRDQIVAVLLAARETTAVTLSWTLYELA

RHPELVQKLREEILERLGPGGKPTYTDLKVMKYSQRVINETLRLYPAVVVPFNVRMSLKDTNLPRGGGPDGLDPVGMPKH

TIFAYSTLTMQRREDLFGPDVEKFDPDRWERWAPKSWQYIPFNGGPRICVGQEFALTEMLYVLVRIFQRFGAVESRQTEE

QYQSCNIVISPGTGVPVSFKPARFPEPEITTR*

>CYP6608A1|7463|Tubae1

MFLKLLFLSAFTQVLPLTLLALVASYVYSRYASPLKKVPGPALASFTRLWLNVGLVARGNLHGGILDAHRKYGRVVRVGP
NKVYFSDPALIPEIYGVGSKYRKSDLYMMADPEPGQGSTFSERSAEKHAALRRRVGPAYSNSSVLQMESYVDALVSAWIS
ALSTEFTATETSFKSCDFACWAQFLTYDVISELAFGKAFGFIATRGDHKQYIASLQQALPIIVILSYFEELVKFMALGWV
RKFATPIINDKTGYGNLTKAATELVRERWAKGGVGERRDMLQRFYEQGMTEKEAMVDSTLILLAGSDSTATAIRAAVMFI
CSNPRAYITLKSELAAKIVRTTPPSIVSFSTARSLPYLSACIKETLRLFPPGAGAMERVVPAGGAILSGYFIPENTIVCI
HPWPVGRDEVFGEDGDLFRPERWLEEKDEKRLAKMEKALDFVFGAGPHYCLGRPIAHMELYKSITELFLTFDIGLSNPMQ
PWKSREYGFFLQKHMMVNLKRVDG*

>CYP663L7|7780|Tubae1

MCTVGDCTETPGGGASSPITTRPLSLLISFLAILIATYLFQRKRYQLPLPPGPRGLPILGNIFQLASKYHWLQQQEWTKK
YGPIFKIRFGSHTIIVLGTYKAARDLLDRRAKIYSDRPDLVVCGKHISRGYRTLLMRGEMWNAHHRLQATILSPKMSQKY
KPVQDLESKHMIHALLKKPDDFARQFHRYSASLVFSLGYGKRLVTAHEKELRAIDLIMRNFTEAGAVGRWWVDLFPILDL
LPECLAEWKRVGLKFHQIECELHLANMREALEEKGWNWAKVYKNSPLSQGMSELEISYDAGILYEAASDTTTIALEVFIL
AMVKFPHVARRAQEELDRVVGESRLPGWQDQASLPYIQKVIKETLRWRPVAVNAFYHAVTEDDEYLGYRIPKGSWIVGNV
WGIHMDPEVYPNPNDYNPDRYDDETLGYLSFGFGRRACTGRHIAKNSLYITISRMLWAYDIGPNIRPDGAEVPVDDMAFT
NGFLSRPVPFECSIKPRNQNRAEVIEREWAETDKDLHSLMTAESFA*

>CYP6220A2|7916|Tubae1

MMIDGEYFPTGISSTEYLTAVICAFGALIAWKLVSAIFFSPLSHVPGPLVTAITPHYINFLSALNRRTVGTYSLHRRYGP
IVRVSPTEVSVLSPQAIKEVYSSPHYAKYAPLYSIFTHFGAQTAFTSCTREEHGWRRKAVSEAYSLSFVLKDEVATGKVL
QAVKDYLSFVESDRRVDIYNANTFYATDVVTGKIFGLEASMKTLAGNEAHRDIVLGHYARTRRTQVWMYIEFPLVMNVFE
WFAFYAGKVWSLVCGEKIEDWEMVSRIQRWGWDAYMDVKSNSREGTLAGRLARLAEDGGGAEGVWDDRGVASEAMDQMLA
GMDTTGDTLSFLMYQLSLPESRNVQKRLHSELLDAFPKPAEISGSSWPHSMDIQSLPHETILKVLNLPYLEAVLKETLRV
YTAIPITLPRVVPPSATTDRVSGHRNGRIIEGKFIPAGTTIGTFAYGIHRDEVFAVETGRKDNLGDVDNFLPERWLINGG
LEDKLTPDEMEQEKQRIRTMEARLWAFGSGARNCVGRHLSILEMKLLLSTIYSRYQTQVTPNSGVKITHNRWHQRRTFRD
VLPFSGVDGVVTFTPYE*

>CYP6220A1|7917|Tubae1

MIDSKHFLNGISNTGYLTAAICAFGVLIVWKLISAIFFSPLSHIPGPLLTAITPHYINFLSALNQRTIGTYSLHRRYGPI
VRVSPTEVSVLSPEAVREVYSSPHYAKYAPLYSMFGHFGAPSAFSSCTREEHGWRRKAVSQSYSLSFVLKDEVATGKVLQ
AVKDYLNFVESDRRVDIYNANTFYATDVVTGKIFGSEASMKTLAGNEAHREIVLGHYDRTGRVPTWMLLELPFITNVLEW
LAFYRRKAWSWVWGGEVKDREIVARIRKWSWDAFMDAKSNSREDTIAGRLAKLVREGGSAEGVWDDRGAIIAEGNDTPQS
GSFGYSTSYLTLLDYWVEGDDPIITLNAQKRLHSELLDAFPKSAEISGSGWPHSMDVQILPHETILKALNLPYLDAILKE
TLRIYTAVPIRLPRVVPPSAATNSTSSHRSGRIVEGKFIPAGTTIGAFAYGIHRDEVFAVETGEKDKPGDVDSFLPERWL
INGGVEGELTPDASAKEKQRIRAIEARLWAFSSGGRNCVGRHVSILGMKLLLVAIYSRYQTRVTPNSGVKVTHNRWNDRR
TFRDVLPFLGVDGVVTFTPYE*

>CYP567V1|8294|Tubae1

MINERDPHKHSEMKRKLSHGFSAKALLEQEDIVQGYVDKLIGQINVHGTRPEGDEMVKWYNFFTFDLIGDLAFGQSFGSL
NDGMSKPHFWVSLLLGNVRAIAWRSVARWFPVFEKLGVWTVPKRVMEMRIKHSEYSRKMIVQQVPPLEQTWAESANLLSK
TNEFEDYSQGFAFRTVRPRRPGHDYPRIIWPGQHNHVPSLFLRALPECLATAGSETTATFLSGTTYHLLKNPRVYNLLAE
EIRSAYNTYDEITDANASKLKYLSAVIDEGLRIYPPVGTGTYRESPGETVDGIYIPKGVELSTSPWSTCHSPENFHDHEV
FKPERWLDPACTDKKHASQPFSLGSRVCIGRNLALKEIRLALSKIFWVYEMELVNRGVDLNRDSTSFVLWDKPDLWVRFT
RRPGVRVPVLDSE*

>CYP617W1|8337|Tubae1

MSTKTLLMGCAASAILLSCRTRFGAITMFLLLASLALTCNLLYRLILWPKLLSPTRHLPGPTSASFLMGNFPIIHGVRSG

APQANWMRTIPNSGLLRYLDLFNIERVLPTTAEALKEVLHTKSYCFIKPPILTSDVAKILGRTGLLFVEGEEHKMQRKLL
LPAFSHAHIKALVPGFWSKGMEMTEKVAAVVRASRSSGGEGEEGVVVEMGRWCSLATLDIIGSCGFGYEFRALESASISG
SSDTEEKSGSELANAYNTIFNKGSPSWIAVVLSTIFPSSFVQYLALKRTREVARAAGTIRRVTARILAAKKSALATSPKD
SESNDILSVMLKSDTYTGPEGESSMRDQMMTFLAAGHETTATSMIWAIYTLSLPENRHIQSRLRAEIHAAFPSGVPATVT
YDQIANLKYLSYITSEVLRLHPPVGVTLRMAGEDTTLNGEFLPKGTSIVISPFAINRSVALWGADAEEFKPERWAGGADG
AAAVESNYGFLTFLAGPRGCIGHVFARAEFKCLLAAAVGRFEFGQDGKKELVVKAGVTAKPVGGIPVTVREVVWG*
>CYP548BE2|8758|Tubae1
MAIFKTPIQSLDHMAIAVDMAALLNLIKLLSTSFAVYISSLVLYRLFFHPLARIPGPFLAKITDWYTVYYAYRGDRHLAL
YKAHEKYGPVIRFAPNLISFNSASSLKAIYGHTPLSRSLQKSQFYAAFPAVKGVHNTHNAISKTEHGRKRRVLSAAFSEA
ALKSAEDLVLDNIDVFSGKVNDAFKRGVGVDMGDLFSWLTFDVMGDLCFGKSFGMLTEEPTRFVTNLVSQAAHNHYINGN
YLPLVTLKLSKVLFPTISRDRWRFIRHSRACADERVNLGKTYKKDFFYYLLDAKDPETGEGFGTKELWGEANVLMIAGSD
TTATAMSAAMFYLCRNPRVLETLKKEIRGTFTKKEQIVGGKELGDCHYLRACIDEAMRLAPPVPGLLPREVIAKEGIEID
GVYIPEGTVAGTPIYALHHNPAYFPDPFSFKPERWLPAFTSQEEVEAARSAFTPFSIGPRGCIGKSVAYMELRLTLARLL
FEYDCEEVETEGKAALWKEGYAMVDGEYRLMDHFTSRKEGPVMKFSRR*
>CYP6001C26|4741|Tubae1
MASNTNGTNGTTNGPSNGASGGVSKGKASKYATQAKPPPPVAKPTRAEVDSTFEQFSSLLHASNRPLPNRFGDGKDQRPP
EVKQTGVLTDINTLRRGGFFWESVGTLWDVLKGKIKGGPVDDKTMIMERVIQLTSRLPPTSKVRVALTTTQVGQLWDSLQ
HPPLSYLGDEFNYRQADGGYNNILCPQLGRAGSAYARSVKPMIKMPGAPPDAYTLFDRIFSRGPNDEHFREHDNNVSSML
FYTATIIIHDLFRTNRADPNISDTSSYLDLAPLYGNSVEDQSKIRTFQDGKIKPDSYCEKRLLAFPPGVSVFLIMFGRFH
NYVVENLKAINEGGRFSLKFPRYPNGDDEATRQANALRQQDEDLFQTGRLVTCGLYINFVLNDYLRTIVNLNRVDTTWTL
DPRFEASKVYNPDGTAAGVGNMVSAEFNLIYRWHSCISKRDDQWTKDFYNGLFPGRDTRDIEMPEFMRGVGRWEAGISDD
PLQRNIAGLQRKADGSYHDDDLVKILTESIEDVAGAFGARNVPHVLRLIEVLGIEQSRKWKVASLNEFREFFGLKPHATF
EDINPDPAVANTLRQLYDHPDFVEMYAGLVAEADKKPMVPGVGIGPTYTISRAILSDAVTLVRGDRFYTADYTAAHLTNW
GLQEASSDPAIVHGCVGYKLIIKAFPNHFKFNSIYAHYPLTIPEENHKIHTALKTVDQFDFERPVYTPLRIPISSYDATK
QILRDAENFKVTWGAGFDFIMRADFMLSGDKPSNSEQKQFVRQRLYLGDVDWKQQIRQFYQEVTEKLIRKKAYSLGDTYQ
VDAVRDIGNIAQTIFAASIFNLPMKSEDHPKGIYTEQELYTILCVMFIVIFFDIDSSKSFPLRQAGFKVVRQYGTLVEAQ
VKAIKNWSWLQGVWDPLNIRGRNKSSPLTSYGWNMIKRLLETGKSPYDVTWSYIVPTAGASAPNQGQIFAQVLDFYLEDR
NAHHLAEIQRLAQLGTEEAWETIKKYALEGGRLAGTFGLYRRVEPDNITIEDNGRDIDLRKGDIVFVNFITASRDPVVFP
DPLEVRLDRPEDSYMQYGDGPHQCLGKAANIIGLTTMLMQFGKLKGLRRAPGPQGTLKYIPKPGGFKVYMKEDWSAYWPF
PTTMKVRFDDII*
>CYP52AV5|4993|Tubae1
MELAFKALFLLAAAFYLLLRLATYLLTAHKNRIFAKSHNCLPPRRFPSPFLGLPNWVRLMRAAKRGNVLEHITDRYPTYG
NTWKGRILIGTSIGTVEPENIKAILATNFKDFGLGPQRHANFYPLLGDGIFTLDGPGWEHSRANLRPQFSREQVSDIEAL
EVHVQRLMNRLPEGDGEVADLQPLFYCLTLDSATEFLLGESVDSLLSPELNPTGDGGGEMSFAQAFNVSQGYLINRTRVR
GLYWMINPKRFRDANAIVHRLVDRYVDMALHPEKRAKKISEKKYVFLDAIAAETKDPKYLRDQTLNILLAGRDTTAGLLG
FIFWLLARHPHIYQKLREEIISAFGTGRDGGGKRPSFSALKDLTYLRYVLNETLRLYPSVPLNGRTAVRNTILPRGGGED
GLSPVFVPKGQRVDYSCYGLHRRKDLYGEDADTFRPERWGEGVGRGWEFLPFNGGPRICLGQQYALTEASYTVTRILQKY
ARIEVADTYTGPMMDLTLTVAPKKVLLRFWRA*
>CYP6001E1|6865|Tubae1
MSFLGLFGGGSSANPNAEYGDESAATQEITYTGIAEDIKASGGKIPEDLKLLLETGVQKASKGPVDDRKLVMERLIGLVA
SLPQNSANRKKLTSTIIDTLWDSLQHPPLSYVGDKYQYRQADGSYNNIMYPDLGKAGTEYARTVRQDKKLYGAKPDPGLL
FDLLMARGDNFKQNPAGISSVLFYHASIIIHDVFRTDRENFAKSETSSYLDLAPLYGSNQEEQDQIRTMQDGLIKPDTFS

EKRLLGLPPGICVLLVMYSRFHNYAAKTLKAINENGRFSLPRSHTTDTPEAQAKNLAKLDNDLFQTARLITNGLYVNISL
HDYIRGIANLNHSDSTWTLDPRVEIDKTFDGEGTPRGVGNQVSCEFNLLYRFHSGVSKRDDAWTKDFFGKIFPGQDPTTI
GVPQLLQGLKIFEKGIAEDPAKRTFGGLKRTGADGTGAFSDDDLVKILKESIEDPAGAFGANTVPEILKPVEVLGIIQAR
KWQVASLNEFRAFFDLKKHKTFEDINPDPYVANTLRKLYDHPDMVEMYPGMFLEDTKPRMDPGMGLCAPYTVTRAVFSDA
VTLVRSDRHLTLDYTPANLTNWGITEVAQDYDTLGGAKMFHLILNAFPSYFKYNSVYAMQPFYTPTESRKIFDKFEKSHL
YSFDPPAKTASPIPILTHAGLKRILNDQKNFKVPWGESMEALNNENDFMLAGDLSSHTAQRNLVGDAIYDVTGSRKQFKD
YTEEITLKLLKREVYQLGKVPFSQVDIVRDIGNLACLHFAASLLYLPLKSDENPNGQYSELELYKTLTDLTWFVFSDSDP
TKSWEHRREAKKSIDKLGEVMVGEIKKFKTPLGVWSKLTGAPGIRPPPPSLKDYGSNLVKRLLDSGKSVEDVAWMLLWSG
CAFVANSACAFAQLIDFYLQDDNRKHWAEIQRLSTLNTPEADKLLTKYTLEGTRLSNSLGIFRNVEPVDSQAITIKQLGQ
DVVLKKGDKVFVSFVYASKDASVFPDPLEIKLDRPTELYITHGEGQHQCLGKDINIIQNTYMLKTLAKLRNFRRAPGDEG
KLKFIPKAGGIKLYMNADWSKFTPYPTRLEKGVFKGIGNIEIGYLY*

Tuber borchii Tbo3840 v1.0

>CYP52AM1|1119975|Tubbor1
MELAVKFFLLLAILLYLLLKLTAYLLITYQNRIFAKSHNCLPPGRLPSSFVGLSNWVRLTRTAKRGDILDHILSHFPIHG
NTWKGRVLFSTSIGTIGPENIKAILATSFKDFGLGSQRHNNFYPLLGDGIFTLDGAGDVEALELHVQRLMNRLLEGDGEV
ADLQPLFYCLTLDSATDFLLGESVDSLLSPELNPTGAASGGKEEMSFAQSFNRFRDANAIVHGLIDRYVDMALHPEKRAK
KAPEGKYAFLDAIAAETRDPGYLRDQPRVMKEGETRMGRHRVLLLVHTPVFAD*
>CYP5959A15|1062621|Tubbor1
MGIFMRLIDYAEDKKLYSYSLFSATLALLFLGLFLFLKKEWSIHSSTRFPIAGIESPGYFGLVKARDRWVSDALHIVKNG
YHKYRGRNFVVTTHSFDKIVLTYDQIKELSNAPDDMVGAQDSGTETMLGSYTGLDRFLEHNYIQGVARIKLTQNLGSIKD
AIIEEARFALDAELPECTTDEWKSVQIFTTVLRTVTRTSSRVFVGLPLCRNEDWLTVAMGYTGDVFRTVDKLTSIPKPIR
PFYAWMFNSTKLIQSHRKRAETLLGPAIQRRLEEKRLAEKNGAIYEKPDDMLQWLEDLVQPHHKTTESLSELQLLASLAS
IHSTCNTFLNALFDLAAHQECVQPIREEIEAVISANNGVIDREALRKMRKTDSFFKESMRKNPHLFGFNRKILKSLTLSD
GTRLPKGALVAAPSGILSTDPDFIENPEAFDGFRWYKKSLEAEGRAARDASWTTTSAWDLGFGHGKHACPGRYFVTEEMK
IILTFIILQYDFKYPEGQSRPASLSHAEFFYPDTTRKLLFKKLPGPKKFSFL*
>CYP61A1|1063739|Tubbor1
MDEMANSSNFSPPSAVSSAEQPFQTLVGGLETIGKTLEGLNGWQIFFSIVILSVTYDQFRYIWNKGSIVGPALKIPFMGP
FLESVNPKFSEYLAKWNSGPLSCVSVFHKFVVIASTRDLARKVFNSPGYASPCVVDVAKKILRPDNWVFLDGRAHVEYRK
GLNGLFSRQAMGMYLPGQEELYDIYFKRWLELSKEGKPIPFMSEFRDINCAVSLRTFVGHYISDEAVKEISENYYKITAA
LELVNFPIILPWTKTWYGKKCADFVLNEFAACAAKSRIAMKEGKEPGCTMDSWIKSMLEAKESEASKIAAAKGLPQIREF
SDMEISMTVFTFLFASQDASSSATTWQFQIMADRPDVMKKIREEQLRVRDGDPYKKLDLDMVDKMVYTRAVVKEQLRYRP
PVIMVPYEVKKSFQITPEYKIPKGAMVVPTLYPSLHDPEVYNDPESFVPERWLEGGEAEAAKKNWLVFGAGPHVCLGQNY
AIMNFMSMIGKASLMMDWEHHTTPKSEDIKVFATIFPEDDCFLVFKERDPYAPAE*
>CYP5959A13|1077072|Tubbor1
MGIFMRLINYTEDKRLHDYSLVSATLALLFLALFLLLKKEWSIHSSTKFPIVGIESPGYFGLVKAREKYISDAFHIVKNG
YHKYRGKNFVVTAHSYDKIILTSDQINELSNAPDDTVSFSHFLAEVMQGPYTGMDRFLGTNYLAEVVRIKLTKSLGTIKE
AIIEEVRFSLDTELLGCTTDEWRSVEIFILILRTVARTSGRVFVGLPLCRNEDWLAVSMAFTGDLFMAIEKLERIPKPIR
PFYTWIFNSTKLISLHRKKAQTLLGPVIRERLEEERLAKENGSAYKKPSDMLQWLTDLVEPRHKNTKDLSELQLFASVAS
IHTTSLSFLNVLYDLAAHQECVQPIREEIEAVISANNGIIDRAALHKMTKTDSFFKESTRGQIDVLSFGRKILKNVTLSD

GTPLKQGTLVSAPAAMFSSDPDLLEDPETFDGFRWYKKSLEAESRAAHNSNWTTTSAHHLTFGHGKHACPGRFFVTEELK
ILLTFIILQYDIKYPEGESRPATIKYGEAIYPDTTRKLLFKKLPGPKKFSFL*
>CYP5959A12|1077074|Tubbor1
MGIFMRLIDYTEDKRLNDYSLFSATLALIFLALFLLLKKERSIHSSTKFPIAGIESPGYFGLVKAREKFISDAFHIVKSG
YHKYRGKNFVVTTHNHDRVILTSGQVKELSSAPDDTVSFSHFIMETMMGPYTGLGRFSGTNYLAEVSRIKLTRKLGIIKE
AVIEEARFALDTELPECTTDEWKSVEIFTTVLRTVARTSSRVFIGLPLCRNEDWLTVAMGFTGDLIITVNKLASTPKLIR
PFYAWMFNSTKLISSHRRKAQTLLGPIIQRRFEEERLAEENGTIYEKPDDMLQWFIDRVEPRHKNTEDLSELQLLSGLAS
VHATSLSFTNALYDLAAHQECVQPIREEIEAVISENNGVIDRAALRKMRKTDSFFKESARGQLNVLTFGRKILKNVTLSD
GTPLKQGTLVSAPVAMFSSDPDLLEDPETFDGFRWYKESLEAESRAAHNSGWTTTSAHYLTFGHGKHACPGRFFVTEEMK
ILLTFIILQYDIKYPEGQSRPANIQRGASLVPDPTQKLLFKKLPGPKKFSFL*
>CYP6470B1|1080969|Tubbor1
MSKLSEILRGQSYGVVFTPVNTVLAITAFFFLYYLKQRFFGPLRHLPGPWWSHYTQFPDTLHTFSGNRSLYIHSLHEKYG
DTLRLGPNTVGVTGQEGVKTIFGGGSKRPFHKEPVITEMLNFGRTFEETMAGTADPVSSSKRRMMYGNAYSRGSVLNMQD
IYQKCFMNFIARIEESRKNSQDGIIGVTPYFRAMAYDVLTEAAFGGLCKGTEYGTDAVGIMDNVMNANLFQWYFGTFIYN
LTMKIPWKKLDWIRSKEEYLQATQEVVSVYQSNVKAHPDRVRKDLLGNLILNNLDPKKLYHEAYGNIWAGTDTTTNTLGV
LMLAIVRHPRIYKNLVAELDRAFPPGEYVPFAPMELQKLETECPYLTCCIKECLRRYPLIPGPSPRIVPKEGTNINGWFV
PGNATIFAQTYTTNHNAKIFPNPKMYIPERWQAGKETAEMKAMMTSFGSGPRICAGENLAMMELHCATALLFRNYVVSIP
DGYDNDHVEFWITTPKGGSIFLRIEPREV*
>CYP6135A8|1085747|Tubbor1
MLAPIFLTTALLYIGYTCYSLHILLCKVRPLGIPYFVVPFYPSPFVRVFIFPLMKLLIKVSGLTHPRFFLMASDWQVRQR
YEIYRLIGSDIFFTVTPWKIILCVADPDMAVEVLAGKDENGELYPKPERVSKVMGLFGENVLTVEGAVWRGHRKIAGPVI
GKSSLSIVWEESLTQIKQMIQYYDKHEATNTSYRDFRRATLGVIAHAGFSKVMEWVPLTAAIAKEKDSEGSYQKYLHVLF
ENFKWTTIAPSWILEMLPFKALREAGGAANGFRFFMNEWFEEKKAKIELDNKTALKPFREDLMESLVRSSGLDKGSTLET
PLLSKSEVIGNAFIFILAGHETTAHTIANAIYFLAMYPEYQVKLQEEIDSILGDSDHSLTSYETHFDAFSSGWIAAIMFE
TLRLIPAVIITTRHMGPSPRTFPRAGGQRPITLPPGIESMIQIIGLAHNPKYWTQPGKTEAESAISEFRPERWLPKSGDN
IMFKPYNGSYIPFSIGTRGCIGKKFAHVEFTAIMIGLFREMSVEFDSCGGRKTFEEARLECLAQMEKFESKITLQPSGEM
PSIKWVRRRR*
>CYP6135A10|1085752|Tubbor1
MLAPLFLTIGLLYIGYACYSLHTLLCKVRPLGIPYFVVPFYRSPFVLVFIFPFMKLLIKAFGLTHPRFFLMAPDWQVRQR
YEIYRFIGSDIFFTVSPWKIILHVADPDMAVEILAGKGGDGELCPKPSGGGMVDLFGENVVTAEGEVWRSHRKIAAPVIG
KSSLSIVWKEALTQIEQMIQYYDRHEAINTSYRDFRRVTLGVISHAGFSKVMEWVPLTTAIANEKDGEGSYQKCLHVLFE
NFKWTAIAPSWLLEILPFKALREAGWAVNGFRLFINEWFEEKKAKVELDNKTALKPPKADLMGILITSNNAIWLGNLLTR
NAFVQAHPLSLQHLANSLQIFILAGHETTGHTIASAIYLLAMYPEYQVKLQKEIDSILGDSDHRLTSYETHFDAFSSGWI
AAILFETLRLFPAVTVTGRQIGPTPRTFPRAGCQRPITLPSGIDFMIQILGLSHNPKYWTQPGKTEAESAISEFRPERWL
PKSGDSTMFKPYNGSYIPFSIGTRGCLGKKFAHVEFTAIMIGLFRDMSVEFDSCGGRKTFEGARLECLEQMKKFELKLSL
QPTGEMPGIKWVRRRR*
>CYP6135A4|1085755|Tubbor1
MLASLFLTTALLFMGYAGYSLHTLLYKVRPLGIPYFVIPFYPSPFSRIFLIPFVNLLIKVFGLTHPRFFLMTLDWQVRQR
YEMYRFIRSDIFFTVTPWKVILHVADPDMAVEVLAGKGGNGELYTRPDVYNSMLGLFGENVLTEEGAVWRDHRKITAPVI
GKSLSVVWEESLTQIQQMIRYYDKHEAINTSYRDFRRATLGVIARAGFSKVMEWCPLATAIANEKDDEGSYQKCLHVLFE
NFMWAVIAPSWILEMLPFKALREAGGAANNFRFFVNEWFEEKKTKIELDNKTTLGPSSKADLMVPLLSKSEVIGNAFAQA
HPLSLQHLANSPQIFILAGHETSAHTIANAIYLLAMYPEYQVKLQEEIDSILGDSDHNQTSYKTHFDAFSSGWIAAILFE

TLRLIPELTVMARQIGPTPRTFPRAGDLRPITLPPGIEFWIHVIGLSHNPKYWTQPGKTEAESAISEFRPERWLPKSGDS

TMLKPYNGSYIPFSIGARGCIGKKFAHVEFTAIMLGLFREMSVEFDSCGGRKTFDEARLECLAEMENFESKITLQPSGEM

PSIKWVRRRR*

>CYP51070A7|1086989|Tubbor1

MELLKENLKLLQAVSSILFVLIIYCSSKKKSNAPLVGVEPGILGYLKAPYRWKQDPMSLLKEGAEKYGKYGKAFRITTPS

RDMIVFSNQEVLEELKTLPASVATFRPAGDTMIRSEYNFHEGIIQDAYHVDLMRKNMTEKLSSLLPDVVGEVTDAFEELT

DVKNEWTNVNVLDTSVGVIARVTSRIFVGLPTCRNQEYLNHMIQFSLNVMKSATVIDVLPWFLRWIVASFFLKKKDSQDI

IVKHVGGLFETRQKTEMPHTSSATTTSDAIQWILDAAPPGTPTLKLVQRLMVFNFASVHTSSITLAHALYDLAANPEFQD

PLCIEIEEGLRTEGGWTKQALTKMKKLDSFLRESSRMNGVNIVTVVRKVITSHTFIDGTYAPKGVWVCAPAATLHLSSEL

YENPEIFDGFRFYRMRQLEGNAHNYQMASPSLGYLPFGIGKFACPGRFFATNELKIAVAYILCNYRLRLNGGVCGKKPPN

LFSGIVCMPNPTVGIELKKREGREKSVLFPGHVTSYSL*

>CYP51F1|1090672|Tubbor1

MGVLSTLLVPMGPYYEELMELGGLALCGIGFVAFVVLSVILNVLQQLLFKDPTKPPLVFHYFPFFGSTVVYGMDPYKFFS

DCREKYGDTFTFVMLGRKMTATLGPKGNDFVFNGRLSEVSAEDAYAHLTVPVFGEGVVYDVPNHVLMEQKKFMKFGLTVE

NFRSYVPLIVEQVEDYIKRSKFFKGANGSVPLSDIIPELTIFTAARTLQGKEIRDALDGSFAKLYHHLDSGFTPMNFLFP

WFPFPQNKRRDHAQRTMAQFYMDKITKRRANEKNDEDQERSDMMWNLMNCSYKDGRKVPDKEVAHMMIALLMAGQHTSMA

TITWMLLHVAAQPKLVARILAEQKRVFGDELAPLSYEKLVLECTFLGHIIRETLRIHPPLHSILRKVKSPMHIDGTNWVV

PKGHYLLAAPGVSSMDEKYFKNPLAFDPSRWEGQKAEEEAEKFDFGFGLISKGTASPYLPFGAGRHRCIGEQFANVQLMT

IMATFVRNFEIQRPGGGSDVPAPDYSSMIALPAPPSIIEWVKRDP*

>CYP567Y3|1094055|Tubbor1

MSQVHVGYLHTMALHIWGIVGFSAILGAVFVLGRAFYNVFFHPLSKIPGPKLCAATDFIVAYHFLSGRYPFYIKSLHDKY

GTVVRIGPNQLSFSSASSWQDIYGHSYRRKLFPKSTFYSYGARHLGNELDPQRHSEMKRKLSHGFSVKALSEQEDIVHNY

LDKFIRQINVYAAGPKGDEMVKWYNFFTFDLIGDLAFGEPFGSLNDAKPHFWVSLLLGNVKAIAWRSVSRWFPVFDKLGV

WTAPKSVMKMRDEHMEYSRKKIIQRMNTKTTRRDLLSGQFGPNGPGMTIPELSAQASLFITAGSETTATFLSGLTYHLLK

NPRVYNLLVEEIRSAYNTYEDITDIKASQLKYLSAVMDEGFRMYPPIGVGLYRLSPGETVDGMYIPKGVELSTNPWSTCR

SPENFHNPDEFKPERWLDPDCTDKKHASQPFGLGSRVCIGRNLATKETRLVLSKMLWVYDMELVNKDIDLDRDSTNYFLW

SKPEIWVKFARRRGVQVPILDSE*

>CYP567Y3|1101102|Tubbor1

MVVNIWEIVGFSAILGAVFVLGRAFYNVFFHPLSKIPGPKLCAATDFIVAYHFLSGRYPFYIKSLHDKYGTVVRIGPNQL

SFSSASSWQDIYGHSYRRKQFLKSNFYAYHPRHLGNERDPQKHSEMKRKLSHGFSVKALSEQEDIVQNYLDRFIRQINVY

AAGPKGDEMVKWYNFFTFDLIGDLAFGESFGSLNDAKPHFWVSLLLGNIKALAWRSVSRWFPVFDKLGVWTAPKRVMKMR

TEHMEYSRKKIVQRMNMKTTRRDLLSGQFGPNGPGMAIPELSGQASLFITAGSETTATFLSGTTYHLLKNPRIYNLLVEE

IRSAYNTYEEITGTKASQLKYLSAVMDEGFRLYPPFAVGLYRESPGETVDGIFIPKGVELSTSPWSACRSPENFHNPDEF

KPERWLDPDCTDKKHASQPFSLGSRVCIGRNLALKEIRLVLSKMLWVYDMELVNKDLDLDRDSTTYFLWIKPEIWVRFTR

RQGVQVPILDSE*

>CYP6136A1|1101634|Tubbor1

MFLNLFGQAVYSLLQHKLYFFSGILILTLAYWTHAYLTSPFRAQKIPGPFLGKFTNAYRWYYVMRHTWHRDLVDLHKKYG

PIVWIAPNDISVSDPTLRNVIYGFQNHKKEFTFFKKSPSYETGSINQDFSFIFEQDPEKARLGKYHMSHFYSEQGLLNLE

ENFDKAVDELIEGLDKHHAKTGTPCKMVDWAEFFALDLVAQLTTDRSAGFCLAGTDINNAAYGMRVIIKTVGALMPLPWV

LSATSRAIRQTLLIKFLINLYRNVLLLPTFTFDSGTADLEFLKEKHPNHLLAKFYNAQSKMREHYPHGNQAEGTTIQLFN

LIAGAVGVVPHSQVKLIQELALNPEVLQKVREELATTNNSFHIKDFLRYNNRQNKYPIFESAVREAVRPPAGCQLHQYHI

PPGYNVGMAAYQVNYDEGYFGPDVAQFRPGRWLEDHPTEMLEGEKRSMKNYIEAGWLSFGSGGRVCIGRHLAMFMMMKFT

AAVVREFDIRVVKQPEEHFTLVTEMLGMEVLLSRKCSV*

>CYP567U3|1101678|Tubbor1

MAILHQLLGAVKDVPAWKLCVYVTLLFPLYAITMGIYNTFFHPLRNFPGPKRAALSNIWYSHAWLSGKYPHKMHALHEKY

GSVVRVAPNQLSFSTSSSWKDIYTNHNNRPTFRKTGFYDADKLDPETNIVREPDPAKHSQIRNMFSQSFSPKSLLEQEPI

VQGYVDLLIEQIGKYGTGKDGLEIVRWYNYCTFDIVGDLAFGEGFNAVKDGKPHFWISIILDSVYAATFIYVTKRFPWLA

KLIPLLIPAHMVEHRIRHLNYGRDKIKRRINSDNKRKDFLTNVLDNHRDKISDEALSSNAQIFVIGGSETTATTLSALTY

FLLRNPHTYQRLVSEITSSFTSYSQITSTAAGKLEYLGAVITETLRVYPPLPVGLPRTSPGETVDGYYIPKGVAVSTSSW

AAVHDPKNFHEPWAFKPERWLDGECKEKDVREASQPFSLGPRACLGRNLAIIELRLIVCKMLYTYHLELLDSKLDWVRDS

TAYLLWIKPGLRVRLYRR*

>CYP548BE2|1101996|Tubbor1

MITFLNLIKLLATSFAVHISSLVLYRLFFHPLARIPGPFLAKITDWYTVYYAYRGDRHLALYRAHEKYGPVIRFAPNLIS

FNSASSLKTIYGHTPLSRSLQKSQFYSAFPAVKGVHNTHNAISKAEHGRKRRVLSAAFSDTALKSVEDLVLNNINVFSGK

VNDAVKRGVGVDMGDLFSWLTFDVMGELCFGKSFGMLTEETTRFVTDLISQAAHNHYINGNYLPLVTLKLSKVLFPTISR

DRWRFIQHSRACANERMSLGRGYKKDFFYYLLDAKDPETGEGFETKELWGEANVLMIAGSDTTATAMSAAMFYLCRNPQT

LETLKNEIRSTFANKEQIVVGKELSDCRYLKACIDEAMRLAPPVPGLLPREVTAKEGIEIDGVYIPEGTVAGTPIYALHH

NPAYFPDPFSFKPERWLSTCTPEEEVEAAQSAFTPFSIGPRGCIGKSVAYMELRLTLARLLFEYDCEEVETEGKAALWTE

GYAMVDGEYRLMDHFTSRKEGPVMKFSKR*

>CYP6592A5|1107457|Tubbor1

MNVATKAPILRGKRTTLSPIDTSLLVLAPFAVVVYNLYYHPFSPFPGLFMASTGSSWLTWEYDSDLEDLEPLLHEEYGPI

VRISPNIISVNDPNYVKEISIQANPPHWWLPFGHEGVNDVFFVPPPGLHAEWRKCSAPTCSVGMVGLWEGVVDCRVREWA

EKLKAKYGSSEAHLDLSEWARHLSVDLVGLVVFGVDMGCTQNREDNKGRTPAFESELENVGVLAELRTIYRTLTWNSFIP

NISGEKADREGPEALRVKAGLIIDTAWMSREKSEAGGEEWHLNSEYATQAVTMLEKAMSAKNSEGTRFTKEEVLTKCFPP

FLASKDSIAFAITNTLKLLFTNPRVLATLLSELATYYWQKPLSEIPPWTDIEDQGTRLPYLSAVLHESLRFNPAFLMSFP

RIVTSSGTELTHRNCLYTLPGGLQIYLNPIVICRNRRIFGEDVHHFCPERWLEGTREKIERMKTVGFGWGAGSSDCFGKA

LAQMIVAKAVVTVLRNFEVELPVGYSSGDVWNLGAMGGSSAAEFWAKVKSKEWPGAEGEMGGPSEHL*

>CYP6135A7|1108740|Tubbor1

MLAPLFVTTALLYIGYTCYSLHTLLCKVRPLGIPYFVVPFYPSPFIRVFIFPFMKLLIKVLGLTHPRFFLMTLDWQVRQR

YEIYRFIGSDIFFTVTPWQIILHVADPDMAVEVLAGKDENGELYPKSERVSQVMGLFGENVLTVEGAAWRGHRKIAGPVI

GKSSLSIVWEESLTQIQQMIQYYDKHEATNTSYRDFRRAALGVIAHAGFSKVMEWVPLTTPIANEKDREGSYQKHLHVLF

ENFMWAAIAPSWILEMLPFKALREAGGAANGFRFFMNEWFEEKKAKIELDNKTALNPSREDLMESLVRSSGLDKGSTLET

PLLSKSEVIGNAFIFILAGHETTAHTIANAIYFLAMYPEYQVKLQEEIDSILGDSDHSLTSYEAHFDAFSSGWIAAIMFE

TLRLIPAAVITTRHMGPTTRTFPRTGSQRPVTLPPGIEFWIQIIGLSHNPKYWTQPGKTEAESAISEFRPERWLPKSGDN

VMFKPYNGSYVPFSIGTRGCIGKKFAHVEFTAIMLGLFREMSVGFDSRGGRKTFEEARLECLAEMEKFESKITLQPTGEM

PGIKWVRRRR*

>CYP51070A6|1108898|Tubbor1

MEFVKENLRLLQAVSSILFVLIIYYSSKKKSNAPLVGVEPGILGYLKAPYRWKQDPMSLLKEGAEKYGKYGKAFRITTPS

RDMIVFSNQEVLEELKNLPASVATFRPAGDTMIRSEYNFHEGMVQDAYHVDLMRKNMTQKLSSLLPDVVSEVTDAFEELT

DVKNEWTNVNILDISVGVISRVTSRIFVGLPTCRNQEYLNHMIQFSLNVMKSATVIDVLPWFLRWIVASFFLKKKDSQDI

MVKHIGGLFETRQRTKTPHTSSTTTTSDAIQWILDAAPPGTPTLKLVQRLMLFNFASVHTSSITLAHALYDLAANPEFQD

PLCIEIEEALRTEGGWTKQALTKMKKLDSILRESSRMNGVNIITVVRKIITSHTFIDGTYVPKGVWVCAPAATLHLSPEL

YENPEIFDGFRFYRMRQLEGNAHNYQMASPSLDYLPFGIGNFSCPGRFFATNELKIAVAYILCNYQLRLNGGVCGKKPPN

VFSGIVCMPNPTEGIELKERQDREKSVLFPGHVTSCSL*

>CYP6135A14|1118736|Tubbor1

MNTKPQILATEIFAEQHSELSLMLGLIRLWSGSLTTAIGNGKDGEGSYQMCLHVLCENFIWTVIAPSWLLEMLPFKALRE

AGGAASNFRFFMNEWFEERKAKIELDNKTALKPPSTADLMESLVRSSGLDKGSTLETPLLWKSEVIGNAFIFILAGHDTT

GHTIANAIYFLAMHPEYQVKLQEEIDSILGDGDHHQTSYETHFDTFSSGWRETIVRNSPPHSCSDHSKPPNRFHPPHLPS

HRQSGTHHTPSRYQILDPNNRPSHNPKYWTQLEKTEAESGISKFRPERWLPKLGDNAMFKPYNESYIPFSIGARGVYWEE

DAYIEFTAIILGLFREMSVEFDSYRGRKAFDEAKLECLAQMSKFKMKLSLQPTGEMPGIKWVRRR*

>CYP6135A9|1126296|Tubbor1

MLAPLFVTSVLLFMGYTCYSLHILLCKVRPLGIPYFVIPFYHSPLQRLFLFPLVKLLIKVFGLTHPRFFLMTLDWQVRQR

YEIYRLIGSDIFFTVTPWKIILHVADPDMAIEVLAGKGENGELYSRPDFFSGIALFGENLATVEGAVWRGHRKVTGPVIG

KSLSIVWEESLTQIEQMIRYYDKSEAINTSYRDFRRATLGIMTRAGFSKVMRWAPLTAAIADEKDGEGNYQKCLDILFGN

LMWPIIAPSWLLEMLPFKALREAGRAANHFRFFMNEWFEEKKAQIELDNKTALKHSSRADLMESLVRSSGLDKGSTLETP

LLSKSEVIGNAFIFILAGHESTAHTIAHAIYFLAIYPEYQVKLQKEIDSILRDSDHNKTSYDTHYDAFSSGWIAAILFET

LRLIPAVIGVSRKIGPTPRTFPRSGGQRPITLPPGVEIWIEFIGLAQNPKYWTQPGKTEAESAISEFRPERWLPKPGGDT

MFKPYNGSYIPFSIGTRGCIGKKFALVEFTAIMLGLFREMSVEFDSCGGRKTFDEARQECLAEMDRFKSKITLQPSGEMP

GIKWVRRKR*

>CYP6135A3|1128001|Tubbor1

MLAPIFLTTALLFMGHTCYSLHTQLCKVRSLGIPYFVIPFCPSPILRVFLIPFVNLLIKVFGLTHPQFFLMTLDWQVRQR

YEIYRLIGSDMFFTVSPWKVVLHVADPDMAVEVLAGKGGNGELYTRPDVYNTMLGLFGENVLTVEGAVWRDHRKITAPVI

GKSLSVVWEESLTQIEQMIQYYDKLEAINTSYRDFRRVTLGVIARAGFSKVMEWCPLTTAIANEKDGEGSYQKCLHVLFE

NLIWAVIAPSWILEMLPFKALREAGGAANNFRFFMNEWFEERKTKIELDNKTTLGPSSKADLMESLVRSSGLDKDSTLEA

PLLSKSEVIGNAFIFILAGHETSAHTIANAIYLLAMHPEYQVKLQEEIDSILGDSDHNQTSYEAHFDAFSSGWMAAILFE

ALRLIPELTVMARQIGPTPRTFSRAGDLRPITLPPGIEFWIHVIGLSHNPKYWTQPGKTEAESAISEFRPERWLPKPGDN

IMFKPYNGSYIPFSIGARGCIGKKFAHVEFTAIMLGLFRDMSVEFDSCGGKKTFEEARLECSVQMENFESKITLQPTGEM

PGIKWVRRRR*

>CYP5959A18|1166028|Tubbor1

MLIIYTVTEWRSIEIFTPIRRMVAHTSSRVFVGLPLCRNEDWLTISMRYTSEFLMTVDKLRSIPKLIRPFYAWMFNSTKL

IKSNRKKAQTLLGPIIQRRLEEERLAKEIGSVYKKPNDMLQWLTDLIEPRHKTIEDLSELQLLAILASIHTSLSFLNALC

NLAANQECVQPIREEIEAVISANNGVVDSRKILKSVTLSDGTRLPQGTRVSAPSAMFSSDPDLLEDPETFDGFRWYKKSL

EAEGSAVYNTNLATTSTRDLGLGHGKHACPGRFFATEEMKILLIFIILQYDFLVSKTDAPKVTASIGKYQTATGTSAGT*

>CYP5945C2|1166238|Tubbor1

MISTLEKLNGKETNCVNIYDNMAFDIMADLGFGQKPEESMQSGAGDPSYMKFLHGWMRASNVLGSLRNLCEIGAYIPQDA

ESEGFNRLGEKMLEARQKMGKHRPDIFTHLLGEDGESGLKFNHAQLLINAQMLMVAGSDTTSVTMTCLFRLLSMHMEKQQ

KLRKEIADAFPNGETPTCAATAALPFLNGVVQEALRLWPAVPTGPQATTPPSGYTIAGTFVPGNVDIRIPPMALLSDARY

FPRPDEFLPERWTPEMPELVKDKRAFIPFGYGAHSCIGRPLALNEMRTAVARVIQRFEVQLGESYDDKVFRDRWKDYFLV

QLGPCPLKFVPREI*

>CYP6136A3|1195697|Tubbor1

MFLNLFGQALYGFVQHKLYLVTGIIILTLAYWIHSYLKSPFRAQKIPGPFLGKFTNAYRWYYTMRHTWHRDLMALHKKYG

HIVWIAPNEISVSDPNLRNVIYGFQNHKRDFTFFKKARSYEIGSINHDFSFVFEQDAGNARLGKYHMSHFYSEQGLLDLE

ENFDKAVDELIEGLDKHHVKTGTPCKMVDWTEFFALDIVAQITADQSAGFCLAGKDADNTAYGVRVILRAIGILMQLPWV

LSVTSRTIRGNIFLNYLTTLYRNLLLLPTFTFETGTADLKTLKEKSPKHLLSKFYNTQSKMRGHYPQGNEAEGTTIHIFN

LIAGALGVVPHTQVRVIHELSRHPEYMQKIREELSKTDNTFRLDDFLPYNGGENKYPIFESAVRETIRLHPAVSFSLSRE

VPPSGCQLHQYHIPPGYNVGMASYQVNYDEAYFGPDVAQFRPERWLEDHPTAMLDGEKRSMKNYIEAGWFTFGAGGRVCI

GRHLAMFMMMKFTAAIIREFDIRVVKEPEEVHTLFNEMPGMEVLLSRRCAV*

>CYP6498A1|1196312|Tubbor1

MGTLLILVVCFGAAIAAYTISSIIHLLYNIREVRKSGLPYIILPWHEMNLFHVLTCGFNRDLWQYLPQWTYFKIFWRDWC

HHTKFELFEKYGDVICAVSPGGVTIYVGSVEVARQMYERRNDFPKATKVYEYVRFYGDNVLTLEGAEWRRHSRYTHPPFN

EGVHKVVWDEGVKQARAAVNVWSRQNSCLNIGRDLRTISMNVLSLSNFGVAMPFDYESETTEHFGWRNIPPGHKMSYGST

VNHVLDNIIPLAIAPKWLLRNGPESLKRIGQSYDELGIYLKELTQDDGKSGASDGGNLLRALVKASAGDGPGKGSRLTDA

EVFGNAFIFAVAGLETTTGTLHYAIMYLALNPDVQDWLYTDLQEALKDEDQDPSKWEYGKVYPKMASVLCVIHETLRLNA

PHMHIPKWTAGKYQPVNWKGKQCLIPPGASAYITTTALHYNPSLWGDTVNGFHPQKWDLRSSSEGWVRTDPKTGVKTPIT

PSETPRIGTQSAPYCYLRTPVKGAFAPFSDGWRACVGKNFALVEMAAVLAVLFRDCSVRIKRQEGETQGMADNRGKSAIS

NSKSYLTVMIRHDVELEWIRR*

>CYP567Y3|1196592|Tubbor1

MSQVHVGYLYTMALHIWGIVGFSAILGAVFVLGRTFYNVFFHPLSKIPGPKLCAATDFIVAYHFLSGRYPFYIKSLHDKY

GTVVRIGPNQLSFSSASSWHDIYGHSYHRKQFPKSHFYSFGTRHLINERDPQKHSEMKRKLSHGFSVRALSEQEDIVHNY

LDKFIRQINVYATGPRGDEMVKWYNFFTFDLIGDLAFGESFGSLNDAKPHFWVSLLLGNVKAIAWRSVSRWFPVFDKLGV

WTAPKSVMKMRIEHTEYSRKKIIQRMNTKTTRRDLLSGQFGPNGPGMTIPELSGQASTIITAGSETTATFLSGTTYHLLR

NPRVYNLLIEEIRSAYNTYEDIMDTNTSKLRYLSAVIEEGFRIYPPIGVGTYRESPGETVDGIYIPKGVELSTSPWSACR

SPENFHNPDEFKPERWLDPDCTDKKHASQPFSLGSRGCIGRNLALKEIRLVLSKMLWVYDMELVNKDLDLDRDSTSYVLW

TKPEIWVRFARRRGAQVPILDSE*

>CYP5959A6|1197474|Tubbor1

MGIFMRLIDYTEDEKLHGYSLFPTTLVLLFLGIFLLVRKEWSVHSSTKLPIVGIELPGYFGLVKARLKFVSNGFHIVKNG

YYKYRGRNFVVTTHGYDKIILTHNQVKELSSASDDIISSQHALTETMMGPYTGLDRFVGTGYLEAAVRVKLTQNLGTIKE

AILEETRFALGAEMPEFTTDEWRSVEIFAPILRVVARMSSRVFVGLPLCRNEDWLTVSMTFTGDVFLTLAKLTSIPKPIR

PFYAWITSSTKLINSHRKKAQILLGPVIQGRLEEERLAEKNGTVYKKPNDMIQWFIDLVEPHHKTTESLSELQLLAILAS

IHTTSLSFLNTLFDLAAHPECVQPIREEIESVISENNGVLDRATMRKMKKTDSFFKEAMRARIGLFSFNRKVLKSVTLSD

GTCLPKGALIAAPTSMFSTDPEIVEDPETFDGFRWYKKSLEADGSRSATSNINWATTSSKDLTFGHGKHACPGRYFATEE

MKMILTFIILQYDVKYPEGQSRPASIDHGEFSYPDTTKQLLFKKLPGPKKFSFL*

>CYP5093H2|846442|Tubbor1

MIASAPQMPQSSPFTSSVEAPLASLVTSLHLSNGLKFVRQYEGTVIVPKDPVARFHSLSCLAACALLSALAISCLLVALQ

DYRRHAIMPPGPSPVPFVGNKWDLPLQKPWYKFKQWTDMYGSLVTVWTGRRPTIVIGDPKVACDLLDRRSVIYSSRPRFV

VMGELFTNNDSLLTMPHGDKWRKTRKIFHMGLHRRACESYKPIQEAESQRLTRDLLVTPEIFGKHLERYASSIMICVAYG

RRVDDLEDPIVKKIYDRMAYMSTLNVPGAFWAESFPILKLIPDCLAPWKREVKRRAKDSTEMLSRLALDVRDQIKKGDAP

ASFTKTLWEKREGNPEALSEREIAYATGSLFGAGSDTSSATLMSFFLAMTCFPRVAAEAQEELDRVVGRDRSPTWSDEPN

LPYCRAVIKETLRWRPVAVMGGTPHASIKDDQYNGHFIPKGTTILGNLWAIHHNEKYFKDSHDFIPERYLGSGKVGGMEP

YPHRDGHSAFGWGRRICPGKQLAENSLFITIARVLWAFNISKATDKHGQEITPNIFAYTDGFNSKPQPFQCRIQPRTPGI

QQVIEREARLGEQFLDKYKCS*

>CYP5959A16|971595|Tubbor1

MGIFMLLIDYAEDKKFHDYPLIPTTLALLSLALFLFLKKERSIHSPTKFPIAGIESSGYFGLVKVRGRFVSDALQIVKNG

YYKYRGKNFVVTTYTFDRVILTHDQVKEISNAPEDIVSARAATNETLMGSYTGLDRFPPTNYIQDAVRIKLTQNLGTIKD

AMIEEMRSALDTELPECTTDEWRSVEIFPPLRRTIARTSSRVFVGLPLCRNEDWLDISMKFTRDFFMTIHILSVIPKLLR

PLYAWMFNSGKVLTSYKKRAGILLAPTIQRRLEEEKLAEQNGAVYEKPNDMLQWLTDLAEPHHKNIESLSEMQLLTILAS

IHTTTNFLLNTLYDLAAHREYIQPIREEMEAVISANEGVLDKVALRKMRKTDSFFKESMRKNTFLINFNRMIMKNLTLSD

GTRLPKGALVSAPILMFSTDPDFVEDPETFDGFRWYKKSLEAEGLATHNTNWTTTSSNNLIWGHGKHACPGRFFAGEMMK

TLLTFIILQYDFKYPEGQSRPDGFRRAEFTIPDITRKLLFKKLPGPKKFSFL*

>CYP5959A17|971618|Tubbor1

MGILMRLTDCAEDKKLHDYSFLSASLVLLFLGLFLLLKKEWSIHSSTKFPIAGIESPGYFGLVKARARFVSNAFHIVKSG

YHKYRGKNFVVTTYNYDIVILTHDQVKELSNEPEDTVSNQASGIERMMAEHTGLGRFDGHNYLSEAVRIKLIQSLGTITD

AITEEIRFALDTELPECTADEWRPIGIFTTIQRTVARTSSRVFAGLPLCRNEDWLTISTRFTRDLFMTVIELTRIPKPIR

PFYAWMFDSKRVMNSYRKRAEILLAPTIQRRLEEERLAEKNGAVYEKPNDMLQWLTDLVEPRHKTTDSLSELQLMTTFVS

IQGTSNTFLNALFDLAAHQECVQPIREEMEAAISANNGVIDRATLRKMRKTDSFFKESMRKNPPLFTFNRKIMKSITLSD

GTCLPKGAMISAPVSMFSTDPDFVEDPETFDGFRWYKKSLEAEGLATHNTKWTTTSARDLGFGHGRHACPGRFFASDVMK

TLLAFIILQYDFKYPEGQSRPPTLETGEYFSPNTTKKLLFKKLPGPKKFSFL*

>CYP5959A14|971693|Tubbor1

MGIFLRLIDYAEGKSLHGYPLFSAALALLFLGIFLLLKKEWSIHGSENFPIVGIEFPGYFGLLKARARFVSDAFHIVKDG

YHKYKGKNFLVTTHGYDKIILTYNQVQELSSAPDDAVSIQHAVVETMMGQYTGMDRFVGTTYVQEAARIKLTQNLGTIKD

AMIEEIRFALDTELPECTTDEWRSIEIFTTILRTVARTSSRAFVGLPLCRNEDWLTVSMKFTGDIFMTVDKLSSIPKLIR

PFYAWIFNSARLINSHRKKAQTLLGPIIQGRFEEERLAKENGTIYEKPDDMLQWLTNLVEPHHKNIEDLSELQLLSNLAS

IHTTSLSFLNALYDLAAHQECVQPIREEIEAVLSANNGVIDKAALRQMRKTDSFFKESTRETLGLFSFNRKILKSFTLSD

GTRLPEGALIAAPVAMFATDPDLLEDPETFDGFRWYKKSLEAEGRATHNANWTATSAKDLAFGHGKHACPGRFFVAEEMK

ILLIFIILQYDIKYPEGQSRPASLKHGEFFFPDTAKKLLFKKLPGPKKFSFL*

>CYP530G1|973143|Tubbor1

MAIPIFWTTAPGLALGVCLASFVATIIYFTKGTSRGKNFPPGPPTLPILGNMHLVPQERPYLKFTEWAKQYGGIYSIKIA

KQTIVLISDVKILKELYDKRGAIYSSRPLPHIGAEIVCPDQTHIIYMPYGETSRNYRAQYHQFMGPGKVEQILPWQSAES

TLLLKKIATSPDRYYEHTMRMATAVILESVFGVLPKDYDDPEVTELWTVQREFSEILALTGPPVDHFPFLKWLPDIVSPW

RIHARSVRAMHRKLYFRLLNLNKARMEKGERYGTFVEKLIDDKPKHGLSDERIAYVCATLMEAGSDTTASQALDFMMALL

AFPDVLKKAQEEVDRVCGTSRLPTLDDRDQMPYIEACVNEVLRWRPPLPYGAPHLLMKDDWYEGYFMPKGTVLFQVQWAM

NMDENVYEKPQDFMPERYIRNRFGTKFNAEKDAETGRKEQYGFGLGRKICPGQWFARNTLFVLFAKLVWAFDMKVPTDPK

TGKPVPLDTDVRTAFMDGLTTTPFKFPIEFKIRSKAHEEALNTDLVASDKIFAKYGGATV*

>CYP50043A4|981437|Tubbor1

MGSLNFGLVRSAVYLLGAWVAYLALLVFYRLYLHPLRRFPGPKLAAATGWYAGYWDLHMGGQMVKHLADLHKEYGPIVRS

EPNQLHFSSPEAYSTIYSPTSKLTKDPALYHSFGASESVFTFLDPTTVRARRKVIGPMFSRRAVLSLQPLINGKIRKLCD

ILSGYADRDEAADIASGFHCAAIDIISQYCYSECLDSLDVEGFKHGVLVYAKATSETFWTIKYFPLVEWLLSLPRSVSLR

LVPELKGFLAFRDTIEEQVNRYTKNPSLLEKSSHTSVYQRFLDPQVKGGTPSFSSLVDEAQNLFFAGSDTVSGALTFGTY

HILATPGLQEKLFAEICRMWPVLGEEPTYEQLEKSMYLTAIIKESLRLSHGTVSPLPRIVPASGMTIQDQPIPGGTVVSM

DAPTIHLNPTIFPSPDTFLPARWLDSGAKDLDKHLVAFSRGPRSCIGSNLAWAELYIAFAAVFRRFDMVLWETSKEDMEW

MDCFVPHTKGDLKVKFKVRKE*

>CYP51070A8|1027190|Tubbor1

MSLLKEGAEKYGKYGKAFRIKTPSRDMIVFSSREVLEELKNLPASVVNFRPAGDTMIRTEYNFHEGMVQDAYHVDLMRKN

MTQKLSSLLPDVVSEVTDAFEKLTDVKNEWTNVNILDTSVGVVSRYFGIHRRVFGLPTCRNQEYLNHMIQFSITVMKRAN

VIDVLPWFLRWIVASFLLKKKDSQDIMVKHIGGLFETRQKTKTPHTSSTTTTSDAIQWILDAAPPGTPTLKLVQRLMLFN

FASIHTSSMTLAHALYDLAANPEFQEPLCIEMEEVLRTEGGWTKQALTKMKKLDSFLRESSRMNGVNTVTVVRKVITSHT

FIDGTYAPKGVWVCAPATTLHRSPELYENPEIFDGFRFYRMRQLEGNAHNYQMASTSLDYLSFGIGKYSCPGRFFAANEL

KIAVAYILCNYQLRLNGGVCGKKPPNVSSGFVCMPNPTEGIELKEREGREKSVLFPGHVTSCSL*

>CYP6271A3|1027707|Tubbor1

MALDAVMSYVQEQILPLSKGTIAAIVFGVWLLYRIALVFYRLYLDPLSKFPGPKLAAATSLYEMYYDIVQDGTLVWKMDE

LHRRYGPIVRISPHSLRLRKSSAYHEIHRMGTPFIKDIRFYHLFGVPRSMFATIDVNLHRQRRSLLNPMFSRKGILDLEF
LIKEKIDILCRRMREYEVQDKFLNCHRAFAALTADIVTEFAYAKCYNVLSTPDFASRAFDSYDTQHEVFLVLKHFPLMAK
VMQALPFWLLVRLMPGGAGFAELEVDAKANLKALSTRMRTGKMEKNPTHRTVFGEILAQHPDPENADPTEMTNEATSIVA
AGIHTSRWALCTGLLEVARDPLIQMKLFEELKTASPDINAQLSYLDCEKLPYLRGVILEMLRLSYGVIGPLPRRVPKTGA
VVGGYHLPGDSTIEMDNYSLHHDEDIFPDSHRFWPERWQTSESKQNEKFVNAFGAGPRQCLGINLAFCELYLTVGTIFRR
FEIDISARGTERMKSKEHWMNILRDEPLKCKVISRKE*

>CYP512CQ1|1028216|Tubbor1

MDAVIDNDTSLRYGGLKMAVEEVLLKRQVNPGVAIGGLVLWMFVWVAWKSYSLRVKVDAVGVSPGLFGGWRAMFGFFPRG
NDWIAEGYKKFSPTGKWFQIPSVARYMILPTSPKILEEMMAEAENILSFNEALADKLAIEWTIHPCIKHDRSHVKLIQTK
LSQRLTTILPEVMDELVLAWEENANVGKEWTKVQISDMMLQIVARTTNRMFVGVPLCRNQEYLDNVIQYAGKVMMMGSIL
DCLPRIFRGPLTNPLMKKSYHYAIMRKHVGHILRERKEKMRELGEEWKDRPDDLIQWVLDFAVDEKASSEEELIARLLMM
NFVSIHTTSEAILQTLYDIGSHPELQPPLHAEITEILSKDGMCKQSLTKMKKLDSVIRESQRLSPITTVTMVRKAMAPHT
FSDGTHIPAGAWMGAPAAEIQRSNFIPNPTVFDGFRWERMGVENQSSGKAGKYAAVTTSFDHLVWGHGKYACPGRFFATT
VEKILLSHIIERFEVRCPEGRPKNKLFGMACIADPKGVIELKMR*

>CYP6001E1|1096761|Tubbor1

MSFLGLFGGGSSSNSQSEYGDESAATQEITYSGIAEDIKAAGGKIPEDLKLLLETGVQAKSSAPVDDKKLVMERLIGLVA
SLPQNSANRKKLTSTIIDTLWDSLQHPPLSYVGDKYQYRQPDGSHNNIMYPDLGKAGTEYARTVRQDKKLYGAKPDASLL
FDLLMARGGNFKQNPAGISSVLFYHASIIIHDVFHTDRNNFSISQASSYLDLAPLYGNNQAEQNKIRTMQDGLIKPDTFS
DTRLLGLPPGICVLLVMYSRFHNYAAKTIKAINENGRFSLPRTHDTDTPEAQKTNLAKQDNDLFQTARLITNGLYVSISL
HDYIRGIANLNHSESTWTLDPRLEIDKTFDGVGTPRGVGNQVSCEFNLLYRFHSGVSKRDDAWTRDFFGKIFPGQDPTTI
GAPQLLQGLKAFESTIPKEPEKRTFGGLKRTGADGTGAFSDDDLVKILKESIEDPAGAFGANTVPEILKPIEVLGILQAR
KWQVASLNEFRGFFNLKKHKTFEDINPDPYVANTLRKLYDHPDMVEMYPGMFLEDTKPRMDPGMGLCAPYTVTRAVFSDA
VTLVRSDRHLTLDYTPANLTNWGITEVAQDYDTLGGSKMFHLILNAFPSYFKYNSVYAMQPFYTPTESRKIFDKFGKSSL
YSFDPPAKIAAPIPILTHAGLKRVLNDQKNFKVPWGESMEALNNEHDFMLAGDLSSNTEQRNLVGDAIYDVTGSRKQFKD
YTEEITLKLLKREVYQLGKVPFNQVDIVRDIGNLVSLHFAAALLYLPLKTDDNPNGPYSEAELYKTLTDLTWFVFSDSDP
TKSWEHRREAKKSIDKLGAIMVEEIKKFKAPVGIWDKLTGGAGVRPPPPTLKDYGSNLVKRLLSKGRSAEDVAWMLLWSG
CAFVANSACAFAQLIDFYLQDDNRKHWSEIQRLATLNTPEADKLLTKYTLEGTRLSNSLGIFRNVDPVDSQTITINQLGQ
DVVLKKNDKVFVSFVYASKDASVFPDPLEIKLDRPTELYITHGEGQHQCLGKDINIIQNTYMLKSLAKLRNFRRAPGDEG
KLKFIPKPGGIKLYMNADWSKFTPYPTTMRVQFDGPIVA*

>CYP6001C|1128940|Tubbor1

MASHSNGTDGATNGHSNGYSNGASNGVSKGKTAKYATRAKPPPPVAKPTRAAVDSTFEQFANLLHASNRPLPNRFGDGQD
RRPPEVKQTGVLTDIKTLRSGGFFWESVGTLWTLLKQKKKGGPVDDKTMIMERVIQLTSRLPPTSKVRVLLTTTQVGQLW
DSLQHPPLSYLGDEFSYRQADGGCNNIMYPQLGRAGSTYARSVKPMTKMPGAPPDAYTLFDSIFSRGPNDEHYREHDNNV
SSMLFYTASIIIHDLFRTNRANPNISDTSSYLDLSPLYGNSIEDQSKIRTFQDGKIKPDSYCEKRLLAFPPGVSVLLVMF
GRFHNYVVENLKAINEGGRFNLKFPRYPNGDDEATRQANALRRQDEDLFQTGRLVTCGLYINFVLNDYLRTIVNLNRVDT
TWTLDPRFEASKVYNPDGTPAGIGNMVSAEFNLVYRWHSCISKRDDQWTKDFYQGLFPGRDTRDIEMPEFLRGVGRWEAS
LSDDPVERNIAGLDRKADGSYHDDDLVKILTESIEDVAGAFGARNVPHVLRLVEVLGIEQSRKWQVASLNEFREFFGLKP
HATFEDINPDPAVANTLRQLYDHPDFVEMYAGLVAEADKKPMVPGVGIGPTYTISRAILSDAVTLVRGDRFYTVDYTATH
LTNWGLQEASSDPAIVHGCVGYKLILKAFPNHFKYNSIYAHYPLTIPEENHKIHTALNTVDQFDFERPVYTPLRIPISSY
SATKQILCDAESFKVTWGAGFDYIMRADFMLSGDKPSNTEQKQFVRQRLYLGDVDWRQQIRQFYEECTERLIRKKAYRLG
DAYQVDAVRDIGNIAQTMFAASIFNLPMKSEDHPKGIYTEQELYTILCVMFIIIFFDIDSSKSFPLRQAGFKVVRQYGSL
VEAQVKAIKNWSWLQGVWDPLNIRGRNKSSPLKSYGWTMIKRLLESGKSPYDVTWSYIVPTAGASAPNQGQIFAQVLDFY

LEDRNAHHLAEIQRLAHLGTDDAWETIKKYALEGGRLAGTFGLYRRVEPDNITIEDNGRNIDLRSGDMVFVNFITASRDP

VVFPDPLEVKLDRPEASYMQYGDGPHECLGKAANIIGLTTMLMQFGKLKGLRRAPGPQGELKYIPKPGGFKVYMKEDWSA

YWPFPTTMKVRFDDII*

>CYP663N1|987307|Tubbor1

MSLIQAISSLPGGSAAALALLVLSVGILAQQLFKKKDPLPLPPGPPGLPIVGNSFQIPLINPWRKQAEWTKQYGPIYKLK

LGKDTVIVLGTQQAAKDLLEKRSKIYSSRPRTVMAGENVSRGKRMLNRPLGDKWRMHRRIQGQVINQNVANKYKGFQNLE

STQLAKELVDRPEGFFDSFHRYNSSVIFAMAYGKRMPRGDEEDVVAVDEITENFLYSARLGTWIVDSFPFLNYLPTFMAP

WKRIGDNFYNWAEKMHTFNRNEALQRKGWNWTKEIARMKEAQNISPLELAFMVGFLYEAGSDSTTIALEVFILALLKHPE

VLKRGQEEVDRVVGPDRLPNFDDKDNLPYIRHCVDEVLRWRSPSAGGVPHVVEEDDEYMGYRIPKGAIVVGNLWSIHQDP

EVYPNPTKFMPERWDDEENVHYGFGFGRRACPGRHIAGNSLFVNYARIFWGFNVEHAKNPDGTVIPVDEWDMTQGFMSRP

VRYKASITPRDAKRVEVINQAWKGAEVQLQDMSKVLKMEDF*

>CYP663L3|995887|Tubbor1

MASYCTWRFCSLFNITSSPFDKMCIAGTCTEDPGGNTPATVTTKPLALLISFLAVLTATYLFQQKRRQLPLPPGPRGVPI

FGNLFQVALKHQWRQQQEWTQKYGPIFRMRLGAQTVIVLGTHQAARDLLDKRSKIYSDRPELVVSAKHLSGGYRSLLMRG

EMWETHYRLQSTVLSPRMSQKYKPVQDLESKHLIHALLKKPDDFSQQLHRYSASLAFSLGYGKRLVTPHEKELESIDTIV

RNFAEAAALGRWWVDIFPILDILPKYFAEWKRISPKLYQHESELHSTNLNAAREQKGWNWAKVYKDSPYSQGMPDVEIGF

DLGSLYEAASDTSTIAQEVFILAMVKFPYVARRAQEELDRVIGKSRLPTWQDKDSLPYIEKVIKETLRWRPVAVNAFYHA

VTEDDEYLGYRIPKGSWVAANVWGIHLNPEMYPNPHDFNPDRYDDEKLGHVAFGFGRRACTGQHIAKNSLYINISRMLWA

FDIGPKIRSDGTEVPVDDMAFTNGFLSRPLPFECSIVPRDQGRVKVIEKEWAEADKDLASILAEEKLT*

>CYP663L1|1040356|Tubbor1

MASYCTWRFCSLFNIASLLDKMCISGTCTQDPGGSTSRPVTAKLPALFISSLAVLIATYLIQRKRRQLSLPPGPRGVPIF

GNLFQIASKYPWRQQQEWTQKYGPIFRMQLGAQTVIVLGTHQAARDLLDKRWKIYSDRPEFIVCAKHLSGGYRTLLMRGE

IWNTHHRLQATALSPRMSQKYKPVQDLESKHLIHALLRWWVDIFPMLDLLPKYFAGWKRISAKFHKYESELHLTNLREAR

ERKGWNWAKLYKNSPFSQGMPDLEVGYDPVGTLYEAGSDTTTTALEVFILAMIKFPHVARRAQEELDRVVGKSRLPTWQD

KESLPYIEKVIKETLRWRPVAVNAFYHAVTEDDEYLGYRIPKGSWVVANVWGIHLDPELYPNPHDFNPDRYDDETLGHVA

FGFGRRVCTGQHIAKNSLYINISRMLWAFDIGPKLRSDGTEVLIDDMAFTNGFLSQPLPYECSIVPRDQERVKVIEREWA

ETDKDLLSILAEEKFT*

>CYP617W2|1070705|Tubbor1

MSTTNILVGCAASTILISRYAHYSAITTFVLLTSLALTCKVLYHLILWPKFLSPTRHLPGPTNPSLLMGNFATVRKMQSG

AAQAFWMRTIPNSGLLRYLGIFNMERVFPTTPEVLKEVLHTKSYSFVKPPLIAINIGSILGKKGLLFAEGEEHKAQRKLL

LPAFSHAHIKGLVPGFWSKGVEMTEKVAEVVRSSRDGPGDGGEEGVVVQMGRWFSLVTLDIIGSCGFGYEFRALESASIS

GSSNIEDKSGSELADAYKAVFNTGGPSRMVAILSMIFPFRLVRLLPLKRTRDVDRASSTIRRVTAQIIAAKKSMLATSPE

DSESGDILSIMLKSNAYTGPDGECSMRDQMMTFLAAGHETTATSMIWAIHVLSLPENRHMQSRLRAEIHAAFPSGLPTTV

TYDQISNLRYLSQIMSEVLRLYPPVGATLRMAAEDTSLNGSFIPKGTTLMISPFAINRSVALWGADAEEFRPERWASKED

GAAAVESNYGFLTFLAGPRGCIGNMFAKMEFKCLLAVTIGRFEFSQDGKREVVVKGGAANKPQGGIPVSVNEVVWG*

>CYP617W3|1070696|Tubbor1

MSTTNILAGCAASTILLSRYTHYSAITTFVLLTSLALACKVLYHLILWPKFLSPTRHLPGPTNHSLLMGNFSIIREMQSG

APQVIWMRTIPNSGLLRYLGIFNMERVFPTTPEVLKEVLHTKSYSFIKPPLLTRDIGNILGKKGLLFAEGEEHKVQRKLL

LPAFSHAHIKGLVPGFWSKGVEMTEKVVEVVRSSQDSTGDGGKEGVVVQMGRWFSLVTLDIIGSCGFGYEFRALESASIS

GSSNIEDKSGSELADAYNTIFNTGGPSRILAMLSMVFPFWLLRSLPLKRTRDVACAESTIRRVTTQIIAAKKSTLATSPE

DSESGDILSIMLKSGAYTGPDGECSMRDQMMTFLAAGHETTATSMIWAIHVLSLPENRHIQSRLRAEIHAAFPSGLPTTV

TYDQVSSLKYLSQITSEVLRLYPPVGVTLRVAAEDTSLNGAFIPKGTTVILSPFAINRSVALWGADAEEFRPERWASRED

GAAAVESNYGFLTFLAGPRGCIGNVFAKMEFKCLLAVTIGRFEFSQDGKRELVIKAGVTSKPQGGIPVSVKEVVWG*

>CYP617W1|1070700|Tubbor1

MSTTNILAGCAASTILLSRYTHYTAITTFVLLTSLALTCKVLYHLILWPKLLSPTRHLPGPSNHSLLMGNFSIIREMQSG

APEAIWMRTIPNSGLLRYLGIFNIEQIFPTTPEVLKEVLHTKSYSFIKPPLVARNIGSILGKKGLLFAEGEEHKVQRKLL

LPAFSHAHIKGLVPGFWSKGVEMTEKVVEVVRSSRDGAGDGGEEGVVVQMGRWFSLVTLDIIGSCGFGYEFRALESASIS

GSSNIEDNSGSELAGAYNTISNMGGPSRFVAMLSMIFPFRLVRSLPLKRTRDFERAASTIRRVTAQIIAAKKSTLATSPE

DSESGDILSIMLKSNAYTGPDGECSMRDQMMTFLAAGHETTATSMIWAIHVLSLPENRHIQSRLRSEIHAAFPSGLPTTV

IYDQISSLKYLSQITSEVLRLYPPVGVTPRVAAEDTSLNGAFIPKGTTIILSPFAINRSVALWGADAEEFRPERWASRED

GAAAVESNYGFLTFLAGPRGCIGNVFAKVELKCLLAVTIGRFEFSQDGKREVVIKAGITSKPQGGIPVSVKEVVWG*

>CYP52AV6|1073961|Tubbor1

MELAVKSFLLLATALYLLLKLTAYLLIAHQNRIFAKSHNCLPPGRLPSSFLGLSNWVRLTRAAKRGDILDHIVSRFPIYG

NTWKGRILFGTSIGTIEPENIKAILATSFKDFDLGPQRHDNFYPLLGDGIFTLDGAGWEHSRANLRPQFSREQVSDVEAL

ELHVQRLMNRLPEGDGEVADLQPLFYCLTLDSATEFLLGESVDSLLSPELNPTGAARGGKEEMSFAQAFNVSQAYLVQRS

RLRELYWIINPKRFRDANAIVHRLIDRYVDMALHPEKRARKAPEGKYVFLDAIAAETKDPKYLRDQTLNILLAGRDTTAG

LLGFTFWLLARHPHVYQKLREEILSAFGTGLNNEGKRPSFSALKNVTYLRYVLNETLRLYPSVPLNGRTAVRNTVLPRGG

GEDGLSPVFIPKGQRVDYTCYGLHRRKDLYGEDADAFRPERWEEGVGRGWEFLPFNGGPRICLGRMFPHLLVIVVAVGRL

LTPSVNRAIRPDRSLLHSYQDLAEICED*

>CYP6135A|1085741|Tubbor1

MLPPLFLTTALLYIGYICYPLHNLLCKVRPLGIPYFVVLFYPSQFTRTFIFPPMKLLIKVLGLTHPQFFPIASDWQVRQR

YKIYRFIGSDISFTVTPWKIILHVADPDMAVEVLAGKGWNGELYPKPEQESLAQIKQMIQYYDKREATNTSYRDFRRATL

GAIAHAGFSKVMEWAPLTTAIANEKDSEGSYQKCLHVLIENFKWTLIAPTGGAANGFRFFMNEWFEEKKAKIELDNKTAV

KPSREDLMGSLVRSSGLDKGSTLETPLLPKSDVIGNTFIFILAAHETTAHKIANAIYFLAMYPEYQVKLQEEIDSILGDS

DHSLTSYETHFDAFSSGWIAAIMFETLRLIPAVIVTARHMGPTPGTFPRAGGQRLITLPPGVKFIIQILGLSHNPKYWAQ

PEKTEAESAISEFRPERWLPKSGDNTMFKPYNGSYMPSSIGTRGCIGKKFAHVEFTAIMLGLFREMSVEFDSCGGRKTFE

EARLECLAEMKKYESKITLQPSGEMPSIMWVRRRSYVIS*

>CYP6135A5|1085753|Tubbor1

MLAPLFLTTALLFMGHTCYSLHSLLCKVRPLGIPYFAIPFCPSPISRVFGLTHPQFFLMTLDWQVRQRYEIYRLIGSDIF

FVVSPWKVVLHVADPDMAVEVLAGKGGNGELYTRPDVYNSMLGLFGENVLTAEGAVWRDHRKITAPVIGKSLSVVWEESL

TQIEQMIRYYDKREAINTSYRDFRRATLGVIARAGFSKVMEWGPLTTALANEKDDEGSYQKCLHVLFENFIWAVIAPSWI

LEMLPFKTLREAGGAARNFRFFMNEWFEERKTKIELDNKTTLGPSSKADLMESLVRSSGLDKGSTLETQLLSNLRIFILA

GHETSAHTIANAIYFLAMYPEYQVKLQEEIDSILGDSDHNQTSYETHFDAFSSGWIAAIMFETIRLIPGLAVMPRQIGPI

PRTFPRAGDLRPITLPPGIELWIHVIGLSHNPKYWTQPGKTEAESAISEFRPERWLPKSGDNTLFKPYNGSYIPFSIGVR

GCIGKKFAHVEFTAIMLGLFREMGVEFDSCGGRKTFEEARLECLAEMENFENIITLQPTGEMPGIKWVRRR*

>CYP6135A6|1085762|Tubbor1

MLAPLFLTIALLFMGYTCYSLHAQLCKVRPLGIPYFVVPFYPSPFPMIFLLPFVKLLIKVFGLTHPQFFLMTLDWQVRQR

YEIYRLIGSDIFFTVSPWKVVLHVADPDMAVEVLAGKGQNGELYSRPEIVSRMMGLFGENVLTVEGAVWRGHRKITAPVI

GKSLSVVWEEALTQIQQMIQYYDKHEAINTSYRDFCRATLGVIAHAGFSKVMEWCPLTTAIANEKDDEGSYQKCLHVLCE

NFIWTVIAPSWLLEMLPFKALREAGGAARNFRFFMNEWFEERKVKIELDNKATLKPSSTADLMESLVKSSGLDKGSTLET

PLLSKSEVIGNAFIFILAGHDTTGHTIANAIYFLAMYPEYQVKLQEEIDSILGDSDHNQTSFETLRLIPALITATRQIGP

TPRTFPRAGSQRPVTLPPGIEFWIQIIGLSHNPKYWTQPGKTEAESAISEFRPERWLPKSGDNTMFKPYNGSYIPFSIGA

RGCIGKKFAHVEFTAVMLGLFREMSVEFDSCGGRKTFDEARLECSAQMSKFEMKLSLQPTGEMPGIKWVRRR

>CYP52AU5|1100054|Tubbor1

MGIFVVDGISWSHSRALLRPSFDRTNVADLSQLEGFMQIFFSKINESPQQPFTHSRVVELQELFQRLTMDSSTNFLFGKP

IGALTSKKFTLGNGEMTFDQAFDIAQHGLAMRAPLSSFYWLYNGREFRKACLTARTQAAVYVSHTLKKLSLAEKKAEKEG

LDKKYVFLEELAEQTQDPQVLQDQVLSVMLAGRDTTAALLSWTFLCLAKNPEVFKKLRAEISVVVGVDENARLPTQAELR

SMQYLRWIIQEVLRLYPPVAINARRALHPTTLPYGGGPDGNSPISIRKGERVAYSTFSLHRRTEVYGPDAQEFRPERWGE

EALRKVGWSWLPFNGGPRICIGQQMALTHASYFIARMMQVYKEITLQDSSSVDDAYDTKLVMASGRGVHVLLS*

>CYP6135A1|1108743|Tubbor1

MNFTVSDPPRDKGLSKEWNTIFSQSSAAMLTPLFLTTALLFMGHTCYSLHTQLCKVRPLGIPYFVIPFYPGPLPRIFLIP

FVQLLIKVFGLTHPQFFLMTLDWQVRQRYEIYRLTGSDIFFIVTPWKVVLHVADPDMAVEVLAGKGGNGELYTRPEIFSS

MLGLFGENVFTAEGAVWRDHRKITAPVIGKSWRRLSVVWEESLTQIQQMIRYYDKQEATNASYRDFRRVTLGVIARAGFS

KVMEWVPLATAIANGRDDEGSYQKCLHVLFENFIWAVIAPSWILEMLPFKALREAGGAANDFRFFMNEWFEERKTKIELD

NKTTLGPSSGADLMESLVRSSGLDKGSTLEAPLLSKSEVIGNAFVQALPFSLQHLANSPQIFILAGHETSAHTIANAIYF

LAMYPEYQVKLQEEVDSILGDSDHNQTSYEAHFDAFSSGWIAAIMCETLRLLPGVATTARQIGPTPRTFPRAGGQRPVTL

PPDIEIWIQMIGLSHNPKYWTQPGKTESQSAISEFRPERWLPKSGDNTMFKPYNGSYIPFSIGARGCLGKKFTHVEFTAT

MLGLFREMSVEFDSCGGRKTFEEARRECLAQMENFESKITLQPTGKMPRIKWVKRRR*

>CYP6135A15|1126297|Tubbor1

MLASLFLASALLFMGYACYSLHTLLCKVRPLGIPYFVIPFYPSPLPRVFLLPLMKLIIKVFGLTHHWFFLLTLNWQVHQR

YKIYRLLESDIFFTVTPWEVILHVADPDMAVEVLAGKGENGELYPKTEVVSRMMGLFGKNVLTVEGAVWRGHRKVTAPII

GKSLSIVWEEALTQIGQMIQYYDKNEAINTSHRDFRRATLGIIAHAGFSKIMNWVPLTTAIANEKDGEGSYQKCLDILFE

NFKWTVIAPSWLLEILPFRASREAGGAAKGFRRFINEWFEEKKAKIELDNNTGPRPSSRTDLMESLVRNSGLDKGSTLET

PLLSKSEVIGNAFMFILAGHESTGHTIANAIYFLAMYPEYQVKLQNEIDSILGDSDHNQTTYNTHFDTFSSGWIAAIMFE

TLRLIPAVVVVTRQIGPVPAPSLAQVAEDLSRSPPVLNSGSNLSAWLKTQNTGPSPGKLKPNLQSPNSGLNAGSRNQEGI

PWLFREMSVEFDSCGGRKTFDEARRECLAEMDKFATVITLRPSAEMPGIKWVRRKKGVAA*

>CYP6135A11|1141696|Tubbor1

MLALLFLTSALLFMGYTCYSLHTLLSKIRPLGIPYFIVPFYPSLLSRVFLLPFVRFLIKAFGLTHHWFFLMTFDWQVRQR

YEIYRLIGSDIFFTVTPWKAILHVADPDMAVEVLAGKGKDGELYPKSKIISSIALFGENLVTVEGAVWRGHRKVTGPVIG

KSLSVVWEESLAQTEKMIQYYDKSEAINTSYRDFRRATLGVIAHAGFSKVMEWVPLTTAIANEETGERSYQKYLNILFEN

YKWTLIAPSWLLEMLPFKAAQDAGRAVNHFRSFTNEWFEEKKAKIELDNKTALKPSSREDLMESMVRSSGLDKGSTLETP

LLSKSEIIGNSYIFVLAGHESTAHTIAHAIYFLAMYPEYQVKLQNEIDSILGDREHNQTSYDTHYDAFSSGWIAATMLET

LRLIPATSVIFREIGPTPRTFPLSGGQRPVTLPPGVEFWVQPIGLARNPKYWTQPGKTEAESAISEFRPERWLPKPGGNT

MFKPYNGSYIPFSIGARGCIGKKFAQVEFTAIMVGLFREMSVEFDSCGGRKTFDEARQECLAEMEKFVMKLTLRSSGEMP

GIKWVKRKRQVVT*

>CYP6135A2|1193869|Tubbor1

MLTPLFLTTALLFMGYTCYSLHTLLCKVRPLGIPYFVVPFYPGPLTRIFPIPFVNLLIKVFGLTHPRFFLMTSDWQVRRR

CEVYRFIRSDIFFTVTPWKVILHVADPDMAVEVLAGKGGNGELYTRPDVYNSMLGLFGENVITAEGAVWRDHRKITAPVI

GKSLSVVWEESLTQIQQMIRYYDKHEAINTSYRDFRRATLGVIAHAGFSKVMEWCPLTTAIANEKDGEGSYQKCLHVLFE

NFMWAVIAPSWILEMLPFKALREAGGAANNFRFFMNEWFEERKTKIELDNKTTLGPSSKADLMESLVRSSGLDKGSTLET

PLLSKSEVIGNAFIFILAGHETTGHTIANAIYFLAMYPEYQVRLQEEIDSILGDSDHNQTSYETHFDALSSGWIAAIMFE

TLRLIPGATTISRQIGPTPRTFPRAGSQRPVTLPPGIEFWIQIICLSHNPKYWSQPGKTEAESAITEFRPERWLPKSEDN

TMFKPYNGSYIPFSIGARGCIGKKFAHVEFTAIILGLFREMSVEFDSCGGRKTFDEARLECLAEIERFEPKITLQPSGEM

PGIKWVRRR*

>CYP6220A2|964006|Tubbor1

MIDSEYFPNGVSKTEYLTAVICAFGGFIVWKVISAIFFSPLSHIPGPLVTAVTPHYINFLSALNRRTVGTYSLHKRYGPI

VRLSPTEISVLSPQAIKEVYSSPQYTKYTPLYSIFTHFGAQNAFTSCTREEHGWRRKAVSESYSLSFVLKNEAATGKVLR

VVKDYLGFVESDRRVDIYNANTFYATDVVTGKIFGSEASMKTLVGNEAHREIVLGHYARTRRTQVWMYIEFPFIMNVFEW

VLFYTGRIWSWVCGEVIEDWEMVSQIQKWGWDAYMNAQSNSGEDTVAGRLGRLPQEGSSTEGVWDDKGAVSEILDQMLAG

MDTTGDTLSFLMYQLSLAQSRNAQKRLHSELLDAFPKSAEKSSSGWPHSMDIQSLPHETILKVLNLPYLDAVLKETLRVY

TAIPITLPRVVPPSTTTDSVSGHRNGRIVEGKFIPAGTAIGTLAYGIHRDEVFAVETGRRDEPGDVNSFLPERWLINCGI

EGELTPDELAQEKQRIRIMEARLWAFGSGARNCVGRHLSILEMKLLLATIYSRYQTQATPNSGIKITHNRWNERRTFRDV

LPFRGVDGVVTFTPYES*

Tuber brumale v1.0

>CYP6135A16|1055265|Tubbr1_1

MLASIFLTLVLLFLGYACYSLDTLVLKVRPLGVPYFLLINAFGLTHVWFHLLTLDWQVHQRYEIYRLIGSDIFFTITLWK

VILHVADPDMAVEVLSGKDQNGELYPKTAVVGRMMGLFGENVITTEGTVWRGHRRVTAPVIGKSLSIVWEEALAQVKKML

QYYDKQEDIDTSHRDFRRVTLGVISHAGFSKAMDWVPLMAKGVIKSVSISYLRISSGPLLHRVGFWVKMIPFKASREAGG

AANKFRFFANEWFEEKKVKVELDNKTALKSSRVDLMESLVRSSNVNEGSTLGTPLLSKSEVIGNAFIFILAGHESTGHTL

ANAIYFLAMYPEYQVKLQNQIDSVLGRTDHNETSYDKHFDAFSSGWMAAIMLETLRLIPAAVVVVAC*

>CYP6271A3|1077236|Tubbr1_1

MALDVVIAYVRELAPSLSRGTIAATVLGVWLLNRIGLVFYRLYLDPLSKFPGPKLAAATSLYEMYYDIVQDGSFTWKMKE

LHEKYGPIVRVSPYSLRLRKSSAYHEVHRMGTPFSKDIRFYRQFFIYDAIFSIMDNELHRQRRSLLNPVFSRKAILNSEP

VIKEKLDLLCRRMKEHEAQDKLFNCHKAFAAFIVDIITEFAYSKSYDILSLPDFDSRIEHALDSQLEAILIFKHFPILAE

IVSWLPIWIINSLMPDGGSFAELELDAKVHLMEVFARVSDGTIKNNPAHRSIFGGMLAENPDLKSTGPTKLAQEAMSIVA

AGVHITRWTMCVGLLEVARDPLIQAKLYEELKMASPDINGEFSYLRCEKLPYLRGVILEMLRLSYGIPSTLPRRVPKEGA

IVGGYHLPGDSTIEIDCYSIHHDEDIFPDSHRFWPERWLTPESKQNEKFVVSFGAGPRQCIGINLAMCELYMSIATIFRR

FEIDISARGTERMKFTEHWSVILRDEPLKCKVISRKE*

>CYP6188A3|1079589|Tubbr1_1

MAKILRPIESTLQELHLEAPTQKTGTGNTTAKYLIAFLTNLSTESQWDYPLSFIPGILGAVLVYILVKLGLHLHRTRNDT

PLKNVPGPWLASCSPLYRFWYAVLKGNFHHDLTNLHRRYGDVVRIAPNEVSIWDPRATSEIYAHGDKGYAKCDMYDIALP

NGFFNLAVERDIQTHAEGRRAIAKDYSMTATLMAEAHFDNIIKDFILALDKNFAQKGGVCDFTIWSEYFTYDMITDLVFG

EAYGFCKTGLDVDGSLKDLRQMLLLSPFLSYLPWIWPITQNQWVKKIGMNHYARNIKSNIERRLNHGNPSGRRDLLDRLF

RSRNHAYIFILAAPDTASVALRAILINLVRNRNVYNEVMAELIGLKLSNPATWKELAGAPLLHAIVKETLRLHPPAGFNL

PRAVPAGGRTICGYYLPEGTTVGMSAWCVHANEGFWGKDTMEFKPERWLDPERAFELDHYGLSFGQGARACLGKNIAMVQ

LVKVTAQILLNFEFELVDEHKIRGVFLLLVVLDGVKVAFKRRAGGPLDDTIGTEGVAN*

>CYP663L6|37731|Tubbr1_1

MEVFNPGSGPTTPRPAWRFCSLVNFAFYPFDKMCIAGTEDPGGNTSLPITIRPLPLFTSFLALMIGTYLFKRKWRQPPLP

PGPRGIPIFGNLFQIAPKYQWRQQQEWSQKYGPIFKLQLGAHTVIVLGTYRAARDLLDKRWKIYSDRPAFVVADKYFSGG

YRTLLMRGEMWNAHHRLQATVLSPRMSQKYKPVQDLESKHLIHALLKQPDDFARQFHRYSSSLVHTLGYGKRLVTGREKE

VQMIDTIMRNFTEAGRVGRWWVDIFPILELIPTYFAEWKRISAKFHEHESNFHIKNLNEALERKGWNWAKLYKNSPFSQG

MSHLEVGYDLGTLNEAASDTTTIALQIFILAMIKFPHVARRAQEELDRVVGESRLPIWQDKDSLPYIEKVIQETLRWRPV

AVNAFYHAVTEDDVYLGYRIPKGSWVVANSWGIHMDPELYPNPHDFNPDRYDDETLGHVAFGYGLRTCTGRHIAKNSLYI

NISRILWAFDIGPKMGADGTEVPVDEMAFTTGFLSQPLPFECSIRPRDQDRVGVIEREWAQADKDLLSIMAEEKFA*

>CYP5959A5|1086684|Tubbr1_1

MGIFAQLIDYAKDKNLYGYYLLGTSLALVFLGLFLLLMKEWSIHGPTKLPIIGVESPGYFARVKARQKFLFNGFQMVRDA

YYKNCGKGFVVTTHSYDKVILTHAQVRELSNAPDDTLSLAHASARTLMAEYTGMGRFVDTGYVRDATKTKLTQNIGNMIG
ALLEETKFALDRELPACTADAESVPVDIVTAVIRIVARVSARSFIGLPLCRDEDWLTISINFTRDIFKTAMKLNAVPKLI
RPFYAYIWNSSKIIDTHKRKAQSLLTPIVRKRFEERSLAEKNGEVYQTPNDLLQWLTDLVKPQHKTVDALSELQLGVSLA
SIHTTSSTFVNTILDLAEHQECIQPLREEMEKAISESGGVLDREAIRKMRKADSFFRESMRTRSFLFTFNRMAMKNLTLS
DGTYLPKGTLLGAPNAMFSTDPGFIEDPETFDGFRWYKKSLEAGDSAPKGNGCAATSASSLAFGHGKHACTGRYFAVEEM
KIMLAFIVLQYDIKYPEGQSRPDDIVVGEFSFPDSKQKVLFKRLPGQKEKYSFL
>CYP512CQ2|1096156|Tubbr1_1
MDTVMNNDTGSLYGWFKATVEGIFLKRGVDRGVVMGGLLLWMIGWMVWKSYSLRVNVDAVGDSPGPFGGWRAMFKYFDHG
SDWITEGYKKYSPTGKTFKVPTVARYIVFPTSPKILEEMMTEPEHILSFDEALTEKVAVEWTLHPSIKYDTYHLKLIRTK
LTQRLSIVLPEVMDELTLAWEENTNIGKEWTKVRVWDVMLQIVARTINRMFVGVPLCRDQEYLDNVIQYTIKVVKAGAIL
DILPRIFRAPLTNLIMQKSHHYAIMRKHVGHIFSERKEKMRELGSEWKDRPDDLIQWILDLAGDGKASSEEELIFRLLFM
NFASIHTTTSTIVQALYDIAANPELQPPLHAEITGALSKDRMCKQSLTKMKKLDSVIRESQRLNTITSMTVIRKALVPYT
FSDGTHLPVGTWVAAPATAIHLSASIPNPTVFDGFRWERMAAEDQASGKAGKHAAVTTSFEQLAFGHGKHACPGRFFATN
ELKILLSYVIERFEFRCLEEGRPKSRFFGVACLANPEGVVEFRMR*
>CYP5959A7|1138793|Tubbr1_1
MGIFIQLTDYAEDKSLYGYSLFGTTLALISLGLFLLLFKEWSIHGPTNLPIVGVESPGYFARVKARRKFVSNGFQIVRDA
YYKYYGKNFVITTNSNEKVILTYDQVKELANAQDDTVSMAHSSAQTMMAQHTGLSGFVGTHYVREAVRIELNRNLGNMIG
DLLEEAQFAMNIELPEFTTDEWVSVDIPAAVLRIVARISGRAFIGLPLCRNEDWLTLSISFTTDVFQEINKLTPVPKILR
PFFAYIWNSAGSVNSHKRKAQSLLAPVFQRRFEEEALAEKNGTVRQNHRDLLQWLTTRVAPNHKNVNVFSGLQLMIGLAS
VHTTTSGFTNMIFDLAEHQECIQPIREEMETLISANGGVLDRATLRKMRKTDSFFRESFRTSLLLLGFNRMIMKNMTLSD
GTYLPKGTLVAAPAAMFSSDPDFVEDPETFDGFRWYKKSLEVTDSAADNNNATNISPTNLIFGYGRHACPGRYFVVEVMK
IMLAFMLLQYDIKYPEGQSRPPNIQMGELSYPDRTQKLVFKKRPGPKKFGFLQ*
>CYP52AV3|1140086|Tubbr1_1
MELAFKFLLLLATAFYLVLKLIAYLLIVRQNRIFAKSHNCLPPRRFPSPFLGLPNWVYLTRAAKRGDALEHIVSRYPTYG
NTWKGRVLIGTTIGTIEPENIKAMLATSFKDFSLGPQRRDYFYPLLGDGIFTLDGAGWEHSRASLRPQFSREQVSDIEAL
EVHVQRLMNQLPEADGEVADLQPLFYCLTLDSATEFLFGESVDSLLSPELNPTGAVSGGKEEMSFAQAFNVSQAYLIQRT
RLRGLYWMVNPKRFRDANAIVHRLVDRYVDMALNPEKRAKRVSEKKYVFLDAIAAETKDPKYLRDQTLNLLLAGRDTTAG
LLGFTFWLLARHPHIYQKLREKILEAFGTGRDGEGKRPSFSALKDVTYLRYVLNETLRLYPSVPLNGRTAVRNTALPRGG
GEDGLSPLFIPKGQRVDYTCYGLHRRKDLYGEDADTFRPERWEEGVGRGWDFLPFNGGPRICLGQQYALTEASYTVTRIL
QKYARIEVADTDTGPMMDLTLTIAPKKVLLRLWKA*
>CYP567X2|181289|Tubbr1_1
MVTLHRFEGAALLVQESVKDVPAWKLCVYVALLFPLYAITMGIYNLFFHPLRNFPGPKKAALSNIWYSYIWLCGRYPHRI
HALHEKYGSVVRVAPNQLSYNTASSWKDIYTNYGNRQGFNKTGLYDKDKMDPDTNIAREADPVKHSRIKRLFSHAFSTKS
LTEQEPIVQEYVDLFVSQVGKHGTGKDGLDIVKWYNYCTFDIIGDLAFGEGFNATKEGEPHFWISLILDSVYAASFINVT
TRFPWLSKLVPSIAPAGMVERRQRHLNYGVDKVNRRVNSDNKRKDFLTNVLENYRDQISDGELYSNAQIFIVAGSETTAT
TLSGLTYYLLHNPHTYQRLVSEITSSFTSYSEITSITAGRLEYLGAVINETLRMYPPVPIGLPRYSPGETVDGRFVPKGA
MVSTSPWASGHCPKNFHDPWAFKPERWLDGECEEKDIREASQPFSLGTRGCLGRNLALMELRLIVCKMLYTYHLELLDTK
LDWERDSTAYVLWVKPDLRVRLHRR*
>CYP6136A1|687042|Tubbr1_1
MFFNLFGQAVYGLIQHKLYLLAGTLILTLAYWTYAYLTSPFHKQKIPGPFLGKFTNAYRWYYVMRHTWHRDLAALHKKYG
PVVWIAPNDISVSDPSLRNVIYGFQNHKKDFTFFRKSHAYETGSINQDFSFIFELDPEKARLGKYYMSHFYSERGLSDLE
ENFDKAVNELIEGLDKHHARTGIPCKMVEWGELFALDLVAQITIDQSAGFCLAGKDVNNTAYGMRIILKTVGALMMLPWV

LSATSRAIRQNLLLRFLINLYRNVLLLPTFTFDNGIADLKSLKDKNPKHLLAKFYNAQSKMREHYPHGNQAEGTTIQLFN

LIAGAAGVVPHTQVKLIQELALHPEILQKILEELATTDNTFRLEDFIHHDNRQKYPIFESAVREAVRLHPVVSFSLSRKV

PPSGCQLHQYHIPPGYNVGMASYQVNHDEGYFGSDVLQFRPERWLEDHPTEMLEGEKRSMKNYIEAGWLTFGSGGRVCIG

RHLAMFMMMKFTAAVVREFDIRVVKQPVEYHTLFTEMQGMEVLLSRKCAV*

>CYP6608A1|877866|Tubbr1_1

MPSLVLMSLVPFLKLLVGLVFTQMLPLALLALVASCVYSRYASPLKNVPGPALASFSRIWLNVGLVARGDLHGGILDAHR

KYGPVVRVGPNKVYFSDPGLIPEVYGVGTKYRKSDLYVMSDPEPGHGSTFSERSAEKHAALRRRVGPAYSNSYVLQMESY

VDTLVSAWISALSSEFTFAKTSVKSCDFARWAQFLTYDVISELAFGEAFGFIATRSDYKQYIASLQQALPIIVIMSYFEE

LVKFMNLGWVRKFTTPSIKDETGYGNLTKTATDLVRERWAKGGVGERRDMLQRFYEQGMTEKEAIIDSTLILVAGSDSTA

TAIRAAVMFICSDPSAYRTLKSELGANIPRTNPASVISFSVARSLPYLSACIKEALRLFPPGAGAMERVVPAGGASLSGY

FIPENTVVCLHPWPVCRDEVFGEDVDSFRPERWLEEKDGKQLAKMEKTMDFVFGAGPHYCLGRQIAYMELYKSIAELFLT

FDIGLSNPTQPWKSREYGFFLQRDMMVKIKKVDG*

>CYP530G1|883262|Tubbr1_1

MAIPVFWTTVPGLALGVCLASFIATIIYFAKGTSRGKNFPPGPPTLPILGNMHLVPQERPYLKFTEWAKQYGGIYSIKIA

KQTIVLISDVKILKELYDKRGAIYSSRPLPHIGAEIVCPDQTHIIYMPYGETSRNYRAQYHQFMGPGKVEQILPWQSAES

TLLIKKIATSPDRYYEHTMRMATAVILESVFGVLPKDYDDPEVTELWTVQREFSEILALTGPPVDHFPFLKWLPDIVSPW

RIHARNVRAMHRKLYFRLLNLNKARMEKGERYGTFVEKLIDDKPKHGLSDERIAYVCATLMEAGSDTTASQALDFMMALL

AFPDVLKKAQEEVDRICGTSRLPTLDDRDQMPYIEACVNEVLRWRPPLPYGAPHLLMKDDWYEGYFMPKGTVLFQVQWAM

NMDENVYEKPQDFVPERYIRNRFGTKFDAEKDAETGRKEQYGFGLGRKICPGQWFARNTLFVLFAKLVWAFDMKVPTDPK

TGKLLPLDTDVRTAFMDGLTTTPFKFPIEFKIRSKAHEEAMNTDLVASDKIFAKYGGATV*

>CYP52AU4|883312|Tubbr1_1

MGIFSVDGVSWSHSRALLRPSFDRTNVSNLSQLEGFMQIFFSRIDELPKQPFTHARGVELQELFQCLTMDSSTNFLFGNP

IGALATKKYTLGNGEMTFDQAFDIAQYGLAMRAPLSNFYWLYNGREFRKACSTTRAQVALYVSRTLRKLHLAEKSQEKKY

VFLEELAERTQDPQVLQDQVLSVMLAGRDTTAALLSWTFMCLAKNPAVFKKLRAEISEVVGVDEDARLPNQAELRSMVYL

RWIIQEVLRLYPPVPINARRALRATTLPYGGGPDGNSPISIRKGERIVYSTFSLHRRTDIYGRDADEFRPERWGEEALRG

IGWSWLPFNGGPRICLGQQMALTHASYFVTRILQVFKEITPQDSALEGDAYDTKLVMASGRGVHVFLS*

>CYP51070A5|886585|Tubbr1_1

KQYGKYGKSFRVMTPARDIILLSNQEALQELIDLPDSVTSSRQAGDEMLGTTYTFHEGMIQDAYHVDLIRKNMTEKLLNI

LPDIVDEIKVASEELIDVKDDWTNINVSNISSGIISRYIITRLILVGLPLCQNQEYLNNISRFSLNVIKGAMAIETVPWF

LRWTIVRFFLNKNGVQDLVVKHVGGVFEGRQKLKISGTSSPTPQSDAIQWILDAAPPGTPILKLVQRLMFFNFASIHSMS

IILAHFLYDLAANPEFQDPVCAEIEEVLISEGSWTKQALTKMKKLDSLFRESARMNTPSILSPIRKVLIPHTFSDGTYVP

KGVWVCGSPHIVHNLSALYANPEVFDGFRFYRMRQLEGRAHHYQMSSPALDYLPFGTGKNSCPGRFFASNQLKIAAAYIL

YNYKLRLNGGASGKKPSNVCMGITCIPNPTAGIELMKRERSEKSLLFH*

>CYP548BE2|889630|Tubbr1_1

MTFLGLAKLLFTSFAVYISSLVVYRVFFHPLARIPGPFLAKITDWYTVYHAYRGDRHLALYRAHEKYGPVIRFAPNLISF

NSASSLRAIYGHTALSRSLQKSQFYSAFPAVKGVHNTHNAISKAEHGRKRRVLSAAFSDAALKSMEDLVLNNVDIFSSKV

NDAFKHGVGVDMGDLFSWLTFDVMGELCFGQSFGMLTEETTRFVTGLISQAAHNHYINGNYLPLTTLKLSKILFPTISRD

RWRFIEHSRACANERMSLGRGYKKDFFYYLLDAKDPETGEGFETRELWGEANVLMIAGSDTTATAMSAAMFYLCRNPQTL

QTLKNEIRSTFTNKEQIVGGKELNDCHYLKACIDEAMRLAPPVPGLLPREVIAKGGIEIDGVYIPEGTVAGTPIYALHHN

PAYFPDPFSFKPERWLSTFTSEEDVEAARSAFTPFSIGPRGCIGKSVAYMELKLTLARLLFEYDCEEVEIEGKGALWKEG

FAMVDGEYRLMDHFTSRKEGPVIKFSER*

>CYP5945C1|890822|Tubbr1_1

MISILEKLKGEETNSVNICDNMAFDVMADLGFGQRPEESMQSGAGDPSYMDFLHGWMRVSTVLTSLRNLCELGAYIPQNT
ESKEFDKMTEKMLGTRQRMGKSRQDIFTHLLDEDKESGVNFTQAQLLTNAQVLMVAGSDTTSVTLTCLFRLLSMHPEKQQ
KLYKEIMGAFPNGEIPTCATTAALPFLNGAVQESLRMWPAVPSGPQATTPPSGYTIAGTFVPGNVEVRIPHMTMLSDPRY
FPRPDEFLPERWTPEMPELVQDKKAFIAFGFGAHSCIGRPLAMNEMRTAAARVIQRFQVQLGESYDDKVFRDEWKDYFTV
KLGPCPLKFVPRKM*

>CYP5093H2|891877|Tubbr1_1

MYGSLVTVWTGRRPTIVIGDPKVACDLLDRRSVIYSSRPRFVVMGELFTNNDSLLTMPHGDKWRKTRKIFHMGLHRRACE
SYKPVQEAESQRLTRDLLLTPEVFGKHLERYASSVMICVAYGRRVDSLEDPIVKKIYDRMAYMSTLNVPGAFWAESFPIL
KLIPDCLAPWKREVKRRAKDSTEMLSRLALDVRDRMKKGDAPASFTKTLWEKREGNPEALSEREIAYATGSLFGAGSDTS
SATLMSFFLAMTCFPRVAAEAQEELDRVVGRDRSPAWSDEPNLPYCRAVIKETLRWRPVAVMGGTPHASIKDDQYNGHFI
PKGATILGNLWAIHHNEKYFKDSHDFIPERYLGSGRIDGIEPYPHRDGHSAFGWGRRICPGKQLAENSLFITITRVLWAF
NISKATDKHGQEITPNIFAYTDGFNSKPQPFQCRIRPRSSGIQQIIEREARLGEQFLDKYKCN*

>CYP663N1|893993|Tubbr1_1

MSLIQAISSLPGGSAAALILLVLSVGILAQRLVKKKDPLPLPPGPPGLPIVGNSFQIPLINPWRKQAEWTKQYGPIYKLK
LGKDTVIVLGTQQAAKDLLEKRSKIYSSRPRTVMAGENVSRGKKLLAHLTGDKWRMHRRLQGQVINQNIANKYKGFQNLE
SAQLIKELADRPEDFFDSFHRYNSSVIFAMAYGKRMPRGDEEDVLAVDEITENFLYSARLGTWIVDSFPFLNYLPTFMAP
WKRIGDNFYNWAEKMHTFNRNEALQRKGWNWTKEIARMKEAQNVSPLELAFMVGFLYEAGSDSTTIALEVFILALLKHPE
VLKRAQEELDRVVGPDRLPTFEDRDNLPYVRNCVNEVLRWRSPSAGGVPHVVEEDDEYMGYRIPKGAIVVGNLWSIHQDP
EVYPNPTKFMPERWDDEENVHYGFGFGRRACPGRHVAINSLFVNYSRILWGFNVEHAKNPDGTVIPVDEWDMTQGFMSRP
VRYKASITPRDAKRVEVINQAWKAAEVQLQDMSKILRMEDF*

>CYP50043A2|896074|Tubbr1_1

MAAATGWYAGYWDLYLGGQMVNHLVDLHKEYGPIVRCGPNHLHFSSPEVYSKIYNTTNKLTKDPNLYHSFGADDSVFTVL
DPAIARNRREVISPMFSRRMVLSLQPLISEKIRKLCDKLSSYANRDEAADVASGFRSASIDIITQYCYNECLDSLDVEGF
KHDMLLSIKGTSEAFWVAKYFPMIHWILNLPTNVTLRLMPELKGFLALKDSIEEQVKRYTENPSLLEKSSHPTVYQRFLD
PDVKGGIPSASTLIDEAQNLFFAGSDTVGTALSFGTYHILTTPGLQEKLFAEICQVWPVLEKEPTYDQLEKSAYLTAIIK
ESLRISHGIVTPLTRVVPTPGMTIQDQPIPGGTVVSMDSPTVHLNPTIFPSPATFLPTRWLDSNAKDLEKYLVAFGKGSR
ACIGSNLAWAKMYIGFASVFRRFEMTIWETSKEDMAWMDCFTPITRGDLKVKFRVRGE*

>CYP5959A3|918853|Tubbr1_1

MGVFVQLIDRVEDKKLYGYSLFSTSLALLFFGIFLLLKKEWSTHSGTKFPIVGIESPGYIGIVKARLSFVANGYKIVRNA
YHKYPGKNFVVTTYSYDKVVLTHDQVKELSNAPDDTISISHAAVETMMGDYTGLDQFVGTTFVEDVVRIKLTQNLGSMNE
AMLEEARFALNTELPECTTDEWRSVEVFTTIIRMVARMSARAFVGFPLCRNEDWLTITMVFTGHVFMTHSKLTCVPKPIR
PIYAYIRNLVKDITEDKRKAESLLAPVIRRRLEEERLAEKNGTTFQKPNDMLQWLSDRVDPKHKTTKGLSELQLVLSLAA
IHTTSISFLNAIFDLLAHPECIQPIREEMEAIITANNGVLDRVAMRKMKKTDSFFKESMRGKIGLFSFNRKVLKSVTLSD
GTHLPKGTLITAPTSMFSFDPDFVEDPETFDGFRWYKKSLEADSSNNYWATTSSNDLAFGHGKHACPGRFFATERMKTIL
IFILLQYDVKYPEGQSRPENIDHGEFSYPDTTRQLLFKKLPGPKKYSFL*

>CYP51F1|919458|Tubbr1_1

MGVLSTLLVPMGPYYEELMKLGGPALCGIGFITFVVLSVIINVLQQLLFKDPTKPPMVFHYFPFFGSTVMYGMDPYKFFF
GCREKYGDVFTFVMLGRKMTATLGPKGNDFVFNGKLSEVSAEDAYAHLTVPVFGEGVVYDVPNHVLMEQKKFMKFGLTTE
NFRSYVPLIVDQVEEYIKKSKFFKGAKGSVPLSEIIPELTIFTAARTLQGKEIRDALDGSFAKLYHHLDSGFTPMNFLFP
WFPFPQNKRRDHAQRTMARFYMDKINKRRATEKDDVGQERSDMMWNLMSCSYKDGRKVSDKEIAHMMIALLMAGQHTSMA
TITWMLLHVAAQPKIVARILAEQKRVFGDELAPLTYEKLVLECTFLGQILRETLRMHPPLHSILRKVKSPMPVDGTNWVI
PKGHYLLAAPGVSAMDQKYFKEPDHFDPNRWEDQKPEEEAEKFDFGFGLISKGTASPYLPFGAGRHRCIGEQFANVQLMT

IMATFVRNFEMQRPGGGNDVPAPDYSSMIALPTPPSIIEWVKRDP*

>CYP6592A3|920080|Tubbr1_1

MNAASTSILRHEYTALSLIDSFLLSLVLLPILVTVVYNRYYHPLSPFPGPFLASIWSGWRDWEHYSNLEDQEPLLHERYG

PIIRVSPNTISVADPDYLKEIYTRTGCTERWLSFPHNGVVDVPSAMLHAELRKRSIPGYYVGTARSWEGIVDERVREWVG

KLKAEYGGSGVQFGFSEWARYLSVDLVGWVVFGTDMGCTQGGGDNRGRITGSESRLGVSRGLAKFPNIYRTLTSDSSIRN

ILTAKADKEWVGAMRVKVGLLIDAAWKSREEAEARGEEWHLNGEAMSAKTSNGTRLTKEELHSECFLPFIVGQDSTAFAI

TNTLKLLLDNPPVLSALLSELTTNYAQKPLWHIPPWIDIVDLDAKLPYLSAVLRESLRLHPPFPVGNPGMVPSSGITLTH

RNRLYTIPGGYEIRANPIVIGRNKRIFGEDAHCFRPERWLEGSEEEIKRMKGVGFEWGTGSGGCLGKALAQMAVAKAIVM

VLRNFEVELAVVMREGRYGG*

>CYP51070A1|921433|Tubbr1_1

MEFIKPNLGLLQILAVLLILLIVSSFYKKKCNAPLVGIKPGILGWFTTPYLWLQDPISILREGAEKYGKYGKTFRITSPT

YDRIVFSNKEVIQEMRDLPDGVLSFRHGADEMIQSTYTFHKGIVQDAYHVDIIRKNMTEKLSKILPEISKEVEDVFEKIT

DIQNEWTNVNILNMSVGLISRMTSRTLVGIPICQNQQYLESITQFSINVMKVARILDFLPWFLHRITTRFFLKKNTVQKV

IIKHLGGEFETRQEIKRLYPNSPSTTSDAIQWIIDATPPETPILKLIQKLLLFNFVSIHTSSIMLTHALYDIAANTQYQD

PVCAEIQAVLRAEGGWTKQALTKMKKLDSILRESARMNSINIITPIRKVLKSHTFIDGTYVPKGSWVCVYLSQVHYSPEL

YENPEIFDGFRFYKMRQEEGKAQKFQMASPSLDYLAFGVGKNSCPGRFFAANQLKVILAYILCNYKLRLNGGVCGKKPPN

IYSGMVCIPNPTIGIEFKAREGRNKSVMFKHNITC*

>CYP5959A19|925157|Tubbr1_1

MGIFIQIIDYAEDKHLYGYSIFGTTLALCSLGLFLLLFKEWSIHGPTKLPIIGIESKGYFARIKARRKFISDGFQIVKGA

YYKHYGKNFVITTNSYEKVILTHAQVKELSNAPDDVISFSHASAQSLMNEYTGMSRFMLTTYVQDATRIKLTQNLGNMLS

AILEETQFALNAELPGSTDEWTSVDFFPTALRIVARVGSRSFIGLPICRNEDWLDLSISFTQDLISTASNLSTVSKLFRP

FYAYMWNSAKTIRAHKLKAQTLLAPTIEKRFEEKRLAEENGTVYQGPNDLLQWLIERVEPHHKTVDALSELQLLVGLASV

FATARSVVNIIYDLAEHQECIQPMREEMEAEISANGGVLDRGVLRKMKKTDSFIRESMRTGTFLLSFNRMVMKNVTLSDG

TYLPKGTLVAAPALMFASDPDFIEDPETFDGFRWYKRSLETGDGAQKGNGCATTSSNNLIFGHGKHACPGRYFAVDEMKA

ILTFFVLQFDIKYPKGQSRRPANIHMGELATPDPSQKILFKKLPGPKKFSFL*

>CYP6220A2|944385|Tubbr1_1

MTESVHTHNKPSIVLLSHCPPPTRMIDSEYFLNGISNTEYLTTAIRAFGAIIVWKLISAIFFSPLSHIPGPLLTAITPHY

INFLSALNRRTVGTYSLHKRYGPIVRLSPTEISILSPQAIREVYSSPHYTKYTPLYSIFTHFGAQNAFTSCTREEHGWRR

KAVSESYSLSSVLKDEAATGKVLKAVKDYLSFVENDRRVDIYIANTFYATDVVTGKIFGLEASMKTLAGNEAHREIVLGH

YARTRRTQVWMYIEFPLVMNLFEWLAFYTGRVWSWIHGEEIESWEMVSQVQKWGWDAYMDAKSNSREGTVAGRLAKLVQE

DSCAEEVWDDRGAVSEVMDQMLAGMDTTGDTLSFLMYQISLPESRGVQKRLHSELLDAFPKSAEIPGPGWPHSMDIQSLS

HETILKVLNLPYLDAVLKETLRVYPAIPITLPRVVPASTTTDRISGHRNGRILEGKFIPAGTTVGTLAYAIHRDEVFALE

TEQKNKPGDVDSFLPERWLIKGGIGGKSTPDELEQEKQRIRTMEARLWAFGSGGRNCVGRHLSILEMKLLLGTIYSRYQT

QVMPNSGVKITHNRWDQRRTFRDVLPFRGVDGVVIFTPCGC*

>CYP51070A2|962913|Tubbr1_1

MEFFKANLRLLQTGIIAVVILIIHSFSKKKSNAPLVGVKHGIFGWFKTHYNWVHDPVSILKEGQEKYGKYGKSFRITSPT

KDRIVFSNTEVVQELKDLPETILSFRHAGDELVQSAYTFHEGIFQDTYHIDLIRKNMTEKLSNLLPEISDEIQEVLGNLT

NYGNEWTNVNILNMSVELISRVTSRIFVGIPLCHNQEYLDSISQFSIKVTNCARILEFLPWFMCRIVARFFLKKDAVQQV

IIKHLGGIFKTRQEMKKLWPNSPSTTSDAIQWILDTAPLETSILKLIQKLMFLNFASIHTSSITLAHVLYDMAENPEFQD

PVCTEIEVVLKAEGGWTKQGLTKMKKLDSVLRESSRMNGIGLITAIRKVLTPHTFIDGTYVPKGAWVCVLMPQVHYSPAL

YKNPETFDGFRFYKMRQEEGKVHQYQLASPGLDYLAFGIGKNSCPGRFFATNELKISLAYILYNYKLRLNGGVSGKKPPN

VYSGVVCMPNPTIGIEFKAREGRDKSVMFHHDITC*

>CYP6498A1|965067|Tubbr1_1

MGTLLILVTCFGVTITAYTISSIIHLLYNIREVRRSGLPYIILPWHEMNLFHVLGCGFNRELCQYLPKWTYFKIFWRDWG
HHTKFELFEKYGDVICAVSPGGVTIYVGSVEVARQMYERRNDFPKATKVYESVRFYGDNVLTLEGAEWRRHNKYTRPPFS
DAVHKVVWDEGVKQAHAAVNVWNRENSCLNVGRDLRTISMNVLSLSNFGVALPFDHGSETTEHFDRNIPPGHKMSYGKAV
NHVLDNIIPLVIAPKWLLRNGPESLKKIGRSYDELGIYLKELIQIGDKAAASDRKNLLGSLVEASAGDGPEKGSGLTDAE
VIGNAFIFAVAGLETTTGTLHYAIMHLALNPDVQDWLYKDLQEALKDEDQDPSKWEYEKVYPKMATVLCVIHETLRLNAP
HTHIPKWTADKYQPVNWRGKQCLMPPGALAYITTTALHYNPSLWGDTVKGFHPQRWDLRFSSEGWVRTDPATGVKTPITP
SETPRTGTQSAPYCYLRTPVKGAFAPFSDGWRACVGKNFALVEMTAILAVLFRDSSVRIKRQEGETREMADNRGKSAISN
SKGYLTVMIRDDVELEWVGR*

>CYP617Y2|992016|Tubbr1_1

MGKWCSLAALDIIGSSGFGYEFRALESASVSGSPEEKSGSELADAYNTIFNMGGPSRIVAILSMIFPASLVGYLPLKRTR
DVAHAASTIRRVTAQIIAAKKSALAASPEDTESKDILSVMLKSNTYTGSDGESSMRDQMMTFLAAGHETTATSMIWAIHA
LSLPENRHIQSRLRAEIHAAFPSGAPATITYDQISSLKYLSHITSEVLRLYPPVGVTLRVAAEDTSLNGVFVPKGTSIVI
SPFAMNRSVALWGADAEEFRPERWAGGEDGTATVESNYGFLTFLAGPRGCIGNVFAKVEFKCLLAAVIGRFEFEQDGKRE
LVVKGGLTAKPQGGIPVTVREVVWG*

>CYP61A1|1002962|Tubbr1_1

MDEIANSSTFVPPSAVSSAEQPFQTLVEGFETIGKTLEGLSGWQIFFSIVILSITYDQCLPFSLPLSPIPPRPGRYIWNK
GSIVGPSLKIPFMGPFLESVNPKFSEYLAKWNSGPLSCVSVFHKFVVIASTRDLARKVFNSPGYVSPCVVDVAKKILRPE
NWVFLDGRAHVEYRKGLNGLFSRQAMAMYLPGQEEVYDVYFKKWLELSKEGKPIPFMSEFRDINCAVSLRTFVGHYISDE
AVKEISENYYKITAALELVNFPIILPWTKTWYGKKCADFVLNEFAACAAKSRIAMKEGNEPGCTMDSWIKSMIEAKESDA
AKIAAAKGLPQIREFSDMEISMTIFTFLFASQDASSSASTWQFQIMADRPDVMKKIREEQLRVRDGDPYKRLDLDMIDKM
VYTRAVVKEQLRYRPPVIMVPYEVKKSFQITPEYKVPKGAMVVPTLYPALHDPEAYNDPESFVPERWLEGGEAEAAKKNW
LVFGAGPHVCLGQNYAIMNFMGMIGKASLMMDWEHHATPKSEDIKVFATIFPEDDCFLVFKERDPYAPA*

>CYP539A50|1010552|Tubbr1_1

MLFGAVVQSASGLLIAVVVVGVLTSVFKKVRENWKIHKLGGLDEIKRTLVAAKANKTMEFWESRFKTTGSHTVEVASWDP
ENIRAVLASQFHDYGKGERFRQDWKSFLGDSIFTTDGQLWHASRQLIRPQFIRGRVSDLDIFERHVSHMLEYIPRDGAIV
DISDLFYRFTLDSATDFLLGQSVDSLGSPQVEFAHAFADIQKHMSDKSKAGPLRWIFPEGKYRKDLKVLNSFVEPYVEQA
LRMGPEELKSKNEKSYNFLHALAEFTRDKQMLRDQLVAVLLAARDTIAVTLSWTLYEIARQPEIVRKLRMEILERLGPNG
KPTYTDLKEMKYLQHVINETLRLYPAVPFNVRVSLKDTSLPRGSGPDGLDPVGMPKNTVFGYAPLTMQRREDLFGPGVEK
FDPDRWEKWSPKSWQYIPFNGGPRICIGQQFALTEMSYILVRMFQQFGSVESRQAEEQYQICNIVLSPGTGVPVSFKPAG
FP*

>CYP6001E1|85192|Tubbr1_1

MSFLGLFGGGSSSNPNSEYGDESAATKEVTYTGIVEDIKAAGGKIPEDLKLLLETGAQKVSKGPVDDRKLVMERLIGLVA
SLPQNSSNRKKLTSTIIDTLWDSLQHPPLSYVGDKYQYRQADGSYNNIMYPDLGKAGTEYARTVRQDKKLYGAKPDAGLL
FDLLMSRGENFKQNQAGISSVLFYHASIIIHDVFHTNRKDFSKSDASSYLDLAPLYGSNQEEQDLIRTMQDGLIKPDTFS
DKRLLGLPPGICVLLVMYSRFHNYAAKTIKAINENGRFSLPASHATASPEAQAKNLAKLDNDLFQTARLITNGLYVNISL
HDYIRGIANLNHSESTWTLDPRLEIDKTFDGEGTPRGVGNQVSCEFNLLYRFHSAVSKRDDAWTKDFFGKIFPGQDPASI
GMSQLLQGLIIFEKGIPEDPAKRTFGGLKRIGADGSGAFSDDELVKILKESIEDPAGAFGANTVPDILKPVEVLGILQAR
KWQVASLNEFRAFFDLKKHKTFEDINPDPYVANTLRKLYDHPDMVEMYPGMFLEDTKPRMDPGMGLCAPYTVTRAVFSDA
VTLVRSDRHLTLDYTPANLTNWGITEVAQDYDTLGGSKMYHLILNAFPSYFKYNSVYAMQPFYTPTESRKIFDKFGKSNL
YSFDPPAKTASPIPILTHAGLKRVLNDQKNFKVPWGESMEALNNEHDFMLAGDLSSHTQQRNLVGDAIYDVTGSRKQFKD
YTEEITLKLLKREVYQLGKVPFNQVDIVRDIGNLATLHFAASLLYLPLKSDENPNGQYSEAELYKTLTDLTWFVFSDSDP

TKSWEHRREAKKSIDKLGEVMVEEIKKFKTPIGIWDKLTGGAGVRPPPPSLKDYGSNLVRRLLGSGNSAEDVAWMLLWSG

CAFVANSACAFAQLIDFYLQDDNRKHWAEIQRLSTLNTPEADKLLTKYTLEGTRLSNSLGIFRNVDPVDTQAITIKQLGQ

DVVLKKGDKVFVSFVYASKDASVFPEPLEIKLDRPTELYVTHGEGQHQCLGKDINIVQNTYMLKTLAKLRNLRRAPGDEG

KLKFIPKAGGIKLYMNADWSKFTPYPTTMRVQFDGPIVA*

>CYP6001C26|753301|Tubbr1_1

MASNTNGTNDTTNGHSNGYSNGASNGVRGKKSKYATQSKPPPPVAKPTRAAVDSTFEQFASLLHASNRPLPNRFGDGKDQ

RPPEVKPTGVLADINTLRRGGFFWESVGTLWTVLKQKTKGGATDDKTMIMERVIQLTSRLPPTSKVRVALTTSQVDQLWD

SLQHPPLSYLGDGFSYRQADGGYNNIMYPELGRAGSAYARSVKPMTKMPGAPPDAYTLFDSIFSRGPNDEHFREHDNNVS

SMLFYTASIIIHDLFRTNRENPNISDTSSYLDLAPLYGNSVEDQSKIRTFQDGKIKPDSYSEKRLLAFPPGVSVLLIMFG

RFHNYVVENLKAINDGGRFSLKFPRYPNGDDEAARQAKALRQQDEDLFQTGRLVTCGLYINFVLNDYLRTIVNLNRVDTT

WTLDPRFEASKVYNPDGTPAGIGNMVSAEFNLVYRWHSCISKRDDQWTKDFYQSIFPGRDTRGIEMPEFMRGVGRWEESL

SDDPTQRNIAGLERKADGSYHDDDLVKILTESIEDVAGAFGARNVPHILRLVEVLGIEQSRKWRVGSLNEFREFFGLKPH

ATFEDINPDPAVANTLRQLYDHPDFVEMYAGLVAEADKKPMVPGVGIGPTYTISRAILSDAVTLVRGDRFYTVDYTAAHL

TNWGLQEASSDPAIVHGCVGYKLILKAFPNHFKFNSIYAHYPLTIPQENHKIHTALNTVDQFDFERPVYTPPRIPISSYD

ATKQILCDAENFKVTWGAGFDFIMRADFMLSGDKPSNSEQKQFVSNRLYLGGVDWKQQIKQFYEEYTEKLIRKKAYSLGD

AYQVDAVRDIGNIAQTVFAASIFNLPIKSEDHPKGIYTEQELYMILCVMFIVIFFDIDSSKSFPLRQAGFKVVRQYGTLV

EAQVKAINNWSWLQGIWDPLNIRGRNKSSPLKSYGWTMIKRLLESGKSPYDVTWSYIVPTAGASAPNQGQIFAQVLDFYL

EDRNAHHLAEIQRLAHLGTEDAWETIKKYALEGGRLAGTFGLYRRVEPDNIVIEDSGRNIELRKGDMVFVNFVTASRDPV

VFPDPLEVKLDRPEASYMQYGDGPHECLGKAANIIGLTTMLMQFGKLKGLRRAPGPQGTLKYIPKPGGFKVYMKEDWSAY

WPFPTSMKVRFDDII*

Tuber indicum v1.0

>CYP530G1|524103|Tubin1_1

MAIPVFWTAAPGLALGVCLASFIATIIYFAKGTSRGKNFPPGPPTLPILGNMHLVPQERPYLKFTEWAKQYGGIYSIKIA

KQTIVLISDVKILKELYDKRGAIYSSRPLPHIGAEIVCPDQTHIIYMPYGETSRNYRAQYHQFMGPGKVEQILPWQSAES

TLLLKKIATSPERYYEHTMRMATAVILESVFGVLPKDYDDPEVTELWTVQREFSEILALTGPPVDHFPFLKWLPDIVSPW

RIHARNVRAMHRKLYFRLLNLNKTRMEKGERYGTFVEKLIDDKPKHGLSDERIAYVCATLMEAGSDTTASQALDFMMALL

AFPNVLKKAQEEVDRICGTSRLPTLDDRDQMPYIEACVNEVLRWRPPLPYGAPHLLMKDDWYEGYFMPKGTVLFQVQWAM

NMDENVYEKPQDFMPERYIRNRFGTKFDAEKDAEIGRKEQYGFGLGRKICPGQWFARNTLFVLFAKLIWAFDMKVPTDPK

TGELLPLDTDVRTAFMDGLTTTPFKFPIEFKIRSKAHEEAMNTDLVASDKVFAKYGGATV*

>CYP5959A5|550397|Tubin1_1

MGIFVQLVDYVEDKSLHGYYLFGTTLALIFLGIFLLLIKEWSIHSPTKLPIVGVESKGYFARVKARQKFLFNGFQMVRDA

YYKNYGKGFVVSTNSYDKVILTHAQVRELSNAPDDSLSLAHASARTLMAEYTGMNRFVGTGYVRDATKTKLTQNIGNMIG

PLIEETKFALDRELPACTSDEPVPVDIVAAVIRIVARVSARSFIGLPLCRDEDWLAISIAFTGDIFKTAMKLNAIPKLIR

PFYAYIWNSAKIIENHKTKAQSLLTPIVRKRFEEKKLAEKNGEAYQNPNDLLEWLTDLVEPRHKTADALSDLQLAVSLAS

IHTTSSTFVNTILDLAEHQECIQPLREEMETAIIESGGVLDREAIRKMRKADSFFRESMRTRSFLFTFNRMTMKNLTLSD

GTYLPKGTLLGAPNAMFSSDPGFIDNPETFDGFRWYKRSLEAGDTAPKGNGCAATSASSLAFGHGKHACTGRYFAVEEMK

IMLAYFVLQYDIKYPEGQSRPDDIVVGEFSFPDSTQKVMFKRLPGPKKFSFL*

>CYP548BE2|550922|Tubin1_1

MAIFKSSIQSLDRIPITIDMMPFLDLAKLLFTSFAVYISSLVLYRLFFHPLARIPGPFLAKITDWYTVYHAYRGDRHLAL

YRSHEKYGPVIRFAPNLISFNSASSLKAIYGHTALSRSLQKSQFYSAFPAVKGVHNTHNAISKAEHGRKRRVLSAAFSDA

ALKSTEDLVLSNIDLFSGKVNDAFKHGVGIDMGDLFSWLTFDVMGELCFGQSFGMLTEETTRFVTGLISQAAHNHYINGN
YLPLTTLKLSKILFPTISRDRWRFIEHSRACADERMSLGRGYKKDFFYYLLDAKDPETGEGFETKELWGEANVLMIAGSD
TTATAMSAAMFYLCRNPQTLQTLKNEIRSTFTDKEQIVGGKELNDCHYLKACIDEAMRLAPPVPGLLPREVIAKGGIEID
GLYIPEGTVVGTPIYALHHNPAYFPDAFSFKPERWLSTCTSEEEAEAARSAFTPFSIGPRGCIGKSVAYMELKLTLARLL
FEYDCEEVETEGKGALWKEGYAMVDGEYRLMDHFTSRKEGPVMRFSRR*

>CYP6135A17|554221|Tubin1_1

MLASIFLTLALLFVGYACYSLHILVLKVRPLGIPYFVIPFYPSPLPRIFLVPFVNLLINVFNLTHVWFHLLTLDWQVRQR
YEIYRLVGSDLFFTITPWEVILHVADPEMAVEVLSGKGQDGELYPRTEIVGRMMGLFGKNILTAEGAVWRGHRKVTAPVI
GNSLSIVWEESLTQVKEMLQYYDKQEDIKTIHRDFRRVTLGVISHAGFSKVMNWVPLTTTITSGKDGEGSYQKCLNILFE
NFEWTAIAPSWLLEMLPFKASREAGGAANRFRFFTNEWFEEKKAKVELDNKTTLKSSSRVDLMESLVRSSGLDEGSTVET
PLLSKSEVIGNTFIFILAGHETTGHTLANAIYFLAMYPEYQVKLQNQIDSVLGSTNHNETSYDKHFDAFSSGWMAAIMLE
TLRLIPAAIVVSRQNGPTARTFPLSGRGPITLPPDIQLWIELIGLSHNPKYWTVPGKTEAESAISEFRPERWLPKSEGES
MFKPYNGSYIPFSIGARACIGKKFAQVEFAAVMMGLFREMNVEFDLCGGKKSFEEARRECLMQMERYETIITLRPTGEMP
GIKWVRRKK*

>CYP617W1|555391|Tubin1_1

MSTTNILAGCAASTILLSRHTQFGAITTFILLTSLAVTCKLLYRVILWPKLLSPTRHLPGPSSPSFLMGNFALIQKMQSG
GPHAIWLRTIPNSGLLRYLGIFNTERVFPTTPEVLKEVLHTKSYTFIKPTLLTNDIGRILGRKGLLFAEGEEHKVQRKLL
LPAFSHAHIKGLVPGFWSKGVEMTEKVAEVVRASRGAGGEEQGVVVEMGKWCSLVTLDIIGSSGFGYEFRALESASVSGN
PEERSGSELADAYNTIFNVGSPSRIVAILSMIFPALLVRSLPLKRTHDVAHAASTIRRVTAQIIAAKKSALATSKEDAES
EDILSVMLKSNAYTGSDGESSMRDQMMTFLAAGHETTATSMIWAIHALSLPENRHIQSRLRAEIHAAFPSGAPATITYDQ
LSSLKYLSHITSEVLRLYPPVGVTLRVAAEDTSLNGVFVPKGTSIVISPYAMNRSAALWGADAEEFRPERWACGEDGTAT
VESNYGFLTFLAGPRGCIGNVFAKVEFKCLLAAAIGKFEFEQDGKRELVVKGSITAKPRGGMPVTVREVVWG*

>CYP6608A1|34773|Tubin1_1

MPSTLLMSLAPSLKSLVVLAFTQMLPLALLALVASCVYSRYASPLKNVPGPALASFSRIWLNVGLVARGNLHGGIMDAHR
KYGPVVRVGPNKVYFSDPALIPEIYGVGTGYRKSDLYMMSDPEPGHGSTFSERSAEKHAVLRRRVGPAYSNSSVFQMESY
VDALVSAWISALSSEFTVAKTSLKSCDFARWSQFLTYDVISELAFGKAFGFIATRSDYKQYIASLQQALPMIVTMSYFEE
LVKFMTLGWVRKLTTPSIKDETGYGNLTKTATDLVRERWAKGGVGERRDMLQQFYEEGMTEKEAMIDSTLILVAGSDSTA
TTIRAAVMFICSDPSVYRTLKSELAANIPRTSPPSVVSFSVARSLPYLSACIKEALRLFPPGAGAMERIVPVGGATLSGY
FIPENTVVCLHPWPVCRDKVFGEDVDSFRPERWLEEKDEKRLVKMEKTLDFVFGAGPHYCLGRQIAYLELYKSIAELFLT
FDIGLSNPMRPWKSCEYGFFLQRDMMVKIKRVDG*

>CYP6271A4|560945|Tubin1_1

MALDAVIAYARELAPASISRGAIAATVLGVWLLNRIWLVFYRLYLDPLSKFPGPKLAAATSLYEMYYDIAQDGAFTWKMD
ELHKKYGPIVRVSPYSLRLRKSSAYHEVHRMGTPFVKDVRFYGQFKIYNSVFAIIDTELHRQRRALLNPVFSRKAILDSE
PVIREKLNHLCRRMKEHEVQDKLLNCYTAFAAFTVDIITKFAYDKSYDILSLPDFDCRIGAAFDSQLEFLLVMKHFPILA
QILHSLPIAVINRLMPSGASFAELELDAKLHLIDLISRMRDGTMKNSTAHRSIFGGILTEHPDLKADPTELAQEAMSTVA
AGVHVTRWTLCVGLLEIARDSLIQTKLYEELKMACPDINADFSHVQCEKLPYLRGVILEVLRLSYGAIGPLPRRVPKDGA
VVGGYHLPGNSTIEMDSYSIHHDEDIFPDSRRFWPERWLTPESKQKEKFVTSFGAGPRQCLGINLAMCELYLATATIFRR
FEIDIGARGPERMKFTEHMVVVPRGEPLQCKVISRKE*

>CYP52AU2|564238|Tubin1_1

MFIDSIPELDFHGYYFAAGIFFGGIFCMLVWSIINRGTVRTAANGQKCQSPPVLKSKLPFGIDSMLMLMRSVREFRLPVA
LTELHDQMGHTYASMLFGMRAIRTRDPRNIKAILATQFEDYGLAQERRAHRSLLGSMGIFAVDGVSWSHSRALLRPSFDR
TNVSDLGQLEGFMKIFFSRINELPIQSFAHARAVELQELFQCLTMDSSTNFLFGNPIGALASKKYTLGNGEMTFDQAFDI

AQYGLAMRAPLSSFYWLYNGREFRKACSTARAQAALYVSRTLRKLNLAEKSEGKKYVFLEELAERTQDPQVLQDQVLSVM
LAGRDTTAALLSWTFLCLAKSPEVFKKLRAEISEVVGVDEDARLPTQAELRGMVYLRWIIQEVLRLYPPVPINARRALRA
TTLPYGGGPDGNSPISIRKGERIVYSAFSLHRRTDIYGPDADEFRPERWGEEALRGIGWSWLPFNGGPRICLGQQMALTH
ASYFITRIMQVYKEITPQDSALEGDAYDTKLVMASGRGVHVFLS*
>CYP50026A1|568018|Tubin1_1
MTEALATNLLENGNTTGKRNMSHDLLQCRNDDNTPLSKKELVGEMLAFTTAGSDPTAYEISAILDRICRHGEVREKLLQE
LRAVGELEQSSAEGVVTYAQTLRLPYLLAVVKETMRLNPAFQGQFSRVAPEGGCGGEVGGLEVLPGVVVPGRVWLSINTY
VSQRDKLVFGEDAEEFKPERWLPIGGDRYHAMSKYLSVFGYGSTACMGQHLASQKINKTVVEILRRFNVELKDPKTPLKE
RNIIQMFISDLFMTFTEREKKI*
>CYP567U1|568943|Tubin1_1
MMATLHRFEGAALLVRQAVKDVPAWKLCAYVTLLFPLYAITMGIYNLFFHPLRNFPGSKKAALSNIWYSYIWLSGRYPHI
IHALHEKYGSVVRVAPNQLSFNTGSSWKDIYTNYGNRKTFRKNTLYDRDKLDPVTNIAREPDPVKHNQIKKLFSHAFSMK
SLTEQEPIVQEYVDLLVSQIGKHGTGEDGLDMTKWYNYCTFDIIGDLAFGEGFDATKEGKPHFWISLILDSVYAISFINV
TGRFPWLSKIVPSITPAEVVERRQRHINYGHDKVNRRVKSDNKRKDFLTNVLENHRDQISDAELYSNAQVFIVAGSETSA
TTLSGLTYYLLRNPRTYQRLVSEITSSFTSYSEINSVTAGRLEYLGAVINETLRMYPPVPIGLPRLSPGETVDGQFIPKG
AMVSTSPWASGHCSKNFHDPWTFKPERWLDGECEEKDIREASQPFSLGQRACLGRNLALMELKLIICKMLFTYHLELLDT
KLDWERDSTAYVLWVKPELKVRLHRR*
>CYP5945C1|575772|Tubin1_1
MISILEKLKGEETNSVNIYDNMAFDVMADLGFGQRPEESMQSGAGDPSYMDFLHSWMRASTVLTSLRNLCELGAYIPQDT
GSKEFNKMGEKMLGTRQKMGKSRQDIFTHLLNEDKESGVNFTQAQLLTNAQMLMVAGSDTTSVTLTCLFRLLSMHTEKQQ
KLYEEIIEAFPNGETPTCAATAALPFLNGAVQESLRMWPAAPSGPQATTPPSGYTIAGTFVPGNVEVRIPHMTMLSDPRY
FPRPDEFLPERWTPEMPELVQDKRAFIAFGFGAHSCIGRPLAMNEMRTATARVIQRFQVQLGESYDDKVFRDEWKDYFTV
KLGPCPLKFVPRKM*
>CYP6220A2|584274|Tubin1_1
MIDSEYFLNGISNTEYLTAAICAFGALIVWKLISAIFFSPLSHIPGPLVTAVTPHYINFLSALNQRTVGTYLLHKRYGPI
VRLSPTEISILSPQAIKEVYSSPHYTKYTPLYSIFTHFGAQNSFTSCTREEHGWRRKAVSEPYSLSFVLKDEAATGKVLK
AVKDYLGFVESDRRVDIYNANTFYATDVVTGKIFGLEASMKTLVGNEEHREIVLGHYARTRRTQVWMYIEFPLIMNLFEW
FAFYTGRVWSWIHGEEIEGWEMVSQAQRWGWDAYMDAKSNSREGTVAGRLAKLVQEGGSGEEAWDDRGAVSEVMDQMLAG
MDTTGDTLSFLMYQMSLPESRSVQKRLHSELLDAFPKSVEMPGFGWPHSMDVQTLPHETILKVLNLPYLDAVLKETLRVY
SAIPITLPRVVPASTTTDRISGHRNGRILEGKFIPAGTTIGTLAYGIHRDEVFAVETKQKDEPGDVDSFLPERWLINGGI
EGKLTLDQLEQEKQRIRKMEARLWAFGSGGRNCVGRHLSILEMKLLLAAIYSRYQTQVTPNSGVKITHNRWDQRRTFRDV
LPFRGVDGVVTFTPYEY*
>CYP539A50|589200|Tubin1_1
MELWESRFKTVGSHTVEVSIMGTRVIFTEDPENIRAVLASQFHDYGKGEPFRQDWKSFLGDSIFTTDGQLWHASRQLIRP
QFIRGRVSDLDTFERHVSHMLEYIPRDGAIVDISDLFYRFTLDSATDFLLGHSVDSLGSPQVEFARAFADIQKHMSDKSK
AGPLRWIFPEGKYRKDLKVLNSFVEPYVEQALRIGPEELKSKNEKSYNFLHALAEFTRDKHMLRDQLVAVLLAARVIICL
FAPNDTIAATLSWSLYEIARQPEIVRKLRMEILERLGPNGKPTYTDLKEMKYLQHVINETLRLYPAVPFNVRMSLKDTSL
PRGAGPDGLDPVGMPKNTIFGYAPLTMQRREDLFGPGVERFDPDRWEKWSPKSWQYIPFNGGPRICIGQQFALTEMSYVL
VRMFQQFGSVESGQKEEQYQNCNIVLSPGTGVPVSFKPAGFP*
>CYP5959A20|599398|Tubin1_1
MGIFIHLIDYAENKNRYGYSVFGTTLALLSLGLFLLLIKEWSVHGPTNLPIIGVESPGYFARVKARQRFVLNGFQMVRDA
YYKHYGKNFVITTNSYEKVILTHAQVRELSNAPNDVFSFSHASAQLLMDEYTGMARFVETQYLQDATRIKLTQNLGNMLG

AQLEETQFALDAEIPGSATNEWVSVDFFPTGLRIIARVSSRAFIGLPVCRNEDWLKLAIAFTEDLMATASNLGSISKLFR
PFYAYMWNSAKIIKSHKRKAQTLLAPTIQKRFEEQRLAEENGAAYQGPNDLLQWFIDLVEPHHKTVDALSELQLLVGLAS
VFATARTLVTMIYDLAEHRECIQPMREEIETLISENGGALDRGVLRKMRKTDSFLRESMRMGTFLLSFNRMAMKNVTLSD
GTYLPKGTLVAAPALMFSSDPDFVEDPETFDGFRWYKKTLEAKAGALMGNGSATTSSNNLAFGHGKHACPGRYLAVEEIK
TILTFILLQYDIKYPDGQSLPPNIQIGELASPNRTQKIMFKKLPGPKKFSFL*

>CYP51070A3|599456|Tubin1_1
MEFVQANLIICQAVGVLLATLIISSFYKTTSNAPLVGVKPGILGWFTTPYHWLQDPVSILKEGEEKFGKYGKTFRITSPA
QDIIVLSNPEVIEELRDLPQSILSFRPAGDEILQSTYILHEGIIQDAYHVDLIRKNMTERLSKILPEIAKNVEEVFEDIT
DIKDEWKNVNILNMSVGLISRVTSRIFVGLPICQNPEYLESITQFSVSVMKCARIMDYLPWFLHWIVAQIFLKKNTVQNV
IIRYLGDEFKTRQAIKKLSPNSLSSKSDVIQWILDATHPETPILRLVQKLMFFNFAALHTSSITLTHALYDLAANPEFQD
PVCTEIQEVLKAEGGWTKQALTKMKKLDSVLRESSRMNGINIITAIRKVLKPHTFIDGTYVPKGAWVCAYTSHVHYSPEL
YDNPETFDGFRFYKMRQEEGKAHLYQMASPALDYLAFGIGKNSCPGRFFAANQLKIALAYILSNYKLRLNGGVCGKRPQN
VYSNIVCMPDPMIGIELKARKGSDKSAMFQHNLTN*

>CYP51070A4|599458|Tubin1_1
MQEVKENLELLLVVAAILTGMILYYLSKKKSNAPLVGIEPGILGFIKTPYNWIRDPIALIKEGAEKYGNFGKTFRIATPR
RDIIVASSQEVIQELCNLPTSIVSLRHAGEELFQTAYTFHDGILQDAYHIDVMRKNMTAKLSSLVPESVDEIKNAFEELT
DIKNEWTNINVLDISSGVISRATSRTFVGLPLCRNTEYLDNITQFSINVAKTANIVDLLPWFFRRTAVRFLLRKNASEDI
LMKYLAREFETRKIIKSSLTESPISTISDAIQWILDATSLETPIMKLVQRLMFFNFASIHPTSITLAHTLYDIAANPEIH
DSICTEIDEVLIAEGGWTKQGLTKMKKLDSVLRESSRMNGINVFSVIRKVLTPYTFIDGTHVQKGDWVCVALPIIHYLPE
HYETPETFDGLRFYKMRQQEGKAHQYQMASPALDYLIFGAGKNSCPGRFFATNQLKIAVAYILYNYKLRLNGGVGGGKPP
NFYSGISCIPNPAVGIEMKAREDRRKSATDYSHTVS*

>CYP61A1|88412|Tubin1_1
MDEIANSSTFVPPSAVSSAEQPFQTLVEGFESIGKTLEGLSGWQIFFSIVILSITYDQCRYIWNKGSIVGPSLKIPFMGP
FLESVNPKFSEYLAKWNSGPLSCVSVFHKFVVIASTRDLARKVFNSPGYVSPCVVDVAKKILRPENWVFLDGRAHVEYRK
GLNGLFSRQAMGMYLPGQEEIYDVYFKKWLELSKEGKPIPFMSEFRDINCAVSLRTFVGHYISDEAVKEISENYYKITAA
LELVNFPIILPWTKTWYGKKCADFVLNEFAACAAKSRIAMKEGKEPGCTMDSWIKSMMEAKESDAAKVAAAKGLPQIREF
SDMEISMTIFTFLFASQDASSSASTWQFQIMADRPDVMKKIREEQLRVRDGDPHKRLDLDMIDKMVYTRAVVKEQLRYRP
PVIMVPYEVKKSFQITPEYKVPKGAMVVPTLYPALHDPEAYNDPESFVPERWLEGGEAEAAKKNWLVFGAGPHVCLGQNY
AIMNFMAMIGKASLMMDWEHHATPKSEDIKVFATIFPEDDCFLVFKERDPYAPA*

>CYP6136A1|617465|Tubin1_1
MFLNLLGRAVYGLMQHKLYSLAGILTLTLAYWTHAYLTSPFRRQNIPGPFLGKFTNAYRWYYVMRHTWHRDLMDLHKKYG
PVVWIAPNDISVSDPSLRNVIYGFQNHKKDFTFFRKSHSYETGSINQDFSFIFEQDPEKARLGKYYMSHFYSEQGLLNLE
ENFDKAVNELIEGLDKHHARTGTPCKMVDWAELFALDLVAQITIDQSAGFCLAGKDVNNAAYGMRVIIKTVGALMPLPWV
LSATSRAIRQNLLLRFLINLYRNVLLLPTFTFDTGIADLKTLGEKNPKHLLAKFYNAQSKMREHYPHGNQAEGTTIQIFN
LIAGAVGAVPHTQVKLIHELTLHPEILQKVREELATTDNTFHLEDFIRHNGRQNKYPIFESAVREAVRLSPAVAFSLSRK
VPPSGCQLHQYHIPPGYNVGMAAYQVNYDEGYFGPDVAQFRPERWLEDHPTEMLEGEKRSMKNYIEAGWLSFGSGGRVCI
GRHLAMFMMMKFTAAVVREFDIRVVKQPVEHHTLITEMLGMEVLLSRKCAVRVGQHDG*

>CYP5959A7|625847|Tubin1_1
MGIFIHLVDSGEVKNLYGYPLFGTTLALLSLGIFLLLFKEWSIHGPTNLPIVGVESPGYLARVKARRKFVSHGFQIVRDA
YYKNYGKNFVITTNSNEKVILTYDQVKELSNAPDDTVSMSHSSAQTMMAQHTGLSGFVGTHYVREAVKAELNKNLGNMIG
DLLEEAQFALRTELPEFTTDEWVSVDIPAVALRAVARISGRAFIGLPLCRNEDWLALSIAFTTDVFQEVTKLTPVPKLLR
PFFAYIWNSAGSVKTNKRKAQSLLAPVIQRRLEEEGLAEKNGTVRQNHRDLLQWLTARVTPDHKNVNALSGMQLMTGLAS

IHTTGTGFTNMIFDLAEHRECIQPIREEMETLIAANGGVLDRATLRKMRKTDSFLRESFRTKLSLLGFNRMIMKSMTLSD

GTYLPKGTLVAAPAAMFSSDPGFVEDPEVFDGFRWYKKSLGVADRAVDNNNASNTSPTNLIFSYGRHACPGRYFVVEVMK

IMLAFMLLQYDIKYPEGQSRPPNIRMGEFSYPDRRQKILFRKRPGQKKFSFL*

>CYP5959A21|634116|Tubin1_1

MTLFVQLMEYADNSNIYGYSVFGTALALLSLGVSLLLVKGWSVHGPTNLPIVGVESPGYFARVKARQKFVLNAFQIVRDA

YYEHYGKNFVVTTNSYDKVILTHAQVRELSNVPDDVLSFAHANTQLLMGQYTGMNRFVDTQYIQDAMKIKLTRNLGNILG

AQLEETQFAMDAELPGSAPNEWVSVDLFPTAIRIVARVSARSFIGLPVCRNEDWLKLAITFTEDLISTASKLGAISKLFR

LFYAYMSNAVKTIKSHKKKAETLLAPTIQKRFEEQRLAEENGAVYQGPKDLLQWLIDRVEPHHKTVDALSELQLLIGLAS

IFATAGTLVGMIFDLAEHRDCIEPMREEMEAGISENGGVLDRGVLQKMRKTDSFLRESMRMGTLLLSFNRMAMKNVTLSD

GTYLPKGTLIAAPALMFASYPDFVEDPATFDGFRWYKKTLEAKDGAPKGNGSATTSSNNLAFGHGKHACPGRFFAVEEIK

TILTFILLQYDIKYPEGQSRPPNLRIGEFAFPNRTQKIMFKKLPGLKRFSFL*

>CYP50043A2|636299|Tubin1_1

MAAVTGWYAGYWDLYRGGQMVNHLVDLHKEYGPIVRCEPNHLHFSSPEVYSTIYNATGKLTKDPNLYLSFGANDSVFTLL

DPTIARTRREVISPMFSRRMVLSLQPLVSGKFRKLCDKFSSYADRDEAADMASGFRSASIDIITQYCYGECLDSLDVEGF

KHDILLSIKATSEAFWVVKYFPMDWILTLPANFTLRLMPELKGFLAIRNNIEEQVKRYMKNPSLLEKSSHPTVYQRFLDP

DVKGGIPKASALADEAQNLFFAGSDTVGTALAFGTYHILTTPGLQEKLFAEICQVWPVLEEEPTYDQLENSAYLTAIIKE

SLRISHGVVTPLTRVVPASGMTIQDQPIPGGTVVSMDVPTVHLNPNIFPSPDTFLPTRWLDSNAKYLDKYLVAFSKGSRA

CIGSNLAWAEMYIGFAGVFRRFEMAVWETSKEDMEWMDCFTPITRGDLKVKFKVRGE*

>CYP567Y4|641179|Tubin1_1

MSPEHVSYLYTLVMGARGIVAVLVILGAVFVLGRVFYNVLLHPLSKIPGPKLCAATDVTYHFLSGRYPFYIKSLHDRYGT

VLSFSSASSWRDIYGHSYSRKQFPKSHFYSFGARHLISERDPQKHSEMKRKLSHGFSAKALLEQEDIVQGYVDKLTGQIN

VYATGPEGDEMVKWHNFFTFDLIGDLAFGESFGSLNDAKPHLWVSLLLGNVRAIAWWSVARWFPLFENAMEMRIKHSEYR

RKMIIQRMDTKTTRRDLLSGQFGPNCPGMTIPELSGQASTIITAGSEATETFLSGTTYHLLKNPRVYKLLFGEIRSAYKT

YEEITDTHAAKLKYLSAIIDEGLRIYPPVAIGTYRESPGETVDWIYIPKGVELSASPWSTCRSPENFHDPEEFKPEQWLD

PNCTDKKHTNQPFSLGSRACIGRNLALKEIRLVLSKMFWVYDMELVNKDADLNRDSTSFVLWDKPDLWVRFTRRREVQVP

VLDRE*

>CYP51F1|130439|Tubin1_1

MGVLSTLLVPMGPYYEELMRLGGPALCGIGFIAFVVLSVIINVLQQLLFVDPTKPPMVFHYFPFFGSTVMYGMDPYKFFS

GCREKYGDVFTFVMLGRKMTATLGPKGNDFVFNGKLSEVSAEDAYAHLTVPVFGEGVVYDVPNHVLMEQKKFMKFGLTTE

NFRSYVPLIVEQVEDYIKKSKFFKGAKGSVPLSEIIPELTIFTAARTLQGKEIRDALDGSFATLYHHLDSGFTPMNFLFP

WFPFPQNKRRDHAQRTMARFYMDKIKKRRAIEKDDVGQERSDMMWNLMNCSYKDGRKVSDKEIAHMMIALLMAGQHTSMA

TITWMLLHVAAQPKIVARILAEQKRVFGDELAPLTYEKLVLECTFLAQILRETLRMHPPLHSILRKVKSPMPVDGTWVIP

KGHYLLAAPGVSAMDQKYFKEPDRFDPNRWDDQKPEEEAEKFDFGFGLISKGTASPYLPFGAGRHRCIGEQFANVQLMTI

MATFVRNFEMQRPGGGDDVPAPDYSSMIALPTPPCIIEWVKRDP*

>CYP512CQ2|342158|Tubin1_1

MKDQSMETVMNNDTGSRSGWFKAALEGVLLKRGVDRGVVLGGLVLWMIGWMVWKSYSLRVNVDAVGDSPGPFGGWRAMFK

YFDHGSDWITEGYKKYSPTGKTFKVPTIARYIVFPTSPKLLEEMMAEPEHILSFDEALTEKVAVEWTLHPSIKYDTYHLK

LIRTKLTQRLSIVLPEVMDELTLAWEESTNIGKEWTKVRVWDVMLQIVARTINRMFVGVPLCRDQEYLDNVIQYTVKVVK

AGAILDILPRIFRAPLTNLIMQKSHHYAMMRKHVGHIFSERKEKMRELGSEWKDRPDDLIQWILDLAGDGKASSEEELIF

RLLFMNFASIHTTTSTLVHALYDIAANPELQPPLHAEITGALSKNGMSKQSLTKMKKLDSVIRESQRLNTITTITMMRKA

LVPYTFSDGTHLPVGTWVAAPATAIHLSASIPNPTVFDGFRWERMGAEDQASGKVGKHAAVTTSFEQLAFGHGKHACPGR

FFATNELKILLSHVIERFEFRCLEGKRPKSRFFGVACLADPDGVVEFRMR*

>CYP663N1|363449|Tubin1_1

MAGENVSRGLRIALLQRGDKWRIHRRLQGQVINQNITNKYKGFQNLESAQLIKELVDRPEDFPDSFHRYNSSVIFAMAYG

KRMPRGDEEDVVAVDEITQNFLYSARLGTWIVDSFPFLNYLPTFLAPWKRIGDNFYNWAEKMHTFNRNEALQRKGWNWTK

EIARMKEAQNVSPLELAFMVGFLYEAGSDSTTIALEVFILALLKHPEVLKRAQEEIDRVVGPDRPPAFEDRDNLPYVCNC

VNEVLRWRSPSAGGVPHMVEEDDEYMGYRIPKGAIVVGNLWSIHQDPEVYPNPTKFMPERWDDEENVHYGFGFGRRACPG

RHIAINSLFINCARILWGFNVEHAKNSDGTVIPVDEWDMTQGFMSRPVRYKASITPRDAKRVEVINQAWKAAEVQLQDMS

KILRMEDF*

>CYP5959A3|416922|Tubin1_1

MGVFIQLIDSVEDKKLYGYSLFSTSLALLFLGIYLLLKKEWSTHSGTKFPIVGIESPGYIGILKARLSFVANGYKIVRNA

YYKYPGKNFVVTTYSYDKVVLTHDQVKELSNAPDDTISISHAAVETMMGDYTGLDQFVGTTFVEDVVRIKLTQNLGSMNE

AMLEEARFALNTELPECTTDEWRSVEVFATIIRIVARMSARAFVGFPLCRNEDWLTITMVFTGHVFMTHSKLACVPKPIR

PIYSYVRNLVKDIAEDKRKAESLLAPVIRKRLEEECLAKKNGTVFQKPNDMLQWLSDRVDQKHKTTKGLSELQLVLSLAA

IHTTSISFLNAIFDLLAHPECIQPIREEMEAIISANNGVLDRVALRKMKKTDSFFKESMRGKIGLFSFNRKVLKSVTLSD

GTYLPKGTLITAPTSMFSFDPDFVEDPETFDGFRWYKKSLEAEGNNNYWATTSSNDLAFGHGKHACPGRFFATERMKTIL

IFILLQYDIKYPEGESRPENIDHGEFSYPDTTKQLLFKKLPGPKKYSFL*

>CYP5959A20|479174|Tubin1_1

MGIFMQLKDYADDKNLYGYSVFGTTLALLSLGLFLLLIKEWSVHGPTNLPIIGVESLGYFGRVEARQKFVSNGFQMLRDA

YYKHYGKNFVITTNSYDKVILTHAQVRELCNAPDDVISFSHANAKMLMDKYTGMARFVETQYLQDATRIKLTQNLGNMLG

AQLEETQFALDAELPGSATNEWLSVDIFPTILRIVARVSARSFIGLPVCRNEDWLKLAITFTEDLIATASNLGTTSKLFR

PFYAYMWNSSKTIKSHKMKAQTLLAPTIQKRFEEKRLAEENGAVYQGPNDFLQWLIDRVEPHHKTVDALSELQLLIGLAS

VFATAGTLVGMIFDLAEYRECIQPMREEMEAAISENSGVLDRGVLRMMRKTDSFLRESMRMGTLLLSFNRMAMKNVTLSD

GTYLPKGTLVAAPSLMFSSDPDFVEDPETFDGFRWYKKTLEAKDGAPMSNGSATTSSNNLVFGHGKHACPGRYFAVEEIK

TILTFILLQYDIKYPDGQSRPPTIRIGELASPNRTQKIMFKKLPGPKKFSFL*

>CYP6220A3|482029|Tubin1_1

MIDSGYFLNGISNTGYLTAVICSFGVLIVWKFISAIFFSPLSHIPGPLVTAITPHYINFLSALNQRTAGTYSLHKRYGPI

VRLSPTEISILSRQAIKEVYSSPFYSKYTPLYSVFASFGAQNVFSSSTREEHGWRRKAVSESYSLSFVLKDEAATGKVLK

VVKDYLSFVESDRRVDIYNANTFYATDVVTGKIFGLEASMKTLAGNEAHRKIILGHYARARRTQVWMHTEFPLIMNVFKR

VTFYAGMVRSWVRGGETVSQIQQWGWDAYMDAKSNSRVGTVAGMLARLVEKGTGSSDQGVWDDRGAASEAMIQMLAGMNT

TSDTLSFLMYQMSLPESMNVQKRLHSELLDTFPKSAEMPGLGWPHSMDIQHLPRETILKVLNLPYLDAVLKETLRVYSAI

PITLPRVVPASTTTDRISGYGDGSTVEGKFIPAGTTIGTLAYSIHRDEVFAVETKQKKKPGDVDSFLPERWLISGGIEGE

LTPNELEQEKQRIRTMEARLWAFGSGGRNCLGRHLSILEMKLLLATIYSRYQTHVTPNSGVEITHNRWNISTTLRDVQPF

LGVAGVITFTPYEL*

>CYP52AV2|489268|Tubin1_1

MELAFKPLFLLATAFYLLLKLIAYLIIAHQNRKFAKSHNCLPPRRLPSSFLGLPNWVHIMRAAKRGEALEHIVNRYSTYG

NTLKGRVLIGTTIGTIEPENIKAILATSFKDFSLGPERHDNFYPLLGDGIFTLDGAGWEHSRANLRPQFSREQVSDIEAL

EVHVQRLMDRLPGADGEAADLQPLFYCLTLDSATEFLLGESVDSLLSPKLIPTGTISRGKEEMSFAQAFNVSQGYLIQRA

RLRGLYRMVNPKRFRDANAIVHRLVDRYVDMALNPEKRTRKISENKYVFLDAIAAETKDPKYLRDQILNILLAGRDTTAG

LLGFTFWLLARHPHVYQKLREKILEAFGTGGDGEGKRPSFSALKDVTYLRYVLNETLRLYPSVPLNGRTAVRNTVLPRGG

GEDGLSPVFIPKGQRVDYTCYGLHRRKDLYGEDADAFRPERWGEGVGRGWDFLPFNGGPRICLGQQYALTEASYTVTRIL

QKYARIEVADTDTGPMMDLTLTIAPKKVLLRLWKA*

>CYP5093H2|489950|Tubin1_1

MYGSLVTVWTGRRPTIVIGDPKVACDLLDRRSVIYSSRPRFVVMGELFTNNDSLLTMPHGDKWRKTRKIFHMGLHRRACE

SYKPIQEAESQRLTRDLLVTPEVFGKHLERYASSVMICVAYGRRVDSLEDPIVKKIYDRMAYMSTLNVPGAFWAESFPIL
KLIPDCLAPWKREVKRRAKDSTEMLSRLALDVRDRMKKGDAPASFTKTLWERREGNPEALSEREIAYATGSLFGAGSDTS
SATLMSFFLAMTCFPRVAAEAQEELDRVVGRDRSPTWSDEPNLPYCRAVIKETLRWRPVAVMGGTPHASIKDDQYNGHFI
PKGATILGNLWAIHHNEKYFKDSHDFIPERYLGSGKIDGMEPYPHRDGHSAFGWGRRICPGKQLAENSLFITITRVLWAF
NISKATDKHGQEITPNIFAYTDGFNSKPQPFQCRIRSRTPGIQQIIEREARLGEQFLDKYKCN*

>CYP6498A1|490774|Tubin1_1

MGTLLILAICAGATITLYTVSSIIHLLYNIREVRRSGLPYLILPWHEMNLFHVLTCGFNWELWQYLPNWTYLKIFWQDWC
HHTKFELFEKYGDVICAVSPGGVTIYVGSVEVARQIYERRNDFPKATKVYEYVRFYGDNVLTLEGAEWRRHNKYTRPPFN
EAVNKVVWDEGIKQAYAAVNVWNRKNSCEKVGRDLRIISMNVLSLSNFGVALPFDHESETAEHFGRNIPPGHKMSYGKAV
NHVLDNIIPLVISPKWLLRNGPESLKKIGQSYDELGIYLKELTQIDNKAAASDRKNLLGSLAKASAGDGPDKGSGLADAE
VIGNAFIFAVAGLETTTGTLHYAIMHLALNPDVQDWLYEDLQEALKDEDQDPSKWDYEKVYPKMAAVLCVIHETLRVNTP
HMHIPKWTADKYQPVDWRGKQCVMPPGALAYITTTALHYNPSLWGDTVKGFHPQRWDLRFPSEGWVRTDPETGVKTPITP
SETPRTGVQSAPYCYLRTPVKGAFAPFSDGWRACVGKNFALVEMTAVLAVLFRDSSVRIKRREGETQEMADNRGKSAISN
SKRYLTVMIRHDVVLEWVGR*

>CYP663L5|495232|Tubin1_1

MEACYSGSRGACYSGSWTMPPSRPWRFCSLVNFAFYPFDKMCIAGAEDPGGRTPPPITIRPLALFTSFLAVMIAAYLFKR
KRRQPPLPPGPRGVPIFGNLFQIAPKYQWRQQQEWSQKYGPIFKLQLGVHMVIVLGTYQAARDLLDKRWRIYSDRPGFVV
SAKYISGGFRTLLMRGEMWNAHRRLQATVLSPRMSQKYKPVQDLESKHLIHALLKKPDDFARQFHRYSASLVHSLGYGKR
LVTGLEEEVQTVDTIMRNFTEAGRVGKWWVDMFPILELVPKYFAEWKRISAKIFEYESNFHIKNLNEALERKGWNWAKMY
KNSSFSQGMSDLEVGYDLGILNEAASDTTTIALEVFMLAMLKFPHVARRAQEELDRVVGESRSPTWQDKDSLPYIEKVIQ
ETLRWRPVAINAFYHAVTEDDEYLGYRIPKGSWVVANSWGIHMDPELYPNPNDFNPDRYDDEKLGHVAFGYGLRACTGKH
IAKNSLYINISRILWAFDIGPKMRADGTEVLVDEMAFTTGFLSRPLPFECSIRPRDQGRVGVIETEWAEADKALLSIMAE
EKFT*

>CYP6592A2|516023|Tubin1_1

MNAANTPKLRPEYTTLSSTDSFLLSLVLLPILITIVYNRYYHPLSPFPGPFLASIWSGWRDWENYRNMEDQEPLLHERYG
PIIRVSPNMISVADPDCLKEIYTRTDFLERRRSFPHNGAVDAPLGSSVLLHTELGKGIMPGYSVGTAGSWESIVDERVTE
RVGKLKAVYGGSGVRFGFSEWARYLSVDLVGWVVFGVDMGCTQNGRDNGDHIPGSKSGLGVAGGLARFPNIYRTLASDSF
IRNILMAKADKEGLGVVREKIGLIIDAAWKSREEAEARGEEWHLNGKAMSAKNSGGTRLTKEEVHSECFLPFIAGQYSTA
LAIANPLKLLLANSRVLATLLSELTTHYAQKPLSHILPWTDIVDLDAKLPYLSAVLRESLRLHPPFPVGNPGMIPSSGIT
LTHRNRLYTIPGGYEISVSPIVIGRNKQIFGEDAHCFRPERWLEGNEKEIKRMKDVGFEWGTGSSRCIGKALAQMAVAKA
IVMVLRHFEVELAVVIREERCGG*

>CYP6001C26|567762|Tubin1_1

MASSGDGTNGAANGHSKYATQSKPPPPVAKPTRAAVDSTFEQFANLLHASDRPLPNRFGDGKDQRPPEVKPTGVLTDINT
LRRGGFFWESVGTLWDFLKQKVKGGAVDDKTMIMERVIQLTSRLPPTSKARVALTTLQVDQLWDSLQHPPLSYLGDEFSY
RQADGGYNNIMCPQLGRAGSAYARSVKPMTKMPGAPPDAYTLFDRIFSRGPNDEHFREHDNNVSSMLFYTATIIIHDLFR
TNRENQNISDTSSYLDLAPLYGNSVEDQSKIRTFQDGKIKPDSYSEKRLLGFPPGVSVLLIMFGRFHNYVVENLKAINEG
GRFSLKFPRYPNGDDEATRQAKALRQQDEDLFQTGRLVTCGLYINFILNDYLRTIVNLNRVDTTWTLDPRFEASKVYNPD
GTPAGIGNMVSAEFNLIYRWHSCISKRDDQWTKDFYKGLFPGRDISDIEMPEFLRGVGKWERSLSDDPIQRNIAGLERKA
DGSYHDDDLVKILTESVEDVAGAFGARNVPHVLRFVDVLGIEQSRKWMVGSLNELREFFGLKPHATFEDINPDPAVADTL
RQLYDHPDFVEMYAGLVAEADKKPMVPGVGIGPTYTISRAILSDAVTLVRGDRFYTADYTASHLTNWGLQEASSDPATVY
GCVGYKLILKAFPNHFKFNSIYAHYPLTIPQENHKIHTALNSVDQFDFERPVYTPPRIPISSYNATKQILCDAENFKVTW
GAGFDYIMRADFMLSGDKPSNSEQKQFVRDRLYLGGVDWKQQIRQFYEEVTEKLIRKKAYSLGDTYQVDAVRDIGNIAQT

IFAASIFNLPMKSEDHPKGIYTEQELYTILCVMFIVIFFDIDSSKSFPLRQAGFKVVRQYGTLVEAQVKAIKNWSWLQGV
WDPLNIRGRNKSSPLKSYGWTMIKRLLDTGKSPYDVTWSYIVPTAGASAPNQGQIFAQVLDFYLEDRNAHHLAEIQRLAQ
SGAADAWETIKKYALEGGRLAGTFGLYRRVEPDNITIEDNGRNIELRKGDMVFVNFITASRDPVVFPDPLEIKLDRPEAS
YMQYGDGPHECLGKAANIIGLTTMLMQFGKLNGLRRAPGPQGALKYIPKPGGFKVYMKEDWSAYWPFPTSMKVRFDDII*
>CYP6001E1|495006|Tubin1_1
MSFLGLFGGGSSANPNSEYGDESAATKEITYTGIAEDIKAAGGKIPEDLKLLLETGAQKVSKGPVDDKQLVMERLIGLVA
SLPQNSANRKKLTSTIIDTLWDSLQHPPLSYVGDKYQYRQADGSYNNILYPDLGKAGTEYARTIRQDKKLYGAKPDAGLL
FDLLMARGDNFKQNQAGISSVLFYHASIIIHDVFHTNRKDFSKSDASSYLDLAPLYGSNQEEQNLIRTMQDGLIKPDTFS
DKRLLGLPPGICVLLVMYSRFHNYAAKTIKAINESGRFSLPASHATASPEDQAKNLAKLDNDLFQTARLITNGLYVNISL
HDYIRGIANLNHSESTWTLDPRVEIDKSFDGEGTPRGVGNQVSCEFNLLYRFHSAVSKRDDAWTKDFFGKLFPGQDPASI
GMSQLLQGLIVFEKSISEDPAKRTFGGLKRTGADGSGAFNDDELVKILKESIEDPAGAFGANTVPEILKPVEVLGILQAR
KWQVASLNEFRAFFNLKKHKTFEDINPDPYVANTLRKLYDHPDMVEMYPGMFLEDTKPRMDPGMGLCAPYTVTRAVFSDA
VTLVRSDRHLTLDYTPANLTNWGITEVAQDYDTLGGAKMFHLILNAFPSYFKYNSVYAMQPFYTPTESRKIFDKFGKSYL
YSFDPPAKTASPIPILTHAGLKRVLNDQKNFKVPWGEAMEALNNEHDFMLAGDLSSHTQQRNLVGDAIYDVTGSRKQFKD
YTEEITLKLLKREVYQLGKVPFNQVDIVRDIGNLAALHFAASLLYLPLKSDENPNGQYSEQELYKTLTDLTWFVFSDSDP
TKSWEHRREAKKSIDKLGEVMVEEVKKFKTPIGIWDKLTGGTGVRPPPPSLKDYGSNLVKRLLGSGKSAEDVAWILLWSG
CAFVANSACAFAQLIDFYLQDDNRKHWAEIQRLSSLNTPEADKLLTKYTLEGTRLSNSLGIFRTVDPVDTQTITIKQLGQ
DVVLKKGDKVFVSFVYASKDASVFPDPLDIKLDRPAELYVTHGEGQHQCLGKDINIIQNTYMLKTLAKLRNFRRAPGDEG
KLKFIPKPGGIKLYMNADWSKFTPYPTTMRVQFDGPIVA*

Tuber magnatum v1.0

>CYP6498A1|117759|Tubma1
KFELFEKYGDVICAVSPGGVTIYVGSVEVARQMYERRNDFPKATKVYEYVRFYGDNVLTLEGAEWRRHNKYTRPPFCEAL
HKVVWDEGVKQARAAVNVWSRENSGVRVGRDLRVISMNVLSLSNFGVALPFDCGSETTEHFGQKNIPPGHKMSYGNAVNN
VLDNIIPLAIVPKWLLRNGPESLRKVGLSYDELGIYLKELTQNGERATTPEKENLLGSLVKASAGDGPEKGPGLDDAEVI
GNAFIFAVAGLETTTGTLHYAIMHLALNPDAQDWLHKDLQEALKDEDQDPSRWEYERVYPKMASVLCVLHETLRLNAPHT
HIPKWTADKCQPVNWRGRECLLPPGALAYITTTALHYNPSLWGDTVKGFNPQRWDLRSSSEGTPVKGAFAPFSDGWRACV
GKNFALVEMAAVLAALFRDCSVRIKRQEGETQGMADNRGKSAISSSESYLTVMIRHDVELEWVRR
>CYP548BE2|164149|Tubma1
MVAFLNLIKLLSTSLAVYISSLVLYRLFFHPLARIPGPFLAKITDWYTVYHAYRGDRHLALYKAHEKYGPVIRFAPNLIS
FNSASSLKAIYGHTPLSRSLQKSQFYAAFPAVKGVHNTHNAISKAEHGRKRRVLSAAFSDAALKSVEDLVLNNIDVFSSK
VNDAFKQGVGIDIGDLFSWLTFDVMGDLCFGKSFGMLTEETTRFVTNLISQAAHNHYINGNYLPLTTLKLSKVLFPTISR
DRWRFIEHSRACANERVSHGKGYKKDFFHYLLDAKDPETGEGFETKELWGEANVLMIAGSDTTATAMSAAMFYLCRNPQT
LETLKNEIRGTFTNKEQIVGGKGLGDCHYLKACIDEAMRLAPPVPGLLPREVIAKEGIEIDGVYIPEGIVAGTPIYALHH
NPAYFPDPFSFKPERWLSAFTSNEEIEAARSAFTPFSIGPRGCIGKSVAYMELRLTLARLLFEYDCEEVETEGKAALWKE
GYAMVDGEYRLMDHFTSRKEGPVMKFSRR*
>CYP51070A9|176862|Tubma1
YGKYGKAFRIATPGRDIILFSNAEVLQELKTLPNSIAAFRPAGDEMIQSSYTFHEGILQDAYHVDLMRKNMTEKLSNLLP
DIVEEITEAFEGLTNIKNEWTNLNIFNISIVKCILISLVGIPICQNLDYLDSVNQFSLNIMKSALIIDTVPWFLRGIVNS
FLKKSDAENIIIMHIGREFEIRRKAKKSQSTSNIIASDAIQWILDAVPPETPILKLVQKLMFFNFASVHTTSIALTHILY

DLAAHPEFQDPVCIEIEEVLRAEGGWTKQAITKMKKLDSILRESLRLNGAAIVTVVRKIITPYTFIDGTFAPKDTWVGAA
STTIHHSPELYKDPEIFDGFRFYEKRQLKENSFHFQTTSLALDYLPFGIGKNSCPGRFFATIELKVAVAYILCNYRLRLN
GGASGKRPQNIYEGFVCMPDPTTGIELKEREDRQKSVLFPSHMASHK*

>CYP6220A4|180393|Tubma1
MTDSDYSLNGISDTATDYLTAAICALGGLIVWKVVSAIFFSPLSHIPGPLATAITPHYINFLSALNRRTVGTYLLHKRYG
PIVRVSPTEVSVLSPQAIKEVYSSPNYTKYAPLYSAFTHFGAPSTFTSCTREEHGWRRKAVSQSYSLSFVLKDEATTGKV
LKAVKDYLNFVESDRRVDIYNASTFYATDVVTGKLFGLGASMKTLAGNEAHREIVLRHYARTGRAPIWMYLEFPLIMKVL
DWLSFYAGRAWSWVCGEEVKDREVVSRIQKWGWDAYMDAKSNSREGTVAGRLARFVREGDSTEGAWDDRGAVSELMAQML
AGMNTTSDTLSFLMYQLSLPESRGVQKRLHSELLDAFPKSAVISSSDWPHSMGMGGLPHETILKTLNLPYLDAVLKETLR
VYTAIPITLPRVVPPSTATDSISGRRNGRIVEGKFIPAGTTIGTFAYGIHRDEVFAVETGKKDKRGDVDSFLPERWLING
GVGGELTPDELAQEKQRIRTMEARLWAFGSGGRNCVGRHLSILEMKLLLATIYSRYQTQATPNSGVKITHNRWDERRTFR
DVLPFRGVDGVVTFTPYE*

>CYP5093H2|276378|Tubma1
MPPGPSPVPFVGNKWDLPLQKPWYKFKQWTDMYGSLVTVWTGRRPTIVIGDPKVACDLLDRRSVIYSSRPRFVVMGELFT
NNDSLLTMPHGDKWRKTRRIFHMGLHRRACESYKPIQEAESQRLTRDLLVAPEIFGKHLERYASSVMICVAYGRRVDSLE
DPIVKKIHDRMAYMSTLNVPGAFWAESFPILKLIPDFLAPWKREVKRHAKDSTEMLSRLALDVRDRTKKGDAPASFTKTL
WEKREGKPEALSEREIAYATGSLFGAGSDTSSATLMSFFLAMTCFPRVAAEAQEELDRVVGRDRSPTWSDEPNLPYCRAV
IKETLRWRPVAVMGGTPHASIKDDRYNGHFIPKGTTILGNLWAIHHHEKYFKDSHDFIPERYLGSGRIDGMEPYPHRDGH
SAFGWGRRICPGKQLAENTLFITITRVLWAFNISKATDKHGQEITPNIFAYTDGFNSKPQPFQCRIQPRTPGIQQVIECE
AKLGEQFLEKYKCN*

>CYP5959A2|276760|Tubma1
MGILIQFIDYAEDKNLYGYSLLCTTIALLFLGLFLLWVKEWSIHSPTKLPIVGIESQGYFALLRARRKFVSDGFRIVRDA
YYKYRGKSFVITTNSYYKVVLTHNQVKELSNAPDDTVSFVHASAETMMAEYTGLSQFSVDKYIPDVLRAKLTQNLGSMKE
ALLDETRFALSSEIPGSATDDEWVPVEIFTAVHRIIARINGRIFIGLPLCRNEDWLTMSITFTGVVFRAIEKLAGVPKLI
RPLYSYTRNLVKDINWHKRKARSLLDPIIHKRLEEESLAEENGAVYRKHNDVLQWFTDHVMPEHRTVEALTEMQLHLSMA
SVHTTTLYFLNAILDLAEHQECIQPIREEMEAVISANGGALDRTALRKMRKTDSFFREAMRGKFALFTFNRKVMKNLTLS
DGTYFPKGTLLAAPSAMLSTDPEFVEDPETFDGFRWYKRSLEVKDSGAEKNNWGTTASYDLTFGHGKHACPGRYYATEEM
KLMLTFILLQYDIKYPEGQSRPVNFDRGEYSYPNRAQKLLFRKLPGKKKFPFL*

>CYP6592A1|278413|Tubma1
MNATSTPILRREHTTLSSIDSSLLALILLPILVMIVYNRYYHPLSPFPGPFLASIWPGWQAWENPGNVEDQEPLLHERYG
TIIRVSPNMISVADPECLKEICSRADRTQWWLPFGHKGIADGSSIRSSGLHEELRKRAGIAYSADVTRWWENVVDWRARE
WIRELKGECGGGGIRLNFSEWARYLSVDLVSLVVFGVDIGCKGNERDNGGHIQAFERGLGMAGVLARLPNIYGALACNPL
INNSWMAKVEKEGLGAVRVKVGLIIDAAWKSREEGTTKGVTLLEEAMSAKNSDGTRLTKEGVYSECFLPFLTAQDSTALA
ITSTLKLLLTNPRVLATLLSELATYYVGKPFSHIPPWTDIADLDTKLPYLSVVLRESLRLHPAFNMGILRIVSGVTLTHR
ERLYTIPGGCKIRANPFVIGRNKRVFGEDAHCFRPERWLDGSKEGVERMKAVRLEWGTGSSDCLGKALAQLAVAKAIVMV
LRNFEIELASGHARGEMWKLVATGAHHAAELWVKVEPKEDKTDGKEVAGEMDEASEHS*

>CYP530G1|284506|Tubma1
MAIPVFWATVPGLTLGVCVASFIATIIYFAKGTSRGKNFPPGPPTLPILGNMHLVPQERPYLKFTEWAKKYGGIYSIKIA
KQTIVLISDVKILKELYDKRGAIYSSRPLPHIGAEIVCPDQTHIIYMPYGETSRNYRAQYHQFMGPGKVEQILPWQSAES
TLLLKKIATSPDRYYEHTMRMATAIILESVFGVLPKDYDDPEVTELWTVQREFSEILALTGPPVDHFPFLKWLPDIVSPW
RIHARNVRAMHRKLYFRLLNLNKARMERGERYGTFVEKLIDDKPKHGLSDERIAYVCATLMEAGSDTTASQALDFMMALL
AFPDVLKKAQEEVDRICGTSRLPTLDDRDQMPYIEACVNEALRWRPPLPYGAPHLLMKDDWYEGYFMPKGTVLFQVQWAM

NMDENVYENPQDFMPERYIRNRFGTKFDAEKDAETGRKEQYGFGLGRKICPGQWFARNTLFVLFAKLVWAFDMKVPADPK

TGKPIPLETDVRTAFMDGLTTTPFKFPIEFKIRSKAHEEAMNTDLVASDKIFAKYGGATV*

>CYP5959A8|292313|Tubma1

MGIFMQLINCAKDKNFYGSSLSCVTIAFLFIGLPLLLNKEWSVHNPTKLPIAGIEYPGYFSLVDARRKFVSNGFHILRDA

YYKYHGKNFVIATKTYCKVVLMHDQVRERSNAPEDTISFSRGAAEQSRDPIRMLFFSGSKKFCKSLYSEILMKVQSIGSM

KEAILAETQFTLNTELPGFSTDEWVSVAIFPVALRIIARVSARAFISLPLCRNEDWLTITITFARDVSKAVDELAGVPKM

IRPLYAYLWNPAKEVGSHKRKAESFLAPIIRERLEEESLAEKNATDYQKPTDLLQWLLDRLERHHKTPEALSLMLLQIGL

ASIHSTALSVTNTMFDLSQHQECAQQIREEMEATISANGGVLDRTVLRKMRKTGSFFKEVIRRGSLSIARSCMKNVTLSN

GTYLPKGTIIGAPSAMFATDPEYIEGPETFHGFRWYEKSLGVKHGEVNANSCGTTPPNDLSFGHGRNALPGRFFAIEEMK

TILTFILLQYDIKFPEGQSRPDNIYTGENSYPDLSRKLLFKKLLGPKKFPFL*

>CYP5959A4|292316|Tubma1

MGLFMQFIDSAKEQNLYGYPLFCTTLALLSFGLFLLWMKEWSIHGPTQLPIVGVESPGYFALIKARRKFASSGFRLVRDA

YYKYHGKNFVAPTSRNERVVLTSRQVKELSNAPDGVVSFYHAAGATLMAKYTGLDRFLPVGYIRDALRIKLSQDLGSMRE

VILEETRFAIDCEFPRCTADEWTSVSVFAVILRLVARPSARAFVGLPLCRDEDWLTISIAFSAEVLKAADNVARVPKFLR

SFWSYLFNPGKVLDSHKRKAESLLAPIIQKRLEEESLAEKNGTVYERPNDVLQVLLDRVTPHHRTPDCLAEIQMLICLAS

IHSVSLVFVNTIFDLAEHQECIQPIREEMESLISANGGVLDRMVLRKMRKTDSFCKEVSRTTVGLFNFNCMARKGLTLSD

GTYLPKGTMLAAPAAIFATDPDIIEHPETFDGFRWYKRSLQVREGEVNSNGWATTSPHDLVFGHGKHACPGRFFATEELK

FMLTFILLQYDFKYPEGQSRPDSVYKGEFRYPNPAQKILFKKLPGPKKFSFL*

>CYP6220A2|293286|Tubma1

MIDSKYLLDRISGTEYLTAAICAFGALVVWKVVSAIFFSPLSHIPGPLVTAMTPHYINLLSALDRRTVGTYSLHKRYGPI

VRVSPTEVSVLSPQAIKEVYSSPHYTKYTPLYSIFTHFGAQNAFTSCTREEHGWRRKVVSETYSLSFVLKDEAATGKVLK

AVKDYLNFVESDRRVDIYNANTFYATDVVTGKIFGLGASMKTLAGNEAHREIVLGHYARTRRTQVWMYIEFPLVMNVFEW

FAFYTGRVRSWLRGEEIEGWEMVSQIQKWGWDAYMDAKSNSREGTVAGRLAKLVQEGSSAEEDWDDRGAVSEVMDQMLAG

MDTTGDTLSFLMYQLSLPESTNVQKRLHSELLDAFPKPAEISGSDWPHSMDIQSLPHETILKALNLPYLDAILKETLRVY

TAIPITLPRVVPPSTTADKISGHRNGRIIEGKFIPAGTTIGTLAYGIHRDEVFAIETGGKDGPGDVDSFLPERWLINGGI

EGELTPDELAQEKQRVRTLEARLWAFGSGARNCVGRHLSILEMKLLLATIYSRYQTQVTPNSGVKIAHNRWNERRTFRDV

LPFRGVDGVVTFTPYEC*

>CYP6136A1|295303|Tubma1

MFLNLLGQAVYSLIQHKPYSLAAILILTLGYWTRAYLKSPFYAQKIPGPFLGKFTNAYRWYYVMRHTWHRDLMDLHKKYG

PIVWIAPNDISISDPNLRNVIYGFQNHKKDFTFFRKSHSYETGSINQDFSFIFEQDPERARLGKYHMSHFYSEQGLLNLE

ENFDKAVDELIEGLDKHHAKTGTPCKMVDWAEFFALDLVAQITADQSAGFCLAGKDVNDTAYGMRVIIKTVGALMPLPWV

LTATSRAIRQTLLLKFLINLYRNVLLLPTFTFDTGTADLKSLKEKNPKHLLAKFYNAQSKMREYYPHGNQAEGTTVQLFN

LIAGAAGVVPHTQVKLIHELTRHPEVLQKVREELATTGNTFHLEDFLRHNNCQNKYPIFESAVREAVRLSPAVAFALSRV

VPPSGCQLHQYYIPPGYNVGMAAYQVNYDEGYFGSDVAQFRPGRWLEDHPTQMLDGEKRSMKNYIEAGWLSFGSGGRVCI

GRHLAMFMMMKFTAAVVREFDIRVVKQPTEYHTLVTEMLGMEVLLNRKCAV*

>CYP50043A3|295436| Tubma1

MGILNIGLVSSAVYLLGAWIAYLVLLVFYRLYLHPLRRFPGPKTAAATGWYAGYWDMHMGGQMVKHLVDLHKEYGPIVRC

EPNHLHFSSPEVYSTIYNPTSKLTKDPNLYHSFGAPESVFTLLDPAIARTRREVIAPMFSRRMVLSLQPLINDKIRKLCD

KLSSYADRDKAVDIVSGFRSAAMDIITQYCYNECLDSLDVEGFKHDILVSSRATSESFWVMKYFPLAEWMLTLPTNVTLR

LMPELKGFLAIKDTIEGQVKRYTKTPSLLEKSPYPTVYHRFLDPEVKGGIPSASSLVDEAQNLFFAGSDTVGSALAFGTY

HILATPGLQEKLFAELCQIWPVLEEEPTYEHLEKSAYLTAVIKESLRLSHGTVTPLTRVVPASGMTIQDQPIPGGTVVSM

DTPTIHLNPTIFPSPDTFLPTRWLGSNAKDLDKYLVAFSKGPRACIGSNLAWAELYIALATVFRRFEMTVWETSKEDMEW

MDCFTPITKGDLKVKLKVRGE*

>CYP663N1|297060|Tubma1

MSLIQVISSLPGGSAGTLVLLVLSVGILAQQLFKKKDPLPLPPGPPGLPIVGNSFQIPLINPWRKQAEWTKQYGPIYRLK

LGKDIVIVLGTQKAARDLLEKRSKIYSSRPRTVMAGENVSRELRVALLQRGDKWRTHRRLQGQVINQNISNKYKGFQNLE

SAQLIKELVDRPEGFLSSFHRYNSSVIFAMAYGKRMPRGDEEDVVAIDEIAKNFLHSARLGTWIVDSFPFLNYLPTFMAP

WKRIGNNFYNWAEKMHTFNRNEALQRKGWNWTKEIARMKEAQNVSPLELAFMVGFLYEAGSDSTTIALEIFILALLKHPE

VLKRAQEELDRVVGPNRLPTFDDKDNLPYVRNCVDEVLRWRSPSAGGVPHVVEEDDEYMGYRIPKGAIVVGNLWSIHLDP

EIYPNPTKFMPERWDDEENVHYGFGFGRRACPGKYIAINSLFVNCARILWGFNVEHAENPDGTVIPVDEWDMTQGLMSRP

VRYKASITPRDAKRVEVINQAWEGAEVQLQDMGKVLRMEDF*

>CYP5945C1|298817|Tubma1

MISILEKLNGKETNSVNIYDNMAFDIMADLGFGQKPEESMQSGAGNPSYMNFLHGWMRASTVLTSLRNLCEIGAYIPQDA

ESKEFSKMGERMLGARQKMGKSHPDIFTHLLSGNQESGVKFTQSQLLVNAQMLMVAGSDTTSVTLTCLFRLLSMHPEKQQ

KLYKEIMEAFPNGETPTCATTAALPLLNGAVQESLRMWPAVPSGPQATTPPSGYTIAGTFVPGNVEVRIPQMTMMSDARY

FPRPDEFLPERWTPEMPELVKDKRAFIAFGYGAHSCIGRPLAMNEMRTATARVIQRFEVQLGESYDDRVFRDEWRDYFSV

KLGPCPLKFVPRKM*

>CYP51F1|344734|Tubma1

MGVLSTLLVPMGPCYEELMKLGGPALCAIGFVAFVVLSVVINVLQQLLFKDPTKPPVVFHYFPFFGSTVVYGMDPYKFFS

DCQEKYGEVFTFVMLGRKMTAALGPKGNDFVFNGKLSEVSAEDAYAHLTVPVFGEGVVYDVPNHVLMEQKKFMKFGLTTE

NFRSYVPLIVGQVEDYIKKSKFFKGAKGSVPLSEIIPELTIFTAARTLQGKEIRDALDGSFAKLYHHLDSGFTPMNFLFP

WFPFPQNRRRDHAQRTMAQFYMEKINKRRANEKDDDGQERSDMMWNLMNRSYKDGRKVPDKEVAHMMIALLMAGQHTSMA

TITWMLLHVAAQPKLVARILEEQKRVFGDELAPLSYEKLVLECTFLGHIIRETLRMHPPLHSILRKVKSPMHVNETNWVI

PKGHYLLAAPGVSSMDQKYFKEPNSFDPNRWEGRKAEEETEKFDFGFGLISKGAASPYLPFGAGRHRCIGEQFANVQLMT

IMATFVRKFEMQRPGGGNDVPAPDYSSMIALPTPPSIIEWVKRDP*

>CYP6271A5|353597|Tubma1

MSLDNVISYVREQASDPSRATIAATVFGVWLLSRIVLVFYRLYLDPLSKFPGPKLAAATSLYEMYYDVVQDGTFTWKTDE

LHRKYGPIVRISPHALRLRKSSAYHEIHRMGTPFAKDIRFYRLFNVPRSTFATIDIDLHRQKRSLLNPMFSKRGILDTEF

FIKEKIELLCRRMKEYEAEDKIFNCHEGFAAMAVDIVTEFAYGKSYDVLNSPGFSCRAFAAFDAQHELLLVLKHFPLLGK

VMQALPYWVHLLLMPDGAGMAELEEEAKVNLRALSARMGTTMKKNSAHRTVFEELLAGADPEHTDPIELANEAMTIIAAG

MHTVRWSICVGMLAVAADPLIRTKLYEELKTANPDINAEFSYLQCEKLPYLRGVILEALRLSYGIMGPLPRRVPKEGAVI

GGYHLPGNSTIEMDNYSLHHDEEIFPDSHHFWPERWLTPESKQKEKFVNSFGAGPRQCLGINLAMCELYLTFATVFRRFE

LDVSARRTKRMKVKEHWLTILRDEQLKCKIISRKE*

>CYP567Y2|355416|Tubma1

MSLAHVSYLYTLAAHIRGVVGFSAILGAVFILGRAFYNVFLHPLSKIPGPKLCAATDFVVAYHFLSGRYPFYIKSLHDEY

GTVVRIGPRQLSFSSASSWRDIYGHSYSRKQFPKSHFYSFGTRHLINERDPQKHAEMKRKLSHGFSAKALLEQEDIVQGY

VDKLIGQINVYGTRPEGDEMVKWYNFFTFDLIGDLAFGESFGSLNDAKPHFWVSLLLGNVRAIAWRSVSRWFPVFEKLGV

WTVPRSAMEMRIKHSEYSRRMIIQRTTRRDLLSGQFGPDGPGMTIPELSGQASTIITAGSETTATFLSGTTYHLLKNPRV

YNLLVEEIRSAYNKYDEITDNNASKLKYLSAVIDEGLRIYPPVGAGTYRESPGETVDGMYIPKGVELSTSPWSTCHSPEN

FHDPEEFKPERWLDPDCPDKKHASQPFSLGSRVCIGRNLALKEIRLVLSKMFWVYDMELVNKEVDLNRDSTSFVLWAKPD

LWVRFMRRKGVHVPVLDSE*

>CYP6001C26|356988|Tubma1

MASNTNGTNDAAQGRSNGCSNGASNGVSKAKTSKSAARAKPPPPVAKPTRAAVDNTFEQFANLLHASNRPLPNRSGDGKD

QRPPEVKQTGVLTDINTLRRAGFFWESVGTLWDVLKGKVKGGLVDDKTMIMERVIQLTSRLPPRSKVRVALTTTQVGQLW

DSLQHPPLSYLGDEFNYRQADGGYNNIMYPQLGRAGSAYGRSVKPMTKMPGAPPDAYTLFDRIFSRGPNDEHFREHDNNV
SSMLFYTASIIIHDLFQTNRANPNISDTSSYLDLAPLYGNSVEDQSKIRTFQDGRIKPDSYCEKRLLAFPPGASVLLIMF
GRFHNYVVENLKVINEGGRFDLKFPRYTNGDDEATRQEKALRQQDEDLFQTGRLVTCGLYINFILNDYLRTIVNLNRVDT
TWTLDPRFEASKAYNPDGTPAGVGNMVSAEFNLVYRWHSCISKRDDQWTKDFYEGLFPGRDTRDIEMPEFLRGVGRWEAS
LSDDPLQRNVPHVLRLVEVLGIEQSRKWGVASLNEFREFFGLKPHATFEDINPDPAVTNTLRQLYDHPDFVEMYAGLVAE
ADKKPMVPGVGIGPTYTISRAILSDAVTLVRGDRFYTTYYTAAHLSNWGLQEASSDPAIVHGCVGYKLIIKAFPNHFKFN
SIYAHYPLTIPEENHKIHTALNTVDQFDFERPVYTPLRIPISSYRATKQILCDAENFKVTWGAGFDFIMRADFMLSGDKP
SNSGQKQFVSERLYLGGVDWKQQIRQFYEEVTKKLIRKKAYSLGDTYQVDAVRDIGNIAQTIFAASIFNLPMKSEDHPKG
IYTEQELYMILCVMFIVVFFDVDPSKSFPLRQAGFKVVRQYGTLVEAQVKAIKNWSWLQGVWDPLNVRGRNKSSPLKSYG
WTMIKRLLESGKSPYDVTWSYIVPTAGASAPNQGQIFAQVLDFYLEDRNAHHLAEIQRLAQLGTEEAWETIKKYALEGGR
LAGTFGLYRRVEPDSITIEDNGRDIELRKGDMVFVSFITASRDPVVFPDPLQVKLDRPEASYMQYGDGSHECLGKEANII
GLTTMLMQFGKLEGLRRAPGPQGTLKYIGKPGGFKVYMKEDWSAYWPFPTTMKVRFDDII*
>CYP61A1|359533|Tubma1
MDEIANSSTFAPPSAVSSAEQPFQTLVEGFETIGRTLEGLSGWQIFFSVVILSITYDQCRYIWNKGSIVGPSLKIPFMGP
FLESVNPKFSQYLAKWNSGPLSCVSVFHKFVVIASTRDLARKVFNSPGYVSPCVVDVAKKILRPDNWVFLDGKAHVEYRK
GLNGLFSRQAMGMYLPGQEEIYDAYFKRWLELSKEGKPIPFMSEFRDINCAVSLRTFVGHYISDEAVKEISENYYKITAA
LELVNFPIILPWTKTWYGKRCADFVLNEFAACAAKSRIAMKEGKEPGCTMDSWIKSMIEAKESDTAKIAAARGLPQIREF
SDVEISMTIFTFLFASQDASSSATTWQFQIMADRPDIMKKIREEQLRVRDGDPHKRLDLDMIDKMVYTRAVVKEQLRYRP
PVLMVPYEVKKSFQITPEYKVPKGAMVVPTLYPALHDPEVYNDPDNFVPERWLEGGEAEAAKKNWLVFGAGPHVCLGQNY
AIMNFMAMIGKASLKMDWEHHATPKSEDIKVFATIFPEDDCFLVFKERDPHXGVALLSIVLPGLFSSYR*
>CYP5959A1|361990| Tubma1
MAISIQLLDYAEEKKLYGYSLFSTTLALLFLGLFLLLKKDWSTHSATKLPIVGIESPGYFGIANARSNFVANGFHILRNA
YQKYPGKNFVVTTYGHDKVVLTHEQVKELSNAPDDTISSSHAAAETLMGEYTGLDRFVDTKYVEDVVRVKLTQNLVIGVK
INKKNPDPEHTPSPDSKLFHQYSDSELLTKIQSTATLKEAILEETRFALNTELPECTTGEWTPVDIFTLIIRMVARMSGR
AFVGLPLCRNEDWLKITIQFTTDVFMTQSKLTSVPKLFRPLYVFMRGSAKDLAGDRKKAESLLAPIIRKRLEEERLAEKN
GTVIRKHNDMLEWLSDRVHPQDKNLEVLSELQLILSLASIHSTSLSFFNAILDLAEHQEYIQPIREEIEAIISANNGVLD
RAALRKMRKTDSFFKESMRGKIGLSMFLTFREVSFNRKVLKDYTLSDGTYLPKGTLIAAPTLMFSTDPDFIEDPGVFDGF
RWYKKSLETERGAGRRHYWATTASNDLTFGHGKHACPGRFFATEEMKIILTFILLQYDIKYPEGQSRPANINRGEFSTPD
ATQKLLLKKLPGPKKFSFL*
>CYP6135A18|362276|Tubma1
MLAHLFLTLLLLFLGYACYSLHTLIRKVRPLGIPYFVIPFYPSPLPRVFLLPFVKLLINVFNLTHIWFFLLTLDWQVHQR
YDVYRLVGSDVFFTITPWEVILHVADPDMAVEVLAGKGGNGELYPKTEVVGRMMGLFGKNVLTTEGAVWRGHRKVTAPVI
GKSLSIVWEEALAQVEKMLQHYDKHGDISASHRDFRRATLGIIAHAGFSKLMDWVPLTATTTREKDSEGSYQECLDILFE
NFKWTVIAPPWFLEMLPFKASRKAGAAASGFRFYLNEWFEEKKAKIERDDKTVFESSSRADLMESLVRSSGLDEGSALKT
PLLSKSEMFILAGHESTGHTIANAVYFLAMYPEYQVKLQNQIDSILGDSNHNETSYDKHFDAFSSGWMAAIMHETLRLIP
AVVVVTRQIGPAPRTFPRSGGQSPVTLPPGVEFWIELIGLSHNPKYWTQPGKTEAESEISEFRPERWLPKPGDNSMFKPY
NGSFIPFSIGTRGCIGKKFAHVEFAAVMLGLFREMSVEFDSCGGTKTFDEARLECLAQMEKFATVITLRPSGKMPGIKWV
RRRREVAG*
>CYP6188A5|363833|Tubma1
MAKIFQSTEAALRDLLPGARAQQKMGPGNTTAKYFIALLDTHPTESQWDYPLSFIPGILGVIFVYIFVKLGLHLYRTRND
TPLKDVPGPWLASCSPLYRFWYAVVKGNFHNDLTNLHRKYGNVVRIAPNEVSVWDPRAASEIYAHGDKGYPKCDMYDIAL
PNGFFNLAVERDIQKHAEGRRAIARDYTMTATLTAESHFDNIIRDFILALDKNFAQKGSVCDFTIWSEYFTYDMITDLVF

GEAYGFCKTGLDFDGSLKDLRQMLNLSPFLSYLPWIWPITQNPWIKKIGMNHYARNVKANIMKRLNNGNFSGHRDLLDGL
IESRLAAPDTASVALRAILINLVCNRDVYNAVMDELTGLKLSNPATWKELADAPLLHATVKETLRLHPPAGFNLPRAVPA
GGRTVCGYHLPEGTTVGMSAWCVHANEDFWGKDALEFKPQRWLDPENAFKFDRYGLSFGQGARACLGKNIAMVQLVKVAA
QILLNFEFELVDENKIREMFLLLVVLDGVKIAFKRRAGGPLDDTIGMEGVAI*
>CYP52AU3|216767|Tubma1
MGIFSVDGVSWSHSRALLRPSFDRANVSDLGQLEGFMQIFFSRINELPRQPLTHTRAVELQELFQCLTMDSSTDFLFGDP
IGALASKKYTLGNGEMAFDKAFDIAQYGLAMRAPLSSFYWLYNGREFRKACSTARAQVAIYVSRALRKLNLAEKKSGEEG
LGKKYVFLEELAERTQDPQVLQDQVLNVMLAGRDTTAALLSWTFLCLARNPEVYKKLRAEISAVVGVDEDARLPTQAELR
SIQYLRWIIQEVLRLYPPVALNARRALHATTLPYGGGPDGNSPISIRKGERIVYSVFSLHRRTDLYGPDANEFRPERWGE
EALRRIGWGWLPFNGGPRICLGQQMALTHASYFVTRMMQVYKEITPKDFALADDSYDTKLVMASGRGVHVFLF*
>CYP52AV4|294719|Tubma1
MELTFKSLLLLAAAFYFLLRITAYLLIAHQNRIFAKSHNCLTPRRFPSSFLGLPNWVRVMRAAKRGDVLEHIASRYPTYG
NTWKGRILFGASIGTIEPENIKAMLATSFKDFGLGPERHANFYPLLGDGIFTLDGAGWEHARANLRPQFSREQVSDVGAL
EVHVQRLMNRLPEGDGEVADLQSLFYCLTLDSATEFLVGESVDSLLSPELNPTGDVDGGKEEMSFAQAFNVSQTYLINRT
RVRGLYWLINTKRFRDANAIVHRLVDKYVDMALHPEKRARKISEKKYVFLDAIAAETKDPKYLRDQTLNILLAGRDTTAS
LLSFTFWLLARHPHIYKKLREEILLAFGTGRNGEGKHPSFSALKDLTYLRYVLNETLRLYPPVPLNGRSALRNTVLPRGG
GEDGLSPIFIPKGQRVDYSCYALHRRKDLYGEDADTFRPERWGEGIGRGWEFLPFNGGPRICLGQQYALTEASYTVTRIL
QKYARIEVAGTHTGPTMDLTLTVAPKKVLLRLWKA*
>CYP6001E1|339907|Tubma1
MSFLGLFGGGSSANPNSEYGDDSGATKEITYTGLVEDIKAAGGKIPEDLKVLLETGVQKVSKGPIDDRQLVMERLIGLVA
SLPQNSANRKKLTSTIIDTLWDSLQHPPLSYVGDKYQYRQADGSHNNIMYPDLGKAGTEYARTVRQDKKLYGAKPDPGLL
FDLLMARGGNFKPNPAGISSVLFYHASIIIHDIFRTDRDNFAKSETSSYLDLAPLYGSNQEEQDQIRTMQDGLIKPDTFS
ERRLLGLPPGICVLLVMYSRFHNYAAKTLKAINENGRFSLPRSHTTDTPEAQAKNLAKQDNDLFQTARLVTNGLYVNISL
HDYIRGIANLNHSESTWTLDPRIEIDKTFDGEGTPRGVGNQVSCEFNLLYRFHSGVSKRDDAWTKDFFGRIFPGQDPTTI
GVPQLLQGLKIFEKSIPADPAKRTFGGLKRTGADGDGAFNDDELVKILKESIEDPAGAFGANTVPEILKPVEVLGIIQAR
KWQVASLNEFRAFFNLKKHKTFEDINPDPYVANTLRKLYDHPDLVEMYPGMFLEDTKPRMDPGMGLCAPYTVTRAVFSDA
VTLVRSDRHLTLDYTPANLTNWGITEVAQDYDTLGGAKMYHLILNAFPSYFKYNSVYAMQPFYTPTEGRKIFDKFGKSHL
YSFDPPAKMAPPIPIVTHAGLKRILNDQKNFKVPWGESMEALNNQHDFMLAGDLSSHTEQRNLVGDAIYDVTGSRKQFKD
YTEEITLKLLKREVYQLGKVPFNQVDIVRDIGNLASLHFAASLLYLPLKSDENPNGQYTEPELYKTLTDLTWFVFSDSDP
TKSWEHRREAKKSIDKLGEVMVEEIKKFKTPIGIWDKLTGGAGIRPPPPSLKDYGSNLVKRLLSTGRSAEDVAWILLWSG
CAFVANSACAFAQLIDFYLEDDNRKHWAEIQRLATLNTPEADKLLTKYTLEGTRLSNSLGILRNVDPADSQTITINQLGQ
NVVLKKGDKVFVSFVYASKDASVFPDPGEIKLDRPTELYITHGEGQHQCLGKDINIIQNTYMLKTLAKLRNFRRAPGDEG
KLKFIPKPGGIKLYMNADWSKFTPYPTTMRVQFDGPIVS*
>CYP539A43|355211|Tubma1
MLFGAITQSVPALLIALAVVGVLTPVFKKVRGNWKIHELGGRAKPVPSRWFLFGLDVVERAVVAARANKYMELWEGWFKI
AGSRTIEISIIGTRAILTDDPENIKAVLASQFHDYGKGEPFRQDWKSFLGDSIFTTDGQLWHASRQLIRPQFVKDRVSDL
DIFERHVSHMLEQIPRDGATVNVSDLFYRFTLDTATDFLLGQSIDSLGTPQVEFARAFADIQKHMSNKSKVGPMGWIFPE
GKYKKDLKVLNSFVEPYVERALEMRPEELKSRNEKSYNFLHALAEFTRDKRLLRDQLVAVLLAARDSAAATLSWTLYEIA
RRPEVVQKLRAEILGRLGPNGKPTYADLKEMKYSQHVINEALRLYPAVPFNMRMSLKDTYLPRGAGPDGLGPVGMPKHTI
FAYSALTMQRRVDLFGPDVEKFDPDRWENWTPKSWQYIPFNGGPRTCIGQQFALTEMSYVLVRMFQRFESVESRQTEEQY
QNCDIVVSPGAGVPVSFKLAGVP*

Tuber melanosporum Mel28 v1.2

>CYP663L2|369|Tubme1v2

MEACYSGLTEACYFGSRPVPPCRPWGFCSLVNFAFYPFDKMCIAGAEDPGGRTPRPITIRPLALFTSFLAVMIAAYLFKQ

KRRQPPLPPGPRGVPIFGNLFQIAPKYQWRQQQEWSQKYGPIFKLQLGVHMVIVLGTYQAARDLLDKRWKIYSDRPGFVV

SAKYISGAHPGFRTLLMRGEMWNAHRRLQATVLSPRMSQKYKPVQDLESKHLIHALLKKPDDFARQFHRYSASLVHSLGY

GKRLVTGREEEVQTVDTIMRNFTEAGRVGKWWVDMFPILELIPKYFAEWKRISAKIFEYESNFHIKNLNEALERKGWNWA

KLYKNSSFSQGMSDLEVGYDLGTLNEAASDTTTIALEVFMLAMLKFPHVARRAQEELDRVVGESRSPTWQDKDSLPYIEK

VIQETLRWRPVAINAFYHAVTEDDEYLGYRIPKGSWVVANSWGIHMDPELYPNPSDFNPDRYDDEKLGHVAFGFGLRACT

GKHIAKNSLYINISRILWAFDIGPKMRADGTEVLVDEMAFTTGFLSRPLPFECSIRPRDQGRVGVIETEWAEADKSLLSI

MAEEKFI*

>CYP6271A4|512|Tubme1v2

MALDAVIAYARELAPASISRGAIAATVLGVWLLNRIWLVFYRLYLDPLSKFPGPKLAAATSLYEMYYDIAQDGAFTWKMD

ELHKKYGPIVRVSPYSLRLRKSSAYHEVHRMGTPFVKDVRFYGQFKIYNAVFAIIDTELHRQRRALLNPVFSRKAILDSE

PVIKEKLNHLCRRMKEHEVQDKLLNCYTAFVAFTVDVITKFAYDKSYDILSLPDFDCRIGAAFDSQLEFFLVMKHFPILA

QILHSLPISVINLLMPSGGSFAELELDAKLHLIDLISRMRDGTMKNSTAHRSIFGGILTEHPDLKADPTELAQEAMSVVA

AGVHITRWTLCVGLLEIARDPLIQTKLYEELKMARPDINADFSHVQCEKLPYLRGVILEMLRLSYGVVGPLPRRVPKDGA

VVGGYHLPGNSTIEMDSYSIHHDEDIFPDSRRFWPERWLTPESKQKEKFVTSFGAGPRQCLGINLAMCELYLATATIFRR

FEIDISARGPERMKFTEHMVVVPRDEPLQCKVISRKE*

>CYP6188A2|1616|Tubme1v2

MAKIIRSVESTLQDLHLEAPTQTTGPGNTMAKYLIALLTNLSTESQWDHLRSFIPGILGVVFVYILVKLGLHLHKVRNDT

PLKHVPGPWLASCSPLYRFWYAVLKENFHHHLTNLHRRYGDVVRIAPNEVSIWDPRVTSEIYAHGDKGYAKCDMYDIALP

NGFFNLAVERDIQTHAEGRRAIAKDYSMTTTLMAESHFDNVIKDFILVLDKNFAQKGVVCDFTIWSEYFTYDMITDLVFG

EAYGFCKTGLDVDGSLKDLRQMLLLSPFLSYLPWIWPVTQNQWVKKIGMNHYARVRHIPTNIEKRLNNGNPSGRNDLLDR

LLRSRYRDGHTLPLGEIAHVSNLAAPDTVSVALRAILINLVRNRNIHNEVMAELTGLKLSNPATWKELAGAPLLHAIVKE

TIRLHPPAGFNLPRAVPAGGRTLCGYYLPEGTTVGMSAWCVHANEDFWGKDTLEFKPERWLDPERAFKLDQYGLSFGQGA

RACLGKNIALVQLVKVTAQILLNFEFELVDERKIRGVFLLLVVLDGVKVAFKRRAGGPLDDTVGAEGVVN*

>CYP530G1|1688|Tubme1v2

MAIPVFWTAAPGLALGVCLASFIATIIYFAKGTSRGKNFPPGPPTLPILGNMHLIPQERPYLKFTEWAKQYGGIYSIKIA

KQTIVLISDVKILKELYDKRGAIYSSRPLPHIGAEIVCPDQTHIIYMPYGETSRNYRAQYHQFMGPGKVEQILPWQSAES

TLLLKKIATSPERYYEHTMRMATAVILESVFGVLPKDYDDPEVTELWTVQREFSEILALTGPPVDHFPFLKWLPDIVSPW

RIHARNVRAMHRKLYFRLLNLNKTRMEKGERYGTFVEKLIDDKPKHGLSDERIAYVCATLMEAGSDTTASQALDFMMALL

AFPNVLKKAQEEVDRICGTSRLPTLDDRDQMQYIEACVNEVLRWRPPLPYSAPHLLMKDDWYEGYFMPKGTVLFQVQWAM

NMDENVYEKPQDFMPERYIRNRFGTKFDAEKDAEIGRKEQYGFGLGRKICPGQWFARNTLFVLFAKLIWAFDMKVPTDPK

TGMPLPLDTDVRTAFMDGLTTTPFKFPIEFKIRSKAHEEAMNTDLVASDKVFAKYGGATV*

>CYP51062C1|1707|Tubme1v2

MFIDSIPELDFHGYYFAAGIFLGGIFCMLVWSIINRGTIRTEANGRKCRSPPVLKSKLPFGIDSMLMLMRSVKEFRLPVA

LTELHDQMGHTYANYGLAQERKAHRSLLGSMGIFAVDGVSWSHSRALLRPSFDRTNVSDLGQLEEFMKIFFSRIDELPIQ

SFAHARAVELQELFQCLTMDSSTNFLFGNPIGALASRKYTLGNGEMTFDQAFDIAQYGLAMRAPLSSFYWLYNGAEFRKA

CSTARTQAALYVSRTLRKLNLAEKSEGKKYVFLEELAERTQDPQVLQDQVLSVMLAGRDTTAALLSWTFLCLAKYPEVFK

KLRAEISEVVGVDEDARLPTQAELRGMVYLRWIIQEGEFLKMPALTVSVVLRLYPPVPINARRALRATTLPYGGGPDGNS

PISIRKGERIVYSAFSLHRRTDIYGPDAGEFRPERWGEEALRGIGWSWLPFNGGPRICLGQQMALTHASYFITRIMQVYK
EITPQDSALEGDAYDTKLVMASGRGVHVFLS*
>CYP512CQ2|2038|Tubme1v2
MKDQSMETIMNNDTGSRSGWFKAALEGVLLKRGVDRGVVLGGLVLWMIGWMVWKSYSLRVNVDAVGDSPGPFGGWRAMFK
YFDHGSDWITEGYKKYSHTGKTFKVPTIARYIVFPTSPKLLEEMMAEPEHILSFDEALTEKVAVEWTLHPSIKYDTYHLK
LIRTKLTQRLSIVLPEVMDELTLAWEESTNIGKEWTKVRVWDVMLQIVARTINRMFVGVPLCRDQEYLDNVIQYTVKVVK
AGAILDILPRIFRAPLTNLIMQKSHHYAMMRKHVGHIFSERKEKMRELGSEWKDRPDDLIQWILDLAGDGKASSEEELIF
RLLFMNFASIHTTTSTLVHALYDIAANPELQPPLHAEITGALSKSGMSKQSLTKMKKLDSVIRESQRLNTITTITMMRKA
LVPYTFSDGTHLPVGTWVAAPATAIHLSASIPNPTVFDGFRWERMGAEDQASGKVGKHAAVTTSFEQLAFGHGKHACPGR
FFATNELKILLSHVIERFEFRCLEGKRPKSRFFGVACLADPNGVVEFRMR*
>CYP50043A2|2832|Tubme1v2
MSILNIGLVSSAVYLLGAWMAYLVLLIFYRLSLHPLRRFPGPKMAAVTGWYAGYWDLYRGGQMVNHLVDLHKEYGPIVRC
EPNHLHFSSPEVYSTIYNATGKLTKDPNLYLSFGANDSVFTLVDPTIARTRREVISPMFSRRMVLSLQPLISGKFRKLCG
KFSSYADRDEAADMASGFRSASIDIITQYCYGECLDSLDVEGFKHDILLSIKATSKAFWVVKYFPMDWILTLPANLTLRL
MPELKGFLAVRDNIEAQVKRYMKNPSLLEKSSHPTVYQRFLDPDVKGGIPKASALADEAQNLFFAGSDTVGTTLAFGTYH
ILTTPGLQEKLFAEICQVWPVLEEEPTYDQLEKSAYLTAIIKESLRISHGVVTPLTRVVPASGMTIQDQPIPDGTVVSMD
VPTVHLNPNIFPSPNTFLPTRWLDSNAKDLDKYLVAVTSVYWIEVTTPSTFLFWLPQELMMIVSLGPRCISASREYFAGL
RWRFGRLVRRIWNGWIVSRRSRGGT*
>CYP548BE2|3749|Tubme1v2
MAIFKSSIQSLDRIPITIDIMTFLDLARLLFTSFAVYISSLVVYRLFFHPLARIPGPFLAKITDCRYTVYHAYRGDRHLA
LYRSHEKYGPVIRFAPNLISFNSASSLKAIYGHTALSRSLQKSQFYSAFPAVKGVHNTHNAISKAEHGRKRRVLSAAFSD
AALKSMEDLVLSNIDVFSAKVNDAFKHGVSVDIGDLFSWLTFDVMGELCFGRSFGMLTEETTRFVTNLISQAAHNHYING
NYLPLTTLKLSKILFPTISRDRWKFIEHSRAYADERMSLGRGYKKDFFYYLLDAKDPETGEGFETKELWGEANVLMIAGS
DTTATAMSAAMFYLCRNPQTLQTLKNEIRSTFTDKEQIIGGKELNDCHYLKACIDEAMRLAPPVPGLLPREVIAKGGIEI
DGLYIPEGTVVGTPIYTLHHNPAYFPDAFSFKPERWLSTCTSEEEAEAARSAFTPFSIGPRGCIGKSVAYMELRLTLARL
LFEYDCEEVETEGKGALWKEGHAMVDGEYRLMDHFTSRKEGPVMRFSRR*
>CYP61A1|3918|Tubme1v2
MDEIANLSTFVPPSAVSSAEQPFQTLVEGFETIGKTLEGLSGWQIFFSIVILSITYDQCRYIWNKGSIVGPSLKIPFMGP
FLESVNPKFSEYLAKWNSGPLSCVSVFHKFVVIASTRDLARKVFNSPGYVSPCVVDVAKKILRPENWVFLDGRAHVEYRK
GLNGLFSRQAMGMYLPGQEEIYDVYFKKWLELSKEGKPIPFMSEFRDINCAVSLRTFVGHYISDEAVKEISENYYKITAA
LELVNFPIILPWTKTWYGKKCADFVLNEFAACAAKSRIAMKEGKEPGCTMDSWIKSMMEAKESDAAKVAAAKGLPQIREF
SDMEISMTIFTFLFASQDASSSASTWQFQIMADRPDVMRRIREEQLRVRDGDPHKRLGLDMIDKMVYTRAVVKEQLRYRP
PVIMVPYEVKKSFQITPEYKVPKGAMVVPTLYPALHDPEAYNDPESFVPERWLEGGEAEAAKKNWLVFGAGPHVCLGQNY
AIMNFMAMIGKASLMMDWEHHATPKSEDIKVFATIFPEDDCFLVFKERDPYAPA*
>CYP5959A20|4024|Tubme1v2
MGIFLQLKDYADDKNLYGYSVFGMILSLLSLGLFLLLIKEWSVHGPTNLPIIGVESPGYFARIKARQNFVFNGFQMVRDA
YYKHYGNNFVITTNSYDKVILTHAQVRELCNAPDDVVSFSHANAKLLMDKYTGMARFVETQYLQDATRIKLTQNLGNMLG
AQLEETHLALDSELPGSATNEWLSVDLFPTILRIVARVSARSFIGLPVCRNEDWLKLAITFTEDLIATASNLGTISKLFR
PFYAYMWNSAKTIKSHKRKAQTLLAPTIQKRFKEQRLAEENGTVYQGPNDLLQWLIDRVEPHHKTVDALSELQLLIGLAS
VFATAGTLVGMIFDLAEYRDCIQPMREEMEAAISENSGALDRGVLRKMRKTDSFLRESMRMGTLLLSFNRMVMKNVTLSD
GTYLPKGTLVAAPALMFSSDPDFVEDPETFDGFRWYKKTLEAKDGVQMSNGSATTSSNNLAFGHGKHACPGRYFAVEEIK
TILTFILLQYDIKYPEGQSRPPNIRIGEIASPNRTQKIMFKKLPGPKTFSFL*

>CYP5093H2|4339|Tubme1v2

MIASAPQMPQSSPFTSSVEAPLASLVTSLHLSSRLEFVRQYEGAVIVPKDPVARFHSLSCLAACVLLSALAVSCLLVALQ

DYRRHAIMPPGPSPVPFVGNKWDLPLQKPWYKFKQWTDMYGSLVTVWTGRRPTIVIGDPKVACDLLDRRSVIYSSRPRFV

VMGELFTNNDSLLTMPHGDKWRKTRKNFHMGLHRRACESYKPIQEAESQRLTRDLLVTPEVFGKHLERYASSVMICVAYG

RRVDSLEDPIVKKIYDRMAYMSTLNVPGAFWAESFPILKLIPDCLAPWKREVKRRAKDSTEMLSRLALDVRDRMKKGDAP

ASFTKTLWERREGNPEALSEREIAYATGSLFGAGSDTSSATLMSFFLAMTCFPRVAAEAQEELDRVVGRDRSPTWSDEPN

LPYCRAVIKETLRWRPVAVMGGTPHASIKDDQYNGHFIPKGATILGNLWAIHHNEKYFKDSHDFIPERYLGSGKIDRMEP

YPHRDGHSAFGWGRRICPGKQLAENSLFITITRVLWAFNISKATDKHGQEITPNIFAYTDGFNSKPQPFQCRIRSRTPGI

QQIIEREARLGEQFLDKYECN*

>CYP5945C1|4408|Tubme1v2

MFLLDPYRQATIAACAAFLTQQVLSPIPEVSTTKSILFYTIANGLLVLALKRAGNCMLHLLIRVSLINISYLTSLAFFTI

VHRLYFHPLRKFPGSKVARLSKLYEAWLNYNGMNGRVVRDLCRKHGDFIRTGPNELAINNVEAVEIIWGRTQPRSRGPFW

EFANFIGERHLVSQRDKAIHASGRGIHIRRTYSDTXXXXXXXXXXXXXXXXXXXXXXXXXXXXXXXXXXXXXXXXXXXXX

XXXXXXXXXXXXSHGTLLYFIFSPRVEYHVDKMISILEKLKGEETNSVNIYDNMAFDVMADLGFGQKPEESMQSGAGDPS

YMDFLHGWMRASTVLTSLRNLCELGAYIPQDSESKEFNKLGEKMLGARQRMGKFRQDIFTHLLNEDKESGVNFTQAQLLT

NAQMLMVAGSDTTSVTLTCLFRLLSMHPEKQQKLYEEIIEAFPNGETPTCATTAALPFLNGAVQESLRMWPAAPSGPQAT

TPPSGYTIAGTFVPGNVEVRIPHMTMLSDPRYFPRPDEFLPERWTPEMPELVQDKRAFIAFGFGAHSCIGRPLAMNEMRT

ATARVIQRFQVQLGESYDDKVFRDEWKDYFTVKLGPCPLKFVPRKM*

>CYP6608A1|5282|Tubme1v2

MPSLLLMSLAPSLKTLVVLEFTQVLPLALLALVASCAYSRYASPLKNVPGPALASFSRIWLNVGLVARGNLHGGIMDAHR

KYGPVVRVGPNKVYFSDPALIPEIYGVGTGYRKSDLYMMSDPEPGHGSTFSERSAEKHAVLRRRVGPAYSNSSVLQMESY

VDALVSAWISALSSEFTVAKTSLKSCDFASWSQFLTYDVISELAFGKAFGFIATRSDYKQYIASFQQALPMIVTMSYFEE

LVKFMTLGWVRKFTTPSIKDETGYGNLTKTATDLVRERWAKGGVGERRDMLQRFYEEGMTEKEAMIDSTLILVAGSDSTA

TTIRAAVMFICSDPSVYRTLKSELAANIPRTSPPSVVSFSVARSLPYLSACIKEALRLFPPGAGAMERVVPVGGATLSGY

FIPENTVVCLHPWPVCRDKVFGEDVDSFRPERWLEEKDEKRLVKMEKTLDFVFGAGPHYCLGRQIAYLELYKSIAELFLT

FDIGLSNPMRPWKSREYGFFLQRDMMVKIKKVDG*

>CYP5959A3|6154|Tubme1v21

MGVFIQLIDSVEEKKLYGYSLFSTSLALLFLGIYILLKKEWSTHSGTKFPIVGIESPGYIGILKARLSFVANGYKIVRNA

YYKYPGKNFVVTTYSYDKVVLTHDQVKELSNAPDDTISISHAAVETMMGDYTGLDQFVGTTFVEDVVRIKLTQNLGSMNE

AMLEEARFALNTELPECTTDEWRSVEVFATIIRIVARMSARAFVGFPLCRNEDWLTITMVFTGHVFMTHSKLACVPKPIR

PIYSYVRNLVKDIAEDKRKAESLLAPVIRKRLEEESLAKKNGTVFQKPNDMLQWLSDRVDQKHKTTKGLSELQLVLSLAA

IHTTSISFLNAIFDLLAHPECIQPIREEMEAIISANNGVLDRVALRKMKKTDSFFKESMRGKIGLSMFLIPREVSFNRKV

LKSVTLSDGTYLPKGTLITAPTSMFSSDPDFVEDPETFDGFRWYKKSLEAEGNNNYWATTSSNDLAFGHGKHACPGRFFA

TERMKTILIFILLQYDIKYPEGESRPENIDHGEFSYPDTTKQLLFKKLPGPKKYSFF*

>CYP663N1|6473|Tubme1v2

MSLIRVIPSLPGGSAAGLILLVLSVGILAQRLFKKKDPLPLPPGPPGLPIVGNSFQIPLINPWQKQAEWTKKYGSIYKLK

LGKDTVIVLGTQQAAKDLLEKRSKIYSSRPRTVMAGENVSRGKKLLAHLIIALLQRGDKWRVHRRLQGQVINQNITNKYK

GFQNLESAQLIKELADRPEDFFDSFHRYNSSVIFAMAYGKRMPRGDEEDVVAVDEITQNFLYSARLGTWIVDSFPFLNYL

PTSMAPWKRIGDNFYNWAEKMHTFNRNEALQRKGWNWTKEIARMKEAQNVSPLELAFMVGFLYEAGSDSTTIALEVFILA

LLKHPEVLKRAQEEIDRVVGPDRLPAFEDRDNLPYVRNCVNEVLRWRSPSVGGVPHMVEEDDEYMGYRIPKGAIVVGNLW

SIHQDPEVYPNPTKFMPERWDDEENVHYGFGFGRRACPGRHIAINSLFINCARIIWGFNVEHAKNSDGTVIPVDEWDMTQ

GFMSRPVRYKASITPRDAKRVEVINQAWKAAEVQLQDMSKILRMEDF*

>CYP5959A7|6603|Tubme1v2

MGIFIHLVDSGEVKNLYGYPIFGTTLALLSLGIFLLLFKEWSIHGPTNLPIVGVESPGYLARVKARRKFVSHGFQIVRDA

YYKHYGKNFVITTNSNEKVILTYNQVKELSNAPDDTVSMSHSSAQTMMAQHTGLSGFVGIHYVREAVKAELNKNLGNMIG

DLLEEAQFALRTELPGFATDGKMLITLTTTEWVSVDIPAVALRAVARISGRAFIGLPLCRNEDWLALSIAFTTDVFQEVN

KLTPVPKLLRPFFAYIWNSAGSVKSNKRKAQSLLAPVIQRRLEEEGLAEKNGTVRQNHRDLLQWLTARVTPDHKNVNVLS

GMQLMTGLASIHTTGTGFTNMIFDLAEHQECIQPIREEMETLIAANGGVLDRATLRKMRKTDSFLRESFRTKLSLLGFNR

MIMKNMTLSDGTYLPKGTLVAAPAAIFSSDPGFVEDPEVFDGFRWYKKSLGVADRAVDNNNASNTSPTNLIFSYGRHACP

GRYFVVEVMKIMLAFMLLQYDIKYPEGQSRPPNIQMGEFSYPDRRQKILFRKRPGPKKFSFL*

>CYP6592A2|8024|Tubme1v2

MNAANTPKLWPEYTTLSSIDSFLLSLVLLPILITIVYNRYYHPLSPFPGPFLASIWSGWRDWENYRNMEDQEPLLHERYG

PIIRISPNTISVADPDCLKEIYTRTNFPERRRSFPHNGTVDAYPLGSSVLLHTELRKGIMLDYSVGTAESWESIVDERVR

ERVGKLKAVYSGSGVRFGFSEWARYLSVDLVGWVVFGVDMGCTQNGRDSGGHILGSKSGLGVAGGLARFPNIYRTLASDS

FIRNILMAKADKEEGLGVMREKVGLIIDAAWKSREEAEARGEEWHLNDKGTTRGVTLLEGAMSAKGSDGTRLTKEEVHSE

CFLPFIVGQDSTALAITNPLKLLLANPRVLATLLSELTTHYAQKPLSHIPPWTDIVDLDAKLPYLSAVLRESLRLHPSFP

VGSPGMVPPSGITLTHRNRLYTIPGGYEISVSPIVIGRNKRIFGEDAHCFRPERWLEGNEKEIKRMKDVGFEWGTGSSGC

LGKALAQMAVAKAIVMVLRHFEVELAVVIREGRCGG*

>CYP5959A19|8074|Tubme1v2

MGIFVQLIDYVEDKSLHGYYLFGTTLALIFLGLFLLLIKEWSIHSPTKLPIVGVESPGYFARVKARQKFLFNGFQMVRDA

YYRNYGKGFVVSTNSYDKVILTHAQVRELSNAPDDTLSLAHASARTLMAEYTGMNRFVDTEYVRDATKTKLTQNIGNMIG

PLMEETKFALDRELPACTSDEPVPVDIVTAVIRIVARVSARSFIGLPLCRDEDWLTISIAFTGDIFKTAMKLNAIPKLIR

PFYAYIWNSAKIIENHKTKAQSLLTPIVRKRFEEKKLAEKNGEAYQNPNDLLEWLTDLVEPRHKTVDALSELQLGVSLAS

IHTTSSTFVNTILDLAEHQECIQPLREEMENAIIESGGVLDREAIRKMRKADSFFRESMRTRSFLCKSITAISLAPVTFN

RMAMKNLTLSDGTYLPKGTLLGAPNAMFSTDQGFIDNPETFDGFRWYKRSLEAGDTAPKGNGCAATSASSLAFGHGKHAC

TGRYFAVEEMKIMLAYFVLQYDIKYPEGQSRPDDIVVGEFSFPDSTQKVMFKRLPGPKKFSFL*

>CYP5959A19|8076|Tubme1v2

MARFANTQYLQEATRIKLTQNLGNMLGAQLEETQVALDAELPGFPTNEWVSVDFFSTALRIIARVSARSFIGLPVCRNED

WLNLAIAFTEDLIATASNLGTISKLFRPFYAYMWNSAKTIKSHKRRAQTLLAPTIQKRFEEQRLAEENGAVYQGPNDLLQ

WLIDRVEPHHKTVDALSELQLLVGLASVFATARTLVTMIYDLAEHRDCIQPMREEMETVISENGGELDRGVLRKMRKTDS

FLRESMRMGTFLLSFNRMAMKNVTLSDGTYLPKGTLVAAPALMFSSDPDFVEDPETFDGFRWYKKTLEAKAGALMGNGSA

TTSSNNLAFGHGKHACPGRYFAVEEIKTILTFILLQYDIKYPEGQSRPPNIQIGELASPNRTQKIMFKKLPGPKKFSFL*

>CYP567U2|8431|Tubme1v2

MMATLHRFEGAALLVQQVVKDVPAWKLCAYVTLLFPLYAITMGIYNLFFHPLRNFPGPKKAALSNIWYSYIWLSGRYPHI

IHALHEKYGSVVRVAPNQLSYNTSSSWKDIYTNYGNRQTFRKNSLYDRDKLDPVTNISREPDPVKHNQIKRLFSNAFSMK

SLTEQEPIVQEYVDLLVSQIGKHGTGEDGLDMTKWYNYCTFDIIGDLAFGEGFDATKEGKPHFWISLILDSVYAISFINV

TGRFPWLSKFVPSITPAEIVERRQRHINYGRDKVNRRVKSDNKRKDFLTNVLENYRDQISDAELYSNAQVFIVAGSETSA

TTLSGLTYYILRNPRTYQRLVSEITSSFTSYSEINSVTAGRLEYVGAVINETLRMYPPVPIGLPRLSPGETVDGQFIPKG

ALVSTSPWASGHCSKNFHDPWTFKPERWLDGECEEKDIREASQPFSLGPRGCLGRNLALMELRLIICKMLFTYHLELLDT

KLDWERDSTAYILWVKPDLRVRLHRR*

>CYP6136A1|9536|Tubme1v2

MFLNLLGRAVYGLMQHKLYSLAGILILTLAYWTHAYLTSPFRKQNIPGPFLGKFTNAYRWYYVMRHTWHRDLMDLHKKYG

PLVWIAPNDISVSDPSLRNVIYGFQNHKKDFTFFRKSHSYETGSINQDFSFIFEQDPEKARLGKYYMSHFYSEQGLLNLE

ENFDKAVNELIEGLDKHHARTGTPCKMVDWAEFFALDLVAQITVDRSAGFCLAGKDVNNAAYGMRVIIKTVGALMPLPWV

LSATSRAIRQNLLLRFLINLYRNVLLLPTFTFDTGIADLKTLGEKNPKHLLAKFYNAQSKMREHYPHGNQAEGTTIQVFN
LMYAIHPTRLFFARLIIILHSAGAVGAVPHTQVKLIHELTLHPEILQKVREELATTDNTFHLEDFIRHNGRQNKYPIFES
AVREAVRLSPAVAFSLSRKVPPSGCQLHQYHIPPGYNVGMAAYQVNYDEGYFGPDVAQFRPERWLEDHPTEMLEGEKRSM
KNYIEAGWLSFGSGGRVCIGRHLAMFMMMKFTAAVVREFDIRVVKQPVEHHTLVTEMLGMEVLLSRKCAVRVGQHDG*
>CYP6135A17|9582|Tubme1v2
MLASIFLTLSLLFVGYACYSVHTLVLKVRPLGIPYFVIPFYPSPLPRIFLVPFVNLLINVFNLTHVWFYLLTLDWQVRQR
YEIYRFIGSDLFFTITPWEVILHVADPDMAVEVLSGKGQNGELYPRTEIVGRMMGLFGKNILTAEGAVWRGHRKVTAPVI
GNSLSIVWEESLTQVKEMLQYYDKQEDIKTIHRDFRRVTLGVISHAGFSKVMNWVPLTTTITSGKDGEGSYQKCLDILFE
NFEWTAIAPSWLLEMLPVKASREAGGAVNRFRFFTNEWFEEKKAKVELDNKTTLKSSSRVDLMESLVRSSGLDEGSTVET
PLLSKSEIFILAGHETTGHTLANAIYFLAMYPEYQVKLQNQIDSVLGSTNHNETSYDKHFDAFSSGWMAAIMLETLRLIP
AAIVVSRQNGPTARTFPLSGRGPITLPPGIQFWVELIGLSHNPKHWTLPGKTEAESAISEFRPERWLPKSEGDSMFKPYN
GSYIPFSIGARACIGKKFAQVEFAAVMVGLFREMNVEFDLCGGKKSFEEARRECLMQMERYETIITLRPTGEMPGIKWVR
RKK*
>CYP51F1|9803|Tubme1v2
MGVLSTLLVPMGPYYEELMRLGGPALCGIGFIAFVVLSVIINVLQQLLFVDPTKPPMVFHYFPFFGSTVMYGMDPYKFFS
GCREKYGDVFTFVMLGRKMTATLGPKGNDFVFNGKLSEVSAEDAYAHLTVPVFGEGVVYDVPNHVLMEQKKFMKFGLTTE
NFRSYVPLIVEQVEDYIKKSKFFKGAKGSVPLSEIIPELTIFTAARTLQGKEIRDALDGSFATLYHHLDSGFTPMNFLFP
WFPFPQNKRRDHAQRTMARFYMDKIKKRRAIEKDDVGQERSDMMWNLMNCSYKDGRKVSDKEIAHMMIARTLGSKPTRIV
ARILAEQKRVFGDELAPLTYEKLVLECTFLAQILRETLRMHPPLHSILRKVKSPMPVDGTNWVIPKGHYLLAAPGVSAMD
QKYFKEPDRFDPNRWDDQKPEEEAEKFDFGFGLISKGTASPYLPFGAGRHRCIGEQFANVQLMTIMATFVRNFEMQRPGG
GDDVPAPDYSSMIALPTPPCIIEWVRRDP*
>CYP50026A1|10007|Tubme1v2
MDQATELLSRVEHILLKPLYHSSSLHALAAFLVWYVVYQRYFHPLRKFPGPFLASLTIFWRLGNILTFRQSLNDHALHKK
YGPVFRDGPNSLSISDPRALEPIYGTKNELNKTPWYLVMDPDNTGEDYSVFSSRRAEQHKRLKKRIAGAYSMTSVRVYEP
VIDRNINDLLAWIKELKTLDVSVWTHYFAMDSMSEIAYGNRMGFLINGTDVNGYRKALHESVVWIATMGYLTGLNYVIKS
KWLSPYLAPSPKDKHGYGHMIGMTEALATNLLENGNTTGKRNMSHDLLQCRNDDNTPLSKKELVGEMLAFTTAGSDPTAY
EISAILDRICRHGDVREKLLQELRAVGELEQSSAEGVVTYAQTLRLPYLLAVVKETMRLNPAFQGQFSRVAPEGGCGGEV
GGLEVLPGVVVPGGVWLSINTYVSQRDKLVFGEDAEEFKPERWLPIGGDRYHAMSKYLSVFGYGSTACMGQHLASQKINK
TVVEILRRFNVELKDPKTPLKERNIIQMFISDLFMTFTEREKKI*
>CYP6001C26|562|Tubme1v2
MASNGDGTNGATNGDSKYATQSKPPPPVAKPTRAAVDSTFEQFSNLLHASNRPLPNRFGDGKDQRPPELKPTGVLTDINT
LRRGGFFWESVGTLWDFLKQKAKGGAVDDKTMIMERVIQLTSRLPPTSKARVALTTLQVDQLWDSLQHPPLSYLGDEFSY
RQADGGYNNIMYPQLGRAGSAYARSVKPMTKMPGAPPDAYTLFDRIFSRGPNDEHFREHDNNVSSMLFYTATIIIHDLFR
TNRENQNISDTSSYLDLAPLYGSSVEDQSKIRTFQDGKIKPDSYSEKRLLAFPPGVSVLLIMFGRFHNYVVENLKAINEG
GRFNLKFPRYPNGDDEVTRQAKALRQQDEDLFQTGRLVTCGLYINFILNDYLRTIVNLNRVDTTWTLDPRFEASKVYNPD
GTPAGIGNMVSAEFNLIYRWHSCISKRDDQWTKDFYQGLFPGRDASDIEMPEFTRGVGRWERSLSDDPIQRNIAGLERKA
DGSYHDDDLVKILTESVEDVAGAFGARNVPHVLRLVEVLGIEQSRKWMVGSLNELREFFGLKPHATFEDINPDPAVANTL
RQLYDHPDFVEMYAGLVAEADKKPMVPGVGIGPTYTISRAILSDAVTLVRGDRFYTADYTASHLTNWGLQEASSDLATVY
GCVGYKLILKAFPNHFKFNSIYAHYPLTIPQENHKIHTALNSVDQFDFERPVYTPPRIPISSYNATKQILCDAENFKVTW
GAGFDFIMRADFMLSGDKPSNSEQKQFVRDRLYLGGVDWKQQIRQFYEEATEKLIRKKAYSLGDTYQVDAVRDIGNIAQT
IFAASIFNLPMKSEDHPKGIYTEQELYMILCVMFIVIFFDIDSSKSFPLRQAGFKVVRQYGTLVEAQVKAIKNWSWLQGV
WDPLNIRGRNKSSPLKSYGWTMIKRLLDTGKSPYDVTWSYIVPTAGASAPNQGQIFAQVLDFYLEDRNSHHLAEIQRLAQ

SGAVDAWETIKKYALEGGRLAGTFGLYRRVEPDNITIEDNGRNIELRKGDMVFVNFITASRDPVVFPDPLEIKLDRPEAS
YMQYGDGPHECLGKAANIIGLTTMLMQFGKLKGLRRAPGPQGALKYIPKPGGFKVYMKEDWSAYWPFPTSMKVRFDDII*

>CYP6220A2|1367|Tubme1v2

MIDSEYFLNGISNMEYLTAAICAFGALIVWKLISAIFFSPLSHIPGPLVTAVTPHYINFLSALNQRTVGTYSLHKRYGPI
VRLSPTEISILSPQAIKEVYSSPHYTKYTPLYSIFTHFGAQNSFTSCTREEHGWRRKAVSEPYSLSFVLKDEAATGKVLK
AVKDYLGFVESDRRVDIYNANTFYATDVVTGKIFGLEASMKTLAGNEEHREIVLGHYARTRRTQVWMYIEFPLIMNLFEW
FAFYTGRVWSWIHGEEIEGWEMVSQAQRWGWDAYMDAKSNSREGTVAGRLAKLVQEGGSGEEAWDDRGAVSEVMDQMLAG
MDTTGDTLSFLMYQISLPESRSVQKRLHSELLNAFPKSAEMPGLGWPHSMDVQSLPHETILKVLNLPYLDAALKETLRVY
SAIPITLPRVVPASTTTDRISGHRNGRILEGKFIPAGTTIGTLAYGIHRDEVFAVETKQKDEPGDVDSFVPERWLINGGI
EGKLTPDELEQEKQRIRTMEARLWAFGSGGRNCVGRHLSILEMKLLLATIYSRYQTQVTPNSGVKITHNRWDQRRTFRDV
LPFRRVDGVVTFTPYEY*

>CYP6220A3|1368|Tubme1v2

MIDSGYFLNGISNTGYLTAAICSFGVLIVWKLISSIFFSPLSHIPGPLVTAITPHYINFLSALNQRTVGTYSLHKRYGPI
VRLSPTEISILSRQAIKEVYSSPLYSKYTPLYSVFASFGAQNVFSSSTREEHGWRRKAVSESYSLSFVLKDEAATGKVLK
VVKDYLSFVESDRRVDIYNANTFYATDVVTGKIFGLEASLKTLAGNEAHRKIILGHYARARRTQVWMHTEFPLIMNVFKR
VTFYAGMVRSWVRGGETLSQIQQWGWDAYMDAKSNSRVGTVAGMLARLVEKGTGSSDEGVWDDRGAASEAMTSDTSYFRM
FGYGGDTLSFLMYQISLPESMNVQKRLHSELLDAFPKSAEMPGLGWPHSMDIQNLPRETILKVLNLPYLDAVLKETLRVY
SAIPITLPRVVPASTTTDRISCHGDGSIVEGKFIPAGTTIGTLAYGIHRDEVFAVETKQKNKPGDVDSFLPERWLISGGD
IESELTPIELEQEKQRIRTMEARLWAFGSGGRNCLGRHLSILEMKLLLATIYSRYQTHVTPNSGVEITHNRWNISTTLRD
VQPFLGAAGVITFTPYEP*

>CYP539A49|5365|Tubme1v2

MLFGAVVQSAPGLLIAAVVAGVLTSAFKRVRENWKIHKLGGRARSLPGRWILFGLDEIKRTLVAAEANKNMELWESRFKT
VGSHTVEVSIMGTRVIFTEDQENIRAVLASQFHDYGKGESFRQDWKSFLGDSIFTTDGQLWHASRQLIRPQFIRGRVSDL
DTFERHVSHMLEHIPRDGAIVDISDLFYRFTLDSATDFLLGHSVDSLGSPQVEFARAFADIQKHMSDKSKADPLRWIFPE
GKYRKDLKVLNSFVEPYVEQALRMGPEELKSKNEKSYNFLHALAEFTRDKHMLRDQLVAVLLAASVIICLFAPNDTIAAT
LSWTLYEIARQPEIVRKL*

>CYP6001E1|5372|Tubme1v2

VFPKYRSTSAITLFPLEQFKMSFLGLFGGGSSANPNSEYGDESAATKEITYTGIAEDIKASGGKIPEDLKLLLETGAQKV
SKGPVDDKQLVMERLIGLVASLPQNSANRKKLTSTIIDTLWDSLQHPPLSYVGDKYQYRQADGSYNNILYPDLGKAGTEY
ARTIRQDKKLYGAKPDAGLLFDLLMARGDNFKQNQAGISSVLFYHASIIIHDIFHTNRKDFSKSDASSYLDLAPLYGSNQ
EEQNLIRTMQDGLIKPDTFSDKRLLGLPPGICVLLVMYSRFHNYAAKTIKAINENGRFSLPASHATASPEDQAKNLAKLD
NNLFQTARLITNGLYVNISLHDYIRGIANLNHSESTWTLDPRVEIDKSFDGEGTPRGVGNQVSCEFNLLYRFHSAVSKRD
DAWTKDFFGKIFPGQDPASIGISQLLQGLIVFEKSISEDPAKRTFGGLKRTGADGSGAFNDDELVKILKESIEDPAGAFG
ANTVPEILKPVEVLGILQARKWQVASLNEFRAFFNLKKHKTFEDINPDPYVANTLRKLYDHPDMVEMYPGMFLEDTKPRM
DPGMGLCAPYTVTRAVFSDAVTLVRSDRHLTLDYTPANLTNWGITEVAQDYDTLGGAKMFHLILNAFPSYFKYNSVYAMQ
PFYTPTESRKIFDKFGKSYLYSFDPPAKTASPIPILTHAGLKRVLNDQKNFKVPWGEAMEALNNEHDFMLAGDLSSHTQQ
RNLVGDAIYDVTGSRKQFKDYTEEITLKLLKREVYQLGKDPFNQVDIVRDIGNLAALHFAASLLYLPLKSDENPNGQYSE
QELYKTLTDLTWFVFSDSDPTKSWEHRREAKKSIDKLGEIMVEEVKKFKTPIGIWDKLTGGTGVRPPPPSLKDYGSNLVK
RLLGSGKSAEDVAWMLLWSGCAFVANSACAFAQLIDFYLQDDNRKHWAEIQRLSSLNTPEADKLLTKYTLEGTRLSNSLG
IFRTVDPVDTQTITIKQLGQDVVLKKGDKVFVSFVYASKDASVFPDPLDIKLDRPAELYVTHGEGQHQCLGKDINIIQNT
YMLKTLAKLRNFRRAPGDEGKLKFISKPGGIKLYMNADWSKFTPYPTRLEKGVFRG

>CYP52AV2|7486|Tubme1v2

MELAFKLLFPLATAFYLLPKLIACLIIAHQNRKFAKSHNCLPPRRLPSSFLGLPNWVRLMRAAKRGEALEHIANRYPTYG
NTWKGRVLIGTTIGTIEPENIKAILATSFKDFSLGPERHDNFYPLLGDGIFTLDGAGWEHSRANLRPQFSREQVSDVEAL
EVHVQRLMDRLPGADGEAADLQPLFYCLTLDSATEFLLGESVDSLLSPKLIPTGTISRGKEEMSFAQAFNVSQGYLIQRA
RLRGLYWMVNPKKFRDANAIVHRLVDRYVDMALNPEKRTRKVSENKYVFLDAIAAETKDPKYLRDQILNILLAGRDTTAG
LLGFTFWLLARHPHIYQKLREKILEAFGTGGDGEGKRPSFSALKDVTYLRYVLNETLRLYPSVPLNGRTAVRNTVLPRGG
GEDGLSPVFIPKGQRVDYTCYGLHRRKDLYGEDADAFRPERWGEGVGRGWDFLPFNGGPRICLGQQYALTEASYTVTRIL
QKYARIEVADTYTGPMMDLTLTIAPKEVLLRLWKA*

FRAGMENTS

>CYP617-fragment1|359407|Ascni1
MPRLLPITLLSLTFAYLLPRHYPALLSPSPAITFLTVFLPLLLTSALYHVLLYPRFLSPLRYLPGPKPSTLLLGEFPLIY
RLPTGFPHITWQKLPNDGLIRYLGLWNTERLFPTSIEMLKEILHTQADVFVKPNTLGGLSTILGKRGLFFAEGEEQKHYR
RMLLPSFGVTHLRKLVPVFWDKAMEMVDCIIREAQIPTEDETVTDEEREKGRLVDISKWASLATLDIIGTAGFGYDFNAL
KNGEEANELSNAYAKLLKRDRAAMGWIMLMMLLPWWLAYWLPTESARTIRKSQKTFQRVSTQLIRERREKQLSNPQSKDS
DIKDNDILSVMLRSGEFDTPSGEEIARDQIMTFLAAGHETTATALSWALHALTLHPAHQSLLRTELYTLFPRGPPKALTH
ADLTQLPKLRNFCLEVLRLYPPVPLTLRTAKRETILNGGVRVPKGTMVVVVPWALHRSTGLWGDDAGEFRPGRWEGYGQP
GKECTINSWMLQTFISGPRSCIGEKFSLWEFMVLVTGVVGRVEVRERVEGVGRRLEEMGSIVEIQGGITGKPKDGRVWVK
EVEWGSSRGEKEE*
>CYP-fragment2|1788484|Chove1
MAELWTYYYAESGDPQDPGEVLQHAKDDGKDDYFRITVASQYPTKVLVQVPWIVRILALMPWLNKEYYKFLAWCKDIVDE
RKEKTVDQPDIFSWLIEEGPLNVGPHKVSMAIESRTAIIAGRSLLFLISHRNLKPDNFQRHHNPLLSPAQNPSLQQRLRR
ELDSIFKNSHNWEFTILSDIGYNAATHTPLLESCIKEALRINPPFPPG*
>CYP663M-fragment1|1820648|Chove1
MLFPAFITTAKSALDSQCRDDIPTFTDLQNLPYITAIVQEVLRWCTVVTAGFTHATTTPDEYAGHHIPTGTIVIPNHWWV
HCDPAIYRDPAEFRPERCLGEAEGGIGKNAAFGLGKRVCLGQYLAVQSRSIVIARVLWGV*
>CYP663M-fragment2|1821555|Chove1
MLFPAFITTAKSALDRQCRDDIPTFTDLQNLAYITAIVQEVLGWRPVVTAGFTHATTTPDEYAGHHIPTGTIVIPNHWGV
HCDPAIYRDPAEFRPERCLGEAEGGIGKNAAFGLGKRVCLGQYLAVQSLSIVIARVLWGF
>CYP6470B-fragment1|338407|Chove1
MKAIPGAEKTIFKILADCYSRMSRLRKILAEQSFGMEVLTLGNIALAITTCFFLHCFKQRFFGPLRHLPGPWWSHYTQFP
DTLHLLAGDRGLYVHSLHERYGDTFRLGPNTVGATGREAVKTIFGGGSKRPFHKEPVFTQMFNFRRTPEENIAGIHDPKS
SAKRRMMYGNVYSRGNVLNMQDIYQKCFMNFITKLEECRKNSHDGIVGVISHFRAMAFDVLTEAAFGGLYKGTGYGTDVV
DILDSVMNANQFQFYFGTPIYNLAMKIPWKKLDWLRTKEEFCDKRPPGKPNSKQPRPVKTLRRSVREYFCRDRQPRTLSA
SSP*
>CYP663M1-partial|1574287|Chove1
LPPGPPALPIIGNIHQLPPTDLWKQYAAWTAQYGPIFKLQLGSALVIVLGTAEVARELIVKRAARYSGRPRGIIPNAHFS
RGLRPVLLNDTHPHWRLSRRLHAALLSNSESSKYQSFQELESLQVIVDFLHRPDAFAKHVHRFAASLMFTLCYGKRMVTG
QESDVVELDKMVRNFVQAGEVGRGIVEAFPILEWLPKCVAGWKREGEAGFDVARTIYLANYREAKVREGENWVKKVQGAS
YVETLSELEVAFSVGVLYEAGTDTTSASLETFFLAATLFPAFITTAQSALDAICPDRIPTFTDLQNLPYITAIVHEVLRW
RPVVISGLAHATTKPDEYAGYHIPAGTIVIPNHWGIHRDPAVYADPEEFRPERFLGEGEGGMGKHVAFGLGKRACPGRYL

AVQNLGIVIARVLWGFDIRAGEKVWEGGFTQGIVSKPVGLGVCVEPR

>CYP5945C-fragment1|1769406|Chove1

MSLLDPYHQATIAASAAFLTQQVLSPIPEVSTTKSILFYTIANGLLALALKRAGVGILYFFMHMTLINFSYLTSLTFFTI

VHRLYFHPLSNFPGNKVARLSKLYEAWLNYNGMNGPVVRDLCRKHGDFIRTGPNELAINNAEAVEIIWGRTQPTARGPFY

EFANFVGEKHLASQRNKAIHASWRRIWDKGFTSQTVVSYSPRVEHHVDKMISILEKLNGKETNFFFLLNPWSYS*

>CYP50026A1-fragment1|1803036|Chove1

MDQATALLAWVEYIFLKPLHYSSLQALAASLVWYAVYQRYFHPLRKFPGPFFASLTVFWRLSNILTFRQSRNDHALHKKY

GPVFRDGPNSLSIADPRALEPIYGTKNELNKTPWYLIMDPDNTGEDYSVFSSRKAEQHKRLRKRIAGAYSMSSVRVYEPV

IDRNINDLLARMKELKTLDVSVWVHYFAMDSSMLLCFTRSPVTSETSH*

>CYP676-fragment1|1810205|Chove1

MTDIEIVATSVGAWSDTVSVTLQAFVYYVFRNPEAVVRLRREIGGARLQGEVVSYADAKELVFLQACVGLVSSRYVGV*

>CYP-fragment1|1818363|Chove1

MPVENFTDVTLPWETVVFLLIELPGLYIVYGSFARQELALRLPPGPPTLPIIGNIHQLPPTNLSSSM*

>CYP-fragment1|1823923|Chove1

MPVENFTDVILPWETVVFLLIKLPGLYIVYGSFARQGLALRLPPGPPTLPIIGNIHQLPPTNLSSSM*

>CYP6135A-fragment2|167558|Chove1

MGSSNHSETSYNTHFDAFSSGWMAAIIVIGRNPPPHPRSGSSNPPTRPGSAHIPRSSQPPITLPPGVEFWIEIIGLSQTP

NYWTIPGKTESQSAIREFRPERWLLKSPNAGMFKPYNGSYILFSIGTRGCVGKKFAQMEFTSVMLGLFREFTVEFGTLGG

TKTQEEVKKECLVEMERFATIITFRPAGGMPGARWVRREKKVVGTGDLD*

>CYP50026A1-fragment2|1675317|Chove1

MTEALATSLLENGNTSGKHNMSHDLLQCRNDDNTPLSKKELVGEMLAFITAGSDPTAYAISTILDRICRHRAVREKLLEE

LRAAGELEQSSADGVVTYAQTLRLPYFLAVVKETMRLSPAFQGQFSRVAPDGGCEGGLEVLPGVAVPGGVWMSVSTYISQ

RDKSVFGEDAEEFEPERWLPIGGERYHAMAKYLSVFGYGSAACMGQHLALQKINKTVVEILRRFNMELRDPKTPLKERNI

IQMFISDLFMTFTEREEKI*

>CYP677-fragment1|1777371|Chove1

MIKEIQRVSSAVQSRLPRIVDRNGITLAGTEVPPGTIVGMSLHQVHHDPKIFPSPEVFNPERWLGDNAKRLENYYVPYSR

VGRQCLAVS*

>CYP6592A-fragment1|1785992|Chove1

MRVPPNMISIADPDYLEEIYTRTDRTKQWLPFAHRGTMDALSNEGHIEGFESGLVTIGLLCRLPNVYRAPTCNSFVRNIF

MAKADTEWVGAMRVKVGVIIDAAWKPREEAEARGEEWYLDSIGPRQGVAMLEKVSIFDYLSHPTPCISTILTADKAMSAR

DSDGTGLTKEEVHSECFIRFLAGQDSTAFSITSVLKLPLTNPHVLSTPLSELKTYYAEKPPSYIPPWANIQDLDTKLPYL

SAVLRESLRLYPAFPTGIQRMVPPSGVTLTHDNRTYIIPGGCEIGANPIVPGRNKRIFGEDAQCFRPARWLEDSEEGSKK

VRTAGLEFSAGSRV*

>CYP52-fragment1|1806741|Chove1

MSRNKRVWDKLQQEVLSAVERDERPDFNQGKDMKYLRCVLNETYHNTTLPAGGGPDGQSPILIPAGKKVIYSIFEFHRRK

DIWGPDVDELVPERWEDGRHHAWEFMLFNARPRICV

>CYP6188A-fragment4|1820013|Chove1

MVRNRDVYNAVIAELTGLKLSNPATWKELADAPLLHAIVKETLRLHPPAGFNLPRAVPAGGRTVCGHYLPEGTTVGMSAW

CVHANEDFWGKDTLEFKPQRWLDPETAFKLDRYGLSFGQGARACLGKNIAMVQLVKVCLSLLHFSAVY*

>CYP6470B-fragment2|338286|Chove1

MELSKLEKECPYLTACVKESLRRDPLVPGPNPRIVPAEGAVINGWELPGNTIIFAQTQTTNHDPKIFPNPTLYIPERWLG

EKEMAEMRAMMTTFGSGPRTCIGENLAMMELHCVTALLFRNYNVSIPDGYDNEYVEFWITRPRGEDVFLRIEPRGV*

>CYP-fragment7|796716|Kalpfe1

MAIFLFDPLTKVPGPFWARVTNWWSVYFSLTYKQHEVEHAVHKKYGHLVHYAPNISTFDDPKMLPIVYHRYADRILTSME

LTALIEEEDWLCSSYGRYLLSTDRGTNQKVFTTRSLNVNSLFNGESDPDRVQEFQAKMDDFAKGGGENGLAEWSHFFAYD

VISELTFGGNLVSWRRERISIIFSSSERHELARAELEGELEPARGFILLAAIR*

>CYP-fragment5|764598|Kalpfe1

MAMIEALFIATDTLRILKKSWVLLAALAITLKFAQIFLFDPLSKFPGPVSPIGGHLVRHAPNVLTFDYPKMVPIVYHRVQ

EFRAKMDAFAKAGGNIDFAEWSQFFTYDVISELTSGGEFGFMEKGKHFHNLVSGLAVGLPLAGLTMRINSLLTKKFIADK

VMPRLTDKDGIGAVMGYRNKLLDQRIKLGPVDGRRDILHHTMIAKNPNGTTITLEGWRGSDATAGTLRSIIYRTLQTPGV

YGKMIAEIDEAFNSGRISKPVVTYDECLGLPYFCACIKETLRLDPSGPTANPWVINCNKDFYGEDAEIFRSERWIESEER

TRQLENACFTFVYGARICLGKSRALMEVHKVILQLVDLDKHGLRVNYANCFQSGKETVNNCSTVVYCKRESVAIGKKWTG

E*

>CYP-fragment10|796717|Kalpfe1

MWMESGQETRQLENASFTFGYGARICLGKGIALTEVHKLIFQFPLNFRLESVDLDKHGPKVNIGNFFQRDMWVKVHARTP

KPSS*

>CYP6521B1-fragment1|622597|Morimp1

MVEKRKQFKPEKPDIFHFLLEAEPQNIGEHHIDLIGDAQLASVAGSDTTANTFINIFTQLANNPHVLQKLQHEIDAIYNA

QYDTDKGSVVPEVILNGNSATTRYLDSVINEGLRLNPAVPGGVQRVTPEEGVK*

>CYP-fragment1|1748|Pyrco1

MYEAAGYGREEIGGKVALVVFGYTTDMPYTAFLLCFHLFSRPELLEKVREKVKGKEVEGLQDCRLMKAAWLETLRVCTSA

HSVREVVSDTVVDGFLFKKGAMVMGVSRTPQTAPEMWGAEPEK*

>CYP6713-fragment1|95|Pyrco1

MQCIDAFLGQLRERGTVDMNDWALFLTFDSMGLSVFNQTYGMLKTGEWHPEVYKQKKNMSFLELGLTVPWLVRMKADIPK

RTTSVSRVLSWYYEQMDKKIAEFTAGKEPTDFAAMLLADKNYGYGKMHIDAHHDDCRLIILAGGFVPIRSRNSYGRR*

>CYP6713-fragment1|3236|Pyrco1

MESVKQQEAGTLLGQLSVGRIFVAIFAILAINIAHTIIKTRYFHPLSKFPGPFWASVSRLWITYHNLTGKEHEVLYDLHK

KYGPVIRITPTMLMCSDPKMLPVIYHSHADKADHYVTGSFGKTPSVFNIQPHKVHAAARRKIAQPYAFSAIKPMETLVDV

RIDEWTSKLEANFAAGNKKFDFAAWATVSFFAYDVISELAFGKALGFVAKGYDIDNLIRSFHEGLPAFGFLCRLHPFTKW

IKTTWIADKYMIPQPGDSTGIGNIMSFRDQLIDERIAENKANPNNDRRDLLQSFLKARDVKDGIHMDDLKAETLLVLLAG

SDTTATEFQAVMIDILKNPKVYERLMAEIDAAPLSRIPTYDEALEHCPYYIACVKEAMRLCPAAPNMFPRVVASGGLQLY

GKFAPEGTEITCNPYITHRNKEMYGEDAEEFRPERWLDNAEKVAEWEKHDFGFGFGSRKCLGQSIALMELYKAPLQFFRH

FRPRLVNEINPSRYIVAGGVSRHTDLWLTIEKRA*

>CYP-fragment9|3255|Pyrco1

MSEKAMVDEIRLIINAGIDTVSLVISLGMYFLTKHSRVYKKLQKLLDEKFPDRKFNHAKVISVEYLDAIIMEALRLYPPA

IAGTPRQIPKEGMSIAGRFIPGDVVVSVPPYTMNRHPDFWLEPDEFVLERWTAAGEHLAKDRAVCLPFSMGTYRCPGKEF

AMMQLRTLFSEVAMNFDVAFADPVQAEGYERGMKDYFTLYAPALCMKFSPR*

>CYP-fragment4|3460|Pyrco1

MPPGFLPTLSSSFSPLLWRLLGLYLVIYTVLIFTTSLFQATLIATSFNTTLLLSIGSHRIFFHRLCHFPRPFGASISRFW

LLYKLHKRHDRFRLQESLHKRYGPIVRFGVSHHARSKPWHRAFNGTNLLLAFPRVNKCITLFISQTKEKKTVDMNLWGLY

LAFDVIGEAAYERGFGIMETGIPHPACEMQRNAMGFFEVALILPWWERMVMSLPQDASTLGRAMR*

>CYP-fragment3|12859|Pyrco1

MYAKKVEDFNIERAPFEGLLGKKGVFTLTGSAWAHSRKLVRPAFEKATISDLFKTKILSLPADDAGYREIDLIEWFQALI

MDPAGHTLFGSSVGSIDSIGKSSLISKGSDIEMTYTEAFDFAQEVLAVQMALPLFMHFLVNSKRYKKACQKSKEEVMGYI

ERALENRVKGMKYDFLGVIVEQTQDREMIRDQVLALRVYPPVYINTRTPIRDSVLPFGGGKYGEAPFQVNKGERIVLSSF
ALHGNTTIYGKASREFRPERWLEDDSLRGIGWAYLPFGGGPRICPGQKMALITASFVIVRMLQQFENIAGDGRKQEEILY
EVKILMTIAGGFKIKLR*
>CYP505-fragment1|404|Pyrco1
MYDIASELVMKWARHGPEHRILAADDFTRLSLDSLALCTMNLRFNSFYKDELHLFVHAMSIPTLSFHCVDAWIERQVRGR
QNPKKDLLTCMVEGRVPKTGVIDNGLVVKNLITFLIVCNAGVNFDHRHDAPAFQFAI*
>CYP6713A-fragment1|3461|Pyrco1
MERKIKEFKAGEKPRDLASVLLADEKCGYGKLSMAAIYEDGRLIIGAGSETATGALTGALFYLHTYPRVLAKLRTILDAT
FTNGDEDYVYSFTHQIPYIDDIITELFRIQSPVADGLPRLTPKEGLVIDDIFIPGDVVVHVPIWVIQRQEMYYKKTEEFI
PERWSDQPELVKDKRAYMPFGLGL*
>CYP52AV-fragment1|912690|Sphbr2
MRLFGEEIVFTIEPRNIQAVLATKFKDFSLGNLRKEAFLELLGDGIFTLDGKGWEHSRAFAEAAIFEGAGLGRRGSEPVD
LQPWIFDLTLYSATEFLFGESANSLLMGHEKAGFADAYDSPRSRGFTRLAKGGEVIFQTH*
>CYP51041-fragment1|945656|Sphbr2
MFRRSSPGQSPTYCNVVFLACIDTEWQCLVSSGCGAQGKATASEKKPFYQYCQPPCQTCALIERQYQKPPPFAHNWVLKI
CGIACAAGILHTETKIFPNPTVFDPERWLGPDAVPDKCLSAFSRGPRQCFRISLAWCELYLALGTLFRLFEVELFETTEA
DLDYVAHFISSYKVGYVRAKVKFRASEGIIAAVV*
>CYP51-fragment1|789500|Terbo2
FMKFDLTSETFRAYVPLIVKNLRPTARTAHCSPLRRAPAAIPELTIFTASRTLQGKEIRKEITGEVAGLYHDLDAGFAPM
NFMFPWFPFLQNKRRDAAQRKKAQIYMDIITDPRQKSW*
>CYP51F-fragment2|850992|Terbo2
MRKVKSPMRVPNTHWVIPDNHYLMAAPGVSAIDGKYPRNPTAYDPYRGAGEKLTTEEEEEKFDSGYGLVSKGTASPYLPF
GAGRHRCIGPAPPDYTHPATMIYEWRKPEK*
>CYP50042A1-partial|1225360|Tercla1
IAGFGVQLFPQEMRWLVEPLLSFRNRRRIAFAAKKLTPIIEERKAIIERNSKDESSSPEEPDDLLQYLLHEAIGQGPPHD
SSYQVSCRYLLVNFAAIHTTSLTFSNSVANMAAYTDELTGCGYWDLLREEVEAVDRESEEGPGVWTKRKLNKLVGLDSFI
RETLRKNFSGPVGLVRKVMPKEGYTYANGLHVNHGELVGVPTLSVHIDDDNTGKQALDFIGFRYSRPYQELAGQAATDIS
ATGGIGKLAAVTTADEYLPFGHGKHACPGRFFGVIELKMSLKFCLLNYEIQPTKRAQTQYLWANPTPPFNIVIKMRKRRV
D*
>CYP5242B-fragment1|1270507|Tercla1
MAAVPIFKAEYLRWVEKLPEDKLVSYKRSDGSGFVIDALSACRKIPFKLIAMVLYRDMLTDKFFDELWELNEVHDRITFA
ALLRVLPAKKWYSFLPTRDNKLLEAYLKDWERLNMAVINEARATGTYCPVTEMYKGVEKGDMTLEQFLQSLDEILFTNVD
VTSTIFACVLINLARDREFQSALRQEILSYSHGLDAYITDDEALLHFAYLEALRVNPVAWFSIPETTGPDPKYIGSFLIP
PHTSVIIDLKLLNTQSPIWGSDGHIFRPLRWRGLSPSAVRYSFHRYGMGPRKCMGKNVANILIKMLMVTLLERYEIVADA
EGEGKYRTDKFTRAPEKEVEFRALLGKGGE*
>CYP6902-fragment1|701571|Tirniv1
MSFDILSALVRGYYNSEKERWQIQSKRFWKTIMILAGHETTANTIRYSIMMLTIHPELQIKLQGGINRILGDREPDYERD
YQARGGVNETLRFFSPVVNLPKYTKKPQPITFNKGGTLFLKEFIFISTPCCTPETKIMGSRGKTEEEAQPHKYIPERWLL
ETKRKEATDFGEDGLAPEGKDTSKAVQRLVHPIFRCVELEVKEAESWVDAKRRALGSMSRIVDLLLLCSQRASMLVVSG*
>CYP-fragment6|713727|Tirniv1
MIKELDDAYVAGRISKPVATYDECVTSALLLKESSDSTLPDQVSSPALLQREVTIEWTNFYGEDADVFNPQRWLVSEERS
KMMKNCGFTWGYGTRIFLGKNIALMEAHKAICQLFLHFAPKLVDLEQQGKRINLGE*

>CYP6648B1-fragment1|443126|Trihyb1

MTLLDYLTLNPAATAGTIFALVALIPSVWAYLASFRALSSIPTIGLSERRSWIDFWRARQRFVTDSLGLSIEGHKKYPRN
SVYKLWTPDGFKVMISPDLSGEITGAPDSVLNTHEAFQDSFFGKYTGIDANVDLRIKCVRVDLTKSLERKIPEIVEEANY
AIPLQIGECEEWTGFKLQPILLQIVALISGRLFIGPEKNRDPKWLNTAIQYTTDAFISAEYLRILPAFLRPLGARIIPEV
RRCGQHFTVVKEIIGPLIDAR

>CYP58-fragment1|874121|Trihyb1

MFESRIIDWSAGTAMAILAILYVVKIIYRLHFHPLRYFPGRTAAKITNLYEFYHNGYRGGKYLEEIVKAHQEFGPVIRIS
PNRPHFSDPEVYTFIYTSRPILVKDPSFYAAFGIPNGMGGTSDLTWH*

>CYP6855B-1-fragment|710307|Trihyb1

DDWPFERLVTEICTTSFAAIHTTSMTITTVLLELVARPEYQVSLRDEIRSMVASKGWTLEALDAMSQLDSFMLETRRFRP
LADIVLNRMCMQDTTLSDGTAITPGTYVSVAYSPRLLDGRYYSSPTEFDGLRFLKTEERFTDVDGAISLGFGAGKHA

>CYP6006B-fragment1|804466|Trihyb1

MGLASLLPSFLQFIAAMNINKDFKTIRKILIPVIQRRRLALPTKNDLIFFLDFILDAVPDDTRAADLVAVIVFGGLTNLQ
STFSSTVLDILNIPSLQSTLLPSLSQASASNINVFSPPPQSSPWSPLRAAMFESIRLCGPITGPARIIASPTTLSSDPKL
HLPKGQAATLSSFYTHRDPEMWGANAACYKYDRFVEGDPPIGMPEYIPWGLKGPHTCPGRWFAMTTIQVMTKELLVAYDF
VQDFVVKEEEKYIYSAGNVKRLEVGVEVTRRV*

>CYP6761-fragment1|860766|Trihyb1

MESIINPPEGVAKQKLPFVDVVEEVVIMVMGGTDSTASHLQFATWRFLTEPGVKEKVLAELDSVERDEHNRFQLNKLEAL
PYFSGFIKEVLRVYIIATARLPRIVPKEGLTIPSTGLYIPTGSSVTQYIGLLHHDPRIFEDPEIFKPERWIGNPGLDKWL
LTFSKGDRICIGMNLAYAEINFVLANLFTRFDLQLWNTTEEDMQWTDCGVAKPVGRIQVMAKKRV*

>CYP671K-fragment1|905154|Trihyb1

MTASKTINFHTACLDNLGVQQVIQAVIPHFDVDKLELRARAGTVVGAVECSNFYAFDVMGAVGSGKLWGMLGSGELHEAV
SQLHSAMVPLAVLRPVSWLLRLATDLPGANKPMQNFMNWCWNQLSEKKKNLDYEGKPKDVMTWILTDSMKPSDIAVNEDS
CLMIIAGSDTTDAALANALYFLLVNPHVYKKLQNILDDIFPGGDGDFDYSKASSIPFLDGIIHETLRLQPSVPAGLTRIT
QAEGLTIDDVYIPGDVVVNVPGHTIQRDERYYEKALEFIPERWTEEKAEMIKDKRAYAPFSLGTYGCVGKGLAMMELRMA
IADMSIKESEYYPEDHVHDLASHPLRFPQGMTL*

>CYP5268A-fragment1|919280|Trihyb1

MAGKNHIVLSTENREHIHFASDLLSHNLRPLVLPYNERCSSGLIFRIRFGKTVATGNEAYVRRILGVVHTLEHAASPEAY
LCDTLSILQYGFSDNEGAYAIRTMFRADSETTTSAMMSLLQCIVQYPEWQKRGQEDVDTVCGDHLSQFDDIPSVLGYHPI
TASVVPHILIKDDTYVGYFFAGTIVHANQWAIQNYPAFGFGRRICPGLNIAERSLYILTARRNFEAPEYEYTEGFNVKPL
SFYFGLVERNRRKVCEDVWEKAKEEWRRVG*

>CYP663-fragment1|5702|Tubae1

MSEHTAKSRNLRPLLLHDMHPHWGLARRLYTAFLSNTESTKYQQLQELESLRTIVDFLDRPDAFVKHVHRFAAPLMYKLC
YEKRVVTGEESEVVEIDQMLMKFVQAGEHGRWTVEAFPILEWLPKRFAGWKREGEQEAKKSEGDSWVKKVQGTILRKHYS
GTTLRGRNGHIIRNPRNLLFDCLKVPSFIPKAHATLDEICPDRVPKFSDLPNLPNITAVVYEVLRWRPAVIGGFAHATTR
EDEYHGYHIPVGAVVIPNHWGIHRDSAVYENPAESSPKRFMVDGVADGLKKLVAFGLGKRQGEFTHGFMSKPAGLDVHLS
PREGKKEIIMKEWEAVGQRGRGLNNSFFLNHV*

>CYP512CQ-fragment1|2812|Tubae1

MRELGAEWKDRPDDLIQWILESAGDGEASSEEELIARLLFLNFASIHTTTAAVVHALYDLAANPELQAPLLAEVTEILSK
SGMSKQSLTKMKKLDSVIRESQRLNTTSTISMMRKALVPYTFSDGTHLPIGTWVVAPAAAIHQSASIPNPTVFDGFRWER
MGAEDEASGKAGKHAAVATSFENLIFGHGKHACPGRFFAMQELKILLSHVVERFELRLGGAGGRPRNRFLGVSCLADREG
VVEFRMR*

>CYP567-fragment3|1128069|Tubbor1

MKRKLSHGFSVKALSEQMDIAYNYVDKLIRKINVHASGPKGDEMVKWYNFFTFDLIGDLACREAFGSLNDSMLCLFGVSL

LLGNVKAIAWRSVSRWLPVFDKLGVWTSPKSVMKMRIGHTEYS*

>CYP617-fragment3|1146549|Tubbor1

MSKSGKRIDCLITYFELKLVLICSTRDRPFSSSRTTGKPYFLRSSSTSSSGLSNLFQTQLVPRPTNSRFLISNFAVAQKV

RSQALQVIWVCTIPNSGPLRYLVISNIELAFSTKLEVLKVVLHPKGYGFSKLPRVTNEISNIPEKKGLLFAKGEKCRVER

KLLRPAFPHTHIKGLVSGFWSNRVEMSEKVAGVMRASQGLAGGWGRREVGSYEFRALESSSVSDSSNTEEKSGPELADAY

NTIFNMGGIPDRGDAIYDISLPPRAIPIAKASPRRCAARRLL*

>CYP617-fragment2|181475|Tubbor1

MRAIPNSGPLPRLGSPNIDRVFPPTPEALKVVLHAKDYAFSKLPRATNKTSNILGKKRLLFAKERKVQRKLLLPAFPHAH

IKGLVSGFWSNGVEMFENVAGVMRASQSGAGGWRKGRVGEMRELFSLVTLGKIGSYGFSYEFRVLESASISGISNTKEKS

GSRLADAYNTIFNMGGRLGS*

>CYP617W-fragment4|1118274|Tubbor1

MLSRLVAKRSVALWGAGAEEFWPGRSAGEEDAAAAVGSNYGFLIFLEEPRECIGDALAKVNLQCLLTATISRFEFEQGGK

REGLLRRGSPKIPRGYSYTVKGVV*

>CYP6136A-fragment2|1134625|Tubbor1

MLDGENRSMNNYIESGWFTFAVDGRVCIGRHLAMFMMKFTAAIIREFDIRVVKQPEEVHTLFNEMPGMEVLLSRRCDVCD

RMRVKVEG*

>CYP50026A1-fragment1|258645|Tubbor1

MDQATELLGQVEHLLLRPLHRSSLQALAAFLVWYVIYQRYFHPLRKFPGPFFASLTVFWRLSNILTFRQSLNDYALHKKY

GPVFRDGPNSLSVADPRALEPIYGTRNELNKTPWYLIMDPDNTGEDYSVFSSRKAEQHKRLKKRIASAVCLITLSSVDE*

>CYP52AV6-fragment1|279842|Tubbor1

MGAPPCAAQSSRVAAGRTGYRPCSYPKDRVDYTCYGLHRRKDLYGEDADAFRPERWEEGVGRGWEFLPFNGGRGFVSGVL

GRKLKIRKG*

>CYP617Y-fragment1|961463|Tubbor1

LLRYLGAFNTERVFFTTPEVLKKVLRVRSCGFIKRPLVTNDFGDILGKKGLLFAEGEEQRKLLLPAFSHAHIKGLVPGF*

>CYP5945C-fragment5|971827|Tubbor1

MLLLDPYHQATIAACAGFLTQQVLSPIPEVSTTKSILFYTIANGFLVLALKNAGQPLALLHYFVRISLINISYLTSLIFF

TIVHRLYFHPLRKFPGDKFARLSKLYEAWINYNGMNSPLVRDLCRKNGDFIRTGPNELAINNVEAIEIIWGRSQPTARGP

YYEFANFVGEKHLVSQRNKAIHASWRRIWFAPELSIF*

>CYP567Y-fragment1|1085838|Tubbor1

KIIQRMNMKITRRDLLSGQFGPNGPGMTIPELSGQARTIITAGSETTATFLSGTTYHLLKNPRVYNLLVEEILSAYSTYE

DITDTNAWRLKYLSAVMDEGFRIYPPIGAGIYRERMKKTVEPSTSSWSTCRSPENFHNPDEFKPERWLDPDCTDKKHASQ

PFSLGSRVYIGRNLALKEIRLVLSKMLWVYDMELVNKSLDLDRDSTSYVLWGKPEIWVRFARRRGAHVPLLDSE*

>CYP539A-fragment1|1088006|Tubbor1

MVFLGLDEIKRGLVAAKANKDMELWQNRFKIAGRHTVETTVINVRVILTEDPENIRAVLASQFHDYGKGESFNREWKSFL

GDGILTTDGQLWQASRQLIRPQFIKDRVSDLDTFERHVSHMLEHIPRDGATVNISNLFYRFTLDSATGFLLGQSIDSLGS

PQAELARAFADIQEHMTNRSKAGPLRWMFPEGKYKKDLKVLNSFVEPYVEQTLKMRPEELKNKKEKSYNFLHALAEFTRD

KQMLRDQIVSVLLAARVVICLFALV*

>CYP50026A1-fragment2|1115955|Tubbor1

MTQILATNLLENGNTTGKRNMSHDLLQCRNDDKTPLSKKELIGEMLAFTTAGSDPTAYEISAILDRICRHQEVREKLLQE

LRGVGELEQSSTDGVVTYAQTLRLPYFLAVVKETMRLNPSFQGQFSRVSPEGGGVGGGGLEVLPGVVVPGGVWLSVNTYV

SQRDKNIFGEDAEEFKPERWLPIGGDRYRAMSKYLSVFGYGSAACMGQHLALQKINKTVVEILRRFNMELRDPKTPLKEK

NIIQMFISDLFMTFTEREDKI*

>CYP6136A-fragment3|1134626|Tubbor1

MTREHYPQRNEAEGTTIQIFNLIAGALGLVPHTQVRVVHKLSRHPEIMQKIREKLLKTDNTFRLDDSPRYNCRKNKYLIF

ESAVRETIRLHPAVSFSLSREVPPSGCQLHQYHIPPGYNVGMASYHVNYDEG*

>CYP6136A-fragment1|1134631|Tubbor1

MPSASSDKIEDEIPVIPNTPQNIPSHMDIATLDLSETLPVHQAHVTLGMPGPKLTMRRSIQLSPAGAKLGPNIFLGRPCA

PERSAFIPIPEVILPLGNPGRVLYVTSRAIRGNIFLNALTTLYPNVLLLPTITFETGTADLKTLKEKSPRHLLSEFYNSP

DNTFRLEDFLPYNGGENKYPIFESAVRETIRLHHVVSFSLSREVPPSGCQLHQYHIPPGYNVGMASYQVNYDEGYFGLDV

AQFRPERWLEDHPTAMLDGEKRGMKNYIEAGWFTFGAGGRVCIGRHLAMFMMMKFTAAIIREFDTRVVKKPEEVHTLFNK

MSGMEVLLSRRCAV*

>CYP567Y-fragment2|1136429|Tubbor1

MRDRGVELSTSSWSACRSSENFHNPDEFKSERWLDRNSTDKKHTSQPLSLGSRVCIGRNLALKEIRLVLSKMLWVYDMEL

VNEDLDLDRDSTTYFLWIKPEIWMRFTRRQGVQVLLELAQKRSESKHKVMGPMNKRKKEIDVILLVLPIDAPETGVLIAV

RSMNIHQYDSSHFLVPHVPSSLATGRTYLYCMSH*

>CYP6136A-fragment3|368160|Tubbor1

MRGHYPQGNEAEGTTIQIFNLIAGALGVVPHTQVRVIHEVSRHPELMQNILEELLKTDNTLRLDDFLPYNGRENKYPISE

SAVHETIRLHPAVSFSLSREVPPSGCQLHQYHIPPGYNVGMASYQVNYDEAYFGPDVAQFRPERWLEDHPTAMLDGEKRS

MKNYIEAGWFTFGAGGRVCIGRHLAMFMMMKFTAAIIREFGIRVVKEPEEVHTLFNEMPG*

>CYP6188A-fragment3|922250|Tubbor1

ISSLAAPDTASVALRSILINLVRNRDVYNAVMAELTSLKLSNPATWKELEGAPLLHAIVKETLRLHPPAGFNLPRAVPAG

GRTVCGHYLPEGTTVGMSAWCVHANEDFWGKDTLEFKPQRWLDPESAFKLDRYGLSFGQGARACLGKNIAMVQLVKV

>CYP6188A-fragment2|925532|Tubbor1

PLKDVPGPWLASCSPLYRFWYAVVKGNFHNDLTNLHRQYGNVVRIAPNEVSIWDPRAVSEIYAHGDKGYAKSLLKLPRYD

IALPNGFFNLAVERDIQTHAEGRRAIAKDYNMTATLMAEAHFDNVIKDFILALDRNFAQKGGVCNFTIWSEYFTYDMITD

LVFGEAY

>CYP539A-fragment2|990034|Tubbor1

EVLERLGPNGKPTYTDLKEMRYLQHVINETLRLYPAIPFNLRRSLKDTYLPRGGGPDGLDPVGMPKNTIFLCSSLTLQRR

EDLFGPDADKFDPDRWEKW*

>CYP567-fragment2|1141501|Tubbr1_1

MSPVHASYLYTLVIGVREIAAFLAILGAVFVLGRAFYNVLLHPLSKIPGPKLCAATDVRSYHPPPLLQSINHPLLKSAQN

TLQGFSAKALLEQEDIVQGYVDKLIGQINVYATGPEGDEMVKWYNFFTFDLIGDLAFGESFASLNDAKPHFWVSLLLGNI

KAVAWWSVARWFHLFQILGVWTAPKSAMEMRINHSEYSRKMIIQRMNTKSTRRDLLSGQFGADGPGMTIPELSGQASTII

TAGSETTAIFLSGTTYHLLKIPRVYRLLVEEIRSAYNTYEEIADTNATKLRYLSAVIDEGLRIYPRRDRHVKLSASPWST

CRSPENFHDPEEFKPERWLDPHCTDKKHARQPFSLGSRPCIDRSLALKEIRPVLSKVFWAYDMELVNKDVDLNRDSTSFV

LG*

>CYP5945C-fragment3|890801|Tubbr1_1

MFLLDPYHQATIAACAAFLTQQVLSPIPEVSTTKSILFYTIANGLWVLALKRAGTCVLHLLIRVSLINISYLTSLTFFTV

VHRVYFHPLRKFPGGRVARLSKLYEAWINYNGMNGLVVRDLCRKHGDFIRTGPNELAINNVEAVEIIWGRTQPTARGPFY

EFANFIGERQLVSQRNKAIHASWRRIWFAPRLLKI*

>CYP6135A-fragment1|381490|Tubbr1_1

MGLTACTFPLGGRDSITLPPGVEFWVELIGLSHNPKYWIQRGETEGESAISEFRPERWLPKSGGDGMFKPYNGSYIPFSI

GNRACIGKKFAHVEFAAVMIGLFREMSVEFDLCGGKKSFEEARRECLMQMESGKALGATSAFAE*

>CYP50026A1-fragment1|878333|Tubbr1_1

MDQATELLSRVEHILLKPVHHSSLQALAAFLVWYVVYQRYFHPLRKFPGPFFASLTIFWRLSNILTFRQSINDHALHKKY

GPVFRDGPNSLSISDPRALEPIYGTKNELNKTPWYLVMDPDNTGEDYSVFSSRRAEQHKRLRKRIAGTYSMTSVRVYEPV

IDRNINDLLAWIKELKTLDVSVWTHYFAMDSSMLLCS*

>CYP50026A1-fragment2|939445|Tubbr1_1

MTEALATNLLENGNTTGKRNMSHDLLQCRNDDNTPLSKKELVGEMIAFTTAGSDPTAYEISSILDCICRHGEVREKLLQE

LRAMGELEQSSAEGVVTYAQTLRLPYLLAVVKETMRLNPAFQGQFSRVSPEGGGGGGGGCGEVGGLEVLPGVVVPGGVWL

SVNTYVSQRDKLVFGEDAEEFKPERWLPIGGDRYHAMAKHLSVFGYGSAACMGQHLASQKINKTVVEILRRFNVELRDPK

TPRKERNIIQMFISDLFMTFTEREKKI*

>CYP-fragment8|639150|Tubin1_1

MPATITYDSISSLKYLSHVMPETLRLYSSISMMLHVDAHDASLIGAFVLKAISIMLPMFAVNRSVEMCGYDAEGRGPGLW

AKVELKYQLSAVIGVHSLGGGTRR*

>CYP5945C-fragment4|317779|Tubin1_1

MIHELRAGLVLENQDRSKKRPTDGADTENELAASIFNRQGLSRSWSYDPYRQATIAACAAFLTQQVLSPIPEVSTTKSIL

FYTIANGLLVLALKRAGTCMLHLLIRVSLINISYLTSLTFFTIVHRIYFHPLRKFPGSKVARLSKLYEAWINYNGMNGRV

VRDLCRKHGDFIRTGPNELAINNVEAVEIIWGRTQPTARGPFYEFANFIGERHLVSQRDKAIHASWRRIWFAPRFSNI*

>CYP6188A-fragment1|638288|Tubin1_1

MIFWIGYFDRGIEMDIPSPWGRLRTFPSSLICDYKLTFFSRNHAYIFMYHTFPPCIFSKANPSSLAAPDTASVALRAILI

NLVRNRNVYNEVMAELTGLKLSNPATWKELAGAPLLHAIVKETIRLHPPAGFNLPRAVPAGGRTLCGYYLPEGTTVGMSA

WCVHANEDFWGKDTLEFKPERWLDPERAFKLDQYGLSFGQGARACLGKNIALVQLVKVYLSLLYLRSLPLFPVE*

>CYP6188A2-fragment1|462774|Tubin1_1

TPLKHVPGPWLASCSPLYRFWYAVLKGNFHHDLTNLHRRYGDVVRIAPNEVSIWDPRVTSEIYAHGDKGYAKCDMYDIAL

PNGFFNLAVERDIQTHAEGRRAIAKDYSMTTTLMAESHFDNIIKDFILALDKNFAQKGVVCDFTIWSEYFTYDMITDLVF

GEAYGFCKTGLDVDGSLKDLRQMLLLSPFL

>CYP617-fragment1|175385|Tubma1

GAGMRGEIVEMGRWYSLVTLDLVASSGVDYELRAPEGASINGGSNTEERLGDTYNTISNTGSPSQIMATLSLILPSRLVQ

FLSLKRTHDIARAALTIKRVTAQIIAIIAAGKSTLATSPEGSRSGDTLSGMLKSNTHTGPDGESGMRDQMMTFLAAGHET

PATSITWTIDALYLRENRHIQPRPRAEIHATFPSGTPATIAYDQLGDEFLVYFVNIVYY*

>CYP5945C-fragment2|211607|Tubma1

MSLLDPHCQATIAACAAFLTQQLLSPIPEVSTTKSILFYTIANNLLALALKRAGINILLHFLMHISLINISYLTSLTFFT

VVHRLYFHPLRKFPGGKIARLSKLYEAWINYNGRNGPVVRDLCRKYGDFIRTGPNELAINNVEAVEIIWGRTQPTARGPF

YEFANFIGERHLVSQRNKAIHASWRRIWFAPRVLKFWNLFTDFD*

>CYP50320A-fragment1|316251|Tubma1

MMKVFARQLRVFTITNTENAASVGELLLGENGGVDVSHEAKSGEGLNLPDKYPQSEGGDGTDMSDGQPNVRATSLLATAS

DTTDRVSIPLLKNILGNSQVCRNLVEGMNSATSSSFFIPATPSTRPKESLSYLHACIKETLRHSIPSSINARVVPNGGYE

ICGRYIPGGTRIFLSPWAASCDKGVYGEDADDFNPERWIRASKARAREFERRSVVWGYGDFMGAERGIVLAGVYKANLLF

FREFEAEIICPQARRGLWSSGLGVRIKRRGPC*

>CYP50026A1-fragment1|351219|Tubma1

MGLTVCWFMQQQQQESVVFIATMGYLTGLNHVMKSKWLSPYLAPSPKDKHGYGHMIGMTQALATDLLESGNTMGKRNMSH

DLLQCRNDDNTPLSKRELIGEMLAFTTAGSDPTAYEISSILDRICRHGEVREKLLQELRGVGELEQSSAEGVVTYAQTLR

LPYFLAVVKETMRLSPSFQGQFSRVAPEGGEGLEVLPGVVVPGGVWLSVNTYISQRDKLIFGEDAEEFKPERWLPIGGDR

YHAMAKYLSVFGYGSTACMGQHLASQKINKTVVEILRRYNVELRDPKIPLKEKNVIQMFISDLFMTFTERENKI*

>CYP567-fragment1|4154|Tubme1v2

MKLKLSHGFSAKALLEQEDIVQGYVDKLIGQINVYATGPQGDEMVKWYNFFTFDLIGDLAFGQSFGSLNDAKPHFWVSLL

LGNVREIAWWSVSRWFPLFENLGVWNPPKSAMEMRIKHSEYSRKMIIQRTDTKTTRRDLLSGQFGPNGPGMTVPELSGQA

STIMYHHYPLAFC*

>CYP51070A-fragment2|8058|Tubme1v2

MTAKLSSLVPEIVDEIKNAFEDLTDIKNENMLIVLVGLPLCRNNEYIDSITQFSINVAKTANIVDLLPWFFRRTAVRFFL

RKNTSENILMKYLAREFETRKMTKRSTTGSPVSIISDAIQWILDATSLETPIKKLVQRLMFFNFASIHPTSITLAHTLYD

IAANPEIHDSIGTEIEEVLRAEGGWTKQGLTKMKKLDSILRESSRINGINLFSVIRKVLTPYTLIDGTHVQKGDWKIMRI

QKPLMGLDFIKCDKKRVKHISTRWLVQP*

>CYP51070A-fragment1|8060|Tubme1v2

YGKYGKAFRTTSPTKDTIVFTNQEVIEEIRNLPETILSFRPAGDELIQTTYTFHEGIFQDDYHIKIIRKNMTEKLSKLLP

EISDEAENAFEDLVNVKNKWTNVNIFNISAGLISKKLIYTIVGLPICRNQEYLQSITQFSINIIQFVRIMNFLPWFFGRI

VARFILKKHIVQDVLRKHLLDASLRQGKERKDC*

>CYP617W-fragment2|503|Tubme1v2

MGKWCSLVTLDIIGSSGFGYEFRALESASVSGSPEEKSGSELADAYNTIFNMGSPSRIVAILSLIFPAFLVQSLPLKRTR

DVAHAASTIRRVTAQIIAAKKSALATSQEDAESKDILSVMLKSNAYTGSDGESSMRDQMMTFLAAGHETTATSMIWAIHT

LSLPENRHIQSRLRAEIHAAFPSGAPATITYDQLSSLKWFGGEFLGYFVNIVYY

>CYP6498A1-partial|7035|Tubme1v2

MYERRNDFPKATKVYEYVRFYGDNVLTLGGADWRRHNKYTRPPFNETVNKVVWDEGIKQAYAAVNVWNRKNSCENVGRDL

RTISMNVLSLSNFGVALPFDHESETAEHFGRNIPPGHKMSYGKAVNHVLDNIIPLVISPKWLLRNGPESLKKIGRSYDEL

GIYLKELTQIDNKAAASDRKNLLGSLAKASAGDGPDKGSGLADVEVIGNAFIFAVAGLETTTGTFHYAIMHLALNPDVQD

WLYEDLQEALKDEDQDPSKWDYEKVYPKMAAVLCVIDTDTRKHETLRVNTPHMHIPKWTADKYQPVD*

>CYP51084-fragment|561887|Wilmi1

MRIPSSLAASLLAVATQIALSPIQEVSTLKVLYGWIIVDLLLFVLFIKTHPISAAILHLIRFNTIYSTTLILAAGIYRLF

LHPLRNFPGPKLAALSKLNEAYLQWQGRNGLEIRALHQQYGDFVRVGPNEIAINNVDGLKLMTRKPFNNRGPVNEHAQLA

GDVHLQNERDAGVHLKWRKIW*

>CYP6713-fragment1|643236|Wilmi1

MLLHLLGISSLAAVGFHLTTLRHELDFYLFHLIGLYIAALVGIAWSLSQTTSVLLAILDTAVIAVSFNTALATSILTHRV

FLHRLGKFPGPLVARISKFWMVKKVWGNSQGYIVIDKTHQKYGDFIRIGPRELSINPPTPCKSSMPPHPAALNLPSMAQL

GLTTTSASSQFGIRNSMRTAGLPGTELSMVQRSYHIAINCSAYIATPRSA*

>CYP6713-fragment1|643235|Wilmi1

MDDVRLIIIAGSDSLAVTISAIMYYLASNPAIYKKLQSILDSIFPGGDSEYSYTKAASIPYLDAIINEALRLQPPAISSL

PRVTPTEVVSVPPYTVNRDPRYWEKPAEFIPERWTDEKPELIKDRCVYTPFTIGIYQCPGKSLAYMEIRMVIVRIALSFK

VAFVDDDDSV*

>CYP548BE8-fragment1|654682|Wilmi1

MGERFSWLTFDVMGELCFGKTFGMLTDETQRFVTDLISKATHNNHYICGNYLPIRFMGLGRLLFPTIAKDRWRFIEHSRAC

ANERMKLDHAAKKDFFYYLLGAKDPETGKGFSTKELWGEANVLMIAGSDTTATALSSTLFHLCRNPSTMTKLQHEIRSAF

SSPDDIVTGKELADCTYLKACIDEALRMTPPVPGLLPRETVAPCKIDGCELPEGTGIGVPIYTIHHNEEYFPEPYEYRPE

RWLQGDEAALEKAKTAFNPFSLGARGCIGKSVAYMELRLAIAKLIWGFEMQQVSVEGKAEQWWEGFAVKEGEYRLMDHFT

CKKEGPVVAFQERKVEESQTSENAINSQTNLTQPIPLQIPP*
