# Supplementary figures and images for: Pezizomycetes Genomes Reveal Diverse P450 Complements Characteristic of Saprotrophic and Ectomycorrhizal Lifestyles
Source: J Fungi (Basel). 2023 Aug 6;9(8):830. doi: 10.3390/jof9080830 (PMC10455484; doi:10.3390/jof9080830)

Tree scale: 0.1

**P450 Families**

- CYP52
- CYP6001
- CYP5959
- CYP567

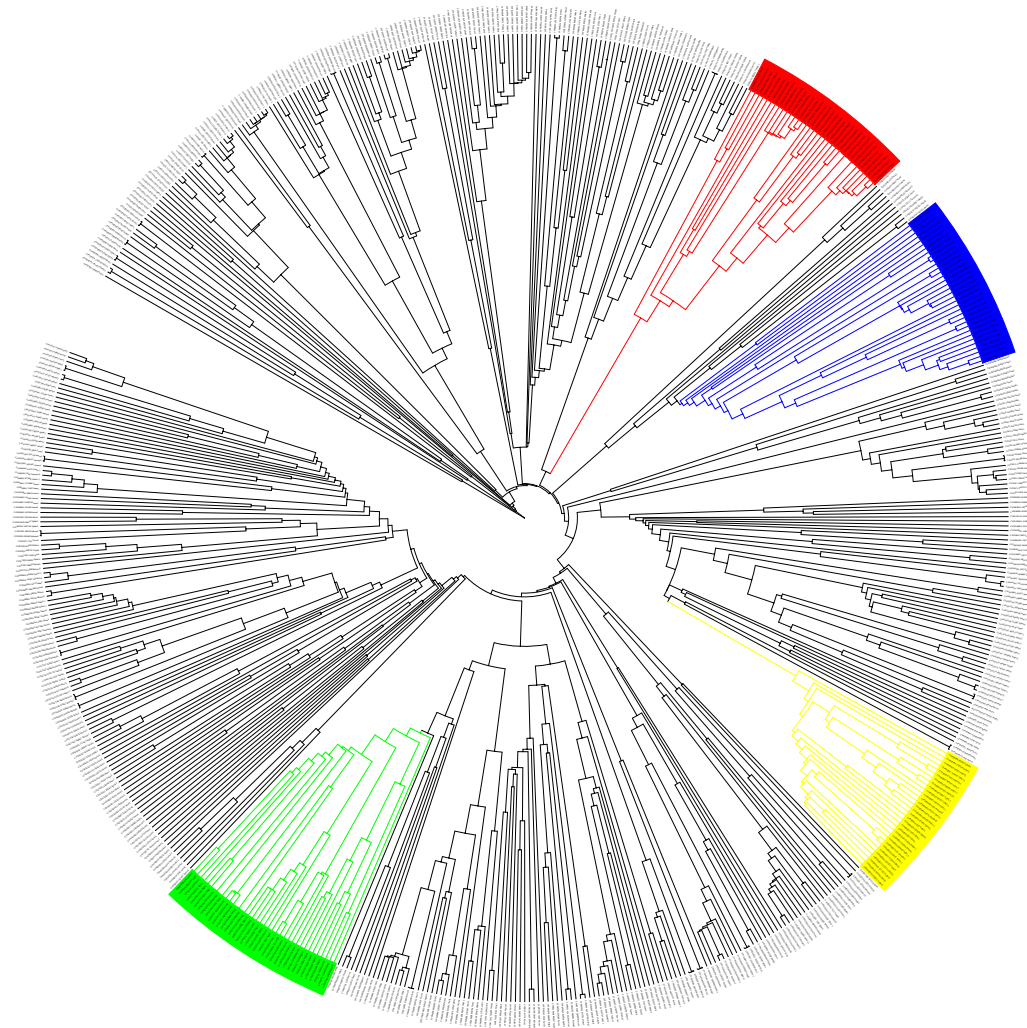

Supplement: Supplementary file 1 [file jof-09-00830-s001.zip › Figure S1.pdf]
